# Analysis of oat seed transcriptome with regards to proteins involved in celiac disease

**DOI:** 10.1038/s41598-022-12711-6

**Published:** 2022-05-23

**Authors:** Leona Leišová-Svobodová, Tereza Sovová, Václav Dvořáček

**Affiliations:** Crop Research Institute, Drnovská 507, Prague 6, Ruzyne Czech Republic

**Keywords:** Molecular biology, Plant sciences

## Abstract

Oat (*Avena sativa* L.) is considered to be a healthy food. In contrast to other grain crops, oat is high in protein, lipids, dietary fiber, antioxidants, and uniquely in avenanthramides. The question of whether it can also be consumed by people suffering from celiac disease is still unresolved. The main aim of this study was to extract and sequence genes for potentially harmful avenins, globulins, and α-amylase/trypsin inhibitors in six oat varieties and to establish their variability using PacBio sequencing technology of enriched libraries. The results were compared with sequences of the genes already present in databases. In total, 21 avenin, 75 globulin, and 25 α-amylase/trypsin inhibitor genes were identified and mapped in the hexaploid oat chromosomes. In all of the three gene families, only marginal sequence differences were found between the oat varieties within the individual genes. Avenin epitopes were found in all four types of avenin genes occurring in all oat varieties tested within this study. However, the number of avenin genes was nearly four times lower than of globulin genes and, on the protein level, formed only 10% of storage proteins. Therefore, the question of whether oat is safe to celiac disease people is a question of boundary values.

## Introduction

Celiac disease (CD) represents a complex of human body responses to gluten which is connected to the inability of human small intestine hydrolases to cleave peptide bonds in gluten proteins from the prolamin family^1,2^. CD is determined genetically by the presence of HLA-DQ2.2, HLA-DQ2.5, or HLA-DQ8 haplotypes of receptors on the plasma membrane of dendritic cells (DCs) which are important antigen-presenting cells^3^. The enzyme tissue transglutaminase 2 (tTG2) plays an important role in CD biogenesis because it deamidates glutamine residues to glutamic acid in uncleaved gluten peptides thus forming gluten epitopes with negative charge^4^. These epitopes are recognised by positively charged HLA-DQ2/8 T-cell receptors^5^. The interaction leads to T-cell proliferation which initiates inflammatory processes^3^. (Haboubi et al. 2006). Globally, 1.4% of individuals are reported suffering from CD^6^. The only lifelong efficient treatment of celiac disease lies in consumption of gluten-free diet.

The oat (*Avena sativa* L.) is the most commonly cultivated member of the *Avena* genus. It is an important cereal crop for both feed and human consumption with global production that ranks sixth among cereal crops^7^. Increased oat consumption is often promoted due to nutritional attributes including antioxidants, such as avenanthramides, and high soluble fibre^8^. The protein content in the oat grain is high (15–20%). The majority of oat proteins consist of globulins (85–90%), in contrast to wheat in which the vast majority of proteins consist of gluten including glutenins and wheat prolamin peptides called gliadins^9^. In oats, the prolamin peptides, which are rich in proline and glutamine, are called avenins and exist as monomers and disulphide-linked aggregates^10^. Avenins make up only 10–15% of the total oat seed protein content. Little is still known about the number of genes coding for avenins. While Gilissen et al.^9^ expected a maximum of ten genes occurring in the hexaploid oat genome, Chesnut et al.^11^ estimated that there are 25 avenin and 50 globulin genes per haploid genome in oat. Comino et al.^12^ identified 16 reactive proteins on the western blot.

Proteins of the prolamin family in wheat have certain aminoacid sequences that act as epitopes for CD. They resist degradation in the gastrointestinal tract due to high content of proline and glutamine. Oat prolamins—avenins—do not contain the known CD immunogenic epitopes from wheat, but the T-cells recognize four avenin specific epitopes DQ2.5-ave-1a, DQ2.5-ave-1b, DQ2.5-ave-1c, and DQ2.5-ave-2 with close sequence homology to barley T-cell epitopes that are immunotoxic in CD^13^. Antibodies developed against epitopes extracted from wheat gliadins were used by Balabio et al.^14^ who found differences in gluten content between 36 oat varieties ranging from 3 to 80 ppm. Comino et al.^12^ studied avenins also on the protein level. They observed significant polymorphism patterns in avenin proteins of Spanish and Australian oat varieties separated on 1D SDS-PAGE gels which ranged from 20 to 70 kDa.

Sequence analysis of avenin protein sequences revealed four avenin groups called A, B, C1, and C2^15,16^ with a molecular structure analogous to other prolamins. The protein sequences contain three conserved regions interspersed by two repetitive regions with lower proline and glutamine content when compared to other prolamins, especially wheat α- and γ-gliadins^10^. Thus, it can be expected that avenins are more easily hydrolyzable by duodenal enzymes than other prolamins and therefore its consumption should be safe for CD patients^9^. However, recent studies cast doubt on the safety of oats associating oat consumption in some CD patients with inflammatory symptoms^5,17^. Regardless of possible contamination by wheat, barley or rye, contamination-free oat varieties differ in their capacity to induce inflammatory response in CD patients^18^. Moreover, there are other compounds such as α-amylase/trypsin inhibitors (AATI) in oat seeds that can also contribute to the intestinal inflammation via activation of innate immune pathways^2,19,20^. On the one hand, oats can form a healthy, nutritious, fibre-rich, and safe complement to the gluten-free diet^9^ and on the other hand, its consumption may present a risk for CD patients.

The main aim of this study was (1) to identify all avenin, globulin, and α-amylase/trypsin inhibitor genes present in selected oat varieties and to map them on the oat chromosomes; (2) to investigate differences in oat avenin, globulin, and α-amylase trypsin inhibitor composition in six oat varieties with various level of reactivity. As oat is an allohexaploid (2n = 6x = 42), and its genome is large and complex with 2C = 25.7^21^, we employed NGS technology to explore the oat seed globulin- and avenin-enriched transcriptome. Seed transcriptome has been already investigated by Illumina technology, but only with respect to the avenanthramide and tocol pathways^22^. To the best of our knowledge, this is the first study that uses NGS approach to investigate oat avenin, globulin, and α-amylase trypsin inhibitors.

## Material and methods

### Plant material and sampling

Six oat varieties were selected according to results of previous internal screening using immunosorbent assay (ELISA) monoclonal antibody G12. A group of oat varieties with higher reactivity was represented by ‘Sirene’ (FRA; 309.4 ppm), ‘Atego’ (CZE; 17.2 ppm), and ‘Poncho’ (FRA; 9.4 ppm); ‘Dalimil’ (CZE; 2.7 ppm), ‘Jim’ (USA; 2.0 ppm), and ‘Ebene’ (FRA; 2.7 ppm) formed a group of non-reactive oat varieties.

All six varieties were planted in a field experiment. Developing hulled seeds were collected at 21 days after anthesis (DPA) because avenin expression levels have been proved to peak between 20 and 28 DPA^10^. All samples were frozen in liquid nitrogen and stored at − 80 °C until RNA extraction.

### RNA extraction

Pooled samples of approximately 20 seeds of each sample were used for RNA extraction using the TRIzol method (Invitrogen, Carlsbad, CA, US) according to the manufacturer’s instructions. RNA was then purified with the RNeasy Plus Mini Kit (Qiagen, Hilden, Germany) following the standard protocol and treated with RNase-free DNase I (Qiagen, Hilden, Germany). The quality and integrity of the RNA was determined electrophoretically and spectrophotometrically with a GeneQuant *Pro* spectrophotometer (Biochrom, Cambridge, UK).

### Avenin, globulin, and α-amylase/trypsin inhibitor transcriptome enrichment

To enrich target sequences, the first strand of cDNA was created using reverse transcriptase and oligo dT. The second strand was then synthetized using biotinylated probes specific to avenins or globulins (Table 1). cDNAs with ligated probes were selected using streptavidin bound to magnetic beads (New England Biolabs, Ipswich, MA). Target sequences were then amplified by PCR with Avena primers (Table 1) and Pfu proof reading polymerase (Fermentas, Lithuania). PCR products were purified using QIAquick PCR Purification Kit (Qiagen, Hilden, Germany). Avenins, globulins, and α-amylase/trypsin inhibitors enriched sequences were put together for each oat variety. This process was repeated three times for each oat variety separately and the respective three subsamples were then pooled. Quality and concentration were verified using Qubit^®^ dsDNA BR Assay (Thermo Fisher Scientific, Waltham, MA, USA).

**Table 1:** Primers, oligos and probes used in the study.

Primers/probes	Sequence (5'-3')	Usage for
Avenins-S	Biotin-GACTGCGTACCATGARGAMCTTYCTCATC	Target sequence selection
Globulins-S	Biotin-GACTGCGTACCATGGYAAYYAYYRGBWTBYSATC	
Trypsin-S	Biotin-GACTGCGTACCATGGCGTCC	
Avena-F	GACTGCGTACCATG	Target sequence amplification
Avena-R	GATGAGTCCTGAGTTT	
Oligo-dT5	GATGAGTCCTGAGTTTTTT	Reverse transcription
Oligo-dT12	GATGAGTCCTGAGTTTTTTTTTTTTT	
Oligo-dT13	GATGAGTCCTGAGTTTTTTTTTTTTTT	
Oligo-dT14	GATGAGTCCTGAGTTTTTTTTTTTTTTT	
Oligo-dT15	GATGAGTCCTGAGTTTTTTTTTTTTTTTT	
Oligo-dT16	GATGAGTCCTGAGTTTTTTTTTTTTTTTTT	
Oligo-dT17	GATGAGTCCTGAGTTTTTTTTTTTTTTTTTT	
Oligo-dT18	GATGAGTCCTGAGTTTTTTTTTTTTTTTTTTT	

### PacBio sequencing

Libraries of target sequences were prepared using a SMRTbell Express Template Prep Kit 2.0 (Pacific Biosciences, Menlo Park, CA, USA). Libraries were validated and quantified using an Agilent High Sensitivity DNA Kit (Agilent, Santa Clara, CA, USA). All sequencing reactions were performed on the PacBio Sequel System with the Sequel Sequencing Kit 3.0 (Pacific Biosciences, Menlo Park, CA, USA). The samples were sequenced in SMRT Cell 1Mv3 with 500,000–600,000 of reads with a 10-h collection. Sequence analysis was performed with SMRT Link v6.0 using common SMRT pipeline providing raw sequences and general run statistics. Data quality control was performed using FastQC (v.0.11.5; http://www.bioinformatics.bbsrc.ac.uk/projects/fastqc) and MultiQC (v.1.6.dev0; http://multiqc.info) tools. All sequence files are available in NCBI Sequence Read Archive: PRJNA774959.

### Data evaluation

The alignment of single-end reads was performed using Minimap2^23^ with default parameters for sequences obtained by PacBio technology. As a reference, PepsiCo OT3098 was used (https://wheat.pw.usda.gov/GG3/graingenes_downloads/oat-ot3098-pepsico). Files with aligned reads for each sample were converted to bam format, sorted, and merged using SAMtools^24^. Only mapped reads were selected and converted to fasta format in SAMtools. Mapped transcripts were searched (blastx, 1E-10) against the non-redundant protein sequences at NCBI. The resulting BLAST hits were processed by Omicsbox software (v.2.0.10; https://www.biobam.com/omicsbox) to retrieve associated GO terms (http://www.geneontology.org/) describing biological processes, molecular functions, and cellular components. Positions and intervals of selected mapped transcripts were transformed to bed format. The reference was then reduced in BEDtools (v 2.30.0; https://bedtools.readthedocs.io/en/latest/) when extracting sequences from the reference PepsiCo OT3098 v2 for intervals defined in the bed file.

Reads of all samples were realigned using the reduced reference in minimap2 software. Sam files were converted to bam files, sorted, and indexed using SAMtools. After variant calling pipeline carried out according to the best practises workflows (https://gatk.broadinstitute.org/hc/en-us/categories/360002302312), consensus sequences were extracted using SAMtools^24^ and bcftools (http://www.htslib.org/doc/bcftools.html). The reads were then visualized with the Integrative Genomic Viewer (v. 2.8.10^25^). Phylogenetic analyses of selected target sequences were done using the Maximum Likelihood method based on the Jukes–Cantor model^26^ in the MEGA tool package (v. 7^27^) with default parameters.

### Research involving plants

Authors confirm that all methods were performed in accordance with the relevant guidelines and regulations. Oat seeds were provided by breeders collaborating on the project QK1810102.

## Results

To compare avenin, globulin, and α-amylase/trypsin inhibitor composition differences among the oat varieties, six target enriched DNA libraries derived from RNA samples were prepared. The enrichment was performed using hybridization by specific degenerate probes, selection of caught molecules, and their amplification. Samples were sequenced using the PacBio platform in a SMRT Cell. After removal of the primer adaptor sequences and low-quality reads, 436,000 single-end reads of 1027 nt on average in length were obtained. The results are provided in six separate files, each containing from 61,329 to 95,485 sequences (NCBI Sequence Read Archive: PRJNA774959).

All sequences were mapped to the oat reference (PepsiCo OT3098), merged, and extracted using SAMtools. After skipping duplicates, 56,394 sequences were obtained in total. These sequences were blasted and annotated (blastx, 1E−10) against SwissProt database. Matches were found for 38,867 sequences (68.9%). Functional classification of blasted sequences was performed through a gene ontology categorization. An ontology annotation was found for 32,925 of the sequences (58%). In total, 14,031 sequences were annotated as avenin, globulin, gliadin, or glutelin. After merging overlapped loci, 213 contigs were defined in a bed file and used to prepare avenin, globulin, and α-amylase/trypsin inhibitor target oat reference (Reference_OT3098.fasta; Supplementary File S1).

**Figure 1:**
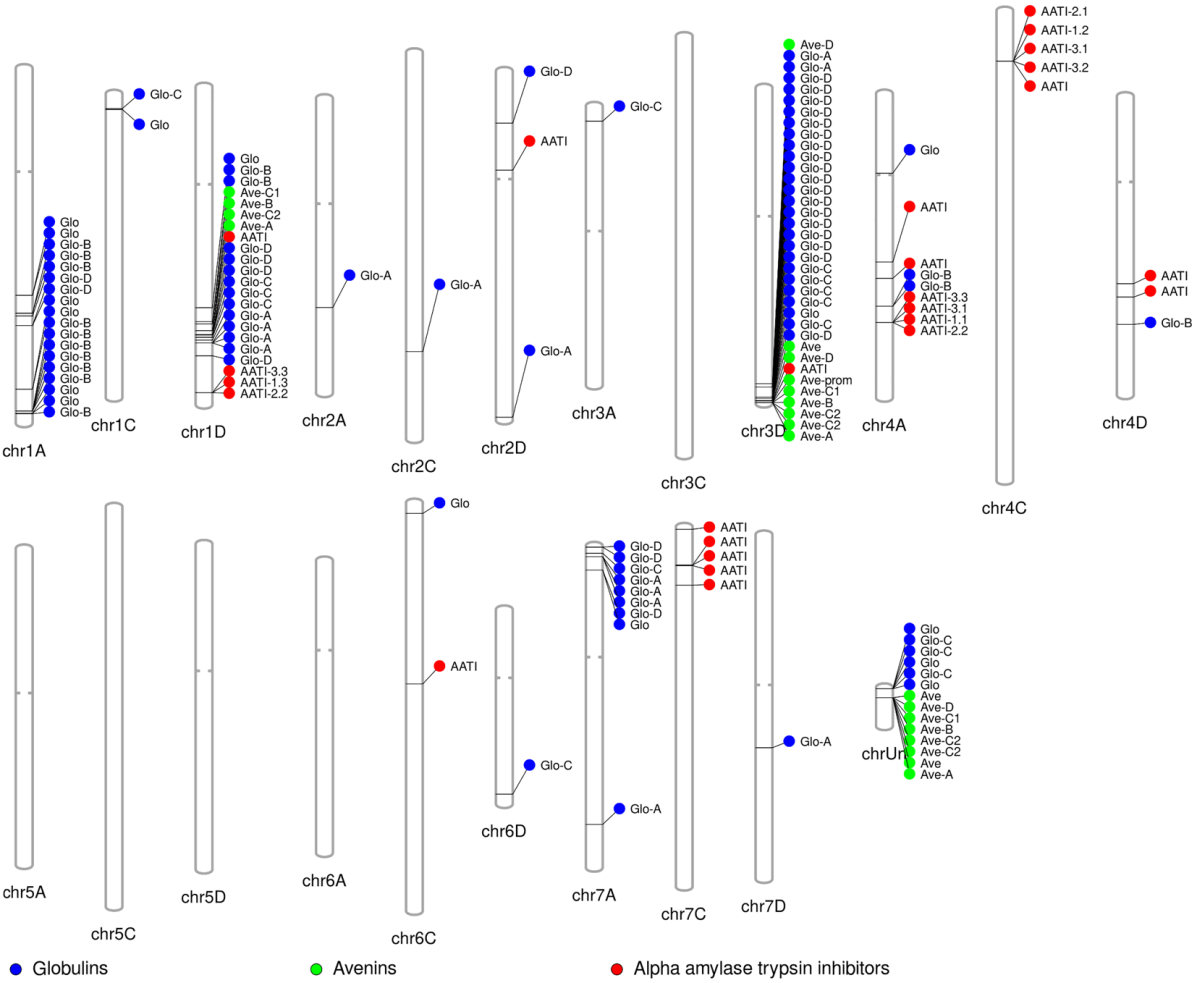
Avenin, globulin and AATI loci in *Avena sativa* chromosomes (AACCDD, 2n = 6x = 42) The figure was drawn using PhenoGram software (http://visualization.ritchielab.psu.edu; ^38^).

Sequences of all samples were mapped against the developed reference (Fig. 1) and consensus sequences were extracted through variant calling process. The presence of sequence of oat samples in each of the 213 loci was checked in Integrative Genomic Viewer and re-annotated (Supplementary Table S1). Out of the 213 loci, 21 genes for avenins, 87 genes for globulins, and 23 genes for α-amylase trypsin inhibitor were found (Supplementary Table S1).

### Avenins

**Figure 2:**
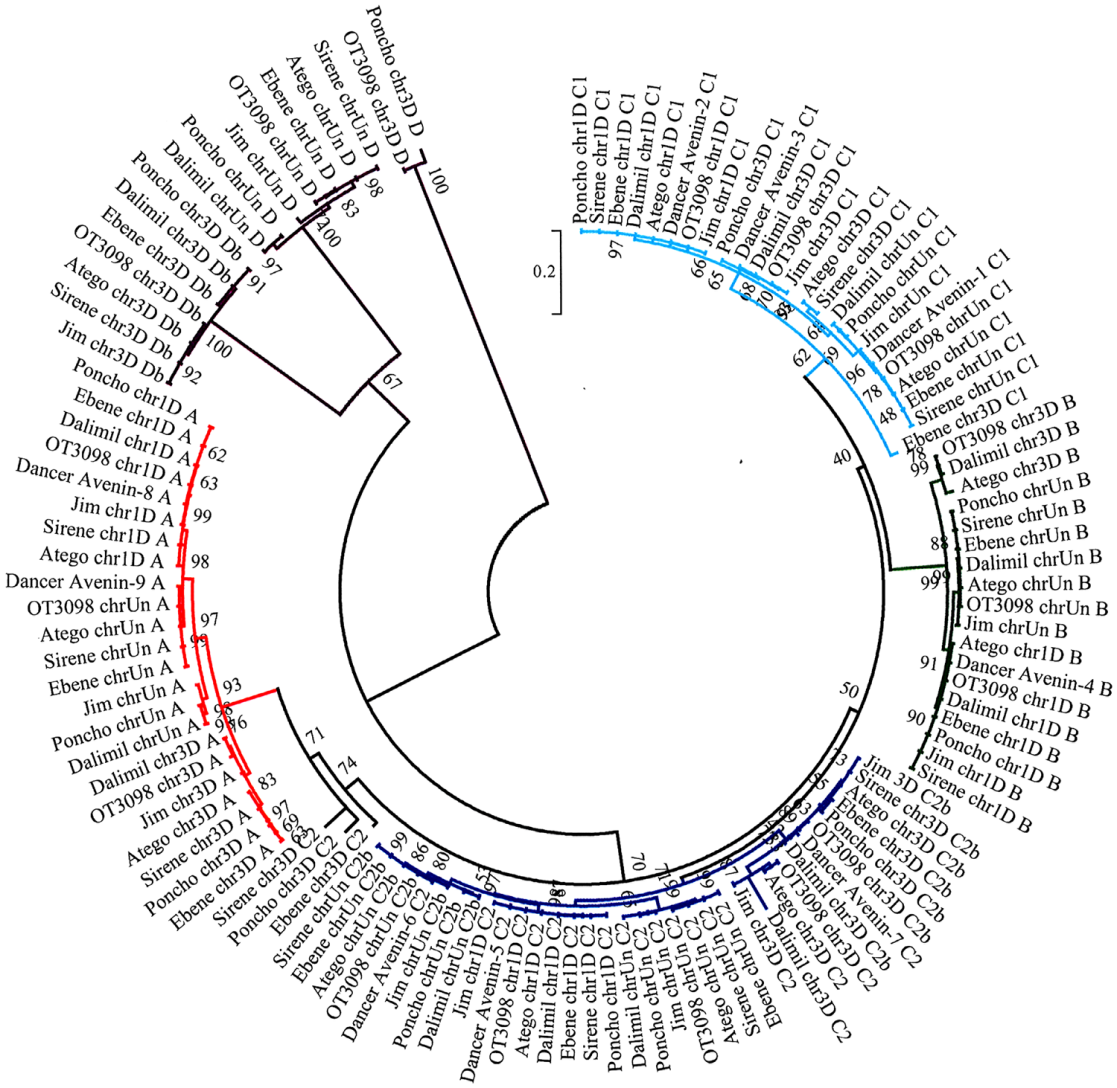
Molecular Phylogenetic analysis of avenins by Maximum Likelihood method based on the Jukes-Cantor model. The tree with the highest log likelihood (− 19412.94) is shown. The analysis involved 119 nucleotide sequences; there were a total of 1043 positions in the final dataset. Colours of branches correspond to the group of avenins: red—avenin (A); green—avenin (B); light blue—avenin (C1); dark blue—avenin (C2); violet—avenin (D).

From the total of 21 contigs identified in this study as avenins, one contig contained a sequence of avenin promoter and three contigs contained only fragments of avenin genes or pseudogenes with internal stop codons. These sequences were thus omitted from the subsequent analyses. Seventeen contigs of OT3098 were aligned together with sequences available in public databases. Five clusters were identified (Fig. 1; Supplementary Figure S1). These contigs of all sample sequences were then aligned and the resulting phylogenetic tree is presented in Fig. 2 showing six groups. Two most genetically distant groups are labelled D. The other groups involve avenins of the type A, B, C1, and C2. The dendrogram also shows that there is a low level of sequence diversity between oat varieties within genes. All varieties had the same gene compositions with the exception of **'**Ebene**'**, **'**Jim**'**, **'**Poncho**'**, and **'**Sirene**'** that did not contain avenin gene B on the chromosome 3D (chr3D-479782203). Further, only **'**Poncho**'** contained four additional gene fragments (chr3D-450843917; chr3D-479670106; chrUn-5633586; chrUn-15655427) (Fig. 1; Supplementary Table S1).

Four avenin genes were mapped on the chromosome 1D and the genes were localized in the order: C1-B-C2-A. Nine genes were situated on the chromosome 3D in the order D-fragment-D in higher distances and then as a cassette promoter-C1-B-C2-C2-A. The remaining eight genes stayed unmapped even when the reference sequence OT3098 v2 was used and were situated on the chromosome Un as a cassette in the order: fragment-D-C1-B-C2-C2-fragment-A (Fig. 1). The length of the avenin proteins ranged from 160 to 265 aminoacids with an average of 223 aminoacids including 19 aminoacids of a signal protein. The most abundant aminoacids in all avenins were proline (8.2–11.0%) and glutamine (24.1–34.9%). In C1 avenins, leucine was the most abundant (15.3–17.4%) (Table 2). The structure of avenin genes consisted of seven domains as was proposed by Anderson (2014): signal peptide, three conserved domains, two variable domains with high representation of proline, glutamine and leucine residues in irregular repetitions, and C-terminal domain. All avenin genes contained eight cysteine residues with the exception of A group avenins that had a ninth cysteine in their C-terminal domains.

**Table 2:** The frequency percentage of aminoacids calculated from the detected and annotated avenin sequences.

	A			B			C1			C2					D*	
	1D	3D	Un	1D	3D	Un	1D	3D	UN	1D	3D-a	3D-b	Un-a	Un-b	3D	Un
Alanin	8.06	8.72	7.18	6.82	6.28	6.70	7.11	6.82	7.44	6.67	8.76	8.26	7.49	6.90	8.00	8.13
Cystein	4.27	4.62	4.31	3.64	3.59	3.57	3.16	3.03	3.31	3.14	3.69	3.48	3.52	3.07	8.57	6.25
Aspartic acid	0.47	0.51	0.48	0.91	0.90	0.89	0.79	0.76	0.41	0.78	0.46	0.87	0.88	0.77	0.57	0.63
Glutamic acid	1.42	1.54	1.44	2.27	2.24	2.23	1.98	2.27	2.07	2.35	2.76	2.61	2.20	2.30	1.71	1.25
Phenylalanin	4.27	3.08	4.31	6.36	6.28	5.80	3.56	3.41	4.13	7.84	7.37	7.83	6.61	7.66	4.57	3.75
Glycin	3.32	4.10	3.35	3.18	2.69	3.13	0.40	0.76	0.83	1.18	1.38	1.30	1.76	0.77	4.57	5.00
Histidin	1.42	1.54	1.44	0.91	0.90	0.45	0.79	0.76	0.83	0.78	1.84	0.87	0.44	0.77	1.71	1.88
Isoleucin	4.74	5.13	3.83	5.00	5.38	5.36	3.16	3.79	3.72	3.53	4.15	3.48	4.41	3.83	4.00	5.63
Lysin	1.42	1.03	1.44	0.45	0.45	0.45	1.19	1.14	1.24	0.78	0.92	0.87	0.88	0.77	1.14	1.25
Leucin	6.16	8.21	6.22	9.55	9.42	10.27	17.39	16.67	15.29	8.24	9.22	8.70	9.25	7.66	7.43	10.00
Methionin	9.48	9.74	9.57	5.91	5.83	4.91	2.77	2.65	2.48	1.96	2.30	2.17	2.64	1.92	6.29	6.25
Asparagin	0.95	1.03	0.96	0.45	0.45	0.45	0.79	0.76	0.83	0.78	0.92	0.87	0.88	0.77	1.14	1.25
Prolin	9.48	8.21	9.57	9.09	8.52	8.93	8.70	8.33	9.09	10.98	9.22	9.57	10.13	10.34	7.43	8.13
Glutamin	27.01	24.10	26.32	29.09	29.60	30.80	34.39	34.85	32.64	32.55	30.88	30.87	30.40	33.72	16.57	16.25
Arginin	2.84	3.59	2.87	2.73	3.14	2.68	1.58	1.89	2.07	2.35	2.76	2.61	2.64	2.30	3.43	3.75
Serin	2.37	3.08	2.87	2.73	3.59	2.68	3.16	2.65	2.89	1.57	1.84	1.74	2.20	1.92	5.71	3.75
Threonin	3.32	3.08	3.35	3.18	4.04	3.13	1.98	1.89	1.65	3.53	2.30	3.04	3.52	3.45	9.14	7.50
Valin	6.64	6.15	8.13	6.36	5.38	6.25	5.93	6.44	7.44	9.41	7.83	9.13	8.37	9.58	6.86	6.88
Tryptofan	0.00	0.00	0.00	0.00	0.00	0.00	0.00	0.00	0.00	0.00	0.00	0.00	0.00	0.00	0.00	0.63
Tyrosin	2.37	2.56	2.39	1.36	1.35	1.34	1.19	1.14	1.65	1.57	1.38	1.74	1.76	1.53	1.14	1.88
Total number of aminoacids	211	195	209	220	223	224	253	264	242	255	217	230	227	261	175	160

*The frequency was calculated only from those genes that do not contain excess stop codons within the gene.

Four celiac disease T-cell HLA-DQ2.5 immunoreactive epitopes^28^ were identified in avenin sequences. The epitope HLA2.5-ave-1a (PYPEQQEPF) was found in avenins of the C2 group, only once per protein, therefore in five molecules per oat haploid genome. The epitope HLA2.5-ave-1b (PYPEQEQPF) was predominant in C1 avenins as PYPEQQQPF once per protein, occurring in three molecules per oat haploid genome. Epitope HLA2.5-ave-1c (PYPEQEQPI) was identified in B avenins as PYPEQQQPI in 'Atego' and 'Dalimil' in three molecules per oat haplotype and in the remaining varieties only in two molecules per oat haplotype. The epitope HLA DQ2.5-ave-2 (PYPEQQPF) was specific to A avenins and occurred in three molecules per oat haplotype. In addition, a 9-mer PFVQQQQPF sequence, formerly known as Av-γ9B epitope, was located downstream of the HLA DQ2.5 epitopes in the first repetitive region only in the C2 avenins, usually in two or three repetitions per each C2 avenin gene. So, 10–15 9-mer PFVQQQQPF sequences occurred per haploid oat genome.

### Globulins

Within all mapped sequences, 87 globulin genes were identified. After removing 12 sequences shorter than 250 bp, 75 contigs were aligned in the MEGA software. When removing before start and after stop codons, 67 contigs represented whole globulin genes. Out of the 75 genes, 64 genes were found in 'Ebene', 65 in 'Atego', 66 in 'Dalimil' and 'Poncho', 67 in 'Sirene', and 68 in 'Jim' (Supplementary Table S1).

**Figure 3:**
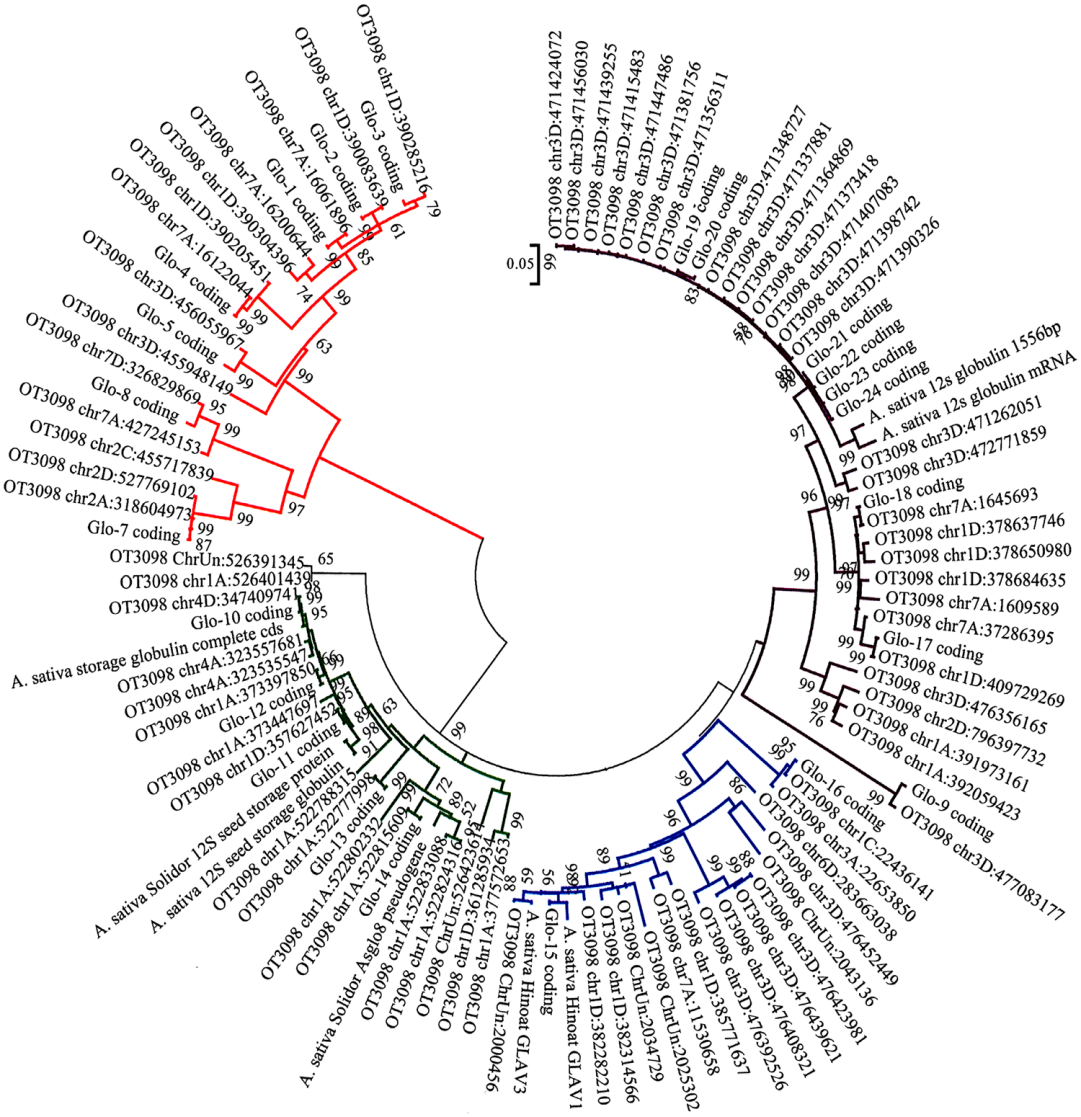
Molecular phylogenetic analysis of globulins by Maximum Likelihood method based on the Jukes-Cantor model The tree with the highest log likelihood (− 29,915,86) is shown. The analysis involved 106 nucleotide sequences; there were a total of 2043 positions in the final dataset. Colours of branches correspond to the group of globulins: red—globulin group A; green—globulin group B; blue—globulin group C; violet—globulin group D.

The globulin sequences of the reference OT3098 were aligned with globulin genes published by Anderson^29^ and phylogenetic tree was constructed (Fig. 3). Four main groups can be seen in the dendrogram. The first group (A) involves globulin genes of the types Glo-1–Glo-8, the second group (B) containes globulin genes Glo-10–Glo-14, the third cluster (C) is formes of the globulin genes Glo-15 and Glo-16, and the fourth cluster (D) involves globulin genes Glo-9 and Glo-17–Glo-24. Similarly to avenins, there were a low level of sequence diversity between oat varieties within genes (Supplementary Figure S2).

The majority of globulin sequences were mapped on the chromosome 3D (26), followed by the chromosomes 1A (16), 1D (14), and 7A (9); eight genes have not been mapped (chrUn) (Fig. 1; Supplementary File S1). Globulin genes contained nine domains: signal domain, four exons, three introns, and C-terminal domain. Besides the introns, the most variable part was the exon 3 between intron 2 and 3. There was a relatively high frequency of glutamin. The second most variable sequence of oat globulins was the C-terminal domain. It varied in sequence and in the length as well.

### Α-amylase/trypsin inhibitor

In total, 25 α-amylase/trypsin inhibitor (AATI) genes were identified within the six oat varieties, 14 as whole genes and 11 as gene fragments or pseudogenes. Most of them were localized on the chromosome 4(10 genes and 3 fragments): five genes and one fragment on the chromosome 4A, four genes and one fragment the chromosome 4C, and one gene and one fragment on the chromosome 4D. Further, one gene and four fragments were mapped on the chromosome 7C, and three genes and one fragment on the chromosome 1D (Fig. 1).

**Figure 4:**
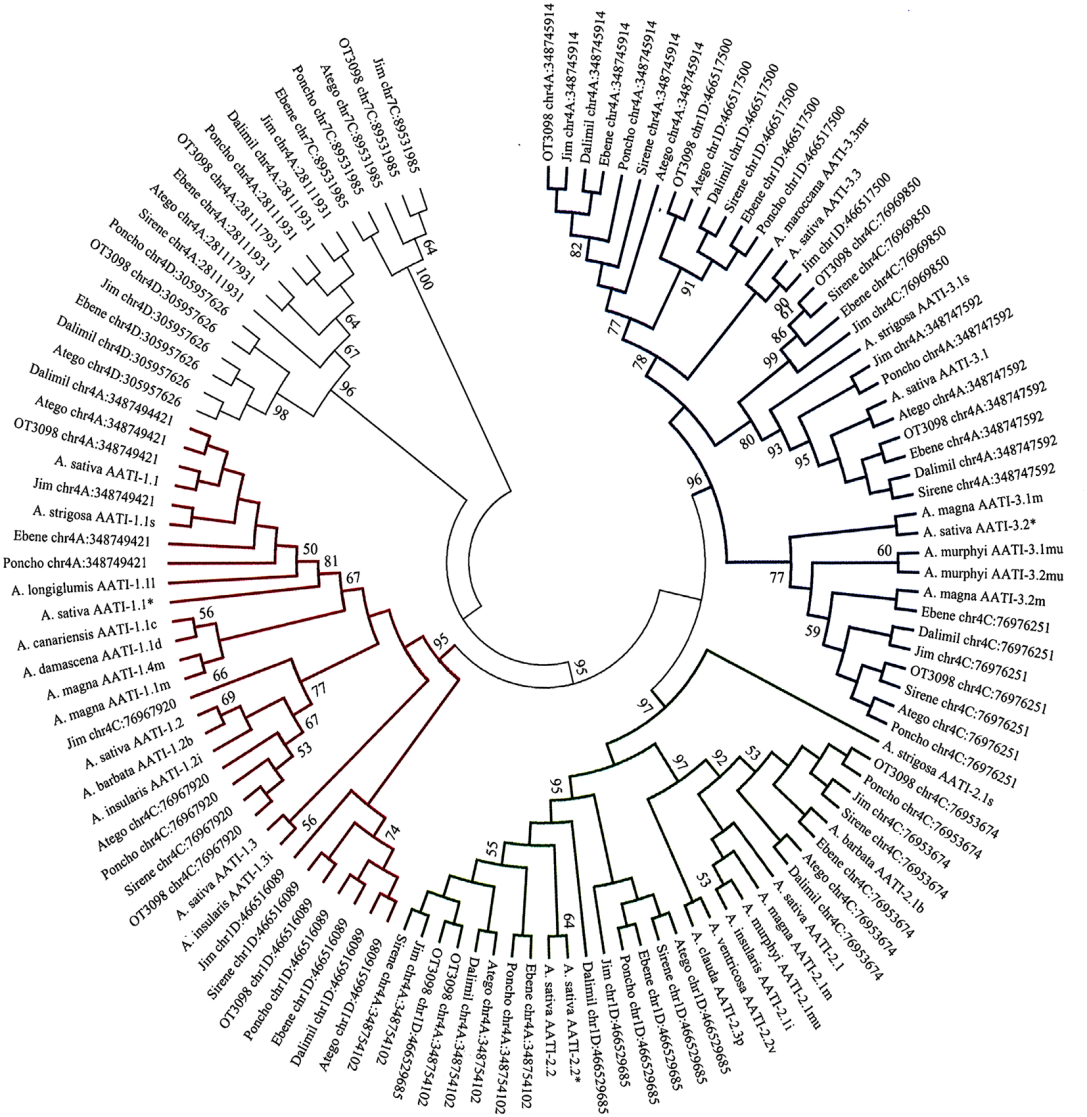
Molecular Phylogenetic analysis of AATIs by Maximum Likelihood method based on the Jukes-Cantor model Bootstrap consensus tree inferred from 1000 replicates. Branches corresponding to partitions reproduced in less than 50% of bootstrap replicates are collapsed. The analysis involved 121 nucleotide sequences; there were a total of 723 positions in the final dataset. Colours correspond to the group of α-amylase trypsin inhibitor: red—AATI 1; green—AATI 2; blue—AATI 3; black—unknown group of AATI.

The AATI sequences of the six oat varieties and of the reference OT3098 were aligned together with AATI genes published by Gazza et al.^19^ and phylogenetic tree was constructed (Fig. 4). Five main groups can be seen in the dendrogram. The first group (A) involves AATI-1.* genes, the second group (B) contained AATI-2.*, the third cluster (C) is formed of AATI-3.* genes, and the last two clusters involve non-classified AATI genes (Fig. 4, Supplementary Table S1). Similarly to avenins, there were a low level of sequence diversity between oat varieties within the genes.. The length of AATI genes ranged from 432 bp (AATI 3.2 on the chromosome 4C) to 696 bp (AATI-? on the chromosome 7C). None of the analysed oat varieties contained all AATI genes. In 'Dalimil' and 'Sirene', 'Poncho', 'Atego' and 'Ebene', and 'Jim', 17, 18, 19, and 21 genes were found, respectively.

## Discussion

The prerequisite for this study was to find as much avenin, globulin, and α-amylase/trypsin inhibitor genes as possible in the selected oat varieties. On the other hand, the sequencing of the whole oat genome or transcriptome would be too expensive. That is why a strategy to sequence enriched transcriptome libraries of oat seeds collected in the peak of aimed gene expression according to Real et al.^10^ was selected. The enrichment consisted in the amplification of the targeted genes selected using degenerate probes specific to avenins, globulins, and AATIs. Results showed that some DNA fragments and non-targeted genes were also selected and sequenced. From the 56,394 sequences, only 14,031 (24.9%) were annotated as the targeted genes. This is probably due to the use of degenerate probes that increase the likelihood of non-specific hybridization.

### Avenins

In this study, 14 avenin genes, six pseudogenes or fragments with internal stop codons, and one avenin promoter were identified. Cluster analysis showed five groups named according to Real et al.^10^: avenin-A, B, C1, C2, and an additional group named D. Avenin-D genes differ from the other four groups in aminoacid composition, and therefore they could be regarded as avenin pseudogenes. To the best of our knowledge, it is the highest number of avenin genes that has been found. Londono et al.^15^, identified ten genes in 'Gigant'. Anderson^29^ assembled avenin ESTs and got nine genes in 'Dancer'.

Avenin sequences were mapped into the reference PepsiCo OT3098. All genes were localized in a kind of cassette in three regions on the chromosome 1D, 3D, and an unknown chromosome, as even version 2 of the reference did not allow to assign all loci to chromosomes. Each cassette of avenin genes contained one gene copy of avenin-A, B and C1, and one or two copies of avenin-C2. Except of the cassette on the chromosome 1D, the others were accompanied by pseudogenes and avenin gene fragments. Therefore, it is likely that each oat variety contained at least 12–14 or even more genes through additional gene duplication^15^. In Tanner et al.^30^, a higher number of avenins in oat genome was indicated by the level of protein via combination of several methods of protein extraction^30^. It is interesting that five groups of avenin proteins were identified that could be assigned in compliance with the epitopes to avenin groups A, B, C1, C2, and D^30^.

Only marginal sequence differences were found between the oat varieties within the individual genes, therefore it is unlikely that the differences in avenin genes on DNA level could be used for oat variety identification. There is a question of whether it is even possible at least on the protein level^10^. This poor diversity can be explained by a more recent evolutionary history of oat compared to other cereals^29^.

The structure of avenin proteins A, B, C1, and C2 corresponded to already published results^15,29^, as well as the content and the positions of cysteine residues. Avenin sequences of the group B and C presented eight cysteine residues, whereas avenins of group A showed nine. It is likely that it formed intermolecular disulphide bonds and formed a polymer similar to wheat glutenins^10,31^. Aminoacid composition differed from the work done by Real et al.^10^ only in the content of alanin in avenins A (7.98% on average in this work and 4.87% in Real et al.^10^) and B (6.60% on average in this work and 4.14% in Real et al.^10^), of leucine in avenins B (11.55% on average in this work and 7.53% in Real et al.^10^), and in the content of glutamine in avenins A (25.81% on average in this work and 22.24% in Real et al.^10^) and C (32.54% on average in this work and 29.25% in Real et al.^10^). On the contrary, the average value of proline and glutamine content was lower than in 'Dancer'^29^. When comparing to wheat gliadin and glutenin, lower proline and glutamine content of avenins was found which could lead to the lower celiac toxicity with respect to wheat prolamins.

The avenin-specific T-cell epitopes DQ2.5-ave-1a, DQ2.5-ave-1b, DQ2.5-ave-1c, and DQ2.5-ave-2^5,28^ occurred only once per protein, therefore there was 12–14 epitope residues per *Avena sativa* haplotype. When compared to the composition of wheat, barley, and rye epitopes (Supplementary Figure S3), avenin epitopes grouped together with glutenin epitopes DQ2.5-glut-L1 and DQ2.5-glut-L2, gliadin epitopes DQ2.5-glia-1a, DQ2.5-glia-1b, and DQ2.5-glia-1(2), hordein epitope DQ2.5-hor1, and secalin epitope DQ2.5-sec1 but with a low level of bootstraps. Avenin epitope similarity to gliadin, glutenin, hordein, and secalin epitopes expressed per each aminoacid incidence was: **P**(0.53)–Y(0.03)–**P**(0.67)–E(0.47)–**Q**(0.60)–Q/E(0.37)–Q/-/E(0.30)–**P**(0.77)–F/I(0.13).

Moreover, Ellis et al.^32^ replaced successively all aminoacids of the epitope DQ2-α-II by alanine residues and studied these epitopes' reactivity. They found that the replacement of any position within the 9-mer led to a significant decrease of the reactivity. Therefore, the precise epitope sequence is important. In oat, there are only four aminoacids (marked above in bold) that approximate avenin epitopes to immunoreactive epitopes of wheat, barley, and rye. Moreover, unlike wheat, barley, and rye where prolamins are the main storage protein constituting 60–80% of the grain total protein content, oat prolamins (avenins) account for around 10%^33^. Hardy et al.^34^ found the low rates of T-cell activation after an oat consumption of 100 g per day. They suggested that doses of oats commonly consumed were insufficient to cause clinical relapse and supported the safety of oats demonstrated in long-term feeding studies^34^. So, it is possible to hypothesise that oat could be safe for most people with celiac disease, but there is a cohort of them who are likely be sensitive to any doze of oat gluten. For them, the strict gluten-free diet will be the only option.

### Globulins

In total, 75 globulin genes were identified and mapped. In spite of the different number of genes found in each of the six varieties, it was around two times more than was found in 'Dancer'^29^. The ratio between the number of prolamin and avenin genes corresponded to the proportion of prolamin (80%) and avenin (10–15%) protein content^35^. Cluster analysis confirmed the existence of two main and two other groups (Fig. 3;^29^). Location into clusters did not correspond to the position of the gene on a chromosome in the oat genome but rather to the type of globulin genes Glo-1–Glo-24^29^. Several genes showed differences from these already published globulins. Non-substantial sequence diversity was found between oat varieties that differed mainly in the presence or absence of individual genes; however, it can be a false negative result despite the prepared oat storage protein enriched libraries.

The structure of globulin genes was in accordance with those identified in 'Dancer'^29^. The only difference was that three introns were identified in the genes when mapping sequences after transcription to the genomic oat reference OT3098. The first intron is 116 to 128 bp in length, the second is the most variable in length (112–137 bp), and the third is the shortest (104 bp). Although some of their regions are relatively rich in proline and glutamine, no epitope-like sequences have been identified.

Oat protein consisted mainly by globulins plays a significant positive role in controlling blood glucose response by slowing the gastric emptying rate, promoting the secretion of insulin, and affecting the digestibility of starch^36^. High protein content (12–17%) and the good ratio between globulins and avenins enable oat to be a good nutritional ingredient for both animals and humans, favourable even for individuals with celiac disease.

### α-amylase/trypsin inhibitors

The third potentially harmful protein family are the AATIs belonging together with lipid transfer and seed storage proteins to the AATI-LTSS protein superfamily which is unique to higher plants. Proteins in this family are known to play important roles in defending plants from insects and pathogens, lipid transport between intracellular membranes, and nutrient storage. They have also been identified as allergens in humans^37^.

From 25 AATI genes, 14 were sequenced from start to stop codons. They belonged to three families named AATI-1, AATI-2, and AATI-3 with contrasting primary structures, molecular weights, and isoelectric points^19^. Unlike 'Donata' where four genes per each AATI gene family were identified^19^, two genes of AATI-1, three genes of AATI-2, and five genes of AATI-3 were found in this study.

Oats synthetize AATI proteins as precursors that undergo specific cleavage of a leader peptide of 25–28 aminoacid residues and then accumulate on the starch granules, together with more abundant amounts of vromindoline proteins. In this study, genes for vromindoline and puroindoline proteins were also blasted in mapped contigs which suggested a homology among all studied proteins. However, detailed analysis goes beyond the aim of this study. Tanner et al.^30^ also found that in 18 peaks of HPLC analysis of extracted oat grain proteins, there were avenins and gliadin-like avenins accompanied by a large number of AATIs, vromindolines, enzymes, and metabolic proteins. The number of AATIs detected in 50% ethanol purified protein was in the same range as the avenins^30^.

Oat AATIs showed a high level of homology compared with α-amylase/trypsin inhibitors from wheat and barley which have been associated with human disease including food allergies and baker's asthma. Similarly to avenins and globulins, higher variability was found between individual genes than between six oat varieties and oat reference OT3098.

## Conclusion

To the best of our knowledge, this is the first study in which such a high number of avenin, globulin, and α-amylase/trypsin inhibitor genes were identified and also mapped to the hexaploid oat chromosomes via a unique procedure of enriched library preparation and bioinformatics. The number of genes corresponded to the ratio of grain storage protein content. Oat prolamins (avenins) formed only 10% of storage proteins and moreover, there was a lower proline content compared to wheat prolamins. Therefore, whether oat is less toxic to people with celiac disease is a question of dose and it depends rather on patient susceptibility. It is likely that oat could be safe for most people suffering with celiac disease. For those who are sensitive to any doze of oat gluten, the strict gluten-free diet will be the only option. Low level of variability was identified in the sequences of avenin, globulin, and AATI genes between the six oat varieties with different immunoreactivity declared at the beginning of the study. Therefore, we can conclude that on the level of gene sequences none of the six oat varieties can be more suitable for patients with CD than others. The reason for the variability found on the protein level should be further investigated on the level of posttranscriptional and posttranslational modifications.

## Supplementary Information

Supplementary Information 1.

Supplementary Information 2.

Supplementary Information 3.

Supplementary Information 4.

Supplementary Information 5.

Supplementary Information 6.

## Data Availability

DNA sequencing data were deposited in NCBI Sequence Read Archive—PRJNA774959. Analysed data are included in this published article, especially in its supplementary files. Material—oat seeds collected within this study are available from the corresponding author on reasonable request.
