# Supplementary material for: Analysis of oat seed transcriptome with regards to proteins involved in celiac disease
Source: Sci Rep. 2022 May 23;12:8660. doi: 10.1038/s41598-022-12711-6 (PMC9127096; doi:10.1038/s41598-022-12711-6)

Supplementary Figure S2 - Molecular Phylogenetic analysis of globulin sequences of the reference OT3098 and all six oat varieties by Maximum Likelihood method

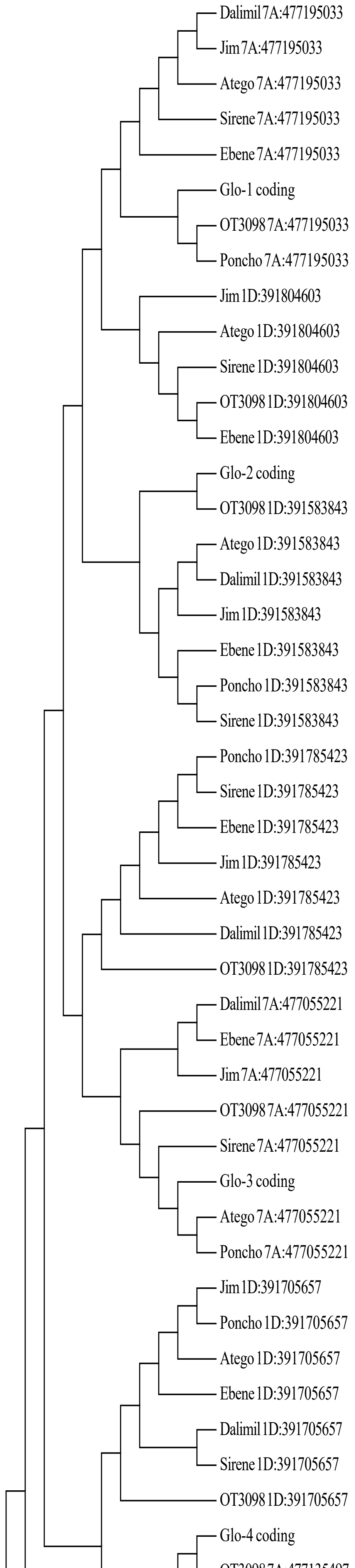

Supplement: Supplementary file 2 — Supplementary Information 2. [file 41598_2022_12711_MOESM2_ESM.pdf]
