# Supplementary material for: Analysis of oat seed transcriptome with regards to proteins involved in celiac disease
Source: Sci Rep. 2022 May 23;12:8660. doi: 10.1038/s41598-022-12711-6 (PMC9127096; doi:10.1038/s41598-022-12711-6)

Supplementary Figure S3 - Molecular Phylogenetic analysis of avenin, glutenin, gliadin, hordein and secalin epitopes by Maximum Likelihood method with bootstrap value 1000

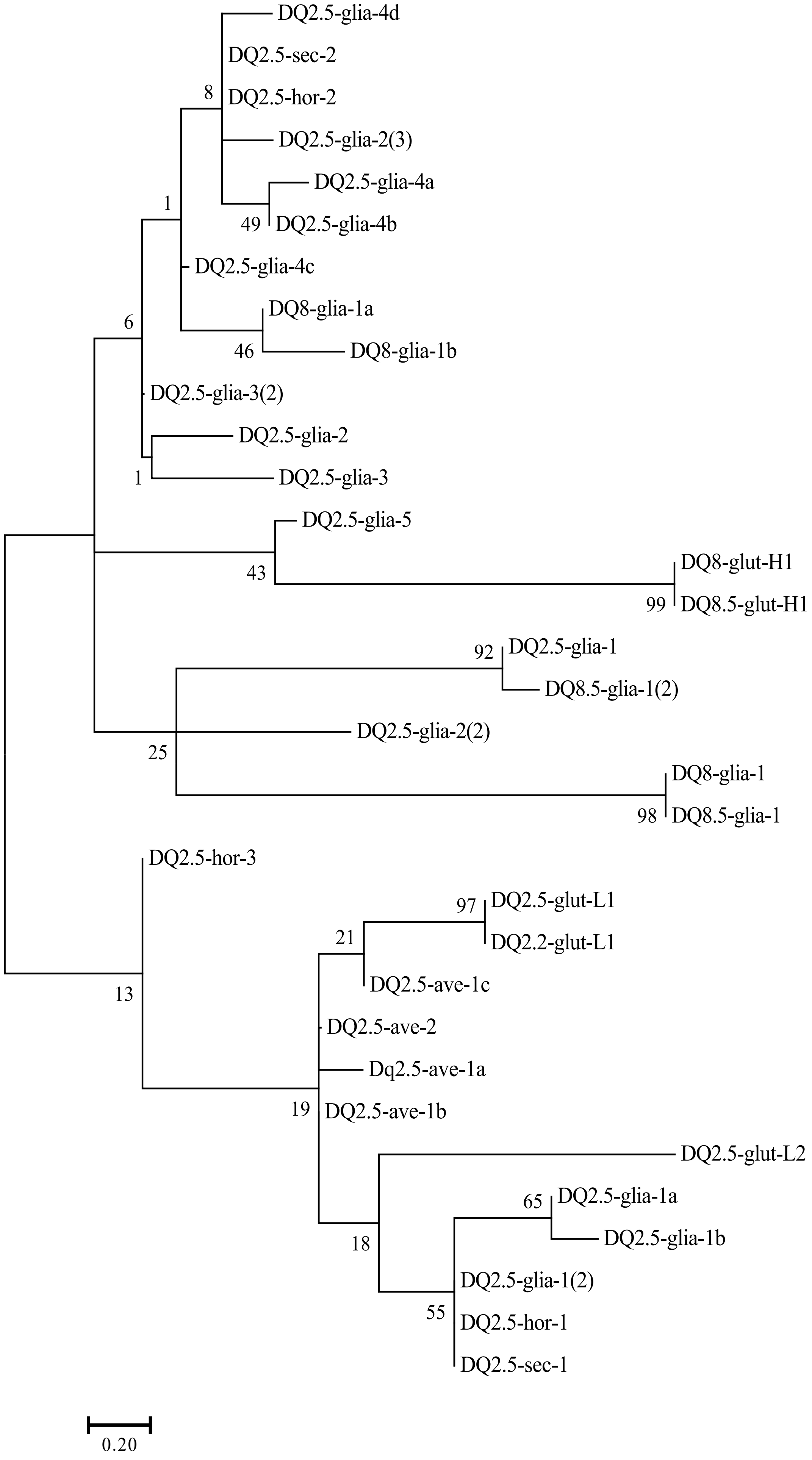

Supplement: Supplementary file 3 — Supplementary Information 3. [file 41598_2022_12711_MOESM3_ESM.pdf]
