# Supplementary material for: Analysis of oat seed transcriptome with regards to proteins involved in celiac disease
Source: Sci Rep. 2022 May 23;12:8660. doi: 10.1038/s41598-022-12711-6 (PMC9127096; doi:10.1038/s41598-022-12711-6)
Supplement: Supplementary file 4 — Supplementary Information 4. [file 41598_2022_12711_MOESM4_ESM.pdf]

Supplementary File S1 - Reference sequence in fasta format extracted from oat reference sequence PepsiCo OT3098 using target loci bed file

>chr1A:329122314-329124314

TGACCCAACAAATATAGCTGCTGCTTGGTATAAATTTTGACGCCTCATACATATAGCTGAGCAAAATGGCTATC
AGTCTGTTACGCCAGCGCACGTGGCAATACCAAGTTACAAATAGAGCTAATCCACTTTTCTCAGTGTTCATGAG
CTTGAGCTGCCAAATCTCAAATTATTCAATGATGGAATCATCCTTTGATCCATGTCAAAACTATTAGCAACAAAA
CTGGCTACCGCGATTTTGACTTCAGACAGTCATAATTTCACTTGTCAGCAACCTCAATGAGCGTCGTCTCGAAAT
CATCAGGAGCCAGGAAACCGTGGAGGTTCATCACGGTGGCTGCAACATTGGCGAGTCCAGGGGTGTTGATAT
CGGTCCTGAATTTCACTCCTGGGTGGAGACCAGGGCCTCCGATCGCAACAGGTACCTGCAAGGTTACGTGCGA
ATTTAAAACCAGACCATAAACACGAACACTCTGGTTGGTTTCAAAGGAAGAATATCAAAAGCACAGCATATGG
CATTGCACTTACTGGCTGAAGCGTGTGCGAGGTAAGAATCTGGATGCTGCCACTCTTGTCAAGAGCCGGCTGG
CCAGATTTGTTTCTTTTCACCATATCCTCTGCGTTTCCATGATCAGCAGTGACAAGGTAAATACCACCAACTTTCT
CCACCGCATCCAAAACAATCTGTTCCAAAGGGAAACAGATGATCGAATAAAGCACCAAACATATAAACAAACT
GTGTAAGCAATTAGGTGCCTAGGAAAATGGCACTTTCGAGAAATTTCCATTCAAACTACAGTAACTAGTGATAT
TTCCACTCAAAATATAACTGTTCAGGACTTCCAGAGAATTTCTAATAAAACTACAGCAACACGAGTCATTTTAGA
TGAATGTAATACTCCCTCCGTTTCAAAAATATAAGATGTTTTAGCTTTGTGAAAAAATGTATCTAGACTTCTTTT
AGTGTGTAATATAAAGATAACAAAAAGAAAACATTGAAAGGGCATAACATAAGAAGTTCCCAAATATACATAT
ACAGATATACGGAAACAAAAGTTGACGACATAAGGTTGTTTCTCTTGTTTATTCTCACCTTGACGGCTTCATCA
GCAGCCTTGCAAGCAACAACTGTGGCTTCAATATCACCGGTGTGACCCACCATGTCACCATTTGGCAGGTTAAT
ACGTACCTACAGAAATATTTTTTATAAGTAATTCTATGGAGGCAAAAAAGACAAGTAATCTACTGTAATTTCCA
GAATACTTTACCTGGTCAAACTTTCCACTGAGGATAGCATCCCGGGTTTTCTCAGCAATTTCAAGTGCCTTCATT
TTGGGTTGCTCATTGAATGTGATACCGCTATCACTAGGAATTTCAATATACTCTTCCTTGGTTTCATCAAAGTAT
CCAGAACGGTTTCCATTCCAGAAAAATGTGACATGACCAAACTTCACTGTCTCACTGTTATGAACAAATCGATG
AGTCCGTAAGTATAAACACAAATATGAAGAAAAATATCTAACCCAACACGAACTGAAACATGTATAATACTGTT
AAACTTTAGCCAGAAAAAGAGCTAGGAGTAGATAATTTCAAGAACCCCGTATACCGATTCTGCCACTACAAAA
GGATATTTGTCTAAAGGAATGTCGAATCAAATGGGTGCCTAAAAGTACCACAGAAACTTAAAATTTCCCTTTAC
TAGTTTGGTGATGACATTATATTGTGATTAAAATGAAGGACACAACAACAAATATATAAGTAGCAGTAAAGCTT
TTGCACAGGTATAAAGCATATGACTAGTGTTCCATATTTTACCTAGATATTAGGGGCCTATAGTTGTTCCAACTG
GAAGATGGGAAAACTTAACACAAGGTCAGTTGAAGCAATGGTTACTTCACCTCCGAGTAATGGCCAGCACTTA
TACAATTTATTTAGTATATACACATGAATGTGAAGAACCAGGTGAAATAGAATAGGAGTATGAAAATTAGGTA
CTT

>chr1A:345839000-345841000

TTGGAGAACCATTTTGTTTGTTCATTATTCTTGGATTCTTATTATAGTTGCATCCAACACTGTGACCACACGTCAC
GATTTATTTTCTAGCTATGGGGTGCATGCATGCATGCCCAAGCAACATGCTGAGTTTAGGAGAGTTCTAGCCTA
GTACCGACTGGTGGAGAAGGCGCTGCCCTCCAGACGGCACATCACCGGCAGCTGCGCTGCGACCTGCCGCGC
CCTAGCCACCACCTGTAGTTGCATCCTTAGACGGGCTGCCTGCTGCTGGGTGCCAAAACGGGATGGAGACGAA
ACCCCGCCGAACCCCGTCTGACTTTGCCTGCCGCAGTACTGGCCTTGTCCCTCCTGCTGCTGCATGGTCTCACCG
AGCGCATGCTGGGCTGGCTGCCGGTAGATGATGATGCGCAAGCGGTAGGGTTCCTGTCCTTGCTGTTGCTTCT
GCGGAAAAGTTTGGCTGGGGCGGTACGACCCTTGCTCTTGGTGTTGGGGACGATAAGTTTGGCTAGGAGGAC
CATACATCCCTTGCTCTAGCTGCTGCTGCTGAGAAGTTTGGCTAGGACCGTACATCCCTTGCTCTTGCTGCTGCT
GAGAAGTTTGGCTGGGATGGTACCACCCTTGCTCTGGCTGCTGCTGAGAAGTCTGGCTGGGACGGTACCACCC
TTGCTCTTGCTGCTGCTGAGAAGTCTGGCTGGGATGGTACCGCCCTTGCTCATGCTGCTGCTGAGAAGTTTGTC
TGGGAATGTACCGCCCCTGTTCTTGCTGCTGCTGAGAAGTTTGTCTGGGATGGTACCACCCTTGCTCTTGTTGT
GGATAAGTTTCACCGGGGTAGTATGCGCCTCCTTGCTCCTCCTCGTCCTCACTGGGGTCGTAAGGTTCTTCTCCC

AGCGGCGCCACGGCTTGATGCTCGTACTGCCTCACGATCTGGCGGACGGCGGCGGCGCGGCACTCGCGGCTG
ATGTCCTGGAGCTGCTGACAGCATTGGACGCGTGCCCCTGTACTCAACTGCGACGATTGTGGGCGGAGGAAG
GGCAGTTGGACGGCCACTTGGTGGTTGACAACTTGCTGGCACGCGTCGAGCGAGCTCTCCTGGAGCTCACGCT
GGCACTGCAGCTGCAGCCTGGTGTTATCTCCTTCTGAGGCGGAGAGAGCCACGAGGGCCACGAGGACCGTGG
CGAAGAAGGCCAATTTAGCCATTTCTGTGGACTGCTAATGAATTAAACTCTTGTTTCTAGAGCTCGGTGCTCTT
GTTGGTGATGATGAGATTGTGGAGATTGGCTGGGTTCTTATAGGAATGAGGTGAAGGTTCAGGACCTTGATA
GTGTGCACGGCGAGGTTAGTTCACATTAAGAAGAAACTTGAGAAGAACAAAATAAGTTGGATAAGCGGAGGA
GTGCAGTGCTCCTAAGAGTTAAAAGGTTGTGTTTTGGAGTTTGCAGAACGGAATTTTGGACTGCCTTGCCAAT
GCTCTGTTATCGGTTTAAACGTGCAAAAGGACACCTTACGTACACCTAAAAGACCTTGCCAGGCTAGCTCTCAC
GTGAGAATTGATGAGATTGTTCTAGCGTCAGTACAAAAAATGGTTGCAGTTCTTGGATTTTGACAGATGATGT
GTCTTTTAGTCATATGCAAATCATCTTCCCAAAAACTGTTATTTACTTGCGGCTTTCAGGTAGCACAAATCCATG
CTATGACTCAACAACATCAATCCAGCATGACGCACAATAGATATCCATGCTATGACACAAATATCAATCCGTGC
TGTGACGCGGCAAACCAAAAAGGGCGCCGATTTTTCCCTACAGTTTCTACACGCAAAGCAACATCTTCTTTCCG
TGATAAATTCAACTAATTGTGATCTTTTGGACGCGCACGCATATTTGGGTGCGTTTATGTAGGAAAAATACTGC
CTGGCTACCAAAACAATGCAGTATAATACTATTTTTTAAAGATAGTAATCAAACTATTTGAGCCTCATTTACAAA
TCGCAACAAGTCCGG

>chr1A:345845279-345847279

ATTCAACATGAGAAATATTTATGTCTTTATTATCGTCATTTGCGCATGATTCATGAACGAACATGATCACACGCA
TACACCACACTTATTTATCATGGCTAATACCATAGATCACTTCAGCTAGGTAGCTAGTCTAATACTGGCCGCCG
GAGAAGACACTACAATCCATGGGCTCGATCTGGCACACGGTGGGTAGCCCCGCCACGTACTGCCGCACCTGCA
TCAGCCTCACGTGACCGACCTGCTGCATCTGCTGCGCCCCCTCGCCGTAGAACTCCGGTCCTCCCTGACGCCTC
GACGTCTCGCCGTAGTAGATTCCTCCCTGCGGCTGGCTTTCAGCCGTCTCACCGCCGTAGTACTCCTGCTGCCG
CGGTGGCATCTCGTGGCGGAGCACCTGTCCTTGCTGACCACGCCCTTCTTCGCCGTGGTACCCTCCTCCCTGCT
GACGGTGTCCCGCTGTCTGACCACCGCAGTATCCCTGCTCTTGCTGCTCCCCCGGCGAGCCGCGGCGCCCTCCT
CCCAGCGGCGGCATGCTCTCCTCGTAGTTCCTCACCATGCCGCGGATGGCGGAGCAGCGGCACCCGCGGCTGA
CGCTCTGGAGCTGCCGGCAGCAGCGCTCCCTGGTGTCCCACTCCGTGTGGAACAGCGGGACGCTGACGGCAT
CCTCGCTCCCGGCGCCGGTCTGCTTTTGCTGGAGGATCTGCCGGCACGCGTGGAGCGGCTTCTGCTGGACCTC
GCGCCGGCACATCACGTCCCTGTAGCTCTGCTCGGTCAGGTCTTGGGCCTCGGAGGCGGCCATCAGGGCCGCA
AAGAATACCGCGAGGAAGACGAACTTGCCCATATTTCTCTAGCTAGCTCTAGCTTGCTCTTCTCGTTTCTGGTGT
ACTTGAATACCTGGTGGTGGCGAGAGATGTGGAAGAGACGGTCAAATTTTATAGGAACATATATGGTGATGA
TTCGGCACCATGTATATGGTTGTGTGCACGGTGCTCAAGCTCTAGTTGGGTTGAAGAAGTTTTAGTTGAAGTG
CAATGCTTGGAAGAGTGAAAAGGCCGGTACAAATTAAGGGTTTGGGACTTCACGTTAGCCACTTTAATTTGGG
TAAGGTATAGCTTTACATCATCTCTCAGATATATGGCCCAGTTCTTTTGGCGGCTTCCCCAGAAGCTACCCTACC
CCCAGCTTCCTGGGAAAGCCGGTTTAAAAAAATTGCGAGGCTTCCAAATTTGTTAAGACTAGGCCCAGTTCTTT
TGGGGCTTCTCCGAGAAGCCGCCCTCCCCCCAGCTTCCTGGGGAAGCTAGTCTAAAAAATTTGCGAGGCTTCC
AAAATAGTAAAGGCTAAACAAATTTAGAAGCCTCGCAAAATTTTTGAACCGGCTTCCCCAGAAAGCTGGGGGT
AGGACAGCTTCTGGGAGAAGCCACCAAAAGAACTGGGCCTTAACTAGTTTGGAAGCCTCGCAAATTTTTTAGA
CCGGCTTCTCCAGAAAACTGTGGGGAGAGCGGCTTCTCGGAGAAGCCCCCAAAAGAACTGGGCCATAATGGC
AGATGTATTTGCTGTGATGGAAGCATATGATGTGTTTTTTTGGAAGAAAAAAGTATATGATGTTGTGTAGAGT
GTCAAGCTGCAAAAGTATGGATACTTGTATGTATCTCTTGATTTTTTAGATTGAGTATGTACTCCCTCCGAGTCC
GCAAATAAGAGTACTTCTAGCTTTTGCATGAGTCAAACTTTATTAAATTTGACCAACTTTATAAATAAATATAGC
AACATATATTATATCAAATTGACATATTATGAAACTAAATTTGAAAACATATCTAATAGTACCAATTTAGTATCA
TAAATATTGCTACGTTTTTCTAAAAAGTTAGTTAAACTTTACTAGCTTTGACTTTAGACAAAGATAGAAGTACAC
TTATTTGCGGACGGGGGGGGGGGGGGGGGGGGTATGTGACATGCTTGCGTGTGCTGCTATGCACAACAAGT
TTCATACAAGACAT

>chr1A:370872866-370874866

CCAGTACCTAGACTAGACAATAGCGAGAAGTGCACACATTTACATCAATTCTGCAGAACCTTCTAAAGGAACTG
GGAAACGATTATTAGAAGCTAGTACAGAAAGAACGGTGTAGACACTAGACACTCGAACGGCAACTCCATTTCT
GAAGACTCGGTCCGATAACTCCACTCTGGCCGCAGCTTCCATTTCGCTCTCCCTCACCTGCCCCTGCAGTCATGT
AAACAGGAAAAGGATTTCAGCAGCCAAAAGACAGATGCAATCTTCCTTGGTTGTAAACTAAATAAACGAAAAA
AAGTCACTGCAGTTACCACACACTCATTCGCTGTCGTCGCTTACAACCGCTCTTCTGCCCCTCGCCGGCCTCTTC
AGAGGGGGATGAACGGTGCCCGACACAGCTCGCGATGAATCGTCTTCAGAACCGTCAGATCACGGATCATTA
GTTCGCGTAAGAAAGATCTATCCAGAGCTTCTGTTCTGTTACACAACAGCGTACCTGAGCTTGTGGCGTCTGGA
GCTCTCTTTCCCTTGTTGGCATTTGGTTGAATTAACCTGACATTCTGAGCACACGACAGGATTAACCATTTTACA
TTCAGTGACTTTTGACATGCAGGAAAGATGAATACTACACATAAGCTTTGGATTTCTGGTTACCTTCGCATCTTC
ATCTTGCCTAGATCTCTGAACCTCCGGCTGGGCTGCCTTATTGTGTCCAGATCTCTTCTCAGCAACAGTGATCAC
ACCAACCTCCATTTGCTCCTCTGGAATCTCACATCTCCCATTGAAAGGTGGCAAGTGGCAAAACCTGAAATTTC
ACAACGTGAACACTTCAGAACTGTCAAAAAGATATTCAACTTCTGTTGTCTACTTAAAATAAATATACTGATGCT
CCTGCTGGAAGCGCTATACCTCTGGTGATACAGGGCTAGTTCGAAATTAACTTGACTGATCAGTGAATAGATTT
AACGGTGCAAAATATTATTTAGATGAAAATAGGTAACATGAACACATCATCCAGTAAGCTCAAAAATCAGCAA
GATAAACAAGAAGAGATCTCTAGCACAGTAAACTTGTAAGGTAAGCAGTGAGATTTTGCAAGTGGTTTGGAGT
TGAGGATGCTAAAGTATTCCAAAGAGATTTTGGATAAATTTGATTTTTGGTCACAGCAACTTTCACTTTTGGTCT
GTTACCCTCTCTCTCAGGTTGGCGAAAGCATTTGTGTAAAAGGTTAGACCTGATCACGGTAGCCAAGGTGGGT
ACCAATGATATGTCGCAAGCTGAAAACTTTGCACATGAATTAAATGTTCCTAGCTGAAAACTTTAAATTTTGCCA
AGTAAATTGGGGGTCCCTAAACACAACTATTAAAATGATAAAATCATCGCATTTCATCAATGGTGTACATCTTT
ATGATAAAAGTGAGCTTGATTGCATACAAACCATTCAGACGGTAGAGACTCTATATTGAAGCCGGCGTTCAGC
ATTCTCCATTTACGACAGCTATAGCACTGAACCCAGTCATCAACAGCCTCGACGCGATCACCACCTTTTCTCTGC
TTGAAAGATAGTTAAGTACAATGAATACTTCTGAAATGCGTAAATATCAAATTTTAGAACAATATGGAAGAATC
TCAACAATTAATGAGCACAGTGAATACCATGCTCCTGACTGCTAAGTAGAAACAAGGATTGAAGAGCAACACC
CATTACAGCCCACGTTAAACCCGTGATAGCATGATTGCATACATGTGGTAATGTATATCCTATCCATATTTGCAT
GTCTATCAATATTAAAAAAGCATAGTAACTAAAAACAATTGTATTATCTACATATCACGATGGTGTGAAATACT
ATGAATGGGTAACTCATGTACTATGTTGCTCTTCCTACTCATCAGTTTGGCTAACATTTTGTTTTTCACACTAGTC
AAATGAATTCATGATAACAGAGAGTATTTTTTCTAAATAAAAGTATGATTGAAGTAGTATGCTACCAACCTAT

>chr1A:373397850-373400412

CAAACAATCATGGCAACTACTAGGTTTCCATCATTGTTGTTTTACTCCTGTATTTTTCTCTTGTGCAATGGGTCAA
TGGCTCAGCTATTCGGGCAGAGCTTTACCCCATGGCAAAGCTCTCGACAAGGAGGTTTAAGGGGGTGCAGATT
TGATAGGCTACAAGCATTTGACCCACTTCGACAAGTGAGGTCACAAGCGGGTATCACTGAGTACTTTGATGAG
CAGAATGAGCAATTTCGTTGTGCAGGTGTATCCGTCATCCGTCGTGTTATTGAGCCTCAAGGCCTCTTGTTACCT
CAATACCACAACGCTCCTGGCTTGGTGTACATCCTTCAAGGTTAGTGTCTAATTGAATATAAAAATTGCCTTTGT
TATACTTCACTTAGGATTTAGATGTGCCAAATGTTACACCGTTCATATTTTTTAACAATGAAACAAATGTGTTTTC
TTTTAGGTAGGGGATTCACAGGGTTGACTTTCCCTGGATGCCCGGCGACCTTCCAACAACAGTTCCAACCATTT
GATCAAGCCCAGTTTGCTGAAGGTCAAAGCCAAAGCCAAAATCTTAAGGATGAACACCAAAGAGTTCACCACA
TCAAACAAGGAGATGTTGTTGCTCTACCGGCTGGCATAGTACACTGGTGCTACAACGATGGTGATGCACCGAT
TGTAGCTGTCTATGTCTTCGACGTAAACAACAACGCTAATCAGCTTGAACCAAGGCAAAAGGTAACTATACAAC
TTAATGCACACAAAATATATACAACTATTTACAAGTGATCCAACTATGATTTGAAGTATACATTAGTGGGATATT
AATGAACTCTGTTTAACTTCATCGATATAAAATTTTAGGAGTTCTTGTTGGCTGGTAACAACAAGAGAGAGCAA
CAGTTTGGACAAAACATATTCAGTGGATTCAGTGTCCAACTTCTTAGTGAGGCCCTTGGTATAAGTCAGCAAGC
GGCACAAAAGATCCAGAGTCAAAATGACCAAAGAGGTGAGATAATTCGTGTGAACCAAGGCCTTCAATTCTTG
AAACCTTTTGTTTCCCAACAAGGACCAGTAGAGCATCAAGCCTACCAACCAATTCAAAGTCAAGAAGAACAATC
AACCCAATACCAGGTAGGGCAATCACCACAATATCAACAAGGACAATCAACTCAATACCAGGCAGGACAGTCA
TGGGACCAAAGTTTCAATGGTTTGGAGGAGAATTTCTGTTCATTGGAGGCAAGGCAAAGTATCGAAAACCCAA
AACGTGCCGACACGTACAACCCACGTGCTGGCAGGATAACACATCTCAATAGCAAGAATTTTCCCACCCTTAAC
CTGGTGCAAATGAGTGCTACAAGAGTAAATTTATACCAGGTATTTATGATACTATATTCAACACACTATCTTATT

TTTAGATATTCTAAGCTTCATACAACCGGTTAATAATATGGCATACAAATAATTGCTATTGCAGAATGCTATTCT
TTCACCATACTGGAACATTAATGCTCATAGTGTCATGCACATGATCCAAGGACGTGCTCGAGTTCAAGTTGTCA
ATAACCATGGTCAGACCGTATTCAATGACATTCTTCGCCGCGGACAACTGCTAATCATACCACAACACTATGTT
GTTCTCAAGAAGGCAGAGCATGAAGGATGCCAATATATTTCATTCAAGACCAACCCCAATTCTATGGTTAGCTA
CATCGCAGGAAAGACCTCCATCTTACGTGCATTGCCCGTTGATGTCCTCGCCAATGCATACCGCATTTCTAGGC
AGGAAGCCCAAAACCTCAAAAATAACCGTGGAGAAGAGTTTGGTGCATTCACCCCTAAGTTTACACAAATGGG
CTCCCAGAGTTACCAGGACAAGGGGGAGTCAACTTCGACTGAGAAGGCATCCGAGTGAATAAGTGAGTGTAA
TGGAAAGTAGTATAGTGAAATAAAGGCATCGCATGTTTGCAGCCTAGTGGTATATAACCGCTTATCTTAATAAA
AAAGTTTCTCCGTGTTATATTGTTTTCTTGTTTCTTGTACTCTTCTTAATTTTATCTTTTATGATCAACCAACTTACC
TCTATTCTTCGATTTTCTACCACTCTTCACATATGCATATCGAGGAGATGTATAGTCCATTTGTACTAGATTTATT
TTTTGATACTATCTTTTGTGGTTGGAGTATTTCTCAGGTCCGATAAAAATGTGCAATTTACTTATGCTAGATTTTT
TGAGCAAACCTAGCCACCTAAAATTGTGAATCACATATCTTAAACTTTCTATGCCAAAGTGTGCGATTTTTCTGT
TCTATAGATCATGACATATTTTCTTTTTGGAGAAATATTATGATATCTTTTCTTTTTGGATAAAGATTATGACATC
ATTTACCATGGACATTGTTTACTGAGACATGATAAGCAACTCTTCGCAATGTGATAATTAATATTGATTGTATCC
TCAGGTCCAACTAGCGTCAAATAATGGTTTGGTATTTGGTATTGGGAGAATCATGTCTATGGCTGAAGGTGAT
GGAATAGATCATCCAATAAAATGGAATGAAACCTAA

>chr1A:373447697-373449697

ATGGCAACTACTAGGTTTCCGTCATTGTTGTTTTACTCCTGTATTTTTCTCTTGTGCAATGGGTCAATGGCTCAG
CTATTCGGGCAGAGCTTTATCCCATGGCAAAGCTCTCGACAAGGAGGTTTAAGGGGGTGCAGATTTGATAGGC
TACAAGCATTTGAACCACTTCGACAAGTGAGGTCACAAGCGGGTATCACTGAGTACTTCGATGAGCAGAATGA
GCAATTTCGTTGTGCAGGTGTATCCGTCATCCGTCGTGTTATTGAGCCTCAAGGCCTCTTGTTACCTCAATACCA
CAACGCTCCTGGCTTGGTGTACATCCTTCAAGGTTAGTGTCTAATTGAATATAAAAATTGCCTTTGTTATACTTC
ACTTAGGATTTAGATGTGCCAAATGTTACACCATTCATATTTTTTAACAATGAAATAAATGTGTTTTATTTTAGG
TAGGGGATTCACAGGGTTGACTTTCCCTGGATGCCCGGCGACCTTCCAACAACAGTTCCAACCATTTGATCAAG
CCCAGTTTGCTGAAGGTCAAAGCCAAAGCCAAAATCTTAAGGATGAACACCAAAGAGTTCACCACATCAAACA
AGGAGATGTTGTTGCTCTACCGGCTGGCATAGTACACTGGTGCTACAACGATGGTGATGCACCGATTGTAGCT
GTCTATGTCTTCGACGTAAACAACAACGCTAATCAGCTTGAACCAAGGCAAAAGGTAACTATACAACTTAATGC
ACACAAAATATATACAACTATTTACAAGTGATCCAACTATGATTTGAAGTATACATTAGTAGGATATTAATGAA
CTATGTTTAACTTCATCGATATAAAATTTTAGGAGTTCTTGTTGGCTGGTAACAACAAGAGAGAGCAATAGTTT
GGACAAAACATATTCAGTGGATTCAGTGTCCAACTTCTTAGTGAGGCCCTTGGTATAAGTCAGCAAGTAGCAC
AAAAGATCCAGAGTCAAAATGACCAAAGAGGTGAGATAATTCGTGTGAGTCAAGGCCTTCAATTCTTGAAGCC
TTTTGTTTCCCAACAAGTACCAGTAGAGCATCAAGCCTACCAACCAATTCAAAGTCAAGAAGAACAATCAACCC
AATACCAGGTAGGGCAATCACCACAATATCAAGAAGGACAATCAACTCAATACCAGCCAAGACAGCCATGGG
ACCAAAGTTTCAATGGTTTGGAGGAGAATTTATGTTCATTGGAGGCAAGGCAAAACATCGAAAACCCGAAACG
TGCCGACACGTACAACCCACGTGCTGGCAGGATAACACATCTCAATAGCAAGAATTTTCCCACGCTTAACCTGG
TGCAAATGAGTGCTACAAGAGTAAATTTATACCAGGTATTTACGATACTACATTCAACACACTATCTTATTTTTA
TTTTAAGATATTCTAAGCTTCATACAACCGGTTAATAATATGACATATAAATAATTACTATTACAGAATGCTATT
CTTTCACCATACTGGAACATTAATGCTCACAGTGTCATGCACATGATCCAAGGACGTGCTCGAGTTGAAGTTGT
CAATAACCATGGTCAGACCGTATTCAATGACATTCTTCGTCGCGGACAACTGCTAATCATACCACAACACTATG
TTGTTCTCAAGAAGGCAGAGCGTGAAGGATGCCAATATATTTCATTCAAGACCAACCCCAATTCCATGGTTAGC
CACATCGCAGGAAAGACCTCCATCCTACGTGCATTGCCCGTTGATGTCCTCGCCAATGCATACCGCATTTCTAG
GCAGGAAGCCCAAAACCTCAAAAATAACAGTGGAGAAGAGTTTGGTGCATTCACCCCTAAGTTTATACAAACG
GGCTCCCAGAGTTACCAGGACGAGGGGGAGTCATCTTCGACTGAGAAGGCATCCGACTGAATAATTGAGTGT
AATGGAAACTAGTATGGTGAAATAAAGACATCGCATGTTTGCAGCCTAGTGGTATATAACCGCTTATCTCAATA
AAAAGGTT

>chr1A:377572653-377574653

CACCATTCCACCTTCTACAATCTCTTCAAACAATCATGGCAACTACTAGTTTTCCATCAATATTGTTTTACTCTTGC
ATTTTTCTCTTGTGCAATGGATCCATGGCTCAACTATTCGGACAGAGCTTTACTCCATGGCAAAGCTCTCGACAA
GGAGGTTTAAAGGGGTGCAGATTTGATAGGCTGCAAGCATTTGAACCGCTTCGACAAGTGAGGTCACAAGCG
GGTGTCACTGAGTACTTTGATGAAAAGAATGAGCAATTTCGTTGTACTGGTGTATCCGTCATTCGTCGTGTTAT
CGAGCCTCAAGGCCTCTTGTTACCTCAATACACAATGCTCCTGGATTGGTGTACATCCTTCAAGGTTGGTGTCTA
ACTGAATATAGCAATTTCATTTGTCATACTACACTTAGGAGTTTAGATGTACCAAATATTACCCTGTTCATTTTTT
TTAACATTGAAACAAATGTGTTTTCTTTTAGGTAGGGGATACACAGGGTTGACTTTCCCGGGATGCCCAGCAAC
CTTCCAACAACAGTTCCAACCATTTGATCAATCCCAGTTTGCTCAGGGTCAAAGCCAAAGACAAAATCTTAAGG
ATGAGCACCAAAGAGTTCATCGCTTCAAACAAGGAGATGTTGTTGCGCTGCCGGCTAGCATAGTACACTGGTG
CTACAACGATGGTGATGCGCCGATTGTTGCTATCTATGTCTTCGATGTAAACAACAATGCTAATCAGCTTGAAC
CTAGGCAAAAGATAACTATACAACTTAATCCACACAAAATATATTTAACTGTTTACACGTGATCTAGCTATGGTT
TAAAGTATACACTAATGGGATATTAATGAACTCTATTTTACTTCAACGATATAAAATTTTAGGAGTTTTTGTTGG
CTGGTAACAACAAGAGAGAGCAACAATTTGGACAAAACATATTCAGTGGATTCACTGTCCAACTTCTTAGTGA
GGCCCTTGGTATAAGTCAACAAGCAGCACAAAGGATCCGCGGTCAAAATGACCAAAGAGGTGAGATAATTCG
TGTGAGTCAAGGCCTTCAATTCTTGAAGCCCATTGTTTCCCAACAAGGACCAGCAGAGCAACAATCCTACCAAC
CAATTCAAAGTCAAGAAAGACAATCAACCCAATACCAGGTAGGGCAATCAACCCAACATCAAGAAGGACAATC
AATTCAATACCAGGCAGGACAGTCATGGGACCAAAGTTTCAATGGTTTGGAGGAGAATTTTTGTTCATTGGAG
GCAAGGCAAAACATCGAAAATCCCAGACATGCTGACACCTACAACCCACGTGCCGGCAGGATAACACGTCTCA
ATAGCAAGAATTTCCCCATCCTTAACCTCGTGCAAATGAGTGCTACAAGAGTAAATTTATACCAGGTATATATG
ATACTAGATTCAACACACTATCTTATTTTTAGATAATTCTAAGCTTCATACAACTGGTTAATATGGCATACAATA
ATTGTTATTGCAGAATGCTGTTCTTTCACCATACTGGAACATTAATGCGCACAGTGTCATCCACATGATCCAAG
GACGTGCTCGAGTTCAAGTTGTCAATAACCATGGTCAAACTGTATTCAATGACATTCTTCGCCGAGGACAACTG
CTAATCATACCACAATACTATGTTGTTCTCAAGAAGGCAGAGCGTGAAGGATGCCAATACATTTCATTCAAGAC
CAACCCAAATTCTATGGTTAGCCCCATCGCAGGAAAGACCTCCATCCTACGTGCATTGCCTGTTGATGTCCTCG
CCAATGCATACCGCATTTCTAGGCAGGAAGCTCGTAACCTCAAAAATAACCGTGGAGGAGAGTTCGGTGCATT
CACCCCTAAGTTTACACAAACGGGCTTCCAGAGTTACCAGGACATCGATAGGGAATCATTTTCAACCGAGAAG
GCATCCGAGTGAATGAGCTGAGTATAATGGAAACTAGTATAGTATAATAAAGGCATCGCATGTTTGCACCCTA
GTGG

>chr1A:391973161-391975457

CCAGTTGGCCCATATGGCTAGTTGATGCGCGTCAGAATGAGGTACAACACGGCAACAAAGCGGTGAAGATAG
ATAAGGGACTTAATTGGAGAATACAAAATTAAATAAAGAACAAGAAAGAAACAAAGGATATAACATGATGAA
CTTCTTTATTAAGATAAGCGGTTATATACCACCCACTTCCACACTTGTAATGCCTTTATTTCACTATATTAATCCTC
ACCCACTCACTAATTTACTCGGATGCTTTAGTAGTCAAAAATGACTCATCCTCGTCCTGTGGATCACGTTGGGAT
CCCGTCTGGGTGAATTTTGGAGTGAATACACCAAACTCTTCTCCCCTGTTGTTTTTGAGGTTTCGGGCTTCCTGC
CTGGAAATGTGGTACGCATTGGCGAGGACATCCACAGGCAAGGCACGCAGGATGGAGCTCTTTCCTGCGATG
TGGCTAACCATGGAGTTCGGATTGGTCTTGAATGAGATATACTGGCATCCTTCACGCTCCGCCTTCTTGAGAAC
AACGTAGTTTTGTGGTACGATTAGCAACTGCCCTTGGCGAAGACGGTCATCGAATACATTCTGACCATTGTTAT
TGACAATTTGAACCCGAGCATGTCCTTGGATCATGTAGACCACACTGTGTGCATTGATGTTCCAGAATGGTGAA
AGAACGGCATTCTGCAATAGCAATTATTTATATGCCATATTATTAATCGGATGTTATGAAGCTTAGAGTATCTA
ACAATAAGATAGTATAGTGAAGGTAATATCGTATATACCTGATATAGATTTACTCTTGTAGCGCTCATTTGCAC
GAGGTTAAGGATGGGGAAATTGTTGCCATGGAGACGTGTTATCCTGCCAGCACGTGGGTTGTACGTGTCGGC
GCGTTTGGGGTTTTCGATGTTCTGCCTTGCCTCCAATGAACAAAAGTTCTCCTCCAAACCATTGAAACTTTTGTC
CGATGACTGCCCTACTTGGTATTGAGTTGATTGTCCTTCTTGATATTGGGTTGATTGCCCTACCTGGTATTGGAT
TGACTGTCCTTCTTGACTTTGAATTGGTTGGTAGGCTTGCCCTTGTTGTGACACCGTGGGCTTCAAGAACTGAA
GGCCTTGACGCACACGAATTATGTCACCTCTTTGGTCATTTTGACTCTGGATCCTCTGTGCTGCTTGTTGACTTA
TACCAAGAGCTACACTAAGAAGTTGGATACTGAATCCACTGAATATGTTTTGTCCAAATTGTTGCTCTCTCTTGT
TATTACCAGCCAACAAGAAATCCTGAGATTTCATAATTATGAAATAAAACAAAGTTCCTTCATATCCCATTAATA

CATACTTTAAAAATCATAGTTAGGTTACTAAATAATTATATATATTTATGTAGATTAATTTGTATAGTTACCTTTT
GTCTAGGTTCAAGCTGATTAGCATTGTTGTTCACGTCGAAGACATAGAGAGCTACAATTGGCGCATCACCATCG
TTGTAGCACCAGTGTACAATGCCAGCCGGCAGCGCAATAACATCTCCCTGTTTAAAGTGGTGAACTCTTTGGTG
CTCATCCTTGATATGGCTTTGGCTTTGGCTCTGGGATTGATCAAACGGTTGGAACTGTTGTTGGAAGGTCGCCG
GACACCCAGGTAAAGTCAACCCCGTGAAACCCCTACCTAAAAAAGACATAATTGTTTCAACATTAAAAACTTAT
GAATGCGGCAACATTTGGAAAATCTAAACTCGGAAGTGTATTATAGCAATGCAATCACTATATTAAGTTATACA
CAAACCTTGAAGGATGTACACTAGGCCAGGTGCGTTGTGGTACTGAGGTAACACGAGTCCTTGGGGCTCAATA
ACGCGACGGATGACAGATACCCCGGTACAACGAAATTGCTCATTCTGCTCATCAAAGTACTAAGTGACACCCG
CTTGTGACCTCACTTGTCGAATTGGTTCAAGAGTTTGTAGCCTGTCGAAACTGCATCCCCTTACACCTCCTTGCC
GGTAGCTTTGCCATGGAGTAAAGCTCTGTCCGAATAGCTGAGCCATGGATCCATGGCAAAAGAGAAAAATGTA
AAAGTAAAACAACATTGATGTAAGTCTAGTAGTTGCCATGATTGTTTGAAGAGATGGTTGTTGGAGGTGGATT
GGTGCTAGTTTCTTAATTGGAAGCTTGGTTTTATAGCCACAAAAAGTAGTTTAGGATGCACATGGTGACATATT
TGCTAATAGAGATAAGAAGATATGCTGGTGACTCAGGCGTTGTTTAACTGGATATAAATTTTACTCAAAAGTAT
A

>chr1A:392059423-392061600

AGATAAGGGAGTTAATTGGAGAATACAAAATTAAATAAAAACAAGAAAAAAACAAATGATATAACATGATGA
ACTTCTTTATTAAGATAAGCGGTTATATACCACCCACTTCCACACTTGTAATGCTTTATTTACTATATTGGTCCT
CACACACTTACTAATTTACTCGGATGCTTTAGTAGTCAAAAATGACTCATCCTCGTCCTGTGGATCACGTTGGGA
TCCCGTCTGGGTGAATTTTGGAGTGAATACACCAAACTCTTCTTCCCTGTTGTTTTTGAGGTTTCGGGCTTCCTG
CCTGGAAATGTGGTACGCATTGGCGAGGACATCCACAGGCAAGGCACGCAGGATGGAGCTCTTTCCTGCGAT
GTGGCTAACCATTGAGTTCGGATTGGTGTTGAATGAGATATACTGGCATCCTTCACGCTCCGCCTTCTTGAGAA
CAACGTAGTTTTGTGGTACGATTAGCAACTGCCCTTGGCGAAGACGGTCATTGAATACATTCTGACCATTGTTA
TTGGCAACTTGAACTCGAGCATGTCCTTGGATCATGTAGACCACACTGTGTGCATTGATGTTCCAGAATGGTGA
AAGAACGGCATTCTGCAATAGCAATTATTTATATGCCATATTATTAATCAGATGTTATGAAGCTTAGAGTATCTA
CAAATAAGATAGTATAGTGAAGGTAATATTGTATATACCTGGTATAGATTTACTCTTGTAGCGCTCATTTGCAC
GAGGTTAAGGATGGGGAAATTGTTGCCATGGAGATGTGTTATCCTGCCAGCACGTGGGTTGTACGTATCGGC
ACGTTTGGGGTTTTCGATGTTCTGCCTTGCCTCCAATGAACAAAAGTTCTCCTCCAAACCATTGAAACTTTTGTC
CGGTGACTGCCCTACTTGGTATTGAGTTGATTGTCCTTGTTGATATTGGGTTGATTGCCCTACCTGGTATTGGAT
TGACTGTCCTTCTTGACTTTGAATTGGTTGGTAGGCTTGCCCTTGTTGTGACATCGTGGGCTTCAAGAACTGAA
GGCCTTGACTCACACGAATTATCTCACCTCTTTGGTCATTTTGACTCTGGATCCTTTGTGCTGCTTGTTGACTTAT
ACCAAGAGCTGCACTAAGAAGTTGGATACTAAATCCACTGAATATGTTTTGTCCAAATTGTTGCTCTCTCTTGTT
ATTACCAGCCAACAAGAAATACTGATATTTCATAATTATGAAATAAAACAAAGTTACTTCATATCCCATTAATAT
ATACTTTAAAAATCATAGGTAGGTTACTAAAGAATTATATATCTTTATGTAGATTAATTTGTATAGTTACCTTTTG
TCTAGGTTCAAGCTGATTAGCATTGTTGTTTACGTCGAAGACATAGATAGCTACAATTGGCGCATCACCATCAT
TGTAGCACCAGTGTACAATGCCAGCCGGCAGCGCAATAACATCTCCCTGTTTAAAGTGGTGAACTCTTTGGTGC
TCATCCTTGATATGGCTTTGGCTTTGGCTCTAGGATTGATCAAACGGTTGGAACTGTTGTTGGAAGGTCGCCGG
ACACCCAGGTAAAGTCAACCCCGTGAAACCCCTACCTAAAAAAGACATAATTGTTTCAACATTAAAAACTTATG
AATGTGGTAACATTTGGAAAATCTAAACTCGGGAGTGTATTATAGCAATGCAATAACCATATTAAGTTATACAC
AAACCTTGAAGGATGTACACTAAGCCAGGTGCGTTGTGGTACTGAGGTAACACGAGTCCTTGGGGCTCAATAA
CGCGACGGATGACAGATACCCCGGTACAACGAAATTGCTCATTCTGCTCATCAAAGTACTGAGTGAGACCTGC
TTGTGACCTCACTTGCCGAATTGGTTCAAGAGCTTGTAGCCTGTCGAAACTGCACCCCCTTACACCTCCTTGCCG
GTAGCTTTTCCATGGAGTAAAGCTCTGTCCGAATAGCTGAGCCATGGATCCATGGCAAAAGAGAAAAATGTAA
AAGTAAAACAACACTGATGGAAGTCTAGTAGTTGCCATGATTGTTTGAAGAGATGGTTGGAGGTGGATTGGT
GCTAGTTTCTTAATTGGAAGCTTGGTTTTATAGCCACAAAAGGTAGTTTAGGATACACATGGTGACATATTTGC
TAATAGAGATAAGAAGATATGCCGGTGA

>chr1A:457675077-457676202

TTTCCAGAAATGTTTAAGGATTGGTAGTGTTCGGATTTCTTGTAAAGTTATTTCAGACCCAAATTAGGGTTTTCT
TCGATTTAGTAGAAAATAGAAATAGAAAGACTCCATGATGATCAGTCATCAGGTTAAGATGGATCTGAACCAA
TCCTTTTTTTCTATACACATAGCCGACGGTTAAACAACTTATTAGAAACGCAAGATAGCTAAAACGAAATGCTA
GAATGGCCGCGTTTAAAGGATGCCCTCAGCGTCGAGGAACATGTGCTAGGGTGTAAGTTCGAAATCTCATCTG
ACCATCTATTTCTCACTGATTGGGAGATTGGTCGTTTAGATCATAACTTCAAACATTTTTTTTGGAAGTATCCA
AATTTTTACGCTAATACTCCATAGATAGTTCGAATTGGATAGCAGCAATAATCAATTTTAGCGCGAATTGTTTG
GAGGGGGAGCCTATCCTTTTTGATGCATTCGACACGTGCAATTTTTTCGTCTGAGATGATCCGCAATTTTGACTT
TAACTTTTCTTATATCCGCTTTTTTAGTTATAATAATATAAATAGTCTGGTCCGGCCTCAGCAAACATGTTTTTTA
TGTTTTTTGAGGGGAGCCTCCGCGAACACGTCAGAATAGTCGGTAAAAGCCATCGACCATCCAGTATCCTTCCT
CTTTCTCCGTGATGATATTAGGCCCATGGGTTGTGAGCGCTAGGCAGCAGCCTACACCGTGATATTGGGCAGA
GAAGCTCTCCTATCAGCAGGCGGTTGGGGGATGAATTTGTTAGCATAAAACCACCACATTACAAGTGAAATTT
TAATTGTATTATCACTTTTTTTTAGACAAAAATCTACCACATTTTTTCTTGGTTGTCTCATAAAATACTAAACTGG
CATTGAAGACGAATTGACAGCCATTCTGATATTAGGGACCACCTGTAAGGCTGATGGCAATATTGATTAAAAA
TATAAAAATCTCGTAAAAAGCAAAGAAGAGAGGTGTTTTCTTCTTCCTCCGTGCAGACCCCTCCCACCTGAGCA
CGCCGCCGGTGTGCCAGAGCCCACCGGTTGCCGAGGCCGCTCCACCGGACGTTGTCGCCGCCCCAGATGTGTA
GCCCGCGTCGCCGC

>chr1A:489689388-489691494

TGGATTGCAACACTGTGACCACACGGTCACATTTATTTATTTTCTAACCGGGGTGCATCCATCCCCAAACAACAT
GCTGAGTTACAAGAGTTGTAGCCTAGTACTGGCCGGCGGAGAAGGCCCTGCCCTGAAGCTGACACATGGCCG
GCAGCTGCGCCGCGACTTGCCGCGCCTTAGCCACCACCCTCGCTTGCCTTAGGCGGGCTGCCTGCTGCTCGTGC
TCGGTGCCATAACGATGAGGAGACGAGGACTCGCCGAACCCTGTCTGCTGGCCTTGCCTGCCAAAGTACGGAC
CTTGTCCCTCTTGCTGCTGAGCGGTTTCACCGAGCTCATGTTGGGCTGCAGGCTGCCGGCCATGGCGGGAGAT
ATCGATGATGCTGTGTAGGTGGGGGGAGCCAAAAGACTCATGTCTTCCCCGCTGCTGGGCCGTGGACTGGCC
GCAGACCCCTCCTTCCTCCTGTTGTTGCTGCTGCGGAAAAGTTTGGCTGTGGTGGTACGACCCTTGCTCTTGGT
GTTGGGGACGATAAGTTTGGTGAGGAGGACCGTACATCCCTTGCTCTAGCTGCTGCTGCTGATAAGTTTGGCT
AGGACCGTACATCCCTTGCTCTTGCTGCCCATAAGTTTGGCTAGGGCCGTACATCCCTTGCTCTTGCTGTTGCTG
CTCCTGCCCTTGCTCTTGTTGTGGATAGGTTTCACTTGGGTAGTAGGATCCTCTTCCGACTGGCGCCACCACTTG
CTGCTCGTAGTGCCTCACGATCTGGTGGATGGCGGCGGCACGACACTCAGGACTGACGTGCCGGAGCTGCTG
ACAGCATTGCCCCCGCACCTCTGATCTCCACTCCGATGATCTCAGGAGCGACGATCGCGGGTGGAAGAAGGGC
AGCTGGACGGCCAGTTGGTGGTCCACGACCTGACGGCACGCCTCCAGCGAGCTCTCCTGGAGCTGACGCTGG
CAGTGTGGCTGCACAGTGTTATCCCCTTCAGCGGTGGATAGAGCAACGAGGGCCACAACCACCGCCATGAACA
AGACCAGTTTCGCCATTTCTGTGGTGCTAACGATCAATTAATCTCTAGTTTGTGGTGCTCGGTGTTATCGGTGAT
GATGAGACTGTGGAGATTGTCTGGGATTTTATAGGAACGGGGTGAAGGTTGAGGACCTTGGATTGTGTGCAC
GATGAGGTTAGTCTAGATTAAGAAGAAATACAAGAATAACAAGTTTTGGTACAGAGGTAGTAATCGGTTTAAT
TGTGCAGAAATGACACCTCGATCACGTGCACCTAACTTCTCACAATAGCTCTCGCATGCAAATTGATAAGATTG
TTCTGGTGTGGTATTTGGAAAAGCGTGCACATTTGTATTGGTGATCTATTTCTTTATCTACAAAAAGTGTTCATG
GTTCTTGGATTTTAGGAGATGATGTGTCTTTTAGTCATTAGGTTTATCTCATGTAGTTTCGACTTGCAGGTTACA
CAAATCCATGCTACATGACTATGACTCACATCATCTATCCAGTATGATGCACAGTGCCATCCATGCTTTTTTTTCC
GAAAGGGGGAAAACCCCGGCCTCTGCATCGTTGATGCACACAACCTTTATTAAAACCAAGATCCAACATCCAA
CGGTCAACACATATTACACATCATGGGTCGCTTAAAGTTGATACATGGATCAACCAGTGAAAAATATGAAGCA
AACGAAAATACATGACGCACAATGTCATCCATGCTCTGACGCACATATCAATCCATGCAGTGAAGCGACACAC
CTAAAACCAAATTTCTGTAAAAAAGTAGTCCAATGTATACAACTAGAGCAACTTCTTCTTTCCGTGATAAAATTC
AATTATGGTGATCGTTTGTAAGTCTTGTCGACATACATGAGTTTCAGAGAAAATTCAATGTCAAACAATTTGAT
TTTTTCCATTGGGATGGGAAAATTTCCACGTCCCAAACGACTCCATAAATGCAGATCGTTTGATCAGTCAAACC
ATGCAGCTTTGGGGCATGTTTATGCCCCCAAACCAAAATTCCTGGTGCTCACTTCCTAGGGGAAATTGGCGGA
GGTGTATGAATTGCATGTTTTCTGATTGATTTTGAGGATGAAGGG

>chr1A:489692737-489695737

GTCAATATATTCAACATTAGAGATATTTATGTCTTTATTATCGTTATTATTCACGCATGAACGAATATGATCACA
CATCCACCACACTTATTATGAGGGCTAATTCCATAGATTACTTCAGCATGGTAGCTAGTCTAATATTGGCCGCC
GGAGAAGACACTACACTCCATGGGCTCGATCTGGCACACGGTGGGTAGCCCCGCCACGTACTGCCGCGCCTGC
ATCAGCCTCACGCGAATGACATGCTGCATCTGCTGTGCCCCCTCGCCATAGATCTCCTGTCCTCCCTGACGCCTC
GACGTCTCGCCGTAGTAGATTCCTCCCTGCGGCTGGCTTTCAGCTGTCTCCCCGCCGTAGTACTCCTGTCCCGCC
TCCTGCTGCTGCCGCGGTGGCATCTCGTGGCGGAGCACCTGTCCTTGCTGACCACGCCCTTCTTCGCCATGGTA
CCCTCCTCCCTGCTGATGGTGTCCCGCCGTCTCACCACTGCAGTGTCCCTGCTCTTGCTGCTCCCCCGGTGAGCC
ACGGCGCCCTCCTCCCAGCGGCGGCATGGTCTCCTCGTAGTCCCTCACCATGCCGCGGATGGCGGAGCAGCGG
CACCCACGGCTGACGCTATGGAGCTGCCTGCAGCAGCGCTCCCTGGTGTCCCACTCCGTCTGGAGCAGCGGGA
CGCCGACGGCACCCTCGCGCCCGCCATCGATCAGCTTATCCTGGAGGATCTGCCGGCACGCGTTGAGCGGCTT
CTGTTGGACCTCACGCCGGCACATCACGTCCCTGTAGCTCTGCTCGGTCAGGTCCTGGGCCACGGAGGCGGCC
ATCAGGGCCGCAAAGAACACCGCGAGGAAGACGAACTTGCCCATATTTCTCTCGCTAGCTTGCTTGCTCTTCTC
GTTTCTGGTGTACTTAATACCTGGTGGTGGCGAGAGATGTGGAAGAGACGGTCAAATTTTATATAGGAACATA
TGGTGATGATTCGGCACCATGTATACGGTTGTGTGCACGGTGATCAAGCTAGTTGGGTTGAAGAAGTTTTAGT
TGAAGTGCAATGCTTGGAAGAGTGAAAAGGGTACAAATTAAGGGTTTTGGACTTCGCATTAGTCACTTTTGGG
TAAGGTATAGCTTTACATCATCTCTCATATATACAATGGCAGATGTATTTGCTGTAATGGAAGTACTCCCTCCGT
CTCAAAATAGGTGTCGCAGGTTTATCAAAATTTAAATGAATCTATATACTAAATAGTGTCTAAATTCATCCAATG
ACAAATCCAAGACATCTATTTTGAGACGGAGAGAGTATATGATGTTTTTTTTTACAGAAAAAAGTATATGATG
TTGTGTAGAGTGTCAAGCTGCAAAAGTATGGATAGTTGTATGTATCTGTTGATTTTTTAGATTGAGTATGTACG
TGGCATGATTGTGTGTGCTGCAGTGCACGGCGAGTTTCATACAAGATATACTATATATCAACAAAATGCTTTCG
TGAAAGTTTTGAGATTTTTGTAATAGACAAGAAAGTAAGATTTGGAGCTTTGCTAACATATCAACAGAATGCTT
GCGTGAAAGTATTTGAGTTGGAAAGTTGCTTTTACTGTCAACACTACCATGTATCATGGATGTACTCGATCCGC
ATGGAACACGTTTCACACAAGCAGAGATGATGGTGCTTGTACTTTATAGGGATTCACGACATTTATGAGATTTC
TCATATCTATGATCTTGTTTGTGCTTTCCGCATCATGTAATCACACAAACCCATTGTGGGCTTTGTTTGAACATG
AAAATTTTCCAATAACAAGTCTATTGTGTATAGGAATAATATCTAAAATAGGGACTCCAATGTCGGCGATGAGG
TGTGCTTCGGTGTCCCCCCTTCAAACATAAGTTTGTATGAGGAAATTTTCTTTTGACCTATCGAAACCCATATCT
AGGCCCATATAAGTGTACTCAGTCGAGCTGGGTATACTCCACGTTTTCACCGGGCGATCTCACCTCCCCTTATG
CAGATCTACATCTTTGCATGCCATGTTTTGAAATTTTATTACTACTGTTATATACCTACCGATTTCCGCCTCCCGC
AGGCACAAGATTATACGTGTACCGGTGTATGGTGAGTATATGTCTAAGCAGACCATACATGATGGGTGGGTTT
GCACGGTCTTCGAAACGAATCGGTATCGTGCGATGCTAGCCGTCCACGAGTTTATGTTTATCAAAGTTAGAAG
AGGAAGGGGGGACACCGAAGCAAAAGTGGTCGGCGATTTGGCAAACAATTCCAAAAGCAGTGGATCTCCATG
GTAGCAAATCTCGTGCACCTACCGGAAAACGGCTCGTTGTCGTGTATTTAGAACTTTGTCGTGTGCCTAGTTCC
GCGGAATACGACAAAGGCGGACTTTGCCGTGTGCCACACAAAAACACACGGTAACTATCGAGAAAATGACAG
GGCTAAGATCTGAACTCGACAAACAATGAGGAAAACACACGGCAAAGTCACAACACAAGGCAAACACACGAC
AAAGAATATATCATGGCATGGCACACTACTCTCTGTCTCCTTATTCCAATTCCCTCTCTCCATATTATCCCCACTA
TTCCTTATCCCGATGTTTGCTATGCACGGGCTCTCACGCGCTGCTCGACTTTGCTCACGATTATCGCCTCCGACG
GTCAGCGCTCGCCGGCAATTGCGCCATCCTATCTGATGGCCAGCACTCGTCGTGCTCTAGCGGTGGCGGCGGT
GGCACGCAGAGGCGGCGGTGAAGGCAAGCAGTGGCGGCAATGACTACACGCTGCGATGGCGGCTAGCGGTG
GCGACGACAAGCAACGGCGAAGGCTAGCGTTGTCGACAGACTGTGACGATCTCTCTGACGGCAAGTAGCGGC
GGCGGCCAGCGACATCGCCCTCCCCACGTGATGCTTCGGTCCTGCTTGTGCGGTGCCCCAAGCCACCAAGCAC
ATTGTGCGGTGTGGGCGTGGGCAGGGAGCCAGGGCGATGTGGGTGGCGGC

>chr1A:522777998-522780444

ATGGCAACTACTAGTATTCCATCAGTGTTGTTTTACTCTTGTATTCTTCTCTTGTGCAATGGATCCATGGCTCAGC
TATTCGGGCAGAGCTTTACTCCATGGCAAAGCTCTCGGCAAGGAGGTTTAAGGGGGGTGCAGATTCGATAGGCT
GCAAGCATTTGAACCACTTCGACAAGTGAGGTCACAAGCGGGTGCCACTGAGTACTTTGATGAGCAGAATGA

GCAATTTCGGTGTACCGGTGTATCTGTCATCCGTCGTGTTATCGAGCCCCAAGGCCTCTTGTTACCTCAATACCA
CAACGCTCCTGCCTTGGTGTACATCCTTCAAGGTTAGTGTCTAATTGAATATAGAAATTGCCTTTGTTATACTGC
ACTTAGGATTTAGATGTGCCAAATCTTACCCCATTCATATTTTTTTAACATTGAAACAAATGTGTTTTCTTTTAGG
TAGGGGATTCACAGGGTTGACTTTCCCTGGATGCCCGGCGACCTTCCAACAACAGTTCCAACCATTTGACCAAG
CCCAGGTTGCTCAGGGTCAAAGTCAAAGCCAAAATCTTAAGGATGAGCACCAAAGAGTTCACCGCATCAAACA
AGGAGATGTTGTTGCACTACCGGCTGGCATAGTACACTGGTGCTACAACGATGGTGATGCGCCGATTGTAGCT
GTGTATGTCTTCGACGTAAACAACAACGCTAATCAGCTTGAACCAAGGCAAAAGGTAACTATACAACTTAATCC
ACAGAAAAGATATATAATTGTTTACAAATGATCTAGCTATGATTTAAAGTATACATTAGTGGGATTTTAATGAA
CTATGTTTTATTTCATCGATATAAATTTTCAGGAGTTCTTGTTGGCTGGTAACAACAAGAGAGAGCAACAGTTT
GGACAAAACATATTTAGTGGATTTAGTGTCCAACTTCTTAGTGAGGCCCTTGGTATAAGTCAACAAGCAGCACA
AAGGATCCAGAGTCAAAATGACCAAAGAGGTGAAGTAATTCGTGTGAGTCAAGGCCTTCAATTCTTGAAGCCC
TTTGTTTCCCAACAAGGACCAGTAGAGCAGCAAGCCTACCAACCAATTCAAAGTCAAGAAGGACAATCAACCC
AGTACCAGGTAGGGCAATCAATCCAATTTGAAGAAGGACAATCAAGTCAATACCAGGCAGGACAATCAAGGG
ACCGAAGTTTCAATGGTTTGGAAGAGAATTTCTGTTCATTGGAGGCAAGGCAAAACATCGAAGACCCCAAACG
TGCTGACACATACAACCCACGTGCTGGTAGGATAACACGTCTCAATAGCAAGAATTTTCCCATCCTTAACCTCG
TGCAAATGAGTGCTACAAGAGTAAACCTATACCAGGTATATATATGATACTACATTTAACACACTATCTTGTTTT
TAGATATTCTAAGCTTCATATAACTGGTTAATAATATATCATACAAATAATTACTACTGCAGAATGCTATTCTTTC
ACCATACTGGAACATTAATGCGCACAGTGTGATGCACATGATCCAAGGACGTGCTCGAGTTCAAGTTGTCAAT
AACCATGGTCAGACCGTATTCAATGGCATTCTTCGTCGAGGACAACTGCTAATCATACCACAACACTATGTTGT
TCTCAAGAAGGCAGAGCGTGAAGGATGCCAATATATTTCATTCAAAACCAACCCCAGTTCCATGGTTAGCCACA
TCGCAGGAAAAAGCTCCATCCTACGTGCATTGCCTATTGATGTCCTCGCCAATGCATACCGCATTTCTAGGCAG
GAAGCCCGAAACCTCAAAAATAACCGTGGAGAAGAGTTCGGTGCATTCACCCCTAAGTTTACACAAACGGGCT
TTTAGAGTTATCAGGACACCGATGGGGAGTCTTCTTTGACTGAGAAGGCATCTGAGTGAATAAGCTAAGTGTA
ATGGAAACTAGTATTATGGAATAAAGGCATCACATGTTCGCAGCCTAGTGGTATATGACCGCTTATCTCAATAA
AAAAGTTTCCTCATGTTATATTGTTTGCTTGTTTCTTGTACTCTTCTTAATTTTATGTTTTGTGACCAACCCCTTGC
CTCTCTTCTCGAATTTCTGCCACTGTGCACCTCAGTGTTCATGCAGCGTACAGGCCATTTGGAATAGATTTTTTT
GCGGTACAATGTTTTTGTGGTGCGAGTATTTTTCAGGTCCTACAAAAACCTTCAATTTTGCTAATTACGTGTGCT
AGATTTTTTCAGAAATCCCAGCTACCTAAAATTTTGAATCACATACCAATTTTCTATGCCCAAGTTGTGCTGCTTT
AGTAAACAAAACATGTAGTTTTTCATTCTATAGATTATCACTGAACTTGCTCCCCGACGAAATGTACCGAAGAG
GCAACCCTAGGGAAGTTCACTCGTTTGTTGTCCGGTCTGGCTAAAATAGAAAACCAGCGACAAAAAGTAACAA
AA

>chr1A:522788315-522790444

ATGGCAACTACTAGTTTTCCATCAGTGTTGTTTTACTCTTGCATTTTTCTCTTGTGCAATGGATCCATGGCTCAGC
TATTCGGGCAGACCTTTACTCCATGGCAAAGCTCTCGGCAAGGAGGTTTAAGGGGGTGCAGATTTGATAGGCT
GCAAGCATTTGAACCACTTCGATAAGTGAGGTCACAAGCGGGTGTCACTGAGTACTTTGATGAGCAGAATGAG
CAATTTCGGTGTACCGGTGTATCCGTCGTCCGTCGTGTTATCGAGCCTCAAGGCCTATTGTTACCTCAATACCAC
AACGCTCCTGGCTTGGTGTACATCCTTCAAGGTTAGTGTATAAATGAATATAGAAGTTGCCTTTGTTATGCTAC
ACTTAGGATTTAGATGTGCCAAATGTTACCCCGTTCATATTTTTTTAACATTGAAACAAATGTGTTTTCTTTTAGG
TAGGGGATTCATAGGGTTGACTTTCCCTGGATGCCCGGCAACCTTCCAACAACAGTTCCAACCATTTGATCAAG
CCCAGTTTGCTCAGGGTCAAAGCCAAAGCCAAAATTTTAAGGATGAGCACCAAAGAGTTCACCGCATCAAACA
AGGAGATGTTGTTGCGCTACCGGCTGGCATAGTACACTGGTGCTACAACGATGGGGATGCACCGATTGTAGCT
GTCTATGTCTTCGACGTAAACAACAACGCTAATCAGCTTGAACCAAGGCAAAAGGTAACTATACAACTTAATCA
ACACCAAAGATATATATAATTGTTTACAAGTGATCTAGCTATGATTAAAATTATACATTAGTGGGAAAGTAATA
AAATCTGTATTACTTCATGGATATAAAATTTCAGGAGTTCTTGTTGGCTGGTAACAACAAGAGAGAGCAACAGT
TTGGACAAAACATATTTAGTGGATTCAGTGTCCAACTTCTGCGTGAGGCCCTTGGTATAAGTCAGCAAGCAGC
ACAAAGGATCCAGAGTCAAAATGACCAAAGAGGTGAGATCATTCGTGTGAGTCAAGGCCTTCAATTCTTGAAG
CCTTTTGTTTCCCAACGAGGACCAATAGAGCATCAAGCCTACCAACCAATTCAAAGTCAAGAAGGACAATCAAC

CCAATACCAGGTAGGGCAATCAACCCAATACCGGGTAGGACAATCAACCCAATATCAAGAAGGACAATCAACT
CAATACCAAGAAGGACAGTCATCGGACCGAATTTTCAATGGTTTGGAGGAGAATTTATGTTCATTGGAGGCAA
AACAAAACATCGAAAACCCCAAACGTGCCGACACATACAACCCACATGCTGGCAGGATAACACGTCTCAATGG
CAAGAATTTCCCCATACTTAACCTCGTGCAAATGAGTGCTACAAGAATAAATTTATACCAGGTATATATGATACT
ACATTCAATACTCTCTTATTTTTAGATATTCTAAGCTTCATACAACCGATTAATAATATGACATACAAATAATTGC
TATTGCAGAATGCTATTCTTTCACCATACTGGAATATTAATGCGCACAGTGTCATGCACATGATCCAAGGAAGT
GCTCGTGTTCAAGTTGTCAACAACTATGGTGAGACCGTATTCAATGACATTCTTCGTCGAGGACAACTGCTAAT
CATACCACAACACTATGTTGTTCTCAAGAAGGCAGAGCGTGAAGGATGTCAATTTATTTCATTCAAGACCAACC
CCAATTCCATGGTTAGCCACATCGCAGGAAAGACCTCCATCCTACGTGCATTGCCTGTTGATGTCCTCGCCAAT
GCATACCGTATTTCTAGGCAGGAAGCCCGGAGCCTAAAAAATAACCGTGTTGAAGAGTATGGTGCATTCACCC
CTAAGTTTACGCAAACGGGCTCCCAAAGTTACCAGGACGAGGGGTACTCATCTTCGAGCGAGAAGGCATCCGA
GTGAATAAGTGAGTGTAATGAAAACTAGTATAGTGAAATAAAGGCATTGCATATTCGCAGCCTAGTGGTGTAG
AACCGCTTATCTCAATAAAAAAAGTTTCTCCATGTTATATTGCTTGCGTGTTTCTTATAGTCTTCCTAATTTTATG
TTTTCCGACCAACCCCTTACCTCTCTCCTTGATTTTATGCTATTGTGCACATACCTGCT

>chr1A:522802332-522804544

ATGGCAACTACTAGGTTTCCATCAGTGTTGTTTTACTTTTGTATTTTTCTCTTGTGCAATGGATCCATGGCTCAGC
TCTTCGGATAGAGCTTTACTCCATGGTAAAGCTCTCGACAAGGAGGTTTAAGGGGGTGTAGATTTGATAGGCT
ACAAGCATTTGAACCACTTCGACAAGTGAGGTCACAAGCAGGTGTCACTAAGTACTTTGATGAGCAGAATGAG
CAATTTTGTTGTACTGGTGTATCATTCATCTGTCGTGTTATCGAGCGTCAAGGCCTCTTATTACCTCAATATCGC
AATGCTCCTGGATTGGTGTACATCCTTCAAGGTTAGTGTCTAATTAATGTTGGGTTCGTAGCATAGAAAACAAA
AAATTTCCTACGAAGAGAACTATACGCCAAGATCTATCTACTAGATATAGAGCAATGGGTGGATATACATCCAT
GGAGAACTTACCCTCGAAGACAATAGAGTTTTGTTAATGAGTTGGAACTCGTGGATGATGTAGTCGTACGCTT
GCCGATCTCAAGATCGTGTGGAAGTCCAAGCGCCGCAAATCAGCAGCGCATCCATCCTCGCACACACGTTCAA
GCCACCGGACAACCTCCACCTCCTCGCTCCAGCGAATCAGCGGAAGCAGCAACTCCTTCCAGACGCAAGGCCA
CCGGGAACATGTATCTCGCTAATCCGAGACCATGGGGGAATATCTAGAGGAGGAGAGAGATCCTTCACGAGG
AGGTGTGCTTTGTGAGGGAGGAGAGGGGGAATTTTATAGGAGAGGGGGGGGCTCCTCCCCTACCATGGCCG
GCGGCCAAGGAGGGTGCCCCCACCTCTTCCTCATTCATGTGGTGGAAGCTCCACTCACATCAATTTTGACTCCA
CCCAAAATTCCACTCACATGCATTAAATTTCCACCAAATTTAATGCAACTCTCAATTAATAAAACTCTTTTATTTA
TTTTATTCAGAATTTTGTTAATTACTTTAATTAACAAACATTCACATAAAACCCCTTGCAACATTTCAATATCACA
TGCAAAACCACTTTCTGCAATTTCACCCGAAACAATTCTGGTTTCTATCGAAATAATTCCTGCGCTTTGCAATCCT
TTCGTATGAAACATATGTGCTCCTAAATGGTGTCTGACAACCCTTAAGTGTGTCACCCTACGGTTCACGAATAA
TGCAGACATGATGGAGACTCCTCTCAGATCAATAATCAGAAGCGGGACCTGGAGATCCATACTGACTCCTGCA
CATTCAACGATGACTTCTATGATCGTGAGAACTTTTAACGCTAGTAACTTAATCCCTTTGTCTCATGATATAATA
CGTGTCCAAGATTCGATCATCAGTATCCAAATACCTAGTTTGATCTCGTTGCCGACATGCACTCTTTTCTTGTTCC
CGTGATATAACATTCTAGTGATCACATCACTTGCTTGCAAGCTATCTTAATGTGATATCACCGAGAGGGCCCAG
AGTATATCTATCCATCATCGGGATTGACAAATTCCACTCTTGATCCATGAGCCTCAACATAACACCTTCGTAGCA
CTGAACCCACCTTTATAGTCACCCTGTTACGGTGTGACGTTTGATGTTAGCAAGATACTCTTCCGGTGTTAGCAA
TTAACATGATCTCATGGTCTAAGGACTAAGATTACTTCGTAAGATGTTATAGCAATATAAACATAATGACTTTG
ATCGTAATGCTATACTTCGGATGAATGTTCATCACATCATTTCACAAATGTTGTGACCTCGTTATTAACAACATC
CAATGTCCATGATCAGGAAACTTTGATCATCTTGTTAATCAACGAGCTAGTCTATAAGAGAGGCATAACTAGGA
ACCCTTTTTTTTATTAAACACACATGTATTGACGTTTCCTATCAATACAATTATAGCATGCAAAATAAACATCTAT
CATATACCAGGAATACATAATAATAACTTTATTATTGCCTCTGAGGGCATATTTCCTTCAGTCTCCCACTTGCACT
AGAGACAATAATCTAGTTTACATAGTGATAAATCTAACACCCATGGAGTTCTGGTGTTGATCATGTTTTTCCCTA
GGGAGAACTTTAGTCAACGGATCTGCTACATTTAGGTCCATGTGTACTTTACAAATATTTATATCTCCATCCTTG
ACATGCTCGCGGATGGAGTGAAACTGGCGCTTAATATGCTTGGAATTCTTGTGAGA

>chr1A:522815609-522817744

ATGGCAACTACTAGGTTTCCATCATTGTTGTTTTACTTTTGTATTTTTCTCTTGTGCAATGGATCCATGGCTCAGC
TCTTCGGGCAGAGCTTTAATCCATGGCAAAGCTCTCGACAAGGAGGTTTAAGGGGGTGCAGATTTGATAGGCT
ACAAGCATTTGAACCACTTCGACAAGTGAGGTCGCAAGCAGGTATCACTGAGTACTTTGACGAGCAGAATGAG
CAATTTCGTTGTACCGGTGTATCCGTCATCCGTCGCGTTATCGAGCCTCAAGGCCTCTTGTTACCTCAATATCAC
AATGCTCCTGGCTTGGTGTACATCCTTCAAGGTTAGTGTCTAATTAAATATATAAATTGCCTTTGTTACATTGCA
CTTAGGATTTAGATGTGCCAAATGTTACCCCATTCATTTTTTTAACATTGGAACACATGTGTTTTCTTTAAGGTA
GGGGATTCACAGGTTTGACTTTCCCTGGATGCTCGGCGACCTTCCAACAACAGTTCCAACCATTTGATCAATCC
CAGTTTGCTCAGGGTCAAAGCCAAAATCTTAAGGATGAGCACCAAAGAGTTCACCGCATCAAACAAGGAGATG
TTGTTGCGCTGCCAGCTGGCATAGTACACTGGTGTTACAACGATGGTGATGCACCGGTTGTAGCTGTATATGTC
TTCGACATAAACAACAATGCTAATCAGCTTGAACCAAGGCAAAAGGTAACTATGCAACTTAATCCACACAAAA
GATATAATTATTTGCAAGTGATCTAGCTACGATTTAAAGTATACATTAGTGGGATATTAATGAACTCTATTTTAC
TTCATCAATATAAATTTTCAGGAGTTCTTGTTGGCTGGTAACAACAAGAGAGAGCAACAGTTTGGACAAAACAT
ATTCAGTGGATTCAGTGTCCAACTTCTTAGTGAGGCCCTTGGTATAAGTCAACAAGCAGCACAAAGGATCCAG
AGTCAAAATGACCAAAGAGGTGAGATAATTCGTGTGAGTCAAGGCCTTCAATTCTTAAAACCCATTGTGTCACA
ACAAGGACCGGAGCAAGCCTACCAACCAATTCAAAGTCAAGAAAGACAATCAACCCAATACCAGGTAGGGCA
ATCAACCCAATATCAAGAAGGACAATCAACTCAATACCAGGAAAGACAATCATATGACCGAAGTTTCAATGGT
TTGGAGGATAATTTCTGTTCAGTGGAGGCAAGGCAAAACATCGAAAACCCTAAACATGCTGACACGTACAACC
CACGTGCTGGCAGGATAACACGTCTCAATAGCAAGAATTTCCCCATCCTTAACCTCGTGCAAATGAGTGCTACA
AGAGTAAATTTATACCAGGTACATATGATGCTACATTCAACACACTATCTTATTTTTAGATATTCTAATCTCATAC
AACTGGTTAATAATATAGCATACAAATAATTGCTATTGCAGAATGCTGTTCTTTCACCATACTGGAATATTAATG
CGCACAGTGTCATCCACATGATCCAAGGACGTGCTCGTGTTCAAGTTGTCAACAACCATGGTCAGACCGTATTC
AATGACATTCTTCGTCAAGGACAATTGTTAATCATACCACAACACTATGTTGTTCTCAAGAAGGCGGAGCGTGA
AGGATGCCAATATATTTCATTCAAGACCAACCCAAATTCCATGGTTAGCCACGTTGCAGGAAAAACCTCCATCC
TACGTGCATTGCCTATTGATGTCCTTGCCAATGCATACCGCATTTCTAGGCAGGAAGCCCGGAACCTAAAAAAT
AACCGTGGTGAAGAGTACGGTGCATTCACCCCTAAGTTTGCGCAAACAGGCTCCCAAAGTTACCAGGACGAGG
GGTCTTCGACTGAGACGACATCCAAGTGAATAAGTGAGTGTAATGGAAACTAGTATAGTGAAATAAAGGCAC
CGCATGTTCGCAGCCTAGTGGTATAGAACCGCTTATCTCAATAAAAAAAGTTTCGTCATGTTATATTGCTTGCTT
GTTTCTTATACTCTTCTTAATTTTATGTTTTCTGAACAACCCCTTACCTCTCTCCTCGATTTTCTGCTATTGTGCGC
CTGAGTGGTCATGCATATCAAGGAGACATATAGGCCACTTGCACTAGATTTTTTTCCGGTAC

>chr1A:522824316-522826744

CCAATCCACCTTCTACAATCTCTTCAGACAATCATGGCAACTACTAGGTTTCCATCGGTGTTGTTTTACTTTTGTA
TTTTTCTCTTGTGCAATGGATCCATGGCTCAGCTCTTCGGACAGAGCTTTACTCCATGGCAAAGCTCTCGACAAG
GAGGTTTAAGGGGGTGCAGATTTGATAGGCTACAAGCATTTGAACCACTTCGACAAGTGAGGTCGCAAGCAG
GTGTCACTGAGTACTTCGATGAGCAGAATGAGCAATTTCGTTGTACCGGTGTATCTGTCATCCGTCGTGTTATC
GAGCCTCAAGGCCTCTTGTTACCTCAATATCACAATGCTCCTGGCTTGGTGTACATCCTTCAAGGTTAGTGTCTA
ATTAAATATATAAATTGCCTTTGTTACACTGCAATAGGATTTAGATGTGCCAAATGTTACCCCATTCATTTTTTTA
ACATTGAAACAAATGTGTTTTCTTTAAGGTAGGGGATTCACATGGTTGACTTTCCCTGGATGCCCGGCGACCTT
CCAACAACAGTTCCAACCATTTGATCAATCCCAGTTTGCTCAGGGCCAAAGCCAAAATCTTAAGGATGAGCACC
AAAGAGTTCACCGCATCAAACAAGGAGATGTTGTTGCGCTGCCAGCTGGCATAGTACACTGGTGTTACAACGA
TGGTGATGCACCGATTGTAGCTGTATATGTCTTCGATGTAAACAACAATGCTAATCAGCTTGAACCAAGGCAAA
AGGTAACTATACAACTTAATCCACACAAAAGATATATAATTATTTGCAAGTGATCTATTTACGATTTAAAGTATA
CATTAGTGGGATATTAATGAACTCTATTTTACTTCATCAATATAAATTTTCAGGAGTTCTTGTTGGCTGGTAACA
ACAAGAGAGAGCAACAGTTTGGACAAAACATATTCAGTGGATTCAGTGTCCAACTTCTTAGTGAGGCCCTTGG
TATAAGTCAACAAGCAGCACAAAGGATCCAGAGTCAAAATGACCAAAGAGGTGAGATAATTCGTGTGAGTCA
AGGCCTTCAATTCTTGAAACCCATTGTGTCACAACAAGGACCGGAGCAAGCCTACCAACCAATTCAAAGTCAA
GAAAGACAATCAACCCAATACCAGGTAGGGCAATCAACCCAATATCAAGAAGGACAATCAACTCAATACCAGG
AATGACAATCATATGACCGAAGTTTCAATGGTTTGGAGGAGAATTTCTGTTCGGTGGAGGCAAGGCAAAACAT

CGAAAACCCGAAACATGCTGACACGTATAACCCACGTGCTGGCAGGATAACACGTCTTAATAGCAAGAATTTC
CCCATCCTTAACCTCGTGCAAATGAGTGCTACAAGAGTAAATTTATACCAGGTATATATAATGCTACATTCAACA
CACTATCTTATTTTTGGATATTCTAATCTCATACAACTGGTTAATAATATGGCATACAAATAATTGCTATTGCAG
AATGCTGTTCTTTCACCATACTGGAATATTAATGCGCACAGTGTCATCCACATGATCCAAGGACGTGCTCGTGT
TCAAGTTGTCAACAACCATGGTCAGACCGTATTCAATGACATTCTTCGTCAAGGACAACTGTTAATCATACCAC
AACACTATGTTGTTCTCAAGAAGGCGGAGCGTGAAGGATGCCAATATATTTCATTCAAGACCAACCCCAATTCC
ATGGTTAGCCACATTGCAGGAAAAACCTCCATCCTACGTGCATTGCCTGTTGATGTCCTTGCCAATGCATACCG
CATTTCTAGGCAGGAAGCCCGGAACCTAAAAAATAACCGTGGTGAAGAGTACGGCGCATTCACCCCTAAGTTT
GCGCAAACGGGCTCCCAAAGTTACCAGGACGAGGGGTCTTCAACTGAGACGGCATCCGAGTGAATAAGTGAG
TGTAATGGAAACTAGTATAGTGAAATAAAGGCACCGCATGTTCGCAGCCTAGTGGTATAGAACCGCTTATCTC
AATAAAAAAAGTTTTGTCATGTTATATTGCTTGCTTGTTTCTTATACTCTTCTTAATTTTATGTTTTCTGAACAACC
CCTTACCTCTCTCCTCGATTTTCTGCTATTGTGCGCCTGAGTGGTCATGCATATCAAGGAGACATATAGGCCACT
TGCACTAGATTTTTTTCGGGTACTATCTACAAGCGCTTCGAGAGTATTTTTTGGGTCCTACAAAAACTTTGAATT
TTGCTAATTAATAGTTGTCTGTGAGAGAATTACACGATTGATGTGTCTGTGAGAGCATATGGCCGGGTGAAAC
TGATCTATTTCAATCCTTGGGGCGGCTATAACTTGCCACCTCATGTTTACATGGGAGGAGCAAGGAGGGAGGA
TGAGCTGGAGTTTAGAATGGTGACGCAGCAGGGCGAAGTGAGGCCAGGATAAATGACAGCA

>chr1A:522833088-522835444

ATGGCAACTACTAGGTTTCCATCGGTGTTGTTTTACTTTTGTATTTTTCTCTTGTGCAATGGATCCATGGCTCAGC
TCTTCGGACAGAGCTTTACTCCATGGCAAAGCTCTCGACAAGGAGGTTTAAGGGGGTGCAGATTTGATAGGCT
ACAAGCATTTGAACCACTTCGACAAGTGAGGTCGCAAGCAGGTGTCACTGAGTACTTCGATGAGCAGAATGAG
CAATTTCGTTGTACCGGTGTATCTGTCATCCGTCGTGTTATCGAGCCTCAAGGCCTCTTGTTACCTCAATATCAC
AATGCTCCTGGCTTGGTGTACATCCTTCAAGGTTAGTGTCTAATTAAATATATAAATTGCCTTTGTTACACTGCA
ATAGGATTTAGATGTGCCAAATGTTACCCCATTCATTTTTTTAACATTGAAACAAATGTGTTTTCTTTAAGGTAG
GGGATTCACAGGGTTGACTTTCCCTGGATGCCCGGCGACCTTCCAACAACAGTTCCAACCATTTGATCAATCCC
AGTTTGCTCAGGGCCAAAGCCAAAATCTTAAGGATGAGCACCAAAGAGTTCACCGCATCAAACAAGGAGATGT
TGTTGCGCTGCCAGCTGGCATAGTACACTGGTGTTACAACGATGGTGATGCACCGATTGTAGCTGTATATGTCT
TCGATGAAAACAACAATGCTAATCAGCTTGAACCAAGGCAAAAGGTAACTATACAACTTAATCCACACAAAAG
ATATATAATTATTTGCAAGTGATCTATTTACGATTTAAAGTATACATTAGTGGGATATTAATGAACTCTATTTTA
CTTCATCAATATAAATTTTCAGGAGTTCTTGTTGGCTGGTAACAACAAGAGAGAGCAACAGTTTGGACAAAACA
TATTCAGTGGATTCAGTGTCCAACTTCTTAGTGAGGCCCTTGGTATAAGTCAACAAGCAGCACAAAGGATCCA
GAGTCAAAATGACCAAAGAGGTGAGATAATTCGTGTGAGTCAAGGCCTTCAATTCTTGAAACCCATTGTGTCA
CAACAAGGACCGGAGCAAGCCTACCAACCAATTCAAAGTCAAGAAAGACAATCAACCCAATACCAGGTAGGG
CAATCAACCCAATATCAAGAAGGACAATCAACTCAATACCAGGAAGGACAATCATATGACCGAAGTTTCAATG
GTTTGGAGGAGAATTTCTGTTCAGTGGAGGCAAGGCAAAACATCGAAAACCCGAAACGTGCTGACACGTATA
ACCCACGTGCTGGCAGGATAACACGTCTTAATAGCAAGAATTTCCCCATCCTTAACCTCGTGCAAATGAGTTCT
ACAAGAGTAAATTTATACCAGGTATATATAATGCTACATTCAACACACTATCTTATTTTTGGATATTCTAATCTCA
TACAACTGGTTAATAATATGGCATACAAATAATTGCTATTGCAGAATGCTGTTCTTTCACCATATTGGAATATTA
ATGCGCACAATGTCATCCACATGATCCAAGGACGTGCTCGTGTTCAAGTTGTCAACAACCATGGTCAGACCGTA
TTCAATGACATTCTTCGTCAAGGACAATTGTTAATCATACCACAACACTATGTTGTTCTCAAGAAGGCGGAGCG
TGAAGGATGCCAATATATTTTATTCAAGACCAACCCCAATTCCATGGGTAGCCACATTGCAGGAAAAACCTCCA
TTCTACGTGCATTGCCTGTTGATGTCCTTGCCAATGCATACCGCATTTCTAGGCAGGAAGCTCGGAACCTAAAA
AATAACCGTGGTGAAGAGTACGGCGCATTCACCCCTAAGTTTGCGCAAACGGGCTCCCAAAGTTACCAGGACG
AGGGGTCTTCAACTGAGACGGCATCCGAGTGAATAAGTGAGTGTAATGGAAACTAGTATAGTGAAATAAAGG
CACCGCATGTTCGCAGCCTAGTGGTATAGAACCGCTTATCTCAATAAAAAAAGTTTTGTCATATTATATTGCTTG
CTTGTTTCTTATACTCTTCTTAATTTTATGTTTTCTGAACAACCCCTTACCTCTCTCCTCGATTTTCTGCTATTGTGC
GCCTGAGTGGTCATGCATATCAAGGAGACATATAGGCCACTTGCACTAGATTTTTTTCGGGTACTATCTACAAG
CGCTTCGAGAGTATTTTTTGGGTCCTACAAAAACTTTGAATTTTGCTAATTAATAGTTGTCTGTGAGAGAATTAC

ACGTTTGATGTGTCTGTGAGAGCATATGGCCGGGTGAAACTGATCGATTTCAATCCTTGGGGCGGCTATAACT
TGCCACCTCATGTTTACATGGGAGGAGCAAGGAGGGAGGATGAGCTGGAGTTTAGAATGGTG

>chr1A:526391345-526393345

ACAACAACATCTCCTTGTTTCAAGTGGTGAACTTCTTTGGTGCTCATCCTTAATATTTTGGCTTTGGCTTTGACCC
TAAGCAAACTGGTCTTGATCAAATGGTTGGAACTGTTGTTGGAAGATCGCTGGGCATCCAGGGAAAGTCAACC
CTGTGAATCCCCTACCTAAAAGAAAACACATTCATTTTAATGTTAAAAACATATGAGCAGGGTAACATTTGGCA
CATCTAAACTCCTAAGTGTAGTATGACAAATGCAATTGCTATATTCAATTAGACACCAACCTTGAAGGATGTAC
ACCAAGCCAGGAGTGTTGTGGTATTGAGGTAACAAGAGGCCTTGAGGCTCGATAACACGACTGACGGATACA
CCAGTACAACGAAATTGCTCATTCTGCTCATCAAAGTACTCAGTGACACCCGCTTGTGACCTCACTTGTCGAAG
TGGTTCAAATGCTTGTAGCCTATCAAATCTGCACCCCTTAAACCTCCTTGCCGAGAAATTTGCCATGGAGTAAA
GCTCTGTCGGAATAGTTGGGCCATGGATCCATTGCACAAGAGAAAGATGTAAGAGTAAACAATATTGATGGA
AAACTAGTAGTTGCCATGATTGTTTGAAGAGATTGTAGAAGGTGGATTGGTGCTAGTTTCCTAATTGTAAGTTT
GATTTTATAGCTATAAAAAGTAGTCCAGGATGCACATGGTGACATAATTAGAGGAGGTAAGAAGATATCCATG
GTGACTCAGCCGATGTTATTTAAAATGTGAATAGATATAACTTTGCCTCAAAAGATATGTGTTGTTTTCCGTTAC
AACTGGTGCTGATTCACAATGCTAGCACCATCTTTTGTATATAGCTCTTGTCCGACGTCTAATCAAAGACATGAT
ATTAGTTGTACTTCCTACAACTATTTTTGGCTTTTGTGACTCCGTTTTATGGCTTGCTTGCGTTTTGGTCTATGCA
CAAGAACATACGTGGATGCTTCTGCCCTTGTGGCCAAAGTTTCTATATCTTTAGTAAATCTATACAACATAGGG
TGTAAAGTGGATGAGTCATGTCCACGTCGTCACAAGTTGATAAGAAACTTACGATGATGTATAATCCACGTTA
GCAGATGAATGTGCAGGGTCGGTCTTGAGATTTGGAGGGCCCGGAGCGAAACTTGAACATGGCCCCCTTTAT
ACAAATATCGTAATTACTGCTAATATAGTTGTATATAAAATATTATGAAATTTAAAGCGCATCCAAATATACCAT
TGCTATCGCACTTATGAACGTATTTCAATCTCATATCTTACCGATTATAATATCGAGTCTGACCGAAATTCTGAA
ATCTTTAGTGCAGGAAACTCCAGGGGCCGGGGCTCCCTATAGCCTGACTTTTGAAAATTTCAAAAAATCTGAAA
GAAAATATACATGCGTAGATTTGATGTCTACCATGTTCACATAAGTTTTCATCAATTTTGTTTTTCATTTTTGACC
TACACAAAAAAGATAAATATGAATACCAAAATCAATTTTGTTTTAAAATACATTATATTTCACAACAAAATTCTA
CACTTGAGTAGAAATTCAGTATTTATATATGTGTAAATGATCAGCTGACGACCCTCGTTCCTAAAAATTATGTTG
TACACCTAGGAGTGTTTAACCTAGGATGGAACAGAAGTTAAGCTGCTATATCCGAGCGTCATGGTCAACTTAC
CGGCTTCATCCTACTAAATACCTTTTTCATGTATAGATATGAGATATCTAATATCTAAGGGATTAAAAAGCCCAA
GTTAAAGGAAAAAAAACCACGTCCCACCCATTGGGAACCGGTGTACCTGAGACATTCTCTTCGCCTTTATGAAC
CGCACAAGAAAGAAACCGACGTCTGCTCTCTAATGGCGGCACAAAAGCTCCACTCTGGCCGCATGTTGGTACC
TAGCTGGGCCCCTCTTGGCCTCGGGGGCCCGGGGTGTGAATGTGACTATGTGAGTCTGACGATCAGTAGGTAC
T

>chr1A:526401439-526403620

ATGGCAACTACTAGTTTTCCATCAATATTGTTTACTCTTACATCTTTCTCTTGTGCAATGGATCCATGGCCCAACT
ATTCCGACAGAGCTTTACTCCATGGCAAATTTCTCGGCAAGGAGGTTTAAGGGGTGCAGATTTGATAGGCTAC
AAGCATTTGAACCACTTCGACAAGTGAGGTCACAAGCGGGTGTCACTGAGTACTTTGATGAGCAGAATGAGCA
ATTTCGTTGTACTGGTGTATCCGTCATTCGTCGTGTTATCGAGCCTCAAGGCCTCTTGTTACCTCAATACCACAA
CACTCCTGGCTTGGTGTACATCCTTCAAGGTTGGTGTCTAATTGAATATAGCAATTGCATGTGTCATACTACACT
TAGGAGTTTAGATGTGCCAAATGTTACCCTGCTCATATGTTTTTAACATTAAAATGAATGTGTTTTCTTTTAGGT
AGGGGATTCACAGGGTTGACTTTCCCTGGATGCCCAGCGATCTTCCAACAACAGTTCCAACCATTTGATCAAGA
CCAGTTTGCTCAGGGTCAAAGCCAAAGCCAAAATATTAAGGATGAGCACCAAAGAAGTTCACCACTTGAAACA
AGGAGATGTTGTTGTAGGGGTTCCGCGTATAAAAACAATAAATCTTACTCTACGCCCCTTGAACCCCTGAAGCT
GCCAGGACCTATCTATTAGTAATACAGTAATGAGAGGGGCTTTTCACGAACTTATCCTTGAAGACCGAAAAGC
GTTGTCAACGCTCAGGAGAGCGTGGTTAATGTAGACGAGCCGAAGCAGACCTCAAGGTCGTGATTAAGTCCTT
GCGCTCCTTTGACTCCAACGACTTGAGTGCCTCTAGTCACTACTAGAAAAAAGCTAATTCGTGGCGCACCTATT
TTCCCCTTCCGTGGCGCACTAGGGGTGCGCCACGACTTCTACGCTACGGCTCAAAAGTGCCCGTAGTGCACTGC
CGGGTGCGCTACAGGAATCTCGACATAGTAGTGGCGCACAACACAATGCGCTACCACTGTTTTCCCTGTGGTG

CATGACAAGTGCGTTGCTAGTACTGGCTGGTGCGCCACTACAGTTATTCCTGTGGCGCATGACGTAGTGCGCG
ACTAGTAGCTGACCAGGTGCGCCACCAACGTGGTGCGCCACTAACAACGTACATGTGCGCCACGGCTTAGCTT
TTCAGAAAACAAAAAAAAAATACTCAATACAGATGATATTCAATACAGATTATATACAACACAGCTGATATACA
CAGATATACAGTACAGAAATAAATACACAGACAAGATAGAAACAAATACATATAGATAAGTCACATACATAAA
TACACAGTATAAGTTATATTATTTCGATCAAGTTACAAAGTAGCTAGTGATGACATGAATACATGTCTCAAACA
AGTTAGAGCTCGCGGAAGCTCGCCTAGACATCTAAAGTAGGAACATGCATGACAGACAACCAAGGACAATAA
CCATCACCACGACAGAGGTATAGGTCTTCATCCGGTTCCTCGTCCTAACTCCCGCTAGATAGCGCGCGTATCTA
GCTTCCGCCTCCGCCATAGTATTGTACCCTTTGTAGCTATTGCCACTAAAACGGTGCACCTATCTCTGGCAGTCT
TCCCAATCATTATAGACTCCAGGAACCCGTTCCTTGTACACGACGTACCATGTCATCTAAAAGTTGGCAATAATA
ATTAAATGGTGGTGAAAAAACAAGTAAAAGTCGCATATAAAGATATACTTATCAAATAAGGTAACAAAGTAAG
CAAAAAATACCAAGTAGTAGCATGTACATGTCGACACACATAAGTACACGTGGGGATAAAGAACTACAACTAG
ACCAACACATCAAGACTTCTGGCTCGGCTCAGGGAACAGGAGGACGCCTTGTTTCGTCATGAACGCCCTATAG
TCACCCTGCATCTTTAGGCGATCTACTACGTCACGGTTATTCGGGGATTGCCTCCGTAGAAGACCTCCCCTGAA
GTCATGACATCACGGCTGATGATTGCCCCAAACTGTTGCTGGATGCGATACATCTCTTGTCTAATATTTGAATCA
TTGATCCTGGCCTTGACTTCGGCCCACCGTTTGAGATGATCTGGCAGCGTAGAATTCTGTTGCCCTAGTAGGAA
CGCCCTCATGTGATGGAGTGCATAGTAGGCTTCCTTCTGAC

>chr1A:526423614-526426528

AAAGATAAAATTAAGAAGAGTACAAGAAAACAAGCAATCAATATAACACTGTGAAACTTTTTTATTGGGATAC
GAGGTTATATATCACTAAGATGCAAGATGCCTTTATTCTACTATACTAGTTTCCATTACACTCAGCTCATTCATTC
GGATGCCTTCTCAGTTGAAGATGATTCCCTATCGATGTCCTGGTAACTCTGGAAGCCCGTTTGTGTAAACTTAG
GGGTGAATGCACCAAACTCTTCTCCACGGTTAATTTTGAGGTTTGGGGCCTCCTGCCTAGAAAGGTGATATGCA
TTGGCGAGGACATCAATAGGCAATGCACGTAGGATGGAGCTCTTTCCTGCGATGTGGCTAACCATAGAATTTA
GGTTGGTCTTGAATGAAATGTATTGGCATCCTTCACGCTCTGCCTTTTTGAGAACAACATAGTGTTGTGGTATG
ATTAGCAGTTGTCCTTGACGAAGAATGTCATTGAATACAGTTTGACCATGGTTATTGACAACTTAAACTCGAGC
ACGTCCTTGAATCATATGAATGACACTGTGTGCATTAATGTTCCAGTATGGTGAAAGAACGGCATTCTGCAATA
ACAATTATTTGTATGTCATATTATTAACCAGTTGTATGAAGCTTAGAATATTTAAAAATAAGATAGTGTGTTGAA
TCTAGTATCATATATACCTGGTATAAATTTACTCTTGTAGCACTCATTTGCACGAGGTTAAGGATGGGAAAATT
GTTGCTATTGATACATGTTATCCTGCCAGCACGTGGGTTGTACGTGTCGGCACGTTTGGGATTTTCGATGTTTTT
CCTTGCCTCCAATGAACAAAAATTCTCCTCCAAACCATTGAAACTTTGGTCCCATGACTGTCCTGCTTGGTATTG
AGTTGATTATCCTTGATATTGGGTTGATTGCCCTACCTGATATTGGGTTGATTGTCCTTCTTGACTTTGAATTGG
TTGGTAGGCTTGTTGCTCTGCTGGTCCTTGTTGGGAAACAATGGGCTTCAAGAATTGAATGCCTTGACTCACAC
GAATTATCTCACCTCTTTTGTCATTTTGATCCTGGATCCTTTGTGTTGCTTGTTGACTTACACCAAGGGCCTCACT
AAAAAGTTGGACACTGAATCCACTGAATATGTTTTGTCCAAATTGTTGCTCTCTCTTGTTGTTACCAGCCAATAA
GAACTCCTGAAATTTTATATCGATGAAGTAAAATAGAGTTCATTAATATCCCACTAATGTATACTTTAGATCACA
GCTAGATCACTTGTAAACAATTATATATCTTTCGTGTAGATTAAGTTATATAGTTACCTTTTGCCTAGGTTCAAG
CTGATTAGCATTGTTGTTTATGTCGAAGACATACATAGCAACAATTGGCGCATCACCATCGTTGTAGCACTAGT
GTACTATGTCAGCCGGTTGCGCAACAACTATTGGAAATATGCCCTAGAGACAGATAGTAGTAAGGACTATTAT
CATATTTCATATAGTTTATGATTAAGTTTTATATCATGCTATAATTGTATTGAGTGGAAACATCAATACACGTGT
GATATACAAACAACCAGGGGTTCCCAGTGAAACTCTAGTCACTGGTTCATTGGTCAACAGATGATCATGTTTCC
TGATCATGTGGCATTTATGCCAGTTGGAAATGAGGTCATCTCATTCCAAGAATAAGATGATGAACCAATACCCA
ACCTAAACATCGTGACCCGATCATGTCACGAGTTCAAGTTACGATGATTAGAAATGGATCCTCGTAGACTATTC
CTTAGACCGTGAGGCTATATCAATCGTTGTCTCCCGAGAGTGCTTAGATAGCATCAAACGCCATCTGGTAACAC
GGTGGTTATAAAGGTGTGTTCAGGTACTCGTAGGAAACCATAGAGGCAATATACGTCAAGAGAGGGATTTGT
CCGTCCATGTGACGGGAAGATACTGAAGGGCCCACTCGGAGAAGTTATATCCAATAGCTGCGCAGCGTGTGA
TTTGATCATGGGGATATCACTTGTCGGAACGAGTCAAGTAGCATTGTTCAGTAACGAGATTGAACACGACATG
GTGATGTCATTGATCAGATCTCGGGCAATGCAAGTATCGAGTGACAAAGGGAATAAGACACGACTGTCCCAGT
TCAAACGACGAAAGATCTTCGTGGAACGCACAGGGGTCACATGGCCATCCAGGTCCCTCTGTCGATCATTGGT

GCGGGAATGTCCCGACCATGTCTGGAGTGTTCCCGAACCGTGGGGTATCACGCTTAAGGGATTGATGATGCTT
AGAGAGTGTTTATAGGATCGGTGGAATCACCAAAGAAGAGAAGGGAGAAAACCGGAAGGGTTTTGGTAAGT
CCGGAGGATGTCAGGAGTGGAGGTGGAAGGTGTTCCATAGCCACGGATATTGAATAATGTGTGAATGAATGT
TAAATGTTGATTTAATCATTTATTCATAATTATTTAATAAAGAGAAAATCAATTAAAGGTCAAAAAGGCTTAATG
GGCCAATTTCCGGAGGGAGAGGGCCCATTAGGGAAAGGAGAGAGGGAGAAGGGGCAAGGGGGCTGGCCG
GCTGGGCCTAGCCCCCGCGCGGGGCCCACTAGGGGCTGGCCGGCGGCCTCCCCACCCTCTCCTCTCCTTTAGTC
CCACCCCGGGACTTTGAGGGAGGGTGTACCCCCTCCTCCCCCTATATATAGTGGGGTATTTGGCCAAGATAAAT
ACACCAATAATCAATAAAGGTCTCCACAGTTTTGGGAGGACCTCCAATTGATATTTTCTCTCTCCCCCTCTCTCTC
TCGTGACCGTGGTCCCAAGTCTGGACTGCGCGAGCGAGAGACAACTTCATCGCGGAGCGCCGTCATGCTGCG
GGATTCGGATCCGAGAGCTCTACTTCCGCAACTGCT

>chr1C:22436141-22438429

CACCCTCTACAATCTCTTCTAACAACATGGCAACTACTAGTTTGTCATCTGTGTTGTTTTGCTTTTGCATTTCTCTC
TTGTGCCATGGATCCATAGCTCAACTATCTGGACAGAGCTTTAGTCCTTGGCAAAGCTCTCGACAAGGAGGTTT
AAGGGGATGCAGCTTTGATAGGCTCCAAGCGTTTGAACCACTTCGACAAGTGAGGTCACAAGCGGGCATCAC
CGAGTACTTTGATGAGCAGAATGAGCAATTCCGTTGTACCGGTGTATCTGTCATTCGTCGTGTTATCGAGCCCC
AAGGCCTCCTATTACCTCAATACCACAACACACCTGGCTTGGTGTACATCCTTCAAGGTTGGTGTCTAATTGAAT
ATGAAAATTTCATTGCTATACTACACTTATGATTTTAGATGTGCCAAATGTAAGAACATTCATAGTTTTTTAATAT
TCAAACAATGTGTTTTCTTTTAGGTAGGGGTTACACAGGGTTGACTTTACCTGGATGCCCGGCGACCTTCCAAC
AACAGTTTCAACCATTTGAGGAAGCCCAGTTTGCTCAGGGTCAAAGCCAAAGCCAAAACATTAAGGATGAGCA
CCAAAAAGTCCATCGCTTCAAACAAGGAGACATTGTTGCACTTCCAGCTGGCATTGTACACTGGTGCTACAACG
ATGGTGATGCACCGATTGTAGCTGTCTATGTCTTCGATGTTAATAACAATGCTAACCAGCTTGAACCTAGGCAA
AAGGTAACTATACAAATTAATCGACACAAAAATATATTGTTGTTTACAAGTAACATAACTATGTTTTTTCAAGTA
TATTTTGGTGGGATATTAATGAACTTGGATTTATTTCCTCAATATTAAATCTCAGGAGTTCTTGTTGGCTGGTAA
CAACAAGAGAGAGCAACAGTTTGGACAAAACATATTTAGTGGATTTAGTGTCCAACTTCTTAGTGAGGCCCTT
GGTATCAGTCAACAAGCAGCACAAAGAATGCAGAGTCAAAACGACCAAAGAGGTGAGATAATTCGTGTGAGT
CAAGGCCTTCAATTCTTGAAGCCCATTGTGTCCCAACAAGAACTAGTACAACAATCCTACCAACCAATTCAAAG
TCAAGAAGGACAATCAACCCAATACCAGGTAGGACAATCAACTCAATATCAAGAAGGACAATCAACTCAATAC
CAGACACAATACCAGACAGGACAGTCATGGGACATGAGTTTCAATGGTTTGGAGGAGAACTTTTGTTCATTGG
AGGCAAGGCAAAACATCGATAACCCCAAACGTGCCGACACGTATAACCCACGTGCTGGCAGGATAACGCGTCT
CTATAGCAAGAATTTCCCCATCCTGAACCTCGTGCAAATGAGTGCTACAAGAGTAAATCTATATCAGGTATAGA
CAATACTTCCTTCAACACACTACATTACTGTTAGATTCCCTAATCATCATAACATTCGATTAATAATATGGTATAC
AATTAATTGATATTGCAGAATGCCGTTCTTTCACCATTCTGGAACATTAATGCGCACAGTGTCGTCTACATGATC
CAAGGACATGCCCGGGTGCAAGTCGTCAATAACCATGGTCAGACGGTATTCAATGACATTCTTCGTCGAGGAC
AACTCCTAATCATACCACAACACTATGTTGTTCTCAAGAAGGCAGAACGTGAAGGATGCCAGTACATCTCGTTC
AAGACTAACCCAAACTCCATGGTTAGTCACATCGCGGGAAAGAGCTCCATCCTACGTGCCTTGCCTGTGGACG
TCCTTGCCAATGCATACCGCATTTCAAGACAAGAAGCCCGAAACCTCAAAAACAACCGGGGAGAAGAGTTTGG
TGCATTCACTCCTAAATTTAGCCAAACTGGCTTCCAGAGTTACCAAGACACCAACGTCTAATCATCTTTGAATGA
TAAGGCATCTGAGTGAATTTGTGTGTGTGATGAGGTCCAATATAGGGAAATAAAGGCATCACAAGTGTGTAA
GTTGGTGGTGTGTAACAGCTTATCTAAATAAAAAACCCGTCATGATGTGATATCATTTAATTGTGTGTTGTGTT
ATTTAATATTATCTTCTCCGATTGATCCCCTTCCCTATTTCCATCAGTTTGTAACTGTTTTGTACCTCATGCTCATG
TGTATCGAGGCATATGGGTCACTTGGCCTAGAATTGTTTTCCAGCGCTGTCTTTTGTGGTTCTTGTATTTTTCAT
GTCCTACGTGAAACTTCAATCTTGCTCACTCTTGGTTACTAGAATTTTTCATAAATCCCAGCCACC

>chr1C:23798301-23800301

TGGTGGGTAAGCGGAGGCGGAGACGGATTCACAGGCTGGTCTTTCGTTTGGATTGCACGACACCGATGGTTG
CTGCCTTAGCTTTGTAAAAATTGTAAGTGCAGAAGTAGTGGTCGAGTGTTTCAGAAGTGTCCGAACAGATGGG
TATTGTTGATTAATGCCATATAAGAGAAGTGGTAATTAATGAGATCTTATTCTCATCTGGCCTTTCCTTTTGACTT

ACGTTTGTTTCCATCAATATTAACTTGCTATAATCGGGAAGGTTCCAGTTCATGCATTGTCAATGAGACGAAACT
GGTCATGACTGCAAAACAGTCTTCTAATTGCAGGCCCGAATGGAATTAAGAAACCCATTGCAAAAACAAAACC
TAACATAGTGTTATTGACATTTTTGTAGTTTCTATTAGGCCTTGTTTACTTCTCTCTGTATTCGTGGGGATCGGA
GGGGAAATCTCTGTTTACTATGTCAATAACAAAACATAGTCTGTTTACTATGTCGGGTTTGGCCGGGATAATAC
CTGGTATATCCCCACAAATTCTCTTGTTTTTGTTAAGATTAAAAACTTCACTCCATGGTAGAGTATTTTTGGAGA
AGGGTAAAGGGACCACTTTGTGGGCCTTCACCCAATAGGGATGTATTCCGTTTCGCCCGAAGGGACCACTTTG
TGGGCCTTCACCCAATAGAACAGGTACACGCCTATTTTTAATTTTCTTGTTTCTATTTTTTATGATTCAAAAATGT
TTTGAAAAAAATTCTAAATTGATTCGAAATTTTTGAAACAGTTCAAAATGGAAAATTGCTGATATTTGAATTTTTT
TTAAAAAATGTTCATAATTGGAATTTGTTTGCATTAAAAAAATGTTTAGAGTTTGAAAAATGTTACTGTACTAGA
GTTTTTGAAATGCTTGAATTTGAAAAAATAAAAAATAAAAAGAAACCAAAAAATAGTCGAATGAAATGAGAAA
AGCGCGAAAAATGGAAAACTATAACCCAAGAAAACTAAATCGAAAGGAAACCGGAACATTATGTTTAATCCCT
ACTAATGGGCTGCGGCCCCGTGTGTGCGCCTTTGCTATAACTCCGTGGCGGGAACCACTAACCAAACCGAAAC
GCTCTCGCCTCTCCTCCGTCACGCAAACAGGCGACGACTCCTCCAGATCTCAGTTGTCGAGAGCCTGGTTTCCTT
TCCCTCCGTTTGCCGCTCCGGCGGTAGGAGGAAGTGGAACCTCATTTCTCCGCCTTATAGTTAGGGTTAGGGTA
TGTCTGCTGTGGGGCGTTGTTGGGATGGTGGGAGCGGCGTCTAGGCAATAAATCTGGCTCAACTCTACTTCCA
CACTGGCAACGGTCTTCACACCGGCGCCTCAAAGTTGATAGCAAGTTGTGCTGGTCGTTCCTCCAGAGCGGCG
CCTTGGAGTTGATGGCAAGGTGTGCTGGTCATTTCTTCAGATCGACAGATTGGTCTTCTCATCGTTCTTCAGATC
TGGCGCGATCAGTTTCTCGATGTGTCGCTGACGTCCATTGTTACTCGTGACGATGCTGGTGGCCGGGGCGAGG
TGGTTTCTCTGGAGCGAGGATGTTGTCCGGAGGTAGTGGATCTGTCGTTCTTCTTCGACTTTGTCTATGAGAGG
CAGCGGATATTGGTTCAAGACAGTATGGGGATGTCCCCCGATCGACGTGCCAGAGCTACATGTGCCTGTCTAC
GTGCAAAGACTCGCAAAGCCTCGTTGGCGATGGTGCTTTTTTGGATCTTGCAATTTTGGAGGTTCGGCGTCTAT
TCCTGAGTCTCAGCGGCGCTGGAGTTGGGGCGGCGGCCGCTGGCTTCGATGATTGCGGAAAATTCTAGAGAT
CGTTTTGTATTTGTTAGTCTTTTTTATCTGCATAGTTTTCAGGATAATCAGTTTGTCTTTTAGTGTTTATATATGTT
ATTACTCCATGCAAATTGATTTTTCGAATAATGAAGTATGTGGTTGCTAAAAAAAAAGCACGCCACAAGGAGC
GCA

>chr1C:381073349-381075349

AGTTCATCTTATAATTTTGTAAATTACAGTTTACGCCTTTGTTTGAGTTACAATTTTGACTTTTATATTTGATTAA
GTAAACAAAAAGCAAACTCATACACTTAGTGGCAGTGTACACGTAACACAGTCCTGACCACTCATTCCTGATCC
TTTCTCTGACATATACAGCCACGAAGCACGCATGCACACCGAAGAGTCAGTCCAGTCCCCTTTTGCTTCCATTCT
CAGTCTGATCCCCTCTTGCATTCTCTTCCGTGATGTTGCTGCCACCACCTGTCCTCAATTCCAGTCGATGGAGTA
TCAATCGTTGCAGGCGAGCAAACCACGGTGCTCCCCATGGCCTGCATCGCCAATTCGTGACCACCGACATGTCC
TTTTGCCCCTTCTCGCGTGGTACGCGCACACAGCCTAGCTCGTGAGCCTTGTCTGCCCTGTAGCCGCTGCTTGG
GAAGAGCGCCACGCATCGCCGCATCGGCGGAGCCATGAAGCGCTAGAAGCTGAGGGTACGTCGCCACCGCC
GGTCGGGCGAAGGGGGGCGCATTTCGCTGGCGGCAGTGAAGCTCCGAACGTACCTGGATGCGCCACCGTGCT
GTTCTTGAGGACAGTAGACATGACCATGTCCGCCCTGCGTCTTCTTGAATGCGACCACTCACCTCTCCAGGCAC
CATGGCCACAAGTCCACCCGTTGACGTGCTACCTCCCCTTTTCTGATAAAACCAAAAGGTTTTTTCCTTCACCTCT
ACCTATCTCACTCTCTGATTTTGCATATTTGCAGGAATTTGTGCCGAGTTTCTTCTCTCTTCTTGTGATTTATTGAC
TCTTCTTGTCCAGAAATGACCGAGCTGCTGAGAACTGAGAAAATATCACTCCAACATGATCAAACAGGCAGCA
GCGCAAAGGAATCAATTTAAAGACAGGTAGTTTTTCTTGATTCTTCATAATTTTACTTAACTCGTTAAGAAGTTA
AACATGCCATACATATACAATATTAATTAAAAGACGATGTATACCATCACGATTCTTCGAAATTCTACTCTTTTA
AGCAAATTCTGCATGAGAAAGCTTAAGCTATGACAGGAAGTAAAGTTCATTCCAAGAAATTATCCGTAGACAT
TGTTTCTTGATTAGACATCTAACTTATGCACGAAGCAGAAAATTATATTCGGGTATCTTTGATTCTGCAAAACTA
TATACCCTTCTTTATTCTACAATATATTTTCAGCCTTTTTTCCAAATTTTTATGTTTCTTTCGGCAAAGGTAGCATA
CAAGGTTAGCTAACTTACTGAACTATGTTCTAGACAGCTGATCTGTTGAGCTAATTAACGGAGGCTGGAGCACT
GGTGAGGCAGTGGCCGCAGGGAATGCCCAATCAAAAGCGGAAAAAGAGAAGCCTTTCCGGTAGGTACCGGC
TACTGCTAAAGAAGAAAAACAATCAACAGGCACTAAGAGACATGAGATAAGCTCCACCTGCAGTGGTAAGTC
ATGACAAAGTTCAAGTGTATACCTTCTCATTTGTTAAGTCATGACAAAGTTTAAGTGTATACCTTCTCATTCATTT

ACTTAGATGTGATGATTTTTTTTGGTCCATTATCACCACTCAAGTAGCAGATCGTCATTCTTGATCTTAGCATGTC
CTTTTGTACACTTACATCGCCCAAACCAAGAATACTGCATGTAAAAATTATATTAGAAAAGGTAAAATATCATG
CATTCAACTCGCGCCAACCAGATTATAACATAAGACGAAACATGCCAGATGTTCCAGCTGATGTTCTAGACTTC
TTGTGTAAGCATGCCAGTCACCCCCAGATTCGTTTGTAAACATCTCTTGCCAGGATACTTATAATTTTGTTTTAA
GATGGTACTACTACATTGCCCATGCAGTGAGCAACATACCTGATCCTTCTGTTTGAAAGTTCACATATAATTTCA
ACTTCAGTTAGAAGCCAATTTCTGAAAATATATAGTACTAATTATTTCAAGTATGAGTTAAACATGA

>chr1D:102003046-102005046

CTGGATATGGAAGTGGGAATGGCAACTCTGGATATCCAAATGCGTGGACTGATCCTTCACAAGGCGGAGGAT
TTGGCGGTTCAGTCAATGGAGCTTCTGAAGGCCAATCAAATTATGGCAGTGGTTATGGTGGTATGCAGCCTAG
GGTTGCTCAGTAAGAGAGGCCATTATAACGTTACGAATTGAACCAGGCTGCTTATGTATTACTGTGGTACTATG
GCTTTGGTCCCATCCTCCTGAAGATCGTCCATTTTGCTGGTTAAATCAGTATTTCTCCTGGTTGGAATGCTTGAT
CCTTTCGTGGCTTACTACTAGAACTTCTAGTCAATAAGTAGCTATTTTGTCGAGTAGCTGTATCTTCGTAATGCT
TTGTTTAAGTTTAGCTTATCCTTCGGTGTGCTTTGTCATGAGTGGAGTTTCAGGTTGCTTCATCTCTAGTTTAAA
GCTAGAGTAGCTTCACTTATGGACCAAATTATCTAGTTATATCTCTATTTGACAGAATCATATATTTGTGTTTGA
CTGTAGTATTTATGCTGGATATTCTCTTTCTCGCATGTGGTAAATGTGTAATGTTTATCTTTTATGGTTGTGTTGA
TATGCGGTGCTGTGCTGTGCTTCCTCATCTACTCTAGCTCATCTTGGAGTTAAAAGTGTTGTGTGATTATTCTTT
AATATTGATGGTTTGCAAAGATGCGAAACCTGAAACTATGCACTGATAGAGGTTTGATACTATAGCTGATTCTG
ACAGATGCTCAAGTGTGTAGTATGCTCCTTTATGTAATAACTGGAATGTTACATCCCTCGTTTATATGATGAAGC
TATGAGTATGCTTCTAATATTTGCTATCTAGATGGATAGACAGGCATAGCTGAGGGAACTGATTACTCTTCCAG
CCTTTTGGCCTTAAATTTTGATATTACCTGAGCAGCAATAAGAACTTACTAGCAGCAAGATGGTAGCATCAACT
TCTAAGGTAGTGTAGATGCAGAGAAATCTGTGATGCTTCTTGAATGATGTACAGGTAGAAAACCTAGAGTAAA
GTAGCGAGGCATTTGGCAAGTAAAATAATTGTCCTTCCTCCTTCGTTTCCTTCATGTACAGGTTGCATGGGATG
CTATTCCTTTTAAAAGCATGTCCAAGTGAGAGTGAAAATGCCATCAGAAACCAGCTGAATTGGCTTGATGAGCT
GATGAGAGGTTGCAATTTTGAAGCATTGGCACAAAATGGAAGCTACTAGCTTTTTACGTGCTTCATTTTCTTGA
GGATCTTGTGCTCTGCTACAAGATGGTGGTACGTGGAAACAAAGATTTATTTTCTTGATGTTTTGTTCGGGAAA
TCTTTTCCAACTTCTCTTTGATATGCTCTGCTCCCAAAAAATGTTCACCAATTCACCATCCTAAAGGTCTAGATTA
CCCTCATAATAAATAACAAAGAAGTGGATTTTTTATTGTAATAATTCCACAATTGTACACCATGTATTTATGTAC
CTTTTTCATGCATACCCCCGATCCAAATTAGTTGTCGCAGGTTCAAAGAACCTAGACAGATTTTAGGCTACATTT
TACCTAAATCTGTGACAAGTAATTTGGATCGGAGGGAGTAGTGAAAATTGGGCCACTAAAGGACAAAAAACA
CCATTCTGTGGCCCCAAAGTCATTGCACTATTGAGTTCTGGGGCACAAGATCCATGAGACTAGCCTTGCCCTTT
CACACATGGTTTAACATTATTCATTCCTTTTTGTTGTTCAATACTTGTTTATTTCTTTTTTTTAGAAAACCTCTTATT
TATTTATTTATTGGATTAAGTTCTACCTGCACACTAAGTCATGCCGTCTATTTATACGTTTGATAGATCTGGTTGC
TCAGCTGCTGACCTTTATTTATTGAGAATATTGAGGATAAATATGAAGTAGAAAATTTGTGTATGATTATGTAC
TTGTAGCGGTATTTTAGCCTAGTTACCGGTTAACAGTAATTAACTTAATATGCTGCCTATTTGT

>chr1D:323681368-323683368

AATGGTGACAACACGAATTGCAAATTAGATCAAAATCTCACAAGACAGGAGGTTCAACATAGCATAATGTTTT
GCTGGCAGATAATGGTTTCAAGTCTAGTACGTTCTACATGTAGTAAAAAACTGGACACCAGAACAGAAGATTG
CCATCAACTTCATGAGCTTGGAATACTCTATTTCTTCTTAGTCTCTGTTCCAGCAGATCTGTAGATCATAGCAGA
AAATTGGGTTAGAACTGGAAAATAGGTTACTTGGTAAGGGATGTAACAGTCAATACAGATACTTCCTTAACAG
AGGAGCAATCGTCAATTAAAGGAGAGTAATTATGTCAGCATCTTAATAAGATTTAATTGGGTTGTGATGCAGG
AGTTTCATTATAGGACGGTTAGGTTATTTATTAACTAGAAAAGAACAAATATTTACAGCAGCATCTGCAGTACA
CTCTGAACAACTCTAACATATTTCAAGCTTCACATACCTCAGTTTCAGGTTGGCTAGTGAGACCAACAAGTTAAC
TAACATATAAAGTACAATTACAGCGAGGATATTGAAGCTAAATATGTAGCACAAATCAAAGTTAACATAATTTT
GTATGAAATATCAGCTATCGAACAATGCAGCATTATAACAACTTGTCAACACAAAAAATGCAGTCGATGCTAAT
GTAACCAAATTTCAGAGTGACTGACCTCATCTTCCTGAGGACACTAGACATCTCCTCTCTCTTCTTCTTGGCCCTC
TTGTGGGTTCCGAGCTTTCGTTTGGCCACCTTCAGTGCACGCTTGTCCTTGCCAACCTTGAGAAGCTCAGTGAT

ACGCTTCTCATAAGGAGCAAATCCAGCAACCTCCCTGATCAGGTTCCTGACGAAATGCACCCTCTTGGTACCTT
TCTGCAAATCAACAAGGGTAACTGGTCAGGCAAAAACATGACAAACTGAAAGATGATGATTGAAATCACCAG
GATACTACTGGAATTATCGTCTACAAAGTGTTTAGTATGCATACAGAAAAGAACATTTAATGGTAACAGAAAA
AACATGACAAACTGAAACTTATCAAAGTACAACTAAGAATCTTCAGAAAATGGATTTCAGGTTGCATATAAACG
AGAGTCCTCCTAAGAGCCTATAACAGATACCCAACAGCAAATCGTATTCCATTTTCAAATGCAGCGTTGAGCCT
ATGGGGATGTCATATAACTTCAATCCACCACCTTAGCAAACTAAGGTCACCATCCGCAATAATAACGCAACCAA
CTAGCCAACTAGCTTTCCTTAATCACTTAGAACTAACAGAAATTTAAATCTCGTCCGAGAGATTGAATAGTTCGT
TGGGTAGAAATGGCAGCAACATCTTAGATCCATCAATAATAGCAACAAGTCTTGATCTAACGAAGACGTCTGC
TGCTTCACATACGAAACTAGACAAGAAAGCAAGCTACCACCGCATAATCTACAAACAAGCAACCGAGCCTACC
ATACTAAATCCACGCAGATCAGCAAAATAGCAAGGTATCTCCGGGTGTGTGTGGGCAGAAGATGCTTACCCCC
TTGCGGTCGGACGGGCGCGGCGGCAGCTCGCGCTTGGTGACGACGTGGCCCTTGTTGATGCCCACGAAGAGG
CCGGACTTCGGCTGCGTCGGCGCCATGGGGGAACCTTACCTGCTCGAACAAGCAGGAGAAACCTACGTTAGC
GACGGGGAGAGGAGAGGCGAGGCACGGAGAGGGCGAGGGTTTTTGGATCCGCGGCGGCGGCGGCGG
CGCATGGGGAGGGGCTTACCTTTACCTGGGAGAAGTGAAGCGGCTGCGTCTAAGGAGAAACCCTAGAGCGAC
GGGGCTGTTTATATAGGAGGTGCCGATGCATCTTCGCGGCCGGTGGACATGGGCCGGGCTAATGTCATATGG
GCTTTATTATTGGGTCTGCCATTCTATTCTATTCTCTTCACTTTTCAGGGCAGATGACTCGGAAATTATCAGATG
GGGTTTTGTTTCCTTTTGG

>chr1D:336686897-336688897

GTGAAATATTTATGTCTTTATTATCGTTATTTGCGCATGAACGAACATGATTACACATACACCACACTTATTATC
AGGGCTAATTCCATGGATCACTTCAGCTAGGTAGCTAGTCTAATACTGGCTGCCGGAGAAGACACTACAATCC
ATGGGCTCGATCTGGCACACGGTGGGTAGCCCCGCCACATACTGCCGCACCTGCGTCAGCCTCACGTGACCGA
CCTTCTGCATCGGCTGTGCCCCCTCGCCGTAGAACTCCGGTCCTCCCTGACGCCTCGACGTCTCGCCGTAGTAG
ATTCCTCCCTGCGGCTGGCTTTCAGCTGTCTCACCGCCGTAGTACTCCTGTCCGGCCTCCTGCTGCCGCGGTGG
CATCTCGTGGCGGAGCACCTGCCCTTGCTGACCACGCCCTTCTTCGCCGTGGTACCCTCCTCCCTGCTGACGGT
GTCCCGCTGTCTCGCCACCGCAGTATCCCTGCTCTTGCTGCTCCCCCGGCGAGCCGCGGCGCCCTCCTCCCAGC
GGCGGCATGGTCTCCTCGTAGTCCCTCACCATGCCGCGGATGGCGGAGCAGCGGCACCCGCGGCTGACGCTCT
GGAGCTGCCGGCAGCAGCGCTCCCTGGTGTCCCACTCCGTGTGGAACAGCGGGACGCTGACGGCATCCTCGC
GCCCGGCGCCGGTCTGCTTTTGCTGGAGGATCTGCCGGCACGCGTGGAGCGGCTTCTGCTGGACCTCGCGCCG
GCACATCACGTCCCTGTAGCTCTGCTCGGTCAGGTCCTGGGCCACGGCGACGGCCATCAGGGCCGCAAAGAAC
ACCGCGAGGAAGACGAACTTGCCCATATTTCTCTAGCTAATAGCTTGCTCTTCTTGTTTCTGGTGTACTGAATAC
CTGGTGGTGGCGAGAGATGTGGAGGAGACGGTCAAATTTTATAGGAACATATACGGTGATGATTCAGCACCA
TGTATATGGTTGTGTGCACGGTGATCAAGCTAGTTGGGTTGAAGAAGTTTTAGTTGAAGTGCAATGCTTGGAA
GAGTGAAAAGGCCGGTACAAATTAAGGGTTTGGGACTTCACGTTAGCCACTTAATTTGGGTATGGTATAGCTT
TACATCATCTCTCAGATATATAATGGCAGATGTATTTGCTGTGATGGAAGCATATGATGCTTTTTTTGGAAGAA
AAAAGTATATGATGTACTATTAGATTGAGTATATACTCTTTCCGTCCATAAATAAGAGTACTTCTAGCTTTTGGG
TGAGTCAAACTTTATTAAATTTGACCAACTTTATATAAATAAATATAGCAACATATATTATATCAAATTGATATAT
TATAAAAATAAATTTGAAAACATATTTACTAGTACCAATTTAGTATAATAAATATTGCTACGTCTTTCTAAAAAG
TTAGTTAAACTTTACTAACTTTGACTTTAGACAAAGCTAGAAGTACACTTATTTGCGAATGGAGAGAGTATGTG
GCATGCTTGCGTGTGCCGGCAAGTTTCATACAAGACGTACATATCAACAGAATGCTTTCTTGAAAGTATTTGAG
ATTGTTGTAATAGACAAGAAAGTGAGATTTGGAGCTTTGCTAACATATCAACAAAATGCTTTCGTGGAACTATT
TGAGTTGGAAAGTTGCTTTTAATACTGTCAACACTATCATGCACTGATGGATGAACTCGATCTGCACAGAACAT
GTTTCATTGGATGTACTCGATATGCACGGAACATGTTTCACACAAGCAACGGTCATGGTGCTTCTATTTTATAG
GGATTCACAACATTTATGAGATTTCTCATATCTATGATCTTGTTGTGCTTTCCGCATCATCTAATCACACAAACCC
ATTGTGAGCTTTGTTTGAACATTAAAAATTTCCAATATCTAGTCTTTTGTGCATAGGAATAATCTCTTAAATAGG
GACTCCAATGTTGGCGATATGGTAAATAATCCAAAAGTAGTGGATCTCGCCTCTTTTTTTTTCTATAAAATAGTG
TCC

>chr1D:355573492-355575492

CAGACTGAAGCCTGATTTGCCGTTGGTCAATTTGAAAAGGAGAGAGCCTTTGGATTATTGTAGTTAAGTCGAG
ACTCGTGAAATGCTGTTTAGCCATGCGGTTGTCTACAATGTTGTATCGATTTGTAGTGCAGTCAGAGACTTCTG
AAATTCTATTTAGCCATGCGGTTTATCTACCATGTTGTCATGATGTTATCTGGCTACTCTACCGTGTGATTTGAG
AAAAGAGCATTTGGAATTCCCATCCGCTATTAGTTGCAGTTTGCAAAAGCTTTTGCTATGGTATGTACCATGTA
GGGTAGAAGCCTTAACCTGAACAGGAGCTGCTGCTAACTTGTGATTATCCGTGTGCAGCAGGTTCTGCCATCT
GCAGTGTTCCTTGAAATGGTTTCAGCTGGGCAGACTTCTGAAATCATGCCCTTTTCCGAGGGCAGTTCTTGGCT
TGGGACTTGATGGCCAAAGAGCCGAATCGATGTACCGTGTGCAGACTTGATATTGTTTGAAGTTGAACTCTGC
AGTAATAGTTCCCCTAATTTCTGTAATGATTTCAGCTCGGCAGACTTCTGAATCTTGTTTTTGATATGGCATCAC
ACTTAGCAGCTGTATACCATTTTGACGGCTCATGGTTTCCATTATTCCTCTGTCGCATGTTCAGACTTAAGTAGT
GCCACATTGTTCAGTGTGTAAATATTGCACTTGGTACCACATTGTTCACTTTGTTTGACTGCATGAACAATAAAT
GTACATCTGTTACACCCTTAGAATGAAAGAAAGAAGAATCAGTCAGTTACAACCATACCAGGTTGAGAAAAGT
TGGGAGCTAAGCTCCAATATACACCTATTACAGACCGGTGCCCATTATTGAGTTGCTGAGCTGATAATACCTTC
AGAGCCAGCTCTCTATGCTGAGCCCAAGAGGTGCAGGATTTCAGTATCTCAAATTGTCAGGCATTACTGAGGA
GAGAACACCAGCATTCCAGCAACCAGTTTCAGGAGAAAAGACAGTAAATTGTTGCGAGTAACAGCAGATTATT
ATATAGGTGGTGGATATGTACAGATTTTCCAGGATCTTGGCGAAAATTACCCACTTATGGGTGCGTTTTAGATC
CTCTGGCCGAAAAGAATCAGTAGCACAACCATTTCTCGCTACCTTTGTTCATTTTCCATCTCAATCGATGCAGCG
CTACAGATTTGGGAGGGAAGACCTCAAAGTCTTTAGGGTAGTCATCAACTGTGCCGCTGTATATGCCACCATTT
TCTTGCATTAGAGAATCGGCTAAAACTAACACATTACATTCTTGACCTTCATCAGGTCCATCTTGTTGACCTTTTC
TGGGTTCTGGATCTACATATTCTGCAGGTGGTTCAGCGGGCATGTCAGCCACTAAGCCAATGATGCAAGTTGC
AGCAAAATGGAGAGTTTTAACTGAGTTGGTGATAGTTTGTAGTTTCTCTTTTTGTATACCTTGCGCTTTTGAACC
CGTCTTTGCAACCAGAGGAACATTTGGTAAAGGAGAGTGTTGCAACGTACCACCAATTTTAATGATGGTTCCCT
CTTCTGCTAATGAACCACAAAGGAAATACGGTACACGGTTCTTCCCAGGTTCCTTGTCCCAGAATCAGGGCGAC
GGGAGCCTTGTGGTAGTTGTGATCTATGTTGTTAAGTCTTATGCACTGTGTTATTGGTTTGGATCTTATGCTACG
TTTGAATATGAATTTGAAAAGTTGAAAGCAATTAATTTTAAAAGTGTGCATCAGAAGTATTAAGGTTTGAATTA
GTATTTTGGGCAAATGTAGTTTATATATGCAAAAAAAACACCAACATGAATTTTATTAGGAGTTAAATTTAGAG
GAAAGACTAGGAACAATGCATCCCTTCGAGGCTTGATTTAATCAAAAGGAGATATAGTTATTTTGTACTCCCTC
CGATCCATATGAACCGTCGCTGATTTAGTACAAAATTGCACTAAATCAGCGACACTTATTATTATAGACCTGGG

>chr1D:357627452-357630495

CATCATGTTTATCCTGGACTACTTTTTATGGCTATAAAATCAAACTTACAATAAGGAAACTAGCACCAATCCACC
TTCTACAATCTCTTCAAACAATCATGGCAACTACTAGGTTTCCATCATTGTTGTTTTACTCCTATATTTTTCTCTTG
TGCAATGGGTCAATGGCTCAGCTATTCGGGCAGAGCTTTACCCCATGGCAAAGCTCTCGACAAGGAGGTTTAA
GGGGGTGCAGATTTGATAGGCTACAAGCATTTGAACCACTTCGACAAGTGAGGTCACAAGCGGGTATCACTG
AGTACTTGGATGAGCAGAATGAGCAATTTCGTTGTGCAGGTGTATCCGTCATCCGTCGTGTTATTGAGCCTCAA
GGCCTCTTGCTACCTCAATACCACAACGCTCCTGGCTTGGTGTACATCCTTCAAGGTTAGTGTCTAATTGATTAT
AAAAATTGCCTTTGTTATACTTCATTTAGGATTTAGATGTGCCAAATGTTACACCGTTCATATTTTTTAACAATGA
AACAAATGTGTTTTCTTTTAGGTAGGGGATTCACAGGGTTGACTTTCCCTGGATGCCCGGCGACCTTCCAACAA
CAGTTCCAACCATTTGATCAAGCCCAGTTTGCTGAAGGTCAAAGCCAAAGCCAAAATCTTAAGGATGAACACC
AAAGAGTTCACCACATCAAACAAGGAGATGTTGTTGCTCTACCGGCTGGCATAGTACACTGGTGCTACAACGA
TGGTGATGCACCGATTGTAGCTGTCTATGTCTTCGACGTAAACAACAACGCTAATCAGCTTGAACCAAGGCAA
AAGGTAACTATACAACTTAATGCACACAAAATATATACAACTATTTACAAGTGATCCAACTATGATTTGAAGTA
TACATTAGTGGGATATTAATGAACTCTGTTTAACTTCATCGATATAAAATTTTAGGAGTTCTTGTTGGCTGGTAA
CAACAAGAGAGAGCAACAGTTTGGACAAAACATATTCAGTGGATTCAGTGTCCAACTTCTTAGTGAGGCCCTT
GGTATAAGTCAGCAAGTAGCACAAAAGATTCAGAGTCAAAATGACCAAAGAGGTGAGATAATTCGTGTGAGT
CAAGGCCTTCAATTCTTGAAGCCTTTTGTTTCCCAACAAGGACCAGTAGAGCATCAAGCCTACCAACCAATTCA
AAGTCAAGAAGAACAATCAACCCAATACCAGGTAGGGCAATCACCACAATATCAAGAAGGACAATCAACTCAA

TACCAGCCAGGACAGTCATGGGACCAAAGTTTCAATGGTTTGGAGGAGAATTTCTGTTCATTGGAGGCAAGGC
AAAACATCGAAAACCCGAAACGTGCCGACACGTACAACCCACGTGCTGGCAGGATAACACATCTCAATAGCAA
GAATTTTCCCACCCTTAACCTGGTGCAAATGAGTGCTACAAGAGTAAATTTATACCAGGTATTTATGATACCAC
ATTCAACACACTATCTTATTTTTAGATATTCTAAGCTTCATACAACCGATTAATAATATGGCATACAAATGATTG
CTATTGCAGAATGCTATTCTTTCACCATACTGGAACATTAATGCTCACAGTGTCATGCACATGATCCAAGGACG
TGCTCGAGTTCAAGTTGTCAATAACCATGGTCAGACCGTATTCAATGACATTCTTCGTCGCGGACAACTGCTAA
TCATACCACAACACTATGTTGTTCTCAAGAAGGCAGAGCGTGAAGGATGCCAGTATATTTCATTCAAGACCAAC
CCCAATTCCATGGTTAGCCAGATCGCAGGAAAGACCTCCATCCTACGTGCATTGCCCGTTGATGTCCTCGCCAA
TGCATACCGCATTTCTAGGCAGGAAGCCCAAAACCTCAAAAATAACCGTGGAGAAGAGTTTGGTGCATTCACC
CCTAAGTTTACACAAACGGGCTCCCAGAGTTACCAGGACGAGGGGGAGTCATCTTCGACTGAGAAGGCATCC
GAGTGAATAAGTGAGTGTAATGGAAACTAGTATAGTGAAATAAAGGCATCGCATGTTTGCAGCCTAGTGGTAT
ATAACCGCTTATCTCAATAAAAAAGTTTCTCCGTGTTATATTGTTTGCTTATTTCTTGTACTCTTCTTAATTTTATC
TTTTATGATCAACCAACTTACCTCTCTTCTTCGATTTTCTGCCACTCTTCACATATGCATATCAAGGAGACGTATA
GTCCACTTGTACTAGATTTATTTTTTGGTACTATCTTTTGTGGTTTGAGTATTTCTTAGGTCCGATAAAAAGATTC
AATTTACTGATGCTAAATTTTTTCAGCAAACCTAGCCACCTAAAATTGTGAATCACATACCTTCAAATTTCTATGC
CAAAGTGTGCGATTTTTCTGTTCTATAGATCATGACATATTTTCTTTTTGGAGAAAGATTATGATATCTTTTCTTT
TTGGATAAAGATTATGACATCATTTACCATGGACATTGTTTAAACAACTCTTCGCAATGTGATAATTAATATTGA
TTATATCCTCAGGCCCAACTAGCGTCAAATATTGGTTTGGTATTTATTGGTATTGGGAGAATCATGTCTATGGCT
GAAGGTGATGGAATAGATCATCTAATAAAATGGAATGAAACCTAAAGCAAACCGGTGCACCGGTAGCATTTAT
TTCGACTCAAAGTCACAATGGCAGAAGTAGCTAAAGCATCCACGGTGCAAAGCTTATAACCATGGAAATGTCT
TCAAATATTCAATCTCTGTGGTGAAATGGTGAATAAGCAATCCAATTACGATGATGATGCCCATGCATGGCGCT
GCCACGCTCTTGTTATTGCAAATTCCTGGTCAATCTATTTCTTCAGAGCTACGGTTTGTTGCTTGAAAAACAAAC
TTGAGATCATTTGTACACATCATATGCATTTACAAGATTAAAAAGGAATCCAAATCTCCACACATCAAGCATAA
ACAGCTACGCTGCAAAACAACCCATGGAGGGACATCACATGGGATAACCTTTTGCGACTACTAGAATTTACTCA
ACCA

>chr1D:361285934-361288200

ATGGCAACTACTAGTTTTTCATCGGTATTGTTTTACTCTTGCATTTTTCTCTTGTGCAATGGATCCATGTCTCAAC
TATTCGGACAGAGCTTTACTCCATGGCAAAGCTCTCGACAAGGAGGTTTAAAAGGGTGCAAATTTGATAGGTT
GCAAGCATTTGAACCGCTTCGACAAGTGAGGTCACAAGCGGGTGTCACTGAGTACTTTGATGAACAGAATGA
GCAATTTCGTTGTACTGGTGTATCCGTCATTCGTCGTGTTATCGAGCCTCAAGGCCTCTTGTTACCTCAATACCA
CAATGCTCCTGGATTGGTGTACATCCTTCAAGGTTGGTGTCTAACTGAATATAGCAATTTCATTTGTCATACTAC
ACTTAGGAGTTTAGATGTGCCAAATATTACCCTGTTCATTTTTTTAACATTGAAAAATATGTGTTTTCTTTTAGGT
AGGGGATACACAGGGTTGACTTTCCCGGGATGCCCAGCAACCTTCCAACAACAGTTCCAACCATTTGATCAATC
CCAGTTTGCTCAGGGTCAAAGCCAAAGACAAAATCTTAAGGATGAGCACCAAAGAGTTCACCGCTTCAAACAA
GGAGATGTTGTTGCGCTGCCAGCTGGCATAGTACACTGGTGCTACAACGATGGTGATGCGCCGATTGTTGCTA
TCTATGTCTTCGATGTAAACAACAATGCTAATCAGCTTGAACCTAGGCAAAAGGTAACTATACAACTTAATCCA
CACAAAATATATTTAATTGTTTACATGTGATCTAGCTATGGTTTAAAGTATACACTAATGGGATATTAATGAACT
CTACTTTATTTCAACGATATAAAATTTTAGGAGTTTTTGTTGGCTGGTAACAACAAGAGAGAGCAACAATTTGG
ACAAAACATATTCAGTGGATTCACTGTCCAACTTCTTAGTGATGCCCTTGGTATAAGTCAACAAGCAGCACAAA
GGATCCGCGGTCAAAATGACCAAAGAGGTGAGATAATTCGTGTGAGTCAAGGCCTTCAATTCTTGAAGCCCAT
TGTTTATCAACAAGGACCAGCAGAGCAACAATCCTACCAACCAATTCAAAGTCAAGAAAGACAATCAACCCAA
TACCAGGTAGGGCAATCAACCCAACATCAAGAAGGACAATCAATTCAATACCAGGCAGGACAGTCATGGGAC
CAAAGTTTCAATGGTTTGGAGGAGAATTTTTGTTCATTGGAGGCAAGGCAAAACATCGAAAATCCCAGACATG
CTGACACGTACAACCCCCGTGCCGGCAGGATAACACGTCTCAATAGCAAGAATTTCCCCATCCTTAACCTCGTG
CAAATGAGTGCTACAAGAGTAAATTTATACCAGGTATATATGATACTAGATTCAACACACTATCTTATTTTTAGA
TAATTCTAAGCTTCATACAACTGGTTAATATGGCATACAATAATTGTTATTGCAGAATGCTGTTCTTTCACCATA
CTGAAACATTAATGCGCACAGTGTCATCCACATGATCCAAGGACGTGCTCGAGTTCAAGTTGTCAATAACCATG

GTCGAACTGTATTCAATGACATTCTTCGCCGAGGACAACTGCTAATCATACCACAACACTATGTTGTTCTCAAG
AAGGCAGAGCGTGAAGGATGCCAATACATTTCATTCAAGACCAACCCAAATTCTATGGTTAGCCCCATCGCAG
GAAAGACCTCCATCCTACGTGCATTGCCTGTTGATGTCCTCGCCAATGCATACCGCATTTCTAGGCAGGAAGCC
CGTAACCTCAAAAATAACCATGGAGGAGAGTTCGGTGCATTCACCCCTAAGTTTACACAAACGGGCTCCCAGA
GTTACCAGGACATCGATAGGGAATCATTTTCAACCGAGAAGGCATCCGAGTGAATGAGCTGAGTATAATGGA
AACTAGTATAGTATAATAAAGGCATCGCATGTTTGCACCCTAGTGGTATATAACCGCTTATCTCAATAAAAAAA
GTTTCGCTGTTGCGTTATATTGATTGCTTGTTTCTTGTACTCTTCTTAATTTTATCTTTTCTGACCAATCCACTTAC
CTCTCTGCCGATTTTCTGCCACCGTGCATCTGAGTGGTCATGCATAATGAGCATACATATAGGCCACTTGGACT
AGCTTTTTTTTATACTCTCTTTTGTGGTTTGAGTAGTTTTCGGGTCCCAAAAAGCCTTCAATTTCCCTAATTACTC
ATGCTAGATTTTTTTTGAAACCCTGGTCGCCTAAAATTTTGAATTA

>chr1D:365065827-365067827

TTTATCAAAGGGATTTATCCCCAATCTCATTAAGAAAACCATAGAGCTACTACATATAGATGGCAAACCAGGTA
AGAGAGAACATTTGCGGGACAACACTTATCCCCGCGCATTATGACCGGCCTAGATGGCACACCAACTACATAG
TACACGGCAAAAACCAATCACATGTTTCCTGTCATGCTGCAAGAAGATGGTTTTCCACGGTAACTGCAGCCTAG
TCCTTGTACTTCATGTCACGCACGCGGAAGCCAGGCATCTCGAACGAGATAGCAAACTTCTCAACCTCTGCCCT
GAGATCCTCGATGTCCTTGTTCTTCTCCAGGTCGACGATGAAGTCCACATAGCGCTTGCCTCGCTTCTTCTGGAC
GTCCAAGCAGATGGCCACGGCCTGGTGGAGGTACTCCGCGATCTGCACAAAGTCCTTCTCGACCAAACCTCTC
GATGTCATTGCTGGTGTACCTGGAACAAGAAATTTATAATGATGAACTTAGCTGGTAGTATTGTTGACATTCAG
AAATCGTCTGTGAATGTATAGGAGTACGAAGAATCCAGATTTCTCCTAGAAAAAACTTTGCTGTTGAGTTCATC
TGTGCAGTATGTTTCTTTCCCTCAGTTGTTCCGAGAGGGATAGTTTAGTGATGCATGTCTGAAACTGACCAGTT
TTATGAAGGTTTAACATGCAAGAAATGCTCTAACTAGGAAGAAGACTTGAGCTCTCTAGTGCTTCTATTTTCTCT
TAGAACGCATTTACTAGTATTCAATGAGATTCTGAAAGATGGTTTGAAATATCAATAATTGATCAAGCAACCAG
TAGGTTATCTTTCATTGCAGCGGAAGTTTTTTGGTACAGAATGTACTACATTATCGAGACTTATGAAGTTACTGT
GCTATCAACTAGACTCACTGTTTTGGTAAATCTTTGTACCAATCTTGTAAATAGGCACATCGTACAGCGCTCTGT
GTACATACCTTCTTGTCACTAACACTATGTTTTTGGGTAAAAAAAAGCGTTGAGACCCCTGGAATGTTAAAACA
ACTTTCTTGTGGACCTTTAGCACTGTTCTGAATATATTTTCACATATCTTTTGCACATTTTAGACTGCAGTGTAAG
AATTACGAGTTTTTGCCTGCGAAATGCAATTTTTTGTTCTTTTTTCAAGCAGTCAGTGTTCCTGGTCCTGGTACTC
CAGGACAAGTTGTTGGCACAGCAAAACGCAGGGTTGATGTGATAATATCGGTAAGTACCTAATCGTTTCCATA
ATTATTTACAACCATTAAAGCATTCTTCCTAACATAATCTTTGCATATGCTGTATATACCTAAGTAAATCTATGGA
TATAAGAGCCAGTAGGATGGACAGTATTGCCAGCCGGATGGACAGTATGTGGGTGAGTGTGTTACTGGGGAG
TCAGTTCTGTCTGCTCCATGTGAACCAAAATGCTACGATGGTCTGTAACGAGCCCTCTTTTTCTAAAAAAATAAG
GTAGGATGCAAAGTGTTGCAAATTAATTTTGGTTCAGTTATCCCAGTATTCAGGTACACAGAAAACAAATAATG
CTAACTAATCAGTATTTTCTTAACCCTGCAGCTTCCTATGAAGCAAGCAGGTCTACAACTTCAGGATAATGTAA
GCTCCTGAGGCTTATATTTGCATGGCTCTTTCTAGTTGCATAAGGTTACAGTAATAATTCAGAGACACAACCAG
TAAGCTGCTGCCGGCTGCCACGTGTAGGCTGGCTAGTTGCTTGGTTGACCTTTTAAGAGACCTAATTAACTAAT
TTGCACCAACACTCCTCCAAGCCACATATGTGCCCTGGAATGTAGGGCCTTGAGACAGATTAGACTTACACAAG
ATCCTTTCATCAAGGAAACAGGAACATCCTAGTACAGAGGGAGAGGAACAACACTCCGCAAGAAATTTTTCAT
TAACAAATCTGCAACACACTAATGTGTTGGCCCACCTCACATGCTCTGGAAGACCAGTGTGAGTGGCAATA

>chr1D:372066412-372068021

AAACAAACAAACAACATCCAAATCCATTCAAACATTCACCACACTTTGTAACTTTGGATTTGGTTCTGTTCAGTA
ACATAAACCAAAATTCCAAAACAAAATAATAATTTTATTTTAGGGTCCATTATCAATGAAATAAATAGACCTTGT
TTATTGAATAAAAATAGGATTTGATTTATTTAAAACTCCTTTTGAAATCTAAATATTTCCAAAACCAAATCAGTA
GGTTTTACTTGAGGATTTAAAATATTTGGACAACTTATTTTACACCAAATAAACAAGTCTTGATGATTTAGAAAT
AGTAAAACCTTTATTAATAAAACTTGGATATTAAATCCTTTTACTTTATTTAAACAAACATTTACTAATTTTATCA
AATAGAAATATTTTATTTCAAAGGCTTGGCCAAGTTTAATTTGGCTTACAAAATAAACCTCATTTAAAATCCTTC
CTTTTCTTTTGAAAACTAAATCTTTTTTTATAAAAAGATTTCAATGTAGTAATTCTAAATATTTTTATTTTCCTTTA

ATAAAAGTTTATCTTTTTGATCCCTTTAGGATTTATTTGTTCCTAATAGGTTTAAAGGTTCTCATTGGAATTCAAA
AAGTTTCTTCTCACCTTGAAAATCAACAAAGTTTTATTTTATTCATTTATTTTTCACATTACAAGATTTGGAAAAT
CTTGGGATGTGACAACCAAGGAGGCATGCGGGGTCGCGAAGGGGGCAGTGATTGGCTTGGACCAACTACTG
GGCCCCACATCATGGAATTGTGGACGGGAAAGTATGCCCGGTTGTGCAACAAAGAGTTGATCTCTCATGGGTA
AAGTAACGCACCTCTGCAGAGTGTAAGAATTATAACTTGTTACTCCCTGTTCCGGGAAGGAGCTACGAACACG
ACAGGAAAGGAACTCCATGAAAGTTCTGGTCAACCTGTGAAGACTGATTGTCATAGTTTTTCAGAATAAAATCA
ATCTTTGAAGAAAGATTTACAAAAATTGCACAGCGCCAAGGATTTATAATCCGAGGAGGTAGTTAGAGCATCA
AACTTCCCACATTGCATATAATGAACTTGTTGGGTAACGCTGTTACTCATTCTTTCTTTTGATCCCTTGCTTAGAC
TCTGAGGATTCCGAAGACGTGACAATGTGTCAACACCCGGATTTTTCAATCCTAGATGCCTGTTATGCCATACA
TCGCAATCCCAGGATTACCATTTTTGCGAGACATAAGAGAATTGGATACCATAGTACTTCATCCATTACAAACG
ATACATAAGTTCTTACACAGATAGGGATCTCATGATCCAGTTCTTAATACAACATCAAGCAGCGGAATACGTAA
TAATAACAGTAGCGTAGGCGAAGCTCCATCTTCGGGGTCACAGGCAATGCTGACGATGGGAGATGATCCTAG
TTGTCGTAACCTTCCTGGGGTCCATCCTCCAGGTACTCCTCTTCATAGTCTGGCCATGGGAATAGCCAGGGACA
AAGCCATGAGTACTCTTTAAGTACTCGCAAACTAATACTAACAAGTAAGGGTTTTTTGGATAATATGACACTAG
GCTCTAGGGTTATTTTGCAAAAAGCCAGGTTTATCTCTACTAAGTTC

>chr1D:372082780-372084835

AAACAGCTAGTTAATGCATGAGATTATGGTTACGTACAAACATGAACGGAACAATGGCAAGCAATGTTATCCA
GACACAAACTTGTTTTTTTTTATTTCCAAGATTGGACTAGTACAGGTCTGGGGTCACACATGTTAGTGCATAACC
CTTTATTTTATTTGTTACCGCTACAACCGCCGGTCCATCGGCTAAACACCGATGATCTGTTTATGTAGTACTATA
GCCCTTGTAAGTCTTCTCAGACCACACTGAGCGGGGTGGTGGCTACGGAGCAGTGCGGTGGAACATACACATC
ACACATCGCCGGCAGGGCCTGCAGTGCAAACGCCCTCATCCCCTCGAACTGAGCTTGCTGCTGAGGCTGGAAA
ACCTGTTGCAGCTGAGGCGGGATGAAAACCTGTTGCAGCTGAGGCTGGATCAACTGTTGTTGCAGCTGTTGTT
GCACCTGAGGCTGGAGCAGCTGTTGTTGCAGCTGAGCCTGGAGCAGCTGTTGTTGGAGCTGAGCCTGGAGCA
GCTGTTGTTGGAGCTGAGCCTGGAGCAGCTGTTGTTGCAGCTGAGGCTGGAGCAGCTGTTGTTGCAGCTGAG
GCTGGAGCAGCTGTTGTTGCAGCTGAGGCTGGAGCAGCTGTTGTTGCAGCTTCTGCAAGATGATGGCTTGCAC
GACACTATGGATGGCCGGGCACCGGACCTGCTCGGGGATCTGTGCCAGCTGCTGGCAGCACTGTTGCTTCATC
ACCTGGCAGCTGCTCTGTTGCAGGATCTGCGACCGGAGGAACGGCACCTCTGCCACTGGGCTGCACTGTTGCA
TGAGGAATTGCCTGCATGGGTTCAGCTGTTGCTGCAGAACCTGCAACAATTGTTGTTGTTGCTGCAACAATGGC
TGTTGTTGTAGAAATGGTTGTTGTTGCTCAGGATATGGCTGATATTGTTCGCTAGGGTCAAACTGGGCAGTGG
CCATGGTCGCCGCCATGGCAAGGAGGGTAAGGATGAGGAAGTTCTTCATGGTGGATTTGTGTCCACTACTGTT
TGCTTGGCTCTGATGTGTGTGTTGTAGGTGTGTGAAGGATGGATGATGGAGGAGGATCTTCACAGTGCATGTC
TATTTATACTGCTATGGCGTGGTTTCTTTTCATCTTTGCATTTGCTTTCCTCAAGAGTGTTTGGGTGCTTACCGAA
CTGATCATTTGGTGTCTTGTGTTTGGTGGCACTACTTACAAAATATAATTTTTGGATTGATCATTAAAGTTGCTT
TGCCCTTGCACGTATCATCTTCTGACTCTCAAGAAAGCTTGATAGTTGGTAATATGTCTCATCTTACTCACTTTAC
ATGTCAAACACTGATCTATATTGCATCATTTCTAAAACTAATTTTGTATTTTTCTACAGACTGTTAGGCATAAACC
AGATTATCATGGCTTGCAGAGTTTGTTAACTTGTGCGTGTATAAGATTAAACTAGTAACATGAAAGATTTATCT
AGACTCCATTTCTTTGGCTATGTAGTGTTGTATAATACAATGTTAAGTTGAACTAGTAAACATGACTCATCAAAT
TCGCCTTTTATGTCAAGGATCATAATGGTGTGAAAGTTATTAAACATATCAGATTAGTTAAGCTTTACATGACTT
CGATAGGTCCACTACATGTGAAGATTATATTTGGATTTCATTCATTTGTTGAACTAATTAAGTTTGTATGTATTG
TATGCAAACTGTTCAGATAATATATAGGTTTTATAGCAGATTTAATCAATATGTTCAAGACAATTATTGGTACAG
TGCGTAGGATCATTGCAAATGATAGCATTTATACAACATATAATGGCATATTTGTTTCCTCAAACTGCCAAAGG
ATCTATTGAATAACCATCCATTGCATAACAAACAAATGGTGTTTCCTTTATTGATGCTTTTATCTATCTACCTGGT
TTCAAGCCCCATCAAATCTGCTTCTGCAAAACAAACCAAGAACAGGCGGCGCAACTAATCAGCCAATCACATTT
TGTGATAGGTAGTTTTTCATCTGAGAATGGACATTTGATGCCGAGCTCTCGTTAGT

>chr1D:372085941-372087941

ACTATGAACGGAAAAACGACAATCAGTTTTATGCAGACATGAACTTTCTCTTCTTTTATTTCCAAGTTTGGACTA
GCACAGGTCGGGGGTCACACATGTTAGTGCATGACCCTTTATTTTATTTGTCACCGCTACAAGATCGGTCCATC
GGCTAAACGCCAATGGTATTTATGTAGTACTATAGCTCTTATAGTGTTCTTAGAAGCCACCGAGAGGGGCAGT
GGCAACAGGGCAGTGCGGTGGGACGTATACATCGCACATCGCCGGCAGGGCCTGCAGCGCAAACGCCCTCAT
CCCCTCGATCTGACCTTGCATCTGAGGCTGGAAGATGCCCTGGGTCACCTGTTGCAGCTGAGGCTGGAAGATG
CCCTGTGTCACCTGTTGCATCTGAGGCTGGAAGATGCCCTGTGTCACCTGTTGCATCTGAGGCTGGAAGAACT
GTTGTTGCATCTGAGGCTGGAAGAACTGTTGTTGCTGCATAATGATGGCCTGGACGACGCTATGGATGGCCGG
ACACCGGAGCTGCTCGGGGATCTGCTCCAGCTGCCGGCAGCACTGTTGCCTCATCACCTGGCAGCTGCTCTGTT
GCAGGATCTGCGACCGGAGGAACGGCACCACTGCCACCGGGCTGCACTGTTGCACGAGGAACTGCCTGCATG
GGTTCAACTGTTGCTGCAGAACCTGCAACAATGGTTGTTGCTGCAATAGCATCTGTTGTTGTTGCTGCAACAGC
ATCTGTTGTTGTTGTAGAATTGGCTGTTGTTGCTCAGGATATGGCTGATATTGTTCGCTAGGATCAAACTGGGC
AGTGGCCATGGTCGCCGCCATGGCAAGGAGGGCAAAGATGAGAAAGGTCTTCATGGTGGATTTGTGTTGACT
ACTGCTTTCTCAGTTTTGATGTTTGTGCTCTCGGTGTGTGAAGGATGAATGATGGAGGAGCATCTTCATGGTGT
AGGTCTATTTATATCTGCCATGGCATGCTTTCTTTTCATTTTTGCATTTGCTTTTCCTTAAAAAGTGCTTGTATGCT
TCTCGAACTGATTATTTGGTATGTTGTGTTTGGTGCCACTACCTACAAATGTAATTTTTGGATTGATCATAAAAG
TTTCTTTGCCCTTGCACGTATCATCTTTCTACTCTCAAGAAGGCTTGGTAGTTGGCAATGCATATCATCTTATTCA
CTTTACATGTCAAACACCGATCTATATTGCATCGTTTTCTGAAACTAAGTTTGTATTTTTCTACAGGCTGTTAGGC
ATAAACCATATTGTCGTGCCTCACAAAGTTTGTAAACTTGTGTGTGTATAAGGTAGATTAAACAAGTAACAGGA
AGGATTGTTCTAGACTCCATTTCTTTTTCTGAACAAACATTCTGGTTTTGTATAATACGATGGTATGATGAACTA
GTAAGCATGACTCATCAGATTCGCCTTTCGATTACACTATACCAATTATCAATTCTAATCTTGATTGTTAAAATA
AAACATGCTTTTGAGTCCCTTGTATCTGACTAGTGATTTGCAGTCACTACTCAGCATGAGATGAACTTAATAGG
GTTATTATGCTTAACGACTCTGATGTGATTCACAGTTCTGTTGATTAGTACTGAGCGGCTGAACCTGATGAGCT
TATATATGATTTGTTCTTACTATGCTAGATATCATTAGCTGCTGTATTATTTTGTAATGTTGTTCCCTGAGCTTAA
ATAGCTTGTTTTGTTTTACAAGGAGGCATATCCAACCGATGTCCTTCCATTTGATATCTTAGCTGAGAGAGCTCC
ACAGCAGAAGTTCGAATTGGTTAGTAATCGTAACATTGCGTTCAGTAGAAGTTTGAATTGATTCTTAGTTTTGA
ATTGCTATATCTTTGGTGCTAGCAACTCACTTCTGGATAACCACCTTCGGCGCTTGGACCTTTTCAGACTAAGGT
AATTTCGTTATACACTGTTCTTGGACTGACTTATTGATTGCATCGACGTAGGAAGGCAGAAACATCATTTA

>chr1D:372089422-372091821

AAAACGACAATCAATGTTATGCAGACAAGAACTTTGTCTTTTTTATTTCCAAGTTTGGACTAGTACAAGTCGGG
CGCCACACATGTTAGTGCATGACCCTTTATTTTATTTGTCAACGCTACAACGATCGGTCCATCGGCTGAACTCCG
ATGGTCCATTTATGTACTATAGCCCTTATAGTCTTCTCAGAAGCCACCGAGCGGGACGGTAGCGACGGGGCAG
TGCGGAGGGACGTACACATCGCACATCGCTGGCAGGGCCTGCAGCGCAAACGCCCTCATCCCCTCGAACTGA
GCTTGCTGCTGAGGCTGGTTGAAAACCTGTTGCAGCTGAGGCTGGAAGACCTGTTGTTGCAGCTGAGGCTGG
AAGACCTGTTGTTGCAGTTGTAGCTGAGGCTGGAAGACCTGTTGTTGCAGCTGAGGCTGGATGAACTGTTGTT
GCTGCTGCTGCTGCTGCAAGATGATGGCTTGCACTACGCTGTGGATGGCTGGGCACCGAAGCTGCTCAGGGAT
CTGCGCAAGCTGCCGGCAGCATTGCTGCCTCGTCACCTGGCAGATGGCCTGTCGCAGGATCTGCGACCGGAG
GAACGGCACCGCTGCCACTGGGCTGCACTGTTGCACAAGGAACTGCTTGCACGGGTTCAACTGTTGCTGCAGA
AGTGGCTGGAGAAACATTTGTTGTTGTTGTACAAATGGCTGTTGTTGTTGTACAAATGGTTGTTGTTGTACAAA
TGGCTGTTGTTGTTGTACAAATGGCTGTTGTTGTACAAATGGTTGTTGTTGTTGTACAAATGGTTGTTCTTGTTG
TACAAATGGCGGCTGTTGTTGTACAAATGGTTCTTGTTGCTCGGGATATGGCTGATATTGTTCGCTAGGGTCAT
ACTGGACAGTGGTCGTGGCCGTGGTTGTGGCCACCGCCATGGCAAGGAGGGCAATGATGAGGAAGGTCTTCA
TGGTGGGTTTGTGGTGACTACTGTTTGCTCGCCTTTGACGTTTGTGCTCTAGGTGTGTGAATGATGGATGATGG
AGGCGATCTTCATGGTGCAGGTCTATTTATATCTGCCATGGCATAGTTTCTTTTCATTTTTGCATTTGCTTTTCTC
AAAAAGTGCTTGTATGCTTCTCAAACTGATTATTTGGTATGTTGTGTTGGTGGCACTACCTACAAATGTAATTTT
TGGGTTCATCATAAAAGTTTTCTTTGCCTTGCACGTATCATCTTTCAACTCTCAAGAATTGCTTGATAGTTGGCA
ATATGTATCATCTTATTCACTTTACATGTCAAACACTGATCTATATTGCATCTTTTTCTCAAACTAAGTTTGTATTT
TTCTACTGGCTGGCTGTTAGGCATAAACCAAATTGTCATTGCTAGCAAAATTTGTAAACTTGCGTGTGTATAAG

GTAGATTGAACTAGTAACATAAAGGATTGTTCTAGACTCCATTTCTTTGTCTGAACATCAGTGTAGTTTTGTACA
ATACGATGGTAAGATGAACTAGCAAACATGACTAATCAGATTCTCCTTTTATGTCAAGGATAGTAATGACGTAC
AAGTTATCAATGCATATCATATTGGTAGGGCTTTACATGACTGTCAGTTCAGTACATGTGAAGGTTAGTTTTGC
ATTTCATTGTTTTGTTGAACTAAATAAGTTTGTATGACAATCTCTATGTTTTCTTAGATGATTTCTTCAAAAAAAT
TCGGCGGATCATTGAGCATTTATACAATAAATGATGGTATATTTTGTTTCCTTACACTACAGTGAAACCATTCAT
CTTGTAACAAATAAATTTGGGTTCTTAAATTGACACATTTATCTATCTACCAAGTTGCAAGTCCAGTCAAAATTT
GTTGTTGCCAAAGGAATCAAGAAGAGGTGACAAAACTACTTGGCCAACCATGGTAGGTGGTTTTTGAGAATA
GACATTTGGCTTTCAAGCTCTAGTGACTAGTGAGCTTGTTCAAAACAAGTATATATTTGGATGTACAAAAATAA
ATGGAAAATTATGCGTGAACTATTTCATCCGCAGCATGTGCGGAAAAAACAAGGAAGATAGACAAACATATCA
AATACTGACGCTCCATATTCAATATTTTTATTCAAATCCCACAAAAAAAATAGTATTTGTGCATGAAAGTTACAT
TTCCACTTAATATTTCGTCAATGCATACAATGGTATAATACTTTTTTCATGAAATTTTCCAATGTAAAAAACAGA
AAACAAAGTCTAGTAGCACCGGAGCTCGAACTTCAAAACAAATTTCCGTTTATCATCCCCAAAACGAAAATCAA
TAGTTATGGCACTCTTCAAATCTTTAGTTGTTCCCGATGTACTCTAATGACTTAAGGAGCTATGATCTATGATCC
CAAATAATCCATGGTATATCCAACAACGT

>chr1D:372092060-372094060

CAACCGAAATTCGACACCCATTTTTTTTATGTTCTGATTAATTGTAGCGCACACAGGATTGTGGTTAGGGATGA
ACATCTCACTATGCACATGAACCAAACAATGACAAGCAACGTTATGTAGACAACAACTTTCTTTGTCTGTTATTA
CCAAGTTTGGCTAGGGTCATACATGTTGGTGCATGACCACCTTATTTTTCACCATCGGCTAAACACCGATGGTC
CATCTATCTAGTACTACAACTCTTATCTTCTTAGCAACCACCGATGCCGGCGGCGATACCGCCGACCTGGCCAG
TGGAACAGTACGGTGGGACCTGCACATTGCACATCGCAGGTAGGGTCTGCATCGCTACCACCTTCATCGCCTG
GTACTGAGGTAGCTGTTGAGGTTGGATGAAGACCTGGCTCGCCATTTGTGTCTGAGGCTGAACAAGACCCATT
TGTTGAGCTTGGACGACTTGTTGTTGTTGTTGCTGCTGCATGATGATGGCGTGCGCCATGATGTGGATGG
CCGGGCACCGGAGCTGCTTGGGGACTTGCGCCAGCTGCCGGCAACACTGCTTCCGCATCACGTGGCAGGTGCT
CTGTCGCAGGATCTGCGACCGGAGGAATGGTACCATCTCCACCGGGTTGCACTGTTGCATGAGGAACATCTGG
CACGGGATCATCTGTTGCTGCAGCAATGGCTGCATAAATGGTTGTTGCTGCTGCATAAACGGTTGTTGCTGCTG
CATAAATGGTTGTTGTTGCTGCATAAACGGTTGTTGCTGCTGCATAAATGGTTGTTGCTCGGGATAGGGCTGAT
ATTGTTCGCTAGGGTCATACTGGGCAGTGGCCATGGTTGCTGCCATGGCGAGGACGGCGAGGATGAGGAAG
GTCCTCATGGTGGATTTGTGTTGACTAGTGCTTGCCTGGCTTTGATGGTTGAGCTCTAGGTATGTGAAGGATG
GATGATGGAGGAGGATCATCACGGTGCAGGCCTATTTATAGCTACCATGGAATGGTTTCTTTCCATTTTTTACT
TTTGCTTTCGTTTAAAGTGCTTGGCTGCTTGTCGAACCGATCAGTTGGTGCGTTGTGGTTGGTGGGGACTACTT
ACAAAATGTAATTTTGGATTGATCTTCAAAGTTGTTATGCCCTTTCGTGTCATCCGGATCTCTGGTAAGATTGAT
AATTGGCAATGACTCATGTAACTCATTTTACATGTCAAACACCAATCTAGATTGTGTTATTTTCTTAAACTAGATT
TGTAATGTTCTACATTTTGTTGGGGATAAACAAGATATGCATTACTTGTGTGGAACTATTTTGTAGTCTTGTTTG
TGCCGTTAGCTTAGACCAAACTAGTACATGAAGGGTTGTTCCACACTCCATTTTCTATGTCTTAACTAACTTTGT
AGTGTTGTACACGATGGTAAGATGAACTGCCAGACAAATTATTCACTTTCTAGACTCATCAAATCCCTATTTTAG
GCCAAGGATTGTGATGGTGTATAAGTTATTAATACATATCATATTACGAAGGCTTTACACTACCTTGATTGATTC
ACTCTAACTAAGTACATATGAAAGTACGTTATGAAATCCATTCATCTGTTGAGCTAATTTAGCCCGCACGTATTT
GACTGCAATTGTTCAAATGATCTCTAGGTTTTATTTTGCCGAGTTTATTACAAAAACCTGAAATAATTCACATGT
GTTCATGATTGGATGAAATAGATCATGTCGCTTATGCACAGTCCAATATAACAAAACATTGATGCTTCGAATTT
GGTCTTTTTGTACAACTCACAAAAAATAGTATTTGTACTGGAGAATTGATCATTTATTAGGATTTTTTTTGAAAT
GGAACGCTGACATTCCATTAAATTTGCTCGGCAAGATCGCCGAACATGGCCCTGATTACATCAAAGGGAAAGT
CATCTAGCCAAACATTGTGGTAATCTACCGGCTCAGCCACACCCATTGCCACCACACTCATTGATCATTTAGGCC

>chr1D:377528111-377529111

GCAAAGGTGAGCTGCGTGGAGGCAAGCTCTGAGAAGAAAAACCCAGCCGCGGGGAGCACTGTCACCCATGA
TGATGACAACGAGAAGACGTCGTGGCCTGAACTGGTGGGAAAGTCGGTAGAGGAGGCGAAGAAGGTGATAA
TGAAGGACAAGCCGGAGGTGGAGATTGTGGTGGTGCCAGTGGGGTCAATTATGACCATGGACTATCGAACCG

ACCGTGTCCGCCTCTTTGTCGACACTGTTGCACAGGTCCCCAAGGTGGGATAGAAAACTGTCGATCTCTTTGGG
CGGCATAGATATATGCAGGTCACTCTCTTCTTGGCTTTGTCAAGTTTGAGATACCGCCAGATCAATTAATAAGA
AGCTGATGTCCTGTACATGCAGCTTGCTCTCTCTTCTTGGCTTTGGCCAGATTGAGATAGCTAGATCATATAATA
ATAAGCTGCTCTTTTGTTCAAGGGAAAGTTGGAATTGAGCTGGTGTGTAGGTACACCCCTAACTATGGATAAG
ATTTTGTGATGTAACAAGTACAACAAAACTTCTACAAAATAATCATGAATGTAATAGACCAATATAATATGTTA
AACACAAGATCAAGAGAAAATGACAATCGGTATGTGCCACATAAAAATAACAAGCGACAAAAAAATTATATAT
GAATATAAATTACAAAATTTCTCTTTTTTATATACTAAACAGCACAACAGATCAACTTGAAACTTTCTAGGTCAA
CATACTCCGGTACTACAAACGTAACCTAGACCTAAAAAATCTAGGGATTTTTTCAGAGAACCTGAAATAATATG
GTTTGGCTTTACATGTGACAATGCATCATATTAGTTAGACGTGAATATTCCCTAGAGGGAATTTTTTAATACGA
GTGAGGATTCGGTGCACATGGACACAGTGCTCCCTACATATTTAAAAATTAAAACAATATTTACAAATTTCAGA
AAATTGTAAATCAAAATCCTGAATGTAGCTAATGATGTATCCCACA

>chr1D:378426514-378428514

TTTCTCTTTTTTTTTTTTTTTTTTTTTTTGCGTTGTGCTGTCTGGCCCAACAGTAATCTCACCGGTGGTTGCTATAT
TTATATAGCTGGAGGTATGCCTGTTTCAAGGAGGGTGTGGAAGATTGGATTGTGCACGCAAATTGATGTTTTG
CAATAGAAGTAGTGCACGTTGTCTCATATGGATGTCGTTACCTTGGCGGAGCCACATCATTAATTTAGAACCTG
CAATTTTAATAACACCACTTTGTTATGTCCCTCCATATCTTTGTGCAACTCTTCTTTTTAATATATTAGTAGCGCTA
TTCATGGTTTTGAAGGCTTCGCTTAAGGGTCCCTTAGGCCCCACTCGAAGACGCCCTAACGCCTTTAAAACCAT
GGAGCTTTTCGATCTGTACCATTCGGTGTTTATACAATTAAAATGATGTTGAATATTTGCAAATAAGCTAATAGT
TGAGCTATTGCTAACTGTTTACATTTGTGTGATGTATGGACATATTAAAATCTATGTAAATGAATGATTTTGTTC
CTCATCTGTAAATGAATGTCTTTCTTCCTCGTCTTCGGCAACAAATGTTATTCTTTATGTGATGCACTGATTGAAA
AAAATCATTTTCCTACCTGCAAATGGACATTTTTAATTAAAGTGCACATTGTCTCATGTGTCTCGGCTGCCTCGA
TCAAGCCATACATGGAGACCAATGCCAAATCTAGGCTCATGGAGGCTGAAGCACTACTAGGAAAAACGTTATC
AGTGGCGCACCAAAAATAAATTCTATGGCGTATCTAGTGGTCGCCACTAAAATTAGAATTTTGTGGCGCATATG
AATATGTGTCACAGAATTCTGTTCCAGATTCTATGGCGCATATGGCCATATGCGTCACCGATTTTTTTTCAGAAT
TTTGAAATTTTCAGAATCTGGTATAAATACAGGGTATATTACAGGAAATACACAAAAATTAATAGAAATACATA
GAAAAGCTACACACAGCAGTACAAATATTCTAGTTCACATATAGTACATCAACAAGCACGAATGTTCACAAGTG
TTCATCAATCACATCACTTAGAACCGAATAAGTGTACAAGTACATTTCACTAAGTGTTCATCTCACTTACATATC
TTACATTTCACATCACTTAGAGGCTCGGGAAGGTTCACACATGTCGGATCGACGATTTTGGTTCGATGTGGTTC
GGGTTCGGACTCGGCTGGATCCGTGAAGGAGAACTTGGCGCGGCACCGGGTCTGGTTCGTTCTTCAAGGATCT
ATGTCCATCTTGGAATCAACGCCGAAGATTGGGCTTCAGGGTACCATGGCAAAGATCTTGAGCTTTCCGTGAA
GGAGATCTTGGATCCCATCTTCTGTGTCAATGATGGACTTGGGTCGACGCTCATGACCCGGTTGCACTGTTTCC
GGACATCGAGAGTACCGGCGTCGGGTTGGTCTTGTTGACTGGAGATCTAGGCAACACAGCGAGCATCACCAA
ACCTAAGGGAGTAGGCATTTGCACCAGCATGTCTCAAATCCACACCACATAGGTCAACACCACTAGCACCAGA
GGTAACAACTTGATAAAGCTGGTCACGAGAACAAACGCCTGCACGAAGACATAAACGAACTCGCTGGCAAGG
CCGACGGCGGCAGGGATGGTAGCGTTTGAGGTGGGATCAGAACAGAGAGGAGGACGAGGCCTTGGTGTGC
GCAAGCGACGGCCTCGCAGACGCCAGCGGTAGAGCTTGACGGGTGTAGGGCAGCCTGCACAGCAGAGCACA
TCGTCGGTGCGGGGAGGAACGTGCGGTCGGCCGCGGAGGGGGCGCCGGCGGCTCGAGGACTAGGCCTGCC
ACGACGACGCGATGAGGAGGAGGGAGGTCGCGGAGTCAGAGGAGGTCGCAAAGGCGGTGGAGAAGGTTCA
TGGCCGGAATCCATGGTCGTCACGGGGTTGTAGAGTAGGCGGGGAAGGCGAGCCCGAAGTGGACGAGGTGG
AGGACGAGGCTGTTGG

>chr1D:378637746-378640041

TAGTCGGAGAATATAAAATTAAATAAAGAAGAAGACACAAAGGATATAACATGATGAAGTTATTTATTAAGAT
AAGCGGTTATATACCACCCACTTCCACACTTGCGATGCTTTTATTTCACTATTGATCCTCATCACTCTCACTAATT
TACTTTGATGCCTTATTAGTCAAAGATGACTCATCCTCATCCTCTCGATAAGGTTGGAAGCCCATTTGGGTAAAT
TTTGGAGTGAATACACCGAACTCTTCACCCCTGTTGTTTTTTAGGTTACGAGCTTCCTGCCTAGAAATATGGTAC
GCATTGGCGAGCACATCCACGGGCAAGGCACGAAGGATGGAGCTCTTTCCTGCGATGTGGCTAACCATGGAG

TTTGGGTTGGTCTTGAATGAGATATACTGGCATCCTTCACGCTCCGCCTTCTTGAGAACAACGTAGTGTTGTGG
TACGATTAGCAACTGCCCTTGGCGAAGACGGTCATTGAATACAGTCTGGCCATTGTTATTGACAACTTGAACTC
GAGCATGCCCTTGGATCATGTAGACCACGCTATGCGCATTGATGTTCCAGAATGGTGAAAGAACAGCATTCTG
CAATAGCAATTATTTGTATGCCATATTATTAATTGGATGTTACGAAGCTTGGAATATCTAATAAAAAGATAGTG
CATTGAAGGTAGTATGGTATATACCTGGTATAGATTTACTCTTGTAGCGCTCATTTGCACGAGGTTAAGGATGG
GGAAATTATTGCCATGGAGACGTGTTATCCTGCCAGCACGTGGGTTGTATGTGTCGGCACGTTTGGGGTTTTC
AATGTTCTGCCTTGCCTCCAATGAACAAAAGTTCTCCTCCAAACCATTGAAACTTTTATCCCCTGACTGTCCAAC
CTGGTATTGAGTTGGTTGTGCTTCTTGATATTGGGTTGATTGCCCTACCTGGTATTGGGTTGATTGTCCTTCTTG
ACTTCGAATTGGTTGATAGGCTTGCTGCTCTACTAGTCCTTGTTGGGACACTGTTGGCTTCAAGAACTGAAGGC
CTTGACTCACACGAATTATCTCACCCCTTTGGTCGTTTTGACTCTGGATCCTCTGTGCTGCTTGTTGGCTTATACC
AAGAGCTTCACTAAGAAGTTGGATATTGAATCCACTGAATATGTTTTGTCCAACTTGTTGCCCTATCTTGTTGTT
ACCAGCCAACAAGAACTCCTGAGATTTCATATTGATGAAGTAAAACGAAGTTCCTTAATATCCCACTAATTTATA
CTTTAAAATCCTAGTTAGGTTAATATTGAATTATATATCGCTATGTAGATTAATTTGTATAGTTACCTTCTGTCTA
GGTTCAAGCTGATTCGCATTGTTGTTTACATCGAAGACATAGAGAGCTACAATCGGCGCATCACCATCGTTGTA
GCACCAGTGTACAATGCCAGCCGGCAGCGCAATAACATCTCCTTGTTTAAAACGTTGAACTCTTTGGTGCTCAT
CTTTGAGATGGCTTTGGCTTTGACCCTGAGCTTGATCAAATGGTTGGAACTGTTGTTGGAAGGTTGCTGGGCAT
CCGGGTAAAGTCAATCCCGTGAAACCCCTACCTAAAAGAAGACACAATTGTTTCAATATTAAAAACTTATAAAT
GAGGTAACATTTGGAACATCTGAAGTGTAGTATAGAAATGCAATCACCATATTCAATTATACACGAACCTTGAA
GGACGTATACCAATCCGGGTGCATTGTGGTATTGAGGTAACAAGAGGCCTTGGGGCTCAATAACGCGACGGA
TGACAGATACCCCGGTACAACGGAATTGCTCATTCTTCTCATCAAAGTATTGAGTGACACCTGCTTGTGACCTC
ACTTGTTGTACTGGCTCAAGTGCTTGTAGCCTATCGAACCTGCACCCCCTTAAACCTCCTTGCCGAGAGCTTTGC
CATGGAGTAAAGCTCTGGCCGAATAGCTGAGCCATGGATCCATGGCACAACAGGAAAATGCAGAAGTAAAAC
AACACTGATGGAAAACTAGTAGTTGCCATGATTGGTTGAAAAGGTTGTTGTACAAGGTGGATTGGTGCTAGTT
TCCTAATTGTAAGCTTGGTTTTATAGCTAGAAAAAGTAATTTCAGATGCACATGGTGACGTAATTATTAATAAA
GATAAGAAGATATGAATGGTGACTAGATATTGTTTAAAAAGCAAATAGATATAACTTCTACTCAAAAGTTAGG
TGTTTATTTTCGCTTACAACTAGCTAGTGCTGACTCATATAGCTAATACTACCGTTTGTACATTGCTCTTGGC

>chr1D:378650980-378653141

TAGTCGGAGAATATAAAATTAAATAAAGAAGAAGACACAAAGGATATAACATGATGAAGTTATTTATTAAGAT
AAGCGGTTATATACCACCCACTTCCACACTTGCGATGCTTTTATTTCACTATTGATCCTCATCACTCTCACTAATT
TACTTTGATGCCTTATTAGTCAAAGATGACTCATCCTCATCCTCTGGATAAGGTTGGAAGCCCGTTTGGGTAAA
TTTTGGAGTGAATACACCGAACTCTTCACTCCTGTTGTTTTTGAGGTTACGGGCTTCCTGCCTAGAAATATGGTA
CGCATTGGCGAGCACATCCACGGGCAAGGCACGAAGGATGGAGCTCTTTCCCGCGATGTGGCTAACCATGGA
GTTTGGGTTGGTCTTGAATGAGATATACTGGCATCCTTCACGCTCCGCCTTCTTGAGAACAACGTAGTGTTGTG
GTACAATTAGCAACTGCCCTTGGCAAAGACGGTCATTGAATACAGTCTGGCCATTGTTATTGACAACTTGAACT
CGAGCATGCCCTTGGATCATGTAGACCACGCTATGCGCATTGATGTTCCAGAATGGTGAAAGAACAGCATTCT
GCAATAGCAATTATTTGTATGCCATATTATTAATCGGATGTTATGAAGCTTGGAATATCTAATAATAAGGTAGT
GCATTGAAGGTAGTATGGTATATACCTGGTATAGATTTACTCTTGTAGCGCTCATTTGCACGAGGTTAAGGATA
GGGAAATTATTGCCATGGAGACGTGTTATCCTGCCAGCACGTGGGTTGTATGTGTCGGCACGTTTGGGGTTTT
CAATGTTCTGCCTTGCCTCCAATGAACAAAAGTTCTCCTCCAAACCATTGAAACTTTTATCCCCTGACTGTCCAA
CCCGGTATTGAGTTGGTTGTGCTTCTTGATATTGGGTTGATTGCCCTACCTCGTATTGGGTTGATTGTCCTTCTT
GACTTCGAATTGGTTGATAGGCTTGCTTCTCTACTAGTCCTTGTTGGGACACTGTTGGCTTCAAAAACTGAAGG
CCTTGACTCACACGAATTATCTCACCCCTTTGGTCGTTTTGACTCTGGATCCTCTGTGCTGCTTGTTGGCTTATAC
CAAGAGCCTCACTAAGAAGTTGGATATTGAATCCACTGAATATGTTTTGTCCAACTTGTTGCCCTATCTTGTTGT
TACCAGCCAACAAGAACTCCTGAGATTTCATATTGATGAAGTAAAGCGAAGTTCCTTAATATCCCACTAATTTAT
AATTTAAAATCCTAGTTAGGTTAATATTGAATTATATATCGCTATGTAGATTAATTTGTATAGTTACCTTCTGTCT
AGGTTCAAGCTGATTCGCATTGTTGTTTACATCGAAGACATAGAGAGCTACAATCGGCGCATCACCATCGTTGT
AGCACCAGTGTACAATGCCAGCCGGCAGCGCAATAACATCTCCTTGTTTAAAACGTTGAACTCTTTGGTGCTCA

TCTTTGAGATGGCTTTGGCTTTGACCCTGAGCTTGATCAAATGGTTGGAACTGTTGTTGGAAGGTTGCTGGGCA
TCCAGGTAAAGTCAATCCCGTGAAACCCCTACCTAAAAGAAGACACAATTGTTTCAATATTAAAAACTTAAAAA
TGAGGTAACATTTGGAACATCTGAACTCTGAAGTGTAGTATAGAAATGCAATCACCATATTCAATTATACACGA
ACCTTGAAGGACGTATACCAATCCGGGTGCATTGTGGTATTGAGGTAACAAGAGGCCTTGGGGCTCAATAACG
CGACGGATGACAGATACCCCGGTACAACGGAATTGCTCATTCTTCTCATCAAAGTATTGAGTGACACCTGCTTG
TGACCTCACTTGTTGAATTGGCTCAAGTGCTTGTAGCCTATTGAACCCGCACCCCCTTAAACCTCCTTGCCGAGA
GCTTTGCCATGGAGTAAAGCTCTGGTCGAATAGCTGAGCCATGGATCCATGGCACAACAGGAAAATGCAGAA
GTAAAACAACACTGATGGAAAACTAGTAGTTGCCATGATTGGTTGAACAGGTTGTTGTACAAGGTGGATTGGT
GCTAGTTTCCTAATTGTAAGCTTGGTTTTATAGCTAGAAAAAGCAGTTTCAGATGCACATGGTGACGTAATTAT
TAATAAAGATAA

>chr1D:378684635-378686841

TAGTCGGAGAATATAAAATTAAATAAAAAAGAAGACATAAAGAATATAACATGATGAAGTTATTTATTAAGAT
AAGCGGTTATATACCACCAACCGACACACTTGTGATGCTTTTATTTCACTATATTGATCCTCATCACTCGCACTA
ATTTACTCTGATGCCTTATTAGTCAAAGATGACACATCCTCGTCCTCTGGATAAGGTTGGAAGCCCGTTTGGGT
AAATTTTGGAGCGAATACACCAAACTCTTTCCCCCTGTTGTTTTTGAGGTTTTGGGCTTCCTGCCTGGAAATACG
GTACGCATTGGCAAGCACATCCACGGGCAAGGCACGAAGGATGGAGCTCTTTCCTGCGATGTGGCTAACCAT
GGAGTTTGGGTTGGTCTTGAATGAGATATACTGGCATCCTTCACGCTCCGCCTTCTTGAGAACAACGTAGTGTT
GTGGTACGATTAGCAACTGCCCTTGGCGAAGACGGTCATTGAATACAGTCTGGCCATTATTATTGACAACTTGA
ACTCGAGCATGCCCTTGGATCATGTAAACCACGCTATGCGCATTGATGTTCCAGAATGGTGAAAGAACAGCAT
TCTGCAATAGCAATTATTTGTATGCCATATTATTAATCGGATGTTATGAAGCTTGGAATATCTAATAATAATATA
GTGCATTGAAGGTAGTATGGTATATACCTGGTATAGATTTACTCTTGTAGCGTTCATTTGCACGAGGTTAAGGA
TGGGAAAATTATTGCCATGGAAACGTGTTATCCTGCCAGCACGTGGGTTGTATGTGTCGGCACGTTTGGGGTT
TTCAATGTTCTGCCTTGCCTCCAATGAACAAAAGTTCTCCTCCAAACCATTGAAACTTTTATCCCCTGACTGTCCA
ACCTGGTATTGAGTTGGTTGTGCTTCTTGATATTGGGTTGATTGCCCTACCTGGTATTGGGTTGATTGTCCTTCT
TGACTTCGAATTGGTTGATAGGCTTGCTACTCTACTAGTCCTTGTTGGGACACTGTTGGCTTCAAGAACCGAAG
GCCTTGACTCACACGAATTATCTCACCCCTTTGGTCGTTTTGACTCTGGATCCTCTGTGCTGCTTGTTGGCTTATA
CCAAGAGCCTCACTAAGAAGTTGGATATTGAATCTACTGAATATGTTTTGTCCAACTTGTTGCCCTATCTTGTTG
TTACCAGCCAACAAGAACTCCTGAGATTTCATATTGATGTAAAACGAAGTTCCTTAATATCCCACTAATTTATAC
TTTAAAATCCTAGTTAGGTTAATATTGAATTATATATCACTATGTAGATTAATTTGTATAGTTACCTTTTGTCTAG
GTTCAAGCTGATTCGCATTGTTGTTTACGTCGAAGACATAGAGAGCTACAATCGGCACATCACCATCGTTGTAG
CACCAGTGTACAATGCCAGCCGGCAGCGCAATAACATCTCCTTGTTTAAAACAGTGAACTCTTTTGTGCTCATCT
TTGAGATGGCTTTGGCTTTGACCCTGAGCTTGATCAAATGGTTGAAACTGTTGTTGGAAGGTTGCTGGGCATCC
AGGTAAAGTCAATCCCGTGAAACCCCTACCTAAAATAAGACACAATTGTTTCAATATTAAAAACTTATGAATGA
GGTAACATTTGGAACATCTGAATTTGAAGTGTAGTATAGAAATGCAATCACCATATTCAATTATACACGAACCT
TGAAGGACGTATACCAATCCGGGTGCATTGTGGTATTGAGGTAACAAGAGGCCTTGGGGCTCAATAACGCGA
CGGATGACAGATACCCCGGTACAACGGAATTGCTCATTCTTCTCATCAAAGTATTGAGTGACGCCTGCTTGTGA
CCTCACTTGTTGAATTGGCTCAAGTGCTTGTAGCCTATCGAACCTGCACCCCCTTAAACCTCCTTGCCAAGAGCT
TTGCCATAGAGTAAAGCTCTGGCCGAATAGCTGAGCCATGGATCCATGGCACAACAGGAAAATGCAGAAGTA
AAACAACACTGATGGAAAACTAGTAGTTGCCATGATTGGTTGAAGAGGTTGTTGTACAAGGTGGATTGGTGCT
AGTTTCCTAATTGTAAGCTTGGTTTTATAGCTAGAAAAAGTAGGTTCAGATGCACATGGTGATGTAATTATTAA
TAAAGATAAGAAGATATAAATGGTGACTAGACATTGTTTAAAAAGCAAATAGATAT

>chr1D:382282210-382284478

ACTTTGAAAAAAAATCCTGTCCAACTGGCCCATATATCTCGTCAATATGCATGCACATGTGGTAAAAACAAGCA
GAAAATCGAGGGAGGGGGAAGGAGATTATTCAGAAGAGATAAATTTAAGAAAGGTACAAGACACAAACAAG
TGATATAACATGGTAAAGTTTTTATTCAGGTAAGCACTTATATGACACTAGGTTGCACACTTGCAATGCCTTTAT
TTTACTATACTAGTTCCCTTTACACTCATTCACTCGGATGCTCTAACAGCCGAAGATGACCCCTCCTCGATGTCTT

GATAACTCTGGAAGCCTGTTTGGGTAAGTTTAGGTGTGAATGCACCAAACTCTTCTCCTCGGTTGTTTTTAAGG
TTTCGGGCTTCTTGTCTAGAAATGCGGTATGCATTGGCGAGGACATCGATAGGTAAGGCACGTAGGATGGAG
CTCTTTCCTGCGATGTGACTAACCATGGAGTTTGGGTTAGTCTTGAATGAAATGTATTGGCATCCTTCACGCTCT
GCCTTCTTGAGAACAACAAAGTGTTGTGGTACGATTAGCAGTTGTCCTCGACGAAGAATATCATTGAATACAGT
CTGGCCATTGTTGTTGACGACTTGAACTCGAGCATGTCCTTGGATCATGTAGATGACACTGTGCGCATTAATGT
TCCAGAATGGTGAAAGAATGGCATTCTGCAATAGTAATTTATAAGATCATATTATTAACCGATTGTTATGAATC
TTATACTACCTAAAAATAAGAGAGTCTATAGAGTGTAGTATCATATATACCTGGTATAGATTTACTCTTGTAGCA
CTCATTTGCACAATGTTAAGGATGGGGAAATTCTTGCTATTGAGACGTGTTATCCTGCCAGCACGTGGGTTGTA
TGTGGCGGCATGTTGGGGGTTTTCAATGTTTTTCCTTGCCTCCAATGAACAAAAGTTTTCCTCCAAACCGTTGAA
ACTTTGGTCCCATGATTGTCCTGCCTGGTATTGACTTGATTGTCCTCCTTGATATGGGGTTGATTTCCCTACCTG
GTATTGGGTTGATTGCCCTACCTGGTATTGGGTTGCTTGTCCTTCTTGAGTTTGAATTGGTTGGTAGACTTGCTG
CTCTACTGGTACTTGTTGTTACACAATGGGCTTCAAGAATTGAAGGCCTTGACTCACACGGATTATCTCACCTCT
TTGGTCATTTTGACTTTGGATCCTTTGTGCTGCTTGTTGACTTATACCAAGGGCCTCACTAAGAAGTTGGACACT
TAATCCACTGAGTATATTGTTTCCAGATTGTTGCTCTCTCTTGTTGTTACCAGCCAACAAGAATTCCTAACAATTT
ATACCGATAGAGTAAAACAAAGTTCATTAATACCCCAATAATATATACTTAACAAAAAAATTGTATTACTTCTAA
ACAATTATATATCTGTTATGTGGATTAATTTGTATAATTACCTTTTGTCTAGGTTCAAGCTGATTAGCGTTGTTGT
TTACATCGAAGACATAGATTGCTACAATCGGTGCATCACCGTCGTTGTAGCACCAGTGCACAATGCCTGCCGG
AAGTGCAACAACATCTCCTTGTTTGAAGCGTTGAACTCTTTGGTGCTCATCCTTAATAGTTTGGCTTTGGCTTTG
ACCTTGAGCAAACTGGGATTGATCAAATGGTTGGAACTGTTGTTGGAAGGTCGCAGGGCATCCAGGGAAAGT
CAACCCCGTGAAACCTCTACCTAAAATAAAACATATTTGTTTGAATATTTAAAACTTATGAATGGGTTAACATTT
GACACCACTAAACTCCTGAGTGTAGTATGACAATGCAATTCTATATTCACTTAGACACTAACCTTGGAGGATAT
ACACCAAGGCAGGAGCATTGTGGTATTGAGGCAGCACAAGGCCCTGAGGTTCGATTACACGGCGGATGACAG
ATACACCTGTACAACGAAATTGCTCATTTTGCTCATCAAAGTACTCAGTGATACCTGCTTGTGACCTCACTTGTC
GAAGTGGTTCAAATGCTTGTAGCCTATCAAATCTGCACCCACGTAAACCTCCTTGACGAGAACTTTGCCATGGA
GTAGAGCTCTGGCCAAACAACTGAGCCATGGATCCATGGAACAAGAGGAAAATGCAAAAGTAAAACAACATT
GATGGAAAACTAGTAGTTGCCATGTTTGTTTGAAGTGATTGTAGAAGATGGATTGGTGCTAGTTTCCTAATTTT
AAGCTTGGTTTTATAGTCACAAAAAGTAGTTTATGTTGTACA

>chr1D:382314566-382316778

GATATAACATGGTAAAGTTTTTATTCAGGTAAGAACTTATATGACACTAGGTTGCACACTTGCAATGCCTTTATT
TTACTATACTAGTTCCCATTACACTCATTCACTCGGATGCTCTAACAGCCGAAGATGACCCCTCCTCGATGTCTT
GATAACTCTGGAAGCCTGTTTGGGTAACTTTAGGTGTGAATGCACCAAACTCTTCTCCTCGGTTGTTTTTAAGGT
TTCGGGCTTCTTGTCTAGAAATGCGGTATGCATTGGCGAGGACATCGATAGGTAAGGCACGTAGGATGGAGC
TCTTTCCTGCGATGTGACTAACCATGGAGTTTGGGTTAGTCTTGAATGAAATGTATTGGCATCCTTCACGCTCTG
CCTTCTTGAGAACAACAAAGTGTTGTGGTACGATTAGCAGTTGTCCTCGACGAAGAATATCATTGAATACAGTC
TGGCCATTGTTGTTGATGCCTTGAACTCGAGCATGTCCTTGGATCATGTAGATGACACTGTGCGCATTAATGTT
CCAGAATGGTGAAAGAATGGCATTCTGCAATAGTAATTTATAAGATCATATTATTAACCGATTGTTATGAATCT
TATACTACCTAAAAATAAGAGAGTCTATAGAGTGTAGTATCATATATACCTGGTATAGATTTACTCTTGTAGCA
CTCATTTGCACAATGTTAAGGATGGGGAAATTCTTGCTATTGAGACATGTTATCCTGCCAGCACGTGGGTTGTA
TGTGTCGGCATGTTGGTGGTTTTCAATGTTTTTCCTTGCCTCCAATGAACAAAAGTTTTCCTCCAAACCGTTGAA
ACTTTGGTCCCATGACTGTCCTGCCTGGTATTGACTTAATTGTCCTCCTTGATATGGGGTTGATTGGCCTACCTG
GTATTGGGTTGATTGCCCTACCTGGTATTGGGTTGCTTGTCCTTCTTGAGTTTGAATTGGTTGGTAGACTTGTTG
CTCTACTGGTATGTGTTGGGACACAATGGGCTTCAAGAATTGAAGTCCTTGGCTCACACGAATTATCTCACCTC
TTTGGTCATTTTGACTTTGGATCCTTTGTGTTGCTTATTGACTTATACCAAGGGCCTCACTAAGAAGTTGGATAC
TTAATCCACTGAATATGTTGTTTCCGGACTGTTGCTCTCTCTTGTTGTTACCAGCCAACAAGAACTCCTAACAATT
TATATGTATAGAGAAAAACAAAGTTCATTAATACCCCAATAATATATACTTAACAAAAAATTATAGTACTTCTAA
ACAGTTATATATCTGTTATGTGGATTAATTTGTATAATTACCTTTTGTCTAGGTTCAAGCTGATTAGCGTTGTTGT
TTACGTCAAAGACATAGACAGCTACAATCGGTGCATCACCATCGTTGTAGCACCAGTGCACAATGCCTGCCGG

AAGTGCAACAACATCTCCTTGTTTGAAGCGTTGAACTCTTTGGTGCTCATCCTTAATAGTTTGGCTTTGGCTTTG
ACCTTGAGCAAACTAGGATTGATCAAATGGTTGGAACTATTGTTGGAAGGTTGCAGGGCATCCAGGGAAAGT
CAACCCCGTGAAACCTCTACCTAAAATATAGCATATTTGTTTGAATATTAAAAACTTATGAATGGGTTAATATTT
GGCACCACTAAACTCCTAAGTGTAGTATGACAGTGCAATTCTATATTCACTTAGACGCTAACCTTGAAGGATGT
ACACCAAGGCAGGAGCGTTGTGGTACTGAGGTAGCACAAGGCCTTGAGGTTCGATTACACGGCGGATGACAG
ATACACCGGTACAACAAAATTGCTCATTTTGCTCAGCAAAGTACTCAGTGATACCTGCTTGTGACCTCACTTGTC
AAAGTGGTTCAAATGCTTGTAGCCTATCAAATATGCACCCAGGGAAACCTCCTTGACGAGAGCTTTGCCATGG
AGTAGAGCTCTGGCCAAACAACTGAGCCATGGATCCATGGAACAAGAGGAAAATGCAAAAGTAAAACAACAC
TGATGGAAAACTAGTAGTTGCCATGTTTGTTTGAATTGATTTTAGAAGATGAATTGGTGCTAGTTTCCTAATTTT
AAGCTTGGTTTTATAGCCGCAAAAAGTAGTTTAGGTTGCACATGGTGACGAGTAATTGTTAAGAGAGATAAGA
AGATACGCACTGTGACTAGGACATGGTTTGAATTGTGTATATATATATAACTTTTCCTCG

>chr1D:385771637-385773788

GGATATAACATGGTAAAGTTTTTATTCAGATAAGCACTCATATGACACTAGGTTGCACACTTGCGATGCCTTTA
TTTCACTATACTAGTTCCCATTACACTCATTCACTCGGATGCTCTAACAGCCGAAGATGACCCCTCCTCGATGCC
TTGGTAACTCTGGAAGACCGTTTGGGTAAGTTTAGGTGTGAAAGCACCGAACTCTTCTCCCCGGTTGTTTTTGA
GGTTTCGAGCTTCCTGTCTAGAAATGCGGTATGCATTGGCGAGGACATCCACAGGAAAGGCGTGTAGGATGG
AGCTCTTTCCCGCGATGTGACTAACCATGGAGTTTGGGTTAGTCTTGAATGAAATGTATTGGCATCCGTCACGC
TCCGCCTTTGATAACAACAAAGTGTTGTGGTATGATTAGCAGCTGTCCTCGATGAAGAATGTCATTGAATACGG
TTTGACCCTTGTTATTGACGACTTGAACTCGAGCATGTCCTTGGATCATGTAGATGACACTGTGTGCATTAATAT
TTCAGAATGGTGAAAGAATGGCATTCTGCAATAGTAATTTATAAGATCATATTATTAACCGATGATTATAAATC
TTAGACTATCTAAAATAAGAGAGTCTATAGAGTGAAATATCATGTATACCTGGTATAGATTTACTCTTGTAGCA
CTCATTTGCACGATGTTAAGGATGGGGAAATTCTTGCTATTGAGACGTGTTATCCTGCCAGCGCGTGGGTTGTA
TGTGTCGGCACGTTGGGGGGTTTCAATGTTTTTTCTTGCCTCCAATGAACAAAAGTTTTCCTCCAAGCCATTGAA
ACTTTGGTCCCATGACTGTCCTGTCTGGTATTGACCTGATTGTCCTTCTTGATATGGGGTTGATTTCCCTACCTC
GTATTGGGTTGATTGTCCTACATGGTATTGGGTTGATTGTCCTTCTTGAGTTTGAATTGGTTGGTAGACTTGCTG
CTCTACTGGTACTTGTTGGGACACAATGGGCTTCAAGAATTGAAGTCCTTGACTCACACGAATTATCTCACCTCT
TTGGTCATTTTGACTTTGGATCCTTTGTGCTGCTTGTTGACTTATACCAAGGGCCTCACTAAGAAGCTGGACACT
TAATCCACTGAATATGTTGTTTCCAAACTGTTGCTCTCTCTTGTTGTTACCAGCCAACAAGAACTCCTGACGATTT
ATATGGATGGAGTAAAACAAAGTTCGTTAATATCCCAATAATATACACTTAACAAAAAAATTACTTGTAAACAA
TTATATATATTTTATGTGCATAAATTTGTATAACTACCTTTTGTCTAGGTTCAAGCTGATTAGCGTTGTTGTTTAC
GTCAAAGACATAGACAGCTACAATCGGTGCATCACCATCGTTGTAGCACCAGTGCACAATGCCTGCCGGAAGT
GCAACAACATCTCCTTGATTAAAGCGTTGAACTCTTTGGTGCTCATCCTTAATAGTTTGGCTTTGGCTTTGACCT
TGAGCAAACTGGGATTGATCAAATGGTTGGAACTGTTGTTGGAAGGTCGCAGGACATCCAGGGAAAGTCAAT
CCCGTGAAACCTCTACCTAAAATAAAACATATTTGTTTGAATATTAAAAACTTATGAATAGGTTAATATTTGGCA
CAACTAAACTCCTAAGTGTAGTATGACAATGCAATTCTACATTCACTTAGAAACTAACCTTGAAGGATGTACAC
CAAGGCAGGAGCGTTGTGGTATTTAGGTAGCACTAGGCCTTGAGGTTCGATTACACGGCGGATGACAGATAC
ACCGGTACAACGAAATTGCTCATTTTGCTCATCAAAGTACTCAGTGATACCTGCTTGTGACCTCACTTGTCGAA
GTGGTTCAAATGCTTGTAGCCTATCAAATCTGCACCCACGTAAACCTCCTTGACGAGAGCTTTGCCATGGAGTA
AAGCTCGGGCCAAACAACTGAGCCATGGATCCATGGAACAAGAGGAAAATGCAAAAGTAAAACAACATTGAT
GGAAAACTAGTAGTTGCCATGTTTGTTTGAAGTGATTATAGAAGGTGTATTGGTGCTAGTTTTCTAATTTTAAG
CTTGGTTTTATAGTCACAAAAAGTAGTTTAAGTTATACATGGTTACATAATTGTTAACAGAAATAAGAAGATAT
G

>chr1D:388734854-388736854

ACTGAGTTCTTTCTTTTTATTATTTTCTCTCAGTTTTGGGTTTTTTGGGGGGTCATGAATTATTATTCCCCATGGA
GATGAGATCTGCCCCCCAGGCGATAGCGAGCGAGTATTATTTATTCAGATTTTACCTGTGATTCTTTGACTGAC
TCTGGTCGGGGCTCCCTCCTCGCTTCCTTCCGGGTGAATCTCTCTCTCTCTCTCTGGAGTGCAATAAGACGCTGT

TGTAGCAAGTGTGGGAAAATGATAATCTGGCGAATGACATGGTCAATCGATACTATACTAGCATATGGGTTTC
CAGCAGATTTATGGTCCCAAATTGAAGAGCACATGATGCAAATAGCCCAATTTTTTGGTCAGGATTTCTGTGAA
ATTTGTAACTGTGACTGCTTTTCGTGTGGATCTTTATAACTTGTTCGAAATTGCTGTGGGAAAATCAAGGATGA
TTTATTTGTCTGGGGGGAATTGAAATGGGAGCACGTGGCAGTGGCAGGGGAGATCTGCCCTGGCAGTGCCGG
CAGTGAGATCTGCAGGCGATGAACTGGAATGAATGAATGCGTCGTGTCGCCCAGATCTACTCCTCCATTATTG
GAGCCTTGGAGTTAGGGAGCTCCAGCTTTCGGTAGGCGTCCTACTCGTACAGTACCGCACACATGGCTGGACG
GCTGCTCGTGCAGGTGCAGACCTCGCTCGCTCGAAGGCAGCCGCCGACAGCAACCTTTCGTGTGTCGGCTGAC
AGGCAGTCCGTTCGTACGAGCAACGGAGCAAGATTGGGGGTGCATCGACCTTCGCGCAATGATGACCATGGA
TTAACCGAACAAGCCCTAAGGTGTATTAAATCCCATCAAATCCACTCCAATACTTTCCAATTCCTTTTTAGGGA
GAGATGAACCGAACAAAGCCTAAAGCAGCAAAAGGCTTCATTTCCACCGGCATCCAGGAGAAATCCTTCACCC
CTGCTCTAATGGCGTGGCAAAGTAAATTCCTCCACTAGCATCTAGGAGAAATCCTTCACCACTGCTCTAATGGC
GTGGCAAAGTAAGTTCCTCCACTGGCATAGGTAACTGGGATATGGGGCCCTTGTTATTTTGCGAATTGATTTTA
AAAGTAGAGTTATTTTGTGTTGAATTTTTGAGAATAGGTTAATTTTTGTTTCTTGCCTGCTAGCATTCTCTTCTGA
GATTCGGACATAGCACTCCCTCTCCTATTGTTTTCTCACTATTTGTTATTTTTTAATTCCTTTGTCCGACAAATAG
GTGCAACAATTTTTTATCATCCTGCCAACAACCTAGCACCTGGTTTACTTTTTAGTGTAGATTTCAAAAGGCAAT
ATGTTTTGAAATAAATTCAGATTAAACAATCCATTTAAGCATGTGTATTTGTTTTGTCTTTTAAACAAGCTCAAA
ATAGTTTTGTTTCGACAAAATTCAAAGCCTCAATTCCAAAACACAGATCAAACATTAGTTCAAAAGGGCTTGAA
ATGGTATGAAAATTAGTTTCTGCCATGATGTGTGGTGTGCGGATTGCCCACTTAAAACCCTTGTCCCTGCTCTGT
TTATATGATGTCAGTAGCAAGAGGTGGTTGTTCATGATGTGATGAGAGATGTTTGTTGTTGTCTTTTAGTAGGC
CATTTGGTTCTAATGAGGTCAGAGAAAGGGAAGAGCTGAATAGCCTACTATAAGGGGTCTATTATGGGGATG
AGGATGATAAGGTGAAGTGATTAATTAAAAAGAAAATGCTTTTCTCAACAAACTCACTTTACCCTAGGGTTAGG
GATGTTAGGATAATGGATATGTGGAAAATCGTTTATCCCTTTTTGCGGGTAAAATGGATCCTTTATTTCTCAAA
GAGGGCTACAAAGCGGTCATTTTCAAGATCCTGAAGAATGATCTCAGGTCCTGAACCTAACCAAGTCTTAATCC
ATTGATCCTCTCTTGCTAAGTTAGCAAGGCTGTGGCTAACCCTAACTTGAGATTGATCCACCATAACAAATACA
CA

>chr1D:390083639-390085796

AACCTTCTCAATCTCTTACAACAAGAATGGCAACTACTAGTTTCCCATCGCTCTTATCTTGCCTTTGCATTTTCCT
CTTGTTCCATGGATCCATGGCTCAGGTATTTGGCCAGAGTCAACCATGGCAAAGCTCTCGACAAGGAAGTGCT
AGGGGATGCACATTCGATAGGCTACAAGCATTTGAACCACTTCGACAAGTGAGGTCCGAAGCGGGCCTCACT
GAGTATTTTGAGGAGCAAAATGACCAATTCCGTTGTGCTGGTCTATCTGTCATTCGTCGTGTCATCGAGCCTCA
AGGCCTTCTGTTACCTCGATACCACAACGCTCCCGCCTTGGTATACATCCTACAAGGTTGGTTTATGAACTTAAT
AGAGTGGTTATAGCTTTAGATTATATACTTTAGATTTCTATGTGAAAAATATTACAAAAGTATTAGGAATATTTA
ATATTGAATTGAACTTATTTTCTTGTAGGCACGGGTACTGCAGGGCTGACATTCCCTGGTTGCCCGGAGACCTT
CCAACAACAGTTCGGACAATTTGATCAAGAGCAATCTGAGGGTCAGAGCCAAAGCCAAAAGTTTAGAGATGA
GCACCAGAAAGTTCATCACATTAAACAAGGAGATGTTGTTGCGTTGCCAGCTGGCATTGCACATTGGTTCTATA
ATCATGGCCAAGTTCCGATTGTAGCTCTCTATGTTTTTGACATAAACAACAATGCCAACCAGCTTGAACCTAGA
CTAAAGGTAATTGAGAGTATCTAAAACAATCTCTAGGATTGATAGTCTTTTCGACTAATCTACTAGGTTTAGCCT
TTTTGACCAATATCTAAGATTGTTTGATATATTTATTACTTGATCAAATTGCAGGAATTTTTGTTGGCTGGAGGT
AGTAGGACACTACAACAGTATGTGAGTCAAAACATATTCCATGGATTCAATTTACAGTTGCTTGGTGAAGCCCT
TGGTATAAGTGAACAGACATCTCAAAGACTTCAGAATCAAAATGACCAAAGAGGTGAGATAATCCGCGTGAAT
CGTGCGCTTCAAATTCTACAACCAATTGTGATGCCACAACAAGAGCAACCGTCGTATCAAGAAGAGCAATCTCA
AGCAACACAACCTCAGGTTGGTCAGTCTCAAATAGAGTCGTCTCAGACATGGCAATCTCAGGTAGAGCAATCA
ACTAGTGGCCGTTGGAATGGTTTGGAGGAGAACTTTTGTGATGATAAGCCACCAATGAACATTGAAAATCCTA
CACATGCTGACGCATACAACCCACGAGCTGGTAGGATAACACATGTCAACAGCCAAAAGTTCCCCATTCTTAAT
ACCGTGCAAATGAGTGCTACAAGAGTAAACCTCTATCAGGTACTTACGATGTTACATTTCAATACGTTCTTATCT
ATATTTCTTGTAAGCTTAAAAATAGAAGCATAATTCTATGTATTTTTACTTACTCAACTTGATTAACTGCAGAAC
GCCATTCTTTCGCCATTCTGGAACATTAATGCTCATAGTGTGATGTACGTGATCCAAGGCCACGCACAAGTTCA

GGTTGTTAACAATCATGGCCAAAATGTGTTCAACGACATTGTTCGCCCGGGTCAACTATTAATCATACCACAGA
ACTATGTTGTTCTAAAGAAGGCACAACGCGAAGGATGCCAATACATTTCATTCAAGACCAACGCAAACTCCATG
GTTAGCCACATCGCCGGGAAGAACTCAATCCTCCGTGCCCTTCCTATTGATGTCATCGCTAATGCATACCACAT
ATCCAAGCAAGAAGCCCGAAACCTCAAATACAACAGAGGAGAGGAGTTTGGCGTATTCACTCCTAAGTTTTCT
CAAAGTGGCTTCACAAGAGCTCAAACGGGATTGGTGAACTAGTTTAATGGGAGGACAATCTAGTAAAGTAAA
ATAAAGACATCAAGAAAAGTGTGTAGCACTTTGATGTTCCCTGCATGTCCGCCTCACCATGATATGAAGACAAT
AAAAGCTTGCGTATGTTCCGTATGTGTTTTGTTGTTGGAAATTTTTACACATTTCCGATTTGCAAATTTCACTACC
CAATTTTTATGCTCCACATTTTCCATGTCATTGGCACATCAACGTAATAAACATCACAGACAATGTCAACTATCC
CCGTATATCTT

>chr1D:390205451-390208794

AACCAATATTCTCAATCTCTTCCAACGAGAATGGCAACTACTAGTTTCCCATCGCTTTTATCTTGCTTTTGCATTT
TCCTCTTGTTCCATGGATCCATGGCTCAGCTATTTGGCCAGAGTCAACCATGGCAAAGCTCTCGACAAGGAAGT
GCTAGGGGTTGCACATTCGATAGGCTACAAACATTTGAACCGCTTCGTCAAGTGAGGTCTGAAGCTGGCCTCA
CTGAGTATTTTGAGGAGCACAATGACCAATTCCGATGTGCTGGTCTATCTGTCATTCGTCGTGTTATCGAGCCT
CAAGGCCTTTTATTACCTCGATACCACAATGCTCCCGCCTTGGTATACATCCTTCAAGGTTGGTGTATGAACTTA
ATAGAGTGGTTATAGCTCTATATTAGATACTACAGATTTGTATGTGAAATATGTTATAAAAGTATCAGTAATTTT
TAATATCAAATTAAACTTGTTTACTTGTAGGTACTGGTTCTGCAGGGGTAACATTCCCTGGGTGCCCAGAAACC
TTCCAACAACAGTTCGGACAATTTGATCAAGCGCAATCGGACGGTCAACAAAGCCAAAAGTTTAGAGATGAGC
ACCAGAAAGTTCATCGCATTACACAAGGAGATGTTGTTGCGTTGCCAGCTGGCATTGCACATTGGTTCTACAAT
CATGGCCAAGTGCCGATTGTAGCCCTCTATGTTTTTGACGTAAACAACAATGCCAATCAACTTGAACCTAGACA
AAAGGTAATCGACGGTATCTCAAACAATTTTAAGCTTGATAATATTTTTTACTAATCAACTAGGTTTTAGTCTTTT
CGAACAATCTCTAAGATTGTTGGATATATTTTTTACTTGATCAAATTGCAGGAATTTTTGTTGGCTGGTGGTAGT
AGGACACTACAACAGTATGTGAGTCAAAATATATTCCGTGGATTCAACTTCCAATTGCTTGGTGAAGCCCTTGG
TATAAGTGAACACACATCTCAAAGACTTCAGAACCAAAATGACCAAAGAGGTGAGATAATCCGCGTGAATCGT
GGGCTTCAAATTCTGCAACCAATTGTGATGCCACAACAAGAGCAACAGTCATATCAAGTAGAGCAATCTGAGG
CAACACAACCTCAGGTTGGTCAGTCTCAAATAGAATCGTCTCAGACATGGCAATCTCAGGTAGAGCAGTCAAC
TAGTGGGCGTTGGAATGGTTTGGAGGAGAACTTTTGTAATCATAAGCCAAAAATGAACATTGAAAATCCTACA
CGTGCTGACACATACAACCCACGTGCTGGTAGGATAACACATGTCAACAGCCAAAAATTCCCCATTCTTAACAC
TGTGCAAATGAGTGCTACAAGAGTAAACCTCTATCAGGTACTTACAATGTTATATTTCAATACATTCTTACCTAT
ATATCTTGTAACCTTAAACACAGAAGCCTAATTTTATGTGTTTTTACTTACTCAATTTTATTAACTGCAGAACGCC
ATTCTTTCGCCATTCTGGAACATTAATGCTCATAGTGTGATGTACGTGATCCAAGGCCACGCACGGGTTCAGGT
TGTTAACAATCAAGGCCAAAATGTGTTCAACGACATTCTTCGCCCGGGACAACTATTAATCATACCACAAAACT
ATGTTGTCCTAAAGAAGGCACAACGCGAAGGGTGCCAATACATTTCATTCAAGACCAACGCAAACTCCATGGT
TAGCCACATCGCCGGGAAGAACTCAATCCTCCGTGCCCTTCCTATTGATGTCATTGCTAATGCGTACCACATATC
AAAGCAAGAAGCCCGAAACCTCAAATACAACCGAGGAGAGGAGTTTGGCGTATTCACTCCTAAGTTTTCTCAA
AGTGGCTTCACGAGAGCTCAAACGGGATTGGTGAACTAATGTAATGGGAGGACAATCTAGTAAATTAAAATA
AAGACATCAAGATAAGTGTGTAGCACTTTGATGTTCCCTGCATGTCCGCCTCATCATGATATGAATACAATAAA
AGCTTCCGCGTGTTCGGAATGTGTTTCGTTGTTGGAAATTTGTATACATTTTTTTATTTGAAAATTTCATTAGCCA
TTTTTTATGCTCCATGTTGTCTATGTCATTGGCACATCAACATAATAAACATCACATACAATGTCAACTAACCCCC
GTATATCTTCTATATCTTTTGATAATGTGAGGGCTGCCGTATTTTATTGTTGTACTATTATACAAGGACTCAAGG
ACACGATCATAGGAATGTCTGCAAGTCGCGTTGGCACAACCGCACAAACATGTGGGCCTCACTTTGCAGCCGA
GGCCTAGTGAACCTGTGTCTAATGAGATCAATTGGAGGAACCTGGCTATGTTATTTACACTTGGATGTTATCTA
GAGACGTTCCACCATGTTCTCTCTTAACATCAATTGGAGAGGCAGTTTTGAGAGTTGCTTCCTGGTGGCTGGGC
CTTTTCCTTGCCCCTCTCGCCGGATTAGCCTACTACTAGAAATCCCCAAATTTCCGACGGTGATATTATCGTCGG
CTAAATTCATTCACCGTAGGTAAACCTTAAATCCGACGGTGTACCGACGGTTATGTCCCGTCGGCGATGCCCGG
TCGGCCATTGCTCCAATAGCCGACGGCCCGGTCGGTTAATACATTTTCTTACGGCAATAAACTGACGGTGAGTC
GTCGGCCATATTGTGGTAACCAGCCACCCGATCACCCTTTGATGATGCTTATAGCCGACGGCTCCTTGTCGGGT

GTTTAGCTTTCTGACAGGTGCTACATCCCGTCAGTTTTCGTGATAGTTGAGTGTACATTGTCCCGTCAGGATGG
TTATAACCAAGAGTAAAAAACAACCTCCAGTTAGTATAATTTACGATGGTATATGCAACCCATCGGGGATTCAT
GTATTTGAGAGGCCACCCACACATTCATTTTATTAATTAACCAATGGGCGTAAAGTCCATATCGCCATATGGTA
TAGCCCAGATGTTAGCTCGATCATCGATCATTGTTATACACCACAGAAACATGATTCATATTCCAGAATATTGG
CATTTAAAAGATATGTTAGAATTAACAACCAGAACACAAAAGAACACATGGCATTAGTAATATCCATCAGGTC
GAGTGCAAAAGAAAATAATGGCTCAATCAATCTGCAAAAGCCAAATTGTACAAATATTTGAGAATTTTTTGCAC
GACTCAAAGGTCACCAGATTAATCACCAATGATCTTCATCATCTTGATCACCACGCCCATTTTTTCCATAATGGT
CATCACCATAGCCATCATCATAACCGTAGCCAGTCATCTTCATCACCCTTGCCATAACCATAGCCATTGTCATCG
CCCTCA

>chr1D:390228995-390230995

AGTTTTGGGGGGGGGGGGGGGGGACTAAAACTTTTCTCCGTAACGGCCGAAAGGGAATTAGTTTGATGCGT
CCATGGGCGTTAGCGGACGAAAGAAAGTCCTCTAAGCATTTATTGGTGATCGGTTAGAGTTGCTCTAATAATG
TTGTGAATATTTGTGGAACTAGACAATGACCCCTCAACCATGGAGGAAAGAGGGGGCAGAACTGCTATAGCC
GTAAAATAAAATTAGCATGACCAGAATTTTTGAGGATATATAGAACCAAGGGCGATGTTGACGCTGGTATACC
GTAAAATAAAATTAGTGAAAGGAGGGGAAGGCAACACCATTTCAGTGGCCGTCCATCACTATAGCACGAGTA
GAGTAGGAGCCTGCCTTACACGCATGAATGAATGAATATAAATATAAAAGGGCAAAGAGGATCTACAGAGTA
GGAGCCAGCCAGCCAGCCATCACTCCCGTTCCATTCCATCCCACCATGGAATTACGCGGACAGCGACTCCACAC
CGATCGTCCACCAGCGCAGCGATAGCGACGGCCGGCCGGTCCGAGTTCTTCGTGCACCCGTCCCCTTGCACAT
TACTAGCAGCTACAGTATTCCCGAAAGTTTTAGGAAACTGCTAGGCGTCCCCGGACCATGGGAGAGCTCCCAT
CGTCCTGCTTCCCGTGCTGACACGTGTCCATTTGCTGCAAAACATTCCGTATTTGCTGTATATGTCCCCGCTTTT
GCTTCTGAAGGAAAAAAAAAACGGTTCGTTTTAGAAATAGAACGTTTCGCCGGAGACCTCGTCGGAGACCATG
CAGCAAAAAAAATTATGCTAATGCAGCAAAAACGTCTTCGCTGGAGACATCGTCGGAAACAACTCGTTGATGA
CCCTCGCCGGAGACCATGCAGCAAAAAAAGTTTCCGGCGAAGTCGGTTTTGCTGCATTAGCACAATATTTTTGC
TGCATGGTCTCCGGCGAGGTATCCGGCGACACGTTCTATTTTTAAAACGAACTATTTTTTTTGCTTCGGAAACAA
AAACGAGGACATATATAGCAAAAGCGAGCCGTTTTGCAGCAAAAATGAAAATTGGGACGATTTTGGGTGAGG
AGGACGATAGGAACCCCCAGTCGCCGGGAAATCCATATCATTTTCCAAAGTTTTACTAGCTCGCTTGCAGGCGT
CTCTAGGTAATCAATGTCATGTACGTATGGAGTAGGTACGTAACAAAAACTCGAAATTAATGTAAGCCAATGA
AGATGTCGACGGCATGTCTGATCACGAGCTGCCAGCGCCGTTTCATGCATGACGGTTTTTGGATACGATCGAT
GCAATGCAAGCCTCCACAAGGGACGACGTGCCGTGCCGCCTGTGTCTCCCGAGAGAAACAACAAAATCTTCAT
GGAGGTGGACGGCACGTCGACATGCACGTAAGATAAGATTAATCGCCGGGCCCACATGGAACCCCGCATGCA
CGCTGTACGGCGGGTGTGACACCCACGTAGGCCTCGTTTACTTCTCCTGTTTCTTCCGGGATGGGAGGGTATTT
TCCCGGCTTACTTGTTAATCCCCGAAAACACCGGCAAGGTTGTTTACTTCGAGTGTTTTTCCGAGTTTAATCCCG
GCCCATCCCGAGAAATACCGGACCAAACCGAACAAAGAACGTGTATTCTCGCACACGAGTCTGGACTCGCGTA
TTGGCGAGGAAGGAAGCGAGAAGAGTGGAGGCGGCACGGATGGCGCGACGGCGCGGACGTCAACTTGGGG
AAGCTACCGACGGGCACCTCCTCCGGCTTCTCTCCGGTGAGCCCCCCCCCCCCCCCCCCCCCCCCCCCCACAC
ACACACACACACACACACCAAGCCTCTTCCCATCTACTCCCGTCCTAGAATTTCTCGGCCGCCGCCGGCGTCCAC
CTCCCTCCGGTCTGGTTCTTTAGATCTTGGGGAGAGAAGAGAGAGAGACCATACACGACATGGTGGAGATGG
ATTCGAGGAACATTGGCAGGTCAAC

>chr1D:390285216-390287593

GGAGCATAAAAATTGGCTAATGAAATTTGCAAATCAAAAAATGTATACAAATTTCCAACAACGAAACACATTCA
GAACACGCGGAAGCTTTTATTGTATTCATATCATGATGAGGCGGACATGCAGGGAACATCAAAGTGATACACA
CTTATCTTAATGTCTTTATTTTACTTTACTAGATTGTCCTCCCATTACATTAGTTCACTAATCCCGTTTGAGCTCTC
GTGAAGCCACTTTGAGAAAACTTAGGAGTGAATACGCCAAACTCCTCTCCTCTGTTGTATTTGAGGTTTCGGGC
TTCTTGCTTTGATATGTGGTACGCATTAGCGATGACATCAACAGGAAGGGCACGGAGGATTGAGTTCTTCCCG
GCGATGTGGCTAACCATGGAGTTTGCGTTGGTCTTGAATGAAATGTATTGGCATCCTTCGCGTTGTGCCTTCTT
TAGAACAACATAGTTCTGTGGTATGATTAATAGTTGTCCCGGACGAAGAATGTCGTTGAACACATTTTGGCCAT

GATTGTTAACAACTTGAACCCGTGCGTGGCCTTGGATCACGTACATCACACTATGAGCATTAATGTTCCAGAAT
GGCGAAAGAATGGCGTTCTGCAATTAATCAAATTGAGTAAGTAAAAGGAAATAGAATTAGACTTCTATTATTA
AGCTTACAAGATATATAGATAAGAATGTGTTGAAATGTAACATCGTAAGTACCTGATAGAGGTTTACTCTTGTA
GCACTCATTTGCACAGTGTTAAGAATGGGGAACTTTTGGCTGTTGACTTGCGTTATCCTACCAGCACGTGGGTT
GTATGTGTCAGCACGTGTAGGATTTTCAATGTTCATTGTTGGCTTATGATCACAAAAGTTCTCCTCCAAACCATT
ACAACGGCCACTAGTTGATTGCTCTCCCTGAGATTGCCACGTCTGAGACGACTCTATTTGAGACTGACCAACCT
GAGGTTGTGTTGCTTGAGATTGCTCTTCTTGATACGACTGTTGCTCTTGTTGTGTCATCACAATTGGTTGTAGAA
TTTGAAGCGCACGGTTCACGCGGATTATGTCACCTCTTTGGTCATTTTGATTCTGAAGTCTTTGAGATGTCTGTT
CACTTATACCAAGGGCTTCACCAAGCAACTGTAAATTGAATCCATGAAATATGTTTTGACTCACATACTGTTGTA
GTGTCCTACTACCTCCAGCCAAGAAAAATTCCTGCAATTTGATCAAGTAATAAATATATCAAACAATCTTAGATA
TTGGTCAAAAAGGCTAAACCTAGTAGATTAGTCGAAAAGACTATCAATCCTAGAGATTGTTTTAGATACTCTCA
ATTACCTTTTGTCTAGGTTCAAGCTGGTTAGCATTGTTGTTTATGTCAAAAACATAGAGAGCTACAATCGGCACT
TGGCCATGATTATAGAACCAATGTGCAATGCCAGCTGGCAACGCAACAACATCTCCTTGTTTAATGTGATGAAC
TTTCTGGTGCTCATCTCTAAACTTTTGTCTTTGGCTCTGACCCTCAGATTGCTCTTGATCAAATTGTCCGAACTGT
TGTTGGAAGGTCTCTGGGCAACCAGGGAATGTCAGCCCTGCAGTACCCGTGCCTACAAGAAAATAAGTTCAAT
TCAATATTAAATATTCCTGATACTTTTGTAACATTTTTCACATAGAAATCTAAAGTATATAATCTAAAGCTATAAC
CACTCTATTAAGTTCATAAACCAACCTTGTAGGATGTATACCAAGGCGGGAGCGTTGTGGTATCGAGGTAACA
AAAGGCCTTGAGGCTCGATGACACGACGAATGACAGATAGACCAGCACAGCGGAATTGGTCATTTTGCTCCTC
AAAATACTCAGTGAGGCCCGCTTCGGACCTCACTTGTCGAAGTGGTTCAAATGCTTGTAGCCTATCGAATGTGC
ATCCCCTAGCACTTCCTTGTCGAGAGCTTTGCCATGGTTGACTCTGGCCAAATACCTGAGCCATGGATCCATGG
AACAAGAGGAAAATGCAAAGGCAAGATAAGAGCGATGGGAAACTAGTAATTGCCATTCTTGTTGTAAGAGAT
TGATAAGGTTGGTTGATGGAGGTGTTGTGGGATGTAATGGGTATTTTATAGCTAAGAAAAGTGGCATACACCT
TATGTAAAGACATGGTTTGTTAAGATAGATGTGTGATGACATGAAAATTTGGACGTAATAGAGAAAACAAATG
CATAAGCATGATTGCAAGCATACCCATAAATGATCTAACAAGTCCGAGTTCTGGTTGAAAACCTCATTTGTATT
TGTTTTCAAACGATTTTCGTGCACACTTGGATAACTGTTACTTTTTCACGTATACTCAAAGCTTCTCAAAGAAGT
ATGTA

>chr1D:390304396-390307793

TTCCAACAACGAAACACATAGAACATACGCAAGCTTTTATTGTCTTCATATCATGATGAGGCGGACATGCAGGG
AACATCCAAGTGCTACACACTTTTCTTGATGTCTTTATTATACTTTACTAGATTGTCCTCCCATTACACTAGTTCA
CCAATCCCGTTTGAGCTCTTGTTAAACCACTTTGAGAAAACTTAGGAGTGAATACACCAAACTCCTCTCCTCTGT
TGTATTTGAGGTTTCGGGCTTCTTGCTTGGATATGTGGTATGCATTAGCGATGACATCAATAGGAAGGGCACG
GAGAATTGAGTTTTTCCCGGCGATGTGGCTAACCATGGAGTTTGCGTTGGTCTTGAATGAAATGTATTGGCATC
CTTCGCGTTGTGCCTTCTTTAGAACAACATAGTTCTGTGGTATGACTAATAGTTGTCCCGGGCGAAGAATGTTG
TTGAACACATTTTGGCCATGATTGTTAACAACTTGAACCCGTGTGTGGCCTTGGATCACGTACATCACACTATG
AGCATTAATGTTCCAGAAAGGCGAAAGAATGGCGTTCTGCAATTAATCAAATTGAGTAAGTAAAAGGAAATAG
AATTAGACTTCTATTATTAAGCTTACAAGATATATAGATAAGAATGTGTTGAAATGTAACATCGTAAGTACCTG
ATAGAGGTTTACTCTTGTAGCACTCATTTGCACAGTGTTAAGAATGGGGAACTTTTGGCTGTTGACTTGCGTTA
TCCTACCAGCACATGGGTTGTATGTGTCAGCACGTGTAGGATTTTCAATGTTCATTGTTGGCTTATGATCACAA
AAGTTCTCCTCCAAACCATTCCAACGGCCACTAGTTGATTGCTCTACCTGAGATTGCCATGTCTGAGACGACTCT
ATTTGAGACTGACCAACCTGAGGTTGTGTTGCTTGAGATTGCTCTTCTTGATACGACGGTTGCTCTTGTTGTGG
CATCACAATTGGTTGCAGAATTTGAAGTGCACGATTCACGCGGATTATCTCACCTCTTTGGTCATTTTGATTCTG
AAGTCTTTGAGATGTCTGTTCACTTATACCAAGGGCTTCACCAAGCAACTGTAAATTGAATCCATGGAATATGT
TTTGACTCACATACTGTTGTAGTGTCCTACTACCTCCAGCCAACAAAAATTCCTGCAATTTGATCAAGTAATAAA
TATATCAAACAATCTTAGATATTGGTCAAAAAGGCTAAACCTAGTAGATTAGTCGAAAAGACTATCAATCCTAG
AGATTGTTTTAGATACTCTCAATTACCTTTTGTCTAGGTTCAAGCTGGTTGGCATTGTTGTTTATGTCAAAAACA
TAGAGAACTACAATCGGCACTTGGCCATGATTATAGAACCAATGTGCAATGCCAGCTGGCAACGCAACAACAT
CTCCTTGTTTAATGTGATGAACTTTCTGGTGCTCATCTCTAAACTTTTGTCTTTGGCTCTGACCCTCAGATTGCTC

TTGATCAAATTGTCCGAACTGTTGTTGGAAGGTCTCTGGGCAATCAGGGAATGTCAGCCCTGCAGTACCCGTG
CCTACAAGAAAATAAGTTCAATTCAATATTAAATATTCCTGATACTTTTGTAACATTTTTCACATAGAAATCTAAA
GTATATAATCTAAAGCTATAACCACTCTATTAAGTTCATAAACCAACCTTGTAGGATGTATACCAAGGCGGGAG
CGTTGTGGTATCGAGGTAACAAAAGGCCTTGAGGCTCGATGACACGATGAATCAGTGGCGGATCCAGGATTA
AAGCAAGCCCCGGGCCAAAAACTAAGGGCAAAAAAATTGCTAGAAACATCTTAAAAAAACTTTTTTAATACTTC
CTCTTTTAATACTTCCTCTGTCCCATATTAATTGACTCAAATTTGTCTAAATTTAAATGAATCTAGACACTATTTA
GTATATAGATTCATTCAAATTTTGCAAATCAAAATCAATTAATATGGATCGGAGGGAGTAGCACAAAATTTTAG
TATAATCGCAAAGGTGAACAATTATATATATATATATCCAGATTCATTGTCAACAGTTCAAGAACGCACATAAG
AGATGACTATAAAAACTAAAAAGATATTGTGGAGATAAAAAAAGATAGGGCCAAATCAATGATTTACCTCGTC
TGAGTGTTCCAAGATATTGCCAAATCAATGGTCCAGTTCACCGAGTCTTCCATGACTTCATAGTCAGTAGAAGA
ATCAATACTTTCTAGAAAGAAAAAAATATTTGATAACAGAATGCAAATCTCTTCATCAAGTGTTCCTGCAACATT
GAATTGATAACAAATAACTGTGAAATCAACAAATCGGTTCAATTACAAATAAATGAAATCACCGAGTGTATCCA
TTTGAAGGGATGAAATCAGGGAAAATTTACCTAGTTGTGTAATTAGTCTTGTGAGAGAAGCCGAGCAGACAGC
AGATGACGAGAGGAGGCAGAGCCGCTGGCCTGGAGCAATCGGGCGGCGCTAGCTGGCGGTCGCGCAGATGA
GGATTGGAGGAAGAACCACCGAACCGGCAGCGGCCAGGCAGTGGCGTCGCGTCGATAGACAGGCAGGCGCA
CGGCCGCCCAGGGAGAGTCGGTGTCCGGGGAGCGGAGGAGGTCGGCGGACGGACAAAAATTAGCCTGCCA
GCATGGAGCATCGGCGTGGAGCGCTTCTGGCAGGAGCCAGCGACGGCGGACTCCAGGTCTTGGGCGAACGC
GGGCGAGGAGAAGGTTTTCTCACGGTCATGGACTCACGATCGAGCAAGGCAGGGTAGGGAGGCCAATTGGG
CCAGACTTGGGCTATGCCCTAGGACCAACTTTTTTTCCATAGAGAGTAAGCAGGATTTTTCTCCGCGACCCGG
GCCATGGCCCTAGTTGCCCTGGGCCTGGATCCGCCCCTGCGACGAATGACAGATAGACCAGCACAACGGAATT
GGTCATTTTGCTCTTCAAAATACTCAGTGAGGCCCGCTTCGAACCTCACTTGTCGAAGTGGTTCAAATGCTTGTA
GCCTATCGAATGTGCATCCCCTAGCACTTCCTTGTCGAGAGCTTTGCCATGGTTGACTCTGGCCAAATACCTGA
GCCATGGATCCATGGAACAAGAGGAAAATGCAAAGGCAAGATAAGAGCGATGGGAAACTAGTAGTTGCCATT
CTTGTTGTAAGAGATTGAGAAGGTTGGTTGATGGAGGTGTTGTGGGATGTAATGGGTATTTTATAGCTAAGAA
AAGTGGCATACACCTTATGTAAAGACATGGTTTGTTAAGATAGATGTGTGATGACATGAAAATTTGGACGTAA
TAGAGAAAACAAATGGATAAGCATGATTGCAAGCATACCCATAAATGATCTAACAAGTCCGAGTTCTGGTTGA
AAACCTCA

>chr1D:409721798-409723798

AAAAAAAAAAAAAAAAATCCAGGAGATGATGAGTGGTGGGAGTACTTCCCTTGCCCCTTCTGCTACATCGAGG
TGGGGAGGTCCCCTTCCTCTGTCACCACCTCCAGGAGGAGCACTGCTTTGACATGAGAAATGCTGTAAGAAAC
ATCTTCCTTCCTAGATGTGTTATACTAACTGCATTTCCATTTGCAATAGGCAACATCATCAGTTAGGAACAATAG
CTATGATTGTGGTTCAGTTTGTTCTACTCTGATGATTGAATGGATTAACCTCCAGGTGTGCCCGATCTGCGCCG
ACAACCTCGGGACCGACACGGCGGAGCATTTCAGGGACCGACACTCGAACCTACTGAAGGTACAATCACAGTT
TCACCTCTGCCGGCAGTAGTCATGAACACATCTTGCAATTCGCAAGCAGCTCACTGGCAGCATTGCTCGTGCTG
ATCGCAGAGGAGGAAGTCCTCTTCTTCCAAGGGAAGAGGAGACAAGGAGACATGCGAAGACAGACGATGAC
GACGACTCCTACCTCGAGACGACATCCTACATCGTCGGCAAGCCGGTCCATGACCATTCCCCTGACCCCCTGCT
CTCCCATTTCATCTGCATCGTTGCCGCGCCGCCTCCATTCGTCGGTCCAGAGCCTGGCAATGCCGAGGAGGAG
GAGGAGGACCATGCTTCGCCTTCCTTGGATGATCAGAGGTGACTGACTGTGCAGCTGCATTCCTTGGCCTTCCT
GAATCTGAACAATGGTGACTGACCACCCTGAATGCTGATTTCTTCTTGCTTGCAGGATGGAACGGGTTGAGAT
GGATGATGCGTCGCGGCGGGATCTCGAGGAAAGGTTGCGGAGAGCCGAGTTCGTGAAGCAGATGCTGATGA
CGACGATAGCCCAAGACTGATTTCCTGAGTGTTGGTTTCTGAAGAATTTGGAATTTGACTAGGACTGTTCAGGA
TCTTGGAAGTGCAGGATAGTGTCAGTTTTGCCAAGTCTGACATTTGGCACAAGTAGTGTGAGCTTGGATACCT
GAACGGTCAATCTTAATCATTTAAACTAAACTTTGGCTATCTTCATGTTTCTTGCCAGGATAACAGTTTCTAGGG
CCGTTTTCTCATAATTAACATTGCTTGTTTTGGCACCAAAAGAATATCAATAAGACAAGAACATAAATTGTCTCG
GCTGGCAACAAATAAGAAGCATCAGGCAAGTATGTAATATAACACAACAGAAGCAAAAGTAAAGGTGTACTA
AAATTCTATTTGATAAAAAAAAATTGGTGTATATTCAGTATGAAAAATTGCCGCAGACGGGCTATACAAAGTATG
CATTCCTTACAGTTGGCCAAATTTTTTTAAATAATACATTTTTTTTATTGAATTTCACAAATAATACACAAAACGG

GAAAATTGCAAATCTGTTGGTCGGTCGGCCGTTCAACAGACGAAACACTGAACGGACGACACGCAGCCTTCGT
CCGTTCAACGGACGACACACTCGTCCGTTGAACGGACGAAGGCTGCGTGTCGTCCGTTGAACGGATGAAGGC
AGTGTTTCGTCCGTTGAACGGACGAAACGAGTCTGTCGTCTGTCGAACGGCCGACCGACCAACAGATTTGCAA
TTTGTCCGTTTTGTGTATTATTTGTGCAATTCAATAAAAAAAATGTATTATTTAAAAAAAATTACCAAACATGAA
ACCCAGACAGCCATACTACTGCTTTCAGGGTTAGCAATTGGACTTCTTGATTCCAAGGAGACATCAGAAGTCCA
AAATCCAAGATCACACAGGTTTAGGCAGACTATACTGCTGTATCCGTGCCCTGTGCCTCATGCCATGACCATGA
GGGCTTAGCCAACCACATGTCTGGCAACAGTAGCTCTCGGCTGCAATCTTCCAAAGGGCACGGCTAATTACCC
AATTCTCATAAATATCCCTTGGTAATTTGTTCTTCTAGTTCATCGAAGTTCCAAGCGTCATTGACCGAATAGTCA
CCTATTGAAGCATA

>chr1D:409729269-409731461

ATGGCAACTACTAGTTTTTCATCAGTGTTGTTTTACTTCTGCATTTTCCTGTTGTGCCATGGATCCATGGCTCAGC
TATTCGGCCAGAGCTTTACTCCATGGCAAAGATCTCGGCAAGGAGGTTTAAGGGGGTGCAGGTTCGATAGGCT
ACAAGCACTTGAGCCAATTCAACAAGTGAGGTCACAAGCAGGCGTCACTCAATACTTTGATGAGAAGAATGAG
CAATTCCGTTGTACCGGGGTATCTGTCATCCGTCGCGTTATTGAGCCCCAAGGACTCTTGTTACCTCAATACCAC
AATGCACCAGGATTGGTATACGTCCTTCAAGGTTCGTGTATAATTGAATATGGTGATTGCAATTTCTATACTAC
ACTTCAAGTTCAGATGTTCCAAATGTTACCTCATTCATAAGTTTTTAATATTGAAACAATTGTGTCTTATTTTAGG
TAGGGGTTTCACCGGATTGACTTTACCTGGATGCCCAGCAACCTTCCAACAACAGTTCCAACCATTTGATCAAG
CTCAGGGTCAAAGCCAAAGCCATCTCAAAGATCAGCACCAAAGAGTTTACCGTTTTAAACAAGGAGATGTTAT
TGCGCTGCCGGCTGGCATTGTACACTGGTGCTACAACGATGGTGATGCGCCGATTGTAGCTCTCTATGTCTTTG
ACGTAAACAACAATGCGAATCAGCTTGAACCTAGACAGAAGGTAACTATACAAATTAATCTACATAGCGATAT
ATAATTCAATATTAACCTAACTAGGATTTTAAAGTATAAATTAGTGGGATATTAAGGAACTTCGTTTTACTTCAT
CAATATGAAATCTCAGGAGTTCTTGTTGGCTGGTAACAACAAGATAGGGCAACAAGTTGGACAAAACATATTC
AGTGGATTCAATATCCAACTTCTTAGTGAGGCTCTTGGTATAAGCCAACAATCAGCACAGAGGATCCAGAGTC
AAAACGACCAAAGGGGTGAGATAATTCGTGTGAATCAAGGCCTTCAGTTCTTGAAGCCAACAGTGTCCCAACA
AGGACTAGTAAAGCAGCAAGCCTATCAACCAATTCGAAGTCAAGAAGGACAATCAACCCAATACCAGGTAGG
GCAATCAACCCAATATCAAGAAGCACAACCAACTCAATATCAGGTTGGACAGTCAGGGGATAAAAGTTTCAAT
GGTTTGGAGGAGAACTTTTGTTCATTGGAGGCAAGGCAGAACATTGAAAACCCCAAGAGTGCTGACACATACA
ACCCACGTGCTGGCAGGATAACACGTCTCCATGGCAATAATTTCCCCATCCTTAACCTCGTGCAAATGAGCGCT
ACAAGAGTAAATCTATACCAGGTATATACCATACTACCTTCAATGCACTATCTTAATATTAGATATTCCAAGCTT
CATAACATCTGATTAATAATATGGCATACAAATAATTGCTATTGCAGAATGCTGTTCTTTCACCATTCTGGAACA
TCAATGCACATAGCGTGGTCTACATGATCCAAGGGCATGCTCGAGTTCAAGTTGTCAATAACAATGGACAGAC
TGTATTCAATGACCGTCTTCGCCACGGGCAGTTGCTAATCGTACCACAACACTACGTTGTTCTCAAGAAGGCGG
AGCGTGAAGGATGCCAGTATATCTCATTCAAGACCAACCCAAACTCCATGGTTAGCCACATCGCAGGAAAGAG
CTCCATCCTTCGTGCCTTTCCCGTGGATGTGCTCGCCAATGCCTACCGTATCTCCAGGCAGGAAGCCCGAAACC
TCAAAAACAACAGGGGGGAAGAGTTTGGTGTATTTGCTCCAAAATTTACCCAAACAGGCTTCCAACCTTATCCA
GAGGATATGGATGAGTCATCTTTGACCAATAAGGCATCAGAGTAAATTAGTGAGTGTGATGAGGATCAATATA
GTGAAATAAAAGCATCGCAAGTGTGTTAGTGGGTGGTATATAACCGCTTATCTTAATAAATAACTTCATCATGT
TATATCCTTTGTTTGTGTCATGTTCTTATTTAATTTTATATTCTCCGACTAATCCCCTTCCCTATCTTTGTCTCTTCT
CTGCCGTTTTGTACCTCATTCTCATTCGTATCGACGAGTCATATGGGCCAACTGGACAAGAATTTGTTTTCCAAG
ACTGTTTTTTGTTGTTTGAGTATTTTTCAGATCCTGCCTATAACTCC

>chr1D:458547990-458549990

GATGGTGATGCACCGTTGTAGTACACCCGTGATACTGCGCTTGTACAGCTCGCCATTGATAGTGGTGAAGGCC
TTTGACCGACGAGATATGCGCCGGGCATCTGTCGGGTTGTCAGGCAGTTCTCCATTTAATAGGAACTGGAGGT
ATGGGCGAGTCCAGGATTCTTCGACTGCCATGACCTGCATTAGGTTGACCATGGCGACCTCGGTGTCCAACTTT
TCGGGTCCTTCATGACCTTCGGGTAGCGTAGCCCCCGAGTGTTCTGCTTCTTCGGGTGATCCTTTGGGGAATTT
CCGCTTGATGGACGGCACGCAGATCTGTTCGAGGAACACACCTGGTGGCACTTTTGTGCAAGTGGAGCCGATG

TTGGCGAGTCTGTCGGCTTCCTCGTTGCTTTCTCTTCCGATGTGTCGAAGCTCACAACCGTCGAATTCTCCTTCG
AGTGTGTTGTAGAGCGCGCGGTACGCGATCATGTTTTCGGCGTGTGCGTCGCACTCATTCATCGTCTGCTGGA
CGACGAGGTTGGAGTCACCATAGATGACCAGGCGTGTTGCGCCGCATATCTTTGCCATCCTCATGCCATGGAT
GAGGGCTTCGTATTCGGCCTCGTTATTGGAGGCTCTGAATTTCATCCGAAGAACGTACTTCATCTTGTCGCCTT
GTGGGGAGGTCAGTATGACCCCTGCTCCTGCCCCAGCTTCTCTCTTGGAGCCGTCGAAGTGCATCGTCCATGAT
TGCGACATGTCCGGGGTATCTGGCAGTTGCAGCTCCATCCAATGTACTAGGAAGTCCGGAAGGACTTGAGATT
TAATTGCGTGTCGCTTCTCGTAGACGATGTCCAAGGGTGATAGTTCGATGCCCCACAGGGCCACGCGTCCTGT
GGCCTCCGGGTTGTTCAGGATGCTGTTCAGGGGTGCCTCATTGACGACCATCACCTGATGCTCCTGGAAGTAG
TGGCTCAGTCGTCGAGCCGTCATGAACATGCCGTAGGCTAGCTTCTGGTAGTGTGGGTACCGCTGTTTTGTTG
GAGTGAGAACCTCGCTGAGGTAGTAAATTGGGCGCTGGACCCCGTGGGTCTTGCCTTCTTCGGGTCGTTCGAC
CACTAGTACGGTGCTGACCACCTGATGTGTGGCTGCTATGTACAGCAGTAGGGGCTCGCGCTCCTTTGGCGTG
ACCAAAACAGGCGAGGTGGAGAGGAGCCGCTTGAGGCTTTCGAATGCATCATCGGCTTCTGGTGTCCACTCG
AACTTGTCTGCTTTGCGCAGCAGCTGGTAGAATGGTAGGGCCTTCTTGCCCATGCGGCTGATGAACCTGCTTAG
CGCCGCTACTCGTCCTGCGAGCTGTTGGACGCCCTTTAGGTTTTTCGGCTTCTCCATCGTCATGATGGCTCTGAT
TTTCTTCGGGTTCACCTCGATCCCTCGTTGAGAGATGAAGTACCCGAGCAGCTGCCCTGTTGGGACGCCGAAG
GCGCATTTGTCGGGATTGAGCTTGATCCTGTAGCGATCAAGATTGTCGAACGTCTCGTGGAGATCGTCGATAA
GTGTCCTTGCCTCCTTGGTTTTTATGACTATGTCGTCGACATAGACTTGGACGTTGCGACCGATCTGGTCCTTGA
GGCAGGCCTGCATGCATCGCTGGTACGTGGCACTTGCGTTCTTTAGCCCGAAGGGCATTGTATTGTAGCAGAA
TACTCCAAACGGTGTGATAAACGCCGTTTTCTCTTGATCTTCGACCTTGAGTCGAATCTGGTTATAGCCTGAGTA
GGCGTCGAGGAAGGAGAGTTTCTCACAAACTGCAGTCGTATCGATGATCTGATCGATCCGAGGAAGAGGGAA
GTGGTCCTTTGGGCAATGCTTGTTTAGGGACGTAAAGTCCACGCACATCCACAGGATGTCCGTGTCCTTTTTG
GGACCATGACTGTGTTTGCCAGCCACTCGCTTTCTTTGATTTCCTGGATGAAGTCTGCTGCGAGTAATCTGTTG
ATTTCCTTGCCGATGG

>chr1D:466516089-466520035

ATGGCGTCCAACTCCAAGTGCCATCTTCTCCTCTTGGCCGCCCTCCTGGTCTCTGTCTTTGCCGCTGCCGCCGCC
ACGGGTGATTACTGCTACCCATCGATGGGTCTTCCGAGCCGTCCGCTCGATGGCTGCCGTGAGTATGTGGCAC
AACAAACATGCGGTGCCCGTATCCTCGGGGCGCCGTCCGCCCCCATCGAAAAGTTGATGTATCAGTGTTGCCT
GGAGTTTTCACAGATTCGACAGCATTGTCGCTGCGAGGCGCTGCGCTACTTGATGGGGCCTCATCCAGAAAGA
AGTGGCCTCATGAACCTGCCAGGATGCCCCATTGAGGCGCAGAGGGATTTTGCCAGAATACTCCCCACGCCTA
GGCAGTGCAACTTGGTGACAGATTACAGCACAAGATACTGCTTGGAAATGGACAAGTTCTGGCAGTAGTAGA
GATCGCTCATGAATAAGCATGATGCATCCATGGATGTGTGTGACAAGCATATAAGTGCATATGTGAGCTTCGT
TCACCATATGCTATGATTTAGAATAAAGAGAATCATTTTATGGTTCTTAAATTCCAACTCTACCATTTATTCGTTT
GTTTTGTTCTTGTATAGCCTTATTGTCGATTGATTGGTGTTGCTTATCACGTGTATCACGTGCCTTATTGTTATGT
TTATATGTCTGGACATTATACATCAAAAATCACTCTCGAGCTCGTCCTACAGGGAGCCATTATTCAAAATAAAA
ATTAGAAATTCGATTCCAAAGTTTTAGAAAAATCTGAAAAAATATTTGGACGTATCCAACTATGTATTCGGCCA
TTGTGTAAACTAACAGATCGAAATGCTTCGTATTTAAGCTCAGAAAAAATGAAAAAAAATCACAAAACGACAA
AAAATACACTTGCTTTCATCCAAGACAAAATCTTGTTTTTTCCATTTTTTTTGAGCCCAGAATACAATGTATTTG
TATAGAGATTCTACACACTGATAGACTAGAGTATTGAGTATTCCAGCGGACGCTCTGATGGCGTGGTGGCTAG
ACTATATTGAGATATTTTTCTAGATTTTCTAAATTTATATTCAATTTTTCAATGTTTAAGAAAACGGGCTACCTGG
AGCCCGTGCTCCAAACAGCCGCTTTCTATTATACATGGACAAGTAGGTAACCATATAGGGACATGCTTCGGTAC
TCCCTTTCTAAAAAGTTGAAAGTTTTTGTGCAAATCAACGGTGGAGAATAACCTTACCCGCCTCGCATAAAAAG
GTATTGTATAGTCTTTGGGCTTCAAATACATATACGAATACCATCATTTGGCCTAAATAGCACGTACATTCTTTC
TAACAAAACCATGGCGGCCTGTAGATTCTAATAATAGGGGACCAATAATTCCAAACTTTTATCACGTTAAAGAT
GATGCCCTCGCCATTTGCTGAGTGTGGCATGCTCCTTCGAAGAACAATGCTTTGCAAAGTTGCTCACACAGTGA
TCACATGGTTGTCTCCACTCCGTGCAAAAGCAAATTCCGTTATTTGTTTAGCTACACTTACACGCATAAACCAGC
TTGACTGAAGTTACACTTCAAGGAACTAATGCCACATCAACCACGAGTAGAATTCCAACTTGCGTTTCCCTCCA
AATTCTGTTAATTCCAACAAACTAAAGAATTACCTTCTAGTTCATCATAGCTGTCATACAACAACGCAAAACAGT

TAGTAATTGTTGCCAGCTATCTGCCTCGATCTCTTGCTTTTCCCAGTGTATATATACAATCAATTGTCGTGCGTC
GACACCAATATTGGAGTGCATAAACAAAGTGCACCAACGAACCAGATCTAGCTAGAACAACATGGCGTCCAAG
TCCAATTGCAGTCTCCTCCTCTTAGCCGCCATCCTCGTCTCCGTTTTCGCCGCTGCTGCTGCCTCCTCCGCGGACA
ATCCCTGCTTCCCAAAAACGGCGCCTACTCTGAGCAGTTTGCTCCAAAGGTGCCGTGACTATGTGGAACAACAA
ACCTGCGGCGTCGAACCCTCCGGTCCGTTTACAATTTCTGTAAGAGAGCAGTACATGGTGAAGGAGCGTTGCT
GCTGGGAGCTTGCCAACATTTCGCAGAAGTGTCGCTGTGAGGCGTTGCGCTACCTTATGAGGAAGACGCCTGC
TAGTCTTCCCTATGAAGTCAGCCTCAGGGGCATGCCCGGATGCCCAAGGGAGGCGCAGATGAACTTTGTCCGA
ATACTCGTCACGCCGGGGCAGTGCAACTTGGCGACCATTTACAACGTTAGGTACTGCCCAGCTTTGGACAAGT
TTCAGTACTAGAGACAAAAATCTGTCTCTCATGAATAAGCAAAGCTTGTTGCATCCACTGACGTGTGACATGCT
TATCTACACATGTGCGCTCAAGGTTCATATGTGGTACTGTATGGTATGATTAGAATAAAGAGAATCATTTTGTG
TTTTTTCTCTTGCGAAATTTTATCGATCTATTAATAATCAGCAGCAACGGTATAAAGAGGCATGCAAGTAATATA
AATTACAAATAAGTTATTAGACCACCTAACGACGACTATAAACATTAGAGCGAGTCGAATGCTCACCGTCGTCC
CAGGATAAAGAGAGTCATTTTGTGGTTCTTGAACTGCAACTGAATCTTTTATACTCCCAGGATTCAGAAATATG
TGTCGAAGATTTGTCGATACTTATTTCCGAACAGAGAAAATACAAATGTTTGATGGTCTTATAGCCTCATTTTTA
TCTATGTATACCGTTGGGAAACTGGGTTGGTAGAGGCCCACTCGGTGTGGCCTTTACCAGGCTAGGGTTGGCC
CATGTGGGTATCGTATCCCCCTTAGTCTATAAATAGAGGAACAATCTTTGTACGTGAGTTTACCGAGAGAAAGT
TAACAAAAGTTCTCCTTTGTTCCCTGTGTCATCTACCTGTGTACTTTGTGTGCCTTCTACTAGTCTACTTCTCGGG
TGTGGAACAGCCTCAAGACCGGCCAGTCGGCAGGGGGTGTTCCAACACGTTATCAGCACGTATCTCTCCGTCG
AGCCTCCGTCAAGCCACCGTCGAGCCTGCATCACGCAGGCTTTCATTGAAGGTAACCCAAATCTGACCGCAAA
AATGAATTCATCTCGCGATTTCCTTTCAAGATTTGCGTTCAGATCTTAGATCAGACCCCATCTGGGATACAGATT
TTTCTTTCCGGAAGTCGAGCTGACCTGCTGTTGCCGACCAATGTCGATTTAATCACTTCCGGATCAGAAACTTGT
TGACTGTATCAGTGCACCTGTTGTGCCGATCTAATTTTCGTTTAGTAGATTGGACTGATCTTTGCCGTTTTAAAT
TAGTAGATCGGATCGATCTACCGTTTTGGCCGCATTTTTTTACTGCATGCAAAACGCCATGACTGGGCTGCCGC
CGCGCCTGCGCTGCTGCACGCCTCCGTCGCGCCGCAGCCGCCGCGCCTGCGCTGCTGCACGCCTCCGCCGCTG
CGAGCCCCGCTGGCTCCGCCCCTGGCCATGCCTGGCCCGGCCGCCGCCTCGTGGCCTCGCTCACGCGCGCCCG
CGTGCCCGCCACGCTGGCCTGGCCACGCCATCGCCGTGCCGCTCGCCTAGCCACGCTGGCCACGCCGCTGCGC
CCGGCCGACGCGCGTGCCACGCTGCACAGCTGCGCCCGCCGCCGCGCCCCGCCTGGCCGCCCGCCGCCTGCGC
CGCCGCGCCCCTGGCCCTGCTGGCCACGCCGCTGCGCCCGGCCGACGCGCGCGCCATACCGCGCAGCTGCGCC
CGCCGCCGCCCACCGCCTACGCCACCGCGCCCCTGGCCATGCCGCGCAGCTGCGCCCGCCGCCGCGCCCCATC
GGGCCGCCGCGCGCTCGCTGCCGCGTGCATGCAGCAGCTCTAGTTAAGGGTGGGCCAGGCGGTTGGCCCGCA
CGTGTAGTCCATCTTCTTTGGGCCGTT

>chr1D:466529685-466531685

TGGCGTCCAACTCCAGTAGAAATCTCCTCCTCCTCTTCGCCGCCGTTCTGCTCTCTGTCTTTGCCGCCGCCGCCG
CCACCCGCGGTGACTGCTTCCAAGGGATGATGGGTAGTCCGAGCGATCTGATCCCAAGCTGCCGTGACTACGT
GGAACAAGAGACCTGCGGCTTCGATACCGAAGGGCCACCATTCATGGCCAGGGAGGATTGCTGCAAGCAGCT
TGCAGCAATCCCGCAGCGCTGCCGGTGCGAGGCGTTGCGCTACTTCATAGGGCGGAGGTCTCGTCCTGATCAG
ATCGGCGGCCTCATCGACCTGCCTGGATGCCCCAGGGAGCCGCAGAGGGACTTCGCCAGAATACTCGTCACGC
CGGGGCAATGCAACTTGGCCACCATTCACAACGCTCCGTACTGTCTCGCGATGGACGAGTCTCAGTGGCACTA
GAGATAGATCTCTCTATGGCCCATGAATAAGCATATTGCATCCGTGGATATGTGTGACATGCACAGCTTGTGTC
TGGAAAGCTATCTCTGCTATGAACGAGAGAATAAAGAGAATCATTTTGTGGTTCTTAAATTGCCAATTCATTTTT
ATGCATATGTTTGGGGTTTTGATGTTATCTTATTATAGTTACTGCTTGCGACCCTTAGAAGCCTTTCCTCTATCCA
CTGGTTTGGCATTCTAGATCTGAAGATATTAAATTTTAGGGACAAGATTCTGAAAAATGTTGCCTATATGGTAT
AGGCGTGCCATGTAGGCAGGTCAGACTAATTGGTAGGATCTGTTGTGAAGCACTTATAAACTTTTAGGACCCA
TAATGCATTTTCATACAATTAGGAATTAATTGTCACCCAAATAGTTTTAGTACTTTCCATGCATTTATTCTATTTA
TATATCTAACTTACAATTATAGCTGGACGCGACCGACTCGGAATGGCTTGGGCCGAGCCTAGAAAAAGCTCGA
GCTGAAGCTGGCGAGCCCGAGCCTCCTCCTTGGGGTCGGCACGACCAAGCTACAAACTCGAGCCTGGAGGAG
CCGGCTCGTGAGCCCAACAAGAGCTACAGAGAGATTTTGTCAACTTTAGCCACTGAATTGAACTACCATTGATT

CAAATCTAATTCTAAGAAAGGAGAACATAGAAATTAAAATATAGATGAGCCGATTCAAATGCTCTCCTCATCTT
CCATGTCCACCTGCTACACATCAGTACACAAATCAATATAAGAGGAAAATAGCAGGTTCAAGGAAAGCACGTC
AGTTTACAGGATTTGTTGGGATTTTGCAATACTAAAAATGTTGTCCTTTGCTAAATAACAGGTGATGTATACTCC
TGTTGCATAAAAACAGAGAACGGGGGATGGGAGTGACCTTGCCGAGTTCATGGCTTCAGGCTGCTGCTCGAT
CTACTGCCAATGGACAGGGGGGCGGCCGAGAGGAGATGGGGGAGAGCCAACGAGGCTTAGCGTCGGGGCG
GCGTCATCCTGCTGGCGGCGGCAGTGAGCCCTTTCCCTAGCTTTCTTTGCATGTGTCCCGTGTTCTAAACTCCGA
ACACTCTTTGAACTGAACTCTGAACCCTCTGTGCTCTCTTCTCTGCCCGTAGCGAAGGCAAACGGGCCAAAATC
CAGCCCATATCATCTCCGGCTCTCCCTCTCATCCCTCTATTAAATCGGAAAAAACATTGTGGGCCAACGAGCTG
GACCAAGCCAAGCCAGGCTCGGCCCAAGGTCGCCGTGATCCAGGATCTCTGAATTGGATCGGTCTGGATTTCC
TAGCGGGTCAAGCCCAATTCGATGCGAGCTGAAAAACTCAGGCCCAGGGTCCAAGTATACTTACAATTGATCC
TCCGAGAATGATATGGACCTTTCTTTGGAGGAGATACACTATGGTAGTTATAATTTTGTTGCCTGAATAAATGT
CGTTTGTCCATCTAAGTGCCACTCAGTTTGATACAGAACAAACTTCCTGCACCCCGGGGGCCGTGTCGCTCTCC
AAGAAGGTGGCTC

>chr2A:318585768-318587768

TTTTTTTTTTTTGAGGGTGAAAAGAGCTTTATTAATTAACGAAATAGGGGATTACACTCTAGGCTTAGCAGTTCC
AAAATCATCGGAGGGCTTTGAAGAATCCACTCCAAGTCAATTTGAGAACTTGTAGCCAGTTTAGCTAATTCATG
AGCAGCCCTATTACACACTCTTTTCACTTTCTGAACCTGCACCTCATGAAGTCGTGCAAAGGTTCCTCTGATCTC
ATTTAGAACCGGCATCCACGATGCCCGGTTATCTTCCTTCCCTTGTAACGCCGCGATGATCTCCGCGTTGTCCGA
CTCTAGAATTATGGGCATCTGGACTCGATCCGCTGCCGCCTGCGCACCGTACAAACAGGCCATCGATTCAGCTT
CAATGGCCGAGGCACACTTTGCAATGTGTCTCGATGATGCATATAAGACTAGGCCCCTGCAGTCTCTTGCGATC
ACTCCAACGCTCGCCATCCCAGAGACAGGGTTGAAAGCAGCATCAACGTTGATCTTTGCCCAGTACTCCGGTG
GCGCCGTCCAATGCTCCTTTACCGTAGCCCCCGGCATAGGCGTGACTGGTTCCGGGCATGCAAGCAGTGGCCC
TTTCCCCTTATCCCTTGCACTGGCATACGTTGGGTATATGGAACCAGCTTAGCTTACTAAGAAGGTCACCGAAT
CTGCAATTGTTGCCTCCCCTTTGTCATGGATCATATTGTTGCGCAGATGCCAAGACCTCCATAATAGCATTAAAA
CATGTGCTTTCATGCCGCTATGGGTTGAATCAAGCAATACCAGGAGCCAGTTCTGGCCTGAAAAGCTGAATTT
GCTTTCATCAGGCAATAACCAGACCTTGCGCATGGCTTCACGGAGGGCTCGTGCTCTAGTGCACCGAACAATA
GCATGGTGACTGTCCACATACACGGCATATGCCACTATCTTCAAGCTTCCTTTTGCATTTGTTTTTCCATGTTGG
GAGACTTTCATGAGCTACCCTCCAACCGAAGATACGTATTTTCGGTTGTTCCTCCGTATTCCATAAACTTTGCCA
GATTTTCCTCTCTCCATCAGGAGCCGAACTGGTAGCTTGTTGCCTCACTCCCAGATCAGAAAGAGACCATGCCA
GCTTATACGCACTTTTAACTGTAAAAAGCCCGTTCTTCTCACAATGCCACGCTGGGATGTCCTCTGTATCCCGCT
GAGGAATCCGGATCTTCAAAGCAGCCTCTGCATCATCATCATGTAGCAGATGTCTGACTCTCTGCTTGTCCCATT
GCTTTTCTCCCGTAATCATTAGTTCATTGACATACGTCAGTCTATTAAACTTTCTCTTCTGAATCGGCTTCGCATG
TGGATCTCGGGGGATCCAATTGTGTCGCCATATCTTGATTAATTCCCCGTTTCCGACCCTCCATATAACTCCTTTC
TTCAAAAGCTCCAGCCCATGCTCCACTCCTTTCCATGATGCTGAACTGTCCGAAGGAAACGCTATATCCAACAG
GGAGCCATTAGGAAAATATTTAGCCTTTAAAACCCTCCCACAAAAACTATCAGGATACATAATCAGCCTCCATG
CTTGGCGGGCTAGCAATGCCTGATTGAAGCATGCCATATCTCGAAAACCTAATCCACCCAGTGGACCTTTCTTG
GGGCACGTAAGTTTTTCCCAAGCAACCCAGTGGACCTTTCTTCTATTCTTCTCGTCACCCCACCAAAAATCTCTA
ACCATTCTCATGTAATCTTCACAAAATTGAGCTGGCATTTTGAACACACCCATTACATAGGTGGGAATGGCTTG
TGCCACGCTCTTTATCAACACATCTTTTGCACCTGAGGAGCAGTATTTTTCAGACCAATCGGTAAGTCTCTTTGT
AAACCTCTCCTTGATCGGTTGAAACCTTTCTGCCTTCATCCGTCCTTCCGGGGTCGGGAGCCCCAGATATTTCTC
TTCAAAATGATATGCTCCACCTCCAGCTCATTCTTTATGCTTAGCCGGATATCCTCTGAACAGAA

>chr2A:318604973-318607498

TTGCGAATGTAAATGGAGTGAAAATTTCCCATGACAGATGCTCATGAAACATCACAAACTTTTATTGATATCTT
ATTGAGATGAACAAGGGTACACAGTTTGTTGATGTCTATTTTACTCTGCTAGCTTTTCGTCCCATTAGCTCATTC
ACTTTGATGCCTTATCAATCTAAGACAACTCAGATCTGTCTTGAGGATAACTCTGGAAGCCACTTTGAGAAAAC
TTAGGAGTGAATGCGCCCAACTCCTCTCCTCTATTGTATTTGAGGTTTCTGGCTTCTTGCCTGGAGATGCGGTAC

GCATTGGCGATGACATCAACAGGCAGGGCACGGAGGATTGAGTTCTTTCCTGCGATTTGACTAACCATGGGGT
TTGCGTTGGTCTTGAATGAAATGTACTGGCATCCTTCACGTTGTGCCTTCTTTAGGACAACATAGTTTTGTGGTA
TGATTAATAGTTGCCCCGGGCGAAGAATGCCATTGAACACATTTTGACCATGATTATTAACAACCTGAACCGAT
GCATGGCCTTGGATCATGTACACCACACTGTGAGCATTAATGTTCCAGAATGGTGAAAGAATAGCATTCTGCA
ATTAATCACAATGGTGGGTTAATAAATGGAAAAATATTAGAGTTCTATTTTGAAGCCTATAAAATATACCAATA
AGAATGTGTCAAAATGTAACATCATAAGTACCTGATAGAGGTTTACTCTCGTAGCACTCATTTGCACGGTGTTA
AGGATTGGGAACTTTTGGCTGTTGAGATGCGTTATCCTACCAGCACGGGGGTTGTATGTGTCAGCGCGACTGG
GATTCTCAATGTTTTGTGTTGGCTTATGATCGCAAAAGTTCTCCTCCAAACCATTCGAACGGGTAGAAGTTGATT
GCCCAACCTGAGATTGCCATATCTGAGGCTGCCCTATCTGAGGCTGCCCTACCTGAGATTGCACTGCCTCAGGT
TGCTCTCCCTGATACTGAATATGCGGGTAGAACTCCTGCTCTTGTTGTTGCCCCACAATTGGTTGCAGAAATTG
AAGGGCACGATTCACACGGATTATCTCACCTCTTTGGTCATTTTGATTCGAAAGTCTTTGCGATGTGTGTTCACT
TATACCAAGGGCCTCACCAAGCAGCTGGAAATTGAATCCACGGAATATGTTTTGACTAACGAATTGTTGCAGT
GTCCTACTGCCACCAGCCAACAAAAATTCCTACAGTTTGAACAAGTAAAAAATATCAAACAATATTTCAGTTTCA
CTGCATGAACTACACTAAACCTAGTAGATTAGCCAAAAAAAAGTAACAATCTTAGGGGGTGGTTTAGATATAG
TTACCTTTTGTCTAGGTTCAAGCTGATTGGCATTGCTGTTTATGTCGAAAACATAGACTGCTACAATCGGCACTT
GACCATGATTGTAGAACCAATGTGCAATGCCAGCTGGCAGCGCTACAACATCTCCTTGTCTAATAAGATGAACT
TTTTGGTGCTCATCTCTGAACTTTTGGCTTTGACCCTGGGCAGATTGCGCTTGATCAAATTGTCCAAACTGTTGT
TGGAAGGTCTCTGGGCATCCAGGGAATGTCAGACCTGCAAAACCACTACCTACAAGAAAGCAATTTTAATTTG
ATGTTAAGAACTATAGATATTGTTGTAACATTTTTCACATGCAAACTCCTAAGTATATACTCTAAGGATGCAACC
ACTATATTCAATTTATACACCAACCTTGGAGGATGTAGACTAAGCTAGGAGTGTTGTGATATCGAGGTAACAA
GAGGCCTTGAGGCTCAATAACACGACGGATGACAGATAGACCGGCGCAGCGGAATTGCTCATTCTGCTCCTCA
AAATACTCAGTGAGGCCCGCTTCTGACCTCACTTGTCGAAGTGATTCAAATGCTTGTAATCTATCAAATCTGCAT
CCCTGGACACCTCCTTGCCGAGAGCTTTGCCACGGATTATAGCTCTGCCCGAATAGTTGAGCCATGGATCCATG
GCACAACAAGAGAAGGCAAAAGTAAAATAAAAGCGATGGAAAACTAATAGTTGCCATAATTGTTTTGAGATAT
TGGAGAAGCTTGGTTGATGGAAGTGGTGTGGGATGTAAAGGGATTTTTATAGCCAGGAAAAGTAGCCTAGAG
TTCATGTAAGGACAGAGTTTGTTAAGATAAATGAGTAGTGACATGGTCATTTGGACGTCATAGAAAAGTCGGA
TTGGTATAACCAACTCTCAAAATTTAGATGCATAATTTGAGGCACATCCAGAAGATGCTACAATAAGTCCATAT
TGTCTCCGACAATCCTATGTGTCCTTTATTTACAAAAAGATATCCGTGCACACTTGGTTAAACGTTCCTTTTTGGC
ATACAGCTCCAAACTTCTCAAAGAAGGTTGTACAGCTTTTGTCTACAGACCCTTGCTTATTATGACAAGTTAAGT
AATTATGTTTTTTTGACACGTCAAGTAATTATGTTGCTTGTTTCTTATAATCTCTTGTATCTTCTGTGATGTGATC
GGTGGT

>chr2A:347529081-347531081

TGCAGAAGAAGCAGGTCGGTTAAGCACTCCGCTTCGGTTTTAGCCTCCTGTCACTTTGATGCTGAATCAAACCA
TGCAGGCGGCATATAAGTTGAGAGCTGTACTCGGTCATTGCTATTTTTCTCTTGGACTGTTGCCTGTAAAATTCA
ATTGAATCGTGAGTTTTGAGAATTTGCTCCCAGTTGTCTGCCCGAAAACATGTAAGCAGATCTGTGTAATCTCT
GATATTGAAAGCATGTAAGCAAATCCCGTTGATTCGACGTGTTGAACGGACCGGACCGTGTTGTGCCGTGGCA
AATCTGTGTAATCTCCGTATTGGTGGCGTGTAAGCAATGGATCCCCATTGATTCCCTATCCCTGTACTGTTCCGT
GGCCAGCTTGATGATGCCCCTTTTCCTGTGCACGATCGATCCTACCTTCCTCCACCACCGGAAAAGGTTTGATG
ATATGGTACGTTAAACCCTTTGCTGCTGCATTGCAGTAGTGTGAGATCTTGACGACAAGTACAGCTCCACTACG
CAGCACAAGAGGTCGAGCTATGTCTATGTGGTCACTTCTTTTCTTTCTGCCAGGCTTGACTGACCGCTCCCCGTC
AAGAACGAACGAACGGCTCGCACCCGAGTCTGTCTACTGTCTTGGTGTGTGCAAAGGCAGGCATCTCGATGG
GAACATGCATGTCACACACAGACGCCACCCACCCACCCAGGCAGCTGTTCCCAGCATCGGGCCGGCAATCTGT
ATCTGTACACATACAAATACAAGAACAGTGGCGTCGAGATGGATCGAGCGAGACGTGTGTGGTGTGGTGCAT
GCACACTGGCATCTTCGCCTCAGCAGTGCTGTCTGTCAATCTGTCATTTGAGGAGGAGATCGACCGATCGATCT
TCAGTTCAGGGGATCCGATGGGGGCAGAGCACACGGTATCTGATCAAACCTGGCGAGCTGAATCCATCCATCC
ATCCCCGTCCAGCCACTAGTTGGTCGCCCGAGCTTATCAGAAACCGGCCGGCAGCATCGTAACAAGATCACTG
GCCGCGGTCGTGATTTTCTAAAGCGAGCTAGACGCACGAGACACCGAAAAAGGATAAGAGGAGCCCTATCCC

TATCCTGCCTAGCTTAGCTTATCACCTGATGTCATCTCAGAGACTTTGAATCACAGACAGGGATTAATTAGGCCT
GATCGGGCTAACAGACAGGGAGATCCCGGAGTGCTGCCCAACCGCGCGATCTCCCCCGCCAGGAATAACA
AACAAACAAACAGGCTGCTAGTAGTACGACAGGTAGTGAGTGGAGGCTCGCTCGGTGGGCGACGGGCGGCC
TGCGGTGGCGCCTCTCGATCGCTTTCTCGATCGGCCGAGATCGCCGTCGATCTAGCCGGACGTCGTACGCGTG
CAGGCAAGATCTATCTGTATCTTCTAGGAGCAGTGTGTCTTATCTTCTTCCCTCGATCGATCACCAGGGACGAC
CGGCCGGATCGGTTAGCAGAGCTTCTTGGGTTGGCTAGCACGGCATCATCCGTCCATCTTTTCTGGAGCGTTTG
CACGACGTACGGTCGTGGTCTTTATTTTTAGCGGCGAGGTGTGTGGCTTAGCTTCCCCTGGCTCTCGGTCGTCG
GTCAACACGGCGGTGACAGTCACTTGGTAGAACCCGAGACCGAGAGGCGGGAGCTACTGCGTCTTTCGTGCA
GCGAATCATGATGGCTTTTGACAGACGCGCGGAGTACGTACTGGCTAGAGTAGCATGCTTTCATTTGTGGGAG
ATCTTACCGGCCAGGTCAGGATCGCTGGAAAAAGGACGACGGAGCTAGATGAGGCTACTCGTCGTGGGAAAT
GCAAGTGCATCTCCGCGGGGTTGACCTATTCTGTCGTCTCAAACGGTCCGTTTATGTCTATTTGGATCGGACCA
CGGTTGATTTTGAGAGGGGGTTTCGCGTGTCCGGTCAGCCGCGTTCAGCGGCGCGACCCATTTGACCGTACTT
GCCCAGCGCTGGATAAAGAT

>chr2A:355580515-355582515

GCTACCGAGAGAACGAACCGCAGACCATTGCTCGATGGAAACGAGGAGGCACAAACATCCGGATGATCTGTG
GAGTCATCAAGCACGATTCTTTTTTCTTTCTTTCTTCTTAGCCGGATTATACTTCGGTGGTGCCAGCAACATGTG
AAATGTAAATTTGCGTGTCCAATGGCTGTACCTGTATATACAGAAAAAAAGAAGAGAATCCTTTGCTTCTTTGA
TCTTCTTGGCTTTTGTGTACATACTACAGATACTGGAACAGAGTGGCAGAATCCCTGTATTGACGTTGTAGCTT
GTTTGATTAGGAAGGGGTAATGTTTCCACATCTCAGCGAAATTGTTTCCGATATCTGAAGCTAAGATTGGCTGT
CCGATCTCTCTAGCGGTCGCCAACTATCATCTGATCTCTCATCTATTCCCTAGATACACTAACTTTTCCGTCCCAT
AATGCAAGTTTATCGTAACAAACACATACAGTACCAATAAATAAGTTGAGCTCCAAGGGTGTAAATGCACATG
GAATTGAGCAACCAGAGCCATGGTCCCAATCATTTCGACACTGTTGGTACGTCTGGTAGGTCAGCGGAATAAA
AACCGGTGGCTCAGTTGCCGACTAGAGTGTGGCGTTGTGCACTGCACAAGGAGGGACGATCATGACGCGGAA
CCACCAGTGTGGTCCTCCAGGCTGGGCGCAGCTGCAGCTGCAGCATGTCGCGTCTGGCGAGCCGTCAATCCTC
CCGGTGATCCAGTTGCTGCTCCTCGCGCCCGGCTCCACCAACGCATGTTCGCGAGCAGCACCGCGCGATCGTA
CATGGGATCGTGTACGTACAGCTGCCTACATTGTCAGCAAGTCCACTTCTACTGCGTACATTGTCAACAAGTAA
GTCCACTACTAGGCTACTCCCAAAGTCCCAACTTGGACTACTCCACACGCCTTGTGCATTTTTCTGATAAATCCG
CCCAATAGACAAAACTAAGAGCATCTTCAGCCAACCCAAAAGAGACAAGAGGACTTTTTTAGCGCCGATGAAA
CGTTTTTCTTCCAATTCCTCATGAGCTGAAATTCGTCGGGTTTAGCCGAATTTTCATCCAGCGCACCCAGGCCAA
ACCCAGCACGCCGAGGGCCATCGGGGGCACCGGGGGAAGGAAAAGGACGAGTGGGCTAGCTCTGTCGGCG
ATACAACTGATACTCCCGTCTAGATTTGCCTTTTCAAGCCACCTCTATCCCTCTTCTTCCCTCGCCGCCATTCCTTC
GCCGGTACCACCCCTCCCCACTCCTCCCAAAGATCACCGTTAGCAAGGACACTCTTCACCGCACCAACCTCCATC
TTCCTGTTCAGCGAGTTTTCGCGGTGATTCGACGCTGTGGGCAGCTTATGCGGGCAGCTTCTCGTCCAGCACGT
CCGCGAGGTGTTCGGCGTTTTGCCTACTCAATGATGGACTCCTACATCCAGGAGATGTTCGCCATCCGGATAGA
GGAGGAAGCGATCGCTGGTGATCACGAGGTGCCGGCATCATGGGTTGCCTTCCTCGCTATGCGTCAGGAGAT
CCGAGCCCCACCTATGAATCAATAACTGCAGCATGATCTGATGGAGCACCTATGGAGGTGCAAATGCAACACC
TAGTTTGATGTATTTTATTTTGTGTTTCAAAACTTGTTAAATTTATGCCAAACTATGTGCTTTGTTAAACTATTTG
CTAGCTTTGTCAAACTATTTACCGAATTATGCCATTTTTTGTTTTTCCCCACCAAAATCGTCGGCTGGGGGATGA
AATTGGAGGCACGGCTAGGATCGGTGCCCCCACGCCGAAAATTTCTGTCAACGCCCTCCTCGCCCTCCCCACCA
AGAGGCCCATTTTAATGCCAACAAATGGTTTGAGGGGGCGACGGCTGGAGATGCTCTAATGCTTCATCTGACT
CCTTCATAAAACTATTTGAAATCTGGCCTCTAGTTTTGACGCCAATTAAAATGGAGCCGAAGTTGGGCAGTTTA
AGGACAC

>chr2C:12105926-12107926

GAACTTAAGATGTAAAATTCTCTATTATCCAAGTTTTTTTTTTTGAACTTAGTGGCTCAGCATGTGCGGAGAAAT
GCAGCAAATGTTCTTTATTCTTGGATGCATGCGACATGCTGAGTTGTGACATTTGTAGCCTAGTAGTACAGGCC
AACGGAGGAAGCGCCCCGCCCTCAGGCAGTACCGAACCGGCACATCACCGGCAGCTGCGCCGCGAACTGCCG

CGCCTTCACCAGCCTCAGCTGGGCTGCCTCTGCCTGCTCCTCGGTGCTGATATGTGGCTCAGCTGATAACCCGT
ACTCTTGTCCTCCCTGCTCCCTCGCGGTCTCGCCGCCGAACTCCTGTCCCTCCACCGGGTGTCGCCGATGTGCCT
TCGGCTTGCGGTACATCCGGTCTTCTCCTTGCTGCTGCTGACCCGTGGACTCGCCTTGGAACCCTTGTCCCTCTT
GCCGCTCCACGGTCTCGCCGGATCCTTCCTCCTGCTGCTGCTGCTCGTACTCCCTCACGAGTCCGCGTATGGCG
GCGTGGCGACACTTGCGCCTGACCCCCAGGAGCTGCTGGCAGCACCGCACATGGAGCCCCGAGCTCCACCGC
ACCGACCCGGCCGGAAGCTCCTGGCCGGCTGCCTTGTACGCCTTGTAGTGCATGAGCTGCTGGCACGCCTCCA
GCGTGCTCTCTTCGAGCTCAAGCTGGCACTGCGTCTGCGTGCTGATAAACGGAGAGGAAGTATCCATGGCCTC
GCCGACGGACACGGCCATGAGGGCGGCGACGACCGCCGCGAGGAGGAGGACCAACTTAGACATGGACTGCT
AATTAATATAAACCTCGCAGTAGCTTTTTGATTTGCTGACAGATTTGTTCTTGGTGATGATGAGATGGCAGAGA
TGGGCATGGTTTATAACAAGGTGAAGGCTCCGAATGATGGTTGTGTGCACGGCGATCGGCGAGGTTGGATTA
GAAGAAACGTGAGGAGAAGAGGATTAGTTCGAAAAGTGCAATGCGTCCAAGAGCTGGAGTAAGGATGATTC
CGGAACTTGCAAACCAGATTTTCGATGGTGAGATGACACTTCACATTACGTCTCAGTACCAGATATGTGAATGC
TCAACTACTCCCTCCGTTCGGAAATATAAGACGGTTTAGCTTTATGAATCGAATATGTATCTAAACGTCTTTTAG
TGTATAGGTTCGTAGAAGTTCGGAGTGAACCTATATACTAAAAACATCTAGATACATTCATTACACAAAGCTAA
AATGTTTTATATTTCTGAAAGGAGGAAGTATAAGTAACAGGCTCACTTAAAATGTTCTAGTCTTGGAAACTACT
ATTAGTAGATAACGCTTCAGTAAGACTTTTGCTAAGGGAAACATTATGTTTACGAAAACTCCTTTCTAGGTTTGT
TGCTGTCTTTTGTTAGCACTCATGCTTTATGATGTGGTGTAGGTGATCTTTGGAGATGTTTGTCTTTTGCGTTTC
GCTGTGACCTTGCAGTCTTAGTTTTTCTTTTGCTACTCATGTAGTGTAAGGTTTCAGAACTCGGTGTTTCTTTTCT
ATTATGAAAGGAAACGGAGGAGTAAAATCCTTTTTGATTAAAAAAAAGGTTTGTTGCTGTCACCACGTTGCTCA
ATTCCAAGACAAATCCACGCTATGACTCAGAATATCAATCCAATGACATGACGCAGAATATCGATCCATGACAT
GGCCAATAATATGTCAAACAGCGCTCGAAAATACGACACATAAAGAAAATCCTTCGGTTTAAGCAATTAAAGA
AAATCCTCTTTTTAGATGAACTAACCCCGACTTTAATTTAATAAAGCCACAACGGCAAATACTATGAAAAAACA
ACTTGGTAAGAAACAAACAGAGGAAAATAGAAAACAAAGGACACCTTGAAATGCCAGGGCCATCGTGCACCA
CCATGGCCAAAACAGCACGGAGGCGATGGTCATCCATGGTCATATTGACATACGTTTCCACCATCAAAGAGTG
CCATTTGCCAAG

>chr2C:455717839-455720339

TTGCGAATGTAAATGGAGTGAAATTTTCCATGACAGATACACATATGAAACATCACAAACTTTTATTGATATCTT
ATTGAGATGAACAAGGGTACACAATTTATTGATGTCTTTATTTTACTCTGCGCGAGATCTGTCTTGAGGATAAC
TCTGGAAGCCACTTTGAGAAAACTTAGGAGTGAATGCGCCCAACTCCTCTCCTCTATTGTATTTGAGGTTTCTG
GCTTCTTGCCTGGAGATGCGGTACGCATTGGCGATGACATCAACTGGCAGGGCACGGAGGATTGAGTTCTTTC
CTGCGATTTGACTAACCATGGAGTTTGCGTTGGTCTTGAATGAAATGTACTGACATCCTTCGCGTTGTGCCTTCT
TTAGGACAACATAGTTTTGTGGTATGATTAATAGTTGCCCCGTGCGAAGAATGCCATTGAACACATTTTGACCA
TGATTATTAACAACCTGAACCGACGCATGGCCTTGGATCATGTACACCACACTGTGAGCATTAATGTTCCAGAA
TGGTGAAAGAATAGCATTCTGAAATTAATCACAATGGTGGGTTAAATAAATGAAAAATATTAGAGTTATATTTT
GAAACCTATAAAATATACCAATAAGAATGTGTCAAAATGTAACATCATAAGTACCTGATAGAGGTTTACTCTCG
TAGCACTCATTTGCACGGTGTTAAGGATTGGGAACTTTTGGCTGTTGAGATGCGTTATCCTACCAGCACGGGG
GTTGTATGTGTCAGCGCGACTGGGGTTCTCAATGTTTTGTGTTGGCTTATGATCACAAAAGTTCTCCTCCAAACC
ATTCCAACGGCCACGAGTTGATTGCCCAACCTGAGATTGCTTTACCTGAGATTGCACTGCCTCAGGTTGGTCTC
CCTGATACTGAATATGCGGGTAGGACTCCTGCTCTTGTTGTTGCCTCACAATTGGTTGCAGAAATTGAAGGGCA
CGATTCACACGGATTATCTCACCTCTTTCGTCATTTTGATTTGAAAGTCTTTGCGATGTGTGTTCACTTATGCCAA
GGGCCTCACCAAGCAACTGGAAATTGAATCCACGGAATATGTTTTGACTAACGAATTGTTGCAGTGTCCTACTG
CCACCAGCCAACAAAAATTCCTACAGTTTGAACAAGTACAAAATATCAAACAATATTTCACTGCATGAACTACA
CTAAACCTAGTAGATTAGCCGAAACAAGTAACAATCTTAGGGGGTGCTTTAGATACTCATAGTTACCTTTTGTC
TGGGTTCAAGCTGATTGGCATTGCTGTTGATGTCGAAAACATAGACTGCTACAATAGGCACTTGACCATGATTG
TAGAACCAATGTGCGATGCCAGCTGGGAGTGCTACAACATCCCCTTGTCTAATAAGATGAACTTTTTGGTGCTC
ATCTCTGAACTTTTGGCTTTGACCCTGGGCAGATTGCACTTGATCAAATTGTCCAAACTGTTGTTGGAAGGTCTC
TGGGCATCCAGGGAATGTCAGACCTGCAAAACCACTACCTACAAGAAAGCAATTTTAATTTGATGTTAAGAACT

ATAGATATTGTTGTAACATTTTTCACGTACAAACTCCTAAGTATTATACTCTAAGGATGCAACCACTATATTCAA
TTTATACACCAACCTTGGAGGATGTAGACTAAGCCGGGAGTGTTGTGATATCGAGGTAACAAGAGGCCTTGAG
GCTCGATAACACGACGGATGACAGATAGACCGGCGCAGCGGAATTGCTCATTCTGCTCCTCAAAATACTCAGT
GAGGCCTGCTTCTGACCTCACTTGTCGAAGTGGTTCAAATGCTTGTAACCTATCAAATCTGCATCCCTGGACAC
CTCCTTGCCGAGAGCTTTGCCACGGATTATAGCTCTGGCCAAATAGTTGAGCCATGGATCCATGGCACAACAA
GAAAAGGCAAAAGTAAAATAAAAGCGATGGAAAACTAATAGTTGCCATAATTGTTTTGAGATATTGGAGAAG
CTTGGTTGATGGAAGTGGTGTGGGATGTAAAGGGATTTTATAGCCAGGAAAAGTAGCCTAGAGTTCATGTAA
AGACAGAGTTTGTTAAGATAAATGAGTAGTGACATGGTCATTTGGACGTCATAGAAAAGTCGGATTGGCATAA
CCAACTCTCAAAAGTTAGATGCATAATTTGAGGCACACGCAGAAGATGCTACTATAAGTCCATATTGTCTCCGA
CAATGCTATGTGTCCTTTATTTACGAAAAAAGATATCCGTGCACACTTGGTTAAACTTCCTTTTTGACATACAGC
TCCAAACTTCTCAAAGAAGTTTGTACAGCTTTTGTCTACAGACCCTTGCTTATTATGACACGTTAAGTAATTATG
TTTTTTTTGACATGTTAAGTAATTATGTTGCTTGAGTTTTCTTATAATCTCTTGTATCTTCTGTGATGTGATCGGT
GGTACTTGTTATGTTTCTTGTTTCTTGTTTGTTTGTTTAGTACACCACTAACTGACA

>chr2D:151253550-151254550

GGCACGTGATCACCGGCCCGGTCAGGTACCCGAGCCCGAGCGGCAGGGAGAACGCGTTCCGCGTCGAGAAG
TACAGCGGCGCCGCGGAGGCGCGCGAGTACAAGCTGATGTCCTGCGGAGGGGACGGGGACTCTTGCCAGGA
TCTGGGAGTGTTCAGGGACGGCAAGGGCGGCGCGTGGTTCTTGGGCGCCACCGAGCCGTACCACGTCGTCGT
GTTCAAGAAGGCGCCGTCCACCGTTTAAGTTTCTGTTTTTTCTTGTAGAAGAGTGTGTGTTCGTAGGGTATAGC
GTGCCTGAGAATAAGGTGGGCCTTGAAGTTGTGTTAGTTCCACGTGAGCACCGGAAAGGCGAGGAAAGGGG
CTTCTTCTGGCCTCTTGGGCGACTGCCTCAGCGCAATGCAAAATAAATTTCGCATCTTGTCTGAATTACTGAAGT
GTGAACTTCTCCATGTCCAGATTACTGAATAGTAAACTTCGTGGAGAGCTAATAAGACTGGAGAAAGTAATGG
GTATAAATTCAAATAATTTTGACAATAACTACCCAAAGTTGCCGAGTAGCGAAATGTTTACCAAGTATAGCACT
CCGTAACTACACAAAATATAAGGAAAATAAGACATGATCATGCTCCTATGTAAAACAAAGAGTTTTGGGAGAA
CTGGCTCAATCCTCTACGGGGTGGCCTATCTCATATATTTAATGACGTACAATGGTATAATACATGTATACACA
GTTGGACTACAATATAAAGTCTAACACCTCTCTCAATCTCAACTCACTCCTAAAACATCTAGAAGGTTGAGATTG
CGCCTACAGACCTCGAACATCGGTGAAGGTAGAGGCTTGGTGAAGATGTCTGCAAGTTGATCTTTTGAAGAGA
TAAACCTGATTTGTAGTAGCTTCTGTGCAACATGTTCCCTCACAAAATAATAGTCAATCTCAATGTGTTTGGTCC
GTGCATGGAACACCGGATTAGATGATAGAAACGTAGCTCCGATGTTGTC

>chr2D:235342321-235344321

TGAACTAAGAAACAGTTTCTTAGAGCTCTGCTGTCTTAACAAGAAACACAAATGGACACTTATTAATGGATACT
ACACGTTTGTTGAGTTTACATGCATAAGAGTACTCAACAGCAAGTGATGTATCCAACTCATTGATGATCTGAAA
GACATCAATGTAGTTCGCCCCAATTTCACATTTCCAATTGGAGCCTGACGACACAATTAATAGGAGTATATAGC
TATAGGCAATGGTTAAACCACCGATGCCATTTTAGGAGTAGTTTGGTTTGTAAAATTTTCAAAATCACATCCTG
GACTTGGCTCCCGCTTTTATTATATAGTGTAGAGCTTTCTTCTTCGTCATTATCATCACATCCTGGACTTGGCTCC
CGGCTTAGTCCAGAACTGGTCCTCAGTAACTCCTGCAAGCATACACCAGACATTTAGAAAAAAACATGCATGAC
CAAAATAACTCTGAAAACACGGGCATGTCCGCAGCAACTTGCCTTTGCCCATGGCTGCGGTTGAGGAGATGCC
GCCCTGCACGGTCTTGAGCACCCTGTCATAGAAGTTTGCGCCCGACCACTTCTGATGCTCCAGCGTCTCCACCC
CGTTGTTCCTCTCCTCCCTCTGGATCCTCTCCACGTAGGCCAGCATGCCGCGCCGGGCGAAGTCCCGCGCGAAG
GTGTCCGTGACGAGCGCGTCGGCGTGGAACCCGGCGAGCGTGATGAACTGCCACACGTAGCCGAGCTTGGCG
ACGTGCGGGATGAATGCCGCCATGTCGCTGTCCGTCATGCCGGACGCGTCCCAGTTGAAGGACGGCGACAGG
TTGTATGCCAGCATCGCCTCCGGGAAAGCCGCCTTGACGCCCTGCGCGAAGGCCGTGCACTCTGGGACGTTGG
GGCTGGAAGTCTCCATCCACAGCACGTCGGCGTGGGGCGCGAAGGCGCGCCCGCGGACCACGGCCGCCGCC
ACGGAGCCCTTGAAGCGGTAGAACCCTTCCCTGGTCCGCGGCAGGTCCCAGTCCCAGAACACGGACGCCACG
CCCAGGCTGGCGGCCACCTCGCGCGCCTGCTCGTTGGACAGGCACTTGTCGTAGCCGCTGGTGGCGTTGCTCC
ACTCCTGGAGCCTGCGCTGCTTCTCCTGCTCGCTGGTGCTGAGGCCCGCGATGGCGTCCTTGACGCAGTCGGA
GAAGGTCTTGAGCTGCGCCGAGGCGGTCCAGTCGTCCTCGATGGCCTGGAGCTCCCTGCCGTTCTTTCCGGCC

GCCATGGTGTCGGAGAGCACGGCGGTGAGGCTCCGGTTCTTGAGGCGCGGGTTGGTGGCGCCGAGGATGAA
CTGGTGGTCGCGCGCGTCGATGTTGGTCTGGATGAGCGTGGCCGCGACGGCGTCGGAGCGGGCGACGAGGA
CGGTCTCGACGCCCATGATGTCGAACTGGAGCCGGGCGGCGACGAGGCGGTTCACGTGCTCCGAGACGGCCA
CCAGCACCTTCCCGGCCATGTGTCCGCACTTCTTGGTCACGGAGGACTGGTCCTCCAGGTGGACCCCCGCGGC
GCCGCGCTCGACGAAGAGCTTGCAGAGCTTGACGGTGGCCGTGGCGCCGCCGAAGCCGGTGTCGCCGTCGGC
GATGATGGGCTTGAGGAAGTCGACGTAGGGCAGCTGGGCCCGGTCCGCGCGTGGCAGGGACATGCGCGCCT
CCTGCTGCTTCCGGTCGTGGTACTGCTGCGCGAAGAAGAGGTGCTCCACCTTGTTGGGCACGGTGTCGTAGGG
GTAGTCGGCGAGGTCCGGCCCCGGCTCGTTGGTGGAGGTGTGCGTGGACGAGCACTGCCACCCGGACACGTA
GATGGTGTCCAGATGCTTCGCCATCATCGTCACCTGCACGCACAAGTCAAGGCACCGCTTTGGCGCGCGGAGA
TAACTAGCGATCGACCTGGTTGCGTCGCGTACGTACCTGGACGGGGTCGAGCGCGCCGAAGGTGCGTGACGC
CGTGCCGCCTGCCTGGTGGGCCTTGAGCGTGCGC

>chr2D:267017325-267019325

AGGAGCTGAATTGGCTTTGTTGTAGGTGCCTTGTAATTCTCGTCTTGTAAATTAAGAACCACAGCTTGCTGATG
CTGCTGTACATTTTAGGACCATCTGATGCTTTTGGGCCAGTCTGGTTTATGTATTTGCTGCTAGCGTACCGTGGT
TCGCAATTTGCCAGGGTTTTAGCTATGGCATTGTGCTTGTAGGTATCTGTAGATGCAAGGACCGATCAGGTTGT
ATAAATGCTAACAATAAGTGCCACCTGGGCCAGCTGACTGAATAGGCTATTATCTCGCCATTTCCTTCTCCTTCA
CGGCTTCATTCGTGTTTAATTTATATTGTTATTGTTTGGTCGATTACGATGATCTCACTCCCTAGAACAAATTCCA
TGCACTGTTCTTTTTTTTAATTTAGTTAGGAGGCGGCTTCTCCGTGCTGCCCCTGCCCCTTCTCCCCTCTAATTTCT
CGCCGCCGCTAGAGGAAGCTGGCGCGCGAAGCCCCTCCCGGTAGATGGCATGACGAACATGGGATCTCGTCT
CCTCGTCCATGGTAGATGGCATGACGAACATGGGATCTCGTCTCCTCGTCCATTCTAGTGACAGGATTCTCTAG
GAGGCGGGCCCTAGATGTCGGCTGTGTGCAAGACGGAGTATGGATCTCGGGATGGCAACTCTTGGGCGACAG
TCGCCTGACTGTCTGTGGCTATGAGGCCGATGATGGTGTTGAGGCTGCGGCGGCCTCCTCTCTTCGGGCCGAT
GGGCCTACAGAGCAGGATCTGGAGTGACTTGTAGCGGCAAGGTGGTGCCTACTGGACGATGTTGCTACGTGT
GTTGGGCCAGGCAAGAACATCAACCAATTTTTTCTTCGCCCTAACGCAACACTGCCAAGATCCAACTGAGGAA
GGCCATAGTTCTTACTCAGACGAGCGGTTGCTGAAGTCAATAAAGAACGTCAGGGTAGTGCAGTCCGCGTGCC
ATACATCTTTCCCACCCGGCTCCGACAAACTCTTCTAAGGTGCCGACTTGCTCTTCGTCCACGATAGCGACAATT
CTGCTGCCTCAAGCACCCCCAAGCCTGGTGCATCTGCTGGTCCTCACACATCCAAGTTGGTGGAACAACGACG
GGGAGGTGCTAGCCTCGGTTCCGCTGCCCTCAAGGAAAAAGCTTAAGGTGGTGGTGCGGAACTAACTTGCGG
GTCAAGCTCTCACATCCCCCGCCAATCCAGCCCTCTATATATGCGGAGTAGGTGAGGGAAAGTGGGTTCAACC
CATTGACCAGTTCCTGTTCGTCTAAGTCTCACTGAGATGGCTCATGTCAGTTGGATCTCATCTGTTTGGCTCCAC
TCTTTTAAGTGTGTGTGATCCCATAGGCTCATGGTAAGCCAGGCACAACTCCGAGTCTCACTCATAACTGATAA
GACGAGAGTGGCTTCTAGCAAAGCATGTTGACTCCCAGTGATGAGTAACCTTCAATGTGATGGTCTGTTCAATT
CCCTTTGTCTCAATAGTGCTTTGTCTTGCGATATTGGTGGCGTCATGCATGTGCCAGATTAACTCTCTTTTCTTGT
TTGCGTGACAATTGATTCCTTCTGGCTAACTCATTAGTCAGACTATCTTGGTTAACATTTAACCAAATAGCGCAT
GACCATGCTTCTAAATCGTATCATACGAGAGGGCCAGAGAGTATCTCTCCGTGGCGGAGGGACAAATCATGTC
TTAGTCGATCCACATCACATGACTTTCTTCTTAGACGACCCAACCACCGCCTTTATAGCAGCCCGATATGAACAT
AATTTGGCAGCCTCAAAGCCGGATAATCCAAAACTTGATCATGCGATCACCTCGTGTCTAAGGATTCCATTGCA
TGAGTCGGATCATATGATGTCTTACTCATGCCAGCACAGTCCTTCGGTCAGTCCGGCTAGCCTAGCTCTTTGGT
ATAACCAATGTAAGTCGGTTTAGCACCGTCTTACACATTAGTCTCGGCAGTGTGTCCAGCACAACCTCAAATGA
CTA

>chr2D:527769102-527771102

TGGCAACTATTAGTTTTCCATCGCTTTTATTTTACTTTTGCCTTCTCTTGTTGTGCCATGGATCCATGGCTCAACT
ATTCGGGCAGAGCTATAATCCGTGGCAAAGCTCTCGGCAAGGAGGTGTCCAGGGATGCAGATTTGATAGATT
ACAAGCATTTGAATCACTTCGACAAGTGAGGTCAGAAGCGGGCCTCACTGAGTATTTTGAGGAGCAGAATGA
GCAATTCCGCTGCGCCGGTCTATCTGTCATCCGTCGTGTTATTTAGCCTCAAGGCCTCTTGTTACCTCGATATCA
CAACACTCCTAGCTTAGTCTACATCTTCCAAGGTTGGTGTATAAATTGAATATAGTGGTTGCATCCTTAGAGTAT

ATACTTAGGAGTTTGCATGTGAAAAATGTTACAACAATATCTATAGTTCTTAACATCAAATTAAAATTGCTTTCT
TGTAGGTAGCGGTTTTGCAGGTCTGACATTCCCTGGATGCCCAGAGACCTTCCAACAACAGTTTGGACAATTTG
ATCAAGCGCAATCTGCCCAGGGTCAAAGCCAAAAGTTCAGAGATGAGCACCAAAAAGTTCATCTTATTAGACA
AGGAGATGTTGTAGCGCTGCCAGCTGGCATTGCACATTGGTTCTACAATCATGGTCAAGTGCCGATTGTGGCA
GTCTATGTTTTCGACATAAACAGCAATGCCAATCAGCTTGAACCTAGACAAAAGGTAACTATATCTAAACCACC
CCCTAAGATTGTTACTTTTTTTTGGCTAATCTACTAGGTTTAGTGTAGTTCATGCAGTGAAACTGAAATATTGTT
TGATATTTTTTACTTGTTCAAACTGTAGGAATTTTTGTTGGCTGGTGGCAGTAGGACACTGCAACAATTCGTTAG
TCAAAACATATTCCGTGGATTCAATTTCCAGCTGCTTGGTGAGGCCCTTGGTATAAGTGAACACACATCGCAAA
GACTTTCGAATCAAAATGACCAAAGAGGTGAGATAATCCGTGTGAATCGTGCCCTTCAATTTCTGCAACCAATT
GTGGGGCAACAACAAGAGCAGGAGTTCTACCCGCATATTCAGTATCAGGGAGAGCAACCTGAGGCAGTGCAA
TCTCAGGTAGGGCAGCCTCAGATAGGGCAGCCTCAGATATGGCAATCTCAGGTTGGGCAATCAACTTCTAGCC
GTTCGAATGGTTTGGAGGAGAACTTTTGCGATCATAAGCCAACACAAAACATTGAGAATCCCAGTCGCGCTGA
CACATACAACCCCCGTGCTGGTAGGATAACGCATCTCAACAGCCAAAAGTTCCCAATCCTTAACACCGTGCAAA
TGAGTGCTACGAGAGTAAACCTCTATCAGGTACTTATGATGTTACATTTTGACACATTCTTATTGGTATATTTTA
TAGGCTTCAAAATAGAACTCTAATATTTTTCCATTTATTAACCCACCATTGTGATTAATTGCAGAATGCTATTCTT
TCACCATTCTGGAACATTAATGCTCACGGTGTGGTGTACATGATCCAAGGCCATGCATCGGTTCAGGTTGTTAA
TAATCATGGTCAAAATGTGTTCAATGGCATTCTTCGCCCGGGGCAACTATTAATCATACCACAAAACTATGTTG
TCCTAAAGAAGGCACAACGTGAAGGATGCCAGTACATTTCATTCAAGACCAACGCAAACCCCATGGTTAGTCA
AATCGCAGGAAAGAACTCAATCCTCCGTGCCCTGCCTGTTGATGTCATCGCCAATGCGTACCGCATCTCCAGGC
AAGAAGCCAGAAACCTCAAATACAATAGAGGAGAGGAGTTGGGCGCATTCACTCCTAAGTTTTCTCAAAGTGG
CTTCCAGAGTTATCCTCAAGACAGATCTGAGTTGTCTTAGATTGATAAGGCATCAAAGTGAATGAGCTAATGG
GACGAAAAGCTAGCAGAGTAAAATAAAGACATCAACAAACTGTGTACCCTTGTTCATCTCAATAAGATATCAAT
AAAAG

>chr2D:59487361-59488361

TTTGAATGACTACTTTATTATTCTAGATTTTTCCAATCTGAAGAACAGAATTCAGCATCTTTCCGGCATCAGATCC
CAAATATTTTCACGATGACAAAACACAGAAAAACTGGCAAGATGGATCAGACAAACGGACATCTCACCAAACT
AAGCGCAGTCTCTTGAATTATTTGCTCGCAGAATCCAGACAGGGAAACGCTGAGAAAACAGGGAAAGATACA
TCGGACACCACAGATCCATCCAACAGGCTTAAGAGTCCCACGGGCAGGAATCAGATCTAGGCGAGCTGGCCG
CAGTCGGCGATGACGACGGGCTTGGAGCAGCGGCCGCTGCTGGATCCGACGGCCTCCACCTTCTTGACGACG
TCGAGGCCCTCGGTGACCTGGCCGAAGACGACGTGCTTGCCGTCGAGCCACTCCGTCTTGATGGTGCAGAGG
AAGAACTGGGAGCCGTTGGTGCCGGGCCCGGCGTTGGCCATGGAGAGGATGCCGGGGCCCGTGTGCTTCTTG
ACGAAGTTCTCGTCGGCGAACTTGGCGCCGTAGATGGACTCGCCGCCGGTGCCGTTCCCGGCCGTGAAGTCGC
CGCCCTGGCACATGAACTGCGGGATGACGCGGTGGAAGGTGCTGCCCTTGTAGTGCAGCGGCTTGCCGCTCTT
GCCGACGCCCTTCTCGCCTGTGCAGAGCGCGCGGAAGTTCTCGGCGGTGCGGGGCACCACGTCGGCGAACAG
CTCCATCACGATGCGGCCGGCCGCGGCGCCGCCGATCGTCACGTCGAAGAACACCTTCGGGTTGGGGGCCAT
CTCGCAGGGATCGTAGGGGGTACTGGTGCTTGGCTTGATTTGGGGGGGATAAAACTAGTTTTTCTCTCTCCGG
CGGCTGTGAGATCTGGCGAGGGGGGAGGTGGGGTTAAATAGGCGGGCGGGGCTGGGTAACCCTAGTTAGGT
CAACGTTAGGGTGCAGGCAGCGCTTGACCTTTTCTCACGGCAGTACTCCCAGCTGAG

>chr2D:79639732-79642167

TAAAAACAAGAAAAAAACAAAGGATATAACATGATGTTCTTTATTAAGATAAGTGGTTATATACCACTCACTTA
CACACTTGTAATACCTTTATTTCACTATATTGGTCCTCACACACTCACTAATTCACTCGGATGCTTTAGTAGTCAA
ATATGACTCATCCTTGTCCTGTGGATCACGTTGGAATCCTGTCTGGGTGAATTTTGGAGTGAATACACCAAACT
CTTCTCCCCTGTTGTTTTTGAGGTTTCGGGCTTCCTGCATGGAAATGAGGTACGCATTGGCGAGGACATCCACA
GACAAGGCACGCAAGATGGAGCTCTTTCCTGCGATGTGGCTAACCATGGAGTTCGGATTGGTCTTGAATGAGA
TATACTGGCATCCTTCACGTTCCGCCTTCTTGAGAACAACGTAGTTTTGTGGTATGATTAGCAACTGCCCTTGGC
GAAGACGGTCATTGAATACATTCTGACCATTGTTATTGACAACTTGAACTCGAGCATGTCCTTGGATCATGTAT

ACCACACTGTGTGCATTGATGTTCCAGAATGGTGAAAGAACGGCATTCTGCAATAGCAATTATTTATATGCCAT
ATTATTAATCAGATGTCAATAAGATAGTATAGTGAAGGTAATATCGTATATACCTGGTATAGATTTACTCTTGTA
GCGCTCATTTGCATGAGGTTAAGGATGGGGAAATTGTTGCCATGGAGACGTGTTATCCTACCAGCACGTGGGT
TGTACGTGTCGGCACGTTTGGGGTTTTCAATGTTCTGCCTTGCCTCCAATGAACAAAAGTTCTCCTCCAAACCAT
TGAAACATTTGTCCGATGACTGCCCTACTTGGTATTGAGTTGATTGTCCTTCTTGATATTGGGTTGATTGCCCTA
CCTGGTATTGGATTGACTGTCCTTCTTGACTTTAAATTGGTTGGTAGGCTTGCCCTTGTTGTGACACCGTGGGCT
TCAAGAACTGAAGGCCTTGACTCACACGAATTATCTCACCTCTTTGGTCATTTTGACTCTGGATCCTCCGTGCTG
CTTGTTGACTTATACCAAGAGTTGCACTAAGAAGTTGGATACTGAATCCACTAAATATGTTTTGTCCAAATTGTT
GCTCTCTCTTGTTATTACCAGCCAACAAGAAATCCTGAGATTTCATAATTATGAAATAAAACAAAGTTCCTTCAT
ATCCCATTAATATATACTTTAAAAATCATAGTTAGGTTACTAAATAATTATATATATTTATGTAGATTAATTTGTA
TAGTTACCTTTTGTCTAGGTTCAAGCTGATTAGCATTGTTGTTTACGTCGAAGACATAGAGAGCTACAATTGGC
GCATCACCATCATTGTAGCACCAATGTACAATGCCAGCCGGCAGCGCAATAACATCTCCCTATTTAAAGTGGTA
AACTCTTTGGTGCTCATCCTTGATATGGCTTTGGCTTTGGCTCTGGGATTGATCAAACGGTTGGAACTGTTGTT
GGAAGGGCCCCGGGCATCCAGGTAAAGTCAACCCCGTGAAACCCCTACCTAAAAAAGACATAATTGTTTCTAC
ATTAAAAACTTATGAATGCGGTAACATTTGGAAAATCTAAACTCGGAAGTGTATTATAGCAATGCAATAACCAT
ATTAAGTTATACACAAACCTTGAAGGATGTACACTAAGCCAGGTGCGTTTGTGGTACTGAGGTAACACGAGTC
CTTGGGGCTCAATAACGCGACGGATGACAGATACCCCGGTACAACGAAATTGCTCATTCTGCTCATCAAAGTA
CTGAGTGACACCCGCTTGTGACCTCACTTGTCGAATTGGTTCAAGAGCTTGTAGCCTGTCGAAACTGCACCCCC
TTAAACCTCCTTGCCTGTAGCTTTACCATGGAGTAAAGCTTTGTCCGAATAGCTGAGCCATGGATCCATGGCAA
AAGAGAAAAATGTAAAAGTAAAACAACACTGATGGAAGTCTAGTAGTTGCCATGATTGTTTGAAGAGATGGTT
GGAGGTGGATTGGTGCTAGTTTCTTAATTGGAAGCTTGGTTTTATAGCCACAAAACGTAGTTTAGGATGCACAT
GGTGACATATATTTTATAATAGAGATAAGAAGATATACTGGTGACTCAGGTGTTGTTTAACTGGATATAAATTT
TATTCAAAAGTATATGTGTTGCTTTCCGCCTACAACTAGCTATTGCTGACTCACGTAGCTAGTACCACATATATT
GGCCATTGTGTGAGTCAGTTCAAACATGTGATATCTATCTTCTATCTACTAATTTCTTCTCGGGTAGGTTCGTCT
ACCAGGATTTCCGTCCGCTTTTTTCTGGACGGTTTTGCCCCTGCTGTTTTAGAGTATTTACCCTTCCTGCCACCAG
TCGTTGGTTCCTCTCCTCCGTTCCTCGCCTCCTGCTCGTCTCCTCGCGACCCAAGCC

>chr3A:22653850-22656081

AAGGGGATCAATCGGAGAAGATAATATTAAATAACACAACACACAATTAAATGATATCACATCATGACGGTTT
TTTTATTTAGATAAGCTGTTACACACCACCAACATACACACTTGTGATGCCTTTATTTCCCTATATTGGACCTCAT
CACACACACAAATTCACTCAGATGCCTTATCATTCAAAGATGATTAGACGTTGGTGTCTTGGTAACTCTGGAAG
CCAGTTTGGCTAAATTTAGGAGTGAATGCACCAAACTCTTCTCCCCGGTTGTTTTTGAGGTTTCGGGCTTCTTGT
CTTGAAATGCGGTATGCATTGGCAAGGACATCCACAGGCAAGGCACGTAGGATGGAGCTCTTTCCCGCGATGT
GACTAACCATGGAGTTTGGGTTAGTCTTGAACGAGATGTACTGGCATCCTTCACGTTCTGCCTTCTTGAGAACA
ACATAGTGTTGTGGTATGATTAGGAGTTGTCTTCGACGAAGAATGTCATTGAATACCGTCTGACCATGGTTATT
GACGACTTGCACCCGGGCATGTCCTTGGATCATGTAGACGACACTGTGCGCATTAATGTTCCAGAATGGTGAA
AGAACGGCATTCTGCAATATCAATTAATTGTATACCATATTATTAATCGAATGTTATGATGATTAGGGAATCTA
ACAGTAATGTAGTGTGTTGAAGGAAGTATCGTCTATACCTGATATAGATTTACTCTTGTAGCACTCATTTGCAC
GAGGTTCAGGATGGGGAAATTCTTGCTATAGAGACGCGTTATCCTGCCAGCACGTGGGTTATACGTGTCGGCA
CGTTTGGGGTTATCGATGTTTTGCCTTGCCTCCAATGAACAAAAGTTCTCCTCCAAACCATTGAAACTCCTGTCC
CATGACTGTCCTGTCTGGTATTGTGTCTGGTATTGAGTTGATTGTCCTTCTTGATATTGAGCTGATTGTCCTACC
TGGTATTGGGTTGATTGTCCTTCTTGACTTTGAATTGGTTGGTAGGATTGTTGTACTAGTTCTTGTTGGGACACA
ATGGGCTTCAAGAATTGAAGGCCTTGACTCACACGGATTATCTCACCTCTTTGGTCGTTTTGACTCTGCATTCTT
TGTGCTGCTTGTTGACTGATACCAAGGGCCTCACTCAGAAGTTGGACACTAAATCCACTAAATATGTTTTGTCC
AAACTGTTGATCTCTCTTGTTGTTACCAGCCAACAAGAACTCCTGAGATTTAATATTGAGGAAATAAATCAAAG
TTCATTAATATCCCACCAAAATATACTTGAAAAAACATAGTTATGTTACTTGTAAACAACAATATATTTTTGTGTC
GATTAATTTGTATAGTTACCTTTTGCCTAGGTTCAAGCTGGTTAGCGTTGTTATTAACATCGAAGACATAGACA
GCTACAATCGGTGCATCACCATCGTTGTAGCACCAGTGTACAATGCCAGCTGGAAGTGCAACAATGTCTCCTTG

TTTGAAGCGATGAACTTTTTGGTGCTCATCCTTAATGTTTTGGCTTTGGCTTTGACCCTGAGCAAACTGGGCTTG
CTCAAATGGTTGAAACTGTTGTTGGAAGGTCGCCGGGCATCCAGGTAAAGTCAACCCTGTGTAACCCCTACCT
AAAAGAAAACACATTGTTTGAATATTAAAAAACTATGAATGTTGTTACATTTGGCACATCTAAAATCATTAGTG
TAGTATAGCAATGAAATTTTCATATTCAATTAGACACCAACCTTGAAGGATGTACACCAAGCCAGGTGTGTTGT
GGTATTGAGGTAATAGGAGGCCTTGGGGCTCGATAACACGACGAATGACAGATACACCGGTACAACGGAATT
GCTCATTCTTCTCATCAAAGTACTCGGTGATGCCCGCTTGTGACCTCACTTGTCGAAGTGGTTCAAACGCTTGG
AGCCTATCAAAGTTGCATCCCCTTAAACCTCCTTGTCGAGAGCTTTGCCAAGGACTAAAGCTCTGTCCAGATAG
TTGAGCTATGGATCCATGGCACAAGAGAGAAATGCAAAAGCAAAACAACACAGATGACAAACTAGTAGTTGC
CATGTTGTTAGAAGAGATTGTAGAGGGTGGATTGCTGCTTGTTTCCTAATTGTTAGTTTGACTTTATAGCCATA
AAAAGCAGTTTAAGGATGAACATGGTGACGTAATTTGAGATAAGAAGATATCTATGGTGACTCGGACCTTTTT
AGAATGTGACT

>chr3A:22659036-22661036

CTGGTTTTCATCCGGAGGTGGAAGACCCAAGAGCAGAGGAGACCGATGAGAGAGCGGTGGTCGTGGCGGGG
CGCGGCCGGGAGCACGGCCGTTTCAAGATTCTCGATAGGGTGATCCCGTGAACTCCTGCTTTGAGCCTCACGT
GGATCAAGGCTACCCTCACCGCCGATGAACCTGCTATCCCGCCTCGACGGCCTTCGCGAGCGAACGACGATGT
AAGTTTTTCTCATTTCCATCATCTTTCCGAAAATCGTTCATGAGTGGCTAAGTTGACGAGATTCATTGTTTTTTAA
ATTGTAGCCTGCTTTCGACGCGACCTACAAGGCCACTTTTGCGGAACACTTGGTGACGCTCGAGACGTGGTCCC
AGACCCGAGCGGCCTACGAGGAGTGGGCAGAGTTACGCGATGATGTAAGTTTCTACCTTTATTTTTTCAAAAT
ATGACAAGTCTCGCATTCCCTAGTTTGAATCTTTATTTCTGCAATGCCTCACATCTAAACTATCTTGCAGCTTTTG
GAGAGGTTTTATGGCACTGGAGAGCGAGCCACTCTACTGCCACGGCCACCTAGGCCAGGGCCGCAGCCAATC
TTCCCATCAAAGGATGAATATGCTTTGACCTTCTACTCAGGGACCCCGGTAAGTTATTTGCCAAACCGGTTGTT
ACAACAAACCTGTTCCTTGGTGGTTGCTAACTCTTCCTCAAACATGTAGGGACCGGGATGCTTCAGCAATCCAC
ACTTTGGTCGTCCTGATGCCTCGCCGATTATCTTGCGCGCGATGACACCGGTTCATACCGGTCGTGCTTCTCCTG
GTGCTTCCGCGGTTGGTGGCGGTGGCGGTGGCGGTGATTCTCCTTCTTCCCATCGTGCTAAGGGTGGCGGTGC
TTCTCCTTCTACCGGTGGTGGTGGTCATGGCGGTACTCCTTCTTCCGGTGCTGGTTCGACAACGGCTACTCGAC
GTCAAGCTTCACCTTTCTTCACGCGGGGAGACATGGGTCTCTCGAGCAGCTCTCCGGAGGTGTAGGGATGCGC
ATGTGTCAGAGGTGTAGGGATGCGCATGTGTCGGAGGTGTAGGGATGCGCATGTGTTAGGATGGCACTTAGA
GATGTATCTATGTAGGGATGCTATGTTGTTTGGATGAGGATGTACCTATGTATGGATGATCTAGTTATGTTCAT
TTGATGATGACGATCTTATGTTTGTGTCGATGTGCATGTGTTGTATACTATGCCTGATGATGATCTTATGACTGC
GGGAATTTGAAATGTTGTATTCTGTGCCTGATGTGCATGAGAGAAATAATGAACAAAATCCCAAAAAGTAAAA
ACTGGCAGGGCCTATGCCGTCGGCGTAGAAATTACCATATGGCACGGTTTGTACTTGGCCCGATGGGGGTGCC
GTCGGCGTCAAGGTGCGCCAGCTGGTAGAGTAGGGCCGACGGCTTTGTCGTCGGCGTAGAGGTAGTCGCAG
GCAGCCACAGGGTGGCACGTGGCAGAGTTGGGCCGACGGCATAGCCATCGACGTATAGTACTCACAGGGCTG
CGCCACAGGGTGGCACGTGGCATCTTAGGCCGACGGCTATACCGTCGGCGTATAGCTTCCAGAGCTAACGGTC
GTTAGCTGCTTCAGGTGGGCCTCGCGGTGCCTAGGCCGACGGCGTGTACATCGTCGGCTACGTATAACCGTCG
GGCAAGTCTATACGCCAACGGGAATCTGTACGACGACGGGATTGTGGCATACGCCTACGGCCTGGTACGCCG
ACGCGACTACGCCGACGGCCACCGTCGGGTCAGCCTACGCCGACGGGTAAGCGTGGCTACGCCGACGGGCCC
TAGCCATCGGCCTAATCGGCGTTTCCTGTAGTGTCTACCTACTAAAGAGCTAAGTTCGCTACATGGAAAGTTAT
ATTTGCCAAAAGACTTGTTGACTGCTGGATTTAATAGAAAAAACTGGCAAATGATAAGTGACTGTAGCGCGCA
TGTACCAATTGCTCACATCCAC

>chr3C:582739882-582741882

CTACATGTTCAAGCCGGCCGAGAGGAACCAGTACTTTGCTCTCGATGATGACGAGGAAGAGGCTGCAGAGAT
GGCACTCCGTGAGGAGGAGTTTGAACTCTAGATTGTTGTGTGGCGATACATGTTGGTGGGGAATGCAGCCCTA
CCTGTTCAATTTCTCCTCCTTGTGATGTTGATCATACTATACAGATGCAAGAATTAGCTTATATTTGTCTATGTAC
TATGGTAAATCTTAACTTATTTTGTGCTCATTTCTTTTAGCTTCAAATGTTGCTGATTAGCTTGTCTTGCATACCA
GGGAAAATTCAGTCAAACTGCTTGTTTGTGTGAAGTCACAGTTTTGCTTCTTGGTGATCAATTGTTCTCTTTCCT

GTATAAGCGAACCATTTCAGTTGTATAGTGAGAACATTGATTAACATTTTGTTGCTGCCTCGTAATATGACAGC
AAGATCTTTTACAGAGCTTTGGTGAAAGGACTTCATGATTATTACTGGGAATTTGTCTGTTCGTTCATGGCCTTC
GATTTGTATGCCTCACTGCCTGCTAAACGTTCTACTTCCTCCGTTTCAAATTATAAAACGTTTTGGTAGACATAT
GTTGTAAAAACTTTTTTTAACATGGAAATTTATTGATTTAGCAGCATTTTACAAAGAGTAGCGTGTACTCAGGC
ACGGTGGAAACGGATGTCACATGAGTTTAGACAAGCTCCATTAGCAGAACTTACACATCTGAAAGATAATTAA
GGCTCCTGGATATGTGGAAGACTCTTGTTGCATCAAATGAAGAACTGGCTGAAATATCTGCAAGCTGGGGCCT
TGTTCCCAGTGACCTTGTGTACGAAGTACATCCTGGGATGCTGCTGCCGTTGCCAAAATGGCATTGTCCGTTAG
AAAGGTAGCACCCTGTGAATTCGAAGCATTTCTGCTGCCTTCGTTGCAAGCATTAAGCCAAAGGATTCTGCTTG
CAATGCTACCTGGGAACAGTGCCTACCATTGAATGAGGACACCTAAACCATGCCGCGTCGGTGAATACAATTG
GTGGGTTGTAAAAATTTGGGAAGGCCCAGTGTAGGATATTGTCAGTAGCAAATAATGGAGTGAGCTATCTTGT
TAGTGTTCCGCTCTCTGAACTATGTTTCTTTTCATTATTTCTGCTTATTACAAGATACTACTCCCTCTGTGTCATAA
TAGATGTCACAAATTTATCAAAATTTATATGTATCTAGACACTGTTTAGTAGTATATAGATACATCCAAATTTAG
ATAAATTTCTGACATTTATTTTAAAATGGAGGGAGTACAATCATGGGTTTCCCCGTGATGCTTTACTACAACCGT
TTGTGTTTAGAATAATGAAGTAGCTCATACAGTCGTTGGTATCCCCGTGATGCTGTGAAGTGGAAGCTGTGGG
ACTACAATGGTTTGTTAGCTGGTCTTTCTGAAAGTGACATTGTAATGCTTTTGACACTTCAGTCTGAACTGTATT
TGAGCTGACCAATGTGGAGCAGACTGACAACAAAGATACTCGAATTCAGGGTCGGCAGAATTGGCTTACCTTC
AGAAGTTTGTAAGCAAAACTCGAATCACATCAATCCATACCGCTCTCTCTTTTTACAGGTCTCGTGGCTTAGAAA
CACTCGTGGCTGTAGTTTTTTTTTTTTTTTGCCCTCTCGTGCTTCCACTGTTACCCGCATTCATTTGCTGCCCCAGC
CAGAGCCCTTCCGCTCCAGGCGCCCGCCCGATTTCAGAAGGGCCTGCAGGCCGCAGAAATGCAGCCTCACACT
GTCTAAGGTAGTACCATGGTCATAATCCAAAATGTCCTCTTGTGAATATTTGGATTATGATCACGTGATATAAA
ATTGATAATCCCATCAATTTGGGCCTAAGGTAGTACTATCATTCCATTTTTAGGGAAGTCACTTAGTAGTTCTGA
ATTTGTGATTTTAGAATCATGGAATATGTATTCAACTAAACTCACAGTACGGATTCATAAAGTTTTAG

>chr3C:621443029-621445029

AAAGCCCTCTCAGGTACCTTGGTTGGGTATCATTGTTGTTCTCTTGTTCGGTGTTTGCCGTTGGCGTTTGATGTT
TATATCTATGTGTTGGTTTGTGTACAGATGCTAGCGGTTTGAGCTTTGACCCTCAGGCCCTCCGCCACGACGAC
GCTTTGGCGGGTGCGCTTTCAGCAGTCGAGGACCGCAACATCGCCGTTCAAGATACGCTCTCCAAGGCCAAGG
GCGTTCTCAGTCGCCTCCGCGAGGAGTTGGTGCCCAAGGCGGAGCTAAACGAGAACATGAACGAGCTGGTGG
AGTCCCTCGTCGGGGGTGTTCCTGCATCCTTCAAGAACTCGCTCCGTGCGGCTGGAGCGTCCATGTCGCTTGCC
ATGGTGCAGGCCCACGGAATAGAAGTTGATCTTGCAGCGGTTTCGAAGGCGATGCCCGTTACCATGGACGGC
CAGCAGGTATTCTTTGCTCCCTTCGCGACTGCCTGTGGCAAATATGGGCCGCATCTGATAAACACGGTTGCGAA
GTACTCTGAGGAGCTGCAACGCGCCAAGAAGGCGGCAAAGGCTGCGGCATCAGGGGCTGGCGGAAAGTCAG
TGGCATCTGACACTGCCACGGACTCGATGAAGCCCCCGAAGCCGTAGAAGTCGAAGAAGCCAATCGCTCCTTA
TTAGCTTGTAATAGTTAACTAGTTGATGTCCTTTGTTAAGAACATGCCTGCTGGCGAGTGTATCTGCTGATACTT
TTGCTAGTCTTGAATGCACTTGAATCTTTTGTCTTTGTGTCGATTTGGTCGTCTTATTTGTTTCTGTCTTGGCTTG
TGGTAGGGTACATCAAGTGATCCTTTGAGGGAAACTCAGGACTCGCCGCCTCTTGATGATACTTTGGCACCGC
CGCGCTCCAGGGTAGATGAGTTGTCGTTGCTTTCGCGACTGCATGACCGCGTGAAGCAGCTGGAGCGTGAGTT
GCCCGTGCTCCGCGGTAACACGGATGTATGGAAGTCAAAGGCGGAGAAAGCCGCCGCCCGAGAGCAGTATCT
GCTTGGCAAGGTGACGCAGCTGGGTGATACATTGCAAATGTATCTATAATTTTTGATGTTCCATGCTTGGTTTT
ATCCACTTTCTTGTGTTTTGTTCCAACTTTGGAGTACTTTTCATATGATTTCTTGGACTAACCTATTAACAAGAGC
CACAGTGACAGCTGCCTGTTTTCTGCTGTTTTTGGTTCCAAGAAAGTCAGAAAATGAATATTCTCGGAATTGGA
CAGGACAAAAGCCAAAATGCCTATTTTCCGGATGTCATCAAGTGCCAGAATACGAGACGAAGAGGGACCAGG
GGGCCACCACACCACCTGGCGGCGCGGCCCAGCCTGGCCTGCGCCAGGGGGTGGTGAGGCCAGCCCTGACAC
CCCCTCGACCTTCCCTTCCGCCTATATATTACCCTCGGGACGAAAACCCTAAGTACCCGATCAAAAATCCACGAA
AGAGTCTATAGCCGCCGTCATCTCCATCCCTAGATCGGGAGGGATCTGAAGCTCTTCCCGGCACCCTGCCGGA
GAGGGGAATTCACCCTGGAGGCCTTCTTCATCACCATGCAAGCCTCCGGTGTGATGCGTGAGTAGTTCATCCTT
GGACTACGGATCCATGTAGTAGCTAGATGGCTGGTTTCTCCTCCTTTGTGCTTTCATGTTTAGATCTTGTGAGCT
CCGTTCATGATCAAGATCATCTACTTGTAATTCATACATGTTGTGTTTGCGGGGATTCGATGGATATTGAGATG

CTATTTCAGATTGATTATTGATTCTATCATGTCTTTGTTATTTGAGATCTTGCATGCTTTCCGTTGTTAGTAGCTA
TCTTGGCCAAGTATATGCTAGCAACTCCAAGAGTTAGTATTTATGCTCGATAGTGGGTTCATGCCTCTGGTAAT
CTGGGGAAGTGACAGAAATCTCTAAGGTACTAGATGTGTTGTTGCTACTAGGGAGAAAACAACAATCCTTTGT
TCATAGAACAA

>chr3D:20931143-20933143

GAAACACAAGTGAGTGTCTTAGACGAAAGTGCAAAACAACCAAAATCATCCAACTTCAGTACAGGCAAAAACT
GACAAAGCATTTGGTTTCTAAGGAACACATACATATCCAGCAACCAGGTGAACTTAGATTAACTCAAGGTCTAA
CGAGAGCTGCGTTCTTCTCTTAGCTAGCATGGGACTTGTTGAGGATGACACCCTCATACTTGACCTGGAACCAC
TTCATGGCGTCCTCCTTGGTGACCCTCTGGTGGATGCCAATACGGGCCTTGCACCTGCGGCGACGGGAAACAC
GGTACCCAGCACGCTCCAGAACAACAAAGAAGTCCATACCATAGATACCAGTAGATGGGTCATACCTGGTAAA
GCAAGAAGGTCAGTTAGTGGTCATTAGAGAATTGCAGAACATCAACAAACAGTGATTGCACACTCATAATGAG
ATGCCAGTGTGCAATCAGCAAACAGCAAGAACCATGCACTGTGGCTAATTGACATGAGAGGCTAGTAACCCAG
CAAGGAACTACAGCAAAAAGTAAAATAAACTTTGTTATATTCACTACTAAACTGAATATGATTAGTTATCCACC
AGGAATCAATATATTGTCAAATATTAACAGAGTGATAAGATACATTTGGTGGACAACTAAAGGGAATTTCATTA
TAACATGTAGCTGCATAATGATGCAGGCCCTACTATTACTATTGCCAGTTACCCATCTTGGTGCTCATGATTCAA
ACAGTTATTAAAACGCATAATTACCAGTCTAAGTTATGTCCAAGCAGTTGTTTAATCTACACATAATTACAAGTC
TCAGTTCTCATGTCCAAACAGTTGCTAAAGCTACTCTAGAGAATTGCAAACCTGGAAGTTAAAGTTACATATAT
GATTTCATCTACTGCCTCTACTCAATTTTGCAAGACTTTTTTGAGAGGTTGTAAAAGACATTTTGGTAGGCTATT
TTCGGCTACCAAAACATCTTATTAAAAGGGAACAAAGGGAGTATTTACAAGGAACAGTATCTTAATAAGACAT
AATCGGTAGAAGCCAATGTGCCAGACACAAAAAGTGCCAGCATCAGATAAACTTACTTCATCCCAAGATCAAT
GTGCTCCTGGATACCGAACCCAAAGCATCCAGTGTCACTGAAGTTCCTCCTGAGCAGCTCGTACTCCTTCACCTT
CAGGCCACTCTCCAGCAGCTGCATCGCCTTCTCACCCCTGATCGTCACATAGCACGCAATCTTCTCATTACGTCG
GATACCGAAAGACCTCACAGTGTACCTAGCTGCAACAAGATAATACCATCGCTGTCAGCACCACCAACATCGA
CCACCATTGGACATGGTCAACGAGAACAGGCTGGATGTAAGGAACTTACCCTTGGAGAACACCGGCGACTGTC
CACTCAGCTGCTCCAGCACCTAACAAAGAAATGAAAACTATGCAGTTAACATCAACGAATTACAGTTAAGGTGT
ATTTCCAAACACAACAAATTCAGACAAATCATTTAAACTGCTGTGTGCCACTCAGATTTTCATTTTCGGACTGAA
ATACCATGCACGGGAAATGCCTATGGTGCCACTCAGAGTACACAAATTAAGGTTAATCTACAGCTATCAGAAA
TGGAGAAGACATGACTGCCTACACCCAAACAACTGTGGTTACATGGTAACAGCATTAGTATCACAAGCTGATG
TATGTCCTACTAGCACACTCCAAAAGGCACTGCACCACAGATAGGATAGGCCAGCGCCAAGCCTGGCTAGATC
ATTGCTGGTTTACAATTCCTACTAAAGCAACTAGATTCAACTAAATTGATACTATACCACAACATAACCAAAGCT
AACAGCCTACATGCTAATATATCTAGACGAAACTAATCTAAATCCCAAACGAGCGAGCGCATCGACGAGAGGG
AGAAGATAAATAGATAGGCATATTTATACCTTGGAGGCGCGGGTGAGGCGATCTCCGCTCTCGCCGACGGAG
ATGTTGAGGA

>chr3D:450843917-450844917

GCAGACAAGAACTACGTTTGTCTTTTATTCCCAAGTTTGGACTAGTACTGGCCGGGGGTCATACATGTTAGTGC
ATGACACTTTATTTGTCACTGTTATATCAATTCATCCATCACCTAACACTGATGGCCCATCTATCTCGTACTAGAG
CTCTTGTAGTCTTCTCAAAAGTCACCGCCCCCACCGAACGGGGTGCTGGAGTAGTACACCGGGACCTGCACATT
GCACATTGCCGGCAGGGTTGAACAAACGCCCTCATCACCTAAACTGAGCTACCTGCTGAATTTGGATGAAGCC
CTTGCTCACCTGTTCTGGCTGAGGTTCGGGGATCTGCGGCAGATGCAAGCTGCATTGTTGCCGCATCACCTGG
CAGCTGCTCTGTCGAAACATCTGCGACCGGAGGAACGACACCATCACCACCAGGCTGCACTGGTGCATGCGGA
ACGCCTGACATGGGTTCACTGTTGTTGAAGAAGTGGCTGGAAAACGGTTGTTATTGCTGCATAAATGGTTATT
GTTGCCTGGGAAATTATTGTTTCCGTGGAAATGGCTGATGTTTTGGAAATGGCTGTTGTGGCTGATATTGCCCG
CTAGGGTCAAACGGCAAGGAGGGCAAGGATGAGGAAGGTCTTCATGGTGGGTTTGAGTTTACTAGGTGCGTG
AAGGATGGAGGATCTTCATGGTGTGGGCCTATTTATAGCTGCTATGGCATGGTTTCTTTTTAGCTTCGCATTTCC
TTAACTTAAAAGTGCTTGGATTCTTATCTAACTGATCATTTGGTATGTTGTGTTTGGTAGCACTACATACTAAAG
AAGTTTTTGGATTGATCATCAAAATTGCTTTACCCTTGCACGTATCATCTGTTGAACGAATTAAACTTGGTACAT

ATTGTCCAGAAACTGTTCAGATAATCTCTAGGTTTTTTTAGGGAAATCTCTAGGTTTTCTTTTTTCTGATTTTATT
CAAAAGTATGAAATAATTCACATGCGTACATGAGGAG

>chr3D:455948149-455950271

AATATAACCAAGTTCTTTCATTCCAATTGATGTCATAACCAAAGTTCTTTCATTCCAATTTTATTTATTTATATCAT
GATGAGGCGGACATATAGGGAACATCAAAGTGCTACACACTTGATGTCTTTATTTTACTTTACTAGATTGTCCT
CCCATTACATCAGTTCACCAATCCGATTTGAGCTCTGGAGAAGCCACTTTGAGAAAACTTAGGAGTGGATACGC
CAAACTCCTCTCCTCGGTTGTATTTGAGGTTTCGGGCTTCTTGCTTGGAGATGTGGTACGCATTGGCTATGACG
TCAATGGGGAGGGCACGGAGGATGGAGTTCTTTCCGGCGATGTGACTAACCATGGAGTTTGCGTTGGTCTTG
AATGAAATGTATTGGCACCCTTCGCGTTGGGCCTTCTTTAGAACAACATAGTTCTGTGGAATGATTAATAGTTG
TCCCGGGCGAAGAATGTTGTTGAACACATTCTGGCCATGATTGTTAACAACCTGAACCCGTGCCTGGCCTTGGA
TCACGTACATCACACTATGAGCATTAATGTTCCACAATGGCGAAAGAATGGCATTCTGCAGTTAATCAAATTGA
GTAAGTAAAAGGAAATAACATTAGACTTCTATTTTTAAGCTTACAAAATATATCGATAACAATGTGTCGAAATG
TAACATCATAAATACCTGATACAGGTTTACTCTTGTAGCACTCATTTGCACGGTGTTAAGAATGGGGAACTTTT
GGCTGTTGACATGTGTTATCCTACCAGCACGTGGGTTGTATGTGTCAGCACGTGTAGGATTTTCAATGTTCAGT
GTTGGCTTTTGATCACAAAAGTTCTCCTCCAAACCATTCCAACCGCTAATAGTTGATTGTTCTACTTGTGATAGC
CTTGTTTGAGACAACTATATTTGAGACTGGCCGACCTGAGGTTGTGTTGCCTCAGATTGCTCTTCTTGGTATGAT
TGTTGCTCTTGTTGTTGCATCACAATTGGTTGCAGAATTTGAAGCGTACGATTCACGCGGATTATCTCACCTCTT
TGGTCATTTTGATTCTGAAGTCTTTGAGATGTGTGTTCACTTATACCAAGGGCTTCACCAAGCAACTGGAAATT
GAATCCAGAATATGTTTTGACTCACATACTGTTGTAGTGTCCTACTACCACCAGCCAACAAAAATTCCTACAATT
TGATCAAGTAAAAAATATATCAAACAATCTTAGAGATTGGTTGAAAAGACTAAACCTAGTAGATCAGTCAAAA
AGACTATCAATCCTAGAGATTGTTTTAGATACTCTCAATTACCTTTTGTCTAGGTTCAAGTTGATTGGCGTTGTT
GTTTATGTCAAAAACATAAAGAGCCACAATCGGCACTTGACCATGATTGTAGAACCAATGTGCAATGCCAGCT
GGCAGCGCAACAACATCTCCTTGTGTAATGCGATGGACTTTCTGGTGCTCATCTCTAAACTTTTGGCTTTGGCTT
TGACCCTCATATTGCGCTTGATCAAATTGTCCAAACTGTTGTTGGAAGGTCTCTGGGCACCTAGGGAATGTCAC
CCCTGCAGAACCAGTACCTACAAGAAAACAAGTTCAATTCGATATTAAAAATTACTAATACTTTTGTAACATTTT
TCACATACAAATCTGAAGTATATAATCTAAAGCTATACCTACTCTATTAAGTTCATACACCAACCTTGAAGGATG
TACACCAAGGCAGGAGCGTTGTGGTATCGAGGTAACAAAAGGCCTTGAGGTTCGATAACACGACGGATGACA
GATATACCGGCACAACGAAATTGGTCATTGTTCTCCTCAAAATACTCAGTGAGGCCTGCTTCGGACCTCACTTG
TCGATGAGGTTCAAGTGCTTGTAGCCTATCAAATGTGCATCCCCTGGCACTTCCTTGTCGAGAGCTTTGCCATG
GTTGACTCTGGCCAAATAGCTGAGCCGTGGATCCATGGAACAAAAGGAAAATGCAAAAGTAAGATAGGAAAC
TAGTAGTTGCCATTCTTGTTGGAAGAGATTGAGAAGGTTGGTTGATGGAGGTGTTGTTGGATGTAAGGGGATT
TTATAGCTAAGAAAAGTGGCCTACACCTTATGTAAAGATATGGTTTGT

>chr3D:456055967-456058271

AGGGGGATAGTTGACATTGTCCGTGATGTTTATGTCGATGTGCCAATGACATGGACAATATGGAGCATAAAAA
ATGGCTAATGAAATTTGCAAATCGGAAAGGAGTTAAAATTTCCAACGACGAAACACATACGGAACAGACGCA
AACTTTTATTGTATTCATATCATGATGAGATGGACATGTAAGGAACATCAAAGTGCTACACACTTTTCTTCATGT
CTTTATTTTACTTTACTAGATTGTCCTCCCATCATATCAGTTCACCAATTCGATTTGAGCTCTCGTGAAGCCACTT
TGAGAAAACTTAGGAGTGAATACGCCAAACTCCTCTCCTCTATTGTATTTGAGGTTTCGGGCTTCTTGCTTCGAT
ATGTGGTATGCACTAGCGATGACATCAACAGGAAGGGCACGAAGGATTGAGTTCTTCCCGGCGATGTGGCTA
ACCATGGAGTTTGCGTTGGTCTTGAATGAAATGTATTGGCACCCTTCTCGTTGTGCCTTCTTTAGAACAACATAG
TTCTGTGGTATGATTAATAGTTGTCCCGGGCGAATAATGTCGTTGAACACATTTTGTCCATGATTGTTAACAACC
TGAACCCGCGCATGGCCTTGGATCATGTACATCACACTGTGAGCATTAATGTTCCAGAATGGCGAAAGAATGG
CGTTCTGCAATTAATCAAATTGAATAGGTAAAAGGTACTAACATTAGACTTCTATTTTTAAGCTTACAAGATATA
TCGATAAGAATGTGTCGAAATGTAACATCATAAGTACCTGATAGAGGTTTACTCTTGTAGCACTCATTTGCACG
GTGTTAAGAATGGGGAACTTTTGGTTGTTGACATGTGTTATCCTACCAGCACGTGGGTTGTATGTGTCAGCAC
GTGTAGGATTTTCGATATTCATGGTTGGCTTATGATTGCAAAAGTTCTCCTCCAAACCATTCCAACCGCCACTAG

TTGATTGCTCTACCTGAGATTGCCATGTCTGAGACGGCTCTATTTGAGATTGACCTACCTGAGGTTGTGTTGCCT
CAGATTGGTCTTCCTGATAGGATTGTTGCTCTTGTTGTTGCATCACAATTGGTTGTAGAAATTGAAGCGCACGG
TTTACACGGATTATCTCACCTCTTTGGTCACTTTGATTCTGAAGTTTTTGAGATGTGTGCTCACTTATACCAAGG
GCTTCACCAAGCAATTGGATATTGAATCCACGGAATATATTTTGACTCACATACTGTTGTAATGTCCTACTACCA
CCAGCCAATAAAAATTCCTACAATTTGATCAAGTAAAAAATAATATTAAACAATCTCAAAGATTTGTTGAAATA
GTAACCTAGTAGATTAGTCGAAAAGACTATTAATGTTAGACATTGTTTTAGATACCCTCAATTACCTTTTGTCTA
GGTTCAAGCTGATTGGCGTTGTTGTTTATGTCAAAAACATAGAGAGCTACAATCGGCACTTGGCCATGATTGTA
GAACCAATGCGCAATGCCAGCTGGTAGCGCAACAACATCTCCTTGTGTAATGCGGTGAACTTTTTGGTGCTCAT
CTCTAAACTTTTGGCTTTGGCTTTGACCCTCATATTGCGCTTGATCAAATTGTCCAAACTGTTGTTGGAAGGTCT
CTGGGCATCCAGGGATTGCCACCCCTGCAAAACCAGTACCTACAAGAAACAAGTTCAATTCGATATTAAAAATT
ACTAACACTTTTATAACATTTTTCACATACAAATCCAATGTATATAATCTAAAGCTATAACTACTCTATTAAGTTC
ATACACCAACCTTGAAGGATGTACACCAAGGCGGGAGTGTTGTGGTATCGGGGTAACAAAAGGCCTTGAGGC
TCGATAACACGACGGATGACAGATAGACCGGCACAACGGAATTGGTCATTGTTCTCCTCAAAATACTCAGTGA
GGCCCGCTTCGGACCTCACTTGTCGATGAGGTTCAAGTGCTTGTAGCCCATCGAATGTGCATCCCCTAGCACTT
CCTTGTCGAGAGCTTTGCCATGGTTGACTCTGGCCAAATAGCTGAGCCATGGATCCATGGAACAAGAGGAAAA
TGCAAAAGTAAGATAAAAGTGATGGGAAACTAGTAGTTGCCATTCTTGTTGGAAGAGATTGAGAAGGTTGGT
TGATGGAGGTGTTGTGGGATGTAAGGGGATTTTATAGCTAATAAAAGTGTCCTACACCTTATGTAAAGATATG
GTTTGCTAAGATAAATGTGTGATGACATGAAAATTTGGACGTAATAATAGAAAAAAAATGAATGGATAAGCAT
GATCGCAAGC

>chr3D:471262051-471264181

GAAGGGGATTAGTCAGAGAATATAAAATTTAATAAAGAACAAGACACAAACAAAGGATATAACATGATGAAG
TTTTTTATTAAGATAAGCGGCTATACACCACCAACTTTCACACTCGGGATGCCTTTATTTCACTATATTGGTCCTC
ATTACACGCACTAATTTACTCTGATGCCTTATTAGTCAAAAATGACTCGTACTCGTCCTCTGGATAAGGTTGGAA
GCTCGTTTGGGTAAATTTTGGAGAGAATACACCAGACTCTTCTCCCCTGTTGTTTTTGAGGTTCCGAGCTTCCTG
TCTAGAAATACGGTATGCATTGGCGAGGACATCAACGGGTAGGGCACGAAGGATGGAGCTCTTTCCTGCGAT
GTGGCTAACCATGGAGTTTGGGTTGGTCTTGAATGAGATATATTGGCATCCTTCACGCTCCGCCTTCTTAAGAA
CAATGTAATGTTGTGGTACGATTAGCAACTGCCCCTGGCGAAGACGGTCACTGAATACAGTCTGACCATTGTTA
TTGACAACTTGAACTCGAGCATGCCCTTGGATCATGTAGACCACACTGTGTGCATTGATGTTCCAGAATGGTGA
AAGAACAACATTCTGCAATAGCAATTATTTGTATGCCATATTATTAATCGGATGTTATGAAGCTTAGACTATCTA
ACAATAAGATATTGCATTGAAGGTAGTATGGTATATACCTGGTATAGATTTACTCTTGTGGCTCTCATTTGCACA
AGGTTAAGGATGGGGAAATTCTGGCCATGGAGACGTGTTATCCTGCCAGCACGTGGGTTGTACGTGTCGGCA
CGTTTGGGGTTTTCGATGTTCTACCTTGCCTCCAATGAACAAAAGTTCTCCTCCAAACCATTGAAACTTCTGTCTT
GTGACTGTCCTACCTGGTATTGAGTTGATTGTCCTTCTTGATATTGGGTTGATTGCCCTACCTGGTATTGGGTTG
ACTGTCCTTCTTGACTTTGAATTGGTTGGTAGACTTGATGCTCTACTGGTCCTTGTTGGGACACTGTTGGCTTCA
AGAACCGTAGGCCTTGACTCACACGGATTATCTCACCCCTTTGGTCTTTTTGACTCTGGATCCTCTATGCTAGTT
GTTGGCTTATACCAAGAGCCTCACTAAGAAGTTGGATATTGAATCCACTGAATATGTTTTGTCCAACTTGTTGAT
CTACCTTGTTGTTACCAGCCAACAAAAATTCTGGAGATTTCATATTGATGAAGTAAAACGAAGTTCCTTAATATC
CCACTAATATATACTTTAAAATCAAAGTTAGGTAACTAAAGAATTATATATCTTTATGTAGATTAATTTGTGTAG
TTACCTTTTGTCTAGGTTCAAGCTGATTAGCGTTGTTGTTTACGTCGAAGACATAGATAGCTACAACCGGCGCA
TCACCATCATTGTAGCCCCAGTGTACAATGCCAGCTGGCAGCGCAATAACATCTCCTTGTTTAAAGCGGTGAAA
TCTCTGGTGCTCATCTTTAAGATGGCTTTGGCTTTGACCCTGGGCTTGATCAAATGGTTGGAACCGTTGTTGGA
AGGTCGCTGGACATCCAGGTAAAGTCAACCCCGTGAAACCCCTACCTAAAATAAGACACAATTGTTTCAATATT
AACAACTTATGAATAAGGTAATATTTGGAACATTTGGACTCTGAAGTGTAGTATAGCAATGGTATAGCAATGCC
ATCACCATATTCGACTATACACGAACCTTGCAGGACGTACACCAAGCCGGGTGCATTGTGGTATTGAGGTAAC
AAGAGGCCTTGGGGATCAATAACACGACGGATGACAGATACCCCAGCACAACGTAATTGCTCATTCTGCTCAT
CAAAGTACTGAGTGGTGCCCGCTTGTGACCTCACTTGTCGAACTGGTTCAAGTTCTTGTAGCCGATCGAATCTT
CACCCCCTTAAACCTCCTTGTCGAGAGCTTTGCCATGGAGTAAAGCTCTGTCCGAATAGCTGAGCCATGGATCC

ATGGCACAACAGGAAAATGCAGAAGTAAAACAACACGGATGAAAAACTAGTAGTTGCCATGATTGGTTGAAG
AGGTTGTACAAGGTGGACTTCTGCTATTTTCCTAATTGTAAGCTTGGTTTTATA

>chr3D:471337881-471340181

TAGTCAGAGAATATAAAATTTAATAAAAAAAACAACACACAAACAGAGGATATAACATGGTGAAGTTTTTTATT
AAGATAAGCGGTTAAATACCACCCACTTTCACACTTGCGGTGCCTTTATTTCACTATATTGGTCCTCATCACACT
CACTAATTTACTCTGATGCCTTATTAGTCAAAGATGACTCATCCTCGCCCTCTGGATAAGGTTGGAAGCTCGTTT
AGGTAAATTTTGGAGTGAATACACCAGACTCTTGTCCCCTGTTGTTTTTGAGGTTTCGGGCTTCTTGCCTGGAA
ATGCGGTATGCATTGGCGAGGACATCCACGGGCAAGGCACGAAGAATGGAGCTCTTTCCAGCGATGTGGCTA
ACCATGGAGTTTGGGTTGGTCTTGAATGAGATATACTGGCATCCTTCACGCTCCGCCTTCTTGAGAACAACGTA
GTGTTGTGGTACGATTAGCAACTGACCTTGGCGAAGACGGTCATTGAATACAGTCTGACCATTGTTATTGACAA
CTTGAACTCGAGCATGCCCTTGGATCATGTAGACCACACTGTGTGCATTGATGTTCCAGAATGGTGAAAGAAT
AGCATTCTGCAATAGCAATTATTTGTATGCCATATTATTAATCGGATGTTATGATGCATAGAGTATCTAACAATA
AGATAGCGCATTGAAGGTAGTATGGTATATACCTGGTATAGATTTACTCTTGTGGCGCTCATTTGCACGAGGTT
AAGGATGGGGAAATTCTGGCCATGGAGACGTGTTATCCTACCAGCACGTGGGTTGTACGTGTCGGCACGTTTG
GGGTTTTCGATGTTCTGCCTTGCCTCCAATGAACAAAAGTTCTCCTCCAAACCATTGAAACTTCTGTCTTGTGAC
TGTCCTACCTGGTATTGAGTTGATTGTCCTTCTTGATATTGGGTTGATTGCCCTACCTGGTATTGGGTTGATTGT
CCTTCTTGACTTTGAATTGGTTGGTAGGCTTGATGCTCTACTAGTTCTTGTTGGGACATTGTTGGCTTCAAGAAT
TGAAGGCGTTGAGTCACACGAATTATCTCACCTCTTTGTTCCTTTTGACTTTGGATCCTCTGTGCTGCTTGTTGA
CTTATACCAACAGCCTCACTAAGAAGTTGGATATTGAATCCGCTGAATATGTTTTGTCCAAATTGTTGATCTTCC
TTATTGTTACCAGCCAACAAGAACTCCTGAGAATTCATATTGATGAAGTAAAATAAAGTTCCTTAATATCCCACT
AATACATATACTTTAAAATCGTAGTTAGGTAACTAAATAAATATATATCTTTATGTGGATTAATTTGTATAGTTA
CCTTTTGTCTAGGTTCAAGTTGATTAGCATTGTTGTTTACGTCGAAGACATAGATAGCTACAACCGGAGCATCA
CCATCATTGTAGCCCCAGTGTACAATGCCAGCTGGCAGCGCAATAACATCTCCTTGTTTAAAGCGGTGAACTCT
TTGGTGCTCATCTTTGAGATGGCTTTGGCTTTGACCCTGGGCTTGATCAAATGGTTGGAACTGTTGTTGGAAGG
TCGCTGGGCATCCAGGTAAAGTCAACCCCGTGAAACCCCTACCTAAAATAAGACACAATTGTTTCAATATTAAC
AACTTATGAATGAGGTAATATTTAGAACATTTGGACTCTGAAGTGTAGTATAGCAGTGGTATAGCAATGCAAT
CACCATATTCGATTATACACGAACCTTGAAGGACGTACACCAAGCCGGGTGCGTTGTGGTATTGAGGTAACAA
GAGGCCTTGGGGCTCAATAACACGACGGATGACAGATACCCCGGTACAACGTAATTGCTCATTCTGCTCATCA
AAGTACTGAGTGATGCCCGCTTGTGACCTCACTTGTCGAACTGGTTCAAGTGCTTGTAGCCTATCGAATCTGCA
TCCCCTTAAACCTCCTTGCCGAGAGCTTTGCCATGGAGTAAAGCTCTGTCCAAATAGCTGAGCCATGGATCCAT
GGCACAACAGGAAAATGCAGAAGTAAAACAACATGGATGAAAAACTAGTAGTTGCCATGATTGGTTGAAGAG
GTTGTACAAGGTGGACTGGTGCTAGTTCCCTAATTGTAAGCTTGGTTTTATAGCTAGAAAAAGTAATTTCAGAT
GCACATGATGACGTAATTATTAATAGAGATAAGAAGATATGCTTGGTGACTAGATGTTGTTTAAAATGTAAATA
GATATAACTTCTACTCAAAAGTTAGGTGTTGCTTTTCGCCTATAATTAGCTAGTGCTGACTCACAAAGCTAATAC
CACC

>chr3D:471346323-471348323

TAGTCAGAGAATATAAAATTTAATAAAAAAACAACACACAAACAGAGGATATAACATGGTGAAGTTTTTTAGT
AAGATAAGCGGTTTGAACGAACATGGACTGCTTGCCGTCGCAGAACCTTTTTGGGATAGTTTCTATTGAACTGT
TTTGGCCGACTTTCTGTCGAACCCATTTGGGCGAGATTTCGCCGGCGAGAAGCAAAAAATTCAAATATATCGCC
AAACGATCCAGATCCCCCGGGGGGTCGCGCCTGCCCATGGTCTGGGTCGCGCACCTGTGGACGCGTGAACGT
GGTGGGCCCATGCGAAGCAGTCTACTCTATCTTCTTCCTTTTCCTCTCCCGACAGATCCCAAATGCGTTGTTGTC
CTTCACCGGCCTCCTCCTGCCACTGTCGCTCCCATCACTCGCCTCCCAGCCGTCGCTGTTAGCCACCAGCGCCGC
CTTGCCGTGTGTGCTGATGTTGTAGACCGAATCCCTCGGTGTCGCCGTCCTTCAGCATCTCCGACCGACTACGT
GCGGGTCACCGGAGGTCAAGGCGTCCATGGCGCCACTGACCCTGAGCTACCGCGGCGGTAGGAGCCCGTGG
GCTTCACGCACCTCTCACAGCCCTCTCGTTGCCTCCATGGCCTAGGGTCCCTCGTGTCGGAGATTTTTGCTACAT
GCGTCCGCCGATGGTGCTACGCGTGGCGCCGGAGTTTGCTACTTACAATTAGCCGTGGTGCTATCTTCGCACAT

TTTATTTGCTACAGCCGTTTTATAGTTTCCCTATATTAGCACAAAGTTTTTGCTACGATGTCTCGAGCGAGTCTCC
GGTGAAATCGGCGTTTGGTACATTATCACACTTTGTTTGCTACAACCGTTTTGTGGTTTTTCTACAATAGCGCTA
TTTTTGCTACGAGATCTCTAGCGAGGTCTCCCGGCAAAAGTCTCCGGTGATTCTCCGGCGAAGTCAAGTTTTAC
TATATTATCACATTTTGTTTGCTACAAATGTTTTGTGGTTTTGCTACAATAGTGCATAGTTTTTGCTACAAGTTCT
CCGGCGAGGTCGACTTTGCTACATTAGCACATTTTTTCTACAAAGTCTCCGGCGGGTTTCCGATGAAGTCTCCTC
CGGCGAGACACCCGTTTTTACTTTTTTTTAGCAGACCTTTTTTGCTACATGTAGCAAAAGCGGGACATTTTGTAG
GATGCTTGTAGCAAAATAAGGAATTAGGAAAAAATGAGGGACACGTGGCGGCTAGGGAAGCAGGACGATAG
GAACGCTCCCATCGTCCGGGGGACGCCTAGCGCTCGCCGTTCTACATTTGGTTGGGAACACGACTGGAAAACT
GACCGTTCCCAGCCCGAAAGTTCCGTCCATCCGACATCCAGAGCTTCCTTGAGCTTCCTTGCCCTCTGAGTCCGA
CTGGAAACTATTGTCCTCTAAGTCCATCTAGGCCGTCCATCGCCGATCGGACGGTGTAGCCCAGAGCTTTCATG
CCCTGGCGATAGCGCCGCCGCGCAACCGGCGAACTATTGGACTATGTGCTCTTGGACATCAAAGAAGAGATGT
TCTGGAAACTAAATGTTCAGTTAGTTGTCTAGCAAGAAAAGCACAAGACCGAAGAGCAACTACTAAGACGTGC
CATCCCACATCTCGGATCGTGTTCACCGTGATCCTAGAAACTAGGACTTGCATTGCAGATGGGATTGATCCAGT
TAAAGTAACCGAGCTGAGACTAAGCTCCTCCTAGCTCGTCAAGTTACCTATCCCTTCGGGTAGTGTGCCAATGA
GCCCCAATCCCCCAAAGCCATTGAGCTACTACTACCACGGGTGAGGTTTTGTCTTATGTTTCAGTCGATTTTAGC
GGAGAAGAGTCAAACCTAATATTTTGGCATCTTAGGCTGTTGATGGTTATTATATACACATAAATTATACACGT
ACGGTCATAGAAAAAATTCTCTCTCTGCCAATGAACAATACACCATTATTTCATGCTTGTTGGACTCGAGGATA
CT

>chr3D:471348727-471352481

AGGATATAACATGGTGAAGTTTTTTTATTAAGATAAGCGGTTAAATACCACCCACTTTCACACTTGCGGTGCCTT
TATTTCACTATATTGGTCCTCATCACACTCACTAATTTACTCTGATGCCTTATTAGTCAAAGATGACTCATCCTCG
CCCTCTGGATAAGGTTGGAAGCTCGTTTGGGTAAATTTTGGAGTGAATACACCAGACTCTTGTCCCCTGTTGTT
TTTGAGGTTTCGGGCTTCTTGCCTGGAAATGCGGTATGCATTGGCGAGGACATCCACGGGCAAGGCACGAAG
AATGGAGCTCTTTCCAGCGATGTGGCTAACCATGGCGTTTGGGTTGGTCTTGAATGAGATATACTGGCATCCTT
CACGCTCCGCCTTCTTGAGAACAACGTAGTGTTGTGGTACGATTAGCAACTGACCTTGGCGAAGACGGTCATT
GAATACAGTCTGACCATTGTTATTGACAACTTGAACTCGAGCATGCCCTTGGATCATGTAGACCACACTGTGTG
CATTGATGTTCCAGAATGGTGAAAGAATAGCATTCTGCAATAGCAATTATTTGTATGCCATATTATTAATCGGA
TGTTATGAAGCATAGAGTATCTAGCAATAAGATAGCGCATTGAAGGTAGTATGGTATATACCTGGTATAGATT
TACTCTTGTGGCGCTCATTTGCACGAGGTTAAGGATGGGGAAATTCTGGCCATGGAGACGTGTTATCCTACCA
GCACGTGGGTTGTATGTGTCGGCACGTTTGGGGTTTTCGATGTTTTGCCTTGCCTCCAATGAACAAAAGTTCTC
CTCCAAACCATTGAAACTTCTGTCTTGTGACTGTCCTACCTGGTATTGAGTTGATTGTCCTTCTTGATATTGGGTT
GATTGCCCTACCTGGTATTGGGTTGATTGTCCTTCTTGACTTTGAATTGGTTGGTAGGCTTGATGCTCTACTAGT
TCTTTTTGGGACATTGTTGGCTTCAAGAATTGAAGGCGTTGAGTCACACGAATTATCTCACCTCTTTGTTCCTTT
TGACTTTGGATCCTCTGTGCTGCTTGTTGACTTATACTAAGAGCCTCACTAAGAAGTTGGATATTGAATCCTCTG
AATATGTTTTGTCCAAATTGTTGATCTTCCTTATTGTTACCAGCCAACAAGAACTCCTGAGAATTCATATTGATG
AAGTAAAATGAAGTTCCTTAATATCCCACTAATACATATACTTTAAAATCGTAGTTAGGTAACTAAATAAATATA
TATCTTTATGTGGATTAATTTGTATAGTTACCTTTTGTCTAGGTTCAAGTTGATTAGCGTTGTTGTTTACGTCGAA
GACATAGATAGCTACAACCGGAGCATCACCATCATTGTAGCCCCAGTGTACAATGCCAGCTGGCAGCGCAATA
ACATCTCCTTGTTTAAAGCGGTGAACTCTTTGGTGCTCATCTTTGAGATGGCTTTGGCTTTGACCCTGGGCTTGA
TCAAATGGTTGGAACTGTTGTTGGAAGGTCGCTGGGCATCCAGGTAAAGTCAACCCCGTGAAACCCCTACCTA
AAATAAGACACAATTGTTTCAATATTAACAACTTATGAATGAGGTAATATTTAGAACATTTGGACTCTGAAGTG
TAGTATAGCAGTGGTATAGCAATGCAATCACCATATTCGATTATACACGAACCTTGAAGGACGTACACCAAGCC
GGGTGCGTTGTGGTATTGAGGTAACAAGAGGCCTTGGGGCTCAATAACACGACGGATGACAGATACCCCGGT
ACAACGTAATTGCTCATTCTGCTCATCAAAGTACTGAGTGATGCCCGCTTGTGACCTCACTTGTCGAACTGGTTA
AAGTGCTTGTAGCCTATCGAATCTGCATCCCCTTAAACCTCCTTGCCGAGAGCTTTGCCATGGAGTAAAGCTCT
GTCCAAATAGCTGAGCCATGGATCCATGGCACAACAGGAAAATGCAGAAGTAAAACAACACGGATGAAAAAC
TAGTAGTTGCCATGATTGGTTGAAGAGGTTGTACAAGGTGGACTGGTGCTAGTTCCCTAATTGTAAGCTTGGTT

TTATAGCTAGAAAAAGTAATTTCAGATGCACATGATGACGTAATTATTAATAGAGATAAGAAGATATGCTTGGT
GACTAGATGTTGTTTAAAATGTAAATAGATATAACTTCTACTCAAAAGTTAGGTGTTGCTTTTCGCCTATAATTA
GCTAGTGCTAACTCACAAAGCTAATACCACCGTTTGTACGTAGCTCTTGGCTATTGTGTGTACACATGATATTAC
TTGTACTTTCTAAAACAATTTTTGGCACTTTGTAACACCTTTTTATGGTTTTTTCATGTTTTGTTATAGATACATCA
AGACAGTGGATACTTTTGGTTGTGTGGAAGGAATTCCATGGCTCATGTCTATGGGTATATCTTGTGGATGAGTC
ATGCCCATGTTGATACAAGAATATACTTGTGGATAAGAAACTTATAATAACGTGCAATCCATCTAAGTTAGAAA
GATAGTGTACGAGCAATTTGTTGGTTTACTTGTTTCTAACGACGGATAAAAATATGAGTACGGAAACGAGTTG
CAAAGTGCGATTCGTTGGGCCGTCTGAATCCGTATTTGCTATATTTGTCAACATGGTCGTTTGTTTAACTTAATC
ACCCATGTGAGAGCATACCCACATGAATTACATTAATATTTACCTGTAAATATTTTCAACCTATCGTAATTTGGG
TAAGTGCTAGGTAGGACTTGAAAGTCAGGAGACAAAACAAATAAAAATAGAAAATAATAGAGCCAATGCACA
CAAGTGTTTGAGACGCTAAGAAACACAAGTAGGCAAATCAAAGATGGTCGTCTTGAAACCCGATGAGAATCTA
GGTGGCACGAGTCAAAAGCGACGCCAAAAGGGGGTCGAGAGGGGTTGGGCGACACTTCTCGCTAATACCCAC
AGTTGGTATCCAATCTAAATGGGCCTTAACAGCAACATTGAAATAGCCCGACACGAGGCAGACTTCCTTCGGA
AAAAAACGTGAGTCGAGCAACGCAAGTCAAACTCTCGATCCCTACTCGGCTTCGTCCGCCTCCTGACGCCGTCA
TCATGGTCCATCGTTGCCGCACCGCGCTGCCCCTCTCCGTGTAGGCATGCAGCTCGATATCCTCCCTGTTGGCCT
GGGCCTCCTCTCTGTTGCAGTCCCGACCCGACAAATCCAAGCGCCAAAAGGAATAGCGCCGCCGCCTAACACT
CACACTGATGAACGTCGAGGAGAAGGCAGCTTGGATACTGGCCGAGGAATAAGAAGAGGCAGAGCTGGCTG
AGGAAGAAGAAGAAGCTGCACAAGCCGAGGAGAAGGAGGAGGAGGAAGAGGAAGAAGAAGAAGAAG
AAGAAGAAGAAGAAGAAGAAGAAGAAGAAGAAGAAGAAGAAGAAGAAGAAGAAGAAGAAGAAGAAGAGG
GAACGGCAGGAGAGGTTATTTTCCGCATGTGATCTCAGTTTTGAATTTCAGGTCTGATTTTAGTCTAGTGACTA
CTGACTAGTGGTCTGTAGGAGATTGTTCAATTTCGATTTTAATTTTGTAGGACACTCAACAATCAAATGCTCCTA
AAATGTTTGATCTACCAAATGTTGTCGCCAATCCTGAAATTAGGAGACCAATTGATGAATTTGGTATCCCTGGA
ATTAGAGATAAAATGAGATGGGCATATTTGTCGAGAAAACCTAGTCGTTCGATGCGTTTCCAT

>chr3D:471356311-471358711

AAAAACTCGCAAAACTAGATTTATAGTTAGGACCTGAAAAATATTAGAACCACGAAAAACGGTTCTAGGAAAC
AAATTCTTATCCAGTTTGCCCATAAGACTCATTGATGCGCATGAGAATGAGGTACAAAACGGCAGCATAGCCTT
GAAGAGAGGGAAGGGGATTAGTGAGAGAATATAAAATTTAATAAAAAAAACAACACACAAACAGAGGATAT
AACATGGTGAAGTTTTTTATTAAGATAAGCGGTTAAATACCACCCACTTTCACACTTGCGGTGCCTTTATTTCAC
TATATTGGTCCTCATCACACTCACTAATTTACTCTGATGCCTTATTAGTCAAAGATGACTCATCCTCGCCCTCTGG
ATAAGGTTGGAAGCTCGTTTGGGTAAATTTTGGAGTGAATACACCAGACTCTTGTCCCCTGTTGTTTTTGAGGT
TTCGGGCTTCTTGCCTGGAAATGCGGTATGCATTGGCGAGGACATCCACGGGCAAGGCACGAAGAATGGAGC
TCTTTCCAGCGATGTGGCTAACCATGGAATTTGGGTTGGTCTTGAATGAGATATACTGGCATCCTTCACGCTCC
GCCTTCTTGAGAACAACATAGTGTTGTGGTACGATTAGCAACTGACCTTGGCGAAGACGGTCATTGAATACAG
TCTGACCATTGTTATTGACAACTTGAACTCGAGCATGCCCTTGGATCATGTAGACCACACTGTGTGCATTGATG
TTCCAGAATGGTGAAAGAATAGCATTCTGCAATAGCAATTATTTGTATGCCATATTATTAATCGGATGTTATGA
AGCATAGAGTATCTAGCAATAAGATAGCGCATTGAAGGTAGTATGGTATATACCTGGTATAGATTTACTCTTGT
GGCGCTCATTTGCACGAGGTTAAGGATGGGGAAATTCTGGCCATGGAGACGTGTTATCCTACCAGCACGTGG
GTTGTACGTGTCGGCACGTTTGGGGTTTTCGATGTTCTGCCTTGCCTCCAATGAACAAAAGTTCTCCTCCAAACC
ATTGAAACTTCTGTCTTGTGACTGTCCTACCTGGTATTGAGTTGATTGTCCTTCTTGATATTGGGTTGATTGCCCT
ACCTGGTATTGGGTTGATTGTCCTTCTTGACTTTGAATTGGTTGGTAGGCTTGATGCTCTACTAGTTCTTGTTGG
GACATTGTTGGCTTCAAGAATTGAAGGCGTTGAGTCACACGAATTATCTCACCTCTTTGTTCCTTTTGACTTTGG
ATCCTCTGTGCTGCTTGTTGACTTATACCAAGAGCCTCACTAAGAAGTTGGATATTGAATCCGCTGAATATGTTT
TGTCCAAATTGTTGATCTTCCTTATTGTTACCAGCCAACAAGAACTCCTGAGAATTCATATTGATGAAGTAAAAT
GAAGTTCCTTAATATCCCACTAATACATATACTTTAAAATCGTAGTTAGGTAACTAAATAAATATATATCTTTAT
GTGGATTAATTTGTATAGTTACCTTTTGTCTAGGTTCAAGTTGATTAGCGTTGTTGTTTACGTCGAAGACATAGA
TAGCTACAACCGGAGCATCACCATCATTGTAGCCCCAGTGTACAATGCCAGCTGGCAGCGCAATAACATCTCCT
TGTTTAAAGCGGTGAACTCTTTGGTGCTCATCTTTGAGATGGCTTTGGCTTTGACCCTGGGCTTGATCAAATGG

TTGGAACTGTTGTTGGAAGGTCGCTGGGCATCCAGGTAAAGTCAACCCCGTGAAACCCCTACCTAAAATAAGA
CACAATTGTTTCAATATTAACAACTTATGAATAAGGTAATATTTAGAACATTTGGACTCTGAAGTGTAGTATAG
CAGTGGTATAGCAATGCAATCACCATATTCGATTATACACGAACCTTGAAGGACGTACACCAAGCCGGGTGCG
TTGTGGTATTGAGGTAACAAGAGGCCTTGGGGCTCAATAACACGACGGATGACAGATACCCCGGTACAACGT
AATTGCTCATTCTGCTCATCAAAGTACTGAGTGATGCCCGCTTGTGACCTCACTTGTCGAACTGGTTCAAGTGCT
TGTAGACTATCGAATCTGCATCCCCTTAAACCTCCTTGCCGAGAGCTTTGCCATGGAGTAAAGCTCTGTCCAAA
TAGCTGAGCCATGGATCCATGGCACAACAGGAAAATGCAGAAGTAAAACAACACGGATGAAAAACTAGTAGT
TGCCATGATTGGTTGAAGAGGTTGTACAAGGTGGACTGGTGCTATTTCCCTAATTGTAAGCTTGGTTTTATAGC
TAGAAAAAGTAATTTCAGATGCACATGGTGACGTAATTATTAATAGAGATAAGAAGATATGCTTGGTGACTAG
ATGTTGTTTAAAATGTAAATAGATATAACTTCTAC

>chr3D:471358465-471361681

CCATGGATCCATGGCACAACAGGAAAATGCAGAAGTAAAACAACACGGATGAAAAACTAGTAGTTGCCATGA
TTGGTTGAAGAGGTTGTACAAGGTGGACTGGTGCTATTTCCCTAATTGTAAGCTTGGTTTTATAGCTAGAAAAA
GTAATTTCAGATGCACATGGTGACGTAATTATTAATAGAGATAAGAAGATATGCTTGGTGACTAGATGTTGTTT
AAAATGTAAATAGATATAACTTCTACTCAAAAGTTAGGTGTTGCTTTTCGCCTATAATTAGCTAGTGCTGACTCA
CAAAGCTAATACCACCGTTTGTACGTAGCTCTTGGCTATTGTGTGTACACATGATATTACTTGTACTTTCTAAAA
CAATTTTTGGCACTTTGTAACACCTTTTTATGGTTTTTTCATGTTCAAGACAGTGGATACTTTTGGTTGTGTGGA
AGGAATTCCATGGCTCATGTCTATGGGTATATCTTGTGGATGAGTCATGCCCATGTTGATACAAGAATATACTT
GTGGATAAGAAACTTATAATAACGTGCAATCCATCTAAGTTAGAAAGATAGTGTACGAGCAATTTGTTGGTTTA
CTTGTTTCTAACGATGGATAAAAATATGAGTACGGAAATGAGTTGCAAAGTGCGATTTGTTGGGTCGTCTGAA
TCCATATTTGCTATATTTGTCGACATGGTCGTTTGTTTAACTTAATCACCCATGTGAGAGCATACCCACATGAAT
TACATTAATATTTACCTGTAAATATTTTCAACCTATCGTAATTTGGGTAAGTGCTAGGTAGGACTTGAAAGTCAG
GAGACAAAACAAATAAAAATAGAAAATAATAGAGCCAATGCACACAAGTGTTTGAGACGCTAAGAAACACAA
GTAGGCAAATCAAAGATGGTCGTCTTGAAACCCGATGAGAATCTGGGTGGCACGAGTCACAAGCGACGCCAA
CAGGGGGTCGAGAGTGGTTGGGCGACACTTCTCGCTAATACCCACAGTTGGTATCCAATCTAAATGGGCCTTA
ACAGAAACATTGAAATAGCCCGACACGAGGCAGACTTCCTTCGGAAAAAAACGTGAGTCGAGCAACGCGAGT
CAAACTCTCGATCCCTACTCGGCTTCGTCCGCCTTCTGACGCCGTCATCATGGTCCATCGTGGCCGCGCTGTGCT
GCCCCTCTCCGTGTAGGCATGCAGCTCGATATCCTCCCTGTTGGCCTGGGCCTCCTCTCTGTTGCAGTCCCGACC
CGACGAATCCAAGCGGCAAAAGGAATAGCGCCGCCGCCTAACACTCACACTGATGAACGTCGAGGAGAAGGC
AGCTTGGATACTGGCCGAGGAATAAGAAGAGGCAAAGCTGGCTGATGAAGAAGAAGAAGCTGCACAAGCCG
AGGAGGAGGAGGAGGAAGAAGAAGAAGAAGAAGAAGAAGAAGAAGAAGAAGAAGAAGAAGAATAATAAGAAG
AAGAAGAAGAAGAAGAAGAAGAAGAAGAAGAAGAAGAAGAAGAAGAAGAAGAAGAAGAAGAAGAAGAAGAAG
AAGAAGAAGAGGCAACGGCAGGAGAGGTTATTTTCCGCATGTGATCTCAGTTTTGAATTTCAGTCTCATGTTA
GTCTAGTGACTACTGACTAGTGGTCTGTAGGAGATTGTTCATTCCTCATTGTTCAATTTCGATTTTAATGTTGTT
GGACACTCAACAATCAAATGCTCCTAAAATGTTTGATCTAGCAAATGTTGTCGCCAATCCTGAAATTAGGAGAC
CAATTGATGAATTTGGTATCCCTGGAATTAGAGATAAAATGAGATGGGCATATTTGTCGAGAAAACCTAGTCG
TTCGATGCGTTTCCATTCCTACAAGGACCATCATGGTCATCTTCCTCGACAATTTCGTCTTGCTGACGCGGAAAC
AACTTAATGGTAATACGGTATGATACTAGTAATTTCTAAGACAATCTTGTGTGTTTTTATGTTCTTTCTAGTTGTA
CTCGATGAATTATGAATATTGAATCTTTGTCGACCATTTGTTCTGTAGAGTATCCTTGCTTTTGTGTCCAAAAAC
AATTAAAACTTTTTTGTTCAACTCGGTTCGGTTCAATTCCTGGCTCCACCACTGGCACGAGTGGTATGTGAGTGT
CGATGATCTGGTAGGAAGATCTCCCTTTCATGAGGATTGGAGGCACTGAACCATATGAGCATGTGGATCGCGC
TAGGGGTATAGACTAGCAAGGATCTTGCTAGAAGGAGAGCTCCCGTCAATTGATGATTAAACAAAGATATAAA
ATAAGATGAGCATCGAGATCGCACAATTGGTACAAGAGTGTCAATGATTCATAGGAGCTCGTTGCCATTTTTCC
AGGATCACAAGAAGAACAAAGTTTAACGATACGAGGAGAAGTAGATTTCGGTGGGGGCCAAAGCATATGAGT
CTCATTAGCACATTCAAATAATTTCAGATATACAAGGATTCTATTGCAAACATTTTATCGCCGGTGAGAAAATGC
AGTGAGTAGTAAGAGCATCTCCACTAGCATTCCCAGGAGGGCTTCCCCGGGATATTTTTTATCCGGACGGCGA
AAATTCGCCCAGACGCATTCTCACGTGCCTAGTTTTCGCCGGATTTGGCCCAAATTACACCCGGGTTCCTAGGC

CGAACACGGGGCGCCAAGAGTCTCCTGGGAACACCGGATGAAGCGAAAAAAGGTGGGCCAACCCTGTCAGC
AACATGAGGCTGAGCTCCCGCCTTTCTTCTCCATTCCCCACCACAACTCCCGCTAGTTCCTCGAGCTCCTGCCCG
ACATGCCACCCAAGGGACTCGTCAAACCCCGCTCCACCTTCGGGGAACCAAAGGAGAAGAAGCCAAGAAAGC
CGAGAAAGGAGACTCAAACTTCTCCTTATGTTCGCAGCTGACCCACGCACTCGGCATGTACCACGACGACACC
AAGGCTGAGTTCCAGTTTCACCATGTGTTCGACAAGATCGAGAAGTGCCAGAAATGGCTGGACACACGCGTGT
CCCTCGCTAAGGACAAGGACGGCGTGTACAACCCCGAGGCGCCAGCCCCAGCTACCGGCGAAGGAAGGCCAG
AGCTCGGCCAGAAGAAGGCCAAGGCGCTGATGGTGACGGCACCGCCTGTGGAGCGGTTTCACGCTTCAATCG
AGAAGTCCATCGTTGACTCGAAGGCGCAATGCCGAGAAGAGGGAGGATAAGGCCGAAAAAAG

>chr3D:471364869-471371178

TCTAGAAAACAAATTCTTATCCAGTTTGCCCATAAGACTCATTGATGCGCATGAGAATGAGGTACAAAACGGCA
GCATAGCCTTGAAGAGAGGGAAGGGGATTAGTCAGAGAATATAAAATTTAATAAAAAAAACAACACACAAAC
AGAGGATATAACATGGTGAAGTTTTTTATTAAGATAAGCGGTTAAATACCACCCACTTTCACACTCGCGGTGCC
TTTATTTCACTATATTGGTCCTCATCACACTCACTAATTTACTCTGATGCCTTATTAGTCAAAGATGACTCATCCT
CGCCCTCTGGATAAGGTTGGAAGCTCGTTTGGGTAAATTTTGGAGTGAATACACCAGACTCTTGTCCCCTGTTG
TTTTTGAGGTTTCGGGCTTCTTGCCTGGAAATGCGGTATGCATTGGCGAGGACATCCACGGGCAAGGCACGAA
GAATGGAGCTCTTTCCAGCGATGTGGCTAACCATGGAGTTTGGGTTGGTCTTGAATGAGATATACTGGCATCC
TTCACGCTCCGCCTTCTTGAGAACAACGTAGTGTTGTGGTACGATTAGCAACTGACCTTGGCGAAGACGGTCAT
TGAATACAGTCTGACCATTGTTATTGACAACTTGAACTCGAGCATGCCCTTGGATCATGTAGACCACACTGTGT
GCATTGATGTTCCAGAATGGTGAAAGAATAGCATTCTGCAATAGCAATTATTTGTATGCCATATTATTAATCGG
ATGTTATGAAGCATAGAGTATCTAACAATAAGATAGCGCATTGAAGGTAGTATGGTATATACCTGGTATAGAT
TTACTCTTGTGGCGCTCATTTGCACGAGGTTAAGGATGGGGAAATTCTGGCCATGGAGACGTGTTATCCTACCA
GCACGTGGGTTGTACGTGTCGGCACGTTTGGGGTTTTCGATGTTCTGCCTTGCCTCCAATGAACAAAAGTTCTC
CTCCAAACCATTGAAACTTCTGTCTTGTGACTGTCCTACCTGGTATTGAGTTGATTGTCCTTCTTGATATTGGGTT
GATTGCCCTACCTGGTATTGGGTTGATTGTCCTTCTTGACTTTGAATTGGTTGGTAGGCTTGATGCTCTACTAGT
TCTTGTTGGGACATTGTTGGCTTCAAGAATTGAAGGCGTTGAGTCACACGAATTATCTCACCTCTTTGTTCCTTT
TGACTTTGGATCCTCTGTGCTGCTTGTTGACTTATACCAAGAGCCTCACTAAGAAGTTGGATATTGAATCCGCT
GAATATGTTTTGTCCAAATTGTTGATCTTCCTTATTGTTACCAGCCAACAAGAACTCCTGAGAATTCATATTGAT
GGAGTAAAATAAAGTTCCTTAATATCCCACTAATACATATACTTTAAAATCGTAGTTAGGTAACTAAATAAATAT
ATATCTTTATGTGGATTAATTTGTATAGTTACCTTTTGTCTAGGTTCAAGTTGATTAGCATTGTTGTTTACGTCGA
AGACATAGATAGCTACAACCGGAGCATCACCATCATTGTAGCCCCAGTGTACAATGCCAGCTGGCAGCGCAAT
AACATCTCCTTGTTTAAAGCGGTGAACTCTTTGGTGCTCATCTTTGAGATGGCTTTGGCTTTGACCCTGGGCTTG
ATCAAATGGTTGGAACTGTTGTTGGAAGGTCGCTGGGCATCCAGGTAAAGTCAACCCCGTGAAACCCCTACCT
AAAATAAGACACAATTGTTTCAATATTAACAACTTATGAATGAGGTAATATTTAGAACATTTGGACTCTGAAGT
GTAGTATAGCAGTGGTATAGCAATGCAATCACCATATTCGATTATACACGAACCTTGAAGGACGTACACCAAG
CCGGGTGCGTTGTGGTATTGAGGTAACAAGAGGCCTTGGGGCTCAATAACACGACGGATGACAGATACCCCG
GTACAACGTAATTGCTCATTCTGCTCATCAAAGTACTGAGTGATGCCCGCTTGTGACCTCACTTGTCGAACTGG
TTCAAGTGCTTGTAGCCTATCGAATCTGCATCCCCTTAAACCTCCTTGCCGAGAGCTTTGCCATGGAGTAAAGCT
CTGTCCAAATAGCTGAGCCATGGATCCATGGCACAACAGGAAAATGCAGAAGTAAAACAACACGGATGAAAA
ACTAGTAGTTGCCATGATTGGTTGAAGAGGTTGTACAAGGTGGACTGGTGCTATTTCCCTAATTGTAAGCTTGG
TTTTATAGCTAGAAAAAGTAATTTCAGATGCACATGGTGACGTAATTATTAATAGAGATAAGAAGATATGCTTG
GTGACTAGATGTTGTTTAAAATGTAAATAGATATAACTTCTACTCAAAAGTTAGGTGTTGCTTTTCGCCTATAAT
TAGCTAGTGCTGACTCACAAAGCTAATACCACCGTTTGTACGTAGCTCTTGGCTATTGTGTGTACACATGATATT
ACCTGTACTTTCTAAAACAATTTTTGGCACTTTGTAACACCTTTCTATGGTTTTTTCATGTTTTGTTATAGATACAT
CAAGACAGTGGATACTTTTGGTTGTGTGGAAGGAATTCCATGGCTCATGTCTATGGGTATATCTTGTGGATGA
GTCATGCCCATGTTGATACAAGAATATACTTGTGGATAAGAAACTTATAATAACGTGCAATCCATCTAAGTTTG
AAAGATAGTGTACGAGCAATTTGTTGGTTTACTTGTTTCTAACGATGGATAAAAATATGAGTACGGAAATGAG
TTGCAAAGTGCGATTTGTTGGGCCGTCTGAATCCGTATTTGCTATATTTGTCGACATGGTCGTTTGTTTAACTTA

ATCACCCATGTGAGAGCATACCCACATGAATTACATTAATATTTACCTGTAAATATTTTCAACCTATCGTAATTT
GGGTAAGTGCTAGGTAGGACTTGAAAGTCAGGAGACAAAACAAATAAAAATAGAAAATAATAGAGCCAATGC
ACACAAGTGTTTGAGACGCTAAGAAACACAAGTAGGCAAATCAAAGATGGTCGTCTTGAAACCCGATGAGAA
TCTGGGTGGCACGAGTCACAAGCGACGCCAACAGGGGGTCGAGAGTGGTTGGGCGACACTTCTCGCTAATAC
CCACAGTTGGTATCCAATCTAAATGGGCCTTAACAGCAACATTGAAATAGCCCGACACGAGGCAGACTTCCTTC
GGAAAAAAACGTGAGTCGAGCAACGCGAGTCAAACTCTCGATCCCTACTCGGCTTCGTCCGCCTTCTGACGCC
GTCATCATGGTCCATCGTGGCCGCGCCGTGCTGCCCCTCTCCGTGTAGGCATGCAGCTCGATATCCTCCCTGTT
GGCCTGGGCCTCCTCTCTGTTGCAGTCCCGACCCGACGAATCCAAGCGGCAAAAGGAATAGCGCCGCCGCCTA
ACACTCACACTGATGAACGTCGAGGAGAAGGCAGCTTGGATACTGGCCGAGGAATAAGAAGAGGCAAAGCT
AGCTGATGAAGAAGAAGAAGCTGCACAAGCCGAGGAGGAGGAGGAGGAAGAAGAAGAAGAAGAAGAAGA
AGAAGAAGAAGAAGAAGAAGAAGAAGAAGAAGAAGAAGAGGCAACGGCAGGAGAGGTTATTTTCCGCATG
TGATCTCAGTTTTGAATTTCAGGTCTCATGTTAGTCTAGTGACTACTGACTAGTGGTCTGTAGGAGATTGTTCAT
TCCTCATTGTTCAATTTCGATTTTAATGTTGTTGGACACTCAACAATCAAATGCTCCTAAAATGTTTGATCTAGCA
AATGTTGTCGCCAATCCTGAAATTAGGAGACCAATTGATGAATTTGGTATCCCTGGAATTAGAGATAAAATGA
GATGGGCATATTTGTCGAGAAAACCTAGTCGTTCGATGCGTTTCCATTCCTACAAGGACCATCATGGTCATCTT
CCTCGACAATTTCGTCTTGCTGACGCGGAAACAACTTAATGGTAATACGGTATGATACTAGTAATTTCTAAGAC
AATCTTGTGTGTTTTTATGTTCTTTCTAGTTGTACTCGATGAATTATGAATATTGAATCTTTGTCGACCATTTGTT
CTGTAGAGTATCCTTGCTTTTGTGTCCAAAAACATTTAAAACATTTTTGTTCAACTCGGTTCGGTTCAATTCCTGG
CTCCACCACTGGCACGAGTGGTATGTGAGTGTCGATGATCTGGTAGGAAGATCTCCCTTTCATGAGGATTGGA
GGCACTGAACCATATGAGCATGTGGATCGCGCTAGGGGTATAGACTAGCAAGGATCTTGCTAGAAGGAGAGC
TCCCGTCAATTGATGATTAAACAAAGATATAAAATAAGATGAGCATCGAGATCGCACAATTGGTACAAGAGTG
TCAATGATTCATAGGAGCTCGTTGCCATTTTTCCAGGATCACAAGAAGAACAAAGTTTAACGATACGAGGAGA
AGTAGATTTCGGTGGGGGCCAAAGCATATGAGTCTCATTAGCACATTCAAATAATTTCAGATATACAAGGATTC
TATTGCAAACATTTTATCGCCGGTGAGAAAATGCAGTGAGTAGTAAGAGCATCTCCACTAGCATTCCCAGGAG
GGCTTCCCCGGGATATTTTTTATCCGGACGGCGAAAATTCGCCCAGTCGCATTCTCACGTGCCTAGTTTTCGCC
GGATTTGGCCCAAATTACACCCGGGTTCCTAGGCCGAACACAGGGCGCCAGGAGTCGCCTGGGAACACCGGA
TGAAGCGAAAAAAGGTGGGCCAACCCTGTCAGCAACATGAGGCCGAGCTCCCGCCTTTCTTCTCCATTCCCCAC
CACAACTCCCGCTAGTTCCTCGAGCTCCTGCCCGACATGCCACCCAAGGGACTCGTCAAACCCCGCTCCACCTT
CGGGGAACCAAAGGAGAAGAAGCCAAGAAAGCCGAGAAAGGAGACTCAAACTTCTCCTTATGTTCGCAGCTG
ACCCACGCACTCGGCATGTACCACGACGACACCAAGGCTGAGTTCCAGTTTCACCATGTGTTCGACAAGATCG
AGAAGTGCCAGAAATGGCTGGACACACGCGTGTCCCTCGCTAAGGACAAGGACGGCGTGTACAACCCCGAGG
CACCAGCCCCAGCTACCGGCGAAGGAAGGCCAGAGCTCGGCCAGAAGAAGGCCAAGGCGCTGATGGTGACG
GCACCGCCTGTGGAGCGGTTTCACGCTTCAATCGAGAAGTCCATCGTTGACTCGAAGGCGCAATGCCGAGAAG
AGGGAGGATAAGGCCGAAAAAAGGTGGAAGCAGCTTCTGCAGAACCAAGACAAGAAACTCGACCTCCTCAA
GGCCAACTTCGCCGCGAAGAAGAGGAACACAGACCTGCAATTTCTCATGGGTGGCAAAGACACCTCGGCGAT
GAGCCCCCAGGTCAAGGTCTGGTACATGGCGCGCCACAACGCTATTCTGGACGAGGACATCCCTGTCGCCAAC
ATCCGAGCTTTGTGTCGATGCTCTCCGCCACCACCCCATCACCGAGCTCTGCCTCCGCCTCATCGAACCTGTCGA
CGAGCACTGCCGCAGCTGACTCGGCGGAGGATCCCATCCCCATCGACGAGTGATCGCCGAATTACATGTTTTT
GTCATAATCTATTGGCCGAACATGGACTGCTTGCCGTCGCAGAACCTTTTTGGGCTAGTTTCTATTGAACTGTTT
TGGCCGACTTTCTGTCAAACACATTTGGGCGAGATTTCGCCGGCGAGAAGAAAAAAATTCAAATATATCGCCA
AACAATCCAGATCCCCCGGGGGGTCGCGCCTGCCCATGGTCTGGGTCGCGCACCTGTGGACGCGTGAACGTG
GTGGGCCCATGCGAAGCAGTCTACTCTATCTTCTTCCTTTTCCTCTCCCGACGGATCCCAAATGCGTTGTTGTCC
TTCACCGGCCTCCTCCTGCCACTTTCGCTCCCATCACTCGCCTCCCAGCCGCCGCTGTTAGCCACCAGCGCCGCC
TTGCCGTGTGTGCTGATGTTGTAGACCGAATCCCTCGGTGTCGCCGTCCTTCAGCATGTCCGACCGACTACGTG
CGGGTCACCGGAGGTCAAGGCGTCCATGGCGCCACTGACCCTGAGCTACCGCGGCGGTAGGAGCCCGTGGGC
TTCACGCACCTCTCACAGCCCTCTCGTTGCCTCCATGGCCTAGGGTCCCTCGTGTCGGAGATTTTTGCTACATGC
GTCCGCCGATGGTGCTACGCGTGGCGCCGGAGTTTGCTACTTACAATTAGCCGTGGTGCTATCTTCGCACATTT
TATTTGCTACAGCCGTATTATAGTTTCCATATATTAGCACAAAGTTTTTGCT

>chr3D:471373418-471376978

TAGTCAGAGAATATAAAATTTAATAAAAAAAACAACACACAAACAGAGGATATAACATGGTGAAGTTTTTTATT
AAGATAAGCGGTTAAATACCACCCACTTTCACACTTGCGGTGCCTTTATTTCACTATATTGGTCCTCATCACACT
CACTAATTTACTCTGATGCCTTATTAGTCAAAGATGACTCATCCTCGCCCTCTGGATAAGGTTGGAAGCTCGTTT
GGGTAAATTTTGGAGTGAATACACCAGACTCTTGTCCCCTGTTGTTTTTGAGGTTTCGGGCTTCTTGCCTAGAA
ATGCGGTATGCATTGGCGAGGACATCCACGGGCAAGGCACGAAGAATGGAGCTCTTTCCAGCGATGTGGCTA
ACCATGGAGTTTGGGTTGGTCTTGAATGAGATATACTGGCATCCTTCACGCTCCGCCTTCTTGAGAACAACGTA
GTGTTGTGGTACGATTAGCAACTGACCTTGGCGAAGACGGTCATTGAATACAGTCTGACCATTGTTATTGACAA
CTTGAACTCGAGCATGCCCTTGGATCATGTAGACCACACTGTGTGCATTGATGTTCCAGAATGGTGAAAGAAT
AGCATTCTGCAATAGCAATTATTTGTATGCCATATTATTAATCGGATGTTATGAAGCATAGAGTATCTAACAATA
AGATAGCGCATTGAAGGTAGTATGGTATATACCTGGTATAGATTTACTCTTGTGGCGCTCATTTGCATGAGGTT
AAGGATGGGGAAATTCTGGCCATGGAGACGTGTTATCCTACCAGCACGTGGGTTGTACGTGTCGGCACGTTTG
GGGTTTTCGATGTTCTGCCTTGCCTCCAATGAACAAAAGTTCTCCTCCAAACCATTGAAACTTCTGTCTTGTGAC
TGTCCTACCTGGTATTGAGTTGATTGTCCTTCTTGATATTGGGTTGATTGCCCTACCTGGTATTGGGTTGATTGT
CCTTCTTGACTTTGAATTGGTTGGTAGGCTTGATGCTCTACTAGTTCTTGTTGGGACATTGTTGGCTTCAAGAAT
TGAAGGCGTTGAGTCACACGAATTATCTCACCTCTTTGTTCCTTTTGACTTTGGATCCTCTGTGTTGCTTGTTGAC
TTATACCAAGAGCCTCACTAAGAAGTTGGATATTGAATCCGCTGAATATGTTTTGTCCAAATTGTTGATCTTCCT
TATTGTTACCAGCCAACAAGAACTCCTGAGAATTCATATTGATGAAGTAAAATGAAGTTCCTTAATATCCCACTA
ATACATATACTTTAAAATCGTAGTTAGGTAACTAAATAAATATATATCTTTATGTGGATTAATTTGTATAGTTAC
CTTTTGTCTAGGTTCAAGTTGATTAGCATTGTTGTTTACGTCGAAGACATAGATAGCTACAACCGGAGCATCAC
CATCATTGTAGCCCCAGTGTACAATGCCAGCTGGCAGCGCAATAACATCTCCTTGTTTAAAGCGGTGAACTCTT
TGGTGCTCATCTTTGAGATGGCTTTGGCTTTGACCCTGGGCTTGATCAAATGGTTGGAACTGTTGTTGGAAGGT
CGCTGGGCATCCAGGTAAAGTCAACCCCGTGAAACCCCTACCTAAAATAAGACACAATTGTTTCAATATTAACA
ACTTATGAATGAGGTAATATTTAGAACATTTGGACTCTGAAGTGTAGTATAGCAATGGTATAGCAATGCAATCA
CCATATTCGATTATACACGAACCTTGAAGGACGTACACCAAGCCGGGTGCGTTGTGTTATTGAGGTAACAAGA
GGCCTTGGGGCTCAATAACACGACGGATGACAGATACCCCGGTACAACGTAATTGCTCATTCTGCTCATCAAA
GTACTGAGTGATGCCCGCTTGTGACCTCACTTGTCGAACTGGTTCAAGTGCTTGTAGCCTATCGAATCTGCATC
CCCTTAAACCTCCTTGCCGAGAGCTTTGCCATGGAGTAAAGCTCTGTCCAAATAGCTGAGCCATGGATCCATGG
CACAACAGGAAAATGCAGAAGTAAAACAACACGGATGAAAAACTAGTAGTTGCCATGATTGGTTGAAGAGGT
TGTACAAGGTGCACTGGTGCTATTTCCCTAATTGTAAGCTTGGTTTTATAGCTAGAAAAAGTAATTTCAGATGC
ACATGGTGACGTAATTGTTAATAGAGATAAGAAGATATGCTTGGTGACTAGATGTTGTTTAAAATGTAAATAG
ATATAACTTCTACTCAAAAGTTAGGTGTTGCTTTTCGCCTATAATTAGCTAGTGCTGACTCACAAAGCTAATACC
ACCGTTTGTACGTAGCTCTTGGCTATTGTGTGTACACATGATATTACTTGTACTTTCTAAAACAATTTTTGGCACT
TTGTAACACCTTTTTATGGTTTTTTCATGTTTTGTTATAGATACATCAAGACAGTGGATACTTTTGGTTGTGTGG
AAGGAATTCCATGGCTCATGTCTATGGGTATATCTTGTGGATGAGTCATGCCCATGTTGATACAAGAATATACT
TGTGGATAAGAAACTTATAATAACGTGCAATCCATCTAAGATAGAAAGATAGTGTACGAGCAATTTGTTGGTTT
ACTTGTTTCTAACGATGGATAAAAATATGAGTACGGAAATGAGTTGCAAAGTGCGATTTGTTGGGCCGTCTGA
ATCCGTATTTGCTATATTTGTCGACATGGTCGTTTGTTTAACTTAATCACCCATGTGAGAGCATACCCACATGAA
TTACATTAATATTTACCTGTAAATATTTTCAACCTATCGTAATTTGGGTAAGTGCTAGGTAGGACTTGAAAGTCA
GGAGACAAAACAAATAAAAATAGAAAATAATAGAGCCAATGCACACAAGTGTTTGAGACGCTAAGAAACACA
AGTAGGCAAATCAAAGATGGTCGTCTTGAAACCCGATGAGAATCTGGGTGGCACGAGTCACAAGCGACGCCA
ACAGGGGGTCGAGAGTGGTTGGGCGACACTTCTCGCTAATACCCACAGTTGGTATCCAATCTAAATGGGCCTT
AACAGCAACATTGAAATAGCCCGACACGAGGCAGACTTCCTTCGGAAAAAAACGTGAGTCGAGCAACGCGAG
TCAAACTCTCGATCCCTACTCGGCTTCGTCCGCCTTCTGACGCCCTCATCATGGTCCATCATGGCCGCGCCGTGC
TGCCCCTCTCCGTGTAGGCATGCAGCTCGATATCCTCCCTGTTGGCCTGGGCCTCCTCTCTGTTGCAGTCCCGAC
CCGACGAATCCAAGCGGCAAAAGGAATAGCGCCGCCGCCTAACACTCACACTGATGAACATCGAGGAGAAGG
CAGCTTGGATACTGGCCGAGGAATAAGAAGAGGCAAAGCTGGCTGATGAAGAAGAAGAAGCTGCACAAGCC

GAGGAGGAGGAGGAGGAAGAAGAAGAAGAAGAAGAAGAAGAAGAAGAACAAGAGGCAACGGCAGGAGA
GGTTATTTTCCGCATGTGATCTCAGTTTTGAATTTCAGGTCTCATGTTAGTCTAGTGACTACTGACTAGTGGTCT
GTAGGAGATTGTTCAT

>chr3D:471381756-471385178

TATCCAGTTTGCCCATAAGACTCATTGATGCGCATGAGAATGAGGTACAAAACGGCAGCATAGCCTTGAAGAG
AGGGAAGGGGATTAGTCAGAGAATATAAAATTTAATAAAAAAAACAACACACAAACAGAGGATATAACATGG
TGAAGTTTTTTATTAAGATAAGCGGTTAAATACCACCCACTTTCACACTCGCGGTGCCTTTATTTCACTATATTG
GTCCTCATCACACTCACTAATTTACTCTGATGCCTTATTAGTCAAAGATGACTCATCCTCGCCCTCTGGATAAGG
TTGGAAGCTCGTTTGGGTAAATTTTGGAGTGAATACACCAGACTCTTGTCCCCTGTTGTTTTTGAGGTTTCGGG
CTTCTTGCCTGGAAATGCGGTATGCATTGGCGAGGACATCCACGGGCAAGGCACGAAGAATGGAGCTCTTTCC
AGCGATGTGGCTAACCATGGAGTTTGGGTTGGTCTTGAATGAGATATACTGGCATCCTTCACGCTCCGCCTTCT
TGAGAACAACGTAGTGTTGTGGTACGATTAGCAACTGACCTTGGCGAAGACGGTCATTGAATACAGTCTGACC
ATTGTTATTGACAACTTGAACTCGAGCATGCCCTTGGATTATGTAGACCACACTGTGTGCATTGATGTTCCAGA
ATGGTGAAAGAATAGCATTCTGCAATAGCAATTATTTGTATGCCATATTATTAATCGGATGTTATGAAGCATAG
AGTATCTAGCAATAAGATAGCGCATTGAAGGTAGTATGGTATATACCTGGTATAGATTTACTCTTGTGGCGCTC
ATTTGCACGAGGTTAAGGATGGGGAAATTCTGGCCATGGAGACGTGTTATCCTACCAGCACGTGGGTTGTACG
TGTCGGCACGTTTGGGGTTTTCGATGTTCTGCCTTGCCTCCAATGAACAAAAGTTCTCCTCCAAACCATTGAAAC
TTCTGTCTTGTGACTGTCCTACCTGGTATTGAGTTGATTGTCCTTCTTGATATTGGGTTGATTGCCCTACCTGGTA
TTGGGTTGATTGTCCTTCTTGACTTTGAATTGGTTGGTAGGCTTGATGCTCTACTAGTTCTTGTTGAGACATTGT
TGGCTTCAAGAATTGAAGGTGTTGAGTCACACGAATTATCTCACCTCTTTGTTCCTTTTGACTTTGGATCCTCTG
TGCTGCTTGTTGACTTATACCAAGAGCCTCACTAAGAAGTTGGATATTGAATCCGCTGAATATGTTTTGTCCAA
ATTGTTGATCTTCCTTATTGTTACCAGCCAACAAGAACTCCTGAGAATTCATATTGATGAAGTAAATTGAAGTTC
CTTAATATCCCACTAATACATATACTTTAAAATCGTAGTTAGGTAACTAAATAAATATATATCTTTATGTGGATT
AATTTGTATAGTTACCTTTTGTCTAGGTTCAAGTTGATTAGCGTTGTTGTTTACGTCGAAGACATAGATAGCTAC
AACCGGAGCATCACCATCATTGTAGCCCCAGTGTACAATGCCAGCTGGCAGCGCAATAACATCTCCTTGTTTAA
AGCGGTGAACTCTTTGGTGCTCATCTTTGAGATGGCTTTGGCTTTGACCCTGGGCTTGATCAAATGGTTGGAAC
TGTTGTTGGAAGGTCGCTGGGCATCCAGGTAAAGTCAACCCCGTGAAACCCCTACCTAAAATAAGACACAATT
GTTTCAATATTAACAACTTATGAATAAGGTAATATTTAGAACATTTGGACTCTGAAGTGTAGTATAGCAGTGGT
ATAGCAATGCAATCACCATATTCGATTATACACGAACCTTGAAGGACGTACACCAAGCCGGGTGCGTTGTGGT
ATTGAGGTAACAAGAGGCCTTGGGGCTCAATAACACGACGGATGACAGATACCCCGGTACAACGTAATTGCTC
ATTCTGCTCATCAAAGTACTGAGTGATGCCCGCTTGTGACCTCACTTGTCGAACTGGTTCAAGTGCTTGTAGCCT
ATCGAATCTGCATCCCCTTAAACCTCCTTGCCGAGAGCTTTGCCATGGAGTAAAGCTCTGTCCAAATAGCTGAG
CCATGGATCCATGGCACAACAGGAAAATGCAGAAGTAAAACAACACGGATGAAAAACTAGTAGTTGCCATGA
TTGGTTGAAGAGGTTGTACAAGGTGGACTGGTGCTATTTCCCTAATTGTAAGCTTGGTTTTATAGCTAGAAAAA
GTAATTTCAGATGCACATGGTGACGTAATTATTAATAGAGATAAGAAGATATGCTTGGTGACTAGATGTTGTTT
AAAATGTAAATAGATATAACTTCTACTCAAAAGTTAGGTGTTGCTTTTCGCCTATAATTAGCTAGTGCTGACTCA
CAAAGCTAATACCACCGTTTGTACGTAGCTCTTGGCTATTGTGTGTACACATGATATTACTTGTACTTTCTAAAA
CAATTTTTGGCACTTTGTAACACCTTTTTATGGTTTTTTCATGTTTTGTTATAGATACATCAAGACAGTGGATACT
TTTGGTTGTGTGGAAGGAATTCCATGGCTCATGTCTATGGGTATATCTTGTGGATGAGTCATGCCCATGTTGAT
ACAAGAATATACTTGTGGATAAGAAACTTATAATAACGTGCAATCCATCTAAGTTTGAAAGATAGTGTACGAG
CAATTTGTTGGTTTACTTGTTTCTAACGATGGATAAAAATATGAGTACGGAAATGAGTTGCAAAGTGCGATTTG
TTGGGCCGTCTGAATCCGTATTTGCTATATTTGTCGACATGGTCGTTTGTTTAACTTAATCACCCATGTGAGAGC
ATACCCACATGAATTACATTAATATTTACCTGTAAATATTTTCAACCTATCGTAATTTGGGTAAGTGCTAGGTAG
GACTTGAAAGTCAGGAGACAAAACAAATAAAAATAGAAAATAATAGAGCCAATGCACACAAGTGTTTGAGAC
GCTAAGAAACACAAGTAGGCAAATCAAAGATGGTCGTCTTGAAACCCGATGAGAATCTGGGTGGCACGAGTC
ACAAGCGACGCCAACAGGGGGTCGAGAGTGGTTGGGCGACACTTCTCGCTAATACCCACAGTTGGTATCCAAT
CTAAATGGGCCTTAACAGCAACATTGAAATAGCCCGACACGAGGCAGACTTCCTTCGGAAAAAAACGTGAGTC

GAGCAACGCGAGTCAAACTCTCGATCCCTACTCGGCTTCGTCCGCCTTCTGACGCCGTCATCATGGTCCATCGT
GGCCGCGCCGTGCTGCCCCTCTCCGTGTAGGCATGCAGCTCGATATCCTCCCTGTTGGCCTGGGCCTCCTCTCT
GTTGCAGTCCCGACCCGACGAATCCAAGCGGCAAAAGGAATAGCGCCGCCGCCTAACACTCACACTGATGAAC
GTCGAGGAGAAGGCAGCTTG

>chr3D:471390326-471393243

AATTTAATAAAAAAAACAACACACAAACAGAGGATATAACATGGTGAAGTTTTTTATTAAGATAAGCGGTTAA
ATACCACCCACTTTCACACTTGCGGTGCCTTTATTTCACTATATTGGTCCTCATCACACTCACTAATTTACTCTGA
TGCCTTATTAGTCAAAGATGACTCATCCTCCCCCTCTGGATAAGGTTGGAAGCTCGTTTGGGTAAATTTTGGAG
TGAATACACCAGACTCTTGTCCCCTGTTGTTTTTGAGGTTTCGGGCTTCTTGCCTGGAAATGCGGTATGCATTG
GCGAGGACATCCACGGGCAAGGCACGAAGAATGGAGCTCTTTCCAGCGATGTGGCTAACCATGGAGTTTGGG
TTGGTCTTGAATGAGATATACTGGCATCCTTCACGCTCCGCCTTCTTGAGAACAACGTAGTGTTGTGGTACGAT
TAGCAACTGACCTTGGCGAAGACGGTCATTGAATACAGTCTGACCATTGTTATTGACAACTTGAACTCGAGCAT
GCCCTTGGATCATGTAGACCACACTGTGTGCATTGATGTTCCAGAATGGTGAAAGAATAGCATTCTGCAATAG
CAATTATTTGTATGCCATATTATTAATCGGATGTTATGAAGCATAGAGTATCTAGCAATAAGATAGCGCATTGA
AGGTAGTATGGTATATACCTGGTATAGATTTACTCTTGTGGCGCTCATTTGCACGAGGTTAAGGATGGGGAAA
TTCTGGCCATGGAGACGTGTTATCCTACCAGCACGTGGGTTGTACGTGTCGGCACGTTTGGGGTTTTCGATGTT
CTGCCTTGCCTCCAATGAACAAAAGTTCTCCTCCAAACCATTGAAACTTCTGTCTTGTGACTGTCCTACCTGGTA
TTGAGTTGATTGTCCTTCTTGATATTGGGTTGATTGCCCTACCTGGTATTGGGTTGATTGTCCTTCTTGACTTTGA
ATTGGTTGGTAGGCTTGATGCTCTACTAGTTCTTGTTGGGACATTGTTGGCTTCAAGAATTGAAGGCGTTGAGT
CACATGAATTATCTCACCTCTTTGTTCCTTTTGACTTTGGATCCTCTGTGCTGCTTGTTGACTTATACCAAGAGCC
TCACTAAGAAGTTGGATATTGAATCCGCTGAATATGTTTTGTCCAAATTGTTGATCTTCCTTATTGTTACCAGCC
AACAAGAACTCCTGAGAATTCATATTGATGAAGTAAAATGAAGTTCCTTAATATCCCACTAATACATATACTTTA
AAATCGTAGTTAGGTAACTAAATAAATATATATCTTTATGTGGATTAATTTGTATAGTTACCTTTTGTCTAGGTTT
AAGTTGATTAGCGTTGTTGTTTACGTCGAAGACATAGATAGCTACAACCGGAGCATCACCATCATTGTAGCCCC
AGTGTACAATGCCAGCTGGCAGCGCAATAACATCTCCTTGTTTAAAGCGGTGAACTCTTTGGTGCTCATCTTTG
AGATGGCTTTGGCTTTGACCCTGGGCTTGATCAAATGGTTGGAACTGTTGTTGGAAGGTCGCTGGGCATCCAG
GTAAAGTCAACCCCGTGAAACCCCTACCTAAAATAATACACAATTGTTTCAATATTAACAACTTATGAATGAGG
TAATATTTAGAACATTTGGACTCTGAAGTGTAGTATAGCAGTGGTATAGCAATGCAATCACCATATTCGATTAT
ACACGAACCTTGAAGGACGTACACCAAGCCGGGTGCGTTGTGGTATTGAGGTAACAAGAGGCCTTGGGGCTC
AATAACACGACGGATGACAGATACCCCGGTACAACGTAATTGCTCATTCTGCTCATCAAAGTACTGAGTGATGC
CCGCTTGTGACCTCACTTGTCGAACTGGTCCAAGTGCTTGTAGCCTATCGAATCTGCATCCCCTTAAACCTCCTT
GCCGAGAGCTTTGCCATGGAGTAAAGCTCTGTCCAAATAGCTGAGCCATGGATCCATGGCACAACAGGAAAAT
GCAGAAGTAAAACAACACGGATGAAAAACTAGTAGTTGCCATGATTGGTTGAAGAGGTTGTACAAGGTGGAC
TGGTGCTATTTCCCTAATTGTAAGCTTGGTTTTATAGCTAGAAAAAGTAATTTCAGATGCACATGGTGACGTAA
TTATTAAAAGAGATAAGAAGATATGCTTGGTGACTAGATGTTGTTTAAAATGTAAATAGATATAACTTCTACTC
AAAAGTTAGGTGTTGCTTTTCGCCTATAATTAGCTAGTGCTGACTCACAAAGCTAATACCACCGTTTGTACGTA
GCTCTTGGCTATTGTGTGTACACATGATATTACTTGTACTTTCTAAAACAATTTTTGGCACTTTGTAACACCTTTT
TATGGTTTTTTCATGTTTTGTTATAGATACATCAAGACAGTGGATACTTTTGGTTGTGTGGAAGGAATTCCATG
GCTCATGTCTATGGGTATATCTTGTGGATGAGTCATGCCCATGTTGATACAAGAATATACTTGTGGATAAGAAA
CTTATAATAACGTGCAATCCATCTAAGTTAGAAAGATAGTGTACGAGCAATTTGTTGGTTTACTTGTTTCTAACT
ATGGATAAAAATATGAGTACGGAAATGAGTTGCATAGTGCGATTTGTTGGGCCGTCTGAATCCGTATTTGCTA
TATTTGTCGACATGGTCGTTTGTTTAACTTAATCACCCATGTGAGAACATACCCACATGAATTACATTAATATTT
ACCTGTAAATATTTTCAACCTATCGTAATTTGGGTAAGTGCTAGGTAGGACTTGAAAGTCAGGAGACAAAACA
AATAAAAATAGAAAATAATAGAGCCAATGCACACAAGTGTTTGAGACGCTAAGAAACACAAGTAGGCAAATC
AAAGATGGTCGTCTTGAAACCCGATGAGAA

>chr3D:471398742-471401113

GATTAGTCAGAGAATATAAAATTTAATAAAAAAAACAACACACAAACAGAGGATATAACATGGTGAAGTTTTT
TATTAAGATAAGCGGTTAAATACCACCCACTTTCACACTTGCGGTGCCTTTATTTCACTATATTGGTCCTCATCAC
ACTCACTAATTTACTCTGATGCCTTATTAGTCAAAGATGACTCATCCTCCCCCTCTGGATAAGGTTGGAAGCTCG
TTTGGGTAAATTTTGGAGTGAATACACCAGACTCTTGTCCCCTGTTGTTTTTGAGGTTTCGGGCTTCTTGCCTGG
AAATGCGGTATGCATTGGCGAGGACATCCACGGGCAAGGCACGAAGAATGGAGCTCTTTCCAGCGATGTGGC
TAACCATGGAGTTTGGGTTGGTCTTGAATGAGATATACTGGCATCCTTCACGCTCCGCCTTCTTGAGAACAACG
TAGTGTTGTGGTACGATTAGCAACTGACCTTGGCGAAGACGGTCATTGAATACAGTCTGACCATTGTTATTGAC
AACTTGAACTCGAGCATGCCCTTGGATCATGTAGACCACACTGTGTGCATTGATGTTCCAGAATGGTGAAAGA
ATAGCATTCTGCAATAGCAATTATTTGTATGCCATATTATTAATCGGATGTTATGAAGCATAGAGTATCTAGCA
ATAAGATAGCGCATTGAAGGTAGTATGGTATATACCTGGTATAGATTTACTCTTGTGGCGCTCATTTGCACGAG
GTTAAGGATGGGGAAATTCTGGCCATGGAGACGTGTTATCCTACCAGCACGTGGGTTGTACGTGTCGGCACGT
TTGGGGTTTTCGATGTTCTGCCTTGCCTCCAATGAACAAAAGTTCTCCTCCAAACCATTGAAACTTCTGTCTTGT
GACTGTCCTACCTGGTATTGAGTTGATTGTCCTTCTTGATATTGGGTTGATTGCCCTACCTGGTATTGGGTTGAT
TGTCCTTCTTGACTTTGAATTGGTTGGTAGGCTTGATGCTCTACTAGTTCTTGTTGGGACATTGTTGGCTTCAAG
AATTGAAGGCGTTGAGTCACATGAATTATCTCACCTCTTTGTTCCTTTTGACTTTGGATCCTCTGTGCTGCTTGTT
GACTTATACCAAGAGCCTCACTAAGAAGTTGGATATTGAATCCGCTGAATATGTTTTGTCCAAATTGTTGATCTT
CCTTATTGTTACCAGCCAACAAGAACTCCTGAGAATTCATATTGATGAAGTAAAATGAAGTTCCTTAATATCCCA
CTAATACATATACTTTAAAATCGTAGTTAGGTAACTAAATAAATATATATCTTTATGTGGATTAATTTGTATAGT
TACCTTTTGTCTAGGTTTAAGTTGATTAGCGTTGTTGTTTACGTCGAAGACATAGATAGCTACAACCGGAGCAT
CACCATCATTGTAGCCCCAGTGTACAATGCCAGCTGGCAGCGCAATAACATCTCCTTGTTTAAAGCGGTGAACT
CTTTGGTGCTCATCTTTGAGATGGCTTTGGCTTTGACCCTGGGCTTGATCAAATGGTTGGAACTGTTGTTGGAA
GGTCGCTGGGCATCCAGGTAAAGTCAACCCCGTGAAACCCCTACCTAAAATAATACACAATTGTTTCAATATTA
ACAACTTATGAATGAGGTAATATTTAGAACATTTGGACTCTGAAGTGTAGTATAGCAGTGGTATAGCAATGCA
ATCACCATATTCGATTATACACGAACCTTGAAGGACGTACACCAAGCCGGGTGCGTTGTGGTATTGAGGTAAC
AAGAGGCCTTGGGGCTCAATAACACGACGGATGACAGATACCCCGGTACAACGTAATTGCTCATTCTGCTCAT
CAAAGTACTGAGTGATGCCCGCTTGTGACCTCACTTGTCGAACTGGTCCAAGTGCTTGTAGCCTATCGAATCTG
CATCCCCTTAAACCTCCTTGCCGAGAGCTTTGCCATGGAGTAAAGCTCTGTCCAAATAGCTGAGCCATGGATCC
ATGGCACAACAGGAAAATGCAGAAGTAAAACAACACGGATGAAAAACTAGTAGTTGCCATGATTGGTTGAAG
AGGTTGTACAAGGTGGACTGGTGCTATTTCCCTAATTGTAAGCTTGGTTTTATAGCTAGAAAAAGTAATTTCAG
ATGCACATGGTGACGTAATTATTAAAAGAGATAAGAAGATATGCTTGGTGACTAGATGTTGTTTAAAATGTAA
ATAGATATAACTTCTACTCAAAAGTTAGGTGTTGCTTTTCGCCTATAATTAGCTAGTGCTGACTCACAAAGCTAA
TACCACCGTTTGTACGTAGCTCTTGGCTATTGTGTGTACACATGATATTACTTGTACTTTCTAAAACAATTTTTG

>chr3D:471407083-471409178

GAAACAAATTCTTATCCAGTTTGCCCATAAGACTCATTGATGCGCATGAGAATGAGGTACAAAACGGCAGCAT
AGCCTTGAAGAGAGGGAAGGGGATTAGTCAGAGAATATAAAATTTAATAAAAAAAACAACACACAAACAGAG
GATATAACATGGTGAAGTTTTTATTAAGATAAGCGGTTAAATACCACCCACTTTCACACTTGCGGTGCCTTTAT
TTCACTATATTGGTCCTCATCACACTCACTAATTTACTCTGATGCCTTATTAGTCAAAGATGACTCATCCTCGCCC
TCTGGATAAGGTTGGAAGCTCGTTTGGGTAAATTTTGGAGTGAATACACCAGACTCTTGTCCCCTGTTGTTTTT
GAGGTTTCGGGCTTCTTGCCTGGAAATGCGGTATGCATTGGCGAGGACATCCACGGGCAAGGCACGAAGAAT
GGAGCTCTTTCCAGCGATGTGGCTAACCATGGAGTTTGGGTTGGTCTTGAATGAGATATACTGGCATCCTTCAC
GCTCCGCCTTCTTGAGAACAACGTAGTGTTGTGGCACGATTAGCAACTGACCTTGGCGAAGACGGTCATTGAA
TACAGTCTGACCATTGTTATTGACAACTTGAACTCGAGCATGCCCTTGGATCATGTAGACCACACTGTGTGCAT
TGATGTTCCAGAATGGTGAAAGAATAGCATTCTGCAATAGCAATTATTTGTATGCCATATTATTAATCGGATGT
TATGAAGCATAGAGTATCTAGCAATAAGATAGCGCATTGAAGGTAGTATGGTATATACCTGGTATAGATTTAC
TCTTGTGGCGCTCATTTGCACGAGGTTAAGGATGGGGAAATTCTGGCCATGGAGACGTGTTATCCTACCAGCA
CGTGGGTTGTACGTGTCGGCACGTTTGGGGTTTTCGATGTTCTGCCTTGCCTCCAATGAACAAAAGTTCTCCTC
CAAACCATTGAAACTTCTGTCTTGTGACTGTCCTACCTGGTATTGAGTTGATTGTCCTTCTTGATATTGGGTTGA

TTGCCCTACCTGGTATTGGGTTGATTGTCCTTCTTGACTTTGAATTGGTTGGTAGGCTTGATGCTCTACTAGTTC
TTGTTGGGACATTGTTGGCTTCAAGAATTGAAGGCGTTGAGTCACACGAATTATCTCACCTCTTTGTTCCTTTTG
ACTTTGGATCCTCTGTGCTGCTTGTTGACTTATACCAAGAGCCTCACTAAGAAGTTGGATATTGAATCCGCTGA
ATATGTTTTGTCCAAATTGTTGATCTTCCTTATTGTTACCAGCCAACAAGAACTCCTGAGAATTCATATTGATGA
AGTAAAATGAAGTTCCTTAATATCCCACTAATACATATACTTTAAAATCGTAGTTAGGTAACTAAATAAATATAT
ATCTTTATGTGGATTAATTTGTATAGTTACCTTTTGTCTAGGTTCAAGTTGATTAGCGTTGTTGTTTACGTCGAA
GACAAAGATAGCTACAACCGGAGCATCACCATCATTGTAGCCCCAGTGTACAATGCCAGCTGGCAGCGCAATA
ACATCTCCTTGTTTAAAGCGGTGAACTCTTTGGTGCTCATCTTTGAGATGGCTTTGGCTTTGACCCTGGGCTTGA
TCAAATGGTTGGAACTGTTGTTGGAAGGTCGCTGGGCATCCAGGTAAAGTCAACCCCGTGAAACCCCTACCTA
AAATAAGACACAATTGTTTCAATATTAACAACTTATGAATGAGGTAATATTTAGAACATTTGGACTCTGAAGTG
TAGTATAGCAGTGGTATAGCAATGCAATCACCATATTCGATTATACACGAACCTTGAAGGACGTACACCAAGCC
GGGTGCGTTGTGGTATTGAGGTAACAAGAGGCCTTGGGGCTCAATAACACGACGGATGACAGATACCCCGGT
ACAACGTAATTGCTCATTCTGCTCATCAAAGTACTGAGTGATGCCCGCTTGTGACCTCACTTGTCGAACTGGTTC
AAGTGCTTGTAGCCTATCGAATATGCATCCCCTTAAACCTCCTTGCCGAGAGCTTTGCCATGGAGTAAAGCTCT
GTCCAAATAGCTGAGCCTTGGATC

>chr3D:471415483-471417678

TCTAGGAAACAAATTCTTATCCAGTTTGCCCATAAGACTCATTGATGCGCATGAGAATGAGGTACAAAACGGC
AGCATAGCCTTGAAGAGAGGGAAGGGGATTAGTCAGAGAATATAAAATTTAATAAAAAAAACAACACACAAA
CAGAGGATATAACATGGTGAAGTTTTTATTAAGATAAGCGGTTAAATACCACCCACTTTCACACTTGCGGTGC
CTTTATTTCACTATATTGGTCCTCATCACACTCACTAATTTACTCTGATGCCTTATTAGTCAAAGATGACTCATCC
TCGCCCTCTGGATAAGGTTGGAAGCTCGTTTGGGTAAATTTTGGAGTGAATACACCAGACTCTTGTCCCCTGTT
GTTTTTGAGGTTTCGGGCTTCTTGCCTGGAAATGCGGTATGCATTGGCGAGGACATCCACGGGCAAGGCACGA
AGAATGGAGCTCTTTCCAGCGATGTGGCTAACCATGGAGTTTGGGTTGGTCTTGAATGAGATATACTGGCATC
CTTCACGCTCCGCCTTCTTGAGAACAACGTAGTGTTGTGGTACGATTAGCAACTGACCTTGGCGAAGACGGTCA
TTGAATACAGTCTGACCATTGTTATTGACAATTTGAACTCGAGCATGCCCTTGGATCATGTAGACCACACTGTG
TGCATTGATGTTCCAGAATGGTGAAAGAATAGCATTCTGCAATAGCAATTATTTGTATGCCATATTATTAATCG
GATGTTATGAAGCATAGAGTATCTAGCAATAAGATAGCGCATTGAAGGTAGTATGGTATATACCTGGTATAGA
TTTACTCTTGTGGTGCTCATTTGCACGAGGTTAAGGATGGGGAAATTCTGGCCATGGAGACGTGTTATCCTACC
AGCACGTGGGTTGTACGTGTCGGCACGTTTGGGGTTTTCGATGTTCTGCCTTGCCTCCAATGAACAAAAGTTCT
CCTCCAAACCATTGAAACTTCTGTCTTGTGACTGTCCTACCTGGTATTGAGTTGATTGTCCTTCTTGATATTGGG
TTGATTGCCCTACCTGGTATTGGGTTGATTGTCCTTCTTGACTTTGAATTGGTTGGTAGGCTTGATGCTCTACTA
GTTCTTGTTGGGACATTGTTGGCTTCAAGAATTGAAGGCGTTGAGTCACACGAATTATCTCACCTCTTTGTTCCT
TTTGACTTTGGATCCTCTGTGCTGCTTGTTGACTTATACCAAGAGCCTCACTAAGAAGTTGGATATTGAATCCGC
TGAATATGTTTTGTCCAAATTGTTGATCTTCCTTATTGTTACCAGCCAACAAGAACTCCTGAGAATTCATATTGA
TGAAGTAAAATGAAGTTCCTTAATATCCCACTAATACATATACTTTAAAATCGTAGTTAGGTAACTAAATAAATA
TATATCTTTATGTGGATTAATTTGTATAGTTACCTTTTGTCTAGGTTCAAGTTGATTAGCGTTGTTGTTTACGTCG
AAGACATAGATAGCTACAACCGGAGCATCACCATCATTGTAGCCCCAGTGTACAATGCCAGCTGGCAGCGCAA
TAACATCTCCTTGTTTAAAGCGGTGAACTCTTTGGTGCTCATCTTTGAGATGGCTTTGGCTTTGACCCTGGGCTT
GATCAAATGGTTGGAACTGTTGTTGGAAGGTCGCTGGGCATCCAGGTAAAGTCAACCCCGTGAAACCCCTACC
TAAAATAAGACACAATTGTTTCAATATTAACAACTTATGAATGAGGTAATATTTAGAACATTTGGACTCTGAAG
TGTAGTATAGCAGTGGTATAGCAATGCAATCACCATATTCGATTATACACGAACCTTGAAGGACGTACACCAA
GCCGGGTGCGTTGTGGTATTGAGGTAACAAGAGGCCTTGGGGCTCAATAACACGACGGATGACAGATACCCC
GGTACAACGTAATTGCTCATTCTGCTCATCAAAGTACTGAGTGATGCCCGCTTGTGACCTCACTTGTCGAACTG
GTTCAAGTGCTTGTAGCCTATCGAATCTGCATCCCCTTAAACCTCCTTGCCGAGAGCTTTGCCATGGAGTAAAG
CTCTGTCCAAATAGCTGAGCCATGGATCTATGGCACAACAGGAAAATGCAGAAGTAAAACAACACGGATGAA
AAACTAGTAGTTGCCATGATTGGTTGAAGAGGTTGTACAAGGTGGACTGGT

>chr3D:471424072-471426178

AGGATATAACATGGTGAAGTTTTTTATTAAGATAAGCAGTTAAATACCACCCACTTTCACACTTGCGGTGCCTTT
ATTTCACTATATTGGTCCTCATCACACTCACTAATTTACTCTGATGCCTTATTAGTCAAAGATGACTCATCCTCGC
CCTCTGGATAAGGTTGGAAGCTCGTTTGGGTAAATTTTGGAGTGAATACACCAGACTCTTGTCCCCTGTTGTTT
TTGAGGTTTCGGGCTTCTTGCCTGGAAATGCGGTATGCATTGGCGAGGACATCCACGGGCAAGGCACGAAGA
ATGGAGCTCTTTCCAGCGATGTGGCTAACCATGGAGTTTGGGTTGGTCTTGAATGAGATATACTGGCATCCTTC
ACGCTCCGCCTTCTTGAGAACAACGTAGTGTTGTGGTACGATTAGCAACTGACCTTGGCGAAGACGGTCATTG
AATACAGTCTGACCATTGTTATTGACAACTTGAACTCGAGCATGCCCTTGGATCATGTAGACCACACTGTGTGC
ATTGATGTTCCAGAATGGTGAAAGAATAGCATTCTGCAATAGCAATTATTTGTATGCCATATTATTAATCGGAT
GTTATGAAGCATAGAGTATCTAGCAATAAGATAGCGCATTGAAGGTAGTATGGTATATACCTGATATAGATTT
ACTCTTGTGGCGCTCATCTGCACGAGGTTAAGGATGGGGAAATTCTGGCCATGGAGACGTGTTATCCTACCAG
CACGTGGGTTGTACGTGTCGGCACGTTTGGGGTTTTCGATGTTCTGTCTTGCCTCCAATGAACAAAAGTTCTCC
TCCAAACCATTGAAACTTCTGTCTTGTGACTGTCCTATCTGGTATTGAGTTGATTGTCCTTCTTGATATTGGGTT
GATTGCCCTACCTGGTATTGGGTTGATTGTCCTTCTTGACTTTGAATTGGTTGGTAGGCTTGATGCTCTACTAGT
TCTTGTTGGGACATTGTTGGCTTCAAGAATTGAAGGCGTTGAGTCACACGAATTATCTCACCTCTTTGTTCCTTT
TGACTTTGGATCCTCTGTGCTGCTTGTTGACTTATACCAAGAGCCTCACTAAGAAGTTGGATATTGAATCCGTTG
AATATGTTTTGTCCAAATTGTTGATCTTCCTTATTGTTACCAGCCAACAAGAACTCCTGAGAATTCATATTGATG
AAGTAAAATGAAGTTCCTTAATATCCCACTAATACATATACTTTAAAATCGTAGTTAGGTAACTAAATAAATATA
TATCTTTATGTGGATTAATTTGTATAGTTACCTTTTGTCTAGGTTCAAGTTGATTAGCGTTGTTGTTTACGTCGAA
GACATAGATAGCTACAACCGTAGCATCACCATCATTGTAGCCCCAGTGTACAATGCCAGCTGGCAGCGCAATA
ACATCTCCTTGTTTAAAGCGGTGAACTCTTTGGTGCTCATCTTTGAGATGGCTTTGGCTTTGACCCTGGGCTTGA
TCAAATGGTTGGAACTGTTGTTGGAAGGTCGCTGGGCATCCAGGTAAAGTCAACCCCGTGAAACCCCTACCTA
AAATAAGACACAATTGTTTCAATATTAACAACTTATGAATGAGGTAATATTTAGAACATTTGGACTCTGAAGTG
TAGTATAGCAGTGGTATAGCAATGCAATCACCATATTCGATTATACACGAACCTTGAAGGACGTACACCAAGCC
GGGTGCGTTGTGGTATTGAGGTAACAAGAGGCCTTGGGGCTCAATAACATGACGGATGACAGATACCCCGGT
ATAACGTAATTGCTCATTCTGCTCATCAAAGTACTGAGTGATGCCCGCTTGTGACCTCACTTGTCGAACTGGTTC
AAGTGCTTGTAGCCTATCGAATCTGCATCCCCTTAAACCTCCTTGCCGAGAGCTTTGCCATGGAGTAAAGCTCT
GTCCAAATAGCTGAGCCATGGATCCATGGCACAACAGGAAAATACAGAAGTAAAACAACACGGATGAAAAAC
TAGTAGTTGCCATGATTGGTTGAAGAGGTTGTACAAGGTGGACTGGTGCTATTTCCCTAATTGTAAGCTTGGTT
TTATAGCTAGAAAAAGTAATTTCAGATGCACA

>chr3D:471439255-471442255

TCATCACACTCACTAATTTACTCTGATGCCTTATTAGTCAAAGATGACTCATCCTCGCCCTCTGGATAAGTTTGG
AAGCTCGTTTGGGTAAATTTTGGAGTGAATACACCAGACTCTTGTCCCCTGTTGTTTTTGAGGTTTCGGGCTTCT
TGCCTGGAAATGCGGTATGCATTGGCGAGGACATCCACGGGCAAGGCACGAAGAATGGAGCTCTTTCCAGCG
ATGTGGCTAACCATGGAGTTTGGGTTGGTCTTGAATGAGATATACTGGCATCCTTCACGCTCCGCCTTCTTGAG
AACAACGTAGTGTTGTGGTACGATTAGCAACTGACCTTGGCGAAGACGGTCATTGAATACAGTCTGACCATTG
TTATTGACAACTTGAACTCGAGCATGCCCTTGGATCATGTAGACCACACTGTGTGCATTGATGTTCCAGAATGG
TGAAAGAATAGCATTCTGCAATAGCAATTATTTGTATGCCATATTATTAATCGGATGTTATGAAGCATAGAGTA
TCTAGCAATAAGATAGCGCATTGAAGGTAGTATGGTATATACCTGGTATAGATTTACTCTTGTGGTGCTCATTT
GCACGAGGTTAAGGATGGGGAAATTCTGGCCATGGAGACGTGTTATCCTACCAGCACGTGGGTTGTACGTGT
CGGCACGTTTAGGGTTTTCGATGTTCTGCCTTGCCTCCAATGAACAAAAGTTCTCCTTCAAACCATTGAAACTTC
TGTCTTGTGACTGTCCTACCTGGTATTGAGTTGATTGTCCTTCTTGATATTGGGTTGATTGCCCTACCTGGTATT
GGGTTGATTGTCCTTCTTGACTTTGAATTGGTTGGTAGGCTTGATGCTCTACTAGTTCTTGTTGGGACATTGTTG
GCTTCAAGAATTGAAGGCGTTGAGTCACACGAATTATCTCACCTCTTTGTTCCTTTTGACTTTGGATCCTCTGTG
CTGCTTGTTGACTTATACCAAGAGCCTCACTAAGAAGTTGGATATTGAATCCGCTGAATATGTTTTGTCCAAATT
GTTGATCTTCCTTATTGTTACCAGCCAACAAGAACTCCTGAGAATTCATATTGATGAAGTAAAATGAAGTTCCTT

AATATCCCACTAATACATATACTTTAAAATCGTAGTTAGGTAACTAAATAAATATATATCTTTATGTGGATTAAT
TTGTATAGTTACCTTCTGTCTAGGTTCAAGTTGATTAGCGTTGTTGTTTACGTCGAAGACATAGATAGCTACAAC
CGGAGCATCACCATCATTGTAGCCCTAGTGTACAATGCCAGCTGGCAGCGCAATAACATCTCCTTGTTTAAAGC
GGTGAACTCTTTGGTGCTCATCTTTGAGATGGCTTTGGCTTTGACCCTGGGCTTGATCAAATGGTTGAAACTGT
TGTTGGAAGGTCGCTGGGCATCCAGGTAAAGTCAACCCCGTGAAACCCCTACCTAAAATAAGACACAATTGTT
TCAATATTAACAACTTATGAATGAGGTAATATTTAGAACATTTGGACTCTGAAGTGTAGTATAGCAGTGGTATA
GCAATGCAATCACCATATTCGATTATACACGAACCTTGAAGGACGTACACCAAGCCGGGTGCGTTGTGGTATT
GAGGTAACAAGAGGCCTTGGGGCTCAATAACATGACGGATGACAGATACCCCGGTACAACGTAATTGCTCATT
CTGCTCATCAAAGTACTGAGTGATGCCCGCTTGTGACCTCACTTGTCGAACTGGTTCAAGTGCTTGTAGCCTAT
CGAATCTGCATCCCCTTAAACCTCCTTGCCGAGAGCTTTGCCATGGAGTAAAGCTCTGTCCAAATAGCTGAGCC
ATGGATCCATGGCACAACAGGAAAATGCAGAAGTAAAACAACACGGATGAAAAACTAGTAGTTGCCATGATT
GGTTGAAGAGGTTGTACAAGGTGGACTGGTGCTATTTCCCTAATTGTAAGCTTGGTTTTATAGCTAGAAAAAG
TAATTTCAGATGCACATGGTGACGTAATTATTAATAGAGATAAGAAGATATGCTTGGTGACTAGATGTTGTTTA
AAATGTAAATAGATATAACTTCTACTCAAAAGTTAGGTGTTGCTTTTCGCCTATAATTAGCTAGTGCTGACTCAC
AAAGCTAATACCACCGTTTGTACGTAGCTCTTGGCTGTTGTGTGTACACATGATATTACTTGTACTTTCTAAAAC
AATTTTTGGCACTTTGTAACACCTTTTTATGGTTTTTTCATGTTTTGTTATAGATACATCAAGACAGTGGATACTT
TTGGTTGTGTGGAAGGAATTCCATGGCTCATGTCTATGGGTATATCTTGTGGATGAGTCATGCCCATGTTGATA
CAAGAATATACTTGTGGATAAGAAACTTATAATAACGTGCAATCCATCTAAGTTAGAAAGATAGTGTACGAGC
AATTTGTTGGTTTACTTGTTTCTAACTATGGATAAAAATATGAGTACGGAAATGAGTTGCAAAGTGCGATTTGT
TGGGCCGTCTGAATCCGTATTTGCTATATTTGTCGACATGGTCGTTTGTTTAACTTAATCACCCATGTGAGAGCA
TACCCACATGAATTACATTAATATTTACCTGTAAATATTTTCAACCTATCGTAATTTGGGTAAGTGCTAGGTAGG
ACTTGAAAGTCAGGAGACAAAACAAATAAAAATAGAAAATAATAGAGCCAATGCACACAAGTGTTTGAGACG
CTAAGAAACACAAGTAGGCAAATCAAAGATGGTCGTCTTGAAACCCGATGAGAATCTGGGTGGCACGAGTCA
CAAGCGACGCCAACAGGGGGTCGAGAGTGGTTGGGCGACACTTCTCGCTAATACCCACAGTTGGTATCCAATC
TAAATGGGCCTTAACAGCAACATTGAAATAGCCCGACACGAGGCAGACTTCCTTCGGAAAAAAACGTGAGTCG
AGCAACGCGAGTCAAACTCTCGATCCCTACTCGGCTTCGTCC

>chr3D:471447486-471449678

TTGCCCATAAGACTCATTGATGCGCATGAGAATGAGGTACAAAACGGCAGCATAGCCTTGAAGAGAGGGAAG
GGGATTAGTCAGAGAATATAAAATTTAATAAAAAAAACAACACACAAACAGAGGATATAACATGGTGAAGTTT
TCTATTAAGATAAGCGGTTAAATACCACCCACTTTCACACTTGCGGTGCCTTTATTTCACTATATTGGTCCTCATC
ACACTCACTAATTTACTCTGATGCCTTATTAGTCAAAGATGACTCATCCTCGCCCTCTGGATAAGGTTGGAAGCT
CGTTTGGGTAAATTTTGGAGTGAATACACCAGACTCTTGTCCCCTGTTGTTTTTGAGGTTTCGGGCTTCTTGCCT
GGAAATGCGGTATGCATTGGCGAGGACATCCACGGGCAAGGCACGAAGAATGGAGCTCTTTCCAGCGATGTG
GCTAACCATGGAGTTTGGGTTGGTCTTGAATGAGATATACTGGCATCCTTCACGCTCCGCCTTCTTGAGAACAA
CGTAGTGTTGTGGTACGATTAGCAACTGACCTTGGCGAAGACGGTCATTGAATACAGTCTGACCATTGTTATTG
ACAACTTGAACTCGAGCATGCCCTTGGATCATGTAGACCACACTGTGTGCATTGATGTTCCAGAATGGTGAAA
GAATAGCATTCTGCAATAGCAATTATTTGTATGCCATATTATTAATCGGATGTTATGAAGCATAGAGTATCTAG
CAATAAGATAGCGCATTGAAGGTAGTATGGTATATACCTGGTATAGATTTACTCTTGTGGTGCTCATTTGCACG
AGGTTAAGGATGGGGAAATTCTGGCCATGGAGACGTGTTATCCTACCAGCACGTGGGTTGTACGTGTCGGCA
CGTTTGGGGTTTTCGATGTTCTGCCTTGCCTCCAATGAACAAAAGTTCTCCTCCAAACCATTGAAACTTCTGTCT
TGTGACTGTCCTACCTGGTATTGAGTTGATTGTCCTTCTTGATATTGGGTTGATTGCCCTACCTGGTATTGGGTT
GATTGTCCTTCTTGACTTTGAATTGGTTGGTAGGCTTGATGCTCTACTAGTTCTTGTTGGGACATTGTTGGCTTC
AAGAATTGAAGGCGTTGAGTCACACGAATTATCTCACCTCTTTGTTCCTTTTGACTTTGGATCCTCTGTGCTGCT
TGTTGACTTATACCAAGAGCCTCACTAAGAAGTTGGATATTGAATCCGCTGAATATGTTTTGTCCAAATTGTTG
ATCTTCCTTATTGTTACCAGCCAACAAGAACTCCTGAGAATTCATATTGATGAAGTAAAATGAAGTTCCTTAATA
TCCCACTAATACATATACTTTAAAATCGTAGTTAGGTAACTAAATAAATATATATCTTTATGTGGATTAATTTGT
ATAGTTACCTTTTGTCTAGGTTCAAGTTGATTAGCGTTGTTGTTTACGTCGAAGACATAGATAGCTACAACCGG

AGCATCACCATCATTGTAGCCCCAGTGTACAATGCCAGCTGGTAGCACAATAACATCTCCTTGTTTAAAGCGGT
GAACTCTTTGGTGCTCATCTTTGAGATGGCTTTGGCTTTGACCCTGGGCTTGATCAAATGGTTGGAACTGTTGT
TGGAAGGTCGCTGGGCATCCAGGTAAAGTCAACCCCGTGAAACCCCTACCTAAAATAAGACACAATTGTTTCA
ATATTAACAACTTATGAATGAGGTAATATTTAGAACATTTGGACTCTGAAGTGTAGTATAGCAGTGGTATAGCA
ATGCAATCACCATATTCGATTATACACGAACCTTGAAGGACGTACACCAAGCCGGGTGCGTTGTGGTATTGAG
GTAACAAGAGGCCTTGGGGCTCAATAACACGACGGATGACAGATACCCCGGTACAACGTAATTGCTCATTCTG
CTCATCAAAGTACTGAGTGATGCCCGCTTGTGACCTCACTTGTCGAACTGGTTCAAGTGCTTGTAGCCTATCGA
ATCTGCATCCCCTTAAACCTCCTTGCCGAGAGCTTTGCCATGGAGTAAAGCTCTGTCCAAATAGCTGAGCCATG
GATCCATGGCACAACAGGAAAATGCAGAAGTAAAACAACACGGATGAAAAACTAGTAGTTGCCATGATTGGT
TGAAGAGGTTGTACAAGGTGGACTGGTGCTATTTCCCTAATTGTAAGCT

>chr3D:471456030-471459030

AGGATATAACATGGTGAAGTTTTTATTAAGATAAGCAGTTAAATACCACCCACTTTCACACTTGCGGTGCCTTT
ATTTCACTATATTGGTCCTCATCACACTCACTAATTTACTCTGATGCCTTATTAGTCAAAGATGACTCATCCTCGC
CCTCTGGATAAGGTTGGAAGCTCGTTTGGGTAAATTTTGGAGTGAATACACCAGACTCTTGTCCCCTGTTGTTT
TTGAGGTTTCGGGCTTCTTGCCTGGAAATGCGGTATGCATTGGCGAGGACATCCACGGGCAAGGCACGAAGA
ATGGAGCTCTTTCCAGCGATGTGGCTAACCATGGAGTTTGGGTTGGTCTTGAATGAGATATACTGGCATCCTTC
ACGCTCCGCCTTCTTGAGAACAACGTAGTGTTGTGGTACGATTAGCAACTGACCTTGGCGAAGACGGTCATTG
AATACAGTCTGACCATTGTTATTGACAACTTGAACTCGAGCATGCCCTTGGATCATGTAGACCACACTGTGTGC
ATTGATGTTCCAGAATGGTGAAAGAATAGCATTCTGCAATAGCAATTATTTGTATGCCATATTATTAATCGGAT
GTTATGAAGCATAGAGTATCTAGCAATAAGATAGCGCATTGAAGGTAGTATGGTATATACCTGATATAGATTT
ACTCTTGTGGCGCTCATCTGCACGAGGTTAAGGATGGGGAAATTCTGGCCATGGAGACGTGTTATCCTACCAG
CACGTGGGTTGTACGTGTCGGCACGTTTGGGGTTTTCGATGTTCTGTCTTGCCTCCAATGAACAAAAGTTCTCC
TCCAAACCATTGAAACTTCTGTCTTGTGACTGTCCTACCTGGTATTGAGTTGATTGTCCTTCTTGATATTGGGTT
GATTGCCCTACCTGGTATTGGGTTGATTGTCCTTCTTGACTTTGAATTGGTTGGTAGGCTTGATGCTCTACTAGT
TCTTGTTGGGACATTGTTGGCTTCAAGAATTGAAGGCGTTGAGTCACACGAATTATCTCACCTCTTTGTTCCTTT
TGACTTTGGATCCTCTGTGCTGCTTGTTGACTTATACCAAGAGCCTCACTAAGAAGTTGGATATTGAATCCGTTG
AATATGTTTTGTCCAAATTGTTGATCTTCCTTATTGTTACCAGCCAACAAGAACTCCTGAGAATTCATATTGATG
AAGTAAAATGAAGTTCCTTAATATCCCACTAATACATATACTTTAAAATCGTAGTTAGGTAACTAAATAAATATA
TATCTTTATGTGGATTAATTTGTATAGTTACCTTTTGTCTAGGTTCAAGTTGATTAGCGTTGTTGTTTACGTCGAA
GACATAGATAGCTACAACCGTAGCATCACCATCATTGTAGCCCCAGTGTACAATGCCAGCTGGCAGCGCAATA
ACATCTCCTTGTTTAAAGCGGTGAACTCTTTGGTGCTCATCTTTGAGATGGCTTTGGCTTTGACCCTGGGCTTGA
TCAAATGGTTGGAACTGTTGTTGGAAGGTCGCTGGGCATCCAGGTAAAGTCAACCCCGTGAAACCCCTACCTA
AAATAAGACACAATTGTTTCAATATTAACAACTTATGAATGAGGTAATATTTAGAACATTTGGACTCTGAAGTG
TAGTATAGCAGTGGTATAGCAATGCAATCACCATATTCGATTATACACGAACCTTGAAGGACGTACACCAAGCC
GGGTGCGTTGTGGTATTGAGGTAACAAGAGGCCTTGGGGCTCAATAACATGACGGATGACAGATACCCCGGT
ACAACGTAATTGCTCATTCTGCTCATCAAAGTACTGAGTGATGCCCGCTTGTGACCTCACTTGTCGAACTGGTTC
AAGTGCTTGTAGCCTATCGAATCTGCATCCCCTTAAACCTCCTTGCCGAGAGCTTTGCCATGGAGTAAAGCTCT
GTCCAAATAGCTGAGCCATGGATCCATGGCACAACAGGAAAATACAGAAGTAAAACAACACGGATGAAAAAC
TAGTAGTTGCCATGATTGGTTGAAGAGGTTGTACAAGGTGGACTGGTGCTATTTCCCTAATTGTAAGCTTGGTT
TTATAGCTAGAAAAAGTAATTTCAGATGCACATGGTGACGTAATTATTAATAGAGATAATAAGATATGCTTGGT
GACTAGATGTTGTTTAAAATGTAAATAGATATAACTTCTACTCAAAAGTTAGGTGTTGCTTTTCGCCTATAATTA
GCTAGTGCTGACTCACAAAGCTAATACCACCGTTTGTACGTAGCTCTTGGCTATTGTGTGTACACATGATATTAC
TTGTACTTTCTAAAACAATTTTTGGCACTTTGTAACACCTTTTTATGGTTTTTTCATGTTTTGTTATAGACACATCA
AGACAGTGGATACTTTTGGTTGTGTGGAAGGAATTCCATGGCTCATGTCTATGGGTATATCTTATTGATGAGTC
ATGCCCATGTTGATACAAGAATATACTTGTGGATAAGAAATTTATAATAACGTGCAATCCATCTAAGTTAGAAA
GATAGTGTACGAGCAATTTGTTGGTTTACTTGTTTCTAATGACGGATAAAAATATGAGTACGGAAATGAGTTGC
AAAGTGCGATTCGTTGGGCCGTCTGAATCCGTATTTGCTATATTTGTCAACATGGTCGTTTGTTTAACTTAATCA

CCCATGTGAGAGCATACCCACATGAATTACATTAATATTTACCTGTAAATATTTTCAACCTATCGTAATTTGGGT
AAGTGCTAGGTAGGACTTGAAAGTCAGGAGACAAAACAAATAAAAATAGAAAATAATAGAGCCAATGCACAC
AAGTGTTTGAGACGCTAAGAAACACAAGTAGGCAAATCAAAGATGGTCGTCTTGAAACCCGATGAGAATCTA
GGTGGCACGAGTCAAAAACGACGCCAACAGGGGGTCGAGAGGGGTTGGGCGACACTTCTCGCTAATACCCAC
AGTTGGTATCCAATCTAAATGGGCCTTAACAGCAACA

>chr3D:472771859-472775039

GTTTTTCTAGCTATAAAACCAAGCTTACAATTAGGAAAATAGCACAAGTCCACCTTGTACAACCTCTTCAACCAA
TCATGGCAACTACTAGTTTTTCATCCGTGTTGTTTTACTTCTGCATTTTTCTGTTGTGCCATGGATCCATGGCTCA
GCTATTCGGACAGAGCTTTACTCCATGGCAAAGCTCTCGGCAAGGAGGTTTAAGGGGGTGCAGATTCGATCG
GCTACAAGCACTTGAACCAGTTCGACAAGTGAGGTCACAAGCGGGCATCACTCAATACTTTGATGAGCACAAT
GAGCAATTACGTTGTGCCGGGGTATCTGTCATCCGTCGTGTTATTGAGCCCCAAGGCCTCTTGTTACCTCAATA
CCACAATGCACCAGGCTTGGTGTACGTCCTTCAAGGTTCGTGTATAGTCGAATATGGTGATGGCATTGCTATAC
CATTGCTATACTACACTTTAGAGTCCAAATGTTCCAAATATTACCTCATTCATAAGTTGTTAATATTGAAACAATT
GTGTCTTATTTTAAGTAGGGGTTTCACGGGGTTGACTTTACCTGGATGCCTAGCAACCTTCCAACAACAGTTCC
AACCATTTGATCAAGCCTAGGGTCAAAGCCAAAGCCATCTCAAAGATGAGCACCAAAGAGTTCACCGCTTTAA
ACAAGGAGATGTTATTGCGCTGCCAGCTGGCATTGTACACTGGGGCTACAATGATGGTGATGCGCCGGTTGTA
GCTATCTATGTCTTCGACGTAAACAACAACGCTAATCATCTTGAACCTAGACAAAAGGTAACTATACAAATTAA
TCTACATAAAGATATATAATTCTTTAGTTACCTAACTTTGATTTTAAAGTATATATTAGTGGGATATTAAGGAAC
TTCGTTTTACTTCATCAATATAAAATCTCAGGAATTCTTGTTAGCTGGTAACAACAAGGTAGATCAACAAGTTG
GACAAAACATATTCAGCGGATTCAATATCCAACTTCTTAGTGAGGCTCTTGGTATAAGCCAACAACTAGCACAG
AGGATCCAGAGTCAAAAAGACCAAAGGGGTGAGATAATTCATATGAGTCAAGGCCTTCGGTTCTTGAAGCCA
ACAGTGTCCCAACAAGGACCAGTAGAGCATCAAGCCTACTAACCAATTCAAAGTCAAGAAGGACAGTCAACCC
AATACCAGGTAGGGCAATCAACCCAATATCAAGAAGGACAATCAACTCAATACCAGGTAGGACAGTCACAAG
ATAGAAGTTTCAATGGTTTGGAGGAGAACTTTTGTTCATTGGAGGCAAGTCAGAACATCGAAAACCCCAAACG
TGCCGACACGTACAACCCACGTGCTGGCAGGATCACACGTCTCCATGGCCAGAATTTCCCCATCCTTAACCTTG
TGCAAATGAGCGCCACAAGAGTAAATCTATACCAGGTATATACCATACTACCTTCAATGTATTATCTTATTGTTA
GATAGTCTAAGCTTCATAACATCCGATTAATAATATGGCATACAAATAATTGCTATTGCAGAATGCTGTTCTTTC
ACCATTCTGGAACATCAATGCACACAGTGTGGTCTACATGATCCAAGGGCATGCTCGAGTTCAAGTTGTCAATA
ACAATGGTCAGACTGTATTCAATGACCGTCTTCGCCAAGGGCAGTTGCTAATCGTACCACAACATTACGTTGTT
CTCAAGAAGGCGGAGCGTGAAGGATGCCAATATATCTCCTTCAAGACCAACCCAAACTCCATGGTTAGCCACA
TCGCAGGAAAGAGCTCCATCCTTCGTGCCCTACCCGTTGATGTCCTCGCCAATGCATACCGTATTTCTAGGCAG
GAAGCTCGGAACCTCAAAAACAACAGGGGAGAAGAGTCCGGTGTATTCACTCCAAAATTTACCCAAACAAGCT
TCCAACCTTATCCAGAGGACGAGTATGAGTCATCTTTTACTAATAAGGCATCAGAGTAAATTAGTGCGTGTGAT
GAGGACCAATATAGTGAAATAAAGGCATCCCGAGTGTGGAAGTTGGTGGTGTATAGCCGCTTATCTTAATAAA
AAAACTTCATCATGTTATATCCTTTGTTTTTGTCTTGTTCTTTATTTAATTTTATATTCTCTGACTAATCCCCTTCCC
TCTTTAATTTGCTGCCATTTTGTACCGCATTCTGATGCGCACCAAGCCACCAACGAGTTATATGGGCCAACTAGA
CAAGAATTTGTTTCCAGAAATGTTTTTTGTGGTTCGAGTATTTTTTAGGTCCTACCAATGACTCCAGTTTTCCAAT
ATCCTAGCCGCCTGCAACTAAACCTTTGTGTGTTAAAATTGTGCAGCTTTGATAAGCAAAACTGTAATTTTCGGT
CCATAGATTAGTGTGATCTTTTACACTGGATTAAGCAGCTCTTAACCCAGTCAAAAAAAAGCAGCTCTTAACAC
ATATATGGCTGACGGTATCTTCGAGTCCAACTGCCGTCCGATGTATTGTTCATTGGGAGAGAGACAGTTTATTT
CTACGACAGCCTAACAGTCCAACGGTGCCACATAACGCTTGCTGGGATGGGCTGGTCGGGCCTATGTCGGTGT
AGCATTGCACCAGGCCAAACAGCCGTTAACGAACCCCACGGGTCCTGTTGGTCTGTGCCGACGGCCAGTCTGG
GCCTTCGGCCCACCTCGCTACGCCGAAGGCAGCGCCTATACCGATGGCTATGTGGGCTACGTCGTCAAGGGCT
TGTGCCGAGGGGGATACAGCAACAGCTGCCCTCGGCACAACCTGTACCGACGGCAATCCTAGGTTGTGTCGA
GGGTGGCCGGCCCTTGGCGCCTCGCACGTTTCCTGTAGTGTATGTATGATTACCTAGTTTTGAAAGGATATACA
TAGGGATAAGTTTTGGCAACTAAATGTATTAAAAAGACAAAGTGAAATGCTAAAAAGAAGTACGGTACAAAA
CATGTAGTCGGTTCCATCATATTGTTTACATTTCCTCCAAATTTTACGAGCTTGTCTATCGACATATAGGCACTTT

GGTTTACTACTCCATCCAAAGTACAATCATGCCGATTGGTCATCACAACCCTTTTGCCCTTGTCTAGGTCACCAC
GTGTTGTTGTTGCAATGGCGACCTTGGCAATTGCCTAGGGCCCTTGTCTAGGAGGGGCCCATAATTTATACAG
G

>chr3D:476356165-476358603

AGAATATAAAATTAAATAAAGAACAAGACACAAACAAAGGATGTAACATGATGAAGTTTTTTATTAAGATAAG
CGGTTATATACCACCCACCTCCACACTTATGATGCCTTTATTTCACTATATTGATCCTCACACACTCACGAATTCA
CTCGGATGCTTTATTAGTCAAGAATGACTCATCCTCCGCCGGATCACGTTGGAAGCTCGTCTGGGTAAATTTTG
GAGTGAATACACCAAACTCTTCTCCCCTGTTATTTTTTAGGCTTCGGGCTTCCTGCCTGGAAATGCGGTACGCAT
TGGCGAGGACATCCACAGGCAAGGCACGCAGGATGGAGCTCTTTCCTGCGATATGGCTAACCATGGAGTTCG
GATTGGTCTTGAATGAGATATACTGGCATCCTTCACGCTCCGCCTTCTTGAGAACAACATAGTTTTGCGGTATG
ATTAGCAACTGCCCTTGGCGAAGACGGTCATTGAATACAGTCTGACCATTGTTACTGACAACTTGAACTCGAAC
ATGTCCTTGGATCATGTAGACAACACTGTGTGCATTGATATTTCAGAATGGTGAAAGAATGGCATTCTGCAATA
GAAATTATTTATATGCCATATTATTGACTGGATGTTATGAAGCTTAGAGTATCTAACAATAAGAGAGTGTACTG
AAGGTAATATCGTATGTACCTGGTATAGATTTACTCTTGTAGCGCTCATTTGCACGAGGTTAAGGATGGGGAA
ATTGTTTCCATGGAGACGTGTTATCCTACCAGCACATGGGTTGTATGTGTCGGCACGTTTGGGGTTTTCGATGT
TCTGTCTTGCCTCCAATGCACAAAAGTTCTCCTCCAAACCATTGAAACTTTTGTCTGATGACTGCCCTATCTGGT
ATTGAGTTGATGGTCCTTCTTGATATTGGGTTGATTGCCCTCCCTGGTATTGGGTTGACTGCCCTTCTTGACTTT
GAATTGGTTGGTAGGATTGCCCTTGTTGTGACACCGTGGGCTTCAAGAACTGAAGGCCTTGACTCACACGAAT
TATCTCACCTCTTTGGTCATTTTGACTCTGGATCCTTTGTGCTGCTTGTTGACTTATACCAAGAGCCGCACTAAG
AAGTTGGATACTGAATCCACTGAATATGTTTTGTCCAAATTGTTGCTCTCTCTTGTTATTACCAGCCAACAAGAA
ATCCTGAGATTTCATAATTATGAAATAAAACAAAGTTCCTTAATATCCCATTAATATATACTTTAAAAATCATAG
TCAGGTTACTAAAGAATTATACATCTTTATGTAGATTAATTTGTATAGTTACCTTTTGTCTAGGTTCAAGCTAATT
AGCGTTGTTGTTTACGTCGAAGACATAGATAGCTACAATTGGTGCATCACCATCGTTGTAGCACCAGTGTACAA
TGCCAGCCGGCAGCGCAATAACATCTCCCTGTCTAAAGTGGTGAACTCGTTGGTGCTCATCCTTGATATGGCTT
TGGCTTTGGCTCTGGGATTGATCAAACGGTTGAAACTGTTGTTGGAAGGTCGCCGGACATCCAGGTAAAGTCA
ACCCTGTGAAACCACCCCTGAAAAAGACATAATTGTTTCAACATTAAAAACTTATGAATGCGGTAACATGTGGA
AAATCTAAACTTGGATGTGTAGTATAGCAATGCAATCACCATATTAAGTTATAGGCAAACCTTGAAGGATGTAC
ACTAAGCCAGGTGCGTTGTGGTACTGAGGTAACACGAGTCCTTGGGGCTCAATAACGCGACGGATGACAGAT
ACCCCAGTACAACGAAATTGCTCATTCTGCTCATCAAAGTACTGAGTAACGCCCGCTTGTGACCTCACTTGTCG
AATTGGTTCAAGTGCTTGTAGCCTGTCGAATCTGCATCCCCTTAAACCTCCTTGCCGGGAGCTTTGCCACGGAG
TAAAGCTCTGTCCAAATAGCTGAGCCATGGATCCATGGCAAAAGAGGAAAATGTAAAAGTAAAACAACACTG
GTGGAAGTCTAGTAGTTGCCATGATTGTTTGAAGAGATTGTTGGAGGTGGATTGGTGCTAGTTTCTTAATTGG
AAGCTTGGTTTTATAGCCACAAAAAGTAGTTTAGGATGCACATGGTGACATATTTGCTAATAGAGATAAGAAG
ATATGCCGGTGACTCAGCCGTTCTTTAAAGTGTAAATGGATATAAATTTTACTCAAAAGTATATGTGTTGCTTTC
CGCCTACAACTAGCTATTGCTGAGTCACCTATCTAGTACCATTGATCTTGGCCATTGTGTGAGTTGGTTCAAACA
TGTGATATTACTTGTAGTTTGTACAACTGTTTTGGCACCTTTTATGGAAACTTTTGGTTGTGTGCAAGAAATTCC
ATAAGTCGAGTCTATCTATATATCAGAGTGTAAAGTGGATGAGTCATGTCCATGTCAACAGAGTTAATT

>chr3D:476392526-476394907

AGTCCAAGTGGCCCATATATCTTGTCGATATGCATGAATATGAGGTAGAAACCGACAGAAAATCGATGTACAT
GGAAGGAGATTAGCCCGAAGAGATAAATTTAAGAAAGGTACAAGACACAAACAAGGGATATGACATGATATT
GTTTTTATTCAGGTAAGCACTTATATGACACTAGGTTGCACACTTGCATTGCCTTTATTTCACTATACTAGTTCCC
ATACACTCATTCACTCGGAGGCCTTAACAGCCGAAGAGGACCCCTCCTCGATGTCTTGGTAACTCTGGAAGCCT
GTTTGGGTAAGTTTAGGTGTGAATGCACCAAACTCTTCTTGTCGGTTGTTTTTTAGGTTCCGGGCTTCCTGTCTA
GAAATACGGTATGCATTGGCGAGGACATCCACAGGCAAGGCGCGTAGGATGGAGCTCTTTCCCGCGATGTGA
CTAACCATGGAGTTTGGGTTAGTCTTGAATGAAATGTATTGGCATCCTTCACGCTCTGCCTTCTTGATAACAACA
AAGTGTTGTGGTATGATTAGCAGTTGTCCTCGGCGAAGAATGTCATTGAATACGGTCTGACCATGGTTGTTGA

CGACTTGAACTCGAGCATGTCCTTGGATCATGTAGATGACACTGTGCGCATTAATGTTCCAGAATGGTGCAAG
AATGGCATTCTGCAATAGTAATTTATAAGCTCATATTATTAACCGATTGTTATGAATCTTAGACTATCTGAAAAT
AAGATTCTATAGCATGTAGTATCATATATACCTGGTATAGATTTACTCTTGTAGCACTCATTTGAACGAGGTTAA
GGATGGGGAAATTCTTGCTATTGAGACGTGTTATCCTGCCAGCACGTGGGTTGTATGTGTCGGCACGTTGGGG
GTTTTCAATGTTTTTCATTGCCTCCAATGAACAAAAGTTTTCCTCCAAGCCATTGAAACTTCGGTCCCATGGCTG
TCTTGCCTGGTATTGACTTGATTGCCCTACCTGGTATTGGGTTGATTTCCCTACCTGGTATTAAGTTGATTGTCCT
TCTTGAGTTTGAATTGGTTGGTAGGCTTGCTGCTCTACTGGTACTTGTTGGGACACAAGGGGCTTCAAGAATTG
AAGGCCTTGACTCACACGAATTATCTCACCTATTTGATCATTTTGACTTTGGATCCTTTGTGCTGCTTGTTGACTT
ATACCAAGGGCCTCACTAAGAGGTTGGATACTTAATCCACTGAATATGTTATTTCCAAACTGTTGCTCTCTCTTG
TTGTTACCAGCCAACAAGAACTCCTGAAATTTTATATGGATGGAGTAAAAAGAACTTCATTAATATCACAACAA
TATATACTTAACAAATAAAATTTAGATTACTTGTAAATCGTTATATATCTTTAATATAGACTAATTTGTATAATCA
CCTTTTGTCTAGGTTCAAGCTGATTAGCGTTGTTGTTTACGTCAAAGACATAGACAGCTACAATCGGTGCATCA
CCATCATTATAGCACCAGTGTACAATGCCTGCCGGAAGCGCAACAACATCTCCTTGTTTAAAGTGTTGAACTTTT
TGGTGCTCATCCTTGATATTTTGGCTTTGGCTTTGACCTTGAGCAAACTGGGATTGATCAAATGGTTGGAACTG
TTGTTGGAAGGTCGCAGGACATCCAGGGAAAGCCAACCCTGTGAAACCTCTACCTAAAAAAACATATATGTTT
CAATATTAAAAACTTATGAATGGTAATATTTGGCACAACTAAACACCTAAGTGTAGTATATAACAATGTAGTTG
CTATATTCAGTTAGACACCAACCTTGAAGGATGTAGACCAAGGCAGGAGCGTTGTGGTATTGAGGTAGCAAG
AGGCCTTGGGGGTCGATTACACGGCGGATGACAGATACACCGGTACAACGAAATTGCTCATTTTGCTCATCAA
AGTACTCAGTGATACCTGCTTGTGACCTCACTTGTCGAAGTGGTTCAAACGCTTGTAGCCTATCGAATCTGCAC
CCACGTAAACCTCCTTGACGAGAGCTTTGCCATGGAGTAAAGCTCTGGCCAAACAACTGAGCCATGGATCCAT
GGAACAAGATAAAAATGCAAAAGTAAAACAACAATGATGGAAAACTGGTAGTTGCCATGTTTGTTTGAAGTG
ATTGTAGAAGGTGGATTGGTGCTAGTTCCTAATTTTAAGCTTGGTTTTATAGCCACAAAAAGTAGTTTAAGTTG
CACGTGGCGACGTAATTGTTAACAGAGATAAGAAGATATGCATTGTGACTTGGACATGGTTTAAATTGTGGAC
ATATATAACTTTTCCTCGAATATATGGGCCACTTCCTGCCAACAACTAGTGCTCGGTCACAAAGCTAGCAGCAG
CTTTTGTGCTTAGC

>chr3D:476408321-476410107

TTTTTATTCAGGTAAGCACTTATATGACGCTAGGTTGCACACTTGCATTGCCTTTATTTCACTATACTAGTTCCCA
TACACTCATTCACTTGGAGGCCTTAACAGCCGAAGAGGACCCCTCCTCGATGTCTTGGTAACTCTGGAAGACTG
TTTGGGTAAGTTTAGGTGTGAATGCACCGAACTCTTCTCCTCGGTTGTTTTTTAGGTTCCGGGCTTCCTGTCTAG
AAATGCGGTATGCATTGGCGAGGACATCCACAGGCAAGGCGCGTAGGATGGAGCTCTTTCCCGCGATGTGAC
TAACCATGGAGTTTGGGTTAGTCTTGAATGAAATGTATTGGCATCCTTCACGCTCTGCCTTCTTGATAACAACAA
AATGTTGTGGTATGATTAGAAGTTGTCCTCGGCGAAGAATGTCATTGAATACGGTCTGACCATGGTTGTTGAC
GACTTGAACTCGAGCATGTCCTTGGATCATGTAGATGACACTGTGCTCATTAATGTTCCAGAATGGTGCAAGAA
TGGCATTCTGCAATAGTAATTTTTAAGGTCATATTATTAACCGATTGTTATGAATATTAGACTATCTGAAAATAA
GAATCTATAGAATGTAGTATCATATATACCTGATATAGATTTACTCTTGTAGCACTCATTTGAACAAGGTTAAG
GATGGGGAAATTCTTGCTATTGAGACGTGTTATCCTGCCAGCACGTGGGTTGTATGTGTCGGCACGTTGGGGG
TTTTCAATGTTTTTCGTTGACTCCAATGAACAAAAGTTTTCCTCCAAGCCATTGAAACTTCGGTCCCATGGTTGTC
TTTCCTGGTATTGACTTGATTGCCCTACCTGGTATTGGGTTGATTGCCCTACCTGGTATTGAGTTGATTGTCCTT
CTTGAGTTTGAATTGGTTGGTAGACTTGCTGCTCTACTGGTACTTGTTGGGACACAAGGGGCTTCAAGAATTGA
AGGCCTTGACTCACACGAATTATCTCACCTCTTTGGTCATTTTGACTTTGGATCCTTTGTGCTGCTTGTTGACTTA
TACCAAGGGCCTCACTAAGAAGTTGGACACTTAATCCACTGAATATGTTGTTTCCAAACTGTTGCTCTCTCTTGT
TGTTACCAGCCAACAAGAACTCCTAAAATTTTATATGGATGGAGTAAAAAGAACTTCATTAATATCACAACAAT
ATATACTTAACAAATAAAATTTAGATTACTTGTAAATAGTTATATATCTTTAATGTAGACTAATTTGTATAATCAC
CTTTTGTCTAGGTTCAAGCTGATTAGCGTTGTTGTTCACGTCAAAGACATAGACAGCTACAATCGGTGCATCAC
CATCATTGTAGCACTAGTGTACAATGCCTGCCGGAAGCGCAACAACATCTCCTTGTTTAAAGTGTTGAACTTTTT
GGTGCTCATCCTTGATATTTTGGCTTTGGCTTTGACCTTGAGCAAACTGGGATTGATCAAATGGTTGGAACTGT
TGTTGGAAGGTCGTAGGGCATCCAGGGAAAGCCAGCCCTGTGAAACCTCTACCTAAAAAACATATATGTTTCA

ATATTAAAAACTTATGAATGGGTAATATTTGGCACAACTAAACACCTAAGTGTAGTATATAACAATGTAGTTGC
TATATTCAGTTAGGCACCAACCTTGAAGGATGTAGACCAAGGCAGGAGCGTTGTGGTATTGAGGTAGCAAGA
GGCCTTGGGATTCGATTACACGGCGGATGACAGATACACCGGTACAACGAAATTGCTCATTTTGCTCATCAAA
GTAC

>chr3D:476423981-476425981

TTTTTATTCAGGTAAGCACTTATATGACGCTAGGTTGCACACTTGCATTGCCTTTATTTCACTATACTAGTTCCCA
TACACTCATTCACTTGGAGGCCTTAACAGCCGAAGAGGACCCCTCCTCGATGTCTTGGTAACTCTGGAAGACTG
TTTGGGTAAGTTTAGGTGTGAATGCACCGAACTCTTCTCCTCGGTTGTTTTTTAGGTTCCGGGCTTCCTGTCTAG
AAATGCGGTATGCATTGGCGAGGACATCCACAGGCAAGGCGCGTAGGATGGAGCTCTTTCCCGCGATGTGAC
TAACCATGGAGTTTGGGTTAGTCTTGAATGAAATGTATTGGCATCCTTCACGCTCTGCCTTCTTGATAACAACAA
AATGTTGTGGTATGATTAGAAGTTGTCCTCGGCGAAGAATGTCATTGAATACGGTCTGACCATGGTTGTTGAC
GACTTGAACTCGAGCATGTCCTTGGATCATGTAGATGACACTGTGCGCATTAATGTTCCAGAATGGTGCAAGA
ATGGCATTCTGCAATAGTAATTTTTAAGGTCATATTATTAACCGATTGTTATGAATCTTAGACTATCTGAAAATA
AGAATCTATAGAATGTAGTATGATATATACCTGATATAGATTTACTCTTGTAGCACTCATTTGAACAAGGTTAA
GGATGGGGAAATTCTTGCTATTGAGACGTGTTATCCTGCCAGCACGTGGGTTGTATGTGTCGGCACGTTGGGG
GTTTTCAATGTTTTTCGTTGACTCCAATGAACAAAAGTTTTCCTCCAAGCCATTGAAACTTCGGTCCCATGGTTG
TCTTTTCTGGTATTGACTTGATTGCCCTACCTGGTATTGGGTTGATTGCCCTACCTGGTATTGAGTTGATTGTCCT
TCTTGAGTTTGAATTGGTTGGTAGACTTGCTGCTCTACTGGTACTTGTTGGGACACAAGGGGCTTCAAGAATTG
AAGGCCTTGACTCACACGAATTATCTCACCTCTTTGGTCATTTTGACTTTGGATCCTTTGTGCTGCTTGTTGACTT
ATACCAAGGGCCTCACTAAGAAGTTGGACACTTAATCCACTGAATATGTTGTTTCCAAACTGTTGCTCTCTCTTG
TTGTTACCAGCCAACAAGAACTCCTAAAATTTTATATGGATGGAGTAAAAAGAACTTCATTAATATCACAACAA
TATATACTTAACAAATAAAATTTAGATTACTTGTAAATAGTTATATATCTTTAATGTAGACTAATTTCTATAATCA
CCTTTTGTCTAGGTTCAAGCTGATTAGCGTTGTTGTTTACGTCAAAGACATAGACAGCTACAATCGGTGCATCA
CCATCATTGTAGCACCAGTGTACAATGCCCGCCGGAAGCGCAACAACATCTCCTTGTTTAAAGTGTTGAACTTT
TTGGTGCTCATCCTTGATATTTTGGCTTTGGCTTTGACCTTGAGCAAACTGGGATTGATCAAATGGTTGGAACT
GCTGTTGGAAGGTCGCAGGGCATCCAGGGAAAGCCAGCCCTGTGAAACCTCTACCTAAAAAACATATATGTTT
CAATATTAAAAACTTATGAATGGGTAATATTTGGCACAACTAAACACCTAAGTGTAGTATATAACAATGTAGTT
GCTATATTCAGTTAGGCACCAACCTTGAAGGATGTAGACCAAGGCAGGAGCGTTGTGGTATTGAGGTAGCAA
GAGGCCTTGGGATTCGATTACACGGCGGATGACAGATACACCGGTACAACGAAATTGCTCATTTTGCTCATCA
AAGTACTCAGTGATACCTGCTTGTGACCTCACTTGTCGAAGTGGTTCAAACGCTTGTAGCCTATCGAATCTGCA
CCCACGTAAACCTCCTTGACGAGAGCTTTGCCATGGAGTAAAGCTCTGGCCAAACAACTGAGCCATGGATCCA
TGGAACAAGATAAAAATGCAAAAGTAAAACAACAATGATGGAAAACTGGTAGTTGCCATGTTTGTTTGAAGTG

>chr3D:476439621-476441607

ATGACATGATATTGTTTTTATTCAGGTAAGCACTTATATGACGCTAGGTTGCACACTTGCATTGCCTTTATTTCA
CTATACTAGTTCCCATACACTCATTCACTTGGAGGCCTTAACAGCCGAAGAGGACCCCTCCTCGATGTCTTGGT
AACTCTGGAAGACTGTTTGGGTAAGTTTAGGTGTGAATGCACCGAACTCTTCTCCTCGGTTGTTTTTTAGGTTCC
GGGCTTCCTGTCTAGAAATGCGGTATGCATTGGCGAGGACATCCACAGGCAAGGCGCGTAGGATGGAGCTCT
TTCCCGCGATGTGACTAACCATGGAGTTTGGGTTAGTCTTGAATGAAATGTATTGGCATCCTTCACGCTCTGCC
TTCTTGATAACAACAAAATGTTGTGGTATGATTAGAAGTTGTCCTCGGCGAAGAATGTCATTGAATACGGTCTG
ACCATGGTTGTTGACGACTTGAACTCGAGCATGTCCTTGGATCATGTAGATGACACTGTGCGCATTAATGTTCC
AGAATGGTGCAAGAATGGCATTCTGCAATAGTAATTTTTAAGGTCATATTATTAACCGATTGTTATGAATCTTA
GACTATCTGAAAATAAGAATCTATAGAATGTAGTATGATATATACCTGATATAGATTTACTCTTGTAGCACTCAT
TTGAACAAGGTTAAGGATGGGGAAATTCTTGCTATTGAGACGTGTTATCCTGCCAGCACGTGGGTTGTATGTG
TCGGCACGTTGGGGGTTTTCAATGTTTTTCGTTGACTCCAATGAACAAAAGTTTTCCTCCAAGCCATTGAAACTT
CGGTCCCATGGTTGTCTTTTCTGGTATTGACTTGATTGCCCTACCTGGTATTGGGTTGATTGCCCTACCTGGTAT
TGAGTTGATTGTCCTTCTTGAGTTTGAATTGGTTGGTAGACTTGCTGCTCTACTGGTACTTGTTGGGACACAAG

GGGCTTCAAGAATTGAAGGCCTTGACTCACACGAATTATCTCACCTCTTTGGTCATTTTGACTTTGGATCCTTTG
TGCTGCTTGTTGACTTATACCAAGGGCCTCACTAAGAAGTTGGACACTTAATCCACTGAATATGTTGTTTCCAAA
CTGTTGCTCTCTCTTGTTGTTACCAGCCAACAAGAACTCCTAAAATTTTATATGGATGGAGTAAAAAGAACTTCA
TTAATATCACAACAATATATACTTAACAAATAAAATTTAGATTACTTGTAAATAGTTATATATCTTTAATGTAGAC
TAATTTCTATAATCACCTTTTGTCTAGGTTCAAGCTGATTAGCGTTGTTGTTTACGTCAAAGACATAGACAGCTA
CAATCGGTGCATCACCATCATTGTAGCACCAGTGTACAATGCCCGCCGGAAGCGCAACAACATCTCCTTGTTTA
AAGTGTTGAACTTTTTGGTGCTCATCCTTGATATTTTGGCTTTGGCTTTGACCTTGAGCAAACTGGGATTGATCA
AATGGTTGGAACTGCTGTTGGAAGGTCGCAGGGCATCCAGGAAAAGCCAGCCCTGTGAAACCTCTACCTAAAA
AACATATATGTTTCAATATTAAAAACTTATGAATGGGTAATATTTGGCACAACTAAACACCTAAGTGTAGTATAT
AACAATGTAGTTGCTATATTCAGTTAGGCACCAACCTTGAAGGATGTAGACCAAGGCAGGAGCGTTGTGGTAT
TGAGGTAGCAAGAGGCCTTGGGATTCGATTACACGGCGGATGACAGATACACCGGTACAACGAAATTGCTCA
TTTTGCTCATCAAAGTACTCAGTGATACCTGCTTGTGACCTCACTTGTCGAAGTGGTTCAAACGCTTGTAGCCTA
TCGAATCTGCACCCACGTAAACCTCCTTGACGAGAGCTTTGCCATGGAGTAAAGCTCTGGCCAAACAACTGAG
CCATGGATCCATGGAACAAGATAAAAATGCAAAAGTAAAACAACAATGATGGAAAA

>chr3D:476444421-476446421

CTCAGATGCCTTATTAGCCCAAGATGACTCCTCATTGTTGTCTTGGCAACTCTTAAAACCTGTTTGAGTAAATTT
AGGAGTGAATGCACCGAACTCTTCTCCCCTATTGTTTTTTAGGTTCCGCGCTTCCTACCTAGAAATGTGGTAGGC
ATTGGCAATGACATCACAAGCAAAGCGCGTAGGATGGAGCTCTTTCCTGCGATGTGACTAACCATAGAGTTTG
CATTGGTCTTGAATGAAATGTATTGGCATCCTTCACGCTATGCCTTCTTGAGAACAACATAGTGTTGTGGTATG
ATTAGCAACTGCCCTTGTAGAAGACGATCATTGAATACTGTCCGACCATGGTTATTGATGACCTGAATTCTTGA
TAGTTTTTGATGGGGTTCCGCGGTAGAAAAAAATCCTAGCAACGCAAACACCACAACCAAGATCTATCTATGA
AGATGTAAGGTAATCAGAGGGTTCCGGGATTCATAGCTTTGAAGACCGAAAGCGGTTAACGTATCAAGAGAA
ACGTTGCTGATGTAGTCGAACGACGTAGCCGACCTCAAGGTCATGTAGATGTCCAAACTCCAAGCTCCAAGTG
ATTAACACCACAAGTGGCGGTGCCTCGTGGCTTTACACGCATCCTGTGTAGAGACGTCTCCCAGGCTCCGGTCT
AGCAGAGCAGCGGAAGAAGATTGATCCGGACCGAGTCCAGCAGCACGGCGACGTGTATATGGTGGAGAAAC
CTCTATCCTTGTATACAGTTGCAATCTTGGGAACCAAAAGTGAGGGAGAGAGAGGGGGAGAGATAATAGGTA
AAACCAATTGTTGGGATAGATTTAGGGAGAGGGGTGTCCCTCTTTATAAAGGTGGAAGGGAGGCACCACCTC
CCTCAAACTCCTGGGGTCGGACTAATCAAGGGGGAGTAGGAGGAGGAGGCATCCAGAGGTGGGAATGGGCC
CCACCCTAAGCTCCTTGGCCCACTAAGGCCAACTAGTGGTGGGAGGCCCCCACCCCACGCGCCTTTGATCCTTG
GGGTGGCTAGCCCCCATGGACCCTTCCCTCTGGGAAGGCCCGTGTAGGTGGACTGCCCCATAAATCCATTTAA
ATAATCATTTTAATATTAATTCATTTAATTAATATTAAATAAATATTATTTAATACCTATCACTCTGAAACACATTT
TGGACCCTTCCGATCATCATCGGATATCCCCGAAAACATTCCGATATCACCCTAGAACAATTCCGGAGTTCTCTC
TGTTTTCTCCTACGATCTTGGATCAATACCTAAGTGTCATGAATCCATCAGCAGGACACCCTACAGTTCGTGAAT
ACACAAACATGATCGAGACACTCTCTGATCAATGACCAACAGTGGTGTCTAGAAAGTCATGTTGGCCCCCATGC
ATTCCATGAATAACTTGATCGCTTGAACCTTGAGCGTCGAGTCTCATTCCCTTTGCACTATGATACCTGATTTGT
CCAAGACCTGATCTATGGTGTCAACATACCTAGTTTGATCTTATTACCTACAAACACATATACTTGTTCCCATGA
TATTACATCGTTGTGACCTAGTCACATGTTTGAACTATAGATGTAATCTCACCGAGTGGGCCCAGAGTATCTAT
CTGTCACTCAGATAGGCAAATCCCGATCTCAATACATATGCCTCAACCTATTCATTTGGAATACACAATACACAC
CTTTATGGCCACCTAGTTACATAAATGACAGTTGATGTGATCAAAACATCCGCGGGAGATAGTGATTAATATGA
TCTCACGGTCTAAGGACTGAATCACTACGCTTTGCTAGATATCGCAACATAGGACTTTGTAACTGGATCACATT
TCAATACTACGGTCAGATGTGTCCATCACATTATTCATCCAATGAAGTGACCTCATTGTTAGTTGACGTTAATAA
GTGCATGATCAGGAAATCTCGATCATCAGCTGCCTAACAAACTAGTTACTAGAGGATACTAGGGATTTAGTTTG
TTTACAT

>chr3D:476452449-476454607

ACTTTGAAAAAATTCTTGTCCAAATGGCATACACATGAGGTACAAACCAGCCAAAAATCGAGGGAGAAGGAA
GGAGATTATCCACAAGAGATAAATTTAAGAAAGGTACAAGACACAAACAAGGGATATAACATGATAAAGTTTT

TATTCGGGTAAGCACTTATATGACACTAGGTTGCACACTTGCAATGCCTTTATTTTACTATACTATTTCCCATTAC
ACTCATTCACTCGGATGCCCTAACAGCCGAAGATGACCCCTCCTCGCTGTCTTGGTAACTATGGAAGCCCGTTT
GGGTAAGTTTAGGTGTGAATGCACCAACCTCTTCTCCCCGGTTGTTTTTGAGGTTCCGGGCTTCTTGTCTGGAA
ATGCGGTATGCATTGGCAAGGACATCGACAGGTAAGGCACGTAGGATGGAGCTCTTTCCTGCGATGTGACTA
ACCATGGAGTTTGGATTAGTCTTGAATGAAATGTATTGGCATCCTTCACGCTCTGCCTTTTTGATAACGACGAA
GTGTTGTGGTATGATTAGCAGTTGTCCTCGACGAAGAATGTCATTGAATACGGTCTGACCATGGTTGTTGACAA
CTTGAACTCGAGCATGTCCTTGGATCATGTAGATGACACTGTGCGCATTAATGTTCCAGAATGGTGAAAGAAT
AGCATTCTGCAATCATAATTATTTGAAAGCCATATTTATTAACCAGTTTTATGAATCATAGACTATCTAAAAAAA
AGATAGTGTGTAGCATGTAGTATCATATATACCTGGTATAGATTTACTCTTGTAGCACTCATTTGCACGAGGTT
AAGGATGGGGAAATTCTTGCTATTGAGACGTGTTATCCTTCCAGCACGTGGGTTGTATGTGTCGACATGTTGG
GGGTTTTCAATATTTTTCCTTGCCTCCAATGAACAAAAATTCTCCTCCAAACCATTGAAACTTCGGTCGGATGAC
TGTCCTTCCTGGAATTGACTTGATTGCCCTTCTTGATATGGAGTTGATTGTCCTACCTGGTATTGGGTTGATTGT
CCTTCTTGAGTTTGAATTGGTTGGTAGACTTGCTGCTCTACTGGTACTTGTTGGGACACAATGGGCTGCAAGAA
TTGAAGGCCTTGACTCACACGAATGATCTCACCTCTTTGGTCATTTTGACTTTGGATCCTTTGTGCTGTTTGTTG
ACTTATACCAAGGGCCTCACTAAGAAGGTTGACACTTAATCCACTCAATATGTTGTTTCCAGACTGTTGCTCTCT
CTTGTTGTTACCAGCCAACAAGAACTCCTGAAATTTTGTATGGGTGGAGTAAAAAGAACTTCATTAATATCAAG
ACAATATATACTTACCAAATAAAATTTAGATTACTTGTAAATAGTTATATATCTTTAATGTAGACTAATTTGTATA
ATCACCTTTTGTCTAGGTTCAAGCTGATTAGCATTGTTGTTTACGTCAAAGACATAGACAGCTACAATCGGTGC
ATCACCATCATTGTAGCACCAGTGTACAATGCCTGCTGGAAGCGCAACAACATCTCCTTGTTTAAAGTGTTGAA
CTTTTTGGTGCTCATCCTTGATATTCTGGCTTTGGCTTTGACCTTGAGCAAACTGGGATTGATCAAATGGTTGGA
ACTATTGTTGGAAGGTTGCAGGGCATCCAGGGAAAGCCAACCCTGTGAAACCTCTACCTAAAATAAAACATAT
TTGTTTGAATATTAAAAACTCATGAATGGTTTAATATTTGGCACAAATAAACTCCTAAGTATAGTATAATGATGC
AATTCTATATTCAGTTAGACACTAACCTTGAAGGATGTACACCAAAGCAGGAGCGTTGTGGTATTGAGGTAGC
ACAAGGCCTTGAGGTTCGATTACACGGCGGATGACAGATACACCGGTACAACGAAATTGCTCATTTTGATCAT
CAAAGTATTCAGTGATACCCACTTGTGATCTCACTTGTCGAAGTGGTTCAAATGCTTGTAGCCTATCGAATCTGC
ACCCACGTAAACCTCCTTGACGAGAGCTTTGCCATGGAGTAAAGCTCTGGCCAAACAACTGAGCCATGGATCC
ATGGAACGAGATAAAAATGAAAAAGTGAAACAACACCGATGAAAAACTAGTAGTTGCCATGTTTGTTTGAAGT
GATTGTAGAAGG

>chr3D:477083177-477086418

TTTTTTTTTTGGAAAGGCCATCACCGCCATGGTCATTTTTCTGGGTGTGCCGAGACATCTTCCATGAAGTGGTCC
TCAGGGGCCTGTCAAGAAGCTATGTAGATGGCTTTTTGGCGAACTTGGTCGTACTCACTGCAAAACCATGCGA
ACATTAAAATTTCGCTTCCTCTCCTTTAAAAGAATGGCTAAAAGTTTCTGCATGAGTATATTGGCCGATAATTGA
ATGGCCAAGCCGTGAAATGTGTTAGTAGCCGTTGCTCGTTAAGTCCAAAAACGTTAAATTAGTGTACCAAGATT
TGCCACATCTTGAGAAATAATGGTCCTCACTCACAAGTTCTCGTGTAATTACATCAAAAGTATGACAAAAGGTT
CATGAATGAATGAGTACTTGAGCCACACTCACAAGGTGCAAACGGCTGCAAACCCCTTTTTTTTAACTTGCTTC
AAACCCAGTTTAGGAACCGGCTGCACGCGATTCAGAATAAATGGAACCACTCTGTACTGTTTGGAACAAAGCA
AAACATATATACCGAGAAGTGGCAGATGGCGTTTTCGGAGAGCTCTGTTGTTCAGAATTTATTTTATATATATG
GCAAAGTGATTTGCGCTTCTACGAGTTTTATATATTTTATTGGATGCAACTCCTTCTAATTTTGTCGAAAAACCA
AGTTTGTCCCTTGGCAGCTTGTTGTCTCAAAACAGGTCTTGCGAAAACACGATCGACTAGCTATACATATTGTG
ACAATATAACATATTTTTATAAGTTTAGTCAGCACAACTTCAAAGCGGAAGTTTCATCACTAGCCACCTACGCTT
AAGTTGTAGCAATTGAGAATTAGCAAAACTTAAGTTGTACATATGCTAGAAAAATACTAGAACCACGAAAAAG
AATACTGAAAATATTCTTATACAAGTGGCCTATATATTTGGTTGATATGCATGAGCATGAGGTACAAAATGGCG
GCAAATTGATGTGGATAGAGGGAAAATGATTGTGTGGAAACATAAATTTAACAAAAGTGCATGACATAACAT
GATGAACTTTTTTATTTCGATAAGGGGTTACATGCCACCAAGCGACACACTTTTTATTTCTCATATACTAGCTAT
CATAACACTCATTAATTTACTTAGACGACTCGCCAACATTGTCTTGGTAACTCTGGAAGCTCGTTTGAGTAAATT
TAGGAGTGAATGCACCAAACTCTTCTCCCCTGTTGTTTTTCACGTTTCGGGCTTCCTGCCTTGAAATGCGGTAGG
AATTGGCAATGACATCAACAGGCAGGACACGCAGGATGGAGTTCTTTCATGCAATTTGACTAACCATAGAGTT

TGGGTTGGTCTTGAATGAAATGTATTGGCATCCTTCACGCTCCGCCTTCTTGAGAACAACATAGTGTTGCGGTA
TGATTAGCAACTGCCCTTGGCGAAGACGGTCATTGAACACTGTTTGACCATGGTTATTCACGACCTGAACCCGA
GCATGCCCTTGTATCATGTACATGACACTGTGTGCATTAATGTTCCAAAATGGTGAAAGAACAGCATTCTGCAA
TCACAATTATTTGTATGTCATATTTTTGTTATTGATGCATAGGATGTAAAATCATAGAGAAGTGCGCTAAAATGT
AGCATCATATATACCTGGTACAGATTTACTCTCGTAGCACTCATTTGCACGAGGTTAAGGATGGGGAAATTCTT
GCTGGTGACGCGTGTTATCCTACCGGCACGTGGGTTGTACGTGTCAGCACGTTTGGGGTTCTCGATGTTTAGC
CTTGCCTCCAATGAGCAAAAGTTCTCTTCCAAACCATTCCAACTATCACTCATTGATTGTTCCCCCTGGTACGGA
AACGATTGTCCTTCTTGTTGGTATTGTGTCCATTGACCTTCCTGGTACGGTGTCGATTGTCCTGCCTGGCTTTGA
GTTGGTTGGTAGGCTTGCTCCTCCACTGGTCCTTGCTGGGACACAATGGGCTTCAATAATCGAAGACCTTGTTT
CACACGAATTATTTCGCCTCTTTGATCATTTTGACTCTGGATTCTTTGTGTTGTTTGTCGACTAATACCAAAGGCC
TCACTAAGAAGTTGGACATTAAATCCACTGAATATATTTTGTCCAAATTGTTGCCCTCTCTTGTTGTTACCAGCC
AACAAGAAGTCCTGAAATTTCATATTGATGAGGTAAAACCAAAGTTCAATAACATTTCACACTTGTATACACTTT
GAAAATCACAGGTAGGTTGCTTGTAACCAACCATATCTTGTGTGTGGATTAGTTTGAAACACATGTATAGTTAC
CTTTTGTCTAGGTTCAAGCTGATTAGCGTTGTTGTTTATGTCAAAGACATAGACAGCTACAACTGGAACATCCC
CATCATTGTAGCACCAATGTACGATGCCAGCTGGCAGCGCAACCACATCTCCTTGTTTGAAGCGGTGAATTCTC
TGGTGCTCATCCTGTAACTTTTGGCTCTGGCTTTGACCCTGAGCATATTGTTCTTGACCAAATGGTCGGAACTGT
TTTTGAAAAGTCGCTGGGCATCCAGGGAGGGTCAACCCTGTGAAACCCCTACCTAACATAAAACATGTTTTTTG
AATATTTATAACCCATGTATGATGATGTAAAAATATTCGAAAACTCCTTAGTATAGCAAAGCAATATAATTGTCA
TATCAGTTCGGACACCAACCTTGGAGGATGTACACCAAGCCGGGAGCATTATGGTATTGTGGTAACAAGAGGC
CTTGAGGCTCAACAACACGGCGGATGACCGATACTCCGGTACATCGAAATTGCTCATTCTGCTCATCAAAGTAC
TCGGTGATGCCTGCTTCTGATCTCACTTGCCTATGTGGTTCAAATGCTTGTAGCCTATCAAAAATGCAACCTCTT
AAACCTCCTTGTCGAGAGCTTTGCCACGGAGTAAAGCTCTGTCCGAATAACTGAGCCATGGATCCATGGCACA
AGAGGAACATGGAAAAGTAAAACAAAACGGATCGAAAGCCGGTGGTTGCCATGATTGCTTTAAGAGATTGAA
GAAGGTTGATTGATGCTAGTTTGATGAATGTGAGCTAGGTTTTATAGCCATAAAAAGTAGCATAGGATGATCA
TGACATACATAGTAGATATACATGGTGACTCAGATATGGTTTAAAATGTGAGTCGATATAACTTTGCTTAAAAG
TTATGTTCTGCTTTTTTTTGCTTACCTACAACTAATGCTAACCTACCAAAGCAG

>chr3D:479670106-479672881

GGTCAAACAAGATGGTTCAACATCTTAACATTCAAGCAAGTAATGCTATTCGGACATAAACTTTTTTCTAGTATA
TTATTCTGAATTATCCCAAAGTTACATAGTGCGGGTCACACATGATCATGTGTGAAACTTTATTTGTCACTGTTA
CATCCATCGATTGACTAAGAACTCTTCTCAGAAGCATGCGCCACCAGCACATGATCTAGCGACACCGCCAAATG
GCGTGGCGGTAGTGCTACAACAGTATTGTGGCACATACACGTTGCACATCATCGGTAGGGTCTCCATGACCAT
GGCCCTTGTCGCCTCAAACTGAGCTACTTGCAGAGGCTGGAAGGATCCCTGGCTGAGTTGTTGTTGCATGTGG
ATGGCATGCACAATGCTGCAGATGGCCTGGCACCGTGATTGATGGGGGATCTGCACCAGCTGCTGGCAACACT
GTTGATGCATCACGCGGCAGCTACTCAGTTGCAGCATCTGTGACCGGAGGAATGCACGGTTGGAATGAGAGG
ACAACATCACCGCATCCACTCGAGGGTACTATCCTCCACCACCATAGACGGTTGCCAACCGGACGTGGTAGTA
GGGGCATACCATGCCAAAATAGGCCTGGTCGGATCCAGACCAAGTTCTCGCCGCCATGCTACATCAAATAGGC
CGCCGCTGCACCTCGCGCCGTCCAGGCAAAGCCCCACCACACGCCTTCTCTGGGCCTAGACATGCATCTGGGA
ATAGTGAAGTTAGACATAATACACATTGGAATCGTTTTCTCTTGTCATTGTGAAGTTACATTACTACGCATCTGG
AAATAGAGTGACATCTTTTTGAAGTAACTAGTGGTTTCTACAAAGTACATAACAAGACCATGGAGCCTAGTTTT
CAAAGAAAGTAATCTTGTTATTGCAAGGAATTACTCATCACACACTGTGTGAAATAAAAACATGGGAAAATGA
CAGAAGCAACCAATTCCTCCCTCTCTGAGTGTCACAAGTCTATCCATTCTTAAGAGGAGTCAATTTGCGGTTATT
CCCTACTTTAGTGAGCCCAAAATTAAGAATTGGTATGTTATAAGATTATTGTGCAACATAAATAGTCAATTAAT
GCAAGGCTTTTTATTTTGGAGGGGGTGAGTCAAGGTCTCAAGGCAGGTTGTGGTCAAACAAGATGGTTCAACA
TCTAAACATTCAAGCAAGTAATGATATTCGGACATAAACTTTTTTCTATATTATTCTGAATTATTCCCAAGTTCCA
TAGTGTGGGTCACACATGATCATGTGTGACACTTTATTTGTCACTGTTACGTCCATCGATTGACTAAGAACTCTT
CTCAGAAGCATGCCCCACCAGCACATGTTGTAGCGAGGCCGCCGAATGGCGCGGCGGTAGTGCTACAACAGT
ACGGTGGCACATACACGTTGCACATCGTCGGCAGGGTCTCCATGACCATGGCCCTTGTCGCCTCAAACTGAGC

TATTTGTAGAGGCTAGAAGGATCCCTGGTTGAGCTGTTGCTGCATGTGGATGGCGTGCACAATGCTGCAGATG
GCCTGACACCATGATTGCTCCAGGATCTGCACCAGCTGCCGACAACACAGTTGATGCATCACATGGAATCTACT
CTGTTGCAACATCTGTGAACGGAGGAACGGCGCCGCCATCGTCTGCATCATCAGGTCACACTGCTTCGGCATG
AACGCCTTGCATGGGTTCATGTGTTGCGGTAGGAGAGGTTGGGAAAATTTTTGTTGTTGGATACATGGTTGTG
AAGGGTTCGCAGCATAGAAAACAAAAAAATTCCTACGATGTGAACTACTATATGCCAGGATCTATCTACTAGAT
ACAAGCAACGGGTAGATATACATCCATAGAGAATATACCCTCGAAGACAACAAAGCTTTGTTAATGAGATGGA
ACTCATGGATGATGTAGTCGTACACTTGCCGATCTCAAGATCGTGATGAAGTCCAGCAGGTCCAGCTCCAGCA
CGTCCATCAGATCTAGCTCCAGCAAGTCCAGCACCACCAAGTCCAGCACCGCAAAGCAGCGGTGCATCCATGA
TCGCTCTAGCAAAGTAGCGGAAGCAGCAACTCCTTTCCAGATAAAGCCATCGGGAACATGTATCTTGCTAATCC
GAGACCATGAGAAAAATACTAGAGGGGGAGAGAGATCCTTCACAAGGAGGTGTGAGGAAGAAGGGGTGTG
AGGGTGGCCTTTTATAGGAGGAGGAGGGTGCCCCTCTCCATGGCCGGCGCCCAAGGGGTGACCCTTCACCTG
CTCATTCATGTGGTGGTAACTCCCTCCACATAAATTTGCCCCTTAACATTCCTTTTTAAAGTATCCACTTTAAATG
TATTAAATTTCTCCAAATTTAATGCTCTCTCATTTAATAAAACTCTTTTATTAATTTTATTCAGAATTTTGTTAATT
ACTTTAATTAACAAACATTCACATAAAATCCCCTGCAACATTTCAATATTACATGCAGAAAATTTCAGCAATTTC
ACCCGAAACAGTTTCGGTGTCTATTGAAATAATTCCAGCGCTTTCTTAAATCTTTATCTGAAACATATTTGCTCC
AAATGGTGTCGGTATCCCTTAAGTGTGTCATCCTACGGTTCGCGAATAATGCAGACATGATCGAGACCCCTCTC
AGATCAATAATCAGAAGCGGGACCCAGAGATCCATACTGACTCCTACACATTCAACGATGACTTCTGTGATCGT
GAGAACCTTTAATGCTAGTAATTAAATCCCTTTGTCTCGCGATATATT

>chr3D:479705442-479706819

GACAATAAAAATCTATTTATTATTTGCCTCTAGGGCATATCTCCTTCACTACACTCCCTCACTCTCACTCTCCAAA
TTCTCTAGATCTAGCCAATAAATTTGCCTTAATTATGCATCCTCTGTCCACACCACTCTCCTCCACCCCCTAGCAA
ATTAGCTCACTCTCCCATGCATTGAATTAATCATTCTCCTTGTTTATCTTGGAGGTGTTGAGTCAAGACATGTTG
TGATCAAATAAGATGGTTCAACATCTGAATATTCAAGCAAGTAATGCTATTCGGACATAAACTTTTTTTCTAGTA
TATTATTCTGAATTATTCCCAGGTTCTATAGTGCGGGTCACACATGATTATGTGTGACACTTTATTTGTCACTGTT
ACATCCATAGATTGACTAAGAACTCTTCTCAGAAGCATGCGCCACCAGCACATGATCTAGCGACGCCGCCGAAT
GGCGTGGCGGTAGTGCTACAACAGTACGGTGGCACATACACGCTGCACATCGTCGGCAGGGTCTCCATGACC
ATGGCCCTTGTCGCCTCAAACTGAGTTACTTGTAGAGGCTGGAAGGATCCCTGGCTGAGCTGTTGCTGCATGT
GGATGGCGTGCACAATGCTGCAGATGGCCTGGCACCGTGATTGCTCGGGGATCTGCACCAGCTGCCGGCAAC
ACTGTTGATGCATCACACGGCAGGTACTCTGTTGCAGCATCTGTGACCGGAGGAATGGCACTGCCATCGTCTG
CGTCATCGGGTCGCACTGCTGTTGCATGAACGCCTTGCATGGGTTCATCTGTTGCAGTACGAGTGGTTGGGAA
AATGGTTGTTGTTGGGTACATGGCTGTTGTTGACATCCAAGGCCAGGGATGCAGGTAGTGGTGGCGTTGGTG
CCAATCGCAGCGACGACGAGGAGAGCGAGGATGAGGAAGGTCTTCATGGTGGTTGGTGTTGGCTACTACTTG
ATTGACTTTGATGTTTGAGTTCTACTTGTGTGAAGGATGGGGATTGAATGGAGGAGCATACTCTTGGTGCATA
CCTATTTATAATAAAATTGTTTTGGCTTACCTCACTTCCTTGTATTCAACCCAAACAGATTATTTGGTATTTTGTG
TTAGGCATAATCTTGGACTGGTCGCCAAAGTTGCTTTGGTTTTGTACATTTCATAAAAAAAAATTGTCATTGAAA
CTTGAGATAATTGACATGACTCATCTTATTCACTTTACAGGCCAACACAATGATCAAGATTGCATCTTTTTCTATC
AACTGATTTTGTAATATTGTATGAGTTGTTAGGCATAAACTAGATTGTGCATGGTTTGTGGGGAACAAAGTTTG
TAACCTTGTGGGTTGTTAAGAAGGATCAGACTAGCCTAATTAGG

>chr3D:479725609-479726609

GAGTACTAGATGAGTCCAACAAACACCACAATTTCATAACGACATCCAACTCCAAATATAACTCATAACAAGTA
TTTGTAATCTCTAGCCTAAGTGCCACCATGGTGTGTACGCTGTCACTTTCACACACATGGGGAGAGCAGAGATC
TATTAAAGCACTTGAGTAGCAAGGTAGATCTATAGATTACAAGCTCATGATCACAATATGATCATGCATAGAGA
GAGATGAACACATAGCTACCGGTAGAACCCTTAGCCTCGAGAGAGAACTACTCCCTCCTCATCATGGGAATCA
GTAATGGTTATGGAGATGGTGGTGGAGATCGATGGAGATGGCTCCGGGGGCACTTCCCTGTCCCGGCAGGGT
GCCGGAACAGAGACTTCCTGTCCCCCGAATCTCCCCTTCAGTGGCGGCGGCGCTGCAGAACTTTTCGTGGATTT
CTGATTGATGATCTAGGGTTTCTGCCTTGGGAGGCTCTTATAGGCGAAGGAGGCATGGTCAGTGGCCAGCCAG

GGGGCCCACACCATGCCCTAGCATGGCCTGGGCTGGCCCGCGCCAAGGGGGGTGTGTGGCCACCCTGTGGCC
CCTCTCTGACTCTCCTTCTGGCTTCGTGAGTCTTTCGGGAAAATAGTAACTTTGGCTTTTGTCCCGTCCAATTCCG
AGAATATTCATTTTCTAACTTCCTGGAACCAAAAACAGCAGAAAACAGGCAACTGGCACTGTGGCATCTTGTTA
ATAGGTTAGTCCAAGAAATCATATGAAAAGTACCCCAAAGTGTGGATAAAACATAAGAAAGTAGATAAAACCA
AGCATGGAACATCAAAAATTATAGATACATTTGCAATGTATCAAGGAATACCTTAGCTATGCCCCCATTGTAGG
TGACAATAGTTACGATAACCCCTTGTAGACTCTGTAAGCAGCTTTGCCCTGGCAGCCACCATCAGATATGGTGA
ACCTCCACCAATGACATATATGCATAGATGATCTAGACACCAA

>chr3D:479757451-479759319

GATTATTGTCTCTAGTACAAGTGGGAGACTGAAGGAAATATGCCCTGAGAGGCAATAATAAAGTTGTTATTAT
GTATTCCTTATGTATGATAGATGTTTATTTTGCATGCTAGAATTGTATTGATGGAAACATCAATACATGTGTGTT
TAATAAACAAAAAGGGTTTCTAGTTATGGCTCTCTTATAGACTAGCTTGTTGATTAACAATATGATCAAGGTTTC
CTGATCATGGACATTGGATGTTGTTAATAACAAGGTCACAACATTTGTGAAATGATGTGATGAACATTCATCCA
AAGTATAGCATTAAGATCAAGTCATTATGTTTACATTGCTATAACATCATACGAAGTAAACTTTATCCTTAGACC
ATGATATCATGTTAATTGCTAACACCGGAAGAGTATCTTGATAACATCAAACGTCCCACCGTAACAGGGTGACT
ATAAAGATGGGTTCAGTGCTACGAAGGTGTTATGTTGAGGCTCATGGATCAAGAGTGGAATTTGTCAATCCCG
ATGACGGATAGATATACTCTGGGCTCTCTCGATGATATCACATTAAGATAGCTTGCAAGCAAGTGATGTGATCA
CTAGAATGTTATATCACGGGAACAAGAAAAGAGTGCATGTCGGTAACGATATCAAACTAGGTATTTTGGATAC
CGATGATCGAATCTCGGACATGTATTATATCAAGAGACAAAGGGATTAAATTACAAGCGTTAAAGGTTCTCAC
GATCATAGAAGTCATCGTTGAATATGCAGGAGTTAGTATGGACCTCCAGGTCCCGCTTCTAATTATTGATCTGA
GAGGAGTCTCAATCATGTCTGCATTATTCGCGAACCGTAGGGTGACACACTTAAGAATTACCGGACACCATTTT
GGAGCACATATGTTTCAGACGGAAGAATTGCAAAGCGCATGAATTGTTTTGATAGAAACCGGAAATTGTTTCG
GGTGAAATTGCAGAAAGTGGTTCTGCATGTGATATTGAAATATTGCAAGGGGTTATATGTGAATGTTTGTTAA
TTAAAGTAATTAACAAAATTCTGAATAAATTTAATAAAAGAGTTTTATTAAATGAGAGAGCATTAAACTTAGAG
AATTTAATGCATTTAAAGTGGGGACTTTAAAAGGGAATGTGAAGAGGCATTTTAATGTGGAGGGGTTACCACC
ACATGAATGGGGGAGGTGAGGAGTCACCCTCCCATGCGCCGGCTATGGAGAGAGGGGCACCCCCCTCCTCCT
ATAAAAGGCCCCCCTCTTCCTCCTTCTTCCTCACACCTCCTCGTGAAAAATCTCTCTCCCCCTCTAGAATTATTCCC
CCATGGTCTCGGATTAGCGAGATACATGTTCCCGGTGGCATTTCGTCTGGAAGGAGTTGCTGCTTCCGCTGGA
TCGCTGGAGCGAGGAGGTGGAGGTTGTCCGGTGGCTTGAACGTGTGTGCGAGTACGGATGCGCTGCTGAGTT
GCGGCGCTCGGACTTCCACATGATCTTGAGATCGGCAAGTGTACGACTACATCAACCACGAGTTCCTACTCGTT
AACAAAACTCGTTGCCTTCGAGGGTAAGTTCGATGGATGTATGTCCACCCGTTGCTCTGTACCTAGTAGATAGA
TCTCGGCGTGTAGTTCTCATCGTAGGAATTTTTTTGTTTTCTATGCTGTGGACCCAACACCGTGTGTTGGAGATA
TGCCCAATAGGCAATAATAAAAGAGGTTATTATTATATCTTTGTGTTTATGATGAATGTTTACATCCCATGCTAT
AATTGTATTATTAGGAAACACTAATACTCGTGTGTTTTGTAAACATAAAAGAGTCCCTAGTAAGCCTCTTGTTAA
ACTAGCTTGTTGATTGATTGA

>chr3D:479774263-479775319

CTTCTTTTATTTTCTTATTTGTAAGGCCATGTAATACTTGTATTACAATAATTGTTCATTTTATCTTGTAATAAAGA
ATTCCTTTATTTATATAATTGCAATTCCCTATTTCATTTATTGGTGTTATTTTATTCTTTAATTAAATATTGGAATA
TTCTAAATTCCAGGGTATTCAAATTTGAAATTCAAATCTCAAATTCAAATATAAATTGAATTGTTTCCCCAAAAC
ACGAAAGTAGAAATCCATAGCACTTGTGTTGATGTTTCTATCAGAACAAGAGGGATCTCGGTCCCTCCATAGCT
CTTTTAGTTTTGCGTGGAAATTCTCTCGAATTTTCTTATGCATGAATGCAATGCACACACATGTGTCCTGTCTATT
TCTAGGTGCTATTCCTGAGATGTTAGCCGAAGCCCATCTGCTAGCCAATCTCTAACTGTGCAGCAGGTTGGGTA
CCGCAATGGCAAAGAGGGCGAGGATGAGCAAGACCTTCATGGCGGTGACCGTGTGTGGCTAGCTACTCCCTA
CTCGACTTTTACGTTTTGGTGTAGGAAGATGGAAGAGGATGCCCACAATGAACACCTATTTATAGCTGCCATG
GCACCATCTCTTCTGGTTTTCGCATTTTCTTTTCTCCATAATGATTTGGATACTTCTAAAACCAATCATTTGATACC
TTTGTGTTAGGTGTGGCACTACATACTACAAGTATAAACCTTGCATCACTCACCAAATATGCTTTCTATTGCATA
TACATAAACCCTGGGCCACTCACCAAATGTGCTAGGTGCGGCGCCTCACCCGAGCACAATCTTGTATTGTTAGT

AAAGGTTAATCTTCATGTTTCTTGTTGTTGTACGTACATAGAATGGAAGCTTGTGATTATAATCGATAATCGAA
ATGACTCATCCTCGTCCACTTTACATGTCGACGCTTATCAAGATCATGTCTTTTCCCCAAAACTGATTTTGTACTT
TGCACGAGTTGTTCGCCACAAACAACTCATACAAACAACTTGTAAAAGCCAGGTAAAGAAAAACAACTTGTAA
AAGTAATGC

>chr3D:479779022-479781019

CAATGTTATCCAGACACAAACTTTGTCTTTTTTTATTTCCAAGATTGGACTAGTACAGGTCTGGGGTCACACATG
TTAGTGCATAACCCTTTATTTTATTTGTTACCGCTACAACGGCCGGTCCATCGGCTAAACACCGATGATCTGTTT
ATGTAGTATACTATAGCCCTTGTAAGTCTTCTCAGACCACACCAAGCGGGGTGGTGGCTACGGAGCAGTGCGG
TGGGACATACACATCACACATCTCCGGCAGGGCCTGCAGTGCAAACGCCCTCATCCCCTCGAACTGAGCTTGCT
GCTGAGGCTGGAAAACCTGTTGCAGCTGAGGCGGATGAAAACCTGTTGCACCTGAGGCTGGATCAACTGTT
GTTGCACCTGAGGCTGGAGCAGCTGTTGTTGCAGCTGAGCCTGGAGCAGCTGTTGTTGGAGCTGAGCCTGGA
GCAGCTGTTGTTGGAGCTGAGCCTGGAGCATCTGTTGTTGCAGCTGAGTCTGGAGAATCTGTTGTTGCAGCTG
AGGCTGGAGCAGCTGTTGTTGCAGCTGAGGCTGGAGCAGCTGTTGTTGCAGCTGAGGCTGGATCAACTGTTG
TTGCAGCTTCTGCAAGATGATGGCTTGCACGACACTATGGATGGCTGGGCACCGGACCTGCTCGGGGATCTGT
GCCAGCTGCCGGCAGCACTGTTGCTTCATCACCTGGCAGCTGCTCTGTTGCAGGATCTGCGACCGGAGGAACG
GCACCTCTGCCACTGGGCTGCACTGTTGCACGAGGAATTGCCTGCATGGGTTCAGCTGTTGCTGCAGAACCTG
CAACAATTGCTGTTGCTGCAACAATTGTTGTTGTTGCTGCAACAATGGCTGTTGTTGTAGAAATGGTTGTTGTT
GCTCAGGATATGGCTGATATTGTTCGCTAGGGTCAAACTGGGCAGTGGCCATGGTCGCCGCCATGGCAAGGA
GGGCAAGGATGAGGAAGTTCTTCATGGTGGATTTGTGTCCACTACTGTTTGCTTGGCTCTGATGTGTGTGTTGT
AGGTGTGTGAAGGATGGATGATGGAGGAGGGTCTTCACAGTGCATGTCTATTTATACTGCTATGGTGTGGTTT
CTTCTCATCTTTGCATTTGCTTTCTTCAAGAGTGTTTGGGTGCTTACCGAACTGATCATTTGGTGTCTTGTGTTTG
GTGGCACTACTTACAAAATATAATTTTTGGATTGATCATAAAAGTTGCTTTGCCCTTGCATTTATCATCTTCTGAC
TCTCAAAAAGCTTGATAGTTGGTAATATGTCTCATCTTATTCACTTTACATGTCAAACACTAATCTATATTGCATC
ATTTCTAAAACTAATTTTGTATTTTTCTACAGACTGTTAGGCATAAACCAGGTTGTCATGGCTTGCAGAGTTTGT
TAACTTGTGTGTGTATAAGATTAAACTAGTAACCTGAAAGATTTATCTAGACTCCATTACTTTGGCTATGTAGTG
TTGTATAATACAATGTTAAGTTGAACTAGTAAACATGACTCATCAAATTCGCCTTTTATGTCAAAAATCATAATG
GCGTGAAAGTTATTAAACATATCAGATTAGTTAAGCTTTAGGTTCATTACATGTGAAGATTATATTTGGATTTCA
TTCATTTGTTGAACTAATTAAGTTTGTATGTATTGTATGCAAATTGTTCAGATAATATATAGGTTTTATTTAGCAG
ATTTAATCAAAATGTTCAAGACAATTATTGGTAGTGTGCCTAGGATTATTGCAAATGATAGCATTTATACAACA
TATAATGGCATATTTGTTTCCTCAAACTGCCAAATGATCTATTTAATAACCATCCATTGCATAACAAACAAATGG
TGTTTCTTTTATTGATGCTTTTATCTATCTACCTGGTTGCAAGCCCCATCAAATCTGCTTCTGCAAAACAAACCAA
GAACAGGTGGCGCAACTAATCAGCCAGTCACATTTTGTGATTGGTAATTTTTCATCTGAGAACGGACATT

>chr3D:479782203-479784519

CTTTTACACCCACTCTGTTATTATGTTCACATCAATGAAGGCTACATAAGCAGCTAGTTAATGCATGAGCTTATG
GTTAGGTATAAACATGAATGGAAAAACGACAATCAATGTTATGCAGACAAGAACTTTGCATTCTTTTATTTCCA
AGTTTGGACTAGTACGGGTCGGCTGTCACACATGTTAGTGCATGACCCTTTATTTTATTTGTCACCGCTACAAG
ATCGGTCCATCGGCTAAACGCTGATGGTATTTATGTAGTAGAATAGCTCTTGTAGTGTTCTTAGAAGCCACTGA
GCGGGGTGGTGGCGACGGGGCAGTGCGGCGGGACGTATACATCGCACATCGCCGGCAGGGCCTGCAGCGCA
AACGCCCTCATCCCCTCGATCTGACCTTGCATCTGAGTCTGGAAGATGCCCTGTGTCACCTGTTGCATCTGAGG
CTGGAAGATGCCCTGTGTCACCTGTTGCATCTGAGGCTGGAAGATGCCCTGTGTCACCTGTTGCATCTGAGGCT
GGAAGAACTGTTGTTGCATCTGAGGCTGGAAGAACTGTTGTTGTTGTTGCTGCATAATGATGGCCTGGATGAC
GCTATGGATGGCTGGGCACCGGAGCTGCTCGGGGATCTGCTCCAGCCGCCGGCAGCATTGTTGCCTCATCACC
TGGCAGCTGCTCTGTTGCAGGATCTGCGACCGGAGGAATGGCACTGCTGCCACCGGGCTGCATTGTTGCACGA
GGAACTGCCTGCATGGGTTCAACTGTTGCTGCAGAACCTGCAACAATGGTTGTTGCTGCAACAGCATCTGTTGT
TGTTGCTGCAACAGCAACTGTTGTTGTTGTTGCAGAATTGGCTGTTGTTGCTCAGGATATGGCTGGTATTGTTC
GCTAGGGTCAAACTGGGCAGTGGCCATGGTCGCCGCCATGGAAAGGAGGGCAAAGATGAGAAAGGTCTTCA

TGGTGGATTTGTGTTGATACTGCTTGCTAGGTTTTAATGTTTGTGCTCTCGGTGTGTGAAGGATGGAATGATGG
AGGAGCATCTTCATGGTGCAGGTCTATTTATATCTGCCATGGCATAGTTTCTTTTCATTTTTGCATTTGCTTTTCC
TTAAAAAGTGCTTGTATGCTTCTCGAGCTGATTATTTGGTATGTTGTGTTTGGTGGCACTACCTACAAATGTTAT
TGTTGGATTGATCATAAAAGTTGCTTTTCCATTACATGTATCATCTTTCGACTATCAAGAAAGCTTGATAGTTGG
CAATATGTCTCATCTTTTTCGCTTTACATGTCAATCACTGATCTATATTGCATCATTTTCTGAAACTAAGTTTGTG
TTTTTCTACAGGCTGTTAGGCATAAACCATATTGTCATGGCTTGCAAGGTTTGTAAACTTGTCTGTGTATAAGGT
AGATTAAACTAGTAACAGGAAGGATTGTTCTAGACTCCATTTCTTTTTCTGAACAAACATTGTGGTTTTGTATAA
TATGATGGTAGGATGAACTAGCAAGCATCACTCATCAGATTCGCTTTCGGTTACACTATACCAATTATCGATTCT
AATCTTGATTGTTAAATGCTAATAAGTTGAGTCACTTGTATTTGACTGGTGTTTTGCAGTCACTACTCAACATCG
GATGAACTTAATAGGCTTATGCTTAACAACTCTGATGTGATTCCAGTTCTGTTGATCAGTACTGAGCGGCTGAA
TCTGATGAGCTTATATATGATTTGTTCTTACTAGTTTATTTGTAATGTTCTGTTCCCTGAGCTTAAATAGCTTATT
TTGTTTTACAAGGAGGCCTATTCAACGCCTGTCCTTCCATTTGATATCTTAGCTGAGAGACCTCCATAGCAGAA
GTTCGATTTGGCTAGTAATCGTAACATCGCATTCAGTTGAAGACTAAAATCTTTCATAATTCTAATGTTTGAATT
GCTATACCTTTGGTGCTAACAATTCACTTATGGATATCCACCTTCGGCACTTGGACCTTTTCAGGATAAGTTAGT
TTCGTTATACTCTGTTCCTGGACTGACTTATTGATTGCATCAACTTAGGAAGGCAGAAACATCATTTTATCTCAC
ACTAACAAACCTGATCGCGAGCCCATCGAAATATGGCGCTGCCAAAGGAAGCAAGAAGAGACGTTGAAACTG
TTCATTCACCGCCTTTTTTTAGTAGCTGATTTTCATCCCAGGAAGTAAACAGATGCTCTCATTGAAGCTTTAGTT
GTTACCATTGTACGGTATTGGTGTAAGTAGTGGAAGATCATACAGACAATGTAATTTGGGCTATTAGTACTTGA
GCATCCCTATGTCAGC

>chr3D:479786509-479788509

AACATGAATGTAAAACGACAATCAATGTTATGCGGACAAGAACTTTGTCTTCTTTTTATTTCCAAGTTTGGACTA
GGACAAGTCAGGTGTCACACATGTTAGTGCATGACCCTTTATTTTATTTGTCACCACTACAACGATTGGTCCATC
GGCTGAACTCCGATGGTCCATTTATGTACTATAGCCCTTATAGTCTTCTCAGAAGCCACCGAGCGGGATGGTAG
CGACGGGGCAGTGCGGTGGGACGTACACATCGCACATCGCTGGCAGGGCCTGCAGCGCAAACGCCCTCATCC
CCTCGAACTGAGCTTGCTGCTGAGGCTGGTTGAAAACCTGTTGCAGCTGAGGCTGGAAGACCTGTTGTTGCAG
CTGAGGCTGGAAGACCTCCTGTTGCAGCTGTTGCTGGAAGACCTGTTGTTGCAGCTGAGGCTGGAAGAACTGT
TGTTGTTGCTGCTGCTGCAAGATGATGGCCTGCACTACGCTGTGGATGGCCGGGCACCTAAGCTGCTCAGGGA
TCTGAGCCAGCTGCCGGCAGCATTGTTGCCTTGCCACCTGGCAGATGGCCTGTCGCAGGATCTGCGATCGTAG
GAATGGCACCACTGCCACTGGGCTGCACTGTTGCACAAGGAACTGCTTGCACGGGTTCAACTGTTGCTGCAGA
AGTGGCTGGAGAAACATTTGTTGTTGTTGCACAAATGGTTGTTGTTGTACAAATGGCTGTTGTTGTTGTTGTTG
TTGTTGTTGTTGTACAAAAGGCTCTTGTTGCTCAGGATATGGATGATAATGTTCGGTCGAGGCCGTGGCCGTG
GCCGCCGCCATGGCAAGGAGAGCAATGATGAGGAAGGTCTTCATGGTGGATTTGTGGTGACTACTGTTTGCTC
GGCTTTGATGTTTGTGCTCTAGGTGTGTGGATGATGGATGATGGAGGAGGATCTTCATGGTGCAGGTCTATTT
ATATCTGCCATGGCATAGTTTCTTTTCATTTTTGCATTTGCTTTTCCTTAAAGAGTACTTGGATGCTTATCAAACT
GATTATTTGGTATATTGTGTTTGGGAATAATACATACAAATGTAATTTTTGGATTGATCATAAAAGGTGCTTTGC
CCTGCACGTATCATATTTCTACTCTGAAGAAGGCTTGGTAGTTGGCAATGTGTATCATCTTATTCACTTTACATG
TCAAACACCGATCTATATTGCATCGTTTTCTGAAACTAAGTTTGTATTTTTCTACAGGATGTTAGGCATAAACCA
TATTGTCGTGGCTCACAAAGTTTATAAACTTGTGTGTGTATAAGGTAGATTAAACTAGTAACAGGAAGGATTGT
TCTAGACCCCATTTCTTTTTCTGAACAAACATTGTGGTTTTGTATAATACGATGGTATGATGAACTAGTAAGCAT
GACTCATCAGATTTGCCTTTCGGTTACACTACCAATTATCAATTCTAATCTTGACTGTTAAATGCTAATAAAACAT
GCTTTTGAGTCCCTTGTATCTGACTAGTGATTTGCAGTCACTACTCATCGAATGAACTTAATAGGGTTATTATGC
TTAACGACTTTGATGTGATTCACAGTTATGTTGATCAGTACTGAGCGGCTGAACCTGGTGAGCTTATATATGAT
TTGTTCTTACTATGCTAGATATCAATAGCCGCTGTATTATTTTGTAATGTTGTTCCCTGAGCTTAAATAGCTTGTT
TTGTTTTACAAGGAGGCCTATTCAACCGTTGTCCTTCCATTTGATATCTTAGCTGAGAGAGCTCCACAACAGAA
GTTCGAATTGGTTAGTAATCGTAACATTGCATTCAGCAGAAGTTTGAATTGATTCTAAGTTTTGAATTGCTATAT
CTTTGGTGCTAGCAATTCACTTCTGGATATCCACCTTCGGCACTTGGACCTTTTCAGACTAAGGTAATTTCGTTA
TACACTGTTCTTGGACTGACTTATTGATTGCATCGATGTAGGAAGGCAGAAACATCATTTATCTCACACTA

>chr3D:479789802-479791819

ATGGTTGTGTATAAACATGAATATAAAACGACAATCAATGTTATGCAGACAAGAACTTTGTCTTCTTTTTATTTC
CAAGTTTGGACTAGGACAAGTCGGGTGTCACACATGTTACTGCATGACCCTTTATTTTATTTGTCACCGCTACAA
CGATTGGTCCATCGGCCGAACTCCGATGGTCCATTTATGTACTATAGCCCTTATAGTCTTCTCAGAAGCCACCG
AGCGGGACAGTAGCGACGGGGCAGTGCGGTGGGACGTACACATCGCACATCGCTGGCAGGGCCTGCAGCGC
AAACGCCCTCATCCCCTCGAACTGAGCTTGCTGCTGAGGCTGGTTGAAAACCTGTTGCAGCTGAGGCTGGAAG
ACCTGTTGTTGCAACTGAGGCTGGAAGACCTCCTGTTGCAGCTGAGGCTGGAAGACCTGTTGCTGGAAGACCT
GTTGTTGCAGCTGAGGCTGGAAGAACTGTTGTTGTTGCTGCTGCTGCAAGATGATGGCTTGCACTACGCTGTG
GATGGCCGGGCACCTAAGCTGCTCAGGGATCTGAGCCAGCTGCCGGCAGCATTGTTGCCTTGCCACCTGGCAG
ATGGCCTGTCGCAGGATCTGCGATCGGAGGAACGGCACCACTGCCACTGGGCTGCACTGTTGCACAAGGAAC
TGCTTGCACTGGTTCAACTGTTGCTGCAGAAGTGGCTGGAGAAACATTTGTTGTTGTTGCACAAATGGCTGTTG
TTGTTGTACAAATGGCTGTTGTTGTTGTACAAATGGTTGTTGTTGTACAAATGGCTCTTGTTGCTCAGGATATG
GCTGATATTGTTCGCTAGGGTCATACTGGACAGTGGTCGTGGCCGTGGCCGTGGCCGCTGCCATGGCAAGGA
GGGCAATGATGAGGAAGGTCTTCATGGTGGATTTGTGGTGACTGTTTCTTGCTCGGCTTTGATGTTTGTGCTCT
AGGTGTGTGGATGATGGATGATGGATGAGTGGATCTTCATGGTGCAGGTCTATTTATATTTGCCATGGCATAG
TTTCTTTTCATTTTTGCATTTGATTTCTTAAAAAGTGCTTGTATGCTTCTCGAACTGATTATTTGGTATGTTGTGT
TTGGTGGCACTACCTACAAATGTAATTTTTGGATTGATCATAAAAGTTTGCTTTGCCTTGCACGTATCATCTTTC
AACTCTCAAGAATTGCTTAATAGTTCGCAATATGTATCATCTTATTTGCTTTACATGTCAAACACTGATCTATATT
GCATCTTTTTCTGAAACTAAGTTTGTATTTTTCTACTGGCTGTTAGGCATAAACCAGATTGTCATGGCTCACAAA
ATTTGTAAACTTGCGTGTGTATAAGGTAGATTGAACTAGTAACATAAAGGATTGTTCTAGACTCCATTTCTTTGT
CCGAACATCAGTGTAGTTTTGTACAGTACGATGGTAAGATGAACTAGCAAACATGACTAATCAGATTCTCCTTT
TATGTCAAGGATAGTAATGAAGTACAAGTTATCAATGCATATCATATTGGTAGGTCTTTACATGACTGTTGGTT
CAGTACATGTGAAGGTTAGTTTTGCATTTTATTGTTTTGTTGAACTAAATAAGTTTGTACGTAATGTATGCAGAA
TTTTTAGACAATCTCTATGTTTTCTTAGATGATTTGTTCAAAACTTTTCGGCAGATCATTGAGCATTTATACAATA
AATGACGGCATATTTTGTTTCCATACACTACAAATGCATCTATTTAACACACCATCTTCTACAGTGAAACCATTC
ATCCTGTAACAAATAAATTTGGTTTCTTAAATTGACACATTTATCTATCTACCAAGCTGCAAGTCTAGTCAAAAT
TTGTTGTTGCCAAAGGAATCAAGAAGAGGTGACAAAACCAATTGGTTTTCAAGCTCTAGCTACTAGTGAGCTT
GTTTAAAACAAGTATATATTTGGATCTACAAAAATAAATGGAAATTATGCGTGAACTATTTTCATCCGCATCATG
TGCGGAAAAAACAAGG

>chr3D:479791819-479793319

AAGAAAGACAAATATATCAAACATTGACGCTCCATATTCAATATTTTTCAAATCCCGCAAAAAAAACATTTGTG
GATGAAATTTACATTTGCACTTAATATTTCAATGCATACAATGATACAGCCGTTACTTTCTTTCAGAATTTTCCAA
TGTAAAAAACAGGAAAAAAGTCTAACAGCACTGGAGTTCGAACTTCAAAACGAATTTCCGGTCAAGGCCATTG
CAACTCGTAGCGTGGCCATTAAAGAGTCAATCGATCGATCTGCAGGTGGAATATACTGTTGACAACCATGTTG
CAAATATGACACAGGAGTATTCAGAAGAGTTAATTTGATGCATACAAGTCAAGATAGTCGTCTACTCATAATTA
CGCCTCGCCACTAGAGTTAATTTGATATAAGTACCTATTCCTTACTGAATGGTAGTTCAACTCAAGCATTCGACA
CCAATTTTTTTTTATGTTTTGATTGCACAGGATTGTGATTAGGGATGAACATCTGACCATGCGCATGAACGAAA
CAACGACAAGCAACGTTATGTAGACAACAACTTTCTTTGTCTTTTATTCCCAAGTTTGGCTAGGGTCACACATAT
TGGTGCGTGACACCTTATTTATCACCGATACATCGACGGTCCATCGGCTAAACACCGATGGTCCATCTATCTAG
TACTACAACTCTTATCTTCTTAGCAACCACCAATGCCGGCAGCCATACCGCCGACCAGGCCAGTGGAACAGTAC
GGTGGCACCTGCACATTGCACATCGCCGGTAGGGTCTGCATCGCTACCACCTTCATCGCCTGGTACTGAGATA
GCTGTTGAGGTTGGATGATGACCTGGCCCGCCATTTGTGCCTGAGGCTGAACGAGACCCATTTGTTGAGCTTG
GACGAGTTGTTGTTGTTGCTGCTGCATGATGATGGCGTGCACCATGTTGTGGATGGCCGGGCACCTGAGCTGC
CGGGGGATTTGCGCCAGCTGCCGGCAACACTGTCGCCGCATCACGTGGCAGGTGCTCTGTCGCAGGATCTGC
GACCGGAGGAATGGCACCATCTCCACCGGGCTGCACTGTTGCATGAGGAACATCTGGCACGGGATCATCTGTT
GCTGCAGTAATGGCTGCATAAATTGTTGTTGTTGCTGCATAAATGGTTGTTGTTGCTGCATAAATGGTTGTTGC

TCGGGGTAGGGCTGATATTGTTCGCTGGGGTCATACTGGGCTGTGGCCATGGTCGCCGCCATGGCGAGGAGG
GCAAGGATGAGGAAGGTCTTCATGGTGGATTTGAGTTGACTAGTGCTTGCCCGGCTTTGATGGTTGAGCTCAA
GGTGAGTGAATGATGGATGATGGAGTAGGATCATCACGGTGCAGGCCTATTTATAGCTGCCATGGAATGGTTT
CTTTCCATCTTTATGTTTGCTTTCGTTTAAAGTGCTTGGCTGCTTGTCGAACTGATCAATTGGCGTGTTGTGGTT
GGTGGGGACTACTTACAAAATGTAATT

>chr4A:121020740-121022740

CTTGGTCCTCTTCCAAATGTTGCCTTGCCAATGCTTCCTTGACCCTTGCATCAAACTCATCTTCACTCAAGTATTC
TTCGTCGCCATTGTCATGGATGAACAAAACTTTCTTGTTTCCACATGCGGCCGACTTGTGTCCTTTGCCTCCACA
CTTGTAGCACTCACCATTGAAAGGTTGATCATCCCTTGTGTATTTGCCACTTGGGGGCTGTTTTCCTTGAGGAA
GTGTTGGCTTGAAGGGAAGCTTGGTGGTACTTGATGAGCTTCCACTTGGTGTTGTTGGAGTAGTGGTTGGTGC
TTGCTTGTAGGTGAAGTACGTCTTTGGCTTGTTGTACTTGATGTCCTCTTGCACTTGACGCTCGGCCTTTGATGC
GATGTGCACAAGCTCAACCATGTTGTTGTACTTTTGGAACTCGGTGATGCGCTTGATTGGATGGTTCAAGCCAT
TGAGGAAGCGTGCCATTGTTTGTTCATCACGCTCTTCAATGTTGGCACAGATCATGTTGAGCTCCATTTGCTTGA
AGTACTCTTCCACGGTCTTTGTCCCTTGACTAAGCGTTTGAAGATTGTTGAAGAGGTCACGCGTGCAGTGCGAA
GGAACAAAGCAGGATCTCATATGGGCCTTCATATCTTGCCAAGTTGAGATTGGAGGCTCTTCTTTTGCTTCTCG
AGCGAGTTGGACTTGTTCCAACCAAAGGTTGGCATACTCATCAAATTCGAGGGATGCCATGGCCATCATATTCT
CATTGCTATAGTTGTGCATGCGGAAGAGCTTGTCAACCTTGAGTTCCCACGAGAGATATTCTTCGGGTTCATTG
GAACCGGTGAACTTGGGCATGGTGAATTTGAGCTTGCCGAAAGTTTCTTCTCCATTGCGGTTCCGGTGTCGAC
GGTTGCCATGATTTTGGCGGCCATCTTCATCATCCGAACTCTCATGTCTTGCTTGGTGATGGTGGCGTTGCTCTT
GCGGTTGTTGAGGTGCCGCGTTTGGTGGTGGAACTTGGTTTTGACGACGAGCTTGGGGAGCTTGCTTGTGTTG
TTGGCGTTGTCGAGGACGAGGAGGAGGTTTATGGTCCTCACGACGAGCTTGGGGAGCTTGCTCTTGTTCTTGT
CGTGGTTGATGCCGAGGGGCATTGTCTTGCGCCATGTGGCGAGGAGGACGTCGAGCCGCCTGCGCAATGGGC
TGCTCTCTTTCTTCTTGATGTGGAGGAGGCTCGTCATGATGACCATGTCGTCGACGTTGACGTTCATGTCGACG
ATGACGCCTTGGTGGATCTTCGAGTGCTTCACGCCGACGGCGATCTTGTTCTTCCACTTCGGATGCATCATTTGT
TGTTGAAAGAACATGTCGACGGCGATGTGCTTGGTGGTCCATGTGAGGTTGTTCTTGTTGAATCACAAGTTCCC
TTGGTTGCGGCCGGTCCGCTTCACGTCTTGGAGGTCTTGCGGAGAGGTCACCATCATAGCTTGACATTGTGGC
GTGTCGCGGAGATGGTACCGCTTGAGAAGAGCTCATGGATTGAAGGACAGGTCCATCCCGGTGCCTTGAAGC
GTGACGGCGAGGTGCACTTGATTCACTTCAGGAGTGTCGTGATGAGCTTGAATCGCGCGAGCGTGGCCTTGCT
AGCCCATCAAGGAGGCGTTGAAGCTTCTCATCCGTTGAGGCGAGGGCGGGACCAAGTGCTTCATTTTGATGAC
CGAGGTTGTGTTGCATGGCATGCATTTGGCCTCGGACTTCTTCGAAGCGGTTGTCGATGTTCTTGAATTCCACG
CGGGCTTCGCTAAATTGTTCATCTTGGCGCCGCTCAAGAGCTTCACAACGGTAGTTGAATTCTCCTCGGACCTT
CCAATGATCCCACCTTGTCATGTATTGTGTGTCGTCCTCCGGAGGTGGTGGTGGTTGGGACGGTTCGCCATTCC
TGTCCATGATGCAAAGCCGACAAGGGTTGTATATGGAGAAAAACGGTGCGTAGAGTTAGCCTTTTACCTCACC
TTGGAGA

>chr4A:199198675-199200675

ACGTGTCAACACTCAACTTATATTCCATCAACACAAACACAATCACAAACTCCCAATAATAACATCTCCTCAAAG
TTCATGTTACTACTAGCCTCCAGGTACATGCATGCATGCACACCACACGTACGTACGCTCACGTACGAACCATC
CATGCACGGTGGCCCCAGCTTTATTCAGCATATAAACACGCATAATATGTCGAGTAAGTAGATACTGCAGAGT
GACCATGGCTCAGCTGCCTCAGCATCGACCGGTCGAGTCGAGTGACCTCCAGAGATCAGTTGATCTTGGAGCA
GTCGACGGTTGCGCTGATGTCGCCGTAGGGGACGGTGACACCGCACTTGGTCGGGATGTTGGCGGCGTTGCC
GTTGTTGAGCCCGCTCATCCGGCCGGCGGCCAGCTTGAGGCAGTTGCAGGCGGCGCGCCTGTCGGCTGTGGT
CTTCGTGGCGGCGGAGAGGCTCTTGACGCCGCTGCAGCAGGCCGCGGACGGGGCGGTGGCGAGGCCGCGCG
CGTAGAGGATGCAGGAGCTGAGAGCGGCGTTCACCTGTCCGCAGGTGATGGCCGCGTCGGCGGCGTGCGGC
GCCGCGAGGAGCATCGCTGCCACCGCCAGGGCGACCAGCACGAGATGATCAGGTGTTTCGCGGGCCATCTCT
CGATCTGTCGAGCTAATTGGCAGCAGTACGAATGGCTAGTAGGTTGGCACCTGAGGTTTTGGTAGGTTGTGAG
ATGAGGAGGACGATATAATACGAGGGAATGTGGGAGCTTAAATATAGCTAGCTAGGCCGGGTGGATCTGGC

AGCATTCTGATTTGAGGTGGTGCAGTGATTAATTATCCCAGCCGAACACACGACTAGATGGTTGAGTGGAGTG
ATGATGGTCTCTGGGTTAGCTAGGCGCGGGTGTGGTAATTAAGCGCCGGTGCTGGGGAGAAAAGGAGAG
AGAGGTGATACTCAAACGTCTCGCACCGAACGGTCGTCTAACCCTTGCAGCATCAAACTTTCGCATGCAAGATT
GCTCAGCTTGTTGAAAGACTTTTCTCTTGGATCACTGAAATACGAAGTCCTTCGTCTCAGAATAGATGTCTCGAT
TTTTTTTTAAATTTGAATAAACCTATATATGTACTGGCGGTCAACGGCGGAGCTTCGAGAGTGCCCGTTGGCTT
GACATGGCACTCGGATGCCGGGACGGCGGCCCGGAAGGTGGTTTTTTCTTTTCACCAAGCTAGGAGGTGGCTT
TGCGGGAGAGGTTCTATGCTCCGGCGCCTAGTTCCATTTATGGCCTCAGTTCGTTTGGCGCGAGGGGCTGGCG
TTCTGCGTGCGGGTTGGTGGTGGTGGTCTGGTCGGCCCTCGAGGGGCGGGTGCGAGGCTTGCGACTCTCTAC
CGGGCGAAAGTCTTGTTCTGGCTCCGGCCGGAACCGGCGGCGACGGCGCCTTTTGGCGCCGTGTCCTTCTTGG
AGGCGTCGTCGTGGTGGTGCTCTCGTACCTCTGTCCGGGTTCTCCGGGGGAAACCCTAGATCCCTCACGGGAT
CGGGCGTGGGCGGCGCTTCTTGCGTCGTTTTGCCTCTTGTGGTCCTCACTTTGGATGTTCCGTCGGCCAGAGGG
ACCAGTGGAGTGAGTTGGTTGTTGGTGACGATGAGCTGCTGGGCTACCTCGACTCGGCGGTCAGGCGGACAC
GACTCCGGTTTGGTGTGCTCCGGGGTGAAAACCCTGCACCGGCCTCGGCCGATGCAGGTGATGGCGGCGCTC
TCATTCGTCGCTCCCCTTCTTGGAGGCATCGCTGTGGAGGCACTTCACTGCAAGTGTCGGTGTTTGGTTTGTTT
GTCTTGTCGGTGTCGTCCTTTGATCGTCTTAGGCTAGGTGGTGCACGGCCCCAGGGTCGGCGTGGATGTTGGA
GCGGCGGCTCCGAGTTTTTGCCTCCGCCTTGTCGCGTTTTGAGGTGTTTTGGGTGACGGCCTTGGCCACTTGGG
TGGTTAGGCCGTCGACTCGTGTGGTGCGCGGTC

>chr4A:256517326-256518626

CCATGACTCCATGCAAGAGCACTTTATTCATGAAGGAGTTTACTCTAGTCGAATACATCCAGTTGCATTTATTAA
AATCTAGGATCGGACAAACTTATTCTTGTCTCTATGGATGGGATCACTTCCACCGTTCCATGGCCGGCCGCTTA
CTTAGCTGCTCATGGGCAGAAGATGACCTGGTAGTTGGTGCCGCCGGGGCAAGTGTAGGTGCTGGTGGCGTC
GTCCTTGGGGTAGCTGTACGCGTCGGAGCACTGCCCCTTGAAGAACCTGGAGTAGTCGGTGGGCCCGCAGTT
GTCCGCCGCCGAGCCGGTGCAGCAGTACTTGTCCTGCTTGAACACCGTGCAGGCGTTGTTGCACCCTCCGGTG
GCCTTTAGCTCGTTCGGGCACTGCGGCGTGATCACCTTGGGGCACCGCGGCCCGCCCTTGGGGCAGCCGGCGC
CGCTGCCGGCCGGCAGGAAGTTCATGGGCACGTTGAAGCCGTCGATGAGGGAGATGTCGAAGAAGTCGAGG
TTGTTGAACTTGTTGAGCCCGAACTCGGCCAGGGTGTTGGGCGCCTGCCCGTACTGCGTGCACTGCAGCTTGC
CGCCGCAGTCGCCCGTCTGGCACCGCCCGTTGCCGCTGCCGTCGAAGTTGCAGCCCGTGCGCGCCCACACGCG
CCCGCCCGTCGTGCCGGCAGGCACGTTGATGTTCCATGACTGCCCCGGGTCGAGCTTCCTGCCCCCGCCCGCC
GGCACCGCCGCCGCCCACACCGTGTACTGGCACTTGTTGGTGACGGTGAAGGTCGCCGCGTCGGCGGCAGCG
ACGAGCAAGAGGAGGAGCACGGGCAGCATGGAAGAGGTGGAGAGGGATGCCATGGTGGTGGGTGTGTGGG
GAAGAAATGGAGTTAGAGATCATGGATTTATAGGGTCGGATAGAGTTGAATTCAACGTTTGCAGCTTCTCGAC
GTGATTGTTTTTGGCAGGTCACGAGCCAGGCCGTTAGGACGTACTGTATGACTCATCGACGAGGTAGCTGGAC
TCCAGCTACTGGAGTGCTGGACACGAGACTACTACCCGCTTCATTCCCAGAGCTGCCGACTGGGGTGGAGTCC
TGCTCGAGCAAGGCTTGCATCCCGTAGCATGCTTTGAGCGCCCTCATCACGACAATGCAATTCCCCCCGTAGGA
GGGGCCCCGGCATCATCTCTCCTCTCCATTTGTTGGAGATTCGTGCTATGCGTTTTACAAAATAATCCTTCCACC
TCATCTCTCAGGCCGGCTGCAGTTTTTCTATCTAATCCCTAACGTTTTCGGATATTCTAAA

>chr4A:281117931-281119431

TGCTAACTCCAACACCCCATCCTACTTGAAATAAACATTATAAAGAATGGCGTCCAACCAGCTCATCCTCTCCGT
CGCCGTCCTGCTCTCCGTCCTGGCCGCCGCCTCCGCCAGCGTCGGGGATCAGTGCGTTCCAGGGTGGGCGATC
CCGCACAACCCGCTGCAAGGCTGCCACTCATACGTGGTCAGCCGAATCTGCGGCGCAGGGCCATACCTCGCCA
CCGAGGTGATGAAGGAGCAGTGCTGCCAGGAGCTGTCCGCCATCCCGGCCTACTGCCGGTGCGAGGCGCTGC
GCATCCTCATGGACGGGGTGGTGACGGCGGAGGGCGTGCTCGAGGGCGGCCTCCTCCAGGACTTGCCCCGGT
GTCCCAGGCAGACGCAGAGGAACTTCGCCGCCAACCTCGTGGCCCCTGGGGAGTGCAGCCTAATGACCATCC
ATGGCGGCCCGTACTGCCTCACCCTGGTCGGCCGTCAAGTGGCCGTGTAACTTCTGTACTGTCTAAACTGCTCG
TGATTGTGAATAAGCGTGTTGCATCGACTGATGGTGATGGACGCATGTGTATGTGCATGCACCTATATGCGCA
TGTTTGAACGATAATAAAGAGCATCCTAGTTACATTTTGATAATTGTGCAAGGTATGTTTGGTGGGAAAAATAG

TTTTAGACATTTAAAAAATTCCAAGGCAGTATAATTACCTCGAGAGATGACCATCTGAAATAAAAACGCTCCCT
CCATTTTTTATTACCTAGGTTAAAGTCAAACTTTGTAAAGTTTTACCAAGCATTAAAAAATATATTAGTAACATCT
ATAATACTAAATATATATATATAATATAAATATACTTTATAATAATTATTATGATATTGATTTTATATTATAGATG
TTGATATTTTTTATATATTTGGTCAAATTATATAATTTAACTTCGACCAAATCCAGAAGACAACTCAATTGGAGG
GAGTACGAGTTTTAAAGTAGAGCGCTGAACTCCCTAGAGCTACAAGGTCAAGGCTAATTACAACCTACATTTA
ATACGAACTTCTGGCTATAAAGAGTAGCATACAAATCACACAACCACCAAGACTTTTGTCCCAATAAAGTTGGG
GTAGGCTAGATATGAAACCCAAACGGAAACTAGAGAAAAGTGCAAGTCAAGCGAAAAACAGTTAAAAGTGCA
AGTCAAACAAGAACTAGTGAAAACAAAGCAAGTAAAAAGAACAAAAGAAGTAAATCTAGGTTCAGGCACATG
GATTACTGATTTTCATGCATCCCTATCAAAATCAAATCTCTAGGGACATTTCAGTCCTTTATATCCCTTTTAACTA
CCTCCTCCCATGTCAGCTTTCGTCGTCCTCTACCCCTCTTTGCATTATCAAAACACTTAGAATTTCACAGTTAACC
GGTGCTTCTGGTGGCATCCTTTGGACATGCCCAAACCACCTTAATCTGTGTTGGACAAGCTTCTTTTCAATTGGC
GCCACCCCTATCCTCTTGTATCA

>chr4A:299791997-299793997

AGAAGAAGTAATATTGCTTCAATTAAATAAAAATGAAAGGTAATTCAAGCAAACGATTCCATTTCTTGAAAAAC
AACAAAACAGTTGGTCGACTGTCTGACCATGATGATACAGCACATTTTCGTGTGTGGAGCAACGATAGATACT
CCAGAAGCATAGCATAAACTATCAGTACATGATTCCGAAGAATATGGAAGATCGAACGTCAGGTGGCCTCAGC
TCATGGTTAGACAGACGGCGAAGAAACACAGGTACCAATTTCTGAAAATTACTAATGCCATGAGCTAAGTTTT
AATTTACTACTACTACTAGAGATGATGGTCACAGTAACAAGCCAATCACAAACCAATGTCCCGATTCAATTAGA
AGAGCAAGTGACTGAGGCAAGTAGCTTTCAAAACAATAATAATGCCATCATGTTTTGTATAGTATATGTAGGA
AGTTAGCCACCAAATGATAATAATAAACGAAACAATGGAAGGCCTGACTGAACAGCTAATAACCAATTCTCTG
ACAAGTTCTACCAGAGTGAATGAACTAATCTTTACTTCTGCAGGTAAGCAGGCAGCTACTACTACTTGTGAAGA
AAAACAGTAAGAAAATCGATTTCCAAGATGGGAGCAAGGTGTGAAAATGATACTACTATGGGTTCTACCTTTG
ACGAAAACACGGCGAAGCAGGTTGCAGATTCGGTCGCCAAACATAGCAGCTGTGCCCAACGGCAGGAAGAA
GTGAGGCTGCCGCAGCATGACCTAGCAAGGCATGACCACAGACGCATATACATGTATGCTGCTTGTTGATCAC
TCAGAGCTAGATCATGCTGGCAGCTTCACTCGTTCCCTCGGACAGCCGATCTCTCCAAGATCAAGGTCGCATGA
AATACAGGGAATACAAGAGAGGCAATCACGGTGAAGAAAATCCACGTAGGGAGGAAGAGAGGGAGAGCGA
AGAAGACTATCAATCACAAGCTGGCCAAGAACATGTGTGGGAAGGAAGGTAGGTAGCTAGCTAGCTGCTACA
AGGTGTGCTCCCATGGACATTGCCTACATAACATGGAGCCATCGGCCAGGGATGATACTTGGCAAGAATTCAA
GGAGAGGATGAGGAAGCCACAAAAATGGTGTAGATCACATCTCACCTCAAGAATCAAGGATGAGAGAGAGG
GGAAAGAAGAAGAGGAGAAGCATAGAGAAGGAAGAAGGCAAGGAGCAAGATTTATAGATGGAAGGGCTGT
GGTGGCTTGTGTGCCCGGGGTGGGGTGGGGTGGGGTGGGGAAGATGAGATCCAGAGATCCAACCAGCAGA
GATGTAAACAATATTGCAACTCTCATCAGCTGTTCAGAATAGTGGTTGAGTGGGCAGTAAAGTAGAGAGAGAT
TAGAGAGATGGCAGAAGAGGCCAAGGAGGCTAGTGGTGTAGGGGGAGGCCTAGAGGGGCTAGGGTTCTAG
AGCGTAGAGTAGCTAGGGGGACCTAGACAGCCACCGCTGGCAGAGATGTCAGGACAGGAGGGGGTGGGTG
CAGAAGAGGGGGCAGGAAAAAGAGGAGGGAGAAAAGATCTCGCGTCGCGTCGGGTCGAGTCAATCTGATGC
TTGCATGATGGATGGACACTGGACAGGGGATCAGGTAGCTAGACGGGTAAGAAGAGGGGAGAGCTCAAGAG
CAAGACAAACCCCAGCCCGGCCGTACTGCTTTTTTTTTCTTTAGGCCTCTTTGGACGCACGACTTTTGGAATATA
GGAATAGAGAAAAAATACACATGATTTTATTCTGAAATACAGAATTTTTTTAAAAGTTGTCTTAATTTGAATGG
TGCAAACGAGAGGAACACATGAATTTTATAGGACTTTCATAGGAGAGAGAGCAGATACAGATGGAGTGTAAG
TGGATTGAAGCTTTGGGTAATAAAGCGAGTTTCTGAATTCATGTTGAGATTTCTATGAAACATAGGTCATAGAT
TGGACTTCTTTGGATGCAAGACTTTTGGAATAATAGAGAAGAGAAA

>chr4A:316744313-316746313

TTGTCTCTTCAGCGCTGGAGCTGGCAGCTTTGAAGTCTGCATCATCGTCTTCCTCCTCCTCCTGCCACAGGTAAG
AGAATAGCGTTTTGGGTTAAGACGAGACCCCATCAAATATGTAGCTAGAAACATTCAGGACACATAATTTGATT
TGCAGCTTACATCTGCCTCATCGTCGATGTCTTCGTCCTTAGAGCCTGCAGATTCCTGTCAATTTGAAAACACTG
GCTAGTTAGTACCGAATATACAGTAAAAGTGCAAGTGTGATGCTGAAGAGATGTTTGCATACCTCCTCAAGCC

TCTTCTTCTCGGCCTCGTCAAAGGCAGCCTTCTCAGCCTGCAAAACAAAAGCCATTGTCTTAGTTTTGTACAGCC
AGGCATTTAAAACCAGAAAAAAATTGAAATGCTAAAGATAGGGCTTGCATACGTCTTTATGCTTTCCCCAGGTC
TCCTCTGCAAACTTCTTGGCATACTCAGGGTCATCCGTGATCAGGATGTTGTCGAACAGAGTTCCAGATTTAAC
CTAGTGACAGTCAAACAAGAAATCATTATACGTAGTTGCGACAATAAATTCAGAAAATGTCAGCAATGTGTCA
CAGAATTTTATGCTGGCACTAACCTGCCACAACTCAATGCCGATGTGCTTCAAGCTGTCAAAAGCGTAGATGTA
AGGATCATCCTTGTACTCTGCACAAACAAGGAAAAAAATCAGTAATGAGCCTTGATGTTTCACTGAATTAATTG
AACAGAAAAGATACATGGACACAAGCTAGTCTGAATGTACAGCAGTGGGTAAGCTCAGGTTACCTGGGTTGTC
GATCAAAGGAGCCTTCCACTTGCCCTTGAAGTTCGGGTTCTTGATTTTCTGAATACCCAAATCTCATGTCAGAA
GTTGCAATGCACATTGCTGCTTTGTGTAGAACAAATTAACTGTCTGATCATAATGTCATAGTAATACCTTTTGTG
TCCATGGTCCCTTGTACTCGGGGTTTGGAATGGTTGGAGCTGTCCATTCACCATCTTCCTCCTCATCCCAGTCCT
CAGGCTGACATAGGTATTAATCAACGTCAGAAATTTCACACACTTGGAGGTGACACAACACAGAGTAAAACTG
TTGTAAGAACGGAGAAATAGGCATACCTTTGTTGCATCAGGATCAGTAAGTTCCTTTGGGATGTCATCATAACC
CTGCAAAAAACAATTTGTGTCAGACAAGAAGCCACCAGAGACAAAGAACAGTTTATAATTGTATAGTTTTGCAT
CAGGATGAGTAAGTCTCATGCTGCAGAATATGGTGTCAACTTTGGCTTGGATTGGTTAGAAATCAATACTGTGT
CTACTTTGGCTTAGCTTGGTCAGAAATCAATACTCATCACAAAATCATCGTATACATTATAACATATAGCAAAAA
AGGGAAAAGCTACCTCTGGCTTCTTATCCTCAGGGTCAGGAATGTATTCGTTGTCTTCCCAGTCTTCTGGCTGC
AACCATCAGCACAAATCAAATTATCACCTCTGTCACAGAAACACAACGATCATGTATGGCTATTTAGAATACAA
CAATCTCTGAACCTACCTTCTTGGCGTCAGGATCCCTCTTCTTCTTCGCGGGGAGAATGTCCCAGTCATCATAGA
CACTGCCAGATTGCTTCTCGACATTGTCAATGAGGATGCTGTACGTGGCGTCAGGCCGGATGATCAGTGTGTA
CACGTGTGTCAGCTGGTCCGTCTCGCAGGGCACCTCCTTCTTGATCAGGTTGTTCTTGCCGTACTTGGTGAGGA
TCGCGTGCACCTTCTTGGTGGTGTACCCGCAGATGTCCGGCCCGAACATGATGCTGCAATTCCAAGGACACGA
ATGATCAACCAACCAAGTAACAAACAATCGGAGATGGATTATACACCAACAGATAACGGTGACGAGGATGAA
CCTGTAGGGGGTCTCGCCGCCGAACTTCTTCTGGTCGACGTCGCCGCCAAGCAGCTTGACGTAGCCACCGCCG
CAGT

>chr4A:323535547-323537795

TCCACCTTCTACAATCTCTTCAAACAATCATGGCAACTACTAGGTTTCCATCATTGTTGTTTTACTCCTGTATTTTT
CTCTTGTGCAATGGGTCAATGGCTCAGCTATTCGGGCAGAGCTTTACCCCATGGCAAAGCTCTCGACAAGGAG
GCTTAAGGGGGTGCAGATTTGATAGGCTACAAGCATTTGAACCACTTCGACAAGTGAGGTCACAAGCGGGTA
TCACTGAGTACTTTGATGAGCAGAATGAGCAATTTCGTTGTGCAGGTGTATCCGTCATCCGTCGTGTTATTGAG
CCTCAAGGCCTCTTGTTACCTCAATACCACAATGCTCCTGGCTTGGTGTACATCCTTCAAGGTTAGTGTCTAATT
GAATATAAAAATTGCCTTTGTTATACTTCACTTAGGATTTATATGTGCCAAATGTTACACCGTTCATATTTTTTAA
CAATGAAACAAATGTGTTTTCTTTTAGGTAGGGGATTCACAGGGTTGACTTTCCCTGGATGCCCGGCGACCTTC
CAACAACAGTTCCAACCATTTGATCAAGCCCAGTTTGCTCAAGGTCAAAGCAAAAGCCAAAATCTTAAGGATG
AACACCAAAGAGTTCACCACATCAAACAAGGAGATGTTGTTGCTCTACAGGCTGGCATAGTACACTGGTGCTA
CAACGATGGTGATGCACCGATTGTAGCTTTCTATGTCTTCGACGTAAACAACAACGCTAATCAGCTTGAACCAA
GGCAAAAGGTAACTATACAACTTAATGCACACAAAATATATACAACTATTTACAAGTGACCCAACTATGATTTA
AAGTATACATTAGTGGGATATTAATGAACTCTGTTTAACTTCATCGATATAACATTTTAGGAGTTCCTGTTGGCT
GGTAACAACAAGAGAGAGCAACAGTTTGGACAAAACATATTCAGTGGATTCAGTGTCCAACTTCTTAGTGAGG
CCCTTGGTATAAGTCAGCAAGCAGCACAAAAGATCCAGAGTCAAAATGACCAAAGAGGTGAGATAATTCGTGT
GAGTCAAGGCCTTCAATTATTGAAGCCTTTTGTTTCGCAACAAGGACCAGTAGAGCATCAAGCCTACCAACCAA
TTCAAAGTCAAGAAGAACAATCAACCCAATACCAGGTAGGGCAATCACCACAATATCAAGAAGGACAATCAAC
TCAATACCAGTCAAGACAGTCATGGGACCAAAGTTTCAATGGTTTGGAGGAGAATTTCTGTTCATTGGAGGCA
AGGCAAAACATCGAAAACCCGAAACGTGCCGACACGTACAACCCACATGCTGGCAGGATAACACATCTCAATA
GCAAGAATTTTCCCACCCTTAACCTGGTGCAAATGAGTGCTACAAGAGTAAATTTATACCAGGTATTTATGATA
CTATATTCAACACACTATCTTATTTTTAGATATTCTAAGCTTCATACAACCGGTTAATAATATGGCATACAAATAA
TTGCTATTGCAGAATGCTATTCTTTCACCATACTGGAACATTAATGCTCACAGTGTCATGCACATGATTCAAGGA
CGTGCTCGAGTTCAAGTTGTCAATAACCATGGTCAGACCGTATTCAATGACATTCTTCGTCACGGACAACTGCT

AATCATACCACAACACTATGTTGTTCTCAAGAAGGCAGAGCGTGAAGGATGCCAATATATTTCATTCAAGACCA
ACCCCAATTCTATGGTTAGCTACATCGCAGGAAAGACCTCCATCCTACGTGCATTGCCCGTTGATGTCCTTGCCA
ATGCATACCGCATTTCTAGGCAGGAAGCCCAAAACCTCAAAAATAATCGTGGGGAAGAGTTTGGTGCATTCAC
CCCTAAGTTTACACAAACAGGATCCCAGAGTTACCAGGACGAGGGAGAGTCATCTTCGACTGAGAAGGCATCC
GAGTGAATAAGTGAGTGTAATGGAAACTAGTATAGTGAAATAAAGGCATCGCATGTTTGCAGCCTAGTGGTAT
ATAACCGCTTATCTCAATAAAAAAGTTTCTCGTGTTATATTGTTTGCTTGTTTCTTGTACTTTTCTTAATTTTATCT
TTTATGATCAACCAACTTACCTCTCTTCTTCGTTTTTTGCCACTCTTCACATATGCATATCGAGGAGACGTATAGT
CCACTTGTACTAGATTTATTTTTTGGTACTATCTTTTGTGGTTCGAGTATTTCTCGGGTCCGATAAAAACCGTCA
ATTTACTCATGCTAGATTTTTTCA

>chr4A:323557681-323559795

ATGGCAACTACTAGGTTTCCATCATTGTTGTTTTACTCCTGTATTTTTCTCTTGTGCAATGGATCAATGGCTCAGC
TATTCGGGCAGAGCTTTACCCCATGGCAAAGCTCTCGACAAGGAGGCTTAAGGGGGTGCAGATTTGATAGGCT
ACAAGCATTTGAACCACTTCGACAAGTGAGGTCACAAGCGGGTATCACTGAGTACTTTGATGAGCAGAATGAG
CAATTTCGTTGTGCAGGTGTATCCGTCATCCGTCGTGTTATTGAGCCTCAAGGCCTCTTGTTACCTCAATACCAC
AATGCTCCTGGCTTGGTGTACATCCTTCAAGGTTAGTGTCTAATTGAATATAAAAATTGCCTTTGTTATACTTCA
CTTAGGATTTATATGTGCCAAATGTTACACCGTTCATATTTTTTAACAATGAAACAAATGTGTTTTCTTTTAGGTA
GGGGATTCACAGGGTTGACTTTACCTGGATGCCCGGTGACCTTCCAACAACAGTTCCAACCATTTGATCAAGCC
CAGTTTGCTCAAGGTCAAAGCAAAAGCCAAAATCTTAAGGATGAACACCAAAGAGTTCACCACATCAAACAAG
GAGATGTTGTTGCTCTACCGGCTGGCATAGTACACTGGTGCTACAACGATGGTGATGCACCGATTGTAGCTGT
CTATGTCTTCGACGTAAACAACAACGCTAATCAGCTTGAACCAAGGCAAAAGGTAACTATACAACTTAATGCAC
ACAAAATATATACAACTATTTACAAGTGACCCAACTATGATTTAAAGTATACATTAGTGGGATATTAATGAACT
CTGTTTAACTTCATCGATATAAACTTTTAGGAGTTCCTGTTGGCTGGTAACAACAAGAGAGAGCAACATTTTGG
ACAAAACATATTCAGTGGATTCAGTGTCCAACTTCTTAGTGAGGCCCTTGGTATAAGTCAGCAAGCAGCACAAA
AGATCCAGAGTCAAAATGACCAAAGAGGTGAGATAATTCGTGTGAGTCAAGGCCTTCAATTCTTGAAGCCTTT
TGTTTCCCAACAAGGACCAGTAGAGCATCAATCCTACCAACCAATTCAAAGTCAAGAAGAACAATCAACCCAAT
ACCAGGTAGGGCAATCACCACAATATCAAGAAGGACAATCAACTCAATAGCAGTCAGGACAGTCATGGGACC
AAAGTTTCAATGGTTTGGAGGAGAATTTCTGTTCATTGGAGGCAAAGCAAAACATCGAAAACCCGAAACGTGC
CGACACGTACAACCCACGTGCTGGCAGGATAACACATCTCAATAGCAAGAATTTTCCCACCCTTAACCTGGTGC
AAATGAGTGCTACAAGAGTAAATTTATACCAGGTATTTATGATACTATATTCAACACACTATCTTATTTTTAGAT
ATTCTAAGCTTCATACAACCGGTTAATAATATGGCATACAAATAATTGCTATTGCGGAATGCTATTCTTTCACCA
TACTGGAACATTAATGCTCACAGTGTCATGCACATGATCCAAGGACGTGCTCGAGTTCAAGTTGTCAATAACCA
TGGTCAGACCGTATTCAATGACATTCTTCGTCGCGGACAACTGCTAATCATACCACAACACTATGTTGTTCTCAA
GAAGGCAGAGCGTGAAGGATGCCAATATATTTCATTCAAGACCAATCCCAATTCTATGGTTAGCTACATCGCA
GGAAAGACCTCCATCCTACGTGCATTGCCCGTTGATGTCCTCGCCAATGCATACCGCATTTCTAGGCAGGAAGC
CCAAAACCTCAAAAATAATCGTGGGAAAGAGTTTGGTGCATTCACCCCTAAGTTTACACAAATGGGCTCCCAG
AGTTACCAGGACGAGGGAGAGTCATATTCGACTGAGAAGGCATCCGAGTGAATAAGTGAGTGTAATGGAAAC
TAGTATAGTGAAATAAAGGCATCGCATGTTTGCAGCCTAGTGGTATATAACCGCTTATCTCAATAAAAAAGTTT
CTCCGTGTTATATTGTTTGTTTGTTTGTTTCTTGTACTTTTCTTAATTTTATCTTTTATGATCAACCAACTTACCTCT
CTTCTTCGTTTTTTGCCACTCTTCACATATGCATATCGAGG

>chr4A:338400411-338402411

TGGACACAACAAAGCAAAATTTATCGACATTACAACACTGCTGACACCGGTATGTGAGGGTGCCATTATTAAA
CGGAGTATTACAGGTTTTGTGCAAACTACTGCTCACTGATACAGTTGGTAGGAGCATATATGTGTAGGAGTAC
ACCGCGACAGCAGTACGCACATCTTATTCAGTAACTGCGGGGTGTAATACATAGTTACATACAGAAGACTCGC
TCAGTCTAACACTGAACGCCATCAACGAGCTTCGACAGTTTGATTCCCACCTCGGTTGATGACTCGGTCTACGT
ACTCCTTCCTGAAATCAATGCCGTGGTTCGATCAGCCAGAACAAGATATCAGTTGGAGGTATACAGGAAACAG
AGACAGGAAGGCAACGAATAGATCTGAAGACTCTGGAAAGTACCTAGCTGGGAGATGGTCATGGGGACGGT

TGTACGGTGTCCAGACAGGCTCACCCACAAAGAGCCGCATTGCCTGCGGAACATAGAAAAAAACAGAAGTTTA
AAAGCTCCATACTAGAATAATGTTGAACTTCACAAAGATGGTGCAGCAAATGTTGCCGCAGACAACTGCTTATT
ACGAATTATTTGCCGCAAATGCTCAAATAAGTAGCACTGCTGTCAAATTCAAATATTTGAGCTATCACCAATTCA
TTTACACAAACACAAGTAAACTACAGGCGACTTAACTAAAGAACACAGTTCAGTCATTTTAGTTCATTTAGTATA
TACTAGAATAATGTTGAAGATGGTGACAGACAAGGCAGCAAATGTTGCCACAGACAACTGGTTATTCAGAATT
ATGGTGCCACAAATGCTAAAATAAGTAGCAATGTTGTCAAATTCAATTATTTGAGCTACCACCAATTCAAATAC
TTGACAAGAAAACACAGTTCAGTCATTTTAATTCATTTAGAATACAAGTTTAAGGAGACCAGAAATCATGGTGG
GACCAAAGGAATAAAGTAAAAGATACCTTGATGTAGTTGTCACTATCCAAAGTAAAGCGGTGATACATTCCTG
CAGGCAAAACAATCATGCCCCCCTTCTTCACTGCTACACGGATCCACTGTTCGTTTTCATCCCTCAAATCAAAGT
ATCCTTGGCAACATAAGAACAAATAGGTAAAGCAGGTCGAAAGAATGCCACCATGATTATTACTGGAAATAAA
ACTGTGTATAAAGCTTTGCGATCTTAAATGCGAAAATAAAGGGGATAAAAGGGCACCTACCACTGCCCTCAAG
GCAATAGCGTATCTCTTCGTCAGTATGCAGGTGCTCTTCAAAGAAATTCTTTATCTTGACCTCATAGTTTGGCAA
CTTCTCGGGACACACATCGCAAATGTCCTGCAAAAGGAAAATAGAACATAATCTGTCACACTTCTTTGCTTCTA
GTACTTGCACACATTTCAAGCTTGCCATCAATATGGAAACAGAGAAGAGGGAAAAATGACTAACCACATAAGA
GTATCCCCTGGCCTCACGGATTTTCTTGAGATTCTCATCTTTCTCCCAGTCATCAGCATTCAGGCGCCAGCTTACT
ACACCCAACTCTGTGTATTTTGAAATGCATAAATTTAGCATCCAGCAGGTTTACAAGAACTACTTTTACGGTTTT
ACCACAGCTCATACACAAACTGCAGTGAATGATTTGAGCAGCATATGGACAGACCTGAAAGTTTGTTCAGAGA
AATGAATTCTTTGGGCTCACGGTGATGAGGAAGCCTCTGGTCTTCTTCACTGTCATCCATGTACCATGCTTGGA
TGACCTCCTCCTTGCCATCCTAAGTTAGTAATGGCCACACAAAAGATAACATGTCATAATTATGCAGGAATAAA
TATAAATCTGTACAGAAAAATGCTAACATCAATTAGATGTAAACGTCTACAATAATTTACATGGACCCTCCTTCA
ACTAATCAGAGTTGTTAAAAGACTACCTCCATAAATTTGTACTAACGTAAATACAATTTTGCGATTTGGAGGAA
AAAATATT

>chr4A:348745914-348746914

ATGGCGTCCAACTCCAAGTGCCATCTTCTCCTCTTGGCCGCCCTCCTGGTCTCTGTCTTTGCCGCTGCCGCCGCC
ACGGGTGATTACTGCTACCCATCGATGGGTCTTCCGAGCCGTCCGCTCGATGGCTGCCGTGAGTATGTGGCAC
AACAAACATGCGGTGCCCGTATCCTCGGGGCGCCGTCCGCCCCCATCGAAAAGTTGATGTATCAGTGTTGCCTT
GAGTTTTCACAGATTCGACAGCATTGTCGCTGCGAGGCGCTGCGCTACTTCATGGGGTCTCATCCAGAAAGAA
GTGGCCTCATGAACCTGCCAGGATGCCCCATTGAGGCGCAGAGGGATTTTGCCAGAATACTCCCCACGCCTAG
ACAGTGCAACTTGGTGACAGATTACAGCACAAGATACTGCTTGGAAATGGACAAGTTCTGGCAGTAGTAGAG
ATCGCTCATGAATAAGCATGATACATCCATGGATGTGCGTGACAAGCATAAGTGCATATGTGAGCTTCGTTCAC
CATATGCTATGATTTAGAATAAAGAGAATCATTTTATGGTTCTTAAATTCCAACTCAACCATTTTTTTTTATTTTTT
TTTGTGGTTGTGTAGCCTTATTGTCTATTTGTTTGCTTGTGTTGCTTATCATGTGTGTGGCATGTGCCTTATTTTT
ATGTTTCTATGTCTAGACAATATACATCAAAAAATCACTCTCGAGCTCGTCCTATAGGGAGCCATTCTCCAAATT
AAAAAATAAAAATTCTGAAAATTCGATATCAAAGTTTTAGAAAACCCAAAATATATTTGTACGTATCCAACTAT
ATATTCGTCCAGCGTGTAAATTAACAGATCAAAATACTTTTTATTTTAAGCTCAGAAAATAACAAAATCTCACAA
AACGACAAAAATTACACTTTAGTGCACCGTTTACAGCTGTTCATCCAAGACAAATTCTTGATTTTCCATTTTTTTC
TAGGGCCAGAATACAATGTATTTCGTATAGAGA

>chr4A:348747592-348748592

ATGGCGTCCAACTGCCGTCTTCTCCTCTTGGCCGCCCTCCTGGTTTCTGTATTTGCCGCTGCCGCCGCCACCAGT
GGCGGTGAATACTGCTATCCATCGATGGGTCTTCCGAGCCGTCCACTCTATGGCTGCCGTGAGTATGTGGCAC
AACAAACTTGCGGCGCCCGTATCCTTGGGGCGCCGTCCGCCCCCATCGAAACTTTGATGGAGCGGTGTTGCCT
GGAGTTTTCACAGATTCAGCAGCACTGCCGGTGTCAGGCGGTGCGCTACTTGATGGGGTCTCGTCCAGAAAGC
AGTGGCCTCATGAACCTGCCAGGATGCCCCATTGAGGCGCAGAGGGACTTTGCCAGAATACTCCCCACGCCTA
GGCAGTGCAACTTGGTGACAGATTACAACACAAGATACTGCTTGGAAATGGACAAGTTCATGTAGTACTAGAG
ATCGCTCATGAATAAGCATGATGCATCCATGGATGTGTGTGACAAGCATAAGTGCATATGTGAGCTTCGCTCA
CCATATGCTATGATTTAGAATAAAGAGAATCATTTTATGGTTCTTGACTTCCAACTCTACCATTCATTCGTTTGTT

TTGTTCTTGTATAGCCTTATTGTCGGTTGGTTGGTGTTGCTTATCACGTGTGGCACGTGCCTTATTGTTATGTTTA
TATGTCTGGACATTATACATCAAAAATCACTCTCGAGCTCGTCCTATAGGGAGCCATTATTCAAAATAAAAATTA
AAAAATCTATTTCAAAGTTTTAGAAAAATCTGAAAAAATATTTGGATGTATCCAACTATGTATTCGGCCATTGTG
TAAACTAACAGATCGAAATGCTTCGTATTTAAGCTCAGAAAAAATGAAAAAAAATCACAAAACGACAAAAATT
ACACTTTCATCCAAGACAAAATCTTGTTTTTCCATTTTTTTGAGCCCAGAATACAATGTATTTTGTATAGAGATTC
TACACACTGATAGACTAGAGTATTGAGTATTCCAGC

>chr4A:348749421-348750421

ATGGCGTCCAAGTGCAATTGCAGTCTCCTCCTCTTGGCCGCCATCCTCGTCTCCGTTTTCGCTGCCTCCGCCGCG
GACAATCCCTGCTTCCCAAAAACGGCGCCTACTCTGAGCAGTTTGCTCCAAAGGTGCCGTGACTATGTGGAAC
AACAAACCTGCGGCGTCGAACCCTCCGGTCCGTTTACAATTTCTGTAAGAGAGCAGTACATGGTGAAGGAGCG
TTGCTGCTGGGAGCTTGCCAACATTTCGCAGAAGTGTCGCTGTGAGGCGTTGCGCTACCTTATGAGGAAGACG
CCTGCTAGTCTTCCCTATGAAGTCAGCCTCAGGGGCATGCCCGGATGCCCAAGGGAGGCGCAGATGAACTTTG
TCCGAATACTCGTCATGCCGGGGCAGTGCAACTTGGCGACCACTCACAACGTTCGGTACTGCCCAGCTTTGGA
CAAATTTCAGTACTAGAGATACAAATCTGGCTCTCATGAATAAGCAAAGCGTGTTGCATCCACTGACGTGTGAC
ATGCTTATCGACACATGTGCGCTCCAGGTTCATGTGTGGTACTGTCTGGTATGATTAGAATAAAGAGAAACATT
CTGAGGTCTTTTTTTTGCCAAATTTCACCGGTTTATTAATAATCATCAACAGCAGTATAACGAGGCATACACGTA
ATATAAATTACAAATAGGTCATTAGGACGACTATAAACACTAAAGCGAACCGAATACACGCACCGTCGTCCTA
GGATAAAGAGAGTCATTTTGTGATTCTTGAACTGCAACCGAATCTTTTATACTCCCAGGATTTAGAAATTTGTGT
CGAAGATTTGTCGACACCTATTTTCGAACGGAGAAAATACAAATGCTTGATGGTCTTATAGGCTCATTTTTATCT
CTGTATATACGTCACTACATGGACACATTATGCAGGAACATGCGGGTGCCGCATTAGTGCGATTAATTTTTTGG
CAGGCAACGGTTGTTGCTTCCAGCTTACTATCATTTATGG

>chr4A:348754102-348755105

TGGCGTCCAACTCCAGTAGAAATCTCCTCCTCCTCTTCGCCGCCGTTCTGCTCTCTGTCTTTGCCGCCGCCGCCG
CCGCCGCCACCCGCGGTGACTGCTTCCAAGGGATGATGGGTAGTCCGAGCGATCTGATCCCAAGCTGCCGTGA
CTACGTGGAACAAGAGACCTGCGGCTTCGATACCGAAGGGCCACCATTCATGGCCAGGGAGGATTGCTGCAA
GCAGCTTGCAGCAATCCCGCAGCGCTGCCGGTGCGAGGCGTTGCGCTACTTCATAGGGCGGAGGTCTCGTCCT
GATCAGATCGGCGGCCTCATCGACCTGCCTGGATGCCCCAGGGAGCCGCAGAGGGACTTCGCCAGAATACTC
GTCACGCCGGGGCAATGCAACTTGGCCACCATTCACAACGCTCCGTACTGTCTCGCGATGGACGAGTCTCAGT
GGCACTAGAGATAGATCTCTATGGCCCATTAATAAGCATATTGCATCCGTGGATATGTGTGACATGCACATGTG
CGCTGCGCTCAGCTTGTGTCTGGAAGGCTATCTCTGCTATGAACGAGAATAAAGAGATCAAATCATTTTGTGGT
TCTTAAATATCAACTTCATTTTTATGCATATATGCTTGGGGTTTTGATGTTATCTTATTATTAGTTACTGCTTGCG
GCCCTTAGAAGCATTCCTCCATCCACTGGTTTGGCGTTCTAAGTCTGAAGATATTAAATTTTAGGGACAAGATTT
TGAAAAATGTTGCCTATATGGCATCGGCGTGCTATGTAGGAAGGTCAGACTTGGTAGGACCTGTTGTGAACCA
CCTATAGACTTTTAGGACCCATAATGCATCTTCATACAAGTAGGACTTACTTGCCACCCAAATAGTTTTAGTATC
TAACTTACAATTATAGTTGGACGCGAGCCGACTCGGGCTTGGCTCGGGCCGAATCCAGAAAAAGCTCGAGCTA
AAGCTGGCGAGCCCGAGCGAGGTCCACACTGAGGAAGAGCCT

>chr4A:370598026-370600026

GCAATTTCTCCAATGCCGACATCGCTCATCTCCACGACCTGTCCGTTCACAAATGACAGCCTCCCTTCGCTCACT
CTAAACGCTAATCGACAGTGCTCATTGGTTCTCTTGGGGTAATGATCAGGAAGGTGGTAACGAAGCTGATGTT
TCTTGCGTAGACCTTTTGGCGACCTTATTCGTGGTCTTCTTCTCCTATGCAACTATCTAGGACAGCAAATCCAGT
ATAACTATAATCTCTCTTTCGTACAATGCCGAAGCAGAAGCACGTTATGCTTATATAGACTGCTCGGCTGTATG
AACTTACCAGGAGCTGTGTAAACTCTGCAGCCGCTGCTAATCGAACCTTTGTTACGTGATTCAGTAGTTTCTACT
ACGTAGTAGTACATTTTAGTCCGTGTTGATACCCTTTGTCATCTGTGGCTTACTGTCTTCGTTGAATCGGAATCG
CTATATATTTGATCAACTGGTATGTGTGGCTGTTGACTATTCACTGGATGCCACTGTTTTCATCAGGGTCTTCCG
TTTGGTCACCTGCCTTCACACAAGGTAGTCATCTGCAAGTGGAAACTGAGGGTTGGTGCTTTGGTGGTAGGTT

AATCTGTGTGATGTAATTAGCGCGTGCTCCTACGATCCTGCCAGTGTAAATTGTGTGGTTACTTCTGTTGTGGTT
TATCAATGTAGAGAAATAGCCCAGGTCAAAGCTTTCACCTACAGCTTCGCACACACACATGGGTATTCAGGAG
AGATAACTAAGCTTGATTAGTGACAACCTATACTTATGATTAACAAAGATATATATGGATGATCATCCTTGTGA
CACCACGATTGGATTGTTCAACCTTGGTCCTATTCCGTCTAGCAGGGATGCCTCATTGTTAAATTAAACCTTAAA
GTAAACCGAGCAAACACTTTACCGTTCTAGTATGTTAATGTTATCCCTAGAGAATCACTTTCAATTGTTGTACAC
CTCTTTACTATCACGGGCTTCGCGTGTAGCCTTCCACTCTCTTCTTCTCGTGCCTTCACCTTGATTGATCTTGGAG
TCTTGGGTAACTGACAATTCCTAGGATCAAGTGTACACAAGAGTAAATATATGAAAACATAGAGGGTACACCA
ATAATCCCATCTTGAAGTCCACTGTGTTGCAAGGCTACACCTCAGCCTTAGTCTGAGGGGACTACTCATACATC
GCGATTAGATAAAAAATGGTAAGCAAATGTAAGAGCAAACTACTTTAGGATGAATCCATGGTAGTCTTACAAT
ATTGCAATCGTGAAGGAATGGAGAGTACAAGAGTATATGGTAATGAAAATAACTATAAACATGGCTATGGAT
ATGACTATTGTGGCAGAGCTTCAGCATGTTGGATGTGATGTGAATCTCTATCCAGTTGATGCCCCCTCTCCTTTA
CATAGGGCCAACGATGATACTACGATTTTGAGGAATCTCATATGCGGATTTGACCTGGGGCCCTTCACCAAGTC
ATCAGGGATGACATCACAGAGGCGGTGGCGTACGGTACGAGCATGCGCATGTACCACGCCACGTTATCTTCTC
TGCTAATTTTGTTGACGTTCAATTGACTTGTGGCTTCTCCCTCCCCATAACACTTCTCATATTTGCTAAGATTTTA
TCGTTAATACGTCCATTTGTGCAAGGAAATAACCAATGGAATTTGCACATCTTTGCACTTAGTGATTAGTGCCAT
GGTAAGGAATAGTGAAAGGATATGGATGTCGCCTAGAGGGGGGTGAATAGGCGATTAAAACTTTTCCTAGAG
TGGCTCAACAAAGGCAGAATAAAACTAGCGTTTTACTTTGTCAAGCATAACCTAGAACAACTAGGGTTCACCTA
TGTGCACCAACAACTTATGCTAAGCAATACAAGCAACAAAGTGATAGCAAGATATAGATTACTTAAGAACGAA
GGCTATTACAATGTAAAGTGCATAAGTAAAAGCTCGGGTATAGAGATAACCGAGGCACGCGGGGACGACGAT
A

>chr4C:2303111-2305111

ACCATAGCAAATGCAGTTTATGGTGTATGGGCCAAATACCGCATGGAAGTTACAGGTTTGGCCACTAAGAGTT
TGTCGTATAGTTGGCTCCAACACTGAAATGCGGGGTATACTGGATGGGTTTGAATGATCTGCTCATTTTTCTCT
CTATGACAGGTCGACCACCACTGGAGGAAATTTTTGCAGCTCGGAGACAAGCGGTTAGTTCACTGTATCTCCTA
TTCCTGTAACTTCCTGTAGTGACCATGAATAACTTGATCTTACAGCTTACTGACTGCCTTTTCCTTTGTCAGGGT
GAATGTAAAGATGATAAATCATGCAAGATGTGAGCAGCAAATGGCCCGTCTTCTCTACTCTGCATATTTTTTAC
TTCGGCATATGTTTAGTTAGGGAGACATACATACATAGCTAGTCTTACATCAGAATGACTCCGAGATTGATAAT
ACGGTGTTCTCTGTTTGAGGGTTGTAAATAATTGCAGTCTGTAGACTGATTCTATGGGAAAAGACATGCAACTT
CACTAAGTTGATTCCAGGATGTTGTCTTCAGTTCTGCCTTCCATGGTAGTCTTATCCATGTTCTGCCTTTCATCGT
AACCTTGAGTTTGGGTGGGAGGACGGCTTGACTCGGCGAGTGTGCCATCCGTTGTATTGTCGTTTGGGCCCTT
GGTGTGCTGGTGTTTTTCTGGTTTTGGCACTGGTTGTGTCAGTTTTCGCCCAATTTTTCATTAATTAACTAGGCA
TCATATTGTTAATTAATGGATAAGACAAAGCTTTTGCTTTCGTTTAAAAAAAATATGACGGGATGGTAGCATCA
ATGATGAGGATGATGCTATATGGTGCTATCCATGGCGAGGAGTACTTCAGAGCTTGGAATAGTCATAGTCTAG
TGATGTGTGATACTTTCAAAGTTGATTTGTCTGTTTCATGTAAAAATGTTCAGTAGTTTAAACTATTGAGTACTC
ATTTGAATGATGAAATATGCACTAGCGATGGTACGTACATTCTGGTTTGGAACATGTTATCCTAGTATCTTTTTC
ATAATTCTTGAGTTATATCTATATTATGTTTCAATTAAACAAATACCGAGTTAACGGGTGAATAGAATGTTAAAC
CACCGAACCACCATGATTCATATTCTGGACATTGACAAACAGCGTGATGTGCATACAAATTTCTCGGATTGATA
CACGACGAACAGGCAATTGCAACTTTAGGGTATACTAATAACTGATGGTTTGTACACGCCCTTTGCTATGATTA
CTCCGACGGAGACTCTCGGCAAGTCCTCAAGGCAACGGCCAGGGAGAATACCAGTGACCAACACATGCCTGA
TAGTGCACCGTCGGCCTTATACTGTCAGCTAACCTATTCACCGACTGTACCGTCAACCAATATAGTAATGGCCG
ACCGAGCATCCCGGCGGTGATTTCCGATGGTGACCCTTCGGTTATACACTTATCCAATGGCGAACTCAAATGCC
TCATGGTATTTCAACCCTCAGTAAGTGGGATTCACAACCGCCGTCGCCTCCTGTTGTTTCCCGCCGGATGCGCA
GGTCCAGAGTGGACTTCGAGCCCTCCTGGTGCTACTTTTCAACTTTTTTCTGCTTGGCAGCTGATGTAAGCAATA
TGTTCTGCTCTGTATCTACAACGAATATCCGTTATTGTTCCTGCCTCCTTTTTTTACCAGTTGCTCTGTTTTGGAAC
TGCTGGTTTTTGGTCTACACAACTGATTTGGGGCTGCAGCACCTGATTTTGGCCAGATCCCCTGTTTTTGGTTTG
TGAATTTCTCTTTGTTCTTGCTAAGATTTCCAAAGATGGTTCTATCTTCTCTAGTGACATCAAGCTTGATGGCTCA

AATTATAGAGAGTGGGCATTCTGAGTTAGAACTATTGTGAGAGTTGCTGGTTATGATGACCATTTGACTGACG
ATCCTCCAATTGTAAAGGAGGATGAGGCCACAAGGAAGACTTGGAGAAAATAAGATGCTAAGGTCATG

>chr4C:57331556-57332556

GACGGTATATAGGTATTATTGCTGTCACTACTACTAGAACAAAGAGCATGCAAGGTTGTAAACCTGTCATGTTT
AAGAAGCACACGTACATTAGCTAATAATAATACTGCAATACATAAGAGCCAGCCACAGAAGAAATAATTCCCA
GCGACAAGTCGGCCCATAGATAATAGTACTGGAAGTTGAGAGCGACACACACTTCGGCTCGTCAAAAAATGTA
TTCCATCTCTTCTCCAAGATATTGCATCACCAGTTCAAGACTACTACTTCTGGATCGAGCCCCCACTGGCCATCA
GCTACCTGCACGAATGACAAAACATAGTCACATCACAAACTCTACACCATCTGCTCGTGCTTAAGCATGAATAA
CCGAACAAAGTAACAGCACAACTAAACCACATGAACCTGCTCACTATGCAACAAAAGAACGGCCATATTTACA
ATGAATCAAAGGGAAAGCACAGAACAAGACCGTATGACAATCATGCCTAACAGGCGTCTCAATAGATAGTCTT
CTCGTAGTTCCAAAATACAGAGCAATCTAGACACTCAGAAAGATCACCTATCACAACTTCAAGTACATGAGCAT
ATACATATAGTACTCCCTCCGTTTCAAAATATAAGATGTTTTAGGTTTGTGAAGAAAATGTATCTAAACTTGTTT
TAGTGTCTAGATTCACTCTAAACCTTAGTGAACCTATATACTAAAAGTAGTCTAGATACATTTGCTTCACAAATC
TAAAACATCTTATATTTTGAAACGGAGGGAGTACTATATAATTCAGCACTCCTTGGGCATGGACCTTACAGCAG
CTTCACTGTTTCTGCTCAAGTTCTGAGGTGGCATCACTGAATGTACACCTCAAGAGCAAGGAGGATCAAGAAC
AGTGAAATTACTCAGCTAACATCTAAGATAACTATACTAAATAAGCAAAAAATCAGCTACTTTAGTACTGCGTC
ATGTATACAAGGACTTCTCTATTTCAGTAAAACTTGAAAT

>chr4C:71757840-71759840

TATGGTAATACACAAGTGTCAAGTGTTGTGCATGATCCTTTCAACGATACAATACAACCAGTTACGGCAGGCAA
CATATATTTACATAAAGCTCGTATGAGCAATCAGTTGAGATTATTTTCTTATACATAAAGCTCCAGATTTTTTGG
CACCATCAACCTACGATGGCTGATCGTGCAGCTTATGTCAGAATTGCCAGTTTGCGTACTATTCAAGTTAAGAA
CGTATATACAGCACTATACAAGATCTTCGAATTAAACCAAGGGGCGGGCAGACGCCTCCTCTCACCTCAAACA
GAGGACGCCTTTCACTTCAAAGGCTTCCCTGGGAGGAAGAAGTCCTCCTTCAGCACATGCTCCCATTCCGACGG
CGAGTGGCTTAACCGCTCGTCATGCAAACGAGCAGTTGCAGGTTCGGCTGGTGCTGAGGGAACATGGCACTCT
TCAAGTAACGAACCGACGCATTGGCTTGTGGTTTTGCTGTCCGCTTCATCAGATAGAGCTCTGAACTCAGCATC
ATCAGACGAATCAGTCTTCTCAATTAGCTCATCATGCGCTCCACGACCAAATTGTTTTCTTTCAAACTGACCCAA
CCAATCATCTGAATCGAATACCTCTTCTCCCTCCACGCTGCTACTGTTATCTATACGTCTTTTCAGTACTTGCAAC
CTGGCCATAACACTGTCTTCAAGCTGACCAAATTGGCTAGACTCATTTTTGCTTGCTGTTGACAAGCTTATATCA
TCACTGCAGCACATCAAATCTTTAAGCGTAGACATGAAAGCACCATGGGTTTTGCTATCACTAGATCCTAGCTG
CTCTATGTGGATGTCCTCGTCCATCGAGCTTCTATTGTTGTTACAGCTCAGGAAATCATCAAGTGCGGCAGTAG
TAGCACCATCTGCCTCGTCACCTTCACTGCTACTTGCTTCCGGACGCTCTTTGATGATTTCCTCCACTAAAGAGCT
TATCCTAGCGTCAACAGCATTATCATAAACAGTGTCATCAACAACATCTGTCCTGTTACACTTGCTTGTTTTGGT
CTGCTCACGGAAGCGATTTATAGACTCATCACGGAGCTTTAGAACTTGCAGTCGAGCAAAGACATCAGCATCA
CCTCCCTGAGGTAGTAATATCGCGTTATGGATATGGCTTTCTTTCAGAGGGTTTTGTTGTTTGCTAGAACCATCC
CTCTCTCCAGTACATAATGAGTTCTCAGACTTGTGCAAGTTAGAGGCAGACTCATCACGGAGCTTTAGAACTTG
CAGTCGAGCAAAGACATCAGCATCACCTCCCTGAGGAAGTAATATCGTGTTATGGATAATATGGCTTTCTTTCA
GAGGGTTTTGTTGTTTGCTAGAATCATCCCTCTCTCCAGTACATACTGAGTTCTCAGGTTTGTGCAAGTTAGAGA
CTTTGGCTTTTCCTAATGAATAGGTAGGTATTGTAGCTGCAAGATTATTTAACACACAGGAGTAAGGGAATGCT
CAGAACTACCCGGATAAATTAAGGTGTGGTCTCTTGCTACACACACAAAAAAGAACACAAGTACTGACCTGTTT
GCTGACTGTGGTTTTTCGTTGCAAGTTTCATGCGGGCAAGTTGCAGTTCATATTTGAGTTTACACATGGAAGCT
TCAGCTTCGATCCATAGATTCTTGTACACCATCGTTTGTGCATCTTCAGTACCACCGTCCACGTTCTCCTCTGGAA
GCTTAGCAAGATCCTGCCAACAAGATAGCAATAACATAAGTTAAATGCTCGAGAAGATAGCAGATACCAACAT
ATTCCTAAGGAACAAATCACTACCTGCAAAATAACTGTTTTGAAATCTGCGTCAATGTTCTCCCAATCAAGGCCA
GCAAACTTCTCAGGCTGATGATTCTTTCTTTTTTCTAAGTTCATTTGGCAGCAGTTGCTAGTAACAGCATCAGAT
TTTAATGAAGCCTTGCACGAGAAAGTAGACAAACCAAAATGGGTAAACTGAGAAATTTGACTGTCA

>chr4C:76953674-76955174

CAATTGCGTCATCGAACCGGGTCTAGCTAGAACAAATGAATAATATGGCGTCCAACTCCAGTAGAAATCTCCTC
CTCTTCGCCACCGTTCTGCTCTATGTCTTCGCCCCTGCCGCCGCCACACGCAGTTACTGCTTCCCAGGGATGGTG
GGTAGCCCGAGCGATCTGATCCCAAGCTGCCGTGACTACGTGGAACAAGCGACCTGCGGCTTCGATACCGAA
GGGCCGCCATTCATGGCCAGGGATGACTGCTGCAAGCAGCTTGCAGCAATTCCGCAGCACTGCCGGTGCGAG
GCGTTGCGCTGCTTCATAGGGCGGAGGTCCCGTCCTGATCAGATCGGCGGCCTCATCGACATGGCTGGATGCC
CCAGGGAGCCGCAGAGGGATTTCGCCAGAATACTCGTCACGCCGGGGCAATGCAACTTGGCCACCATTCACAA
CGCTCCGTACTGTCTCGCTATGGATGAGTCTCAGTGGCACTAGAGATAGATCTATGGCCCATGAATAAGCATAT
ATATTGCATCCGTGGATATGCGTGACATTCATATATGTCTCAGCATATGTTTGAAAAGCTATCTCTGCTATGGAC
GAGAATAAAGAGAAGCATTTTGTGGTTCTTAAATTTCAACTTGCTTTTTATGCATATGCTTCGGATTTTGATGTG
ATCTTATTATTAGTCACTGCTTGTGACCCTTAGAACCCTTCCTCGATCAACTGGTTTGGAGTTCTAGATCTGATG
ATATTAAATTTTGGGGATATTTTTTCAATCTTTTTTGCTTACTTGGCATCAGCCAGTGTGCCACCACTACTAGGA
AAAACCTTATATGTGGCGCATGGGTCGTGCGCCACCGTCGTCACGCTACTGATATTTTTTTATCTGTGGCGCAC
GGGCCACAGAAAAGCCCACTTATCAGTGGCGCATGTGGGTAAGGTGCGTCACGGATAATTTTTTCAAAATTCG
AAAACAAAAGGTAGGATATGAAAAGATATATGTGGTGCATGTTGGTATGGTGCGCCACGGATAAATTTTTCAA
AATTCAAAAAAATGGGATCTGGATCTCGTCGTTGTCGTCGTCGCCTCACCACGGCCACCACCTACCACCGCATT
GAGGTGCGGGACACCGTGGCCCCGCTCGGGGCCGAGGAAGAGGATGCCGACGAGCTCGAGGATGGCGCCGT
CGTGTTGGTAAGGGAGTAGCTCGAGGATGGCACCGGCGTTGGTCGGGGAGGAGCTCGAGGACGACCCCGTC
GGGGTCGGCGGGGAGCTCGAGCAGGACACCGTCGGGGGTCTGCTCAGGGAGGAGCTCTAGGACAACACGAA
GAAGGAAGGAGAAGTAGAGGTGGAGAAGGAGAAGTAGAGGTGAAGGAGAAGAAGAGAAGAAGGAGAAG
GGGAGAAGAAGGGGAGGAGAAGGGAAGGAGAAGGGAAGGAGAACAAGAGGAGAAGGAGAAGAAGGAGA
AGGGGATGGGAGGGGAGGCTCGGATTTAAAGTCCAGCCACGCTT

>chr4C:76967920-76968920

ACAAAGTGCACCAACAAACCAGATCTAGCTAGAATAACATGACGTCCAAGTCCAATTGCAGTCTCTTCCTCTTG
GCCGCCGTCCTCCTCTCCATTTTCGCCGCTCCCGCTGCCTCTGCGGCGGACAATCCCTGCTTCCCAAAAACGGCT
ATTCTGAGTAGTATGCTCCAAAGGTGCCGTGACTATGTGGAACAACAAACCTGCGGCGTCGAACCTTCCGGTC
CGTTTATAATTTCTGTAAGGGAACCGTACATGGTGAAGGAGCGTTGCTGCTGGGAGATTGCCAACATTCCACA
GAAGTGTCGCTGCGAGGCGTTGCGATACCTTATGAGGAAGACGCCTGCTAGTCTTCCCTATGAAGTCAGCGTC
AGGGGCATGCCCGGATGCCCAAGGGAGGCGCAGATGAACTTTGTCCGAATACTCGTCACGCCGGGGCAGTGC
AACTTGGCGACCATTCACAACGTTCGGTACTGCCCAGCTTTGGACAAGTTTCAGTACTAGAGATTACAAATCTG
TCTCTCGTGAATAAGCAAGCTTGTTGCATCCACTGACGTGTGGCATGCTTATCTACATATGTGCGCTCCAGGTTC
ATGTGTTGTACTATCTGGTATGATTAGAATAAAGAGAATCATTTTGTGGTTCTTATACTGCAACCCGATCTTTTA
TACTCCCAATGGTTCGGAAATAAGTGTCGCATATTTGTCTACCGTATCTAGACAAATCCAATATACTTATTTCCG
GATGGGGAAATGATGTTACAGCCTTATTTGTATCTATGTTCAAAATATACTTTCATGGAATCCAGAAAATTATTT
TACGGGTTTAACAAATGTTTTCACATGATTCAAAATATGTTCAGGGGTTCCAAAAAAGTTCGCCTCTAAAGTGG
AGACGGTTCTAAAAAAATGTTTAGAAGAATCCAAAATTGTTAATATATTTATATAAACATTTACATGGTTCAGA
ACTTTGTTCACAGTGGTTGAAAACTGTTCTTGATTTTT

>chr4C:76969850-76970850

ATGGCGTCCAACTCCATCTGCCGTTTTCTCCTGTTGGCCGCCCTCCTGGTTTCTGTCTTTGCCTCTGCCGCTGCCA
CCAGTGGCGGTGAATACTGCTACCCATCGATGGGTCTTCCGAGCCATCCGCTCGATGGCTGCCGTGAGTATGT
GGCGCAACAAACTTGCGGCGCCCGAATACTCGGGGCACCATCCGCCCCCATCGAAACGTTGATGTACCGGTGT
TGCCTGGAGTTTTCGCAGATTCAGCAGCACTGCCGGTGCCAGGCGCTGCGCTACTTGATGGGGTCTCGTCCTG
AAAGGAGTAGCCTCATGAACCTGCCAGGATGTCCCATTGAGCCGCAGAGGGACTTTGCCAGAATACTCCCCAC
GCCTAGGCAGTGCAACTTGGTGACAGATTACAACACAAGATACTGCTTGGAAATGGACAAGTTCATGTAGTAC
TTGAGATCTCTCATGAATAATCATGATGCATCCATGGATGTGTGTGACAAGCATAAGTGCATATTGTGAGCTCG

CTCACAATATGCTATGATTTAGAATAAAGAGAATCATTTTATGGTTCTTAAATTCCAACTCTTACCATTTATTCAT
TTGTTTGGTGGTTGTATAGCCTTATTGTCGATTGGTTGGTGTTGCTTATGACGTGTGCCACATGCCTTATTGTTA
GGTTAATATGTCTGGACATTATACATCAAAAAATCACTCTCAAGCTTGTCCTACACGGAGCCATTCTTCAAAAG
AATAATTAAAAGATTTGAAACTTTGATTTCAAAGTTTTAAAAAAATATGAAAAACATTTGGATGTATCCAACTAT
GCATTCGGCCAGTGTGTAAATTAACAGATCGAAATATATTGTATGTTAAGCTCAGAAAAATGATGAAATCTTAC
AAAACAACTAGTGCACCGTTTACAACTGTTTATCCAAGACAAAATCTCGATTTTCCATTTTTTCTGAGCTCGTAA
TACAATGTATCCAGTGGACGCTTCGATAGCGTGGTG

>chr4C:76976251-76977251

ATGGCGTCCAACTCCAAGTGCCGTGTTCTCCTCATGGCCGCCCTCCTGGTCTCTGTCTTTGCCGCTGCCGGCGCC
ACCGGTGATTACTGCTACCCATCGATGGGTCTTCCGAGCCGTCCGCTCGATGGCTGCCGTGAGTACGTGGCAC
AACAAACTTGCGGCACCCGTATCCTCGGGGCGCCGTCCGCCCCCATCGAAAAGTTGATGTACCAGTGTTGCCT
GGAGTTTTCACAGATTCGGCAGCACTGCCGGTGCCAGGCGCTGCGCTATTTGATGGGGTCTGATCCAGAAACG
AGTGGCCTCATGAAGCTGCCAGGATGCCCCATTGAGCCGCAGAGGGACTTTGCCAGAATACTCCCCACACCTA
GGCAGTGCAACTTGATTACAGAATACAACACAAGATACTGCTTGGAAATGGACAAGTTCTCGTAGTACTAGAG
ATCGCTCATGAATAAGCATGATGCATCCATGGATGTGTGTGACAAGCAGAAGTGCATATGTGAGCTTCGCTCA
CAATATGCTATGATTTAGAATAAAGAGAATCATTTTATGGTTCTTAAATTCCAACTCTTACCATTTATTCATTTGT
TTGGTGGTTGTATAGCCTTATTGTCGATTGGTTGGTGTTGCTTATGACGTGTGCCACGTGCCTTATTGTTAACTA
GATCATGTAGGCGCGCATTGCCGCGCCCGTCCACGAGGGCAATATTTTGTTAGGAATCGAAGAAAATATATTG
AGTGCAGGCTAGACAAGTGAAGTAAAACTCCATCCGTATTTTCTAGTTAGTTAGTTGAACCCTCGTCTTATTCA
GAGTAAAAGGAGAGGCCCTAGTAATTCTATGTGGCTGACATTCGTTTCAGGTAAATACATTGTTGCCGGAGTG
TGACATGAGGTATATACTTGGTTTACTGCATCAGGAGGGAGACTTCTGGCTAGTGTCCATCCTAACGATACATC
GGCCATCCATCGTTTGAGTTCCTCAGCTGGTGACCTCGTGTT

>chr4C:76987152-76989324

GCCACCAGGGCTGATTACTGCTACCCATTTATGGGTCTTCCGGGCCGTCCGCTCGAGGGCTGCCGTGAGTACG
TGGCACAACAGACCTGCGGCACCGGCATCATCGGAGCACCGCCCTTCCCCATGTCAGCGTTGAAGGCCCAGTG
TTGCCAAGAGCTCGCAAAAATCCAACAGCACTGCCGGTGCGAGGCGCTGCGCTACTTCATGGGGCCAAAGTCT
CATCCTGACGAGAGCATAATCATGGGCCTGCCCGGATGCCCCAGGGAGCCGCAGAGGGACTTCGCCAGAACG
CTCGTCACGCCGGCGGAGTGCAACGTGCCCACCATTCACAACACTCCCTTCTGCCTCGCTATGGACGAGTAAAT
TGCACGTAACATCAGACTTTGGTGTTAAAGTTACACAAAACATAAAAACAAAACATTTGTTGCAGCTAGCCCGG
TTTGTTGTCTAATCAGTTGCAGCTAGCTCAAATCGAGTATATAGTGCCTTAAGAATATATCTAACACACGTGGG
AACATATTTCTATATTGTATAAATTATGTAAATTAAAAAAATAGATATCATAGTGGTAGAAATATGAGAGCGTC
AATTTGACAGATGAGTTCCCGGAGATCTATTTTTAAAAAATAAAAAAAATCAATTTTTTAGTTTCGAAAAATTCA
GACATGATATCAGAACGTAGCTAATGATTTATTCTACCTTTTTGTGCAGATCTCGGAGCATGATAATGTAACTTT
TAAATCTTCTATAATTTATCCAATTTGTCTATTTGGATGAGCCTAAAATACAAGGTACTTCACATTAAAAATTTGC
ATGCTTGTAACATGCATATTTGACCACATCAAGGTTATTTTTCTGAATTTTTTTGTACATATTCGGTCGATTTTTT
TCTAGTGTACGCGTAGACGTAGACGCTGTAATAGTGGTATCAGGCCGAGTTCGATTGCAACATCAGATGCATT
GGATTGACCTTTGTTCGCTAGAGGATATAAATACAGAAATATTGGCCCACACGAGTGATATACAAGATAGAAA
TACGCTCATACATTAGATACGTTTTTAAGGCACAATAATCTCTATTTGAACTAAATTTGGGCGACCAATAGCCG
GGGTCAGCTGCAACAAGTCTTTTGTTTTTGTGTTTTGTGCAACTTTGACACCAAAGTCTGGTGTTATGCACAATT
TTCTCTCTATGGACGCGTTTTAGTTTGGTGGTACCCATCTATCGCCCATGAATAAGCATGATGCATCCCTGGATG
TGTGTGACCTGCACATATGCGTATGTGTGGTTCTATCTGTTGTGATTGAAAATAAAGAGAATCTCTGGTTCTTA
GATTTCAACTCAATTCAACTGATTTTTCTTTGTTTTAAAGCCATATTATTTTGTCCATTTGTATGGACATGATACA
TGCACATGTGGGGCCGGGGGGCTAATTGGAATAGCCAATTGAAACAGGAGAAAAAATATATTCTGCATCTTAT
TCAGGCTCTAGATACATTGTCTGAAAATTTGGGTCTTTCTAGTGCTCAATGGAATGAAAGATACAAACTAGAGC
AAGACTTCATGAATATTTATGAGATGGAGGAGCTTTATTGGTTACAAAGAGGGGGATGGTTGGAAAAGGGGA
TGCTCTATCTTTTCTTTGGAAACTGATCAGGGAGTCATTTGTGACAAACTTGAACTGCAAAACCACATCTATGCT

TTCAATAATGATTTATTTCAAAAGGATGGCAGGGGTAGGTGTAGACTGGCCCGCGTGATGAGTTTGATCTGGG
TCTCAATTACTGACTCTAGAAGACAATGTTTTGTTAGTTGGGCCCTTTTCCATAGAAGAAGTAGAAGATGCTTTT
AAATATATTTGGACTAACTTACACTACGGGAGAAGCTTAATACGCCGACGGTCGGGTCCCGTCGGCGTAGGCT
GGCCCGACGGCTGCCGTCGGCGTATGGCCGTCGGCGTACTGCCCCGTAGGCGTAGCCTCTTCTCCCGTCGGCG
TAGAAAAGCCGTCGGCGTATATAGCTACGCCGACGGCAAAAGCCGTCGGCGTAGACCATATGGGGCCCACCT
AGATGACTAACGGCCGTTTGGCTACGCCGACGGCTTTACCGTCGGCCTAGATTTTTTTGAATTTTTTTTCCAATT
TTTTTTTCAATTTTTTTTTCAAAATTTT

>chr4D:128967819-128969819

ACACAACAACGCGCAACAAAAGCGTTGGCTTAAATGCCTATTTCATTCCAAGGTGCGCTCCAGCACTTCTACTT
CTACCTGCCACAAGCCCGTATCTGTGACCAGCCGGCTGCACACACAGGTTCCGCAGGAGGCCAGGGCAAACAT
ACCCTTACCAGAAACACACCGTTTTAACATGTTACAAGGGAGGGTGGTGAATCTTCGCAGATATTCTTGCTTTT
AGCATTCTTTCTTCCATAGTCTATCCAAACCCCTTCTCACGCAAGCAGTACTCACCACCCCAATAGATAGTATGG
ACTTGACCTCTCTTCATTCAGGCTTTGCATAAAGCGCTTTCCGTACCTGCTTTCGACCGTGTTGTTCTCTTAGGA
GTCCACGTTTCTTAGTATGGTCCGGTTTTTGACTTGTGTTTTGTGGTTTATCTTATCATGTTATATTTTTCATGACC
AGGACCATGTCCTAGAAGAATTCCTGGGACTCCAGGTGGGGAGTCTGTGCTCGGGGATGTACTATTTTACTGC
AAAATCATGCTCATTAGCCCGCCGGTATAGAAGTGTTGAAGCTTCTACCCTGTTGATGGCAAACATGGTAAGG
GTCTTGGTTAAATGATGCGTCATTAAAGGAGTTGAGGTTATTTAATTTAGAGACTGGATTGAAGTAAATTCAAT
ATTACTATTCCTCACCATCCTGGATGAACGACGAATCTGCCTGATTGTATTGCCCAATTCACCTTGTCAGTCCCG
AGCGAGTTGGCGACAACATGAGTCACCCGATCATCAATCTGGTTAGTGCACACAGCACCAAACTGCTCTGCAC
TTTGCCATAAAGGATGCATGTGGGGGTTAGCCTCTCCAACAGGGAAAATCCGGCTAAAGACAATACGGCAGCC
AGCGAGGATCCTCCGCTGCTCAGATGCTAGTATGCTCCGCACATCAGCATCATTGAGGTTGGGATGAGAAAAG
AAGTTTTGGTGAATGTGTCCAATAACCTATTTCAAGATGCGGAAGTCTGTCAGTATCAAGTTGTATTTCAAAGT
TTAGACAAGGAAATGACCCATCATACCGCCAGCGAAGAAGCGAGAGTGCCATCCTCTGGCCTTTCATCTCGAT
CAATTTCAAGAAGTGATGGTCCAGGAAGGCCAAACTGCCGCCTGCTGCAGGGAAAATAGGTGTATCTACAGA
AAATCGTTCAGAGAAGAGCTTAGTAAGATGCACTCGCTGTTCCTTCACACTCGGGTAAACGTTTGGTAGAGAA
ACATACCTCTCTACAACAATCATATTGTTTTTGTTGTGTGGCCATACTCTCACAGAGTCATCGATGATCACTACT
GCAGATTCCATTCCCAATACCCCATCAAGATCTTTACTTTTCGGTACACGGTCATCACCGTCAAATGTATCGCCA
TCACCACCTCTTGAGTTGCCGTCACCACCTCTTGAGATGACTCTCCCCGCAAACAGGGCCCCAGTAGGGTCAAG
AACCTTGGCCATCTCAGTGGCATACAGCTTGTTCCCCATTGTGTACAGATGTAACTCGTAAAGCTTAGTCGCCT
ACAGCATAAAGCATCAAATAGAACAATATTCAACACGGAGTAGCAAGGCATCCATGAGCAGTTATGACAAATC
TAAGACAGCAAACCTTCTCAAGAAAGTTCCATATTCCTGGTCTCAGTTTGGTCCACATTTGCATATGATGGAAA
CGGAACAAATGGCGCTCTGGCCTCTCCCGGTCTTGTTCTTCTTTCTTCCGCAGAATCTCTTCGTGAATAGGATCC
ACTTCTATAAACTGAATTGGAAGTGTACATACTACCTTAATGAAAATGCCAATTAGTGAAAGAGGTCAAGCCAT
AGGCTACAGTATAATACCTTCGCGGAATTGAGGAGAGTGTGATCCAAATCAAGAACTAAACAAAGTTTCCTTG
CTGAAAACATTTTATGCTGTTCCATGATCCGTCTTGCCCTTTCCTTCTGAATGAGAGCCTTCTGCTGGTCGTCAT
AACC

>chr4D:222129679-222131679

CCACCAGCAAAACAAAATTGAAAACATGAAGGCCTTGTTACTCCTAGCTCTCATTGCTTTGGTAGCGAGCACTG
CATTTGCGCAGTATCTGGAAGTCCGCAATGAGGATGCTGAGGTCCATGGTGAGGTAGAATGCCAGCAGGAAC
AGGTTAGTCTAGGTGCATGCCAAGATTATGTGAAGGATCGGTGCAGGTCGATGAAGTTTCCCATCACCCAGCC
TTGGAAATGGAGAAAGCCTTGGAAATGGACCGAGCCCTGGAAATGGGGGATGAGCTCTTGCGATGATGTTCA
GAAGCGATGCTGCGAAGAGTTAGCAAAGGTGTCGATGCAGTGCCGTTGCATGGCTATTAAGCGAACTATCCA
AGGCGACATCATGTTGCGCTTTCAGCCAGGTCGGATGTCCAAAGTGATGGAGAAGGCTAAGAGTCTGCCTGTC
CTGTGCAACATGCCCCCTTCTGATTGCAAGTCCATGGCTAATGGATATTACTGATGATATGGTTTCCATCAGTG
ACTAATAAACTGTCACATACCATGGTATGTGACCATTGTCATGGAATAATGTATGGGCAGATTTTCCTCTGAGT
AGTGGTTTCTTTGCAAACATCTTGGCATGTCCATCAATGCAATGGTGTTAGCATATGAAATTACAATAGGCGTC

GTTATTTGGTGCTCTTTAGTTTGTGTGTGCATGTCCATCCTCTATTTAGCAAAATTATAATATATGAATTGTTCAC
CGAGTCGTACAAAGCAAAGTGAGGATGACCAAATGGTTCTCTTCAAGAAAGATTCATTGAGTATTTGACAAGC
TGTCTCTTTCTCCAGTTTAACAAACAACTAAAAGTAAAGTCTAGGATATCCTGCAACATGCTAAAACGACCGAC
GGATTTATACACACCAACCACTTCATGTTACATTTATTTTTGAAAAAGACTGAAATCTTGAACAAGCTTTAGGAT
GAACAACATGATTCTAAATAAGGGAAAAAGTTCAGCATCTTGATGTGTTACTTTTTTTAAGTGAACCTTTGGTA
CAACATTACATATCTATCTTGTACTACTACCTTGTAAGACAAATCGGTCTGGATCCATCTTCAACAAGGTGGGCA
ACAATACACATTATGTTTCACTGAAGCGAAAAGTCTTAAACCTTACCATAACATTACATATCTATCTTCTTAAAG
GGGCAAATTGAGTACAAGAAACACCATAACTTTCACATCCGTGTGATGACGACGACTATAATGTAGCTAGCAA
TAGGCCACCTTCCATTGGTGACTTGAAAACGGCCACATATCACCAGATGTGGCCTATAGAAGTGGAATTATATA
TGAAAGAAGTTCTGTGGAAAGTGCCCTGGGATCCCGAGCTCCAATACTCCCTTTGTTTTAAAAAAATTATATAA
AAAAGTTAAAAATAACTAAAAATAAATTCTGGATATAGTCCATGTTGTATCCTACAAACATGCAAAATCGCAAC
CCGAAATTCTTTATATTTTTAGCATAGTCCATGTTGTTTCCTTTGAATATTTATTTAATGTGTTTTTTTGTGGTAGT
TTTTGTGTGTATGTGTGTTACTTAGCATGCACCACTATCTTGTATGACACAATCTCTAGGAAAGTCACAACTAGG
AACATTTGGATCCATCTTAAACAACCTGCACAACTCTACAAGTATAGTTACACAAAATAATAAAATTTGCATTAT
ATAATATTATATTCCTTGGTACAATACAAAACCATAAGCAAAAGAGGATATGCCACATAGACCATTGTTAATGG
TATGTGTGGTACAAGAAACAAAATCTTCTATCTTAGTGATACATGATTTGTTAATACATGACCAAAAAACATGG
CTAGAAGACGATGACGTCTAAATGTATTACTTATCATGTACCACTACATTGTAAGACATAATTTCTAATGATTTG
GTCCATAACTTTTTTGAACAACTTGCACAACACTATACAACTACTTCTCACTGGTATATCTTTACAAAGAAGTAG

>chr4D:285709921-285710921

TAATTAGTCACTTCAGGAGCATTTTATTCATGCGTATGATGGTCGTACAATATTGGAATTTGGTAATATCATGAT
CAGATACCAGACGTATGCATCGAAAAAATTCATTACACACCTGAAGTGCTTATTCTTATCTCACTAATTAATCAC
TTCACGATTATTTTATTCATGCGTACAATTGAAGTGCTTATTCTTATCTCACCCAACTGTATATATGTATGCTGTA
TGCTGGTACGGTACCATAGTAGTACCATTCTGCTCGCTCACGGGCAGAAGATGACCTGATAGTTGGTGCCGCC
GGGGCAAGTGTAGATGCTGGTGGCGTCGTCCTTGGGGTAGCTGTAGGCGTCCGGGCACAGGCCCTTGAAGAA
CCTGGAGTAGTCGGTGGGCCCGCAGTTGTTCGCCGCCGAGCCGGTACAGCAGTACTGGTCCTCCTTGAACACC
GTGCACGCGTTGTTGCACCCTCCGGTGGCCTTGAGCTCGTTCGGGCACTGCGCCGTGATGTCGGCGTCGCAGC
GCGGCCCGCCCTTGGGGCACCCGGTGGTGCCGTCGGCGGGCAGGAAGTCCATGGGCACGTTGAAGCCGTCG
ATGACGGAGATGTCGATGAAGTCCTGGCCTTCGTACTGGTTCAGCCCGAACTCGGCCAGCGTGTTGGGCGCCT
GCCCGTACTGCGTGCACTGCAGCTTGCCGCCGCAGTCGCCCGTCTCGCACTTCCCGTTGCCGCTGCCGTCGAAG
TTGCAGCCCGTGCGCGCCCACACGCGCCCGCTGGTCGTGCCGGCCGCCACCTCGACCTTCCACTGTTGCCCCGG
GTCGAGCTGCTGGCCACCGCCGGGCACGGCCGCACCCCACACGGTGAACTGGCACTTGTTGGTGACGGTGAA
CGTGGCCGCGTGGGCGGCGGCGACGAGGAGAAACAGAGGCAGGACGCGCAGGGCGGAGGAGACGGCTGC
CGACGCCATTGTGTGCAACTGTTGTTCTGATCTTGAGCAAGTACCTACTT

>chr4D:29266670-29268010

ACCATGGTGGTGACGGGTGACGGAGAATTAGGGTTCGATTCCGGAGAGGGAGCCTGAGAAACGGCTACCAC
ATCCAAGGAAGGCAGCAGGCGCGCAAATTACCCAATCCTGACACGGGGAGGTAGTGACAATAAATAACAATA
CCGGGCGCATTAGTGTCTGGTAATTGGAATGAGTACAATCTAAATCCCTTAACGAGGATCCATTGGAGGGCAA
GTCTGGTGCCAGCAGCCGCGGTAATTCCAGCTCCAATAGCGTATATTTAAGTTGTTGCAGTTAAAAAGCTCGTA
GTTGGACTTTGGGCCGGGTCGGCCGGTCCGCCTCACGGCGAGCACCGACCTACTCGACCCTTCAGCCGGCGAT
GCGCTCCTAGCCTTAATTGGCCGGGTCGTGTGTCCGGCATCGTTACTTTGAAGAAATTAGAGTGCTCAAAGCA
AGCCATCGCTCTGGATACATTAGCATGGGATAACATCATAGGATTCCGGTCCTATTGTGTTGGCCTTCGGGATC
GGAGTAATGATTAATAGGGACAGTCGGGGGCATTCGTATTTCATAGTCAGAGGTGAAATTCTTGGATTTATGA
AAGACGAACAACTGCGAAAGCATTTGCCAAGGATGTTTTCATTAATCAAGAACGAAAGTTGGGGGCTCGAAG
ACGATCAGATACCGTCCTAGTCTCAACCATAAACGATGCCGACCAGGGATCGGCGGATGTTGCTTATAGGACT
CCGCCGGCACCTTATGAGAAATCAAAGTCTTTGGGTTCCGGGGGGAGTATGGTCGCAAGGCTGAAACTTAAA
GGAATTGACGGAAGGGCACCACCAGGCGTGGAGCCTGCGGCTTAATTTGACTCAACACGGGGAAACTTACCA

GGTCCAGACATAGCAAGGATTGACAGACTGAGAGCTCTTTCTTGATTCTATGGGTGGTGGTGCATGGCCGTTC
TTAGTTGGTGGAGCGATTTGTCTGGTTAATTCCGTTAACGAACGAGACCTCAGCCTGCTAACTAGCTATGCGGA
GCCATCCCTCCGCAGCTAGCTTCTTAGAGGGACTATCGCCGTTTAGGCGACGGAAGTTTGAGGCAATAACAGG
TCTGTGATGCCCTTAGATGTTCTGGGCCGCACGCGCGCTACACTGATGTATTCAACGAGTATATAGCCTTGGCC
GACAGGCCCGGGTAATCTTGGGAAATTTCATCGTGATGGGGATAGATCATTGCAATTGTTGGTCTTCAACGAG
GAATGCCTAGTAAGCGCGAGTCATCAGCTCGCGTTGACTACGTCCCTGCCCTTTGTACACACCGTCCGTCGCTC
CTACCGATTGAATGGTCCGGTGAAGTG

>chr4D:305957626-305958626

ACACCCCATCCTACTTGAAATTACTAACAGTACACAGAATGGCGTCCAACCAGCTCATCCTCTCCATCGCCGTCC
TGCTCTCCGTCCTGGCCGCCGCCTCCGCCAGCGTCGGGGATCAGTGCGTTCCCGGGCGGGCGATCCCGCACAA
CCCGCTGCAAGGCTGCCACTCATACGTGGTCAGCCGAATCTGCGGCGCAGGGCCATACCTCGCCACCGAGGTG
ATGAAGCAGCAGTGCTGCCAGGAGCTGTCCGCCATCCCGGCCTACTGCCGGTGCGAGGCGCTGCGCATCCTCA
TGGATGGGGTGGTGACGGCGGAGGGCGTGCTCGAGGGCGGCCTCCTCCAGGACTTGCCCCGGTGTCCCAGG
CAGACGCAGAGGAACTTCGCCGCCAACCTCGTGGCCCCTGGGGAGTGCAGCCTGATGACCATCCATGGCAGC
CCGTACTGCCTCACCCTGGTCGGCCGTCAAGTGGCCGTGTAACTTCTGTACTGTCTAAACTGCTCGTGATCGTG
AATAAGCGTGTTGCATCGACTGATGGATGCATGTGTATGCGCATGCACCTATATGCGCATGTTTGAACGATAAT
AAAGAGCATCCCTAGTTTCTACATTTCAATATTGTGCACGGTATGTTGTTTGGTGGGAAAAATAGTTTCAGACG
TTTAAAAAATTCCAAGGCAGTATAATTACCTCGAGATGACCATCTGAAATAAAAACGCTCCCTCCATTTTTGATT
ACCCTAGGTTATGTGTTTAGTTAAAGTCAAACTTTGTAAAGTTTTACCAAGCATTAAAAAATATATTAGTAACAT
CTATAATACTAAATATATATAATATAGAAATATACTTTATAATAATTATTATGATATTGATTTTATATTACAGATG
TTGATACTTTTTATCTATTTGATCAAACTATACTCCCTCTGTCCCAAATTACTCTGCGTTTTAGGTTTAGTCAAAG
TCAAACTTCATAAACTTTGACTACAAATATAAGAAAAAT

>chr4D:347409741-347412166

ATGGCAACTACTAGGTTTCCATCATTGTTGTTTTACTCCTGTATTTTTCTCTTGTGCAATGGGTCAATGGCTCAGC
TATTCGGGCAGAGCTTTACCCCATGGCAAAGCTCTCGACAAGGAGGCTTAAGGGGGTGCAAATTTGATAGGCT
ACAAGCATTTGAACCACTTCGACAAGTGAGGTCACAAGCGGGTATCACTGAGTACTTTGATGAGCAGAATGAG
CAATTTCGTTGTGCGGGTGTATCCGTCATCCGTCGTGTTATTGAGCCTCAAGGCCTCTTGTTACCTCAATACCAC
AATGCTCCTGGCTTGGTGTACATCCTTCAAGGTTAGTGTCTAATTGAATATAAAAATTGCCTTTGTTATACTTCA
CTTAGGATTTAGATGTGCCAAATGTTACACCGTTCATATTTTTTAACAATGAAACAATTGTGTTTTCTTTTAGGTA
GGGGATTCACAGGGTTGACTTTTCCTGGATGCCCGGCGACCTTCCAACAACAGTTCCAACCATTTGATCAAGCC
CAGTTTGCTCAAGGTCAAAGCAAAAGCCAAAATCTTAAGGATGAACACCAAAGAGTTCACCACATCAAACAAG
GAGATGTTGTTGCTCTACCGGCTGGCATAGTACACTGGTGCTACAACGATGGTGATGCACCGATTGTAGCTGT
CTATGTCTTCGACGTAAACAACAACGCTAATCAGCTTGAACCAAGGCAAAAGGTAACTATACAACTTAATGCAC
ACAAAATATATACAACTATTTACAAGTGACCCAACTATGATTTAAAGTATACATTAGTGGGATATTAATGAACT
CTGTTTAACTTCATCGATATAAAATTTTAGGAGTTCTTGTTGGCTGGTAACAACAAGAGAGAGCAACAGTTTGG
ACAAAACATATTCAGTGGATTCAGTGTCCAACTTCTTAGTGAGGCCCTTGGTATAAGTCAGCAAGCAGCACAAA
AGATCCAGAGTCAAAATGACCAAAGAGGTGAGATAATTCGTGTGAGTCAAGGCCTTCAATTCTTGAAGCCTTT
TGTTTCCCAACAAGGACCAGTAGAGCATCAAGCCTACCAACCAATTCAAAGTCAACAAGAACAATCAACCCAAT
ACCAGGTAGGGCAATCACCACAATATCAAGAAGGACAATCAACTCAATACCAGTCAGGACAGTCATGGGACC
AAAGTTTCAATGGTTTGGAGGAGAATTTCTGTTCATTGGAGGCAAGGCAAAACATCGAAAACCCGAAACGTGC
CGACACGTACAACCCACGTGCTGGCAGGATAACACATCTCAATAGCAAGAATTTTCCCACCCTTAACCTGGTGC
AAATGAGTGCTACAAGAGTAAATTTATACCAGGTATTTATGATACTATATTCAACACACTATCTTATTTTTAGAT
ATTCTAAGCTTCATACAACCGATTAATAATATGGCATACAAATAATTGCTATTGCAGAATGCTATTCTTTCACCA
TACTGGAACATTAATGCTCACAGTGTCATGCACATGATCCAAGGACGTGCTCGAGTTCAAGTTGTCGATAACCA
TGGTCAGACCGTATTCAATGACATTCTTCGTCGCGGACAACTACTAATCATACCACAACACTATGTTGTTCTCAA
GAAGGCAGAGCGTGAAGGATGCCAATATATTTCATTCAAGACCAACCCCAACTCTATGGTTAGCTACATCGCA

GGAAAGACCTCCATCCTACGTGCATTGCCCGTTGATGTCCTCGCCAATGCATACCGCATTTCTAGGCAGGAATC
CCAAAACCTCAAAAATAATCGTGGAGAAGAGTTTGATGCATTCACCCCTAAGTTTACACAAACGGGCTCCCAG
AGTTACCAGGACGAGGGAGAGTCATCTTCGACTGAGAAGGCATCCGAGTGAATAAGTGAGTGTAATGGAAAC
TAGTATAGTGAAATAAAGGCATCGCATGTTTGCAGCCTAGTGGTATATAACCGCTTATCTCAATAAAAAAGTTT
CTCCGTGTTATATTGTTTGCTTGTTTCTTGTACTTTTCTTAATTTTATCTTTTATGATCAACCAACTTACCTCTCTTC
TTCGTTTTTTTGCCACTCTTCACATATGCATATCGAGGAGACGTATAGTCCACTTGTACTAGATTTATTTTTTGGT
ACTATCTTTTGTGGTTCGAGTATTTCTCGGGTCCGATAAAAACCTTCAATTTACTCATGCTAGATTTTTTCAGCAA
ACCTAGCCACCTAAAATTGTGAATCACATACCTTAAAATTTCTATGCCAAAGTGTGCGATTTTTCTGTTCTATAG
ATCATGACATATTTTTTTGGAGAAAGATTATGATATCTTTTCTTTTTGGATAAATATTATGACATCATTTACCATG
GACATTGTTTACTGCGACATGATTAAGCAACTCTTCGCAATGTGATAAT

>chr5A:14667702-14669702

AATTTACCTCATACTAATAATCAAATAGCTTCAGAAGATGACATCCACAACGACAACTAATTTGCTATCAATACA
AATGCCTAAACCAGAGGATAAAAGCTTCCAAATGATAATATTAAGCAATTCGGCTGATACTTGTTCATCCGAAC
TACTGAAATAATAAGCAACTTCACTCATAAAATTATGTGCAGTATGTACAGGGGTACATCTGAAAATGTCTGAA
ACCGGTTCTGCAGAACGCATCGAAGACGGCCACAACTTTACAGCACACAGGTACGAGCAGGATCCTTCCTAAG
TTGAATCAGCCACAGAAGATACTCCAAACAGGCCAAGAAGCGGACAGCCATGTTTCTTCAAGGCCTCCATACA
AACCATGGTACAGCTGTACAAGCTACATAAATCATCTTGCATAAGCTAGAGCTACGAATTGCATCAGAGACCAC
TACAGGGTCCATCAATTGTACCACTTACATAATTAAGACATTAACTAAATATTACAGGAGAATAAACCAACCAA
TGTATTACTGATAAAACACAAAGTAGCAAACAGAAGACCCACACATCTCAACTCAGATGGCTTTCGGTTGCAA
GTAGTAATAAAATTACATATTTGGGTTACAAAAACATTGTAATGATGTGCTGTGAGTTCATTAAATCTCAATATT
TGGGTTCAACTGTATAAATTGTTGTGATGTGCTGTGGGTACAAATTATAACAGACTTATATGGACATAATGTAT
TCAAGACGACCTAATCTAGGTAAACGACGTCAGAACATGATTCTATCTCAGTATCGCATGTTTAAGATGAAATA
TTCGACTTCTACATGCTTATGCTTGTGTAAATCTCAGCTGTAAATTAGCGGTTGTACCTTTGATGTTTGGACGGA
AGCTCCATGACGAATTCGCGACGGTGGAAGGTTGTGCCAGGACGACGGATCGGTTGTGACGGCAGGCACACA
ACAGGGGACACCAGACACAGGAGGGGAGAACCCGGGGCTAAAAAAAATAACCCCCGTGCGATCTCCCCCCCA
CGACACAAGAATCATCTTTATTACGGTGGTGGCCTGCCATGCGAGCGCACCCAACATGTCAAAGGCCTCTAGG
TGTCACATCTCTCTTTTTTTCCAACTCTTTTGACCCCTCTCGTCACAAGCACCTCGCTTGTTACATGGTTCTTACCT
TGCAGTCGCCTCGAATCTCTGAGATCTAATAATTATCGAAATTTCATCCGAATAATTATTACAAAGAGACGTGG
TCGTCGCGCGAGGTGGAAGCAGCCCGGATCCCACGAACACGCCCGGAGAAGGCCCATCCTCGCCGCCGCGTC
AACTCGGGTCCGCCAAGAAAACCCCGGATCCGCCTCGAGACATCAAATCTACAAGTCAAATCCGCACAGATAG
TTAGGTACGCAGATCTGTGAAAGAATTACGACACATCGACGCCTCAATCGATAGACAATCTTCGTGGATAGAA
TCACTCACCAGCTACGGATCTGGCTCGCCGGACGGGTGGATCTGAAGTTTCCGTTGGAAGAAGGAGCCGCCTC
GCGTCATCGCTTCGAGATCGCCGAAAGCCCACCGCGTCTCTTCACCACCAGGACCCTGCGCAAGAGAAAGGGC
AAGAGAAAGGGCAAGGGGGTTGCGCATAAATCATGGAAGAGAGCCTCACCGTACCGAGATCGAATCGAGTTC
GGTTATGGAGGGAACTCGACTTCCGATCTGTAGGTAGTTGTGATTGGGTGGACGAGGCGTGTTTATCTAGTCT
AGTCTAGATCTGGCCTAGAGGGGAAGGGTTGCGTGTGGCAGAGGAGGAGGAGGACGCCGCAAGGGAATTTC
CTGGTCTGGTTCTGCAAATATGCGCAGGCGTGGTATTTATTGTCTGGTACCGCAAAGTGGACGGTTCGGATGA
AGTCGTTGGTCATGAACGGCCGAGACGCGTCGCACGTGGCGCGAAGCCATTGGTGGTGACAGCTGGATAGCA
CTGGCAGACCGCAACTTGC

>chr5C:580713850-580715850

TTTACAGACTTCTGTAGCAATTACAATAAGGTAGAAACAAAAATACAACATAGCACATTCCAGTCTCATACTAA
TTTCACAGATAGATACTCAAGTAGACAAGTAGGGTGAAGAATATAGATCCATTGCCACAGAACAATTCAGACT
TTTGAAAGAAACCTGACATTTAAGGTAGATATATACATATACAAACATAACTACTGCTCAACAGAGGCGCCTGA
CACAAGCACTTCTTCATGTCCAGAATACTTATTCCTGGAAAACAATGTGGGAAATAATTATGGAAATGGAAATT
GAAGTGTGACAATAAGTTTTTTGCACAACTGCGGATGCTTACCCTTTGTTGTCTATATGTAGAAATCGGCAGAC
ATGGGCATGTCATCGAAAGTCCAAAGGTCCATGTTGCTGACCACGTCCTGGGATTCATCACTGCTTAGAATGTT

TTCAACCGACTCATCTGACCCACCATCCAGCAGAAACTTCATGTATGGCTCCAAGTCGGTGAGATCAACATTAG
CATTACCCATCACAGGAGGTGCTGCTGCATTACTGGCACTATTCTGGAGGAATGCGGATTCATCGACCTCTGTC
AAGGTGGGAATGGATGAAAGCATAGATGTTATGTCAGGGGTCTTGACATCATTCTCCCAGCTAAAATCTGAGC
AGCCGAAGGAGTTGCTCCCTTGGTCAGAGGATAGATTAATGAAATGAGCCTCAACAGCATTCATTGTAGGCAC
GAATGACATGTTTTGGGACTGCACAAATGGCTCAGATAAGGTGTTGTCAACAAGAGGATAGGAACACACATTT
GTGTTGACCATGGTGTTGATTGTTGGCTTCTCTTCGGTGTTGAATTCAGGCACCTTCATAGAGGTAGGCTCAGC
ACAACGCTGTTGACAAGACACAGGAACCTCGTCTGGGAAATTGACCTTTGCCTTCTTGCCACGAATCCTGCGTG
CTTCAGCATCATAAGCTCTGGCAGCTTCCTCAGCAGAGTTGAAAGTGCCAAGCCAGACACGGACACCCTTGCG
AGGATCTCTGATTTCAGCAGCCCATTTACCCCAAGGGCGGCGTCGGATGCCCCTGAACTGGTTCTTCCTCTTCCT
TTTAGCAGACCCTGTTGCACGGTCATCAGCACCAGCAGAAGTTGCAGTTAAACCATCTGAACGAAGAAGCGAG
ATCAAAGATAAGACTGTTGATCTATAAAACATGATAATTTGACATACACTACTCCGTACAAGTATGCTATACTAT
ATGGTAGTGTTCCTTACTTGAACAATTTTGCATAGGCCTGGACTTTAAGGATTATGTTGCATCAGGGCATATAA
TATTTTAGCACAAGAACTGGCTGCTAAATGAACATTAAAAAAATACAGAGAACTAATGTTCAGACCATTCCGAG
GCTGAGAAAAAGAAAAATAAATCTACAAATCACTGCTACAGCAGCAGTCTTCATACTGAAATATCAACCTTCTC
TACACCCAGTGTGGCATCCAAGAGCAAAATAAATTTACAGACAAACTACGTATAAGTTTACAAATGAAGTCAG
GGTAAGTCAGTTTTAAAATTAAACTCTGATTATCAGCAAAAGCCCAAACTCATATGCTTGTGAGGAAGCTCCAT
CTTATGAATCACTAGGAGTACCAACTAATTTCAGATTCATGATTATAGATGTACCGATAATAATTAGGAAAATT
ATCATAAACTAAGAATGGACATAGCACCGGAGATCTGACTGAAGGACAACACAGACTCGCTGAATTAACCGAA
GGGAACAGATCGAAGTACAACTAACTCAAATCTACCTCCCAAATCCTAAACAAAACATGCAATTCCAAACACG
GATCCCCAACCAGTCATCCAGAAAGGAAACCCCCCAAGATCTCGCCCGAACAGACCAACAAAACAACAGAAAT
CGACTAGGATCCATGTCCTATTACCTCCGTGGGCGCCGTTCCTGCGGGCGGCGAAGGGCTTCACCTCGTCGTC
AGACCCCACAT

>chr5C:604992209-604994209

GCCAACAAACATGATAAATTCTTCGGTTATTTCCAATATACCACACCAAAATGCAAGCACAGTTGGTTCAACAC
AGACTTACAAGCACAACCTGAAACCTCCCTTTTTGCAAAGGAGCAAGCAGCACAACAAAACTTATTGCACTGGC
TATATATATATGTCCTTCACTACAACAAGCGACTGTTCACCATATACGAGAACCCTGCAGATCGATGCTCCATTC
CGCATACATGCTTAGCTCGATTTTGCTTGCAGGATCTCTTCAGGGAGCAGCGACGTTCTCCATGGCGAGCGGC
GCGATTTCCTCGCCGATGATCCCCGGCTCGCTCCCGGCGAACCCCAGGTCCAGCAGGACGTCCTCCCAGTTCTT
TGCAGGCCCCTGCAAAGCACCATCGATCGATTGAATTTAGCAACGATTTACTCTTGGTTCTGCTGCGAAGTACT
GTACTAGAGTTTAGCTTGGCGACGTACCTTCCAGGAGAGATCCTGGGCCATACAGTTCTTGACCATCTCCTGGT
ACGCCGGCGTGCCGACGACCTTGACCGCGCGCTTCAGCGTGGTCGCCACTTTCTTGACGTCTGCAGGCTCCACC
ACCTTGCACTGCAATGGTCACGAAAATAAAGAAAAACCAGTTGATGATCCGAGCCCCCCAAAGATAGGCGAG
ATCAGTGGAATCAGACAGAGGTCAATGGAGCCTACGTCGACGCTGAGGCGGCCCATGTGGAATCCAGTCTTC
CCTTCCACGATCGTGTCCACGAGTCCGCCGGTGGACGCGCACGCGCAGGGCTGAAATTCAGTAGTAAGAAATT
TCGTTTACGATCATGGATCCGTTTCAGGATGTACACTCACGCAGGAGCAGCAGTTGAGAAACTGGCAAGAAGA
GGTTTACCGTTCCGTAGCGCATCCCCTGGAGCTGGATGAGGCCACAGGGCTCGAAGCGGCTGGTGACGGCGA
GCACGTCGGCGCCGGCCATGATCTGGTGGGCCAGCGGCGCATTGAACTTGACCACGGCCCTCACCTTGTC

>chr5D:393480179-393482179

GTCATTACCGAAGACATTCCTTCCCTTAATTTATCATTAATATAGCTCCGTGATGCTTCTGTTTTGCTCATCCACC
TCCAGTGCATAAAAAAAAAGGAATGCGCAAGGCTACAGAAGACCTAATCTCTCCATGGATCACTCTCGGTCCT
CACTTCAGAGGGTTCAACAACCTCCGTCATTGTAGCTTCAGAAGAGTCGATCTCGACATTGGTCTCACTGGTGG
TTGCTTCAGCATCCTCGGAGTCCGATCTTCCCCATCTCCCGCCAAGGGCAATCGTCATCATCTCGTAGATCTTGC
CACCTAACTCAGCTGGACTCTCAGGCTAGACAACCCAAGTAACAATCATTAGGTTACAGACTATAATTATCACC
ACAGACCATGAACACGAGCACAGTTCTATTGTACTTACAGTATATCCACTGGAGATCAGAGCAGTCTCATACAG
CAACTCAACTGCCCTCTTGGCTTCAGTACTTTCAGGCTCGTTCTTGCAAGCAGCCTGTAGTAATTGGCGATACAT
AATGAGTGAATGGACACCAGCACGCAGTAGGTAACATTTGCCAGAACAGATTATTGTGGTTATCTTACACTCA

AGTCCTTGACAATAGGGTGGTCGGGGTTGATTTCAAATATTCTTCTTCCTCTCATGAACTCCAAGCTTGATGTGT
CACCGAGCGTTTGCGCTTTCATGAGCCTATCAAGTTAAAGAAGTTTTAGGAAAACAACTGCATAATTACTTTGG
CAAATGGTGTGTTTGTTTAAGTGATGTGAGCCACCATGCTACCTTTCCATGTTGGCTGACCAACCAAACTTGCC
AGATACAAGAACACATGGTGAAGAGCTGAGTCGCTTTGAAATTTGGACCTTGGCAACCTTCTCACCAAGCTGT
TGCTTAATCCAGTCACAGAGAAGAGTGTATTCCTGCTTGCTTTCTTTTTCTTTGTCTTCGTCTTCATCACCTGGAA
TAGTGAACATGCGTTAATACCATGCAAAAACTCCAAAAGTTTGCAACCAATCACACAAAAATTTCAGGTTTTCTT
ACCTAATTCTAGGTCTTCTTTGCTGATATCAACAAACTTTTTCTCTTTGTATGTCTGCAAATTCTGAATAGCTACT
TCATCAATCGGTTCGATGAGGTAGAGTACCTAAGATAAGAAAATAGTGTCGGTATAAGCTAAATCGTAGTACC
ACAGAAAATCAATTCTATTATCAACAAATAAGCTAATCTCGTACTTCAATATCTTTCTGAACCAGCTTTTCCAAG
AAAGGAGCAGTCTTTGCACTTTGAAGACTGTCTGTAGCAATATAATAGATTGCCTTTTGGCTTTCAGGCATATT
CTCCACATACTGATCGAGACTTATCAAATCCGTCTCGTTTTTGGAGGAGTGGAACCGCAGCAAAGGAGCAAGC
CGTTTCTGATTTCCTGAGTCCTCAATGCAACCAAGTTTCATAAACTTCCCAAAGCTCTCCCAAAATTTCTTGTAAT
CCTGCAGCACAAGGGTCAACAAGCTTCAAAACGAACTCAGTAGAACAATATTTGGGGAAGAAAATAAAGCTA
CATACAGTCTTACCTCTTTCTCATCCTTGTCAGCAATATCCTGAATCATATCAAAAGTCTTCCTAACAAGTCTCTT
ACGCATGATCCGGACCTGAAACATAATGATGGCGATCATTAGTTTCAATAACCACATAATTAAGAAAACCACTG
CATATTAAGTCACAAATGCTTTCCAGTAACTTACAATACGGCTTTCTTGGAGAATCTCACGAGAAACATTGAGA
GGGAGATCATTTGAGTCAACAACACCTTTCACAAAGCTCAAATACCTGGGAAACTGCAGAGAGAACGTGAAAT
TTCAGCCCAATACAACATTTTAATCATAGTAACCAAAGAAGTAACGTCTAACTCTAATATCAGGAGATATAATT
AAAGAAATTGTGGCAACATACCAGCTCGCCATCAAAGTCATCCGAAATGAAAACTCTCTTGACATACAG

>chr5D:408000122-408002122

GCAAACAAGAAATAATACTGCAGTCCAGCAAGTGTCTAGCCTATCGTTGCATCCAAACCAAATGCATTCAACTC
AGCAGCGGAACATCCCAAACGAACGCCTCCCCCCTCGAGTTCATCCTACAAAGCCAGGGTGGCTGTACTCCGC
CCCTGCAGGTCCCAAGCAGGAGAAACGCGAGTTAGCTGCCTCGTAATCAGACAATGGCAATCGCTGAACCTCC
GCTCAAGTCAAAACTTTGTGCACTTGCTTACTTACCTCCTTGAGCTTCTGCTGGTAGAGCATCTGGACCTCTCTC
CGGAGGGCGTCCACCTCCCCGGCGTACTCCTTTTGCTCCTCCGACGGCAACTCTTCCAGCTGCACCCCCAGTCT
GTACAGCACGCCGTCGAGGTCGCACGGCTCCAAGTCTCTAGCATCGGTCGTGGGAAGGTTCAGTAAACTCCTG
ATGGCCAACAAAACCTGTGTAAAAGTGACTGTCACTCTCCAGACGAGCAAAATATTACATGTCTAGACTCTCTC
CTGTTAAGCAGCGTCAAGTGCATAGTCGCATACAGATAGATGACATAAACTGAACTGGCAAAATAAGTAATGC
TTGAGACTACATTAGACGACAAGATACAACGCACAACTGTCTAGGCATAACCAAAATACGATCTACAGAACTA
GGGGCGACTCTGACCATACCTTTTCTTGAAGGTCAAGGTCAAACTTTGATAGCATATCAACCATTAACTTCAGG
AAAACGCGTTCACTTAAGAAAGCAAGCCCCAAGTTCCCAGAGTTTAGCTGAAAGTCCGCTAGATCCGTTACCA
AAAACACCGCCTTCTTCTGAAGCCGGACATCGGTACCGTCGCTTGCCAGTATGTGCTGCGGAAGGGTGCAAGT
TTTGTCACAGCTGAACCAATTTATGTGATTCTATGCCTAAGACGGGATTTTTCTCACTACCTGCAACATTGCGCT
TCCCCTTTCCGAACGGAATGCCTCCTGACCATATACGTTATCTCTGATCAAGGCAGATATAGCATACATTGCCTT
CGCAGCTTCTTCTGCTGAAGTGGAGTAGCCCATCTTCACTAGTCTTCCCAAAGCTCCATAGCCAAGGATCTGGC
CAAGAAAGAAACACTCACATCATATTACATGTCTAGAACAGAAAATGATGATAAGAAAGACTTCAACAAGAAG
CACCCCTGTGGTCTGGGAAGAAATACACAAAGTAATTAACTGTTTAACCATCAAAGAAACATGAAACGGAAAT
ATAAAAAGAAAGCATCCCCGTGCTCCGAGTCTGAGCTAACATGAGAGAATTATATATATATACTACTAAAACCA
GCACAAGACTATTAATAATGATGCAGATACTCTGCAGAAACAAGATACGTGCATTTAAATCCAATTGTTCCTTT
GTGTACGATTAATGGCTTTACCTGGCTTTGGACAAGGGCATTATTCTGACAGGCTTTACCCAGGATCCATGCAG
AGGTGGTCCGTATTTCTTCATTTGCATTATTAAGATCTTGAATCACAGCAACAAGGCCCCCAAGTTTGTCAAGG
TCTGATCACAAATGCATGAGCAAGACACATTTCAGAGAATCTTCCAAAAGGTCTCACAAACAACTCTAAGCTTT
TGTTGCATTCAATGCTGACTCTTTTTTCAATAAAAGGTACTCCCCTCTGATCACTAGTATAAGACCTTGTAGATA
TTTCACTAGTATAAGACCTTGTAAATATTTCATGCATTTTGGTCTGTGTATCATAATAGTGAACAGAGTAAGCAG
TAAAATCACATAGATATATGGAGCATCCTAGTAAATTGTTACATGTCTAAAACAGTGGCTAGGAAAAAAACCAG
CGAATATTTCATTGGTGTGAACTCTAACTGCTAAGTGTTAGATGTTTCAGTTATACATCGTTACAAGTGGACATT

CTAGCTGACCTGCAGAGTGGTGAGATACTAGCATACTGATGGGGGGCTAAAAGAAAGTGCGGATTGTTACCA
TTGGCATT

>chr5D:408443986-408445286

AACTTAAATCCTTTAGGCTGAACGAACGCTGGAATGTTGTTGTAAGGGGGTTTGCAGCCTATGAGGGCACGGA
TGATGACACAGAAGCATTAGGGATTGCTAACAATATGCCTGAGCTCAAAGACCTCCAGTTGATCGGCAACAAC
CTAACTAATGATGGACTGCTGGCAATCCTTGACCATTGCCCCCACCTTGAATCCCTAGACATACGCCAGTGCTTC
AACCTCCGGATGGATGATGCTATGAAATCTAGGTGTGCTAGAATTAGAGATCTCAAGCTTCCTCATGATCCCAT
CTCTGATTTCAAGTACCGAGCTTACATGGGAAGCATCGGGGACGACCATGGATCTGATTTCGAGATCGATATG
TACGACGATCTGCTCGATGTGGTCACGGATGATGATGAGGCTGAATTTGATGATATGGATGATTTCGATGACG
CAGGCTCAGAATCTGCCATGTATGATGATGTATTCGATATCTGAAGGTGTTGTGACCAGCACTCTCGGTTTTCA
CTGAGGCGGCAACTTTGAGCTCAAATGATTCGGTGTTTCCCTGTGTTGGAATTCTCGAAGTTTGGTTTGGGCGC
TTGGTTAACAGGATATTGTCTTATGTTTGCCTTCTTTCTTCACTCCTGTTCTTACAAGAAACTAGTCTACTCTCTAT
CTGTGAGGCTGGGTTGTTCTATTAGCACTTTCTCTCTAATTTGTGCTCCATTTATGACTTGTCATAGAATCGTATT
ATTGTAAATGAAACATATGACGCCCCTTGTTGTTGTTTGTTGAAATAATTAAGGGATGGCCGTGTATGTCCAGT
GATCCAGTAACACGAGGCAGCCAGTATTTGGTTTGTTGTCACATGGATTAGTTCAGGTGGATAACGGGAAGTT
GAGGATTTTTTTTTTTTTTTGAGATGAAGATGAGGATTCTGAAACTATTGGTACTCAAAAGTAACCGTGGCGTG
GCTGGTACTAACGGTGCAACACAGTTAACAACTTAGACACGTGCAGAACGCAAGAATACGTTTAGCCTTCTAA
CCTCCCAGATTAAATATGCCGCCAGTAGAGGCCGATGCGCTTCCTACACGTTGCTCCATCCCTCCTACGATCTTC
TTGTGAGGGAAACATACTTCCATGGTGAGGGCGTTGCAAGAGGGCCTTTGTGGCCGGTGACATGGACGGCTG
TGGCAAGTGTGACGATCACTCTCATTGTTGGACGGACTTGGAGATCGGTCATTAGGGATGCAAGCGGACGTCC
GCTCGACATCCAAGCCTGTCCGCCAGTCCTTCAAATTAGGACCT

>chr5D:483866350-483868350

TCCAGAGATTTAACTTAATAGATAACGAAATGTCAGGTAGAACAGACTACCAAACCCAACGGACTTCACATGG
CAAGAGTAGTACATTCATAGAGTTCGACAAGGGGCACAATGGCCTCAAAAGTCCATTCACTTCGTCGCGGGTG
GGGGAGAGAATTGGCTGGGTTATTTAGGTGATAACGGTTTTTTGTCGATGATGATGATGGGCTCGGCACATGA
TCTCCGGTTGGCCATCTAGGGCTCTAGCCCATGGCGTACTTCTGCGTCCAGGAGCGAGCAGTGGACTCGTACTT
GGCCCTATCAGTCTTGTACATGTGGGCGATCTCAGGCACCAGCGGGTCATCAGGGTTCGGGTCCGTCAGCAAC
GAGCAGATTGACAGGAGGACCTGTTAATACACATACATGTAGGTAATCTTAGATAAAGATCAATAGCTGAAGA
TACTTGTGAGATGAGCTGCTGAAGGAACCATCAAGCTACAATTAAAGAAGGAACAGACAAACAGGAATGTCG
GTCGTTACCTTTGATATGGTCAGAGCCGGGCTCCACTGCTCCTTGAGAATGTCAAGGCAAATGCTGCCGTTGCT
GTTGATGTTCGGGTGGAAGACCTTGGTGCGGAAAGAGACCTGAAAACATGTTCATGTCTGGATGTTAAGAATA
CAATGGATAATAAGATAATAGGTAGCAAACAAGAAGTTACTTAAGGATACACAGAACATCTGCAAACTTAAGT
TCCAGCAACAAAGAATCAAAATAAACTCTCTTACAAGCTTCGGTGTCCGCCCCCTCCTCACAAACTTAAGATAA
AGACACAGGAAGGGCCAGGATTAACCAATACTGCTTCAGTTCGAAGGGCGTGAAGGGGTATCAGCTAATCCT
GGCCGGCCTTGTGTTTTCAGTTTCCTAAGTTTGGAAGGCAGGGGAGGACACCGAAGCAGAACTCCTCCTACAA
AGGTTAGCTAAATTCCAGCAAAGCATAAACTCATATTACACATGACATTACCTTCGGTGGCTTGAAAGGATAAT
CTGGTGGAAAATGAATGTTCACCAAGAATAGACCACCAGTGAATGGGCTGTCCGAGGGTCCCATGATAGTGG
CCTGCCAATGGAACATGTCCTCACCCACAGGACCTGCCAAAATAAGCAGTAACAAAGCGTTATCTAACAGCCAT
ACACGCAATTGCAGCAATCCAGTGTAGGATGATTAGAAACAAGACACATTTTCTGCAAGCCCAAACTGTTGCC
AGTTTTAATTATGGTGAACTAGTTCAGGATTAAACAGAAAATTATGGTATGCATTACCAGCAGAATGATTTTAT
AAGTACAATTCACTCAGACATACAATGGCATGTAAACAAGATAAAATAGGTGGAACCAATTGCTGTACTGATA
TAACATAAGAACCAGGACATCATGTTCACAAATCACGAATAGTACATACAAATCTAGACGGATATGTGACAAA
CAATTCAGGACTTAGGGAGTCATGCACTCTCATATGTCAAAACATAGACGAAATCTACCTCCTGCTTTGATGAA
TACTGCTGGGCTTTTTAAGCCTTCAACACCAACAAGTCATGCCTAACTGTCCACTGAAAATTCTAGTGTCCTTTG
TTGTTTTAATGTCTTTCGGTTATTGGCAGTTAAGGTAGCATACGATGCAATTGAGGCCGTGCATAACTGTAAAC
CGTATCGACACTGACAATAGTTTAATCTAACAGGACAAACGGCTCTAATGACACAAGGGTTTTTAATCATCGA

CGCATAGAGATCACACGCTTACCGTGCTCATATAAAAGGCCATAACCATCGAGTTCATGAGTACTATCCACCAA
TCAACCGATTCAGCATACCTAAAACGTACGTAAAGCACGGCTAGAAAACCAACGACCTAATAACCCCATGACT
GACTAAAACCTAATAACAATCGAGCCACGCAGACCGCGTGCTCGCCCCCTACCGCAATTCGTCGAGATCTAGA
AGGAATCAGACGGCGTAGAG

>chr6A:185435200-185437200

TAGGGAAACCAGTGGGGGTGTGAAGCCCCTAGTTTACTAGCACAAAAATACAAGTATACATATGATTTCATTC
GCCAGTTGATAGAAATGACACATTCTGTGCACATCTACACAACTCTATCGCCGGGTACACAACTACTTACTGTA
AACTTCAAGACCTGGAACTTGGAGCAGCGAACTCGCTATAGTTGAGTATGTGAAACATATTACAGGATCTCAA
CAGAAAAAAGAGAAAATTACAGTGGCTCCTGCCTGGGGAAAAACAATGGTGTATTCAGTTGAAGTTTTTTTGC
AGAAAAGAATGCCCATCCAAATGTAGTCAAGCTTCCTCCATGACATGTTCAGATGTGCTGGTCTGCCGAAGGC
CAGCAATCTTGCATATCTTCCTGAGAGTAATTGCTGCAAAAGAATGAATACCATCACAAACTAAACTGGCAGTT
CACATAAACAAATTGACGGTTGGCAGCTTAAGAACATTATTCTATTGTTGCAAAATGCCAATGCAAATGTCTTT
CAAAGAGAAGTTCTAGTGTGAGAAACACTCGAGACAGGCGTTTGAATTTCGTGTTGATGTAGATTTAATTACTT
GTGTAGCAATTCTGATTTCGAAACCCAATCCTTTCAGAAAGACAGCATGTAAACACATCTAATCATTTTGCTCAT
ATCACCGGGGTTATTCAGCACAAATGTGTACATTCATGTATCCATACAAAATTAACAAGTGTCACCGGGGAAAA
TAAAAGTTTAATTCCCCATTTCCATTTTGGTCACAGTTCCAGCCTAATTTGAAGCTCGTTTTCCCTCTTTAGCACT
GATGAATATAGCATTTTCTCCTCTGAACTTAAACATCACATTGATGGTTTTGTACCTATCACCTTTCCTTCCTCCA
CATAAAAATCGATCCAACTAACAAGGCAACACCAAATTCTGTAATTAAGGTTATTTATATTCAACATTCCATGAG
ATAATGATACAGAAGAACACGTATAGAGGCAGCAGAAAAAAAGGACCTAAGAATCAAGTCGACGCCGTGCG
GAGGGGTGGTGGAAGCCTATCAGACGTCGAGGGTAACAGAAGGCATCTCGAACTCCGAGGTGATCTCGGCG
ATCTGCTTGCGGACGCTCATGTCGATGAGGTTGGAGCTGTCGCTCCCGTACTGCACGGTGAATCCGGCGATGA
GCTCCGGGTCGATGCGGGTCTTGATCCGGACGTTGGCGGCGCCGGTGATCTTCTGGACGTGCTGCGCGATCTG
CGCGAGGTCCTGCGACTCGAGCTGCACGACGGAGGTGACGACGGCCTCCTCGGTGCCGGTGAGCGCGTTGTA
GGCGGTCTCGAACGCCCGCACGATCTGCGGCACGAGCTCGGCGCGCTGGTTGTCCACGACCACGTTGAGGAA
GTTGACGACGTGCGGCTGCAGGCTGGACGACTTGGCGATCTCGTCGATGAGCTCCGTCTTCTCCTCGCGCGGC
ACGGTGGGGTTGTCGAAGAACTCCGCGATGGCCTCCTCGGAGAAGATCTTCTCGAGCTTCTCCATGTCCGACA
CGGTCTCTTCCAGGGTGGAGCTCTCCGAGGCCACCTCGCACAGCGCGGACGCGTAGTTGTCCGCCGCCGGGTG
GGCGGCGGCTCTGGCCCTGCCGAGGTCCCCGCGGCGGGGGCGGGCAGGGGCGAGGCGGAGGGAGGGAAG
GCCGCGCGTGACCGCGCAGGCGTAGGAGGCGGCGCGGGGCGCGGCGGCGGAGGAGGGGGACGCGGCCGC
GGCGGCGGGGCAGAGGGTGATGGAGGTGAGGCGGAGGGCGGCCATGGCTCGCGTCGTGGAGCGTGAGGTG
GGGAGTGGTGTGGACTTGGGAAGCTGGGGGCTTGGCTTGGCTTTGGTCTGGGAGCAGAGGAAGAGGGGTTT
GGGTGGGCAATGGACGGCTGGGATTGAGTAGTTGCGAGATCGGACGGCTGAGGTGTCCGAAGACGTCCAGC
GTGGCTGCAGATGTGTGGCCTGTGGTGGGATGAGG

>chr6A:256163912-256165912

AAGAGAAGTGGAGGAGGCATGGAAGGAAGAAGTGGAGGAACTTCCTCGTGATCACCACCTTGTTGCTCTTCA
TGGTAGGTTATGTGTTCTACTTCATCTAGGTAGAGCTACGTCGTTGGAGTTGTAATGTGATTGTGAACTTGTAC
TCGAAGGTGTAATGTGATTGTGAACTTGTACTCGGAGGTGTGAACTTGTGGCACAAACACTTGATGTATGAAC
TTGTATGTCATGATTGATGTGTAATGTATTGTATCATGTTGGTTCTATTATGCTTTGGCAAATTGCAAGTTTCTG
AAAAATTCTGTGCTCTTTATACTGTATTCTGGACTATATTGTATATTTGAATATCTGTATATTATTATTTATAGTG
GTCTGTAAAACTTGAATTTCAAAAATATTACCGGAGGCGCATATAGGATACTATAGGAGGCGCATATACTATA
AATATACGAGGCGCATTGTAAAACATATGCGAGGCGCATATGATATGCGCCTCCTATAATTCACATATGCGAG
GCGCATATCGATATGCGCCTCGCATATGTGAAGTATAGGAGGCGCATATCATATGCGCCTCGCATAAAAAATA
CCCGAGGCTTGAAGCTAGAGGCGCATATGATATGCGCCTCGGATAGGCTTTTAGGTGTGCCTCGCATAGGCTT
TTCTCTAGTAGTGCTAGAATCTTGAGTAGCAGGTATGCATAGTGCGGTACTACCAGAAATCTTGTAAATGCCAT
GCGCCCGAGGATGGCTTGATACTGCGATGGCCAGTCCACCACTTCGAACTCCAGCCGCTCATGCCTGAATTTTT
TGGGCTGGCCGAAGATGACGTCGAGCCATATATTTCCTAGGGGATATATGCCCTTCCCCGGGACTATGTCGTG

GAACGTCGTGTCAGATTGTTCCAGGCTGCACAGCGGGATCCGCATCACTGCGAGGGTGCTGGCGAACATCAG
GTTGATGCTGCTTCCGCCGTCCATGAAGACGCGACTCATCTCGAATCCTCCGATCTGCGCCTCGAGCACCAGGG
CAGCGTGACCTTGGCGAGGGATCTAGCGAGGGTGATCCGAACGATCGAACATGATGTAGGACTCCGAGCCGC
TGAGGTACTCAGGGATGACAGGTGAAGCGGTCATGGCCAGGTGACCTGTCGGGTGATCTTATTTTGCATCGTG
TTGGTAGGGCGTCCCTTCTGGATCATCAGGGACCCTTTTGACGGTGGATATCCTCCTGTGACATTGTTTGGCGC
AGGTGCGATCGCTGGATTCGACGGTCCAACGGGTGGTGGTGGAGGAGCTCTGTGGGCGATGTCACCTGGTGC
AGGTCCAGGGCCAAACCCAGGTTTCACGACCTGACATCGCATTTGGGTGAGACCCTCGCTTAGTTTCTGGAACT
CGCGATAGTCCTTTAGCATGTGGCTGGACTTCTTTTTACCATCTTGCTTGTCGATGTAGAAGTGGATGGTGCAA
GGGCCAGACAACTTGTCGTATGACGAGGGCTCGTCGTCACGGCTGTGTCGCTGCCATGTTTGTGTAGGGCGCC
CTCCATCGCAGTAGTTGCCTCCACGGTATCCGCTGCTGCGATTGCCGTCGTTGCGTCGGTCGGTGTAGCCAGCC
GCAACCATGTCGACTCCGTCGTGATCGTCATAGACGCGCGATCTTTGTTTGCTGCGGCATTCGCGTCTACGGCC
GTAGTTGTCCTCGTCATCCGAATGCATTCGGTGAAGGGAATCTTCGCCATCTGCCCACCTGTTGGCGATGTCCA
TGAGATGGTCGAGCGTCTTGGGCTTGACGCGCCCAAGATCTTCCTTGAAGTCTGGGCGCTTGACACCATCCTTG
AAGGCATCGATCGTCCTTTCCTCAGAGATATGCGCGGCGAAGTTCTTAATGTGGTACCAACGTAGGATGTACG
AGCGAATGGATTCGCCGGTGCTTTGGGTGCAGGCCCGCAGTTCCTCGATCGAGGTTGGCCTCTTGCTGGTGCC
CTTTAAGTTTCAGATGA

>chr6A:314373547-314384547

TTGACGACAAAACGAACTCCATCTTTATTGCATGGCACAGACATAGTACCAATTATTACACATACAAACATGCG
TCTTTGAGACATCCATGGATCAGACTATTTTATTGGAGGTAGATCCACACGGAGCACTACGACATGCACGATG
GTAGACAATGACTACCACATTTCATCGGAGAGGAGCGCAGCTCCGGTGCATGGCCCGCTCTGGCAGCCGGCG
CCACAGTAGTCGCCTCCGACGCCGCAGTATCCCCACTTGCTGCAACATTGGTTGCCCGAGCAAGGACTGCTGCT
GCTGCACGCGTTAGGCACAACATCTTCATTGAGCTTGGCTCCAGTGCACGGCCCACTCTGGCAGCCAGACCCG
CAGTAGTCGCCACCGAGGCCACAATACCCCCACTTGCTGCAGCATTGGTTGCCGGGGCACGGACTGCTGCTGC
TGCACGCGTTTCCGGGCACAACATCTTGATTGAGCTTGGCTCCGGTGCACGGCCCACTCTGGCAGCCGTCGCC
GCAGTAGTCGCCGCCGAGGCCACAGTACCCCCACTTGCTGCAACATTGGTTGCCGGAACACGGACTGCTGCTG
CTGCAAGCGTTGCCGGGAATGGCATTTCCATCGACGTTGGCTCTTTTACCATAGCAGGGGCCGCTCTGGCAGC
CGGCCCCGCAGTAGTCGCCGCTGAGGCCACAGTACCCGTACTTGCTGCAGCATTGGTTGCCCGGGCACGGACT
GCCGCCGCTGCAGCCGAGGGCGGTCGTTGTTGCACATGCAAGGACCAGCAGACCCAATACGAGAGCCTTCAT
GGCCATGTTCGAAGCTCTCGCTTGCAGTGGATGGATCTCGGAAGATTTTCTTTTTTAGCTTAGCTAGTTTGGTG
GATGAGAAAGCTTCCGATGATGAAGTATGAAGCTTGCGAGGTGCCATTATATAGGCATGCAGTACGTCCGCG
GTGCCTCCTCTAGTGAACCCATGGACAAATATTGTTATCGGCTTTTAGTCACACACGTAGACATGCCAGTCATA
GACAAATATTGTTTGTTCCAAAGCAGCTTTTAGTTGTGTAGGGTTGTAGCAATAAAATGGCTTTAAATCACTTTT
CTTTGTTGGATTAGTATTCGGTTAGGGAACATGTCAAACAAAAGAAGGTATTCTGTTAGGGAATGACATACAT
ACACATCAGCACATTTATGACACTGAAAATTAATATCACAACTGTATTGAGTATGAAAAAGATCATGCACATGC
AGTGGTGTAGGGAACCAAACCGGTGGCACGTGTCACACGAAAGCTCAAGCTCAAGACAATGACAATGGAATG
AAGAGATGGTAGCAGAAGAAACAAGATTGGCCAGGCGGGCATGAACGCTGGACGTACACTTAATCTTCTTCA
TCTTGTATACGCAAAGCAAAAGAAAAGAGTGACGAGTTAGAGGAGGAGCCAAATGAAAATCACTTTTCATTTC
GCTCGTGGGCCTAGCTAGTATAGTATGTGCAAAAACATTATTTGGGAAAATGTTTTGAACTGGGCTAATATGC
AGTAGGTTATCTTGTTAGGATCCTTCTGTTAACTAGTCAAGCCTGTTTGGTTTAGTCCCACCTTGCCTACGGGAG
GAGGCCACACCCATCTTATAAGGGAGAGATTCCTTCCTCCATTCAGTCCGGGTTTCTGGAATGAAGTGGGCTCT
TTTCTTATTATTGGGCTGTGAGCACATGAACGTCCAGAACGTGGACTGAGTGCTTGGCTAGCCAAGTGGACGC
TAACATCTGGTATTAGAGCCTTTCTGAGGGGCTTCTTAGGGAAATGGGATATTAGGACCCCTCTGTTGACTAGT
GGAGGCTGCTGGACTGAGTACTGGGCTAGCCAAGTGGACCTAACATATCCCAATCTGGATCTACCTCTTTGAG
CATGTTCAACTCGTTTGCAATCTGTGTGGAGGGAACATAATGCAAGAGTTTTGGATAGGAGTTTCAAACCTACC
AACATCCTAATAGAATAGATTAAGGCGGAAGCAAAACTCTAATCGTTGGCTAGTAGGAATCGTTTGAACCTTTC
AGGACCTTAATTCGAAATCATAGAAGGATAGCTTCGTTCGGCAATGCTTATTTGTTGTTTTTATTTTGTTTTAAT
ATATTTTTGGGGGTCATTCTTTTTGTAAATTTATGTAAGACCTTAGCCATTCCTATGACTTTTCCTCATCTCATAT

AACACATGCAAGGCACTTGCATGACTCAAAAAAAAAAAACTAGACAAGAGCTATAGCTAGTGAGGTTGAGTTA
GATCGAGATAGAGAATGACTTAACCATAAAACACCCATGCATGGTTCAACAGAAAACCAATATAGTGACAGCA
ATGTGGTACATATGTTTGACGACAAAATGGACTCCATCTTTATTAGTACACGGCACAAGCATAGTACCATTATT
ACACATAAAAACAAACGCATTTAAGACATCCATGGATGAGGCTATTTTATTGGAGGTAGATCCATAAAGAGCA
CTACGACATGCACGATGGTCGACGATGTCTACCACATTTCGTCGGAGAGGAGCGTGGCTCCGGTGCACGGCCC
ACTCTGGCAGCCGGCGCCACAGTAGTCACCTCCAAGACCGCAGTATCCCCACTTGCTACAACATTGGTTGCCTG
GGCATGGACTACTGCTGCTGCACGCGTTGGGCACACCCTCTTCATTGAGCTTGGCTCCGGTGCAAGGCCCGCT
CTGGCAGCCGGCACCACAGTAGTCACCTCCGAGGCTGTAGTATCCCCATTTGCTGCAACATTGGTTGCCGGGG
CACAGATTGGTGCTACGCGTTGCCGGGTACGTTATTTTCATTCAGCTTGCCTCCGGAGCATGGGCCGCTCTGGC
AACCGGCACCACAATAGTCAGGGGTGGTGCCTCAGTAACCCCACTGGCTGCAGCATTGGTTGTCGGGGCACG
GACTGCTGCTGCTGCACGTGTTGCCGGGCACAACATCTTCATTGAGCTTGGCTCCGGTGCACGGCCCGCTTTG
GCAGCCAGAGCCGTAGTAGTCGCCGCCGAGGCCGCAGTATCCCCACTTGCTGCAGCATTGGTTGCCAGGACAC
AGACTGCTGCTACTGCAAGCGTTGCCGGAAACGACATTTCCATCGACGTTGGCTCTTTTACCATAGCAAGGGCC
GCTCTGACAGCCAGCCCCGCAGTAGTCGCCGCCGAGACCACAGTACCCGTACTTGCTGCAACATTGGTTGCCT
GGGCACGGACTACCGCCGTTGCAGCCAAGGGCGGTTGCTGCTGCACAGGCAAGAACCAGCATGCCCAGAGCG
AGAGTCTTCATGGTCATCTTAGAATCTCTCGCTCGTAGTGAATGGAACTTAGAAGGTTTTCTTATTTAGTTTAGC
TAGTTTGCTGGATGAGAAAGCTTCCGATGATGGAGTACAAAGCGTGCGAGGTGCCATTATATAGGCGTACGT
GCGTGGTGTCTCCTCTAGTGAACCCGTGGACAAATATTGTTAGCGGCTAGACGTGTCGGTCATAGACAAATAT
TGTTTGTTCTGAAGCAGCTTTTAATTGTGTTGGGTTGTAGCAATAAAATAGCTTTAAATCACTTTTCTTTGTTGA
ACTAGCGAAGTGGCCCTCCCATTTGCGAGTGCATCATCTTTATTACTACCTTGAACTAGCGGAGTAGATATTTA
CTTTAATGGATTGGAAGTTAATAAGTTAATTTTAGAATGTCATAAGCAAGGACATATTTACTTGTGGTTTACCG
ATTGTTATGTCATTTGTAAAATTACCTTCAAATTATTTACTGCATGATCATTTTCACATGGCAATATTTATAATAA
AAATAAAGGTATGGGAATTTGTATATCATACCTCTAAATGTTTGAAAAGAGAGTAAAATCCCGACAATTGGATT
TCCTAGTCAGATTTGTTCTGGCGATAACTAATATTTGAAAGAGATCATCTATCATAAATACTAAAATATTTTCCG
ATGGAGTGATCTTACTCTTGTGTCCAATACAGAATTAAAACCTCTACAACTGATTGTAGTAGTTATAATTTACAT
ACAAAATACAACTTCACCTTAAGTGTATAGCCATATGTATCAGATCATTCTTTCATCCTAACCTTGCAAAATATT
GGCAATGGAAATAAAAATGTAATAAATGGTCAAGAGATGACCATATTAAAAAGTTCAAAATTATTGGTGTCAT
GCATGTATGGTTATTGTCTTGAACATGAACTTATCATGATGTAATTCTTGCAAGATTCAGAAGCTAAGAGTCAG
CCATGCATATATTAACAAATAACATATCTCCAAGTTCTTTGCAAGTCCAACAGAATAAATAATATATGATTGAAA
GCTATTGAATCCAAACAAAATATAAGTACATAATAAAAAACTTCACAAATCATAATAATTCGCCAAATTCCATA
GGTTGAAACATAACACACAATAATTTAAGAATTTTATTCATTCTTGACGGGGGAATACCAATCGGGGTAAACCC
ACGAAGCGGAGTCGCCGGCTGGATCGAGCCGACTAAAATCTAGCCTGAGCGGGTCGACTCGCCTAATACGAC
GACTGGCACATGCTGGACCCAGACGAGTGGCGCCGACTAGAGGCCAAAGACGGCGACTTCAATACGAAGCCC
ACCAAATACTTGTAAACCCTAACTGGCTGTATATATAAAGCCACCAGGGGCACCCCCACGGGACATCCTCTTCT
ACATCTCATTAGATCTAGTCTCTCCCTCCGCCTACGGCGGCTCCCATGTAAACCAATCTATACTCATACTGAATT
GATAGCAGCACGTAGCGATCCTCCACCGAGGGGACGTGAACCTGGGTACATCGTGTGCCAATCTCGCTCCTGG
ATTCTCCTACGTCGCATTGCTCCGTCTATGTAAGCTACCCCCAGTAGCATCTGCCGTGCAATACCACGACAATTC
TTATAATTATGATATAGAAAATACAAGATAAAAGGTAGAAAAGGGACCGTGTGTTGAGAGTTGAGACCCAATT
CAGTTTGATGCCTTTATATTGTCGAATAGGATGCCCTCAAAACCATGAGTCATCTTGTTCCTACACATCCTTTCAT
TCCGATACTAATCTCTACTCTTCTCACTTCTTATTCTTGTGATCTTGTTTTCTTACAGTGATAAGCTGCAAAATATC
TAGGTGCCCTATATAAATTTTAGCATGTAGCTCAATTGAACATAAGAAAATTTACAAAAGATTATTAGTACACTT
TTTTTCATGAATGTGTTCAAGAAACACCTTTGGAGTGTCTAATGGTTTCTAGCAGTTCTCGCCCTACCAGCAGTC
TTATAAATCGAATGGTTGAAGTGCCACCTTTTGGGTTCATAGTTTAACTTGTTCCACTTTAGTTGCATGCATTTCT
TTGAGCAAAGTAACAGATGATAGAATAAAAAATGATGCCATAATTTATCCCAATATTTTTATGGTTTTCTTCATT
ATTTTATCTCTCCCAGCACATACTGTAATCCTTGGAATCCTTTTGAAAGGCTAACGTTGATCTCAACTTTTCCCTT
TCATTGTAGAAGTAACAAATCAAGGTAAGCATTCACTTGTACCATTGTAAATTGCAGAAAGAGCGGTCTTATGC
CTTACTTAAACCTTTCACATATCTATTAAAAATTACTACAAATTAATACTCATAAAGAATTTGCAGGTCCCCAGC
ATACAATATGGTTGTACAAACAATTTCCATGTTAGAGAAACTGCCATCGATTGACTATGCATACTGCCATGCTT

ATATTAAATGCATAATCTAGGGCTTCATGATTTACATACCTTTTCCATCACGTGATGTGGAGAGTACTAAACTTG
ATTTGGAACATATGTAGTACATATTGCTTTAATTACCTGATGATGAACTACCTCAATACGTCTTACGTTTGAAGT
TTCATCAAAGATTTGGTGTGCATATCCTTCGACATGAGACATAGATCGAGCACCTTGCAAACAATCATTTGTCA
AGATATATTATTCAGTGTATGGTGTGAACTACTCCCTCCGTCCCAAAATAGATGTCTCATATTTGTCAAAATTTA
AATGAATCTAGACATTATTTAGTATATAGATTCATCCAAATTTTAACAAATCTAAGACATCTATTTTGGGATGGA
GGGAGTACATCTAATACTACGCTCGCTACTGTAGACCTGCAGCTCTTCAGAATTTGGTTTTAGATTTTTTGTAAA
TATAAAACACTTACATAAGAGCTTTGCTATGGGTATAGCCGTACTGTTCACAATTAGCACATAATCCTAGCAAG
GAGATGGGATTGCACCATGGGGGAGGAGATGCCGTCTTGCAGCGCTCTCGATCAGGCAGGCGACGATAAGA
GATGATGACCCTGTCTTTTTCCTTCTGGACGGATGTCAACGCACCGGACATCGGTCGGCCGGCGAACCCACGG
CGGTATATTTGCTATAGCTGGGCTCGGCGGCGCGCACGGAGCGAGGCCGAGATCAGGACTACGATTAACGGC
AAGAAGCCATGCAAAAGAAAAAACTATCCGTATGACATAGGGCTGTGTTTCCTGGAACCACACGGGCAGACTT
ACGTGTCAGAAATCGGTGTAATGTAACAGTAATTCTTGTAAAATCCTAGCCATCAACTATAGATCTAACTGTTA
AGATAAATCAGATGATGTGGCTTAACATGGTGTCTTCTAAAGACACCCTTATATTGCTTTTAGTATATACTAGG
AAAAAAGCCCGTGCGTTGCAGTGGGACTGACAAAATAATACTGTAACTTTTCTAAACAAAACTTGCAATGGAG
CTGATAAAACAGCTTAAAAAAACAATTTTAGAACACTTTTAAACAAAAAATATCCCATGACCTAAATTTTACCCC
CGTAAATACATCTAGCAAGCAGCAAAAATCATCAACTGGCTTTGATCAAACTTTTTTTGCAAACACATCTACTCC
CTCCGTTTAAAAATATAAGATGTTTTAGGTTTGTAAAGAAAATGTATCTAGACTATTTTTAGTGTGTAGGTTCAC
TAAGGTTTGGAGTAAATCTACACACTAAAAGTAGTCTAGATACAATCTCTTCACAAAACTAAAACATCTTATATT
TTGAAATAGAGGAAGTAGCAAGCAAGGGTAGGTAGCAGCAACCATATCGAGCAGCTAGCAGCAAAAGCATTT
GCAGCTGCGCGCTCTCACATGGCATAGAAGAAGTGCCGCGTCTCGCTTGGATCCTACAACACGTGAAGGCAAA
AAAGAAAATAAGACATCAGAAAACATACATACCGGGCTTAGACCAGTCAAAATCCCCAATTTTCTTCATGCTCT
CTCCTTGCTCTGCTGCCGGCCGCCTTCCAACCGTGCTACCTCCGGTCATGCCAACGCCTCCGCATCCCGGCTTCC
CTCCTGCGCCTCTACCATGCCGGCGACAACCACCGAACAAGACCGTGTGAGATATGAGTTCCTGATCTTTCTTA
AACAAAAATCGAACACCACTTTTGATTCGTCCATCTTCCATGAAGATAGAGTTAATAGCTTGAAGAATTACGAT
CTAGTACAATATTTTATCATGGAACCAATAAATTGATCAGGCTTGGAAGTTGGAATCAATTGACTTAGATTTGA
TTAATCAATGGGGACATGAAGCAGGCCTTAACCTGATTGGGAACTTGATTGGATGAATGGCCGAACGCCAACT
CCAGCAGGCTTGGGGGGACGTTGCCGTATACGCGTAGATGAAATCAGGACTATCCAGCGAACTTTTTCTCACC
TCGGCACCACGACCCCATCCGTTTCCGATGCCTCTACCCCACCGGCCACGCCTGCTGCTTGTGCCTGCATTTTTT
GTGCGGTCGCGATGGAGTAGTCACGAGAGAAGTCTTGCTCTTGCTTCGGGGGCGGTCTGCGCGACCCTGCCC
GTGCACGCCTCCTAGCCGAAGTCTTGGTCTGAGCTCGGTATGGAGATCGATGGGTGTTGCGCTACGCCTCCCC
CTCGCCCGCAACCGAGCCCAGAAGCACGCCTCCGCGCGCAGACACCACCACGGCCTAGTCCCTTTCCTCCGGC
GACGGAATAACGCCCAATTCGCCCCGGATTTTTGCCAGCAGTTGCGTCGTAGAGCTCCCCTCTTGAAGCCCCGC
CCCGAGCCCTTCCCCAGATCCTGGGTACTCCACCATCGTGAGATCCTCCGTCCCTCGATCTTCTTTTCCCCCTGCT
GCAATTGATCTAGATTCGATTTGGTTTCTCTACGACTTTTGAAAATCCTCTCCATCCTTGCGCGCATCCCGGCCA
TCTCCGTTCCCTTGGCCGGAGGAGGGCAGAGGATTTGATCGCCCGAGATTGCAGGGTGGCGGCGGTCAACAA
GATCTGTTTTCTTTTCCAGAAGTGGTGCCCGTGAGAAGGAGGCGCCGGGGCTTGCAGACGATGCTGACATCTA
GATGCGATGCGATGAGCTGATCCGGGACATCGAACTTGATGGCGGTGCAGTCCCGCAAGGTCGTGGCCGGTG
GGAGAGCCAGCTGCAGCCATCGTTACCATCTACTCGGTGACATCTATGCGTGTCGCCTCTCTCTCTAGATAGAA
GCAATGCACTGAGCTGATGCAATGAGCTGATCCCGGACATCCAACTACAAGAAACACTCCAGTTAGGGTAGAA
CTCTGCTTAGAGGGATGTATGTATATGGCGTGTCCTGTTGGCCGGATCAAAGCTTTTTTTTTCTTGAACGTGGA
CTCCGTCTGTCTCAGCTCCGAAACATAGTTTCTTTACTCATCGATCCACGTAGCTTTGTCCGACTGTTTTTTTACT
TATCGGTGGCATTGGTTGTAATATGTAATTTGGTGGGAGGGTAAAACGGTCCGAAAAAGTGTAGGTACTACAA
AAAACACTCCTTTCTTTATTATTAGGTAGGTATAGATATAGATATATATATATAGATAGATATATAATAGATAGA
TTAGTATTCCGTTAGGGAAGCCTTGTCAAAAAAAAAGTATTCCGTTAGGAATGACACACATACACATCAGCATA
TTTATGGCACTCCAAATTAACATCACAACTGTATTGTGTAGGAACAAAAATCATGGGCATGTAATGATGTAGGG
AACCAAACCGGTGGTGCTTGTCACACGAAATCTCAAGCTCAAGATGACGCCAATGGAACGAGGAGATGGTAG
CAGAAGAAACAAGGTTGGCCAGGCGGGCATGAACGCTGGACGTACTCTTATTCTTCTTCATCTCATATACACAA
AGGAAAAAAAAATAACGATGAGTTAGAGGAGGAGCCAAATGAAAATCGCTTTTAATATCGCTCGTGGGTACTCT

CTCTTGCGCGTCTCCCCCTCCCCTGCTACAGTAGTTGAAGCATTCCAGTACAGATCCGGCCCGAACCAAGGCCA
CTCTCGCTGGCGCCGCCGGTGATGGTTGGCTAAGGGGAGGTGCCGGCGGTAGTTTTAGGTGTAGATGTTTCCC
CTTAGTGGAGTTTTGGAGCTGGACTCTGGATCTTTGTGGCAAGATCTAGGGCGTTGAGTTCTTCTGCTTGCGGT
CGTGGCTTCTCTGGTTGAAGCACGATGGCGAGGGAGGATTCGCGGTCTCCGCAAATAAATTTGTGTGTCGTCG
GGGTGTGGTGGTGCTGTGCTTCTTTTCCTCTCCTGGCCGGCTGTGGAAGCGGGGGGAGCGGCGCAGGGTGTT
GTTGCGGTTTGTTTTTCCCGGTCGGCCGTGGAGGCGAGGAGGAAAAGCGTCGCTCCACTTCTTCATCACATCG
ACGGCGGTTGGAGTCGAGTGTTTGGGCCCGTTTCTCTGCTTCTTCTCTGGCTTGACGCCATGGAGGCGAGATA
CACAGAGGAACGCCGTCTGGGGATGGCGACAGAAGCTCACCGGCGGAGTTCATCCATGAAGACAGAGGTGC
CGACGTGATTTCTGTCCAGAAGAGGCCTCTCCGCGCTGTGGTGTGGGGCTGGCACCAGAGCATCTTCTTCCTC
GGCAGACAAGCCGATACGGAGGTCCTCTGATCTCAAGGCGGCGCTCATCGACGACGACAAACCAAGTGACGT
AGTCCCCGGTGCCGCCGGCGGCGGCTGTTCTTCGATATCTCGTTCGAGTTGTGGCAGTGAAGGACCTGATTTC
TTTTTATGTTTTTTGATTAGGGTCCTTCATGTAATATTTTAGGACTGTTTTATTTCCTCTGTGTTTGTAAGGGTCAT
GCTTCTAAAACTGCCATATGAGTAATACAGCTTCTGGGGCCTTCGGGTCCAATCCCTTCCCCGCGGTCTCTCGCT
CGTGGGCCTAGCTAGTAGTATGTGCAAAAACATCATTTGGGGAAGTGTTTTGAACTGGGCTAATATGCAGCAG
GCCTACCTCTTCGAGCGTGGTGCACATACACAACATCCAATTTGTCAAAAGACATCAACGCCAAAATTCAATGG
GAAATGATACGTGTCCTCCGGGATCCCGCACATCCTATCCCCGGTTCGTTGCGTGGCTTTTACGTGGGTGGGCC
TCCGTACGACTTATCTTCTTTCTCACCGCACAAACTCTGCAAATCGCCGTCGTTACCATGCGCACCTCCACCAAA
ACGGTGATTCTGGTGTGCATGCACGGGCCCACCCTCTCTCTCCTGTAGCTTTATGGAAAGATGTGTTAGAATAT
TATTTGCTACTACTTTTAGTTCTAAAGTTTAAAACACTTATAAAATTCGAACCAAAATTCAAATTTAAAAAATGTT
TGCATATTTGAGATCCTATTGAAGAGAGCTTCAAAATAGGACTAATATTAAATATATTTTGACAATATTTAAAAA
TCAACTTTTAAAACATGATAAAACATTCATAAACTATATGAAACATGGTGATCTTTTATTGTTGAAACAATCTGT
TTAATTGTGTTTCAAACTCCGCTCATCATAGTTAGAAAAAATTTGACAAAATTTTTTGAGATAAAAGAATTAAAT
ATCTAAATGAAAAATAAGAAAAAAATACTGAATTTCGAGTTTAGAATATAAATGTAACAAATAGTCGTGCATTA
AAATAA

>chr6A:314709415-314711457

CCTTTATTTTATTTGTATTATCTTTATTCCATTTTTTTATGGATTTTAAATCAAGGTTTAAATTTTCTTCCAAGTAAA
TTTCTTGGTATTTACTATTTAGGCAATTTGTATTTATTTAAATAAAATTGGATATTGTTCAATCCTTATGATTTTAT
TTAATTAATCATTTTCTTCCTTTTTATTATTCAATAGACTTTCATTCTATTTGTTTATGTATTTGGCCAAGATTTAAT
TTGGCTTTTAAATAATCCTTATTTATTTATTTATTTATTTATTTATCTTATAAAATCCATATCTTTTTATTTATAGAT
CCAAATTTGGTGATTCCAATTTGTATATTTTTATTTGATATTTGTTTATCCTTTTTTAATCCTTTTAGGATTACTTG
CTCACAATTTGGTATGGATTTTTAGCTAGGAGCTTTATTAGCTCTGATATTTATTTTCTTTACCTTATTAAATTCAT
AAGTTTTTATCTATGGCTCCAAATTAAATGAAACCAAGTTCTATGGTTTTTATAATATTTTTATTTATCATTTTTGA
TCATTTATAAATGTTTGTTGACAAATTTTTATATGGAACAAATCCATAGTCTTAGAGAGATTTTGGAAATGATTT
CTTTTATTAGAGTTTAAGGGTCTTGGTTTAACTTTTCCAAATGTTTAAGTTTCAAGATACATTCATTGATGAATGC
ATTTCACTTTATTGAAATTTTTTGGATTCCTTTTTATTAAAATTTTGTTCCAGAAGGGTTTTGATGTTTACATTTAG
AAGCAAACAGTTTCTTTTCACCCTAAATTTCAACAATTATTTCTTTTGTTTATTTATTTTCACATTTAAAATTTTGG
AAAATTTTGGGATGTGATAGACTCTCCCCCTTAAAATAAACCTAGTCCTCTAAGTTCGTATACTCTCACATTTCG
GAGTAAGGAATTGTCTTGCATTGATTATGATATGTCTCAACTCCATGGTAACCTTAAATGGAAATAATTGTAGT
ATTCCATATCCAGATCGTTTTCTTGTATTCTCATCTTACTTGGAATTCTCCAACTTGTTGTCTTCAATACTCCAAAG
TTACTTTTACTATCATCCCTTAGGGATATGTTCTCATTATGGAATCCTTAAATTGGAGTTTCTTCTTTAGTTCATTT
CCCTGATTAGGTATTCTATTCCAACTCCTTCATTTGATCATCGAACTTCCTTAATATATTGTATAAGGTCTAGGAA
GTCTCACTAAAACAGGTTTTAAATTTCTTTTAGCCAATAGTTATAGTCAAAAGACTAATCTTTGATTTATCATTGT
ATGAGTTATACTGGTAGTCCTTCCGAAGATTGGATTTGAACTCATTTAGTCCATACCCATCGAAATTTCTTGTTT
CACTATACATATCCAATTGGATGTCTGTTATTACTTCTTAAGAATTACTTAAGAATTGTTTCCTTTAGAATTATTC
TATTAGGGTTAATCTATTTTATCACCCTTAAGCTTTTGTCTTAGCAAGCTTTGGATAATGCTTTCTTTATCACCTT
AAGTACTTCTTCAACTACCATTTAATAATTGCCTTCAGGTTTGGGTACTACTTCCTTGAAGTAAACTTCGATCTTT
CATCCAGACATACTTCCGAAAGTACTTTAATAATGATGACTTCTTTTAACCTTATGAGAATAAGGATTAATGTTA

TTGTCGGGTAGTCCTTCTTCTGCAACCTTTATTCGGAAAGTCAGATAATGATACTTTCATTTCTAATGTTTACTCT
CCCTCTTAGGGTTTACTAATAGTCTTCAAACTGTCTATATAGTAACCATTGGCTTTATAACTAGAATGTTGGTCTT
TGTTCAGCTTATACTTTACCTAATTAGGGTCTTATGAAAGATTGTACGGTGTATCCACATAATTGTGGATCTTCG
AAGCGAATCTTTCAGCATTATTTGTCTTACTTACTATTTGGACACTTTCACTTTGATAGGTCAAACTTCGCGAAG
CGCCTCGGAAGATGGTCGTTTTAAGGTTTATACTCCATGTATTTCTTAACTTAGGCCAAG

>chr6A:314737354-314738657

TTGATGACAAAATGGACTATCTTTATTAGTACATGGCACATACATAGTACCATTATTATTACAGATAAAACGGG
CGCCTTTAAGACATCCATGGATGAGGCTATTTATTGGAGGCAGATCCATACGGAGCACTACGACATGCACGAT
GGTCGACGGTGTCTACCACATTTCATCGGAGAGGAGCGCGGCTCCGGTGCATGGCCCGCTCTGGCAGCCGGC
TCCACAGTAGTCACCTCCGAGACCACAGTATCCCCACTTGCTGCAGCATTGGTTGCCCGGACACGGACTGCCGC
TGCTGCACGCGTTGGGCACAGCATCTTGATTGAGCTTGGCTCCGGTGCATGGTCCGCTCTGGCAGCCGGACCC
GCAATAGTCGCCGCCGAGGCCGCAATACCCCCACTTGCTGCAACATTGGTTGCCGGGGCAGGGACTGCTGCTA
CTGCACGCGTTGCTGGACACACCATTTTCATTCAGCTTGCCTCCGGAGCACGGGCCGCTCTGGCAGCCGGCACC
ACAGTAGTCAATGGTTGTGCCACAGTAACCCCACTGGCTGCAGCATTCGCCGCCCGGGCACGGACTGCTGCTG
CTGCATGCGTTGACGGGCACATCTTCATTGATCTTGGATCCAGTGCATGGACCGCTCTGGCAGCCGGAGCCGC
AGTAGTCGCCGCCGAGGCCGCAGTACCCCCACTTGCTGCAGCATTGGTTGCCGGGACACGGACTGCTGCTGCT
GCAAGCGTTGCCAGGAACGACATTTCCATCGACGTTGGCTCTTTTACCATAGCAGGGGCCGCTCTGGCAGCCA
GCCCCGCAGTAGTCGCCACCGAGGCCACAGTACCCGTACTTGCTGCAACATTGGTTGCCCGGGCACGGACTGC
TGCCGCTGCAGCCCAGTGCGGTCGCTGTTGCACAGGCAAGGACCAGCAGGCCCAATGCGAGAGCCTTCATGG
CCATGTTCGAAGCTCTCACTCGCAGTGGATGGATCTCGAAAGGTTTTCTTCTTTAGCTTACCTTGTTTGGTGGAT
GAGAAAGCTTCCGATGATGAAGTATGAAGCTTGCGAGGTGCCATTATATAGGCATGCGGTACGTCCGTGGTG
CCTCCTCTAGTGAACCTGTGGACAAATGTTAGCGGCTTTTAGTCACACACGTAGATGTGTCAGTCATAGACAAA
TATTGTTTGTTTCAAAGCAGCTTTTGGTTGTGTTGGGTTGTAGCAATAAAATGGCTTTAAATCACTTTTGTTTGT
TGTATTAGTATTCTGTTAGGGAATATGTCAAGGTATTCTGTTAGGGAATGACACACA

>chr6A:394951960-394953960

CTTAGGTGAAGCCCTGCAGGATTTTCCCTCTACTTCCGACACCACGTCGTTGTGCTGCCGGAATCGCGAGGGG
AGAAATACAACCTCCGCTGCCTGCTGGAACAAGGGAGAGGAAGGTCTTCATCGAGTACCGAACGTGTGACCG
ACTGCGGAAGCGCTACCTAAACGCAGCGCCGAAAGGATCGTCTACACGATCTTGAGATCGGCAAGTGTACGA
CTACATCACCCACAAGAATCTGCTCTCGTTAACCACTTTAGAATCTTCGAGGGTGAGTGCTCTAATCAACTCGTT
GCTACAATCTAGTAGATAGATCTTGGCTTGTTTGTGTGCATGCAATAGAATTTTTTTGTTTTCCATGCTACGAAC
CCCTACAGAGATTGCCATACATTTCACCTCCACTGTCCTAGAACAACCCATCGAGGTCCCAGCCTTCCTGCAGCT
TCTGCACGAGTCTGCTATCAATGCGGGTATGTTGGTGTTGGGGAGCAGTGTGGTCAGACGTCGAAGGTGAGA
TTGCTAGCTACCGGGGCTCCCCCTTTTATTTGCGCACACAATAGGGGCCACAAACCATGAGTTGGTTAGCTACG
CTCCCGATCAGGGCGTTCTGTTAGATACGAGATTGAGGGTCGAACGATTGCTTCATGAGCCTCCTATTATCCTT
TTCTGAAAATTTTGTTGACTTGATTTTAAACCGTGTAGATCAACCAACATTCTCCCCCTTAACCGTTAGGTTTAAA
CTTCATTTTCCCATTCTATGTGGAAATAATACGCCAAAGCAAGGTGTTACAACAACCAATAAAATGTCGCTGAA
CTCAACACTTATGGTATACTAGCCCATTTCAGAATGGATAACATTTTACTTGATGGGAGTGGTTCTTATAGTGAT
CCCTTTAATCCAGAATCATAAGGCTTTTAGTAATCTCATGCCGACAACATGCTCGTGAAAAATACATATGGTAA
GCCTTTCATTCGCAGATCCACAAGAATACAAGTGGTTCTTATATGTGTAACATCAATTGTATGATGCTAGATTCT
GTGTTTCACAATATGAAACTTTACGTCAATATGCTTGGCAAAAACACTCGACTTATTGTTACTCGCATAAAAAAC
TATGGGTTATGATCGCGGTATAATACAAGTGGTCTTGAGATGCTGTCAACCACTTGTAGCCCGGAAATGAAAT
TCTTTAGCCATACATCCTGCCGAGTGGCTTCGTAACATGCTATAAATTCTACTTGCATCATACATGAAGCAAATA
TTGTTTGCCTGAAGCTTTTCCACGATATGGCTCCTCCAGTAAGTATGAACACATAACCTGATGTGGATTTATTAA
TGTCCACATAACCGGCATAATCAGCATATGAATAGCCAATAACCTTTATCAAACTTTCTATACGTAAGCATGTAG
TCTTTTATGTCTTGCATATAACGCAAGACTTTTTTTTCCAGCGGATAGTCCTGGATTTAATTGGAATCTACCAAG
TACTAACCATCCCGGTTATAAAAGTCAAGTCAAGGTGCGTACAAACTTGTACATACTTGTGAGAAAAAAATATAA

TTTACTTTAAAAGAAAAGAAAAAAGATCTAATTTATGGGAATTCAAATCGGGCAGTGGGAGCACACCCATGTC
CAGAGTTATTCCTAGTATAAAAAATTCTAAAAATTTGAGACATGTACGTCCATTTCATACGTGCACATGTAAAAC
TTTGTGGAAAAAATGACATTTTGTGTCATGTGTGAAAGAAAAACAAACTTGGTGCTCCAAAAATCGGTTTTAAC
ATTGTTTCTTTATTATTTTTGCGGAGGACAAAGGACGTGTCCTCTCTTCGTGAACAATGCATGCATGAAAAGCG
TGAATATGTAAAACGTCACAAAACCATATCTCAATTTTTCTTTTTTGAATTTTTTGAATGTGATTCATGATTTTAG
TGTTCACATTGAGAGAATATGCTCCAGGGGAGACAAATGTCTGGCCGACGATGAAGCTGCCAAGCAAAGC

>chr6C:16614873-16617067

AATCAACTCAATACCAGGCAAGACAATCATGGGACGAAAGTTACAATGGTCTGGAAGAGAATTTTGTTTGTTG
GAGACAAGGAGAAACATTGAAAACCCCAAACGTGCCGACATGTACAACACACGTGCTGACAGAATAACACGT
CTCAACAACATGAATTTTCCCATCCTTACCCTCGTGCACATGAGTGCAACAAGAGTAAATCTATACCATATACAT
ACTACATTCAACATGCTATATTATTGTGTATATATTCTAATATTGTGTAGAACGCCGTTCTTTCACCATTCTGGAA
TATGAACACACACAGTGTCATCTACATGATCCAAGGACATGCTCGAGTTCATGTTGTCAATAACCATGGTCATG
CCTTATTCAATGATGTTTTTCGTCAAGGATAGCTGATAAACATACCACAACACTATGTTGTTCTCAAGAAGGTG
GAGTGTGAATGATGCCAATACATCTCATTCAAGACCAAGTCAAACTCCTTGGTTAGCCACATCGCAGGAAAGA
GTTCCATCCTACGTGTCTTGCATGTTAAGGTCCTCGACAATGCATACCACATTCCCAAACAGGAAGCCCAAAAC
CTCAAAAACAACCAGGGAGAAGAATTTAGTGTGTATTCACTCCTAAATCTACGCAGACGGGCTTCCGGAGTTG
TCCAGAGGACGAGGATGAGTCATCTTTGACTAATAAGGCATCCTAGTGAATTAGTGGGTGCGAGGAGGACCA
ACATAGTAAATAAAGACTTGGAAAGTGTGTAAGTTTTATCTGAACAATTTCATATCCTTTTGTTTCGTGTCTTGT
GCCTTATCCTCTGACTATTCCTCTTTTCTATCTCCATCGATTGGCTGCCGTTTTGAACCTCATGCTCACGCATATC
GATGAGACATATGGGCCAATTTTTCAGATCCTACCTATAGCTCCAGTCTTGCTAATTCTCATTTGCTAGAATTAA
AGCTTCTATGTCAAAGTTTTGCAGCTTAAAAAACCGCAATTTCGGACTAAAGGTTATGAGATCTTCTACATTGG
ACATGTGGCACTGCAACTCGATTCAGCGGCTCTTATCAGGCATATCTGACATGCCGAGCACGCGTTTAAGTTAC
TGAACTTTGGCACAGCCTCATGGATTCAGGCCTCTGACCAGTATACAACAATCAGATGCAAGCCCTTATTTAAA
CCAAAGCGTATCTGAACCCCATTAGAGCTGCTATTTGAATTCTACTATGCTCTCCCAGTGACGATGTACAAAGG
CCCTTCTAGATCCGGAGATGTGCTTGTCCTAAAACACAACTTGTTTTTGCTTGCTGGTTATTACTACTTCAGGAT
CTGCTGTGTGCTCTTTGAGGATTGTTCCTTTGCTCAAGACTGTTGGCAAGATATTTTCACAAAGCTAAAACATCT
TATATTTTGAAACGGAGGGAGTACATTATTACTACTGTAATATTAGTTCACAGAAACTGCCAATGGATTTTAGT
TACCGCTGCGTGCAGTCACGCTGACACTTGAGTTCTCATTTCAAACAGGACAGAAAAAAAATCATGATTTTTAG
TGAAAAACAGTTCAGAAAGTACTCTGAAGAAGTTACATACTGCTGCACTATAATTTAAACAAATTGTGACGTCA
CACGGTTCTCATAACAAACAAGACCAAAAAGTTCACAAACATGAGGTTTCCCAACTTGCACTTAAAATCTGGAG
ATAAGTGTCAAACCAAACAAAAAGTACAGTTTATAGAAAGTAAACTCGACAAGTTACCGGTCAATGTCTGAAC
CAAGTAAATAGGGCTGAAAGTAGAGAGCTCACAACGTGGGAATCCCATCTTCTGCTAAACTTGGGAAATTTTA
AGGAGATGGATTCACAGTACAAGTATCTCAATAAGATGAAGTGCAAAGTCCGACAGAGCCGCCACGACATTAT
TTGAAGCAAACAGAACTCATGCCCTTTCTATGTGGTTTTCACCCGGTTTTCCTCGGCTAAATGGCAAAAACTGCT
CTACTAAATGAATTGCAGAAACTTCTGGCATTTCGACATTCTCTCCAGAAAAAAAAACCCCGAATAACCTTCAA
ATGCAGTGGGACTGCCTCCACTGACCGACTAGTCTTGGTATTTGCCTCCATGTCTTAGGTTTAAATGCAGTTACT
GTACATATCACAAACAATGTCAGAACAAACTTGTACAAACTAACGTA

>chr6C:218012984-218014984

CTCAACAAGTTTATGGCTTTCGAATCTAGAGATCCCTTCCTCATCCGCCAAGTGAGTCCAGTTCCACAAGTATCC
TTATTCACCGACCACATTTATATTCTTGGTCTTTCATGCTGCTAATGTATCACCTTTCCTAACGGAAACGCAGATG
CGTGAAGAGCCATCCTTGACCGACTGGGATCGTTTTGCTCGGAGAGAATATATAAGATTGGCGATGGAAGAA
GATGGTGAAGATGCTTCCAATGCAAGTGGAGATGTGTGGGATGAGTCGCTTGAATCTCCTTTCTGAATTGAAG
GCATTCAGGAATGTAGCAACACCCAGCAGAGCATTAACATACAGCCCTAAAATATCGAAATGAATGTGTTGTG
GTGAAGTATTGAGCAACTATTTGATATCTTATCTCAACAGGAAGTACAGCTAAGGCAGTTGTGCACTTTTTGGC
ATCATCTAAAATTTGGCGTAGTATGTTGATGAGTGCTAATCCATGTCTAGAAAGATACTTCCATCCAGTTTGCTG
CACAGTTGCCAATTTTGATTCTTGACAGTGAGTTTCAGTGGCAACTGATTTCATGATTCCATCGGTTTGGCTGCC

ATTATTTGGTTATTCATGGCAACAAACGGTCGCAGGGCACCTTATGATTATTCTGAAGCCCCTGTGATCTGTGA
TCTGTGATCAGGTGGCAAAGCAAGGAAGTGCATGGTTTCAGATAGACGATGCAACCACCATATATATGTTCTG
ATTGTGCATTGGCTCTGGTTGAAACCACAACTTACATTTACATAGTGTTGGTTAGACATATGTTATCTTTTTCAA
TAAGGTCACCCTCGTCAAGAAGACCTTCACTCGTGTGTATTCTGTATCTATGTTTAGCTTGTAGTTACATGGACA
AATGAGTTATATTGCACAGCTTTGTGTTCCATTTTTGTTTTTGAAGGAAATATTGCACAGGATTTAATTGTTGTT
TTTGAAGGAAATATTGCACAGGATTTAATTGTTGTTTTTGTAATTCATAATTTCTTAAGACCTGGAACTTGCCAG
ACCAAAACTGTACAAATACTATCGCTGGATCCTGATACTGAGAGCAGATATACAATTTGAACTACCCTACTTTTC
TGCTGAATTGATGTGCATTTGTGCAGAAAGATAGAACGGCCAGGAGGCAAATCGTTTCTATGGCCTTGGCAAA
AGCAGTGCCACCCAGCAGTATGCAAATCAGCTGATGCTTTGACCAGTTTTATTTCTGCGGATCCAAAAGATTTG
CATCAAAGGGCTGCTTAACTAACACCTTCTGCTTTTGCTTTAACTAATTAATAACTTAATTAAAGCTTTCGTCGAC
CAGGCAATCTGTATGCAGATATATCTCATACTTGCATCGGTGCATTTTGAGGGAGAGAGACCGGCCTTAGTAG
TGGACGAGCTGCTGAGCTTACCTTGCTGTGATCATGCCTTTGCTTTCCTTGATACACCAATGATTGACTCCAGAA
AGAAAATAAAATAAAAGGGTGTCCTGTGCTGTGATCATGGCCATCTTTTGAAGCAAAACGAAAGAGCAAAAA
GAAGCAATCGAGCGTTCAATCATCTTTCCTGCAACGGTGGAGAGTAAGCAACGACCTCATTTAGAATTTATAAA
CACGTCCATGAACAGGAGAACAAGAGATTCCAGCAAACCAACAGCGAAACACTAGCTAGAAAATGAAGAGAA
AGACCGAGGAAGATCTTTATTTTGGTACAAAATTCTTTGCTAATTCTTTAGCTCTTTACACCAATCACAAGAAGC
ACGACTCGATTCGAACTCCACGGTGTACCACGATCTTATCAGCGATGGATCAGTTTATAAAATAACTCATTTTTC
TATCGATGCATCAATATGTGGGCTTTTGTGTTTCCTGTGGGAAAAACTGATTGTTCATCCCTCCTTATGATTCAC
ATAAATCATGCATGACGCGTCTCCTCGGGAGCTACAGGCCTTTCTCATCGCAGTGCAGCAGCACGAAGCTT

>chr6C:278329778-278331113

TGATCCGATTCCTTCAAACAAAAGTTGATTCAAGCTCACTAGAAGCCAAGCCAGTAAGAGGTACATCTTCTACA
ACAAAATCTCTCACTACACATACCTTGAACACTTCTCAGTCCCCGAATACTAAAGACCTCTTTCCCAGGGGATAG
AGAACACAAGACAGCTAACTTCATCTTGTACAGATCAAATGCTTGAGGACAATGGGTGATGCAGGGTGGCCTT
CAGTTCAGCTAAAACTAGCAACATACGCCTATGCCTTCGCTTGTACAGCCTGCATCTTCTGTTGCAGCCTGATTA
TCTGCATGGCAGAAACACGTTAACAAAAAGAAAATACCGAGTATTAGAGTTGACCGAATGCAGGTTCTGACAC
TATAGTATATGTAGTGAATGACCGTAACTGATGCAAAACGTAAGTGTGATTTAAACATAACTCATTGAATCTTT
TATGATGCTGACATCATATGATCATAGTCAAAGTGTTCATTTACACTCTTGCAAGGGTTCAGCATCAGAGAAAT
CAGCCACCTGTGCTTTCAAGGACGCCATAGATACTACTCATATTGGTTGTCATTACATCTTATATCATGGGATAG
AGGGAGTATATTAAACCTTTAGACCTCTGTTGTAACGGGGGAAATTTGCTGTTCCTGATGAACCGACTGCTGTG
AGATATCCTCCCTCACTTAATACACGTAACAATGAGTAGTAAGAGAATCCAAATAGATGTCTGTTCTCCTAACAT
GCTAGAAACCTTTATGGACAAACAGACAACCAACTCACTGAGCTAACAGAATCCAAATACATGTGCTGTCCAAT
TCCTGATTGAAGCTGACAACCAACTCATGGAACTAAGACTACTACTAAGCTATCACAAGTCAAGTATCCAATAG
ACCGCCTAGATTATACTCCCTCCGTTTCAAAATATAAGATGTTTTAGCTTTGTGAAGGGAGTGTATCTAGACCG
CTTCTAATGTCTAGGTTCATTTAGATTTGGAGTGAACCTAGACACTAAAAGGGGTCTAGATACACTCCCTTCAT
AAAGCTAAAACATCTTATATTTTGGAATGGAGGGAGTAGTAGTCAAGCAAAGCCAAACTGAATTAGCCTCTTA
AATCAAAAAAGCAATAGGTGTTTACAATTTAGTTCATGTGGACACCAGAACTTGTTACTAGGTCCATAGCAACT
GATGGTGGAGGTAACCCACTACAGACAATTTGGTTATGATTTTGCTAATGGCTTACATGTGAGCACCACAGGG
AACACTACACACATGAATTCTACATGACAAACCGCATTTGCTAAAGAAGACAAAGCTTGTCTAGCAGCTATGTG
CTT

>chr6C:85309901-85311901

TTTCTCATCCACCAAACTAGCTAAGGAAGAAAACCTTCCCGAGTTCCATCCACCGCGAGCTGAGAGCTTCCAGC
ATGATCATGAAGGCTCTCGCACTGGGCCTGCTGGTCCTTGCTTGTGCAGCAGCGACCGCACTCGGCTGCAGTG
GTGGCAGTCCGTGCCCGGGCAACCAATGTTGCAGCAAGTACGGGTACTGTGGCCTCGGCGGCGACTACTGTG
GGGCTGGCTGCCAGAGTGGTCCGTGCTATGGTAAAAGAGACAACGTCGATGGAAATGCCGTTCCCGGCAATG
CTTGCAGCAGCAGCAGTCTGTGTCCCAGCAACCAATGCTGCAGCAAGTGGGGGTATTGTGGCCTAGGCAGCG
ACTACTGCGGCTCCGGCTGCCAGAGTGGGCCGTGCACCGGAGCCAAGTTCAATGAAAACGTTGTGCCCGACA

ACGCGTGCAGCAGCAGCAGTCCGTGCCCCAGCAACCAATGCTGCAGCAAGTGGGGGTATTGTGGCCTTGGCG
GCGACTACTGCGGGTCCGGTTGTCAGAGCGGGCCGTGCACCGGAGCCATGCTCAATGAAGATGGTGTGCCCA
ACGCGTGTAGCAGCAGCAGTCCGTGCCCGGGCAACCAATGTTGCAGCAAGTGGGGATACTGCGGCCTCGGAG
GCGACTACTGTGGCGCCGGCTGCCAGGGCGGGCCGTGCACTGGAGCCGCGCTCCCCTCCGACGAAATGTGGT
AGACACCGTTGACCATCGTGCATGTCGTAGTGCCCCGTGTGGATCTACCTCCAATAAAATAGCCTGAGGCATGT
ATGTCTCAATGGCTCCCGTTTGTATGTGTAATAATGGTACTATGTGTGTGCCATTTAATAAAGGTGGATTCCGTT
TTGGTATCAAACATATGTACCGCACTGCTGTCACTGCATTAGTTTTCTGTAGAAGCATGCGTTGCGTGTTGCACT
GTTGTTTTACTGTTAAGCTGTTCTCTATATCGATCTAACTCAGCCTCACCAGCTATAGCTCTGGCCTAGTTTTTTC
CTTGACCCATGCAAGAGCCTTACATGTATTAAATTAGAATAGGGAAAGTCATAGAAATGGTCTTTCCCATGCGG
GACACGTGTACCGACTGTTCGACGGCACAGGGCCTCCAAATTAGAAGGCCCCCAACTTTATGTTAAACTACTA
GTATTCGGTGCTGCTAATCATCGATTTAACTTTATGGCCCAACTCCTGAACACCCTTCCTCTCTTCCTCTTTCCTCT
TCTATCTTAAGAGCATCTCCATTTATTCGGCGTTCTCGGGCCTAAATCTGGCGCTATTTAGTCCGGATGGATGTA
ACTTTGGGTCTGGAAACGGCCAGTTTCCTAGTCGCGTATCTAGGCTGAGTTCCCGGCTTCGACCAAGTTTTCAC
AAAGCACGGCAAAACTTTAGATGATTTCAGGAGATTGCATTTGATATAGGCGAGTTCATACATATTATGTTAAA
TTCGGACATATACCCGAATTTTGACTGCAATAGGTTAAGAAAAACTTAAAAAAAAACTGAAAACAGTAAATAA
AGTAGACGATGCCGATGACGGCGCACTGATTACATGCCTAATTGGCGGTAGAACAAGGTGTAGTCCGCGTCCT
GGGTCCCGTCGTCGTCGCTCGGCGGCTGCAGTGGCCGGGAGCTACCCTAGCCGGCGTCGCCGCTGCGCCGAG
GGCCCAGCCTCATTTTTGTCGTCGTCGTCGAGGACGACGAGTGGCGTCCTCTGTTAGGCCCTCTCTTGGCGAGA
GCTGCGAGTGGCATGGCGCCCGGCCTGCTCGTGCAGCCGTCGAGCCGTCGGATGCTCTCGGCCTCCTCCCACT
CCCACTCCGCCCTGGACCAGGCGCAGGCGTCGGGGGCCTGCATCGGCAGCACGTCGTTGAGCGATGTCTCCAT
CGCCATCTTCAGGAGGAAGGCATCCTTGTCGAAGGGCACCGCCCAGACCACCGGCTCCTTCTTCGGCATGTGC
AGCTTTGACCTTGCCGGCC

>chr6D:176722551-176724551

TTTTTAGAACCCGAATCTTATTTATTCTGCTGCTAATATCATGCAATATAGTACGTAGTACTATCAAAGGTCACTT
ATAGTAATAATGGCTAAAGGCAAAGCCTTATTCAGTCTACAAGGGACAACATGTACCAAACATCTGAAGATGG
CACGTCAAGCAAGCCTAGCGCTCGATCAACTACGGGGCTAGCAACTTCGATACAGAGCAGAACACCTGCCGCT
TATTTCTGGTGGTACCTCCCCTGCTCCACCCTCTCCTTGAACTTCTCCTTCTCGTCGCCGGATTCTCCGAGCACGG
CTTCCTTGGTCTCGTGCACCGTCTCCTTCGCGCCTTCGCTCACCTCGTCGGTCTTCTCCTTGGCCATTTTCAGAAA
CTCCTCTGCCGTTGTTCTCGCCTTCTCTCCGATTTCGCCGAGCGTGTCGCTGTCATCCTGTTTCTCTGTTGAGTGA
TACTGGCAAGATGGCAAGACAAAGTAAAGGTTATCAAACAGCAGGAACTATATGTGATGTTGGCAAGAACAT
TTACTGTACTATAAAATGCGCTGTCAGAAGTTACAGAGATCCATTTCAGGCTTTTCAGCAGTATGTAGTACCGT
GAGCTGAATTAGTACAAGTTGACAACTGCATACTCGAGCTACCTTTTTTTTTAGCCCGCACACTCGAGTTACTAC
TTGAGCATTTTTCGATATAATGTCTTGTGACCTGCTGATTGTTCTGCTTTCTTAACCAAAACCTGTTTTCCTAGTT
TTCTCATGATGAATTTCGTCCAGACATTTTGAGCACTCTACATCAGATCTCTCCCGTATACTGTATCTGTAATTGT
GTAGTATGCAAGAAGATGCCACTTTGACAGATTCACAACTACTAGTATAGATTGCTCTGTTACATGGGTCGCGG
AACACTGAACAAGTCATGAAATTCAGAAAGTTAAGGCATCAGTTGTGAAGAGCGAACTCACCCTTGGTAGCGG
AACGCTGGTGCCAAGTGCAGGCATGGCAGACGATTCACCCACCAAAGTCCTGGAGGGGAGACCGATCCCCCT
CCCACCATGAACAAGCAGCCTCTTCATCGATCTCCTACGAAGAAGTCGAACACAGCTTCTCAGGTCACTACTAC
TACTTGCCTTGGCACAGGTGAGGAAAATGGCTTCAGTGGTGAGACCGCAAGAAAGAGGATAGGGATGTGATG
TGACCAGGAGCCGCCATTTCCGCTATAAGAAGTTGGGAAGCGTGAAGACGGGACACGGAAGAGCCGACTAG
AGATCGCTACGTGGCGGGGGAGCATTGTAGCACGTAGCGACCTGGCATGCGAAGCTGTGCAGGGTGCAGGG
CTTTTGTGGCTTTTCAGATCGTGTGGCCTTTGGCTTTGCCCACTTCCTTGGGAAGCGTGGCTGTAGCTGATAAA
AAATGTTTACCGGGAGCGAGCAGGAATCCGATGAGCTGGTGCTCCACGTTGATGCGAGGGTGAGCGGGAGA
AGATACCACTAGGAAGGGAGGTAGGCGCCACAAACGGTACCCAAATACTTCCTTCGATCTATTTGGGTTTATCT
AGATTTACTATATCTTTAACATTGCAACGTTCACTTTACTATCAACAATAAATATTGAGAGAATCATGGCCGTCT
AACCTCTTACTAGTGGATTAGATCTTTCGCATGTTTGATAGGTAAAACTGGTCCTACGTCTTTGATTTTTTTACAA
ATGTACCCCTCTATACGTCCGCATTGTCAAAAAAAACCCTTCTAGATTTAATTGTCAAAACTAACCTGTCAAAAG

GTGAAAAAGCAAACACGTGGCACTTCACGCTGCTGCAGATGGCGGACCAAATATCCCGTCCTAGGGAGAGAC
GGAAGGGCCTTCCCGCCCTTGTTACTGGCGGAAGGTAGTGGGCCCATGTGGTATTCTGGCGGGAGGAACATG
CCGCCAGGCTGCTTGGCGGGAGGTTCGTTGACACTTGACAGCAGTGACGCACGTACAGGCCTAGCGTTCTGTC
GTGTGCTG

>chr6D:277359971-277361971

TTGACGACAAAACGAACTCCATCTTTATTGCATGGCACAGACATAGTACCAATTATTACACATACAAACATGCG
TCTTTGAGACATCCATGGATCAGACTATTTTATTGGAGGTAGATCCACACGGAGCACTACGACATGCACGATG
GTAGACAATGTCTACCACATTTCATCGGAGAGGAGCGCGGCTCCGGTGCACGGCCCACTCTGGCAGCCGGCG
CCACAGTAGTCGCCTCCGAGGCCGCAGTATCCCCACTTGCTGCAACATTGGTTGCCCGAGCAAGGACTGCTGC
TGCTGCACGCGTTGGGCACAACATCTTCATTGAGCTTGGCTCCAGTGCATGGCCCACTCTGGCAGCCAGACCCA
CAGTAGTCGCCACCGAGGCCACAATACCCCCACTTGCTGCAGCATTGGTTGCCGGGGCACGGACTGCTGCTGC
TGCACGCGTTTCCGGGCACATCATCTTGATTGATCTTGGCTCCGGTGCACGGCCCACTCTGGCAGCCGTCGCCG
CAGTAGTCGCCGCCGAGGCCGCAGTACCCCCACTTGCTGCAACATTGGTTGCCGGAACACGGACTGCTGCTGC
TGCAAGCGTTGCCGGGAATGACATTTCCATCGACGTTGGCTCTTTTACCATAGCAGGGGCCGCTCTGGCAGCC
GGCCCCGCAGTAGTCGCCGCCGAGGCCACAGTACCCGTACTTGCTGCAACATTGGTTGCCCGGGCACGGACTG
CCGCCGCTGCAGCTGAGGGCGGTCGTTGTTGCACATGCAAGGACCAGCAGACCCAATGCGAGAGCCTTCATG
GCCATGTTCGAAGCTCTCACTTGCAGTGGATGGATCTCGGAAGATTTTCTTTTTTAGCTTAGCTAGTTTGGTGG
ATGAGAAAGCTTCCGATGATGAAGTATGAAACTTGCGAGGTGCCATTATATAGGCATGCAGTACGTCCGCGGT
GCCTCCTCTATTGAACCCATGGACAAATATTGTTATCGGCTTTTAGTCACACACTTAGGCATGCCAGTCATAGAC
AAATATTGTTTGTTCCAAAGCAGCTTTTAGTTGTGTAGGGTTGTAGTAATAAAATGGCTTTAAATCATTTTTCTT
TGTTGGATTAGTATTCGGTTAGGGAACATGTCAAACAAAAGAAGGTATTCCGTTAGGGAATGACACACATACA
CATCAGCACATTTATGACACTGAAAATTAATATCACAACTGTATTGAGTATGAAAAAAATCATGCACATGCAGT
GGTGTAGGGAACCAAACCGGTGGCACGTGTCACACGAAAGCTCAAGCTCAAGACAATGACAATGGAATGAAG
AGATGGTAGCAGAAGAAACAAGATTGGCCAGGCGGGCATGAACGCTGGACGTACACTTAATCTTCTTCATCTT
GTATACGCAAAGCAAAAGAAAAGAGTGACGAGTTAGAGGAGGAGCTCGTGGGCCTAGCTAGTATAGTATGTG
CAAAAACATCATTTGGGAAAATGTTTTGAACTGGGCTAATATGCAGTAGGTTATCTTGTTAGGATCCTTCTGTT
GACTAGTGGAACCTGTTTGGTTTAGTCCCACCTTGCCTACGGGAAGAGGCCACACCCATCTTATAAGGGAGAT
ATTTCTTCCTCCATTCAGTCCGTAATTCTGGGATAAAGTGGACTCTTTTCTTATTGTTGGGCTGTGAACGCATGA
ACGTCTAGAACGTGGACTGAGTACTTGGCTAGCCAAGTGGACGCTAACATCTGGTATTAGAGCCAGCATCGTC
ATGCCTTTCTGAGGGGCTTCTTAGGGGAATGGGATATTAGGACTCATTTGTTGACTAGTGAAGGCTGTTTGGTT
TAGTCCCACCTTGTCTACGGGAGGAGGCCACATCAGCTTATAAGGGAGAGATTCCTTTCTCCCTTCAGTCCGGG
CTTTTGGGATGGAGTGGACTCTTTTCTTATTGTTGAGCTGTGAGCGCATGAATATCCAGAACATGGACTGAGTA
CTGGGCTAGCCAAGTGGA

>chr6D:277366628-277369106

ATGTTTGACGACAAAACGGACTATCTTTATTAGTACATGGCACATACATAGTACCATTATTATTACAGATAAAAC
GGGCGCCTTTAAGACATCCATGGATGAGGCTATTTATTGGAGGCAGATCCATACGGAGCACTACGACATGCAC
GATGGTGGACGGTGTCTACCACATTTCATCGGAGAGGAGCGCGGCTCCGGTGCATGGCCCGCTCTGGCAGCC
GGCGCCACAGTAGTCACCTCCGAGACCACAGTATCCCCACTTGCTGCAGCATTGGTTGCCCGGACACGGACTG
CTGCTGCTGCACGCGTTGGGCACAGCATCTTGATTGATCTTGGCTCCGGTGCATGGTCCGCTCTGGCAGCCGG
ACCCGCAATAGTCGCCGCCGAGGCCGCAATACCCCCACTTGCTGCAACATTGGTTGCCGGGGCACGGACTGCT
GCTGCTGCACGCGTTGCTAGACACACCATTTTCATTCAGCTTGCCTCCGGAGCACGGGCCGCTCTGGCAGCCG
GCACCACAGTAGTCAATGGTTGTGCCACAGTAACCCCACTGGCTGCAGCATTCGCCGCCCGGGCACGGACTGC
TGCTGCTGCATGCGTTGACGGGCACATCTTCATTGAGCTTGGTTCCAGTGCATGGACCGCTCTGGCAGCCGGA
GCCGCAGTAGTCGCCGCCGAGGCCGCAGTACCCCCACTTGCTGCAGCATTGGTTGCCGGGACACGGACTGCTG
CTGCTGCAAGCGTTGCCAGGAACGACATTTCCATCGACGTTGGCTCTTTTACCATAGCAGGGGCCGCTCTGGCA
GCCAGCCCCGCAGTAGTCACCACCGAGGCCACAGTACCCGTACTTGCTGCAACATTGGTTGCCCGGGCACGGA

CTGCTGCCGCTGCAGCCCAGTGCGGTCGCTGTTGCACAGGCAAGGACCAGCAGGCCCAATGCGAGAGCCTTC
ATGGCCATGTTCGAAGCTCTCACTCGCGGTGGATGGATCTCGAAAGGTTTTCTTCTTTAGCTTACCTTGTTTGGT
GGATGAGAAAGCTTCCGATGATGAAGTATGAAGCTTGCGAGGTGCCATTATATAGGCATGCGGTACGTCCGT
GGTGCCTCCTCTAGTGAACCTGTGGACAAATATTGTTAGCGGCTTTTAGTCACACACGTAGATGTGTCAGTCAT
AGACAAATATTGTTTGTTTCAAAGCAGCTTTTGGTTGTGTTGGGTTGTAGCAATAAAATGGCTTTAAATCACTTT
TGTTTGTTGTATTAGTATTCCGTTAGGGAATATGTCAAGGTATTCTGTTAGGGAATGACACACATACACATCGG
CACATTTATGGCACTCCAAATTAACATCGCAACTGTGTATTGAGTTGGAAAAAAAATCATGAGCATGTAGTGAA
GAAGAGAACCAACCTGTGGCGCGTGTCGCAGGAAAGCTCAAGCTCAAGACGACAATGGGTTGAGGAGATGG
TAGCAGAAGAAACAAGATTGGCCAGGCAGGCATGAACGATGGAGGTACACTTCTTCTTCTTCGTCTCGTATAC
GCAAAGGAAAAGAAAAGAGCGACGAGGTAGAGGAGGAGCCAAATGAAAATCGCTTTTCATCTTGCTCGCGG
GCCTAGCTAGTATGTGAAATTTTTTCCTTGGCCACATACGGCAGATTTGCTTTCGTGGGGAAAACCTTTTCGCG
ATGCAGAGTGCAGTTTGTGTTCAATGGCGTCACGTCCACTTTGTTTGAACTTACAATGGTTAACCTTTTTTTAAC
ATGCAAACAGTGAACCTTGGATGGTGATGAGACAGTAACCACACCACTTTACCTCAGATTAGATGAAACGGAC
TGAACATGGTAGCACATTAGATTTCTGTTATGATGTCACTAGGCTCCTCTCCAATTCGATACAAGTTCAGTACAA
TCTCCTCCTGATCACCCACAGCAAATGAGCGCTCCACTCGATGTTTGCCAGCTTGGTGCTTCATGTGTTGCTGTG
CCCGGAGCAGTTGTTGTTTCAGAAGAGAAGTTATGGCTTCACGCTCTTGTAACCACTAACCAGTAGTGCAAGTC
AGGTACTGAGAAAGAAACAATAGATGATATGCCATAGAGAGCTTCAAAGGGGGCACGACCCATGGCCGAGG
GCAAAGAAGTGCTATACTCTCCCTCCGTTCTAAAATAGATATCTTAATTTTTTAAAAAAATTAAGATAAATCTAT
ATATAATAAATAGTGTCTAGATTTATCTAAATTTTAAAAAGCTTGAGACATCTATTTTGGAATGGAGGGAGTAC
AAAAATTCAGCAAGCGACAACCAGCGAAACCACTTGGTGGGGAAATATTGTTAGTGGCTTTTAATCACACATA
TAGACAGTGTAGTCACAGACAAATACTGTTTGTTCCAAAGCAACTTTTATTTGTGTTGGGTTGTAGCAATAAAA
TCGCTTTAAATCACTTTCCTTTGTTGGATCAGCATTCCGTTAGGGAACATGTGAAA

>chr6D:283663038-283664882

ATGGCAACTACTAGTTTTCCATCAATGTTGTTTTACTTTTGCATCATTCTCTTGTTCCATGGATCCATGGCTCAGT
TGTTTGGACAGAGCTTTACTCCATGGCAAAGCTCTCGTCAAGGAGGTTTAAGTGGGTGCAGATTTGATAGGCT
ACAAGCATTTGAACCACTTCGACAAGTGAGGTCACAAGCAGGTATCACTGAGTACTTTGATAAGGAAAATGAG
CAATTTCGTTGTACCGGTGTATCCGTCATCCGTCGTGTGATCGAACCTCAAGGCCTCGTGCTACCTCAATACCAC
AACGCTCCTGCCTTGGTGTACATCCTTCAAGGTTGGTGTTTAATTGAATATAGCAATTGCATTGTTATACTACAC
TTATGAGTTTAGTCGTGCTAAATATTACCTAATTCATAAGTTTTTAATATTGAAACAAATATGTTTTCTTTTAGGT
AGAGGTTTCACAGGATTGACTTTTCCTGGATGTCCTGCGACCTTCCAACAACAATTCCAACCATTTGATCAAGG
CCAGTTTTCTCAAGGTCAAAGCCAAAGCCAAACTATTAAGGATGAGCACCAAAGAGTTCAACGCTTCAAACAA
GGAGACGTTATTGCGCTTCCGGCGGGCATTGTACACTGATGCTACAACGATGGTGATGCACCAATTGTAGCTG
TCTATGTCTTTGACGTAAACAACAACGCTAATCAGCTTGAACCTAGACAAAAGGTAACTATACAAATTAATCCA
CATAAAAGATATATAATTATTTACAATTAATCCAAATTTGATTTTTTAAGTATATATTATGGAGATATTAACAAA
CTTTGTTTAACACCATCCATATAAAATTTCAGGAATTCTTGTTGGCTGGTAACAACAAGAGAGAGCAACAATTT
GGAAACAACATATTTAGTGGATTCACTGTCCAACTTCTTAGCGAGGCCCTTGGTATAAGTCAACAAGCGGCAC
AAAGGATCCAAAGTCAAAATGACCAAAGAGGTGAGATAATTCGTGTGAGTCAAGGCCTGCAATTCTTGAAGCC
CATTGTGTCCCAACAAGTGTCAGTAGAGCAACAAGTCTACCAACCAATTCAAATTCAAGAAGGACAATCAACCC
AATACCAGGTAGGGCAATCAACCCCATATCAACAAGGACAATCAAGTCAATACCAGGAAGGACATCCATGGG
ACCGAAGTTTCAATGGTTTAGAGGAGAATTTTTGTTCATTGGAGGCAAGGAAAAACATTGAAAACCCCCAACA
TGCGGACATATACAACCCACGTGCTGGCAGGATAACACGGCTCAATAGCAAGAACTTCCCTGTCCTTAACATCG
TGCAAATGAGTGCTACAAAAGTAAATCTATACCAGGTATATGTGATACTACACTCTACACACTATATTATTTCCA
GATAGTCTAAGATTCATAACAACCGGTTAATAATATGATATTCGTAATTACTATTGCAGAATGCCATTCTTTCAC
CATTCTGGAACATTAATGCACACAGTGTCATCTACATGATTCAAGGTCATGCTCGAGTTCAAGTCGTCAACAAC
CATGGTCAGACCGTATTTAATGACATTCTTCGTCGAGGACAGTTGCTAATCATACCACAACACTTCGTTGTTCTC
AAGAAGGCAGAGCGTGAAGGATGCCAATACATTTCATTCAAGACTAATCCGAACTCCATGGTTAGTCACATCG

CAGGAAAGAGCTCAATCCTACGTGCCTTGCCTGTGGATGTCCTTGCCAATGCATACCGCATTTCTAGACTGGAA
GCCCGAAACCTTAAAAATAATCGGGGAGAAGAGTTCGGTGTATTCACACCTAAATTTACCCAAACGGG

>chr7A:11530658-11532958

ATGGCAACTACTAGTTTTCCATCAATGTTGTTTTACTTTTGTATTTTCCTCTTGTTCCATGGATCCATGGCTCAGT
TGTTTGGCCTGAGCTTTACTCCATGGCAAAGCTCTCGTCAAGGAGGTTTGAGTGGGTGCAGATTTGATAGGCT
ACAAGCATTTGAACCACTTCGACAAGTGAGGTCACAAGCAGGTATCACTGAGTACTTTGATGAGCAAAATGAG
CAATTTCGTTGTATCGGTGTATCTGTCATCCGCCGTGTAATCGAACCTCAAGGCCTAGTGCTATCTCAATACCAC
AACGCTCCTGCCTTGGTGTACATCCTTCAAGGTTAGTGTCTAAGTGAATATAGAATTGCATTGTCATACTACACT
TAGGAGTTTAGTTGTGCCAAATATTAACCCATTCATAAGTTTTTAATATTCAAACAAATATGTTTTATTTTAGGTA
GAGGTTTCATGGGATTAACTTTCCCTGGATGTCCTGCGACCTTCCAACAATAGTTCCAACCATTTGATCAATCCC
AGTTTGCTCAAGGTCAAAGCTAAAGCCAAACTATTAAGGATGAGCACCAAAGAGTTCAACGCTTCAATCAAGG
AGATGTTGTTGCACTTCCGGCAGGCATTGTGCACTGGTGCTACAACGATGGTGATGCACCGATTGTAGCTGTCT
ATGTCTTTGACGTAAACAACAACGCTAATCAGCTTGAACCTAGACAAAAGGTAGTTATACAAATTTATGCACAT
AATATATATATAATTGTTTACAAGTAATTTTTTTGTTAAGTATATATTATTGGGATATTAACGAACTTTGTTTTAC
TCCATCCATATAAATCGTCAGGAGTTCTTGTTGGCTGGTAACAACAAGAGAGAGCAACAGTTTGGAAACAACA
TATTCAGTGGATTAAGTGTCCAGCTTCTTGGTGAGGCCCTTGGTATAAGTCAACAAGCATCACAAAGGATCCAA
AGTCAAAATGACCAAAGAGGTGAGATAATTCGTGTGAGTCAAGGACTTCAATTCTTGAAGCCCATTGTGTCCC
AACAAGTACCAGTAGAGCAGCAAATCTACCAACCAATTCAAACTCAAGAAGGACAATCAACCCAATACCATGT
AGGACAATCAACCCAATACGAGGTAGGGCAATCAACCCCATATCAAGAAGGACAATCAGGTCAATACCAGAC
AGGACAGTCATGGCACCAAAGTTTTAATGGCTTGGAGGAAAACTTTTGTTCATCGGAGGCAAGAAAAAACATT
GAAAGCCCCTAACGTGCCGACACATATAACCCACGCGCTGGCAGGATAACACGTCTCAATAGCAAGAATTTCC
CCATCCTTAACATCGTGCAAATGAGTGCTACAAGAGTAAATCTATACCAGGTATACATGATATTGCACTCTATA
GACTCTCTTATTTTTAGATAGTCTAAGATACATAACCATCGGTTAATAATATGATCTTATAAATTACTATTGTAG
AATGCCATTCTTTCACCATTCTGGAATATTAATGCACACAGTGTCATCTACATGATACAAGGACATGCTCGAGTT
CAAGTCGTCAATAACAATGGTCAAACCGTATTCAATGACATTCTTCATCGAGGACAGCTGCTAATCATACCACA
ACACTTTGTTGTTATCAAGAAGGCAGAGCGTGACGGATGCCAATACATTTCATTCAAGACTAACCCAAACTCCA
TGGTTAGTCACGTCGCGGGAAAGAGCTCCATCCTACGCGCCTTGCCTGTGGATGTCCTCGCCAATGCATACCGC
ATTTCTAGACAGGAAGCTCGAAACCTCAAAAACAACCGGGGAGAAGAGTTCGGTGCTTTCACACCTAGACTTA
CCCAAACGGTCTTCCAGAGTTACCAAGGCATCGAGGAGGGGTCATCTTCGGCTGTTAGAGCATCCGAGTGAAT
GAGTGTAATGGGAACTAGTATAGTGAAATAAAGGCATCGCAAGTGTGCAACCTAGTGTCATATGAGTGCTTAT
CTGAATAAAAACTTTACCATGTTATATCCCTTGTTTGTGTCTTGTACTTTTCTTAAATTTATCTCTTGTGATTAATC
CCCCTTCCCTCTCTCCATCGATTTTCTACTGGTTTGTACCTCATGTTCATGCATATCGACGAGACATATGGGTCAC
TTGGACTACAATTTTTTTTCATGCACATATTTTGTGGTCCTTATATTTTCCAGGTTCTACATATAACTACAGTTTTT
CTTTAGTTCTTGGTTCCTAGAAAATTTTCATAAATCCGAGCACCTAAAATTATGAATCACATAGATTACAAGTT

>chr7A:16061896-16064813

CTTTTTTTTCTTTTTTTTTTCTTTTTGTTGGCATGCTCAGGCTGGAGCACAACTGCGGGCAGTGTCACAGGTTCAC
CGCTAATTACTTTTGAAGTGTTTCACCTTGGCCATGAATACAAACATGATGACAATTCGAAAAGCCAGCTAGAG
CCGGCGTCAGAATAATAAAACATCATACCTGCAAGGCTAGTATATATTCTTATCTGCAGCAGAGAAACGACATA
AAGAAAGCCAGTACTATGTTGTGTTAGGTATATCCTTCTAAAGATATAATGTGGTGCTGGGAATCAACAATGCT
TTGGTATCATTAGCCCGTTAATTATTATCAATCTGCATCGCTTGTTTGCCAAGCACACGCGACGACGTGTCACTC
ACTTGATTCGTCCGACATGTTCAGGTGTCATGCATCTTAATCATTGTCAGCCAAAGATTTAGTTCCACGCCTACG
GTGATCTGGTCTGAAATTAAATAACGGCGAGAGAAGACATATTTTGCTTAGTGGTATGGTATGGGTGGATAAT
TTTATTTTAACCTAGACCGTCAAAGTCTAATCTAAAAGGGAGAACATGGTGTGACATCCCTAGATAACATACAA
GCACAAATATCATAACCAAGCTCCTCCCATTGATGTCATCGACACAGGTTCACTAGGCCTTGGCTGCAAAGCGA
GGCCCACATGTTTGTGTGGTTGTGCCAACATGTCCTTGACTCCTTGTATACTACAGCAGTAAAATACGTCAAGC
CCTCACATTCTCAGAAGATATACGGGGATAGTTGACATTGTATGTGATGTTTACTACTTGATGTGCCAATGACA

TGGACAACGTGGAGCATAAAAAATGGCTAATGAAATTTGCAAATCGAAGATGTATACAAATTTCCAACAACGA
AACACATTCGGAACACTCGCAAGCTTTTATTATATTCATATCATGATGAGGCGGACATGCAGGAACATCAAAGT
GCTACACACTTATCTTGATGTCTTTATTTTACTTTACTAGATTGTCCTCCCATTACACTAGTTCACCAATCCCGTTT
GAGCTCTTGTGAAGCCACTTTGAGAAAACTTAGGAGTGAATACACCAAACTCCTCTCCTCTGTTGTATTTGAGG
TTTCGGGCTTCTTGCTTGGATATGTGGTATGCATTAGCGATGACATCAATAGGAAGGGCACGGAGAATTGAGT
TCTTCCCGGCGATGTGGCTAACCATGGAGTTTGCGTTGGTCTTGAATGAAATGTATTGGCATCCTTCGCGTTGT
GCCTTCTTCAAAACAACATAGTTCTGTGGTATGATTAATAGTTGTCCCGGGCGAAGAATGTCGTTGAACACATT
TTGTCCATGATTGTTAACAACTTGAACCCGTGTGTGGCCTTGGATCACGTACATCACACTATGAGCATTAATGTT
CCAGAATGGTGAAAGAATGGCGTTCTGCAATTAATCAAATTGAGTAAGTAAATGGAAATAGAATTAGACTTCT
ATTATTAAGCTTACAAGATATATAGATAAGAATGTGTTGAAATGTAACATCGTAAGTACCTGATAGAGGTTTAC
TCTTGTAGCACTCATTTGCACAGTGTTAAGAATGGGGAACTTTTGGCTGTTGACTTGTGTTATCCTACCAGCAC
GTGGGTTGTATGTGTCAGCACGTGTAGGATTTTCAATGTTCATTGTTGGCTTATGATCACAAAAGTTCTCCTCCA
AACCATTCCAACGGCCACTAGTTGATTGCTCTACCTGAGATTGCCATGTCTGAGACGACTCTATTTGAGACTGA
CCAACCTGAGGTTGTGTTGCTTGAGATTGCTCTTCTTGATACGACGGTTTCTCTTGTTGTGGTATCACAATTGGT
TGCAGAATTTGAAGCGCACGATTCACGCGGATTATCTCACCTCTTTGGTCATTTTGATTCTGAAGTCTTTGAGAT
GTCTGTTCACTTATACCAAGGGCTTCACTAAGCAACTGTAAATTGAATCCATGGAATATGTTTTGACTCACATAC
TGTTGTAGTGTCCTACTACCTCCAGCCAACAAAAATTCCTGCAATTTCATCAAGTAATAAATATATCAAACAATC
TTAGATATTGGTCAAAAAGGCTAAACCTAGTAGATTAGTCGAAAAGACTATCAATCCTAGAGATTGCTTTAGAT
ACTCTCAATTACCTTTAGTCTAGGTTCAAGCTGGTTGGCATTGTTGTTTATGTCAAAAACATAGAGAGCTACAAT
CGGCACATGGCCATGATTATAGAACCAATGTGCAATGCCAGCTGGCAACGCAACAACATCTCCTTGTTTAATGT
GATGAACTTTCTGGTGCTCATCTCTAAACTTTTGGCTTTGGCTCTGACCCTCAAATTGCTCTTGATCAAATTGTCC
GAACTGTTGTTGGAAGGTCTCCGGGCAACCAGGGAATGTCAGCCCTGCAGTACCCGTGCCTACAAGAAAATAA
GTTCAATTCAATATTAAATATTCCTAATACTTTTGTAATATTTTTCACATAGAAATCTAAAGTATATAATCTAAAG
CTATAACCACTCTATTAAGTTCATAAACCAACCTTGTAGGATGTATACCAAGGCGGGAGCGTTGTGGTATCGAG
GTAACAAAAGGCCTTGAGGCTCGATGACACGACGAATGACAGATAGACCAGCACAACGAAATTGGTCATTTTG
CTCCTCAAAATACTCAGTGAGGCCTGCTTCGGACCTCACTTGTCGAAGTGGTTCAAATGCTTGTAGCCTATCGA
ATGTGCATCCCCTAGCACTTCCTTGTCGAGAGCTTTGCCATGGTTGACTCTGGCCAAATACCTGAGCCATGGAT
CCATGGAACAAGAGGAAAATGCAAAAGCAAGATAAGAGCGATG

>chr7A:16066731-16068731

TATTTTAGTTTTTTTTCCGAGATTCATGTAAACCTACCTGCTGAAAACATCTAGATATATGTGTTTTAGAAAAGA
ATTAAAACATCTTCTCTACTATATAATTTGTCTTACCGTGGTTTTCATATGCCGGAGATTTTCGTACGTCGTCCGC
CCCAAGCCTACCATCGCGATGGATAAGCTAAGTTGAGTTCGGACGCAAAAAAAAAGGCAAGTTGAGTTCGTTT
CGGTTTCCTACTTTTCCGTTCGCCCCGATCTAAAACAATTAACATCTCATGCGTGAGTCTGCCTTGGTCACATCA
GGCATCGTGCAAAGATCAGCCGCCTCATGGATCAGCATTTAGCCACAGACTGAGCCCAAGGTTTGGGTGATGA
TTTATCCGGTCCTTCTGCTCTCGCGATCCTGACGAGCAATTTTTCCTAAGCTGCGGCGCCTCCTGTGAGAAGAG
GCGGAGCGGACGCTAGCCGCCTCTTGCGTCGATCTGTGTCAGCGTTTATTGATGGGAGCGAGAACTCGAGAG
AGGGAGAGATCCAGGAGCAATGGCAAGCTTTAGACTTGCCAAGTCTGATCGGTTGTACGCTGCTACCGCTGCA
AAGTCTTTGCCCGGCGAGATTTTCGTCGTGGAGATGCAATGACGAGCGGGGCTGCCAGCCGGCGAGGATCTC
TGGGTGGCGTCAGGGTCCTAGGACGTCGTCGATGCGTGAGCTAGCTATCGCGACCTCAGGCTGTCGGCGACG
CTGGCCGAAGCATTCTCGGGGACGGCGGACAGCGCAAGAATAGCAGCGGGTACCCGCGGAGGATGTACTGG
TGGCCACTCCACCGGCGAGCGACCTCCCCCATCGAAGGCCTCCTCCCCGGCCTGGATCCCGCTGCTCTATGTGC
TTGCCATAACGGTGGCGCTGCCCTGAGAAGTCAACGACGAGCCATAGGTCGTGGTATGTGCCACGCGGGAAG
CCGACTTTCTTGGGCCATCATGCCTGCTCCTTGATCGGGTGAGTGACGCCAGTGCCGATAAGCCGGGCCGTGG
TCCATGTCCTGCAGTGCGTGTGCCGCGCGGGTGTCTACGATGGCCAAACGGCGGAGCCACAGTTCATGTTGAG
CCACGCCCACCGACTGGTAGCAGACCAGGATCCCGGCCGGTGCCGGCACCCGGCGTGCAGGCCGAGATGGA
GGGAAGGGGAGGCAAGGGAGTGAGGGAAGTGGTGCAGGACGGGAGGAGTAGAGGAGATGGAGTCCGACA
GCTGCTACTTTACAGGAGAGAGACAGAGAGGAGAGATTAAGAGACAACGACATATCAGGAGAGTTGGGGGA

GGTTGGCATCAGTAAAGTGTTTTTTTGGTTTCAGTTTATAGACTGGTTCATGGTGGCCTTTTACTTCCAACTGAT
AGAACACGCACAGGTGAAGCCCAGCCACAGCTGACATATAATCAAACGAAGTTTCATACATGACATCAACAAG
GACATTGAAACTTACATGTTTCACCGGGTCAGGACAATCTACACTGCATTCTTAATAAGTTAGTGTTTGCTATTA
TATGTAAGGCTGATATGTACCCAGTCATGTACACACTTCTTATTATGTGTTAACTTTAAACACACTAATCCTTGC
GAGATAACACACTTCTTATTAATGTTTGGCAAATATGAAAAATATGGAGTACTGATGGAAATTTTCCAATACAT
CTATTGTTTGGTGTTATCCTTTTCTTTTCACAATAATGCTTATGTACTATCTAATTTGGCCAACATGAACCCATGA
GAAATAGAAATCAGTCATGTGACATTTTAATTGGATATTTGAAATTCCTAGCCCATGTTAGAATTTAAGCCAAA
CTTGACAGCATTCAAAACATTTGGAGAAGAGGATTTATTTTGGGAGTAACGGGAAAGGAGGCTGGAGAGGCG
GGCGGCCGAACTGGCCGGCATCTGTCGCCAGCAGGCGGAGCAGGGCAAGCGGGAGCCGAGAGGAAGCGTA
GGCAGGCGGGTGAGCATCTTACGGGCG

>chr7A:16081296-16083296

TTTGTTTTTGTTTTTGTTCATTTGAATCAATGAATTCTAGACTTGCTTTGGATATTATGTTGTTGCAAAGTCTGGG
TTTACTTGGTATCATATTGGTATTAATATATTCCCTTTAACAAAAAAGTAGTTGTTGTTGAAGTCGTGTATAGAA
ATTATTTGATCCATTTCTAAATGAAGGCACACAACTGCCATTTTTTACTCTTAGATTAATCTACAAGTACCAGAA
GATCGAGCTAACCATTACATCATATACCATTACTAGCCTCTAGCAAGAAGCAGCTAAAATGTATCGATCCACGC
ATGCAACCTCTTGTGCTTGGGCTCAAGATGGTCTTACTTTCTCAAATGGGCTTTCTTTTATGGCCACTGTCCATA
GAAGACTAATTGCTAGTAAATACTGTTGAAAGTCAAATCGGTATGATGACTTGAATGAGTTAGTTATTTCGTGT
GTTGAGTGAGAGTACTGTACATGGCTGGTTACATCTAGATCACTTAACCGGTACTTGCATTGGTAAGTTGTATA
CAATTGGGGGGTTCTGAGATGATCAAGTCGCCAACAAGTCAAACACTTTCAGATATACACACTAGCCCTAGGT
GGATGCCAAATTTAAGGGTGATCACTTAAACTATGTTTTATTTCCTTGACCGGTGTCTATTGGTCAAAAACATG
ATTCCCTTTTGTCTCGTGTATGTCAATGCTAGAGTTTTGAGAGCACAATCTGCAACTTACAAGGGAAAGAAGCA
CTATATGAGCCAGAATCTTCTTTCTATTGTTGATTTTGATATGATGTTCACATATGTGCTAGATGGTTAAGAAGG
ATCATCCTATGATGCAACCATTCTTCCTAACATGTCTAGACTTGATGGGATCAACATCCCTAATGGGAAGTTCTA
CCTAAGAGATGCTGGATATGGTTATCGCCCTGGGATTCTTCCACCCTCTAGGTTAACCAGGTATCATCTGAACA
AGTTCACTGCAAGGAACGGGCCCCAGAATGCCAATGAACTCTTCAATACTAGGCACTCTAGCCTTAGGGTCAC
CATCAAGAGGGCATTCACTACTTTGAAGAATAGATTCAAGATTGTATGGTCAGAAGCCATCCCACCCTTTCCCC
ACCCACGTAAAGCTTGTTCTTACATGTTGTATTCTTCACAACTGGATCTTGGGGTTTAGCCATGATGACCTTGTG
CCGGATGAGGCAACTATTACGTCCAATGATAATGCTACTTGCCGTGGTGCCTGGCAATCTGACAATGAAGCTT
GGAAGACCAATAGGATAGAATGGGAAAGATCAACATGGAAAGATAGAGGTGACGCCAGTATTTGCGAGGAA
GAGAATGTTGAACTACCCCCTCCTTGACACGATTCATGAACCAACAATTAAAAAAATCTAGGACTACTTGTTTAT
TCGTTGTAGGTAGGGCTGATCTAATGAACTATCATCTATAGTAGGTAGGGGTTGTGATGATGAACAACTTGAG
TAGTACCTTAGGGAATCTCCACCGGGTCGATCCAACCTGTCCGAGCGCGTCCGCGCGGACGCGCGGGACGTG
CGCGGTGTACCAGTGGCACGATCCATCCAGACAGACGCGTCCGACTCGATCCATCCATGTACCAAATTTGGGC
AACGGTTGCATCGGATTGGACGTAGCACAGACAATGCAAGTGTCCGTCGGTCTGCCGGTCAGGGCATATATGT
AAATGAAACATTACTACTTAAACTTGCTTTTCATTAGATTAGGCCCTACCAATTAAAAAGGCACATTTAAAAAAA
ATAAAAGGGACTACTACTACTGGAGGAGGCCGTTGCCGCTGCCGCTGGCCCCGTCGATCGCCTCCCTAGCGAA
CCAGATGGCCCGCTGCTGGAGGCGTTCATGATAACAGTCGCGGACATGGACGCGAGCCGCCTCCTTTCCCGCC
TCCTTCCTGTGGTCCTTCGCCATTCGGTTCGCCAGACCATTCTCCATCTACGCGGCCAAGTGGTCCCTCGCCTCT
TGGT

>chr7A:1609589-1611689

ACTCGTCAATGTGAATGAGAATGAGGTACAAAACAGCGATAGTGATAGGGATTAGTCGGAAAATATAAAATT
AAATAAAGAAGAAGACATAAAGGATATAACATGACGAAGTTATTTATTAAGATAAGCGGTTATATACCACCCA
CTGACACACTTGTGATGCTTTTATTTCACTATATTGATCCTCATCACTCGCACTAGTTTACTCCGATGCCTTATTA
GTCAAAGATGACACATCCTCGTCCTCTGGATAAGGTTGGAAGCCCGTTTGGGTAAATTTTGGAGAGAATACAC
CAAACTCTTCCCCCCTGTTGTTTTTGAGGTTTTGGGCTTCCTGCCTGGAAATACGGTACGCATTGGCAAGCACAT
CCACGGGCAAGGCACGGAGGATGGAGCTCTTTCCTGCGATGTGGCTAACCATGGAGTTTGGGTTGGTCTTGA

ATGAGATATACTGGCATCCTTCACGCTCCGCCTTCTTGAGAACAACGTAGTGTTGTGGTACGATTAGCAACTGC
CCTTGGCGAAGAAGGTCATTAAATACAGTCTGGCCATTGTTATTGACAACTTGAACTCGAGCATGCCCTTGGAT
CATGTAGACCACGCTATGCGCATTGATGTTCCAGAATGGTGAAAGAACAGCATTCTGCAATAGCAATTATTTGT
ATGCCATATTATTAATCGGATGTTATAAAGCTTGGAATATCTAATAATAAGATAGTGCATTGAAGGTAGTATGG
TATATACCTGGTATAGATTTACTCTTGTAGCGCTCATTTGTACGAGGTTAAGGATGGGGAAATTATTGCCATGG
AGATGTGTTATCCTGCCAGCACGTGGGTTGTATGTGTCGGCACGTTTGGGGTTTTCAATGTTCTGCCTTGCCTC
CAATGAACAAAAGTTCTCCTCCAAACCATTGAAACTTTTATCCCCTGACTGTCCAACCTGGTATTGAGTTGGTTG
TGCTTCTTGATATTGGGTTGATTGCCCTACCTGGTATTGGGTTGATTGTCCTTCTTGACTTCGAATTGGTTGATA
GGCTTGTTGCTCTACTAGTCCTTGTTGGGACACTGTTGGCTTCAAGAACCGAAGGCCTTGACTCACACGAATTA
TCTCACCCCTTTGGTCGTTTTGACTCTGGATCCTCTGTGCTGCTTGTTGGCTTATACCAAGAGCCTCACTAAGAA
GTTGGATATTGAATCCACTGAATATGTTTTGTCCAACTTGTTGCCCTATCTTGTTGTTACCGGCCAACAAGAACT
CCTGAGATTTCATATTGATGAAGTAAAACGAAGTTCCTTAATATCCCACTAATTTATACTTTAAAATCCTAGTTA
GGTTAATATTGAATTATATATCGATATGTAGATTAATTTGTATAGTTACCTTCTGTCTAGGTTCAAGCTGATTCG
CATTGTTGTTTACGTCGAAGACATAGAGAGCTACAATCGGCGCATCGCCATCGTTGTAGCACCAGTGTACAAT
GCCAGCCGGCAGCGCAATAACATCTCCTTGTTTAAAACGGTGAACTCTTTGGTGCTCATCTTTGAGATGGCTTT
GGCTTTGACCCTAAGCTTGATCAAATGGTTGGAACTGTTGTTGGAAGGTTGCTGGGCATCCAGGTAAAGTCAA
TCCCGTGAAACCCCTACCTAAAAGAAGACACAATTGTTTCAATATTAAAAACTTATGAATGAGGGAATATTTGG
AACATCTGAACTCTGAAGTGTAGTATAGAAATGCAATCACCATATTCAATTATACACGAACCTTGAAGGACGTA
TACCAATCCGGGTGCATTGTGGTATTGAGGTAACAAGAGGCCTTGGGGCTCAATAACGCGACGGATGACAGA
TATCCCGGTACAACGGAATTGCTCATTCTTCTCATCAAAGTATTGAGTGACGCCTGCTTGTGACCTCACTTGTTG
AACTGGCTCAAGTGCTTGTAGCCTATCGAACCTGCACCCTCTTAAACCTCCTTGTCGAGAGCTTTGCCATGGAG
TAAAGCTCTGGCCGAATAGCTGGGCCATGGATCCATGGCACAACAGGAAAATGCAGAAGTAAAACAATACTG
ATGAAAAACTAGTAGTTGCC

>chr7A:16122044-16124347

GGGGGTTAGTTGACATTGTATGTGATGTTTATTATGTTGATGTGCCAATGACATAGACAACATGGAGCATAAA
AAATGGCTAATGAAATTTTCAAATCAAAAAATGTATACAAATTTCCAACAACGAAACACATTCCGAACACGCGG
AAGCTTTTATTGTATTCATATCATGATGAGGCGGACATGCAGGGAACATCAAAGTGCTACACACTTATCTTGAT
GTCTTTATTTTACTTTACTAGATTGTCCTCCCATTACATTAGTTCACCAATCCCGTTTGAGCTCTCGTGAAGCCAC
TTTGAGAAAACTTAGGAGTGAATACGCCAAACTCCTCTCCTCTGTTGTATTTGAGGTTTCGGGCTTCTTGCTTTG
ATATGTGGTACGCATTAGCAATGACATCAATAGGAAGGGCACGGAGGATTGAGTTCTTCCCGGCGATGTGGCT
AACCATGGAGTTTGCGTTGGTCTTGAATGAAATGTATTGGCACCCTTCACGTTGTGCCTTCTTTAGGACAACAT
AGTTTTGTGGTATGATTAATAGTTGTCCCGGGCGAAGAATGTCGTTGAACACATTTTGGCCTTGATTGTTAACA
ACCTGAACCCGTGCGTGGCCTTGGATCACGTACATCACACTATGAGCATTAATGTTCCAGAATGGCGAAAGAA
TGGCGTTCTGCAGTTAATAAAATTGAGTAAGTAAAAACGCACAAAATTAGACTTCTGTGTTTAAGGTTACAAGA
TATATAGGTAAAAATGTATTGAAATATAACATTGTAAGTACCTGATAGAGGTTTACTCTTGTAGCACTCATTTGC
ACGGTGTTAAGAATGGGGAATTTTTGGCTGTTGACATGTGTTATCCTACCAGCACGTGGGTTGTATGTGTCAG
CACGTGTAGGATTTTCAATGTTCATTGTTGGCTTATGATCACAAAAGTTCTCCTCCAAACCATTCCAACGACCAC
TAGTTGACTGCTCTACCTGAGATTGCCATGTCTGAGACGATTCTATTTGAGACTGACCAACCTGAGGTTGTGTT
GCCTCAGATTGCTCTACTTGATATGACTGTTGCTCTTGTTGTGGCATCACAATTGGTTGCAGAATTTGAAGCCCA
CGATTCACGCGGATTATCTCACCTCTTTGGTCATTTTGGTTCTGAAGTCTTTGAGATGTGTGTTCACTTATACCA
AGGGCTTCACCAAGCAATTGGAAGTTGAATCCACGGAATATATTTTGACTCACATACTGTTGTAGTGTCCTACT
ACCACCAGCCAACAAAAATTCCTGCAATTTGATCAAGTAAAAATTATATCAAACAATCTTAGAGATTGTTCGAA
AAGACTAAAACCTACTTGATTAGTAAAAAATATTATCAAGCTTAAAATTGTTTTAGATACCGTCGATTACCTTTT
GTCTAGGTTCAAGTTGATTGGCATTGTTGTTTACATCAAAAACATAGAGGGCTACAATCGGCACTTGGCCATGA
TTGTAGAACCAATATGCAATGCCAGCTGGCAACGCAACAACATCTCCTTGTGTAATGCGATGAACTTTCTGGTA
CTCATCTCTAAACTTTTGGCTTTGTTGACCCTCCGATTGCGCTTGATCAAATTGTCCGAACTGTTGTTGGAAGGT
TTCTGGGCACCCAGGGAATGTTACCCCTGCAGAACCAGTACCTACAAGTAAACAAGTTTAATTTGATATTAAAA

ATTAATGATACTTTTATAACATATTTCACATACAAATCCGTAGTATCTAATATAGAGCTATAACCAGTCTATTAA
GTTCATACACCAACCTTGAAGGATGTATACCAAGGCGGGAGCATTGTGGTATCGAGGTAATAAAAGGCCTTGA
GGCTCGATAACACGACGAATGACAGATAGACCAGCACATCGGAATTGGTCATTGTGCTCCTCAAAATACTCAG
TGAGGCCAGCTTCAGACCTCACTTGACGAAGCGGTTCAAATGTTTGTAGCCTATCGAATGTGCAACCCCTAGCA
CTTCCTTGTCGAGAGCTTTGCCATGGTTGACTCTGGCCAAATAGCTGAGCCATGGATCCATGGAACAAGAGGA
AAATGCAAAAGCAAGATAAAAGCGATGGGAAACTAGTAGTTGCCATTCTCGTTGGAAGAGATTGAGAATGTT
GGTTGATGGAGGTCTTGTGGGATGTAAGGGGATTTTATAGCTAAGAAAAGTGGCCTACACCTTATGTAAAGAC
ATGGTTTGTTAAGATAAATGTGTGATGACATGAAAATTTGGACGTAATAGAGAAATCAAATGGATAAGCATGA
TTGCAAGCAT

>chr7A:16200644-16204623

TCAACCAACCTTCTCAATCTCTTACAACAAGAATGGCAACTACTAGTTTCCCATCGCCCTTATCTTGCCTTTGCAT
TTTCCTCTTGTTCCATGGATCCATGGCTCAGGTATTTGGCCAGAGTCAACCATGGCAAAGCTCTCGACAAGGAA
GTGCTAGGGGATGCACATTCGATAGGCTACAAGCATTTGAACCACTTCGACAAGTGAGGTCTGAAGCGGGCCT
CACTGAGTATTTTGAGGAGCAAAATGACCAATTCCGCTGTGCTGGTCTATCTGTCATTCGTCGTGTCATCGAGC
CTCAAGGCCTTTTGTTACCTCGATACCACAACGCTCCCGCCTTGGTATACATCCTACAAGGTTGGTTTATGAACT
TAATAGAGTGGTTATAGCTTTAGATTATATACTAGATTTCTATGTGAAAAATGTTACAAAAGTATCAGGAATAT
TTAATATTGAATTGAACTTATTTTCTTGTAGGCACGGGTACTGCAGGGCTGACATTCCCTGGTTGCCCAGAGAC
CTTCCAACAACAGTTCGGACAATTTGATCAAGAGCAATCTGAGGGTCAGAGCCAAAGACAAAAGTTTAGAGAT
GAGCACCAGAAAGTTCATCACATTAAACAAGGAGATGTTGTTGCGTTGCCAGCTGGCATTGCACATTGGTTCT
ATAATCATGGCCAAGTGCCGATTGTAGCTCTCTATGTTTTTGACATAAACAACAATGCCAACCAGCTTGAACCT
AGACAAAAGGTAATTGAGAGTATCTAAAACAATCTCTAGGATTGATAGTCTTTTCGACTAATCTACTAGGTTTA
GCCTTTTTGACCAATATCTAAGATTGTTTGATATATTTATTACTTGATCAAATTGCAGGAATTTTTGTTGGCTGG
AGGTAGTAGGACACTACAACAGTATGTGAGTCAAAACATATTCCATGGATTCAATTTACAGTTGCTTGGTGAA
GCCCTTGGTATAAGTGAACAGACATCTCAAAGACTTCAGAATCAAAATGACCAAAGAGGTGACATAATCCGCG
TGAACCGTGCGCTTCAAATTCTGCAACCAATTGTGCTGCCACAACAAGAGCAACAGTCGTATCAAGAAGAGCA
ATATCAAGCAACACAACCTCAGGTTGGTCAGTCTCAAATAGAGTCGTCTCAGACGTGGCAATCTCAGGAAGAG
CAATCAACTAGTGGCAGTTGGAATGGTTTGGAGGAGAACTTTTGTGATCATAAGCCAACAATGAACATTGAAA
ATCCTACACGTGCTGACACATACAAACCACGTGCTGGTAGGATAACGCAAGTCAACAGCCAAAAGTTCCCCATT
CTTAACACTGTGCAAATGAGTGCTACAAGAGTAAACCTCTATCAGGTACTTACGATGTTACATTTCAACACATTC
TTATCTATATATCTTGTAAACTTAATAATAGAAGTCTAATTCTATTTCCTTTTACTTACTCAATTTGATTAATTGCA
GAACGCCATTCTTTCGCCATTCTGGAACATTAATGCTCATAGTGTGATGTACGTGATCCAAGGCCACACACGGG
TTCAAGTTGTTAGCAATCATGGCCAAAATGTGTTCAACGACATTCTTCGTCCGGGACAACTATTAATCATACCAC
AGAACTATGTTGTTCTAAAGAAGGCACAACGCGAAGGATGCCAATACATTTCATTCAAGACCAACGCAAACTC
CATGGTTAGCCACATCGCCGGGAAGAACTCAATCCTCCGTGCCCTTCCTGTTGATGTCATCGCTAATGCGTACC
ACATATCAAAGCAAGAAGCCCGAAACCTCAAATACAACAGAGGAGAGGAGTTTGGCGTATTCACTCCTAAGTT
TTCTCAAAGTGGCTTCACGAGAGCTCAAACGGGATTGGTGAACTAATGTAATGGGAGGACAATCTAGTAAAGT
AAAATAAAGACATCAAGATAAGTGTGTATCACTTTGATGTTCCCTGCATGTCCGCCTCATCATGATATGAATAT
AATAAAAGCTTCCGCGCGTTCTGAATGTGTTTCGTTGTTGGAAATTTGTATACATTTTTTGATTTGCAAATTTCA
TTAGCCAATTTTTATGCTCCATGTTGTCTATGTCATTGGCACATCAACATAATAAGCATCACATACAATGTCAAC
TAACCCCGTATATCTTTTGATAATGTGAGGGCTGCCGTATTTTATTGTTGTACTAGTATACAAGGACTCAAGGA
CACGACCATAGGAATGTCTGCAAGTCGCGTTGGCACAACCGCACAAACATGTGGGCCTCACTTTGCAGCCGAG
GCCTAGTGAACCTGTGTCTAATGAGATCAATTGGAGGAACCTGGCTATGTTATTTACACTTGGATGTTATCTAG
AGACGTTCCACCATGTTCTCTCTTAACATCAATTGGAGATGCAGTTTTGGGAGTCGCTTCCTGGTGGTTGGGCC
TACTCCTTGCCCCTCTCGCCGGATTAGCCGGAGAGTGGTGGTGAGAAGACGGCACCGGAGTAGGATTAGGTC
TCTGTAGGAGTCGTAGGTGTAGGGTTTGGCCAAGTGTCGTGGTCTAGCATCATGCCATGGAGAAGAAGCTTCT
AGATCTCGTCGGCTGGATCTGGGGGTGTTACCTCCTTCTGTCCCTTGCGGTGGTACTCCGGTGGCCGGAGTCG
GATGCGGTTGAGGGCCATGTTGTAGGGGTTCGTAGCATGGAAAACAAAAATTTTCTATCGCATACAAACAAAC

AAGCCAAGATCTACCTACTAGATTGTAGCAACGAGTCGATGGAATAACTTACCCTCGAAGATTCCAAAGCGGT
TAACGAGAGCAGATTCTCGTGGGTGATATAGTCGTACTCTTTCCGATCTCAAGATCGCGTAGACGATCCTTCCA
GTGCCACGTTCCGGCAGCGCTCCCGCAGTCGGTCACATGTTCGGTACTCGACGAAGACCTTCCTCTCCCTTGTT
CCAGCAGGCAGCGGAGGTTGTAGCACTCTCCTCGCGATTCCGGCAGCACAACGGCGTGGTACCGGAAGTAGA
GGGCAGATCTAGGGCTTCGCCTTAAGCCGGAGCGAGTATGGTGGAGTATGAGAGAGGCTTTAGTGGTCTAGG
GATTAGACACACCAATTAGAAGGCCCTAAACCGAAGGCTAGGGCAATTAGAAGCTCTAAACCAAAACGGCTCT
AAACCGGAGGCTGTGGCTAGGGTTTAGATTTAGAGGATAGGAACTTGTGTGTCTAGGAAGGGCAGCCCTGCC
CCTCCCTTTATAGGTGGGAGGTGCGGCCAAGGGGAGGGGAGTCCCTCCCCAAATCGGCTTGGAGGTGGGCCG
CCGGTTGGAGGAGGGAACTCCTCCCCAACTCGGTTTCAATCAAACCTCCTTCTCCTCTTTACCAAAACAAGCCG
TTTGACTTTTCAAACTAGCCGTTTGACTTTTCAAACTAGGATTTGAATAAATCCTCTATTTGGTACAGCGATGAC
ACTTAGTACATGATGCAATCATACCAACTTGATATAACACATTCATGTACCCTTCCCCCCCCCCCCCCGGAGTGC
CGGACATTCCGGAACCTTCTAGAACATTCCCGATGAAAACACCGGAATCATTCCACAACTCCGAAACGGGACTT
TCCTTATATGAATCTTATTCTCCGGACCATTCCGGATCTCCTCGTGATATCCCGGATCCCATCCGAGACTCCGAA
CAACATTCGGTCTCTTTCTCATCATTCCATAACTACTCAAACAACGTCGAATCCTTAAGTGTGTCTCCCTACGGTT
CGTGAATTATGCGGACATGATCGAGATACTTCTCCGATCAATAACCAATAGAGGAATCTGGAAATTCATATGG
CTCCCACATATTTCTACGATTACTTTGTGATCGAATGAACCACTTACATACGATACCAATTTCCCTTTGTCTCACG
ATAGTTTACTTATCCGAGGTTTTGATCATCGGTATCTCTGCATACCTAGTTCAACCTCGTTACCGAT

>chr7A:1645693-1647765

ATAGGGAAAGGGGATTAGTCGGAGAATATAAAATTAAATACAAACAAGACACAAACAAAGGATAAAACATGA
TGAAGTTATTTATTAAGATAAGCGGTTATACACCGCCCGCTTTCACACTTACGATGCCTTTATTTTACTATATTTG
GTCCTCATCACACTCACTAATTTACTCCGATGCCTTATTAGTCAAAGATGACTCATCCTCGTCCTCTGGATAAGG
TTGGAAGCCCGTTTGGGTAAATTTTGGAGCGAATACACCAAACTCTTCATCCCTGTTGTTTTTGAGGTTACGGG
CTTCCTGCCTGGAAATACGGTACGCATTGGCGAGCACATCCACGGGCAAGGCACGAAGGATGGAGCTCTTTCC
TGCGATGTGGCTAACCATGGAGTTTGGGTTGGTCTTGAATGAGATATACTGGCATCCTTCACGCTCCGCCTTCT
TGAGAACAACGTAGTGTTGTGGTACGATTAGCAACTGCCCTTGGCGAAGACGGTCATTGAATACAGTCTGGCC
ATTGTTATTGACAACTTGAACTCGAGCATGCCCTTGGATCATGTAGACCACGCTATGCGCATTGATGTTCCAGA
ATGGTGAAAGAATAGCATTCTGCAATAGCAATTATTTGTATGCCATATTATTAATCGGATGTTATGAAGCTTGG
AATATCTAATGATAAGATAGTGCATTGAAGGTAGTATGGTATATACCTGGTATAGATTTACTCTTGTAGCGCTC
ATTTGCACGAGGTTAAGGATGGGGAAATTATTGCCATGGAGACGTGTTATCCTGCCAGCACGTGGGTTGTATG
TGTCGGCACGTTTGGGGTTTTCAATGTTCTGCCTTGCCTCCAATGAACAAAAGTTCTCCTCCAAACCATTGAAAC
TTTTATCCCCTGACTGTCCAACCTGGTATTGAGTTGGTTGTGCTTCTTGATATTGGGTTGATTGCCCTACCTGGT
ATTGGGTTGACTGTCCTTCTTGACTTCGAATTGGTTGATAGGCTTGCTGCTCTACTAGTCCTTGTTGGGACACTG
TTGGCTTCAAGAAATGAAGGCCTTGACTCACACGAATTATCTCACCCCTTTGGTCGTTTTGACTCTGGATCCTCT
GTGCTGCTTGTTGGCTTATACCAAGAGCCTCACTAAGAAGTTGGATATTGAATCCACTGAATATGTTTTGTCCA
ACTTGTTGCCCTATCTTGTTGTTACCAGCCAACAAGAACTCCTGAGATTTCATATTGATGAAGTAAAACAAAGTT
CCTTAATATCCCACTAATTTATACTTTAAAATCCTAGTTAGGTTACTATTGAATTATATATCGCTATGTAGATTAA
TTTGTATAGTTACCTTCTGTCTAGGTTCAAGCTGATTCGCATTGTTATTTACGTCGAAGACATAGAGAGCTACAA
TCGGCGCATCACCATCATTGTAGCACCAGTGCACAATGCCAGCCGGCAGCGCAATAACATCTCCTTGTTTAAAA
CGGTGAACTCTTTGGTGCTCATCTTTGAGATGGCTTTGGCTTTGACCCTGAGCTTGATCAAATGGTTGGAACTG
TTGTTGGAAGGTTGCTGGGCATCCAGGTAAAGTCAATCCCGTGAAACCCCTACCTAAAATAAGACACAATTGTT
TCAATATTAAAAACTTATGAATGAGGTAACATTTGGAACATCTGAACTTGAAGTGTAGTATAGAAATGCAATCA
CCATATTCAATTATACACGAACCTTGAAGGACGTATACCAATCCGGGTGCATTGTGGTATTGAGGTAACAAGA
GGCCTTGGGGCTCAATAACGCGACGGATGACAGATACCCCGGTACAACGGAATTGCTCATTCTTCTCATCAAA
GTATTGAGTGACGCCTGCTTGTGACCTCACTTGTTGAATTGGCTCAAGTGCTTGTAGCCTATCGAACCTGCACC
CCCTTAAACCTCCTTGCCGAGAGCTTTGCCATGGAGTAAAGCTCTGGCCGAATAGTTGAGCCATGGATCCATG
GCACAACAGGAAAATGCAGAAGTAAAACAACACCGATGGAAAACTAGTAGTTGCCATGATTGTTTGAAGAG

>chr7A:1703151-1705569

GGAGGGGGGGGAGGGGGAATCGATCGGGAGCGGTATACACATACATCATCATCGGTACATACGAGTGTAGG
GGTTCGTAGCATGGAAAACAAAAATTTTCTATCGCATACAAACAAACAAGCCAAGATCTATCTACTAGATTGCA
GCGACGAGTCGATGGAATAACTCACCCTCGAAGATTCCAAAGTGTTTAACGAGAGCAGATTCTCGTGGGTGAT
GTAGTCGTACTCTTTCCGATCTCAAGATCGCGTAGACGATCCTTCCAGCGCCACGTTCCAGCAGCGCTCCCGCA
GTCGGTCACACGTTCGGTACTCGACGAAGACCTTCCTCTCCCTTGTTCCAGCAGGCAGCGGAGGTTGTAGCACT
CTCCTCGCGATTCCGGCAGCACAACGGCGTGGTACCGGAAGTAGAGGGGAAATCCCGCAGGGCTTCGCCTTA
AGCCGGTGCGAGTATGGTGGAGCTTATGAGAGAGGCTATTAGTGGTCTAGTAATTAGACACACCAATTAGAG
GGCTCTAAACCAATTAGAAGGCTCTAAACCGAATAGCTCTAAACCGGGGTCTAGATCTAGGGTTTAGTTTAGA
GGATAGGAAACTTGTGTGTAGGAGGGGAGCCCTGCCCCTCCTTATATAGGTGGGAGGGGGTGGCGCGCCAG
GGGAGGGAGGGAGTCCCTCCCCAACTCGGCCTAGGCTGGCCGCCGGCTTGGAGGAGAGAAACTCCTCCTCCA
AGTCGACTCCAACTCCTCTTGCTTGTTTCCCACAGTTTCTTGTTTTTGGCAGTTTGACTTTCAAACTGGACATTTG
ACTTTTCTGTTTTAGGATTCAATTTAATCCTATATTTGGGACAGCAAGAACACTTAGTACATGATGCAATCATTC
CAATATGTAATGACACATTCATGTACCCCTCCCCCCCCCCCGGAGTGCCGGACAATCCGGAACCTTCTAGAAT
ATCCCCGATGAAAACACCGGAATCATTCCCGAACTCCGAAACGGGACTTTCCTTATATGAATCTTATTCTCCGG
ACCATTCCGGATCACCTCGTGATATCCCGGATCCCATCCGAGACTCCGAACAACATTCGGGCTCTTTCTCATAAT
ACCATAACTACTCAAACAACGTCGAATCCTTAAGTGTGTCTCCCTACGGTTCGTGAATTATGCGGACATGATCG
AGACACTTCTCCGAACAATAACCAATAGAGGAATCTGGAAATTCATATGGCTCCCACATATTTCTACGATTACTT
TGCGATCGAATGAACCACTTACATACGATACCAATTTCCCTTTGTCTCACGATATGTTACTTATCCGAGGTTATG
ATCGTCGGTATCTCTGCATACCTAGTTCATCCTCGTTACTGATAAGTACTCTTTTCTCGTTCCCGTGATATGTGAT
CCCTTGTGACCAAGTCACATGCTTGCAAGCTAATTGGATGACATTTCACCGAGAGGGCCCAGAGAATATCTATC
CGTCATGTGGATGGACAAATCCCACTCTTGATCCATGTGTTTCAACCAATACTTTCCGAACACTCAATGCCACCT
TTATAGCCACCCTGTTACGGTGTGGTGTTTGATGACATCAAAGCATCCATCCAGTAAAGGTGATATGCATGATC
TCATGGTCGAAGGAATTAGGTTACTATGTATCAGAAAGCATATAGCAATTGAACTATTGAAATGACTTGATCTT
TATGCTACGCTTGTCTATAGGAGTATGTCCACCATATCATTCTCCTAACGATATGATCTTGTTGTTGATAACATCT
CAATGTTCATGATATGGAAACCACAATCATTTAATCAACCAACAAGCTAGTGAAGTATAGAGGCTATACTAGG
GACTTCAATTTAAGTTTACAATTCACACAAGTATTAATGTTTCCTTGTTAATACTATTATAGCATGAATATAAACA
TTTATCATAAACACAAAGATATATAATAATAACTACATTTATTATTACCTCTAGGGCATATCTCCAACAGTCTCCC
ACTTGCACTAGAGTCATCTAATCTAGATTACATGGTAATATACCGAACACCCATGGCAGTTTGGTGTTGGTCAT
GTTTTGCCCTCGGGAGAGGTTTAGTCAACGGATCCGCCACATTCAGATCTGTGTGTACTTTGCAAATCTTTATAT
CCCCATCCTGGATATGTTCTCGAATAGCGTGAAAACGCAGCTTGATATGCTTCAGCTTCTTGTGTGACCTTGGTT
CTTGCGCATTGGCTATGGCGCCCATGTTATCACAGTAGATAGACATTGGGTCCAGCGCACTAGGAACCACACC
CAGCTCTGTAATGAACTCTTTCATCCATATCGCTTCTTGTGAAGCCTCCGAAGCAGCCATGTATTCCGATTCAGT
TGAAGATCTCGCCACAACGCTTTGCTTTGCACTCCTCCAGCTAACTGC

>chr7A:1878391-1880391

GTTTTTTTTTTTTTTTTTTGAACAAAGAGAGGTCCCGAAGGACTTACACAGCTGCATTTTATATTGAGCGGTGG
AAACTATCCAGAAAATAAAAGACACAACCACGTTCCTGCAATTCACAAAAAGGACCCTCGACAAGATAAGAAA
AAAGCAATCGAGTTCATCACCACCGTCTGGAATCACCGACGAGAAGCTTCCACGCGTGGCCAGCAACGTCGCC
ACCGGGGACTAGACCACTTGGGACGACGCCGCCATGAGATGCCACACTGGAGCTAGAGCTGCCTCCCTTTTGG
CGTGCTTGCCGGGAGGAATAACTGGGTCGACGTCGGACTTGTGCCAACGCCGGATCACCGCATGGCCGCCTTT
CGGCCAGAAAAGTCAGCAGCAGCGCGACAAGATGCCATTGAAGAGCTACTGAGGAAGAACACCAAGATATTG
CAGTCTGCACTGCCCCAAGATCAAGCGCACAAGCAACAAATCCAATCCCAACTCCCGCTTCACCACCTTGGTGA
CTTAAGAGAAGAGGACCGGAGGTCACCATGGCAAGATATGAGGCGACCTTGAGCTTATTTAGAGGGTAGGCC
GCAGTTCCCCTCCGTCCCATCTCCGGCTCCGACCTCCAGAGCGCCATGGGAAGGAGAACCACCACCAGGCACC
GGACGCAGCTCGCATGCTCCGGCAACCATCTCCCCTTCGGCATCACGCCATACTCCACATGGGAAAAAGCCAA
CCTATCTAACCTACCGCTACTATGGACAAGGAGGGGCCGGCCTCCTCCCCCCCACCCTCTCCGGCCGGCTAGAC

CGCCGGAGACGAGGGGGAGAGGAGCCGGGGAGCACCAGGCGACTCTCAAAGGAAAGGGAGAGAGAGGGT
CCGGGGACTCAGCGAGGGACCAGTTGGTACCGGCAAAAGCATTGCGTGAAGACATGGAGAGGCTCTCGTTGT
CATCGGCGTGGCAGCATGCGGTTCCGTACTTCGACCCAGTGGCCTGGCCGACGGCAGCGTGGGTGGGGATCC
TCTAGGTCCACTCGTTCTTGGAAATGGGGGTTTGGAGAAGCAAACCGGACTCCGAGCACTTATTAGCAATTGA
GTTTGGAAAAGTATTAAAATCTTATGTGTTTTGTATCCTTACAAAATGATCATATCTTATTTTAAAAAAATGCAG
AAAGGGTATTACTTGTTCAAATCACTAAAAATTATTCAAGTTACTGAAAAAGTGTTTATGCACTATAAAGTCGA
AAAGTTTCATATTTAAAAATGGTTCAGATTCAAAATAAAAGGTTTATGTTCAAAAAAAAGTTCAGATTCATGAG
GTTCAAAACATGTTCATGTTCAAAAATTGTTCATGAGGTTGTAAAACAAATCACGAGATATAATAATAGTTCAC
CGGGTAAAAAGGTTTATACTTTAAAAAAAAAGTTGACCAGGCAAAAAAATCAGATTTATAAAATGTTTATGATTT
AAAATGGTTCATATTTTAAGAATTATGACCCATTTGGTATGGTCATGGGTCTGCATGAAACCCGACCTGATTGC
CTGGCCGAGTTGCAGATATAATATAGAATAGTACGTAGCTTTAATAAAAATCTCTCACAAATTAAGAACACTGA
GCGAGGCAAAAAAAGTTAGGAGTAAAATAAGTCCTGAGAGAGCTCACAAGTTATCCGTCACAAAAATAGCAA
ATCAAAATAAGTTTCACATATAGCGCACGGCAACCAAAGTGTACTTGCTAATTACTTACCCTCAAGTTAAGGGG
CCGATATGAATAGAGCAAATCCGATCAAAGATAGGTGTCCGTTGTGCTAGGCAGGAGCCAGGAGCCCAGGAG
CAGTAGTAGCAATAATGTGGAATTGATCTTCATGCATGGGCGGGTGTGGTTGTGGCAGGGCGCAACTCTGCG
GCGCCGTTCTCATCTCCTTGAGAACACCGTAGACCGAGCACAACAATGCCTGGTGCTAGCCGCTCTGCACCCCT
GGGGTTTCTTGGGACATAAATCTATG

>chr7A:37286395-37288694

TTATTATATACACATAAATTATACACGTATGGTGATGATAGAAAATATTCTCTATCTGCCAATAATGAACAATAC
ACCATTATTGGACCGTAGTTGAACTCGTGGATACCTCAGTCTTGGAAAACAAATTCTTGTCCAGTTGGCCCATA
TGACTCGTCGATACGAATGAGAATGAGGTACAAAACGGCAGAGAAGAGACAAAGATAGGGAAGGGGATTAG
TCGGAGAATATAAAGTTAAATAAGAACATGACACAAACAAAGGATATAACATGATGAAGTTATTTATTAAGAT
AAGCGGTTATATACCATCCACTAACACACTTGCGATGCTTTTATTTCACTATATTGATCCTCATCACACTCACTAA
TTTACTCTGATGCCTTATTGGTCAAAGATGACTCATCCATATCCTCTGGATAAGGTTGGAAGCCTGTTTGGGTA
AATTTTGGAGCAAATACACCAAACTCTTCCCCCCTGTTGTTTTTGAGGTTTCGGGCTTCCTGCCTGGAGATACGG
TAGGCATTGGCGAGCACATCCACGGGAAAGGCACGAAGGATGGAGCTCTTTCCTGCGATGTGGCTAACCATG
GAGTTTGGGTTGGTCTTGAATGAGATATACTGGCATCCTTCACGCTCCGCCTTCTTGAGAACAACGTAGTGTTG
TGGTACGATTAGCAACTGCCCGTGGCGAAGACGGTCATTGAATACAGTCTGGCCATTGTTATTGACAACTTGA
ACTCGAGCATGCCCTTGGATCATGTAGACCACGCTGTGTGCATTGATGTTCCAGAATGGTGAAAGAACAGCAT
TCTGCAATAGCAATTATTTGTATGCCATATTATTAATCAGATGTTATGAATCTTGGAATATCTAATATTAAGATA
GTGCATTGAAGGTAGTATGGTATATACCTGGTATAGATTTACTCTTGTAGCGCTCATTTGCACGAGGTTAAGGA
TGGGGAAATTATTGCCATGGAGACGTGTTATCCTGCCAGCACGTGGGTTGTATGTGTCAGCACGTTTGGGGTT
TTCAATGTTCTGCCTTGCCTCCAATGAACAAAAGTTCTCCTCCAAACCATTGAAACTTTTATCCCCTGACTGTCCA
ACCTGGTATTGAGTTGGTTGTGCTTCTTGATATTGGGTTGATTGCCCTACCTGGTATTGGGTTGATTGTCCTTCT
TGACTTCGAATTGGTTGATAGGCTTGCTGCTCTACTAGTCCTTGTTGGGACACTGTTGGCTTCAAGAACTGAAG
GCCTTGACTCACACGAATTATCTCACCCCTTTGGTCGTTTTGACTCTGGATCCTCTGTGCTGCTTGTTGGCTTATA
CCAAGAGCCTCACTAAGAAGTTGGATATTGAATCCACTGAATATGTTTTGTCCAACTTGTTGCCCTATCTTGTTG
TTACCGGCCAACAAGAACTCCTGAGATTTCATATTGATGAAGTAAAATGAAGTTCCTTAATATCCCACTAATTTA
TACTTTAAAATCCTAGTTAGGTTAATATTGAATTATATATCGCTATGTAGATTAATTTGTATAGTTACCTTCTGTC
TAGGTTCAAGCTGATTCGCATTGTTGTTTACGTCAAAGACATAGAGAGCTACAATCGGCGCATCACCATCGTTG
TAGCACCAGTGTACAATGCCAGCCGGCAGCGCAATAACATCTCCTTGTTTAAAACGGTAAACTCTTTGGTGCTC
ATCTTTGAGATGGCTTTGGCTTTGACCTTGAGCTTGATCAAATGGTTGGAACTGTTGTTGGAAGGTTGCTGGGC
ATCCAGGTAAAGTCAATCCGGTGAAACCCCTACCTAAAATAAGACACAATTGTTTCAATATTAAAAACTTATGA
ATGAGGTAACATTTGGAACATCTGAACTTGAAGTGTAGTATAGAAATGCAATCACCATATTCAATTATACACGA
ACCTTGAAGGACGTATACCAATCCGGGTGCATTGTGGTATTGAGGTAACAAGAGTCCTTGGGGCTCAATAACG
CGACGGATGACAGATACCCCGGTACAACGGAATTGCTCATTCTTCTCATCAAAGTATTGAGTGACGCCTGCTTG
TGACCTCACTTGTTGAATTGGCTCAAGTGCTTGTAGCCTATCGAACCTGCACCCCCTTAAACCTCCTTGCCGAGA

GCTTTGCCATGGAGTAAAGCTCTGGCCGAATAGCTGAGCCATGGATCCATGGCACAACAGGAAAATGCAGAA
GTAAAACAACACTGATGGAAAACTAGTAGTTGCCATGATTGGTTGAAGAGGTTGTTGTACAAGGTGGATTGGT
GCT

>chr7A:37289296-37291296

TTTTTTTTTTTAATAATAAAGGAAGGAGTGTTTCTTGTAGTACCTACACTTTTTTAGACCGTTTTGCCCTTCCATT
AAGCCACTTATTACCCCCAAGCCACCGGTGAGTACACGAGATATCATGCAGTCGTACGTATGTGATTCGCCGG
GCTGTTCTGCTAGGGCGACGGGTATTTTTCTAGCCTGATCGATTGTTTGCCTAGCTTGACCGGCTGATCGGTAC
TTCTTTTTTCCCCTACCAAACGAACTAGATGGTACACGTACGTGGTACTGAGAGTCTGAGATCGATCCAACTGC
TGGTACGGTATCCGCAAGTTGCATAGGGGGGCGTAGAGCACGAATGATCATGGACAACAAGGATTAAATGAA
CGCAGTAGTATGGTGGACCTGTACCAATCTCATGAGGCCCCCGGTTGGTTTAATATGCTGCGACGGGGCCAGT
CTTGGCTGCTAGCAATTGCCGGAGGTGGCACCTGCAAGGCGAGGTTGAGGAGTGGCGTGGGTTGTGCCGGTA
CAGGACATGATCTCAAGGAGCGAAGCGCTGCACTCAGGGATGGGGCCGTGGTCGGCGGCTAGAGCAGAGGC
GGGAGGCGGCGGTCGCCGGCGAGACGAGACTCAAAGGGCGCCTGGAGGCGGACGGAGATAAGCATGACGA
CGCTCTATGGGGCACGAGGAGAGGACTCTAGGGGGACACGAGCGAGTGCTCGGGTTCACGGAGGCCTCGTG
ATCCAGTGGAGAGTGAAGAAGGGGACCTGATTGCTTTCTACAGCTGGGCGGCCGGGTGGTAAGTGTAAAAG
GGACAGGGCACCGACGCACTGTGTGTGTGGTGCCATGCACGTTCATTGACTGGAGCAATATTTGTGTTTTATC
GACTTTTTTGATCAAAATTATTTTTTTCCGTTGCAACGCACGGGGCTTCTTGCTAGTCACTCCTTATTTGTGAACG
GCAAGTGGAAGGAGGATGGCATGATGCACGGGACAGGTGCGGCGGGAACGGGTTGTCCTTTGTCACGATGC
AGGGGACATGTGCGGCGGGAACGCGATGTCATTTTTCATGATGTGCGCCCGTCAAGTATCAACCCTGCCATGA
ACCTTTGTTGGGCTTATGTAGGCCGGCTGCGGCGGGGTGAGCCTTGTACCAGGATTTGGCGATCGATGGAGC
CCCTAGATTTAGACCACCATGAGAGTAGAACCTAAAACTTACATACTAGAACTATACCTTAAACCCGAGACACA
ATTTTTAAAGTGACACATCCTTTCAAAAACCTAGACATGGGCCCACACTCCCAAGATATATACGCGAGATCCCA
AGATCTAGAAACCGAATCCGTAGACCTAGACAATTGGAAAATAGACCCTAGACCAGGAAAGTAGAACATTGA
CCCTAGACTTATACTACATCAAACGTTGGACAAAACTTTGAAATGACGCCTCCTTTGAAAACCCTAGACCGTTG
ATATATACCATATACCCCTAGATCCCAATACCTAAACCCTACACCCTAGGCACAATGATCACCTCCTTTGAAAAC
CCTAGATAATAACTTGCTAGTCGGTGACGTGCCCACAAGCTTGCTTGAGTGCCTCGCCGCCACCAGCGGAAGA
ACCCTCGACGAAGAAGAACCGAACACGATCACTAACACGCTGGGAAATTCGGGACGCTCAACGTCGTCATGTG
TGGGAACAACAACACTCTGTCCGGGAGAAACAACACGCTGAGCGGGAGCGACAACGTCGTCGCCGGCAGCAA
CCAGGTCGTGTGCGGGTGGAACCACGTCGTCTCCGAGAACAACAACGTCGTCACCGGGAACGACAACACCGT
CACCGGGAGCTTCTGCACCGTGTCCGGGCACCACAATACGGTGTCAGGGAGCAACAACACCGTGTCCGGGAG
CTACCATACCGTCTCCGGGAGCAACAAGGTCGTCACGGGAGGGTGATGCATGATCTGTACCAATTTAAGACAT
CTATTTTGGGACGGAGGGAGTATAAATTTGCATT

>chr7A:37314853-37316853

TTTATTCTTATATATATATTGTCCCCATTCTTCATATATATTTAGATCGAACGGATGCGGGACCAACTCCTACCAT
TTGACCGCATACGAGCCATTCAAGAGGAATTGACGGAGTTCATACTTGATGAGGTCGTCACGCCTGGCGGAGA
ACTCTTTAGATATCCATCAAAATGAACGTTGTATATATATGCTTGAACGTGTTTGTCTTGTACTTGATGCTTCAA
CGTTTACGGAGAATACCACAATGACGATGTTTGTGTATAATAATAGTTGCTTAATTATTCATGTACTCGTATGTA
CCGACGACGATGTATGTGTATATCTCTCCCGATCGATCCCCCTCTCCCCCTCCCCCCTCCCCGTCCCCGCCGCGC
CGCAGAAATACGCCCAGAACGGGGCGGCGGCAGAGATTTATGTTGTTTTCCTTCCGCGGCAGAACTCTGCCCG
GCAGAGATTTATGTCTTTTTCCTACCGCGGCAGAGAACCTTTAACACCAGTTGGCAGCCCCACGTGCTCCACCC
TTTAACAGGGGGGCTCTAACACCGAACCCCCAACAGCGGTTCTGGAACCGCTGTTAGAGGCCTCTGTGAACCG
GTGTTAGAGGCCACGTTTCCACTAGTGTGAAGAGTAAACAAGATTCGGAGGAAAATCTAATTACCCCCTTCGA
TCCATCGTTAATTTACACTATTATGGATCAAAGGGAATAAATCATATGTGCATGCACTGGCATGACATCATATA
TATGGAGGCCTCACAACATGTTTGTTACATCGAACTAGTCATGCTGTTTTAGAACCGACTTAAATTGAGGGAAT
TGACCGCTTAATGCAACCAAGTCCATGACATGACTCTGGTGCATTGGGCAAACGAGGCTTCCATCAAGATTAG
TGGCTAAAATGGCCTTAGAAAAAGTCACGACATCCTTTGCCTTGTATCCTTGGAAATTTGGAAGGGACGTACAC

TAGGGCGTTCCATCCTTGGAAACTTCAAAACTATGTTGTTATAAAGTGCAAAGGATTTTTGAAAAAAAAAGTTT
AAAAGATTTCTCCTCGAATCAAGCTTTCGCCCCACTTTATAAATAAAACATCCAAAAGATTTCTTGAGTAGGGTA
AAACTAGGAGAGTAGGGCTTTGTATAAATTCTTGAGCATCCAACCGTGCTGGAGGTTGATATTTCCCAATTTAC
ATCACCTTTATATATCATAATTTATCTCTATTCATTGATATATGTCTTATTATGAGGTATTATTTATGGCTTTTCAT
CTGTTTATGCATGTTGGATGATTCTTGGAGGATGAACTGTCGGAGTCTGGATTCTGATGGAAAAGAAGGCGTT
TCGGTGCAATTTCGCGAAGATCAGAAAATACCAGAAAAATATGGGAAAATATTATTTTTCGAGAAGATTGAGG
AAGCCAGAAGGAGGAGCGAAGAGGGGCCGCATGGGGCCACACCCCCTAGGCGCGGGTCCCCCTGGGCCACG
CCTAGGGCAGGTGTGGCCCACCTGCCCGGCTCCTGACTCCGCCCCTTCGCCTATATCTTCCCTACGAAACCCTA
AGGCTAAGGGGCGACCACAGATAGATTTTCGCCGCCTCTGGGGGCGGAAAACACAGAGATAGAAAGATCACT
CCGGCACACAGATTTCTGCCGAGGAAATTCCTTCCCGGAGAGGGGAGACCGTCGCCATCAACACCATCGTCAA
GCTAGGCTTCATCGGGATCATCATCATCATCCATACCATCATCTCCACCAACACCCTCATCACCACTCCGTCCCG
CTGTAACAATTGTGTTGGATATTGCTTTAGTTCTTAGAGGAAACTTTCCCGGTGTTAATTACTATTGTAGTTGAT
GCTTGTGAGTGGAACCATTGGATTATTGTTTATGTTCAGATTGGTATCCACTACCATATCATCTTTGATCTTGAT
CCATATGATGTCTTGTGAGTAGTTCGTTGTGTTCTTGAGGACATGGGAAAAGTCATGTTGTTAGTAGTCAATTT
GTG

>chr7A:37334397-37336756

AAAAAAGAAAAAAAAAAGAGGAAAACTTACGGCGGCGGCGACCACACGTACGGGCGGCGGCGGAGGCGCG
GGGAAGACAGGCGGCTACGGGGAAGACGGGCGGCGGCAGCGCGGGAAAGACGGGCGGCGGCGACGCAGG
GAAGATAGGGGCGGCGACCACACGTACGGGCGGCTACGGGTGACGCGGGCGGGGCACGGCTACGGGCGAC
AGCGACGGGCGCGGGGAAGACGGCGACAAGATTCACGGGGCGCGGGCGACGGCGACAGCCTGGCGGCGAT
TTGGAGAAGAGGAGGGTGTCCCAGTGCGTGGCTCGGGCCGGGAGGAGGGCAGTGGGTATTTATACCCACCA
CCTCTAGTCCTGGTTCCAGCCTCTAGCCGGGACTAGAGGGGGGGCCTCTAGTCCCGGTCTCAGCCACGAGCCGG
GACTAGAGGTCTTTTCGGCGCACCGTTTCGTTCTCGCGCGCAAAAACCTCTAGTCCCGGCTCGTGGCTGGGGCC
GGGACTAGAGGTTGATTTTTTTATTTTTATATCTTTTGTTCTGTGATAATGAAAAATATGTATTATATATCAAATT
TTGCAGAAAAAAAGATCTATAATGTAAATCTATTAGCATATTTTGTTATAAAATTAATATTTTGTTGAATTAAAAA
ATATCTTTTGTATAGTAGAGTTTGAATTTATTAATATTATTATTTCAATATTTTTTATTCTGTAATGATGAAAAAT
ATGTATCATATATCAAAATTTGCAGGAAAAAAACTCTATAATGTAAATCTATTAGCATATTCTGTTATAAAACTA
ATATTTTGTTGAATTAAAAAATATCTTTTGTATATTAGAGTTTAAATTTATTAATATTATTTTTTCAATATCATTTA
TTCTGTAATGATGAAAAATATGTCTCATATATCAAAATTTGGAGGAAAAAAGATCTATAATGTAAATCTATTAG
CATATTCTATTATAAAACTAATATTTTATTGAATTAAAAAATATCTTTTGTATACTAGAGTTTGAATTTATTAATA
TTATTATTTCAATATCTTTTATTCTGTAATGATGAAAAATATGTATCATATATCAAAATTTGCAGGAAAAAAATA
TCCATAATGTAAATCTATTAGCATATTTTGTTATAAAACTAATATTTTGTTGAATTAAAAAATATATATTTTATTA
GAGTTTGAATTTATTAATATTATTATTTCAATATCTTTTATTCTGTAATGATGAAAAATATGTATCATATATCAAA
ATTTACAGAAAAAAAGATCTATAATATCAATCTATTAGCATACTAAATATGGACTGGGACTAAAGCGTGCGGTA
GGCTAGGGTAGGCAGGACTAGGGCATGCAGGCTACTAGGACAGACATCACTAATGATAGGGGTGAGTGTTA
ACATTATGTCGTGCGTATTTTACCTTGTCCCGGATGGTGTCGTCAACCAGGACTAAAGCTGAACCTTTAGTCCC
GGTTGGAGACACCAACCGGGACAAAACATGAGTCAACACTAATAATAGAGGTGAGTGTCGACACTATGCTGA
GCTACTTGTACCATAAATAGGCATCCTTGTAAGTCCAGTCCTCCAGCCAGTTTGAGACATCTCCAAGATGGTCTT
CAAGATCAGGGACACCACTACGGCAGTTTCAGGGAGATGCCGCCGGAGCCCAGTCTTCCGGCATCTCCAAGAA
ACTGGTCATAGAGGCACTCCCTAATATGTGAAATTAGGTTATCTCCGAGGCGTATTGATCTAGTTAGAGAAGTT
ACAAGCTGCAAGATGGTATTCGAGAGTAGGGACACCACTATGGCCAGTTTCAGGGAGATGCCGCCGGAGCCC
AGTCCTTCGGCATCTCCAAGAAACTGGTCATAGAGGCACCCCCTAATATGTGACACACGTGTTTGGAGATCAG
ATCTTTATGGTAAGTATGAATCGGGAAAACATCTTCATTATTTGGGAAAGTCGTAGTACAAATCGGAACTAAAG
GCCCCCCATTTAGTCCCGGTCCAGCTACATACCGGGACTAACACTCAATATTAAAAACCGGGACAGACCGGTAC
TAGCTAACACTCAGGACTAAAGCCGCCCCCTTTAGTCATAATCATGCCTAGTCCTATAGTATGCAGCTTTAGGG
TCTTCTTCGTCATCGTCATCATCACCTGCCACTCCGAGGATCCTCAGCCCAGCCATGGCATATACCGGGGGCAC
CTGCCACTCCGGCCTCGGATTCACGGCGGGCGGGGGAGGGGTCTCCTGGAAGATGATGCCGCTACCGTTGGG

CGGGGGAGGGGCCTTCTTGTGGCTCTGTGGCAGGTGGCCTTGTGGGCACCGCATGCGAGGATGGTCTCCCCG
GCAATGTA

>chr7A:37339109-37341109

AAAAAAAAAAAAAAAAACACCACAATTGGGGCAAAATCATTAAACACCCACAAAAATGCATGTTTCCGGTGGT
CTTTTGTGAAGGTTTGATAACTTTTGCCCAAATGTGGTGTGTTTTTTTGCAAATCACTAGATTGCAGCCTGCCAA
TCATCGAGTACAGTGCATTTTCTGTCTTAAGCTGTCGTAGACAATAAATTGGAGCAGATAGTGCAGTTCATTTC
TATGATTAGCACTGCATAGTACATTAAATCGTACAACTTGTCGCGAATAAAGAGTACACCAGTTTACAATTGCG
ACAAAGAGTACGCCAGTTTACAATTGCGACAAGAAGATAACAAAAAACAAAAAAGAAATCACAACAATGGGA
CACATATTTAACGTGAAAAAACCTCTCCAACAAAGAGAGAAAAAACCAAGGATATCAGCTAGCGAAACTTTAC
TATATCGGGGAGTATTTACAAACGCCAGAGATTTTACAGATGCAACTCATTCAAACCGGCGGCTTATAAGGAA
TTTATATATTGGTGGAAACCTAGGTCTTCGACGATCCATACCGTACCGCGGGTACTTTATAGAATTTGGATCAA
CATATAACACCAGTGTCAAGCTTACATATGCCCCAGTAGGAGCGATGTCGCTCCAGAGTAAACAAGCTTCGGA
GGAAAGTGTATGGAAAAATATTCAGCGTATGTGCACGGTGAACTACTCACTAGATTCTCCCAATGGCCCAGGA
TGGTCCACTAGTGGAGAATGGGGCATTAGTCCCGGTCCTAGAGGGCCTCTAGTCCCGGATTGACAGTCGGAAC
TACGGAAACGGGACTAGAGGTCTCAGCCTTTAGTCCCAGGCCTATTACGGGCCGGGACTAAAGGCTCTGCACG
TGGCAGCGTAGCAGTGCGAGGGGGGGGGTGGTCTTTAGTCCTAGTCCGTATTACGGGACGGGACTAGTTCCT
TTCTGCCGCGGAAGAATTTTACCAATTCGGCTTCCGCGGCAGCCGATGAGGGTTTCTGCCGCGGCAGAGTTTT
GGGGCCGATTTCTGCAGCGGTAGCCGAATGCGGAGTTCTGCCGCGGCAGGAGGATAGGTAGAGGAGGGTTG
CGTTTGGCGTAAAGTGACATGCAACGAAGAAGCCCATTACACACAACATCGTCATAATTATATTGATATAATTC
TATATTACATATATATATACATAAATGGAGATCACAACCCGTCTATTCGGTTACAAGTTCATTAAATCACTAGTA
CCGGAGGTATGAAATTTTCCTTCTGGGTTTATGACATGGTCAACAAGAAATCCCGCCAATTCCTCTTGAATTGCT
ACTAGACGCTTCCTTGGTTCGAGATACTCCCGCAATCTTTCCAACTATATATAAAAGAAGGAGGTTAATATGAA
TATCAATAGTCATAACGAATATTATGAATGAAACTCAACAAAAATTGTGAATATATTATTCACGTACTTCTATAA
ATGTCCCTCCGTTCCGCTCACTAACCTCGGAGCGAATTTACTCGCACACGAAGTATCCATATAGATTAGTACCTG
GTGGCCGCCTCTCGCACTTTACGAGAATAGAATTCAATCAAATAATAATCAAGCATGACATAATGGTATTGAAA
CTAGAATTGAAGAGATACGCAGATCTAGCTCGTACTACTTACATTGATCCGATGAAAACTAAACTCGTCTTTAA
ATTCACCAACCGTAGCATTGGTGAAAGCTTTCCAAACCCTGCCCGGCAAAAAATAAAGAATAGGTGAGTTATT
AGTTACTTGATATCTCAGGATATATATGAACTAAAGGGACCGATATAGTGCGGTAATGACTAAAATTACATCTG
GAGCACATCTTTAATGAATGCGTACTCTGATTCATCTCTCATTAGCGAGTCCTTGATTTTAACTCTTCAAGCGTC
AACTTCAATGGTTAGCAGAATCCAGTGATAGCTGCGCACATTTATATACGTCAGTAACTCATCAATTACACTTA
GAAGAATCAAGT

>chr7A:37354390-37356390

ATTTATTTCACTATATACCGAAACAGGTTTTCGGGTGAAATTGCATAAAGTGGTTATGCATGTGAAATAGAATT
CTAGCAAGGGGTTATTTGTGAATGTTTGTCATTAAAGTAATTGACAAAATTCTGAATAAATAAAAGGAGTTTTA
TTTAATGAGAGTTTGCATTAAATTTGGTGGAAATTTAATGGGAATTTGAGTGGGTTTTTGAGTGGAGGGAATT
GAAAGGCATTTGAGTGAGTGGAGTGTCCACCCACATGAATGTGGAAGAGGTGGGGGCAGCCCCATGGTTGG
GCGCATGGCCCCTTGGGTTCCCCCCCTCTCCCTATAAAAGGAGCCCCCCTCCCTCTCTCTCACTCACACCTCCTC
GTGAAGGATCTTTCTCCCCCTCTAGAATTTATCCCCCATGGTCTCGAACTAGCGAGATACATGTTCCTGGTGGCT
ATTTGGCAAGAAAGAGTTGTGCTTCCGCTGCATCGCTGGAGCGAGGAGGCAGAGGTTGCTCGGTGCCTCGAA
CGTGTGTGCGAGCACGGATGCGCTGTTGTTGTGCGGCGCTAGGACTTCCTCTCGGTCTTGAGATCGGCAAGAG
ATCGTCTACATCAACCACAAGTTTGGACTCATTAAGCTTTGTCAGTCTTCAAGGGTATGTTCTCTATGGATATAC
ATCCGCCCGTTGTTTGTATCTAGTAGATAGATCTTGGTACGTAGTTCCCATCATAGGAATTTTTTTGTTTTCTTTG
CTGCGAACCCTACACCCACACTCTCTCTGTCACCCTCTCATTCTCATCTCTCTTCTCCTCTTCGACCCCGTGGTCG
ATGGCGCCGCCGCCGTGCGCTGCTCGCTGTTGTGGTCCTCCCTGGCCACTGTCGTGGTGCAGGGCGAAGTTGT
CGACCATGCTCCACCTTCCTGTCCAACTCGATTTGGACCTGGAGCCCTCTTGTGGCCAGACCGGCTCCGTCCCG
AGCCCTAGGGTTTGGGGTGGTGCTGGTGTTGTCGCTGCCGCTGCTCCCGTGTGCTGTGTTGGTGTTCCTCGCCA

TCAGGCGTGGCTGTGGTCGCCGCCTCGGCGTGGTGTGGGTAGCAGCGCCCCCTTGGGGTGCTCCCCGTGCTGT
CGTGCTCGTCGACCCTTCTGCTTCCTCTTCTATTCTACAGCGAGGACCACATGTGAGTTGGTTCTCGGTTATCAC
CCGTGCCTCTACCTCCTCTGAGTTCATATGTGTGTAACTAGTGGCCATCCCTCATTAGGATTCATACTGGTTCTA
GGGTTCTTCCCTAGTTCTAGGGTTCATCCCTCTTTGTGCTTGTAAGATGATGATTTGATCTGGTAGTGATGCCTA
GTGAAGATTGTGCTCACTGCACTTCAGTGCTTTTTGTCATGATTTGATTATCTGATGCTATTGTTGATCTAATGTT
AGTGGTTTAGTAGTGTTGCCTCTAATTGGTGATAAAGCTTGGTTAAGCTGATTGTATAACTATGAAACCTGATT
GGTTAGGTTGATGCTTACTGTGTTTCTCTACTGTTAGTACTCTAGTACTGATAGTAGCACTGTCAGTGGTGATTC
ACTGCACCAGTAAATGTTGGCTTGTGCATTCATGTGCAGATTGAGGCTAATTAGTTTCTTTTAGTAACACTCTGT
ACATATAAACTTGATCCTGATTCCTGGTTATTGTTTCTAGGAAAATGATAGTTATTAGCTTCATGGTTTATTTATG
AAAGCTAAGTAATTGTTTGGCCTGATTTTAGAATTTTTTCTTGTGACACAATCTTAGTATGGCACACAAGTGGTG
ATTCTACTACTAGGTTTTACTATGGTCAGTGCTTAAGATTGGATATATGTTAAAGATAAATTGTAAGCTTCTTTC
TTTAAGCATCAATGCCTATGATGTTGGTCTGAGGCTTAGGAGCTAGATTCCTATCTTGCTGTCCCTCATGGATAT
GCTTAAGTATCAAGGTTGTTCACTGAACCTTGGATCCTAGTTGGCTAAAATGATTGGTTTGCTTAT

>chr7A:427245153-427247622

GGTGCACGAGGTACCTGATTAACTGAATCTGGGTGCCACAAATTTCCATGATGGATACACATTCAAAACATGA
CAAACTTTTATTGATATATCTTACTAAGATGAACATATAGGGAACCAACAAGCGCTACACACTTTTATTGATTAT
TTTACTTAGTCTACTTGCTTGTCGTCCTATTAGCTCAATCATGTTTATGCCTTATCAATGGAAGACAACCCGGTTC
CATCTTAAGGATAACTCTGGAACCTAATTTGAGAAAATTTAGGAGCAAATGCGCCAAACTCCTCTCCTCTATTG
TGCTTGAGGTTTTGGGCCTCTTGCCTGGTAATGCGATATGCATTGGCAATGACATCAACAGGAAGGGCACGAA
GGATTGAGGTATTTCCTGCAATGTGACTAACCATGGAGTTTGCATTGGTCTTAAATGAAATGTATTGGCATCCT
TCACGTTTTGCCTTCTTTATAACAACATAGTTTTGTGGTATGATTAATAGTTGTCCCGGACGAAGAATGCCATCG
AACACATTCTGACCATGATTATTAACAACCTGAACCAACACATGACCTTGGATTGTGTACATCACACTGTGAGC
ATTAATGTTCCAGAATGGTGAAAGAATAGCATTCTGCAATTAATCACAATGAGTCAGTAAAGAACAAAATATC
AGACTTCTATTTTGAATTTTATAAGATATACTAATAAGAATGTTTTAAAATGTAACATCATAAGTACCTGATAGA
GGTTCACTCTTGTAGCACTCATTTGCACGGTGTTAAGGATAGGGAACTTTAGACTGTTGAGATGCGTTATCCTA
CCAACACGTGGGTTGTATGTGTCAGCACGACTGGGATTTTCAATGTTCTGTGTTGGCTTAGTATCACAAAAATT
CTCCTCCAAACCATTCAAATGGCCAAAAGTTGATTGCCATCCCTGAGGTTTCCATGTCTGGGATTGCCCTACCTG
AGATTGACCAACCTGAGAGTAGGGCTCTTGTTCTTGTTGTTGCGTCACAATTGGTTGTAGAAATTGAAGGGCA
CGATTCACACGGATTATATCACCTCTTTGGTCATTTTGATTTGAAAGTCTTTGTGAGGTATGTTCACTTATACCA
AGGGCCTCGCCAAGCAACTGGAAATTGAACCCTCGGAATATGTTTTGACTCACGTATTGTTGTAGTGCCCTACT
GCCGCCAGCCAACAGAAATTCCTAAAAATTTTGAACAAGTTAAAAATTATCAAACAACATTTCACTTTATGAACT
AAACCGAACCTAGTAGATTATCCGAAAAAATTAACAATCTTAGGAGTTGGTTTAGGTATCCTTATTTACCTTTTG
TCTGGGTTCAAGTTGATTGGCATTGCTGTTTACATCAAAAACATAGAGCGCTACAACAGGCACTTGACCATGAT
TGTAGAACCAATGTGCAACCCCAGATGGTAGCGCGACAACATCTCCTTGTCTAATTTGATGAACTTTTTGGTGC
TCGTCTCTGAACTTATGCCCTTGGCTTTGATCCTGGGCAAATTCTGATTGATCAAATTGTCCAAATTGTTGTTGG
AAGGTTTCTGGGCATCCAGGAAATGTTAGACCTGCAAAGCCACTACCTACAAGAAACAAATTTTAGTTAGATAT
TAAGAACTATGGATAGTTTTTAACATTATATTTTGCACATACAATTCCTAAGTATATACTCTTAGGATGTAACCA
CCCTATTCAATTTATACACAAACCTTGAAGGATGTAGACTAAGCCGGGAGTGTTGTGATATCGAGGTAACAAG
AGGCCTTGAGGCTCGATAACACGACGGATGACAGATAGGCCAGCACAACGGAATTGCTCATTTTGCTCCTCAT
AGTACTCAGTGAAGCCTGCTTCTGACCTCTCTTGCCGAAGTGGTTCAAATGCCTGTAACCGATCAAATCTGCAT
CCCCTAACGCCTCCTTGGCGAAAGCTTTGCCAAGGATTGCTCTGGCCAAATAGTTGAGCCATGGATCCATGGAC
AAAGAGGAAAAGGCAAAAGTAAAATAAAACTGATGGAAAACTAGTAATTGCCATGGTTGAAGAGATTGGAG
AAGCTTGGTTTATGGAAATGGTGCAGGATGTGTTGGGATTTTATAGCCAAGAAAAGTGGCGTAAAGTTTATGT
AAAGATAGAGAAGATAATTAAGTGATGACATGGTCATTTGTACTGAACAGAAATGTCGAATTGGTGTAATAGA
CTCTCGGAACTTAGATTGATAATTGCAAGCATAAACAAACTATAAGTCTCTGTTGTCTCTAATAACCCTATATGT
ATTTATTTAGAAAATATTTCCTGCACACTTTCTTAAATGTTCCTTTTTTCACATACAACTCCAAACTTCTCAAAGA

AGGATGTAAAGTTTTTGTCTACCACCCTTGCCTGATATGACACGTTAAGTAACTATGTTGCTTGAATTTCTTATA
ATACTTTCTAGTTTCTGCGATGT

>chr7A:49489115-49491115

ACTTGTGGAACTACGGGAGAAGATCGGAAAATTTAGGAGTCGAACAAGTTTGAAAAAGTAGTACTGATCTGA
CACAGTTTGTACTCAAAAGCAATACCTGGACATCCTTTTGATCTAATTAATGGATGGATATGAACATGAAATGC
TCTATTACTACCGTGTCTTCATGAGAAATGTATGATGTTTCTTTGTAGGGAGATTGTATTTTTGAACTGCCAAAA
TACTCAAAATCTGATAGACAGTTCACTGAAGAAAAGGGAGGAGATCGATCGAATTGAGTCTTTTTCCTTTCTTT
GATGATCTTCAACACACCTATTTGAATTCCCTGCGAGTGAAAGATTTCTCATATGGACTCCTCTTGAGCGCTACC
AGATCTGATTTTCTTATTTCAAATATTACTTGCATAGACATGATACACATATATACCTATCTGGTTACAAAATTTC
AATTTCTGTGTAATGAATTTAAACGCGGTTGGCTTGGAATGACAGGAACAAACGTGTGTTTTGCAACAAAAGA
AACTAAGAAGCTATATGGCATGCTGGGCATCTGCATTGCATGTAAAATTTGGGTCAATAAGATGAACAAACAC
CATAATCATACCAATACCATTCCCAATTCATAATGCGGAATTTTTTACCACAATAGTAAATTCCATGCCACAACA
AATGTCCTTTCACTTTCAGCATACAACAATGCGCATGCTCCAATTGTCGGCCATTAACATTGCATGTCAATTTTG
CATGCAAATGAACATCATACTAGAACAAACACATGTCCTTCCACTTTCAGCATACAACAATGTGCATGCTCAAA
TTGTCGGCCATTAGCATTGCATGTCAATTTTGCATGCAAATGAACATCATACTAGAACAAAGTTCTCCAGAATA
AAATGACAGAAGAAACATTCCCAATGTGGAGATTTTTGTATTGCCACACTAGCATCAGGTTTGTCTTAAAAGAG
TTTAAACCATACAGAAACAAAACATGCAGAACTAACACAAGTCTCTTCCCAGCTGTACAATTTGTGTTCTTTACA
GAAACCAAACCAAAAGCATCATCATCCAGTCATCATCATCAACTCCCCACACATCTATCTGTACAGAGGGGGGT
CAAAAGGCGACATCCGTTGCGACAAAAACCAGTAGATGTGCCAAGACACACCGGGAATCCTTCCTGCACTAAA
CCAATCATCTTCCTCACTTTTTATACAGTTTGGGATTGATACCATCTTTGTCATCGACAGTATCGGCAGTTAGACT
TCACCGCAACTCTGGGCCTGCTGGTTGGTTATATAGTCTACAGACTCCCTGCCATGGAACTCTCTCCGGCCATCT
TGTCGTTGGGATTGTCGTCCTCTTCGTAGCCGAGGGGCTCCCTGCTGGCAACTGCCCCGCCATCCAGAGGCAT
GTGGATGCATATCCCATTCGGAAATGGTTCCTGAGTTTCATTCATGGTATTAGAGCACTTGCATCAAGGGTCAA
TATCATATAAATAAAACACTTGCATCACAGGGATTAGTAAATTGTGGCCCTGGATATTACCACCTCACTTCGAA
AAAGAACTATGATTAATTTGTTTCAATACTCTATTATGCATGATGTGCCAAACTGAGATGCTGAAATTTCAATGA
TCAAGCAAAATTTATATAAAACATGATTTATAGTGCCATAGAATGAACGTGGAATTGTCAAGAACACTTTTAGA
AAATGGCCTCTAAACAGATAGTTGGCTTGGCTTGGAAAATGGTCCCTAAGCTAGATGGTACAGTTCTATATACA
TGGTCAATCTTGTTGGCTTGGAATTTTGCAGCAAGGTTCATAAAGCAGATGACCCTTCACTTTCAGCATACAGT
GCAACAACTTATTACCTGATTTCCAGTCTCTGGTCCGTTGGAATGAAAAAGAATGGGACGGCATCTCTTGTCCT
CGTTCATCAAACTTGAATTCTGGAAATGTGAGATAAGTGCATTCCTTCCCTGGATCCTACCATAAGCCAAT

>chr7C:140672621-140673621

AAACTATAGGATTGTTGAATGCGACAATACCTATGAGTGATTTCTAGCTTGTATTTCAGATTACATGCAACTGA
AAAGACATATCGCCATCAGCTATTACCTTGCATATTCTATACCATTTGCGTGTAATTCTGTATATAGCACTGCAT
CTTGCCCTTCACATGTCATCAGTACTGCTCCTTGAAGAATAAGGCGTAGATGATGCAGCAGATGCTGAAGATG
AAGATGGCAATATCGAAGGTGAGTGCTGTCATGGCCTGATCGCTGAGCTCCATGTTTTTCCCACCAAGGCGGT
CTACCATGTCTGGCACGGACTCGTAGTTCTGGATGTCGAGTTCGTAGGGATCCTCAAGGCGGGACCTGAGCTG
CTGCAGCCGGCGGCTCTCGGCCTCCTGCGCCAGGGTCCAGTCATAGAACCGGCGGGTCTCCTCCTTGCTCAGC
ACGTTGTACACCTCCCGGAGCCGGATGAACTTCTCCGACGCCGATTTCAGCGGCAGCCTCGTCGTGTCCGGGT
GGTACTCCTTGGACAGCCGCCGGTACGCTGCCTTGATCTCCTCGATGTCTGCCTCAGGCGCAATTCCCAAGAAC
CTGGTGTACCAGAGAAGTTTTGGTGCTCAGTTTCCAGAAAGATGGATAAGGAGTGTGCGGTGAAGTTTGCTAA
GCTGAACTCAACTGGTAGTGTGACTCGGACGACGGGTTGTTGATGAGGTCGGCGAATCTCTGTCCCAGTGGTT
CGTTCTTGCGGCCATCGCCTCCCTTCTTTTCCTTGCCTCCCACCCAGCCTTCGTCCGGGTCGGACCAGTGTATCCT
CGTGTCCACCGTCTTCTTCTTCCCCGGCCCTGCCGTCGACGCCGCCGCCTCCTCTTGGGACGGCTCGGCGTCCG
CCGACGTGCTCGCCGCGACGATGAACCTTGTGCGGCGCCGGCTGCGTGCTTTGTGGCTGTAGAGGAAGGGCG
CCAAGGTGCCAGATGCTGTGGATGCCGCCATGAGGCGGCAGCCC

>chr7C:3208387-3210387

GCAGTCAAATTCTTCATCCCCAAATGAACAGAGCCATGGTCATATACAATACATGCATGAACAGAACGAAAATC
GTAGACCCTTCAGAAATTTTCTCACGGTGAGCACAATAATCCTCATCCCTCAGATGAACGGAGACATGATCATG
TGCAGAATGAGAAGTGCAGGTATATCTTCGCTCTAATAAAGTGATATACAACAATTACAGATTCGTTCGGAATC
ATGGCTCAACGGGCGGTTTTAGCCTGATCAGCCTCCGGACTCTGTCCTTCTCCGTGTTCTCGAGCAACCCCTGC
ATCAACAACAACAACACAACAGCTGATTACCAAGGATTCACAACAATCAGGTAACATACAGACACTGCTATTCA
TGTCCAACGGCGATAGAGTAAGAAGTTTGCATAATCGACTGTGTCAGTATAAATAAGTCAGCCCAGGGTGAAG
ACATGTGGAAATGCTACAGGGAAAGAAAATTAGCTTGAAGCTTTACCTTCCAAACAATCTCATCGAGGCACAA
ACAGATCCTCCCGTACTTATCAAGGAACAGGCGCTCACTAGGTGGTTTTCCACAGACATCCTTCACTGCCGATG
TAACAGCAAAGATCACCTCGGACACTACAAGCGGTAGACAGTGCCAAGCGTGAGGCCAAAGCAGGGTGAAAC
AGTAAAGGTTTGCAACGACGTTTTAACAAGAAAAATAACACAGAGTCAATACTTTTATTAACAAAGAAAAAGA
GAAAACTCACGAGCAAGCTCATCGTACTCATCCTTGCCAACAATGTACAGGCAGACGTCCCCAATCACAGTATA
AACAATAGAGACCGATCTGGGAGAGTGTCGACAATCAGATATTGCAAATGAGGAGTAAGAAGCGTACTCATG
GTACAACTGAAGTTAAGGCAACCGCAGATCAATATTTCTAAAGATTAATTTCAATATTTCCTGCGATTTACTGG
GCTCCATATCAGTACATGGTAATGCAAATAATAAGTAAATGAAAAGAATCAAGATGGTCAGCGATATACAAGA
GCATACTAGAAGATTGGAATGACTTTATACTTTGCATAGCATGCACTGTGATTCAATAGGATTACGAGTAATGT
AGACGCATGTCCGCTACTGGGCAGAACTGAAAACATAAACACACCAACTACTGACACTGGCATACAAGTGTGT
GCCAAAGGAAGCTACAGACTAATCCAGGAAAATGGACCACAGGAGCGACATCTACAGATCGAGTTCACCAAG
TGACAATAGGCCTGAATATGTAGTTCTTAATTGTTGAAGAGGCTTTTATTGGTAGGAAAATGACTGCACGATCT
TCTTTTTCCTCTTCTGGAGATGGACCTCCACACAACAGGGGAGCAGCAACGTTGGCACATTGAACTCTGTACTG
TTCCATTACTCGGCACCTAAATTAGCGGCATGACAAATGCTTCTGGAGCACATCTACTGGCAACTGGGTTGTCT
ATCAGTGTGTAATCGTTACAGCAAGATCATACAGAAAGATTCGCGTCTTCATCTGCAAAGTAAACTATAGCATG
CTTGTGGGAAGCAATTCTAGTTCAATCTGGTCAATTAAACCACTACTGGTCGGCACCTCGGTGGCATGGAAACT
CAGTGTGGGGCACGACAAATGTGCTTTGCAGTGAAACAGAGTGTGAAATTCAGGGGTTCTTCACGGTGTGTA
AAATCATGACTGGCAGAGAGATCTGCGTCGAGGGTAGAAGATTCGCGTGCGGCTTACTTGTGGGAGGCGACG
AGGAGCTCCTCGTTCTTGGCCCCCTTGAGGTTCTCGGCGCCGAGCTTGACGAGGAAGGAGCGCCAGTGCAGCC
TCTCCTCCGCGGGGACGCCATGGAACCTGTGATTTTCCCCCCAACAACCCACGCGACACCACTCCTCGTCAGCT
CAATCCCTATCAAATCAGCAACGGAAACCAAGATTCTTCCGCCGCGCTGAATAACCGGTAGATCTCAGGAGCG
ACGCGAGATGAGATCTCGT

>chr7C:44270999-44271999

ATGAAAAAAATGAGCAATTTCCTTGCTGCTGATTCTCAATGTTCATAACATCTCACTGACCAAGGAAATGCAAA
CATCTAAACTACATACATTGGTTCTAAAACAACTATTAACCGAACAAGTATGTTAACCAGCAACAACAACCACA
AGGCTTAGTTTTTTCCAATAGAGCAATCAACTGATTCATCGAGCGTTTCATGTTGGGTACACCTTTGATCAAGAT
AACAGATGCTCCTGTTTGTCTCCTTAGGTTGAGAGGCGCCACCCTCCAAAATCCCAGGAAAACAGCAAGGGCA
GTTCTCAGCTGACTGTCCCAATTGAAAATATGCATATACCTACAAACGGAAAATCACATACGAGTCAAATACAA
GAGTCTCATTTCTAGCTTGTAAGAAGAATCACACTGAGATCATCATCCAGTAATGTATCCATAACCAACCCTGC
ACAGTAAGTTAGAATATAGAAGCAGCAGCAAGGGGAATGAACTGAAGAGGCTAACTTGAGCAGAGTAAAACT
TCATGTGCTCTCCTTCGACTAATACTGTGTCTACTTGTATGTGCGCGTACATTGCTTCTTCGGCTAGGCAGTACT
GGGAAAACAAGGCAACGCCTATTACATGTAAGTCGACTAGTTTGGCCACAATGCACCAATCCTGTAGAGATAA
ACAGCTTGGCCTAAATGAACTGCAATCCCCCCTATATAATTTGTCCTAGGTTTTTCCATTGAAGGTATAGAATAC
AGTTGAAATTTAACTGGTCACATGCTGAAAGTCTGAAACCAGTAGGGAAGTGGGAGAATTAGCTAAAAAAAG
TCTATTACTAACTGCAAGAAAATAACTCATAAGAGGTTCCATAATTTCACCTTCTAAGTGAACTTGGTAATTATA
CACTAACCTGCTACATATGCAGAAGCATGTGGAATTAGATATTTAAACAGCCAATTGATCGATCAAACCAATGT
GAAAAAAGGTAAGCAACACTTTACCTTCTCTGCTGGCAAG

>chr7C:546053884-546055884

GACAATGGTCACATACTGTGGTATGTGACAGTTTATTAGTCACCTATGGAAGCCACATCATCAGTAATATCCAT
CGGCCATGGACTTGCAGTCGGACGGGGGCATTTTGCACAGGACGGGCAGACTCTGGGCCTTCTCCATCACTTT
GGACATCCGACCTGGCTGAAAGTGCAACATGATGTCGCCTTGGATAGTTCGTTTGATAGCCGTGCAACGGCAC
TGCGTCGGCACCTTCGCTAATTCTTCACAGCATCGCTTCTGAACATCATCGCAAGAGCTCATCCCCCATTTCAAG
GGCTCGGTCCATTTCCAGGGCTGGCTCCATTTCCAAGGCTGGGTGATGGGAAACTTCACCGACCTGCACCGAT
CCTTCACATAATCCTGGCATGCGCCTAGACTCACCTGTTCTTGCTGGCATTCTCCCTCACCGTGGACCTCGGCAT
CCTCATTGCGGACTTCTGGATACTGCGCGAATGCAGTGCTTGCTACCAGAGCAATGAGAGCTAGGAGTAACAA
GGCCTTCATGTTTTCAGTTCTGTTTTGCTGGTGGTGAAGATAGATGAGATCATAACACCTTTATTTATAGATGAG
GTGACACCATTTTCATATGATATTGTGTGTTGCTTTGGCTAGTGCTTCCAAATATAGTTGGAAATAGATCAAAGT
TACTTTCCAAGCGCATAAAATGTGCAAACTAGTTAGATATGTGGTGTTTTTGGACTTATCTCAAGATATATTGTT
AGTAGTGTTATACCAAAGCTTTAAACTTTATGCCTTTAATGAAACTGAAAATGTAGTATTGTGAAGGCTGCCCA
CAGAAAATCCAAACTAAAATGGTTAGGTATTTCATTGATGAGTAAAGCATCTAAACATATTTGTTGCTTATACAT
GTTTTTTTTTTTTGCTTTTGTCGGGCATTGAAAATCATGTGGTACTGAGATAGTTGATGTTTCGTACACTACATCT
GCTTCTTTATGGAGATATACTAGTGAGAACTAAACGTGTAGTGTTGTGCAAGTTGTTCAAGAAAGGTCTAGAC
CAAATCATTAGAAATTTTGTCGTACAATGTAGTGGTGCATGATAAGTAATACACTTAGACGCCATTGTCTTCTA
GCCATTTTTTTGGGTCATGTATCATTGAGATATAAGATGTTTCTTGTACCACATCTGCTCCTTTGTGAAGACATA
CCCTTAGATGGTCTATGTGGCACATCCACTTTTTCTTATAGTTTTGTATTGTACCAAGGAATATCATATCGTATA
GTGCTTTAAATTTTATTATTTTGTGTAACTATACTTGTAGGGTTGCGTGGGTTGTTTAAGATGGATCCAAATGTT
CCAAGCTGTGTCTTTCCCACATATTGTGTGGTACAAGATAGTGGTGCATGATAAGTAACACACATACACATAGA
AACTACCACAAAAAGAACACATCAGCAAATGATATTTATTTGTATATGTTATTATTTGTTCCACGATGGATATAC
CGAGATGTTTCTAAGATAAATAAATATGGGAAACGCTTCAAAGCCCCCGGGGGATCACGTGCTCCCGTTCCTC
CGCCACCTGCGTGCGCCACGCGTCCGTCGACGCATGCGAGGAAGCCAGCGCGTGGGGCCCCACCAGAGAACC
TTAACTCTTCACGAGATGTCCTTCTCGACTATTTTACTTTCCATCGTTCGTTGACGCATGCACCGCCTCCTCTTGC
CCCCTCCGCCACCGTACGCCGCCGACCCATCTCCCGGTTCCTCCATGAGCGAGGTCAAGGTGGCATGCGGTCAT
GGCGGTCGAGGCCTGCGTTGGCGCCCTCTCCGCCCGCCCTTTTCTAGGGATGTAGATTTGTGTTCTTGTTTTTTT
GCTACATTCGTATATTTTTTTAGCTACACACTTGTATTTGTGTTGCTACTTTGCTATTCTCTGAGTGCTACACTTGT
TTATGTGTTTTTACCGCAATAAAATGCTTCAATGATGCTACAATGTCATGGTGTTTTTGCTACATC

>chr7C:58693588-58694588

CATTCCAGGGCGGGGGCTCCCCCAGAATCCGCTCACAGCCTGCCACGGATACGTGATCCAACAAGTCTGCCGC
CTGAGGCTCCCCGATGAGGTGACGACGAAGGAGCGGTGCTGCCTGGAGCTATCGGCCACCAGAGCTTCTTGC
AAGTGCGAGGCATTGAGCGTCCTCATGGATAGGGTGGATACTTTGGACTTTGAGGGCATGCGCGTGGGCCCC
ATCGTCCTCGAGGAGCTGCGCAAATGTAACACTAAGAGGCAAAGGGACTATGCTGCCACCCTCGCCTACCCAG
AGCAGTGCGACATATGGGCCAAGCATAGCGACATGTATTGTCGCTCTCTGCACCATCAAGAGGTCGTCTGACT
TCATACTAAAGCGGTCGTGAATAAGCTGATTGCATCGATTGATGGATGGAATGCATGCATGTTATGTATGCATC
TATATGGGCTTGAGTGATATGCATTTAATGATACTCTCTTCGGTCCATATTAATGACGTCACCGCGTTTTAAACA
TGGTGTAACTAACAAAAAGGTATGACTCGTTGAACACAACGTTTCTACTGTGCACGCTATGCCACATGTATGTT
TCATCCATTGATCTATGATTCTCGTGTGGCACTCTATGATGACGTTTCACGAGAACTCGAGAAGGTGAAATAGT
GCACACTACCTAGGGTGCATGCAACATAGAAAGACAATTCCCGTGACTACTAGCCTCTTCTAATGATTAAGAGA
GATATGATGTTGTATCACAAATGCCTATAACTACACCGGGTGTGATCCCTGCTACTGCCAAGAAAAATGTAAGA
GAAAATAAGAAATAGGGTCATGTAGTGGCTACAAGGACAAGTGCGAGAGTCTAGAAATATGGGAGGACTGCT
CTAGAAAAGGCACAAGAATTGAAGAAATTAAAAAACTTGGAGGCCCCTAAAAGTAAGAGAATTCATCATGGT
CTTTCAAATTCCTTTCTTGTTCTAGATAACGTTGTGTTAATGCATAAA

>chr7C:58700139-58701139

TGCTAACCCCAACACCCCATCCTACTTGAAATTACTATATTGTACTTCCATTATCAGAATGGCATCCAACCAGCT
CATACTCTCGGTCGCCGTCCTGCTCTCCGCCCTGGCCGCCGCCTCCGCCAGCGTCGGGGATCAGTGCGTTCCAG
GGTGGGCGATCCCGCACAACCCGCTGCAAGGCTGCCACTCATATGTGGTCAGCCGAATCTGCGGCGTAGGGC

CATACCTCACCACCGAGGTAATGAAGCAGCAGTGCTGCCAGGAGCTGGCGGCCATCCCGGCGTACTGCCGGT
GCGAGGCGCTGCGCATCCTCATGGACGGGGTGGTGACGGCGGAGGGCGTGCTCGAGGGCGGCCTCCTCCAT
GACTTGCCCCGGTGTCCCAGGCAGACGCAGAGGAACTTCGCCGCGACCCTCGTGGCCCCCGGGGAGTGCAGC
CTAATGACCATCCATGGCGGCCCATACTGCCTCTCCCTGGTCGGCCGTCAAGTGGCCGTGTAACTTCTGTACTG
TCTAAACCGCTCGTGAATAAGCGTGTTGCATCGACTGATGGACGCATGTATATGTGTATGGACCTATATGCGTA
TGTGTGATATGCATTGAACGACATCCCTAGTTTCTAGTACATTTCAATATTGTGCAAGGTATGTTTGGTGGGGA
AAATAGTTTCAGACATGTAAAAAATTCTAACGCAGTAGCCTATTATTTTATCCCACCGGCGCCGTAACCCTCCCC
TCCCATGGCGACCCCAGGCGCCGTCGGAGGGTCCCTAGATACCGCCGCCGCCGACCTCGCTCCTCTAGATCCC
ATCCTTTCGACGCTGCCGCCGGCGTCGGCCGCGGTGGCGGCGGCGCCCTAGTACCTAAGGTGCTAGGGGTGG
CGGCTGCGGCAGTCTTCTTCGGAGGGGCGGCTGGGGTGGCACCGATGGAGCGGGTCGGGGGCGGTGGTTGG
GGCGGCGACCTCGGCCAGGCGGTGGCGGGTTCTCCTGCAGCTGCTTGCAGG

>chr7C:59296113-59297113

ATGGCGTCCACCACCAGGCGTGCCCTTCCACTTCCGGTGGCTCTCCTGCTATGCGGTACGTAGCTGCCTTCGCT
TGCTCCCTTCCTCCCTTCTTCCTCCTCTAGCTCCTCTCTGGCGACGCTCTCTCTCTCTCATAGACCATGATTGTGAA
AGGTGTTTTCTCTGATCCGTTCTCTGAATCCTGCAGGGTTCATGGTGATCGGATCCCTACTGCAGACCACGGAG
GCGGAGCTCTGCCAGTTCGACTGCCAGGTTGCCAAGTACATGACGTGCCCGTCGCAGGGCAGCAAGCAGCTG
TTCCCGGCGTGCAACTGCTGCTTCGCCCGCAACATGGAGAAAGGCTGCATCATCTACTACAACAACGGGACCA
TGTCGGACTGCAGTAAAATGTGATCGCAGACTCTTTCCTGTATGTTCAAGTACTACTAGTAGTAAGATTCATCT
GCATGTCAAATAAAAGAGCCTTGAATATTGTCTTCAGAGTTGGTACTTGCTACGCAAAATCGCAGCACTTGCAT
ATCTTTGGACGTGCAGTATTTCGATTTCCCACTGTCTCCCATGCTAGTACCAGCTCTTGATCATGCATATTAATTG
ATGAAGCCTTTTTTTCTTCTGGATATGATGGTAGTACCACACGTATGGCATCGGGACCAATTAGGGCAAATTGG
CAACCAATCAGGCAAGAAGTTTTGGCCTTTCTTTGACTGAAGTGTGTCCACCAAGGGTTAAGGAGCAGAAGCT
TTCCTGATTCGATAGTCACGCTTAAACGTGTGAAGAAACTGACAAATTCTGTACTGTAACTGTAAGGCTCCTTTT
ACAAGTTATACAACAAAACCAATTGGCACTTATGGTCATAGACCTGAGAAAATCAATAGACCTCACGCCAAAA
AAAATATTCAAGTGGTGCTTCATAAGTGCTTGCTCACGCACGCCGATGTACCATCTCGACCTGTTACAAATATCT
TTGCTGCCGTACACAACGAATTCTGTACGATTCTCG

>chr7C:78808675-78810675

AGGGAGTCTGGGAGCCATTTTGTCATTTATAGAACTAACTTAGCCTGTTATTATGTCGCTCGATTCACCCCATCT
GTGAGTTGTTTTGTTGCTCCAATGACTGCAAATGGTTTGGTCTATAATGTGGAATCTTTGTTGGATAGTAGCTTC
TTCAGGGTCATTCGTTTCTCCAAAATCTGCGGTTTTTTCTGGTGCCAGTGATGTATTGTCGGTTGCTTTGGTTTA
GATGGTTTTTGTTTTTTCGTTTGTTTTGGGTAATTTCATGTCGTTGTCTAGTGTCACGTTGGGATGCTTTGGTGG
CTTCAGTAGTGCTTGTCGCATCTTAGCTTTCTATTTTTGACCTTGTGTTTGTTTAATCCAATGCTGTCCGAGAGCT
GCACGATTATCTTTTGTCTTGAGCAATCCAGGTGTGTTTTTTCTAGGATAGGTGTCGCAGTAGCTGATAATTCTG
TGTAGTGGCTGTTTTCTTAATTCCCGGGAGTAGCACAGCACTAACAAAATGATGTTTCTGTTTGCGTGTGGACT
CGTTGCCCGTTGTTTAGTGTTGGAGAGATTCTTCTTGTAGGACTTGGCCAGACAGCTGACCAAGCGAAAAATAT
GTGTATCCGGTTTCTTGTGTTCCGAGAACACGCTCCAGGTTCTGCTGAGCTGAGTATTCGTATTGAATTGCAGG
TTACCAGCTATCACCAGGACGCTTCTATCCCATCGTGCATGTTTGAACTTTTAGTGTTACTCTGTTTGAAGTTACC
GCGTTGTCTCGGTCTGCTTGATATTACCACGTCTCTGTAAACGAGGATAGGTTTGAGGGCTGGGACAAGGTTG
AAAGAAGGTTGAGGCTAGTACAGTAGAAGAAGGCTGTAGCTTTGGAACGGAACCACCGAATCATGGACCGCT
GGGATGCATCATTCATTCTGCCCTGGTGCACGTCGCCTGATGCCGCCCGTAATCATGGCGCCGCGCATGTTACT
ACTAGGCTGTTTTGACCGCTGCTTTGCTTTCCCCCCAAACCTCGATTACTTTTCTCTCATCAAGAGCAGAGCCCC
AAAGCGGAAACTGCAACAGCGTAAAAGCCCCCCCTCCCCTCCCCTCCCCGTCCCCTCGTGCTCGTATTATTATCA
AGTCGGGCGCCCCGAGCGAGGTCGTTGTCGACCAAGATAGTTTTGCTCATCCAATCTCTTCACCTAACTGCTTC
CGCCGATTAACACCCCATCTGCTGGAATGTTGTCTCGTCATCTTCTTCTGAATTAAGATAGAAATATTCTTACGA
GCGCGCGCGTCTCCTCCTCCCTGGATTTAGTAAACACGAACACGCGTGCGCTCGCGTTCATAAAAGAAAGAAG
CGCTACCTAACAAGGCGGGCGGGCATGGATGTCACCGAGGATGTAAACACTCGCGCTCACTCGTTACTTTCGC

CATTATTATTTCCCCCTACGTCCGCATCATTCGCTCTCACGCCTTCACGCCAGGTCGTTACCTTCTCATGCTAGCT
TTTCACACATCGCAGCAGCTAGCTAGATATCCTGCAGTTGAACGGCCCGAGCTAGCTAGATCGAGGGGAGTTT
ATAGCATCGTAGAATCACCACTTTTCATGTCAAATTCCCGTTTAGTACAACTTTTTTTAAAAAAATTCGGTACAA
AATCCCAACTCATTTTTTGGCAAGTTTTTTTTAACAAAACATTAATTGCACATTGATCGCAAATTGAGGTTGTTTC
TGACGGGTGGGACCTATTTGTCGGTGTCAATTTGCTTAGTTTGACTAGGGCCCACTTGTCAGCCTAATTAAAAA
AATCAAGTTTACTTTTTTCCATCTCTCTCACATGTTTCCTTTGATTGTACACACGAATACCAGAAAGATCGATGG
GCCATCGGAGCAGGTGCACAAGGGCATGATCGTCGTCGACGAGGAGAGCATGTGCCACGCAGTGGCGTAGG
GGTGTGGCCGCGGCTTCACTGGCGGCCCTATGCACGGATCGGCCGGTCAAATAGATGGTTGTCGTCC

>chr7C:89531985-89533054

GCATCTAGATATCTTTTAATGTATTATAGGTTCACTAGAAGGGGGGAGTACAAGATTTCCTTCCATGCAAGAGC
ACTTTATTCACGAAGGAGTTTACTCTAGTCGAATACATCCATTGCCAGCTTTACATGTGCATACGAATGTATGCA
TCCAGTTGCACTTATTAAAATACAGGATTGGATAAACTTATTCTTGTTTGTGTGCTTGAGATCACTTCCACCGTT
CCACGGCCGCTTACTTAGCAGCTCATGGGCAGAAGATGACCTGGTAGTTGGTGCCGCCGGGGCAAGTGTAGG
TGCTGGTGGCGTCGTCCTTGGGGTAGCTGTAGGCGTCAGAGCACTGCCCCTTGAAGAACCTGGAGTAGTCGGT
CGGCCCGCAGTTGTCCGCCGCCGAGCCGGTGCAGCAGTACTTGTCCTGCTTGAACACCGTGCAGGCGTTGTTG
CACCCTCCAGTGGCCTTGAGCTCGTTCGGGCACTGCGGCGTGATCACCTTCGGGCACCGCGGCCCGCCCTTGG
GGCACCCGGCGCCGCTGCCGGCCGGCAGGAAGTTCATGGGCACGTTGAAGCCGTCGATGAGGGAGATGTCG
AAGAAGTCGAGGTTGTTGAACTTGTTGAGCCCGAACTCGGCCAGCGTGTTGGGCGCCTGCCCGTACTGCGTGC
ACTGCAGCTTGCCGCCGCAGTCGCCCGTCTGGCACCGCCCGTTGCCGCTGCCGTCGAAGTTGCAGCCCGTGCG
CGCCCACACGCGCCCGCTTGTCGTGCCGGCCGGCACGTTGATGTTCCATGTCTGCCCCGGGTCGAGCTTCCTGC
CCCCGCCGACCGGCACGGCCGCCGCCCACACCGTGTACTGGCACTTGTTGGTGACGGTGAAGGTCGCCGCGTC
GGCGGCAGCGACGAGCAAGAGGAGGAGCACGGGCAACATGGAAGAGGTGGAGAGGGATGCCATGGTGGTG
GTGGTGTTGTGGTGAGGAGGGAGTTAGAGATCATGGATTTATAGGGTCGGATAGTGATGAATCCAACGTTTG
CAGCTTCTCGACGTGATTGCTTTGGCAGCTCACGAGCCAGGCCGTTAG

>chr7D:14246503-14247712

TTCAAGCCATCAACCATCTTGACATATAGCAAGCCATCAAGCAGTACATGGTACTTTCAATAAGAGGTACTTGA
CATCTTCAAATTATGGACGCAATTACACATCACAGAATGCATGAACTGGGTTTACGCTTGACCACAACGGTTAC
TTATAGCATAGCGACATTTGACCAGCCGAATCATAGCCAAACTAGGAGGTGCTTTATTTCGGGTCGGTGAAGA
ACTTGTTGATTCCTGGCATGATCTCAGGGGTCTTGTTCTGCACAAAAGCTCGGAGACCATGCTCGGCATAGCGC
CTCCCCTCCTCATGGTTCTCTAGGACGCCAGCTGCCCTCCCCACCTTGCTGACCCTGCAGAAGAGCATGTTTTGT
GTTAGCAAGATCAGACATATTTCAGGTTTTCTAACAAGATTAAGATGAACTGCCACTATTGAAATAATCAGTAC
ATGTAATGCTTATTAAGATCACCTTCTCATCATAGAGGTGCACTAAACAATTAATTGCAGGTAAAGGTAATTAA
TTGCACGTAAAGGTAAAATACAATACTAGTCCATTATCCATTGGAACACACAGGATAACACACTTGACTTCCTG
TCTACGAAATACGATTGGTAGAACATCCAAGGGCTTCCCCATTTCCGAACAAAAAAGAAGTGATAGGTAGAAC
ATCATACTCGGAACATAAATAAACTATCCTACAAATTCTGGGCGCAATTCCACTAACAATCGCGCAAACAAGCA
AAAACACTCTTATAAAAGGTCAAATGTTTGTGGATTAATTCCCCCCTACCCAAATGAAAACCAGCAACGAATTC
AACACCCCGCGACCAAGAACTGACCCAAATCCAGCGGATCTACTCTATCTAGTCCACCAATAAGTGATATATGC
ACAGCGCCAAGCATACTATAACCCAGATCCGTACCTCACTTCGGGGTTGCCGGTGATGTTGCGGAAGAGGCTG
AACACGCAGATGCCGACGGCCACGCCCGTCGCCGCGAACAGCGGGTACACCTGCGGAGCCGCGAAAGCGGA
TGAGCAAACAGCAGGAAAAAAGAAGCGCGAGGGAGCGGACTAGATGACCGGGCACAGATCTAGGCGTAGA
GCTTACCTCGGGGCGGACCCACTTGCTGGCGGCCATCGGAGTCGCGGGGAGGACGCTGCGACGGAGACTGC
GGCGGCGGAAGCTAAGAGAGAGGAAAGTGGAGGAGAG

>chr7D:326829869-326832072

CCATGGCAATTACTAGTTTTCCATCATTTTTATTTTACCTTTGCCTTTTCCTCTTTGTCCATGGATCCATGGCTCAA
CTATTTGGCCAGAGCAATCCTTGGCTAAGCTTTCGCCAAGGAGGCATTAGGGGATGCAGATTTGATCGGTTAC

AGGCATTTGAACCACTTCGACAAGAGAGGTCAGAAGCAGGCTTCACTGAGTACTATGAGGAGCAAAATGAGC
AATTTCGTTGCGCTGGCCTATCTGTCATCCGTCGTGTCATCGAGCCTCAAGGCCTCTTGTTACCTCGATATCACA
ACACTCCCGGCTTAGTCTACATCCTTCAAGGTTTGTGTATAAATTGAATAGGGTGGTTACATCCTAAGAGTATA
TACTTAGGAATTATATGTGCAAAATATAATGTTAAAAACTGTCCATAGTTCTTAATATCTAACTAAAATTTGTTT
CTTGTAGGTAGTGGCTTTACAGGTCTGACATTTCCTGGATGCCCAGAAACCTTCCAACAACAATTTGGACAATT
TGATCAATTAGAATTTGCCAAGGATCAAAGCCAAGGGCATAAGTTCAGAGACGAGCACCAAAAAGTTCATCAA
ATTAGACAAGGAGATGTTGTCGCGCTACCAGCTGGGGTTGCACATTGGTTCTACAATCATGGTCAAGTGCCTG
TTGTAGCGCTCTATGTTTTTGATGTAAACAACAATGCCAATCAACTTGAACCTAGGCAAAAGGTAAATAAGGAT
ACCTAAACCAACTCCTAAGATTGTTAATTTTTTCGGATAATCTACTAGGTTCGGTTTAGTTCATGAAGTGAAATG
TTGTTCGATGTTTTTTAACTTGTTCAAAAATTTTAGGAATTTCTGTTGGCTGGCGGCAGTAGGGCACTACAACAA
TACGTGAGTCAAAACATATTCCGAGGGTTCAAATTCCAGTTGCTTGGCGAGGCCCTTGGTATAAGTGAACATA
CCTCACAAAGACTTTCAAATCAAAATGACCAAAGAGGTGATATAATCCGTGTGAATCGTGCCCTTCAATTTCTA
CAACCAATTGTGACGCAACAACAAGAACAAGAGCCCTACTCTCAGGTTGGTCAATATCAGGTAGGGCAGTCCC
AGACATGGAAACCTCAGGGATGGCAATCAACTTTTGGCCGTTTGAATGGTTTGGAGGAGAATTTTTGTGATAC
TAAGCCAACACAGAACATTGAAAATCCCAGTCGTGCTGATACATACAACCCACGTGCTGGTAGGATAACGCAT
CTCAACAGTCTAAAGTTCCCTATGCTTAACACCGTGCAAATGAGTGCTACAAGAGTAAACCTCTATCAGGTACT
CATGATGTTACATTTTAAAACATTCTTATTAGTATATCTTATAAAATTTAAAATAGAAGTCTAATATTTTGTTCTT
TACTGACTCATTGTGATTAATTGCAGAATGCTATTCTTTCACCATTCTGGAACATTAATGCTCACAGTGTGATGT
ACACAATCCAAGGTCATGTGTTGGTTCAGGTTGTTAATAACCATGGTCAGAATGTGTTCGATGGCATTCTTCGT
CCGGGACAACTATTAATCATACCACAAAACTATGTTGTTATAAAGAAGGCAAAACATGAAGGATGCCAATACA
TTTCATTTAAGACCAATGCAAACTCCATGGTTAGTAACATTGCAGGAAAGACCTCAATCCTTCGTGCCCTTCCTG
TTGATGTCATTGCCAATGCATATCGCATTACCAGGCAAGAGGCCCAAAACCTCAAGCACAATAGAGGAGAGGA
GTTTGGCGCATTTGCTCCTAAATTTTCTCAAATTAGGTTCCAGAGTTATCCTCAAGATGGAACCGGGTTGTCTTC
CATTGATAAGGCATAAACATGATTGAGCTAATGGGATGACAAGCAAGTAGACTAAGTAAAATAATCAATAAAA
GTGTGTAGCGCTTGTTGGTTCCCTATATGTTCATCTTAGTAATATATATCAATAAAAGTTTGTCATGTTTTGAAT
GTGTATCCATCATGGAAATTTGTGGCACCCAGATTCAGTTAATCAGGTACCTCGTGCACCATCCAAAGATCACA
GCTGCTGGAACACGATACACCTGATATTCAAATCCAAACCATAATATTATAAATATCTCCAAATTGGTTAAACTT
ATTACACCAACAACGAGGCAAAAATACACGCATCTGGTGCATCAACTATGTATAT

>chr7D:424213763-424215763

GCAGTTATACATCTCTATGGATTTTTTTTACTACGGAACCGGACCAATGCGCCCAGCCTTTTACACACATTCAAG
ATACAAATGTCAGGGTTGCAATGTCAGCACACCACATCTAAGGTAAAAACAATTCACAGCAGGCTTCGCACCA
CGAACCACAGACCCTGGAACTTGACAGCATCGTCAGTTATTCCGGACATCCAGGGGTATTTTCTTCTTTTGATG
TCGTGTCAACATAACTGTAATTCTGTAGGTGACATCAGTTGCACAGGTTTTCTTGGTGTGTCTGCCACTAGTTTG
GACGGTTCAGAGCCATGAACTTGCACTGCTCACTCATCGCCTGGAGCTGAGCGTCCTTCTTGTCTACCATGGCA
CGTAGCCTCACGTTTTCCTCCATCACCTTCTGGACTTCGGCTTCTTTCTGTGCTTTCTCAGTTTCGAGCTGGTTCG
TCCTTGCGACTAGCCTGTTCACAAACATAAGAAGCTCAGTACTGGATTTTGGCTATAGATAGCCCATTGGATAC
TTGCTAGCTGTACATGCTAGACTGGCCTGACTAATCCTAAATTACCTACAGTGCAAGTTGTCGGTTACTAGTGA
ATGCATGATCTCTGGTATACGTAAATCTGAGCCAACGACCTATTTTTGGTGGAATGGAGGTAGGAAAGGATGA
GGACTTTTCTTTTCTGAAGAAAAAGGCAGGAGCACTGCCTATTGATTAAGGAGGGAGTGGTTAATATTTACAA
TGTGACTCCCTTTTAAAAGGAAACAAGACCTACTCCCTCCGTTTCAAAATATAAGATGTTTTAGATTTGTGAAAA
AAATGTATCTAGACTTTTTTTAGTGTGTAGATTCATTCCAAAGCTGAGTGAACCTACATATTAAAAATAGTCTAG
ATACATTTGCTTCACAAAGCTAAAACATCTTATATTTTGAAACAGAGGGAATAGAACCTAACAAACCAAAAAAA
CTTATTGCCCTCACTTGCACTGCCTGGCAAAGCATAGTAAAAGACCTACGGTTGATCTCTTCCCAAGATAAATG
AGCCGCCTGTTCATCACAAGACTTCTCTGTATAATTCTATGACTATGATATGGTACTAGTATGAAGTACACCGCA
CAAGAAGGAAGTAAAAGAAGACAACACAGGCATCTCATTTATCCACGGATGTGGTCCGGGAGTCTTATGTACA
TAAACTCCTTTTCTTGGTTAATTGAATAGCACAGCTCCAATCCATTTCTGCCGGAAAAACAACTGCTTGCATTCC
CTTGAGTAGATTTTCCAGTTCAAATGCAATGAAAGCAGTAGAAAGATCAAAGACTAATGATCCAAAATGTCAA

TCTGGAACTGAATGACCTACCGGTCAGAAAGCAGCAAGAACTGAACTTTTGGACCAAATACACCAACAGAAAA
CCAGATTTCTACGCTTACATAATCTTTCTGCATCTACTTGACCACTAAATATCCATTAACATTTTGGACATGATCA
GAGTGCATAAGTGCTTCATTTAGGAGAGCAGTACATTAAATCTATTTGAAACATTTTATGGCAACAATCTCTAT
CAGATGTCAATGAGTGATAAAGAGTAGTAATTGTTTTAAACAATGGTTTTGCATGATATATACATGGTTAACAT
AAATGTAGAAGGCATCAGCAAACATCTTACCAAATCCCCCTCATAAAAGTTGGAGTGGATGACAGTATGTCAA
TATGTGATAGCGAAAGAAGCCTGGCCATGCCATTTAAGTGCTAATTTAAAACAGAAAAGATGTATTGTGTGCC
CTAAAGTTCATAGATAAGGTAGAGCCCATTAAGTCAAAAATAAATTGGAATTGTTAACAGAAGAATCATATAA
TACTAAGCAGCTTAGCATCATATACCTGCAGAAATACTATTTTTGTTTGCATCATGGAGATACCAGTGCTGACC
ATGTTACAAACTAGTTCAATGCATAGCTGATTGAATATGTTGTTAAGACAGTAATCATGGTAACTAACAGTCTG
C

>chr7D:439952167-439954167

GGAGGGAAGGCCAAGCCGCTGAAGCAGGCCAAGGTCGCCGAGAAGGACTACGATGAGGTACGACGTCGTTA
TGCTCTGGCCGTTGCTCCCGATTCGCCGCCCTGATTAGCGATCTGGCCTTGTAATTCATTCGAGGTTTAGACGA
GATTAGGTCACTGTTTGACCGTGGCGTTAGATCGGTCCGTGTTGGGTTTGTTGCCGTAGATTATTTGTTGGGGG
AATAGAAAATTGGTGAAGTTGCTGTGTATCTGAACCTGTTGTGTTGCAGTAGAAGCTTTTTTTGGGGGGAAATT
AGGGGTTCGTCAATGATTGATCCTAGGTGAGTATAAGCAATTTGCTGGGAAGGGGAATTTGGCGAATTGCTAC
TGATTGTCCTGGTTATGCTGGATGGGAGGGTCACATAGCATCTGAAATTTGTGATACTCGAAATGTAAAAAATT
GTGTGTGTGAGCAAGTGAGCAGTCTGATGCACGGTTCTATTCTAATTAATGGTTAGGATTTCGTGTGTATTTGC
CAACATTAATCAATCAAACGAGCGCAAACTTGCCCTCTCTATTTGTGAGGCTACACAATGCAGTCATGGCTTGT
CTAAGGTTAATGTGCTAAGTAGAGTTTGCAGCACAACCTGGGTTGTATACCTGTGTCCTTGTCTCGTTCCTTTCT
TTATGCCATTGTTACTGTAATCTGTTTGTCGGATACAAACAAATGCACACTTATAGTTCACCTTAAAACTGAATT
GTATTAGGTTATCTAGTTCTGGAAAAAAGAGATGAATTCATTTAGTTGTATGAGCCTTGGTGTTTCTTTGTTTGA
TTAGTTACAACTCAAGTCATCTTTTACATGGCACTCCGACCATACTACCATTCTCTTAATACATAAGCTACAGCA
GTGCAAGTATTTTGATGTGTTGTGAGGGGAAAGGAGGGTGATCTTATTTTGATATCAGCCTTGTAAGACAAAG
CTTCTTTATTTTAAAAATCATTCCATGGACAATATAAGTGATCATTCCAGCTACTTCAGAGACCAGTTACAGTTG
ATCTATACTTATCATGGTTTCGTTTCTTGTGCGATTGCAGAATGACCTGGCATACCTCCAGAAGAAGAAAGATG
AGCAAAAGGTGAGACCAGTATAGGATATCTCGTATTTCCTTGTACTCTGCTTATCTTATGACACTTGTTGCTAAC
TTGAACTATATTTATCTTGAAAAACAGGCACTGAAGGAGCTGAAGGCCAAGGCAGGCCAGAAGGGTGCACTC
GGAGGATCCGGTCTCAAGAAGAGTGGGAAGAAATGAGCCTGCCTACTCCATCTGTAATGCCCAGAAAATGAT
AAACCATCTATCTACCTGTGTTGCGCTTCACTATCTGTGGTTGTAAGAAACTACCTTTCAACTGTGTCGAGCGTT
AAGCTTCATCATCGCGGTCGGTTGCTGTAGCCTATTGTGTTTCAGCCTCTCTTCTGCATGGCTATGATACAGATG
GTTTCCTTAGCTAATAATTTGATGTGATTTGTGACGTGATATGGGAGCTCTGTTCCTCTGGTGATTGTTTGCTCC
AATTCTGCTCCATTTTCACTTGCTGCTCCCACTTTGTTAGCTGGTTGCCGATATTGTTTGTTCTGACTGTGCTTCA
TTTTCATCCGATTCGTAACCAGCATTGGATTTTTACCTGCCTTCGTCCCTGTTTCCTCCGCTTACATACTGAATTTT
GGAACATCGGTATTATATAGATCAGTTGTGTTCCAAAACTTGGACCGAAGTTCAAATACTGATTCACTGACTTT
GTGTAATTGCTGCTCAGACTGATGCTCGAATCTAGTACCCCCTCTGTCTCAAATTATATGTTAGAGATTTGTCTA
GATTCACATGTATGGTGCGTTTTAGTGCATAGATGTGAATTTAGACAAATTTTTGACATCTAATTTGAGATAGA
GGGAGCACAACTTGAAATTCCTCATTATGGCCGTGTCGTGCAAAACTTTGAATCTACAACTTAAAATAAC

>chr7D:475711141-475713141

TACAGACTACAGAGGGAAACCGTGCATATATACTATACGTAGCAAATAGCGTAGCCCAGATTTTTGCTTCTTCG
TTCTTAGTGTGCGAAAAAAAATGTGTGTGGTTCGTGTTTGTTGTAGATTTGTAGGTTTCGTTCGTACTTTGGTTG
ACCTCACTTGGTTGTAGAGCGTAGACAAGGTGGTCAGACAGATATCATGTATACATGTCCTGAAACTTCTCTCT
CTGAAGAAAGTAACTGCGACGCACGCACCTCGCTTACGATTCCGCCTCCAGGTTTCTGTGTGTCTTGTGATCAG
GTCCGTGCAGGCACATCGACGAACGGAAGGACGTACGAGCGTGCCTGCCGGTGTGTCCCGCGAGCGGCCGG
CAACCCGCGCAGGCGTCGATCCGTCGTGGAGCACTGGAGCACGGCAGGCGGCGACCACGCTGCCATGGCAG
GCAGGCGTCGGCATCGATCGCTGGGGACTCGTCCGAACATGGCCGCTGCGCTGCGCTGCCATGGCACCGTGG

CAGGCAGAGAGGGCAAGTGTTTCCTCAGCTCTCGCCGTCGGCGCTGCGCCCTGTCGGGGTACGACCCCTGGG
CAGAACCAGGACGTAGTGGCTGGGTGAAAACGGACCAGTTTGATCCTTTTCTGATACTTGAACAAAAGATGAA
CAGCTTTTGAAACAATTTCAGAGATTGACCATTTCGCATGGCGCGCTCCCCAGGACCCTTGAGAAAAGAGCCA
GTTCTGCCTATTTCCACGGTGGCCGGTGCCGTGCGGTGGCATTGGCAGGGTGGTCTTTGAATTCTTGGGGTGC
TTTTGGCGGCGGCAGTAACGACACGGTGACCGTGCAAGGAAGGCTAGCTTGGGCCGGGGCTTTTGGGATTTG
GTCCCTTGATTTGCAGCTCCAGTAATTGTGTTCGAAAAATATCTAGTACACTACCGAGTAGTAGAGATTTGGCA
CGATTGATCTCATTCCTTTAAAAAAAATCAAAAATCATGTTTCTGAAATTCTAAAAAGTTCTGAAAAAATATCCG
GCTGTAGCTAATGATTTATCAAAACGAACATGTAAAAATATCAATTTCCAATATTTCGTATTATGAGCTACATAA
AAATTGATAAAAATGTGGATCTTAGTATGTATAGTTTCAAATCTTCAAATTATATTAGTAGATTTTTTATTTTTGT
GTAGCCTAGAATATATAAAATTTCAGGTTGCCATTTTGCGTGCTTGTACGATACTATGTTGACTATGTCCAAAAT
TTATTTTCCAAATTTCTTGAAACTTATAAATATGGTTTTTATTTTATTTTAAATGAAGGGAACACTAGAGCTTGAG
AGCCGTACACACTTCCCGTTTACTACATTGGTTTCAAATTAATTGACACTGCTCTTTCTAGATGCGTGTGTATCTT
AATATTAAAACTTGTTTAGATACATGTGTATCTAGACAAAGCTACGTTAATTAATTTAGGCAGGAGGATACTAC
AAGCCGCTTGGGAGATAATGAAGTGTTTATATAGCTTCCTAGTGTAAATTGTCTATGTATGAGGCCTTATACGG
TAGCTTAGGCTATAGGTTCACTAAAAAACTTGCGCAATTAATTTTAACAGTGAGTTTTTCATGTTGGGGTTTCCT
TTTTTGTTTGATAGTTATGAACCTTTGATAACTTCCATATCCCTTTAAATTGCATGAAGTCGAGCAAAACATGAA
AAGACATGCATGTGGCAGAAGGAGAATGTGAGAAGATTTTCTATGTCCCCAACTTGGATAGTCTTTCGGCGAT
GTTGGTGCTTTGAGGAAGGCTGGTAGAACTGTTGTTTTTTATCTTTTTGCTAAAATTGACTGAGGTGACATGCA
GTTTGCTTTCATGTTATTCCCTTGTGAAGTTCGGAAAATTTCATATGACATGTTGCTACTTACTTTTGTTTTCCATT
TCATGTGAGTGGATTTTTTAGTCCTCCTAGCAATAAGTAGAACGTACAAGAACTTCCAATTACACATATACAAT
GTT

>chr7D:477214056-477216056

GAGCTTGGCTCGCAGGGGAACAAGTCTACTCCCTCCGATCCATAATAATTGTTATTGATTTAGTAACGAAACAA
CGAGCAACGGCAGATGGTATGTTCGAAGAAGAGATAAGTTACACAATTTTGCAGTCATACAAACACTCCATCT
CCTTGATTTTTTCAAAGCTAGGCGTCCAATTTATTCTTTCCATTTATTCAGCTGGAGCAGCAGCGCAGCAGCTAA
GCACTAATGGTCCGGCCACCGTCCACGAAAATGACCTGGCCGGTGATGAAGGAGGCGGCTGGCAAGCACAAA
AACGACACCGCCGAGGCGATCTCCATCGGCTTGCCGCTTCGGCGCATCGGCGTCTTCGAGTGCTCGTCCTCCA
GTTGATCTTCAGTTGCCTGTGCACAAGATAAGACAGCCAATTCATACGAAGTTCCATTCCTTTTACACATGATAA
TTAGGGTGCTAGCTAGAGAAGAAAGAGGCGTGTGGACTACTTACATCTTTGATCATATCAGTGGTGACGAATC
CTGGGGCGACACCGTTCACACGGATCTTTTCCGGTGCCCACTCCGTAGCAAGGCTCCTTGTAAGCTGGTTTATG
GCTCCTGCATGCATGCCAACATAGATTGTGTTACTGTAATACAGCTTGTCATTTTAAGTAGGACCTAGATGAAC
CTTTTTATTAGTTCCCAAATAATTAAGATTATTTTTTTTCTCTTTGAGCCCTCTTCTTGATAACCATGAAAATGACA
GTTGGCTTGGTCGTAGAAAGAAGTCATACCTTTCGTGACATTATAGATGGCGGCGCCTGCGTAGCTGATAGTG
CCTCCAATGGAGGACATGTTGACGATGCTGCCTCCTCCGGCGTTAAGCAGAAGAGGTCTCGCGAGCTGGCTGA
AGTGGAAGAAGGACTCCAAATTGGTGGCCATCAGATGCGAGTACTCATCCGCCGTACACTCCGCAGCCGCCTT
GACGAACAGTTGCCCTGCGTTGTTTACCTGCATGATGCATGGAGCAAATTATATGAACATCTGTACGTATATGT
CGTCGTCTTCACAAATTTTTTTTTTCGATTTCGAATAGGATGCGTATATTATATTGAAAAAGGGATGATTGGTGA
GAGAAGCTAGCTAGCTTACGAGTATGTCGAGCTTGCCGTCGAAGGTTTCCTTGACGGTCTCCATGAGCTTCTCC
CTGTCGGCGCGCACGGAGACGTCGCAGACGGAGACGGTGACCCGCATGCCCTTCTCCTCCCACCGGCGTCGG
CTGGCCTCCAGCTCGGCCGCGTTCCGGGAGCAGGTGTGCACCCGCGCCCCGAACCCGGCGAGCTCCTCGACTA
TGGCGTGCCCGATCCCTTTGCTGCCGCCGGTGACGAGCGCCGTCGTGCCGGCGAGGCTCCACCGCTCCTCTCT
GCTCTGCTCGGCCGCCATCTCGATCTCTTTCGGCAGGAAGACGGGCAAGATCAATACAATACGGCGCTCTCAG
CTAGGCGTCGTCGAATGTTAAATAGAAGCCACACACGTTGACAGGGACAAGAGTACGAGGTGTTGAATGTTA
AGACGGAGGGATTGTTGGACCGATCTCAGCCGCAGCAGGGCCTCAGAGAGGTGGATGCAAAACACAACAAG
AAACTTAAGACTTTTCTTGGCACGCTGAACGCGGGATAAATCTTTGCTTGGTGGAAAAACTTTGCAGACCTTAA
AAGTTTCTAGTCAAACTGATCGTGTTGCCTGAGGAGCAAGCAAAGCAATCAGCAAAGACGGAGGAAAGCGGC
CTGCGGTGGGTGACTGTTTGTGACGTCCTTGATGATCCGAGTGAAGTTGTTTTTTGTTGAAAAATCCGAGAATG

AAGTATTCAGTGACGACTGGGCGTGTTAATTTTTTTCAGAACTTTCTTTTAGGGTGAATTTTATTTTTGAGGGTG
AATATTTTTTTCTAGAAAACTATGGGAGGGCATGAGGAAGGCATAGAGCCCACAGAGATCGTGGGTTGGCCT
ATTCGCATTCTGGCTCA

>chr7D:74441048-74443048

AAAAAATATTTCAAAAATAACTGCTCCCTGGAAGAAATGCTTTTGCAGCAGAGGAGGAAAACAGAAGAAATTC
TAGGCAGGGGATGATGGGAGACAACCCCAGTGGCAAAATGTACAACAGAAAGTCCTTATTTCGATCGCTTCTG
CTTAAAGACTCAACGTTGTAATAAAGAAGAAGAAACAATGGTCATCAGTTGACGACGTCCAGGACCAGCTTGC
GGGACTTGAGGACCGACCACCTCATGCGACGGTAGTATGCGGCTTGGAGAAGCGCAAGTGACAGTAGGAAG
ATCCATTTGACGCCCATCTAGAAGAGTTGGTATTAGGCACATCAGAACAGTGGCCAAGTAAAATGGTGTATAA
CGTTTAAATAACCTAGCTGTGAAGTAGTTTACCAGTTTCCTCTCTTCCATTTCAGGCTCAGCTGCCCACGACAGG
AACGACACAACATCCTTGCCCATCTGCAATTGAAATATAATGCTTTACTGTTAGCCCAACGAGGCAACAACTTC
CACATGAAAGGCTCAGAAAAGGATAAAAAGACTTGCCTGGGCTTCAGTTGCAGGAGTACCATCTTCATACTCA
ACAGCTCCATCCATAAGCATCTTGGGCATGGCTATGGCACCACCAGGGAAGTAGGGATTGTAATGCAGACCCT
CACGAATCTAGAATTTAATCAGTCTAAGTCAGTTTAAAATACAACAGAGAAAAAATATAAAAAAAAGTAAACA
GTGTTGATATTACAAGAACAATTAAAGTTTGCCTGTCTTGCTAGATGATACACAAAAAAACATTTTTTTTCAAAA
AGTATATGAAGTAATGAGACAATTTGTAGAATATTCAGCAAAAAGAATCAACATTAGCACTTGGAAGGGCAGT
AATTAAGTATTTTCAGACTAGCCAAGACTCCTGATTCTGGAATCAAACAATGATGTTGTGCACAGTGGCAGAGC
TAGCCGACAAAGATTTTTTAGGGGGGGGGGGGGGGGGGGGGGCAATGGCTATTTTCTTTTATCTAGGTCCCTACA
TATTTTTTCCCTTCAATGATTTGTCAAAGTCTGGGGGGGGGGGGGGGGGGGGGGGGGAGCCCTGAGTTGCGCAT
AGCTCCACCAGTGTCATCCGCTACAGTTCAGAACTCAAACAATAATTGACCAGCATGAAAAGCTGAACTAATCC
TAGATTAACAGGATGAAATATCACTAAACAAACAGCATACAGAAAAAACAGTAAAGATATCACAATTGGCAGT
TCGGCACCTTCAAGTTCTTGCAGGATCACTCTTTCACATAACAGTACAATATTGGGTATCAGTACCATGTACTAG
TGTGTTGATGTGAACTATGTCCATATAATTGCCTACCATGTGCAAGCTATTGGCCTTAGTAACTTGCTAATGGTA
TTGAGATGAAGTACAGCAAGAAACTACAAGGCTAAGCTACCTGATGGAAACAAAACAAATAGAACAAAGGCT
TACCTGAACACCAGCAGGTGGGTCATGGTAACCAGTAAGAAGAGCAAACACGTAGTTCTGGCCATTGTGCCTT
GCCTGAGCATATATGAATCATCAGTAAGATACAAGCTGGAGATAGATTACAGCACTGCAATGCAAAGAAATTA
GCTATAATTACCTTTGTGATAAGACTGAGGTCTGGAGGATATGCCCCACCATTTGCAAATCGGGCTGCCTGTTC
ATTTGGATAAGGCTGCGGGAAGCGATCACTCAGCTTGCCAGGACGTGAGAACATTTCACCCTCATCATTAGGA
CCATCCATCACTTCAATTTCAGCAGCCATTGCCTTGGTTTCCTCTTCAGTATAAGCCACCCCAACCAAATCACGG
TATGAAATCAATGACATTGAGTGACAGGATGCACACACTTGCTGATAAACTTGGTGTCCACGCCGGATTCTGCT
CGTAATGTTTTTTTCAGTGTAAGGTCACAGTTCAAAATGAACACCCCACCCTTTATTCAGTATCTTACTGGGAAA
CTACATTAGTGCAGCA

>chr7D:75708772-75709772

ACGTAAGAATGTTTCCAATATTTCTCACACATAATTATTAGTACATGAAAACATCCGAGGAAATAATGTTTCCGT
CTTACAGTTGCTACATTCTCTACACAGACAACAATATAGTGAGAGTCTAGATTTTAACCTATGCTAGAATCAAAC
TACAGACCCTACTGTTTCCCTTAATATGGTGCTTATTCAGCAGTAGCAACAAAAAAGCCCACTTATTCACAAGG
GAAAGCGGTCCAGCACCTCCTCAAAACATAGCCGTAAGTGAAACCAGGGCTTCAGCATTTCCATTCTGTTCAAA
AATTTCAGAACTGAGACACACAGACAAAAAGTGTCGCATATCCCCAGTCAGTCGCAACACCTTCGCTCCTGGTT
TAGACCTCTGTGTGGATTCTTCTTTCTTTTGCGATACAAATAAGGTAGCCTTGGATTCCAGCCACGCCCGATTTA
CAATTTGTGCAGACCACTTCCTTCTTTTTGGAATAAAAATAAGCTACTCTGCGCTGGGCTCCTTTATTCTGCGCC
ATCTGGACACAAGAGAAACCAGACTAATTAAGCATCTGCATTCAGAAGGAACCCAATGCCCATCCAAATGTTT
GAAGTTCCATGTTTTCATGGATTTTTCGCGACCAATGAAGATCACGGGGTTGCAGGTGACCTTATTTGTTCAGT
GTAGTAAGATGTCAGTCTTGAAGGAGGTACTCAAGCCAGTGTGACCTTCTTGCCTTTCGATTCTCTAGCCTGCA
TAACATTCTATCAGTCACCGTCCAAGAAATACCACGCTAAAAAGGAAACTATTGCAGGTGAAGGTGAATCGGA
TGGGGACATTAGAATATTCAATTGGGATATATGGAGCTTGTATGCTGTGAAATTGAGTACGAATTAGCACCTTC

GTGGAAATCGACTTCCTGGACGACACGAATTCGCCAAACTCCTTATCATACCCCCTCTTATTATCGGCTGGTGTA
GAAGGTCTAGCCTTCCTAGCAACAGACATGAGAAG

>ChrUn:15633586-15635586

CAAACAAACAAACAGCAATATTACAAGCCTAAATGCACTATGGCGAGAATGTGAAATGTTGGTGCGCGAGTAA
AACTAGTACAGAGGATCTAAACACCTGTAACATGCTACTTTATCTCTATCATTGAAATAAATGCTTAAACAGCAC
AAACCACAAAGTAATCAAATTGCTAAACTATGGTTCAAAAAAAAAACTTGGCCTAATAATGAACAAACTGAAA
CAGGTAGAGTTGTGCTAAATGAATCTATTCAATCTTGTGAGCAAAGGTATTTTCACAGTAGCAATTGATTTAGT
CTCAAAGTTCTCTAAAACTAATCTGCAAGTACCCTGAAATTATCGGTTTCTGTCCATTCAGACGCATTTATTGAG
AGCTGAATGTTCCAATTTAGGCTATCTTAAGGTTTAACTGCAGAAGTACTTAGTCCATGCATAAGTGAGCATTA
AAATGTGGACCATATGTGAGCAAAGCTAGTCAAAGGGTCTAAAACACGCATACAGTTGTACAAGACAACTTCC
CTCTCTGTACAGCACAAAGTACTCACGTTCCTAAACTACAACTCAACAACAAAAGAAAGAGTGCAACACTTGGC
ACAATAATCAATGAGCTGAAGCAGGTTTTTGTGCAAAATACAAAAAAAGAAAAACTGTTCAATCTTGTGAGCA
AAGGTAATTGCATGGTAGCAACTGATGAATTCATAGTCTTTAACTTCTCTCCGTGTAATCTGCACAGCATAAAG
ACGCATGTGCAACAAACAAGAACAAGAGAAAGCAGGTGAACACATACGTTGCCCTTGTCGAAGTCTCCTCCCT
GAATCATGAAGTCCTTGATGACACGGTGGAAGCTCGATCCCTTGTACCCGAACCCTTTCTCCCCTGCCAAAAAA
GGGAAGCTTTGTCATGCACCCAGTGAGGGCAATTCTTTAAAGATGGCTCCAGCAAGTCCCTCGTACCAGTGCA
GAGAGCGCGGAAGTTCTCGACAGTCTGCGGAACATCATCACCATACAGCCCGATGACGATCCTCCCGACGTTC
TTCCCAACAGGGTTCCCGATGCTTATGTCAAAGTACACCTTGTTTGTAACCTTGGACTGGAGGTCGGCTGCCTG
CAGTGTATGAAAGGACTGCATCAATACCATTAAAAGGTTCCATAAAAGCAGCACACACCAATGCAAAATCCAT
ATTTGCACATCAGAAAGACATAGTAACATAGAACTGCATATATGTTTCCTTAGACCGCCAGCGACAAATATCAA
CTGAACCTAATGAACTAAAATAACTAACATTACCACATGCCATACTCACCCCTGAATCTGATACACCCTAAACTT
GACAAAACTGTCATCAGAAGAAATCCTGTGATGACAAACCTCAATTTGTGATGTTATTCACAGAAAAGTTTCAG
TGAGTGGCTTATTTTTTTTCTCAAGAATATTTCCGCGAAACTTTTTCATGAATACTACAACTGGTACAGCTAGGA
TATCCGTTTACTTAGACTGCCAGTGACAACACCAACTGAACTCGTAACAAACTAGCATTATGATTCCCTGCATCT
TAACACTCAGAGAAGCTAGCCTAACAGTTATTGGAAAGAAGTATGTGATGACAAGTCTAATTTTGCAATTATAT
TCACAAAAGAAAATCCAGAGAGTGACTTAAATTTCTCCTGAATAATTCTAGAAAAAAAATCATGAGTATTACAA
CTGGTACAGGTACGATGCAGAAATGGCGTGGAATTTTCACATCCGACAATCCAGATTCATCATTATAATTATTC
GACTAGAGTAGACAAAAAGCCAGCTTTACATAGGGTTCGGCGCTGGCGGAGGTGACGAAGCGCGCGCGGCT
CTGGGGCCCTCCGGAGACGGAGACGGAGACTGCCGGGGACGCGACGCGGAGGGAGAGGAGCGGCGAGGG
GGACCTGGAGACCAGGAGGGCAGCCCTCTGCGCCGCCGCCGCCGGGCGCGCCATGGCCTGCAAGAAGATGG
TCAGGAGGAGGGTTAC

>ChrUn:15636733-15637914

TGCGTTCCGAGAGAAAAGCTTTATTTGTCCTTTTATTCCCAAGTTTCAACGAGTACTAGTCGAGGTCATGCAAG
ATGATGCATGACACGATATTTATTTTCCACCGCTACATCGATCGATCCTTCGACTTAACAACGATGATCCATCTA
TCAAGATGGAGCCTTATGTTTTTAGTAGCCACCCATGCTGCAACCAGGCATGGTGGTGGCAGCGGCGGTGCAG
TATGATGGCATGTATAAGTGGCACATCGTCGGCAGGGTCCTAAGGGCTATGCTCCTCATCGCTTCGAATTGGC
CTACTTGTTGGGTTTGGCCCGGTTGTTGCATTTCTGGCTGGAAAACACCACGTCTTATCTGTTGTTGGACGATG
ATGGTGTGCGCGAGGTTGCAGATGGCGGGGCACCGAACTGGCGCCGGGATCTGCGCCAACTGCTGGCAGCA
CTGCTGTCGCATCACCTGACAGGTGCTCTGGTCCAACATCTGTGACCAGACGAACGGTGGCATTGCCAGCGGG
ATGCACTGCTGGAGGAGGAATACCTTGCATGGGTTTAACGCATGTTGCGGGAATTGTTGTTGGCCGAAGCCTG
TCTGCAACTGTGCAACAACGCTAGTCACCGCGACGGTGAGGAGAGTGAGGATGAGCAGGATCTTCATGGTGG
ACTAGTGTGTGGCTAGCTACTCCTTGCTCGGCCTTTATGTTTTTGTGTAAGATGATGGAGGAGGATGCCACAAT
GAACACATACTTATCGCTGTCATGGCACCATCTCTTCTGGTCTTTCACATTTCCTTTTCTCCAAAAGGGTTTGGAT
GCTTCTAAAACCAATCATTTTGGTACCTGTGTGTTGGGTGTGGTCACTACATACTAGTATAAACCTTGGGACAC
TCACCAAATGTGCTAGGTACGGCTCCTCACCCAAACACAATCTTGTGTTATTAGCAAATGTTAGTACCTTTTCTA
TTTCTTGTTGTATGTATATATATAATGGAAACTTGCATGCGATGATAATCGATAATCGAAATGACTCATCCCTGT

TCACTTTACATGTCGACGCTTATCAAGATCACATCTTTTCCCAAAACCGATTTTGTACTTTTGTGTATGATGAGTT
GTTAGGCACAAAACAAACTCATACAAACAACTTGTAAAAGTAATGCACCTTTTTTCATGGCTTTATGTGGCAC

>ChrUn:15641416-15643514

GAACGGAACAATGGCAAGCAATGTTATCCAGACACAAACTTTGTCATTTTTTATTTCCAAGATTGGACTAGTAC
AGGTCTGGGGTCACACATGTTAGTGCATAACCCTTTATTTTATTTGTTACCGCTACAACCGCCGGTCCATCGGCT
AAACACCGATGATCTGTTTATGTAGTACTATAGCCCTTGTAAGTCTTCTCAGACCACACCGAGCGGGGTGGTGG
CTACGGAGCAGTGCGGTGGAACATACACATCACACATCGCCGGCAGGGCCTGCAGTGCAAACGCCCTCATCCC
CTCGAACTGAGCTTGCTGCTGAGGCTGGAAAACCTGTTGCAGCTGAGGCTGGAAAACCTGTTGCAGCTGAGG
CGGGATGAAAACCTGTTGCAGCTGAGGCTGGATCAACTGTTGTTGCAGCTGTTGTTGCACCTGAGGCTGGAGC
AGCTGTTGTTGCACCTGAGCCTGGAGCAGCTGTTGTTGGAGCTGAGCCTGGAGAATCTGTTGTTGCAGCTGAG
GCTGGAGCAGCTGTTGTTGCAGCTGAGGCTGGAGCAACTGTTGTTGCAGCTTCTGCAAGATGATGGCTTGCAC
GACACTATGGATGGCCGGGCACCGAACCTGCTCGGGGATCTGTGCCAGCTGCCGGCAGCACTGTTGCTTCATC
ACCTGGCAGCTGCTCTGTTGCAGGATCTGCGACCGGAGGAACGGCACCTCTGCCACTGGGCTGCACTGTTGCA
CGAGGAATTGCCTGCATGGGTTCAGCTGTTGCTGCAGAACCTGCAACAATTGTTGTTGCTGCAACAATGGCTG
TTGTTGTAGAAATGGTTGTTGTTGCTCAGGATATGGCTGATATTGTTCGCTAGGGTAAAACTGGGCAGTGGCC
ATGGTCGCCGCCATGGCAAGGAGGGCAAGGATGAGGAAGTTCTTCATGGTGGATTTGTGTCCACTACTGTTTG
CTTGGCTCTGATGTGTGTGCTGTAGGTGTGTGAAGGATGGATGATGGAGGAGGATCTTCACAGTGCATGTCTA
TTTATGCTGCTATGGCGTGGTTTCTTTTCATCTTTGCATTTGCTTTCCTCAAGAGTGTTTGGGTGCTTACCGAACT
GATCATTTGGTGTCTTGTGTTTGGTGGCACTACTTACAAAATATAATTTTTGGATTGATCATTAAAGTTGCTTTT
CCCTTGCACGTATCATCTTCTGACTCTCAAGAAAGCTTGATAGTTGGTAATACGTCTCATCTTATTCACTTTACAT
GTCAAACACTGATCTATATTGCATCATTTGTAAAACTAATTTTGTATTTTTCTACAGACTGTCAGGCATAAACCA
GATTGTCATGGCTTGCAAAGTTTGTAAACTTGTGTGTGCATAAGATAGATTAAACTAGTAACATGAAAGATTAT
CTAGACTCCATTTCTTTGGCTATGTAGTGTTGTATACGATGTTAAGTTGAACTAGTAAACATGACTCATCAAATT
CGCCTTTTATGTCAAGAATCGTAATGGCGTGCAAGTTATTAAACATATCAGATTAGTTAAGTTTTATATGACTTC
GATAGGGTTCACTAGCAGATTTAATCAAAATGTTCAAGACAATTATTGGTACTGAGTAGGATTATTGCAAATGA
TAGCATTTATACAACATATAATGGCATATTTGTTTCCTCAAACTGCTAAAGGATCTATTTAGCAAACCATTCATT
GCATAACAAACAAATGGAGTTTCTTTTATTGATGCTTTTATCTATCTACCTTGTTGCAAGACCCATCAAATTTTCT
TCTGCAAAACAAACCAAGAACGTGCGACAGAACTAATCAGCCAGTCACATTTTGTGGTAGGTAGTTTTTCATCT
GAGAACAGACATTTGATGCCGAGTTCTCGTGTCTATCTATCTCGTCGTTTTTTTAAACATAAACACCAAATATAT
TTGGCTTTCACAAGAATCTGTAATGCAAATTTCTACGTGAAAATATTTTCATTCATAGCTGACAAATATAGCAAA
TATTAATGCTCCATGTTTTATATTTTCTCACAAAAACTAGTTTCTGTACATGACATTTGTAGTTCAACTTAATATTT
TAAACACACAATGGCAAAAAA

>ChrUn:15644442-15646314

AACATGAATGGAAAACGACAATCAGTTTTATGCAGACATGAACTTTCTCTTCTTTTATTTCCAAGTTTGGACTAG
TACAAGTCGGGGTCACACATGTTAGTGCATGACCCTTTATTTTATTTGTCACCGCTACAAGATCGGTCCATCGG
CTAAACGCCAATGGTATTTATGTAGTACTATAGCTCTTATAGTGTTCTTAGAAGCCACCGAGGGGGCGGTGGC
GACAGGGCACTGTGGTGGGACGTATACATCGCACATCGCCGGCAGGGCCTGCAGCGCAAACGCCCTCATCCC
CTCGATCTGACCTTGCATCTGAGGCTGGAAGATGCCCTGGGTCACCTGTTGTAGCTGAGGCTGTAAGATGCCC
TGGGTCACCTGTTGCAGCTGAGGCTGGAAGATGCCCTGTGTCACCTGTTGCAGCTGAGGCTGGAAGAACTGTT
GTTGCATCTGAGGCTGGAAGAACTGTTGTTGTTGTTGTTGTTGCTGCATAATGATGGCCTGGACGACGCTATG
GATGGCCGGACACCGGAGCTGCTCGGGGATCTGCTCCAGCTGCCGGCAGCACTGTTGCCTCATCACCTGGCAG
CTGCTCTGTTGCAGGATCTGCGACCGGAGGAACGGCACCACTGCCACCGGGCTGCACTGTTGCACGAGGAACT
GCCTGCATGGGTTCAACTGTTGCTGCAGAACCTGCAACAATGGTTGTTGCTGCAATAGCATCTGTTGTTGTTGT
TGCTGCAACAGCATCTGTTGTTGTAGAATTGGCTGTTGTTGCTCAGGATATGGCTGATATTGTTCGCTAGGATC
AAACTGGGCAGTGGCCATGGTCGCCGCCATGGCAATGAGGGCAAAGATGAGAAAGGTCTTCATGGTGGATTT
GTGTTGACTACTGCTTTCTCAGTTTTGATGTTTGTGCTCTCGGTGTGTGAAGGATGAATGATGGAGGAGCATCT

TCATGGTGCAGGTCTATTTATATCTGCCATGGCATGATTTCTTTTTATTTTTGCATTTGCTTTTCCTTAAAAAGTG
CTTGTATGCTTCTCGAACTGATTATTTGGTATGTTGTGTTTGGTGCCCCTACCTACAAATGTAATTTTTGGATTG
ATCATAAAAGTTTCTTTGCCCTTGCACGTATCATCTTTCTACTCTCAAGAAGGCTTGGTAGTTGGCAATGCATAT
CATCTTATTCACTTTACATGTCAAACACCGATCTATATTGCATCGTTTTCTAAAACTAAGTTTGTATTTTTCTACA
GGCTGTTTGGCATAAACCATATTGTCGTGCCTCACAAAGTTTGTAAACTTGTGTGTGTATAAGGTAGATTAAAC
TAGTAACAGGAAGGATTGTTCTAGACTCCATTTCTTTTTTGAACAAACATTCTGGTTTTGTATAATACGATGGTA
TGATGAACTAGTAAGCATGACTCATCAGATTCGCCTTTCGATTACACTATACCAATTATCAATTATAATCTTGAT
TGTTAAAATAAAACATGCTTTTGAGTCCCTTGTATCTGACTAGTGATTTGCAGTCACTACTCAGCATGGGATGA
ACTTAATAGGGTTATTATGCTTAACGACTCTGATGTGATTCACAGTTCTGTTGATCAGTACTGAGCGGCTGAAC
CTGATGAGCTTATATATGATTTGTTCTTACTATGCTAGATATCATTAGCTGCTGTATTATTTTGTATTGTTGTTCC
CTGAGCTTAAATAGCTTGTTTTGTTTTACAAGGAGGCCTATCCAACCGATGTCCTTCCATTTGATATCTTAGCTG
AGAGAGCTCCACAGCAGAAGTTCGAATTGGTTAGTAATCGTAACATTGCGTTCACTAGAAGTTTGAATTGATTC
TTAGTTTTGAATTGCTATAT

>ChrUn:15647763-15649914

AAAACGACAATCAATGTTATGTAGACAAGAACTTTGTCTTTTTTATTTCCAAGTTTGGACTAGTACAAGTCGGG
CATCACACATGTTAGTGCATGACCCTTTATTTTATTTGTCACCGCTACAAGATCGGTCCAGCGGCTAAACACCGA
TGGTATGTTTATGTAGTACTATAGCCCTTGTAGTGTTCTTAGAAGCCACCGAGAGGGGCGGTGGCGACAGGGC
ACTGCGGTGGGACGTATACATCGCACATCGCCGGCAGGGCCTGCAGCGCAAATGCCCTCATCCCCTCGATCTG
ACCTTGCATCTGAGGCTGGTTGAAAACCTGTTGTAGCTGAGGCTGGAAGACCTGTTGTTGCAACTGAGGCTGG
AAGACCTGTTGTTGCAGTTGCAGCTGAGGCTGGAAGACCTGTTGTTGCAGCTGAGGCTGGATGAACTGTTGTT
GTTGTTGCTGCTGCAAGATGATGGATTGCACTACGCTGTGGATGGCCGGGCACCGAAGCTGCTCAGGGATCTG
CGCAAGCTGCCGGCAGCATTGCTGCCTCGTCACCTGACAGATGGCCTGTCGCAGGATCTGCGACCGGAGGAA
CGGCACCGCTGCCACTGGGCTGCACTGTTGCACAAGGAATTGCTTGCACGGGTTCAACTGTTGCTGCAGAAGT
GGCTGGAGAAACATTTGTTGTTGCTGCACAAATGGTTGTTGTTGTACAAATGGCTGTTGTTGTTGTACAAATGG
TTGTTGTTGTTGTACAAATGGTTCTTGTTGCTCAGGATATGGCTGATATTGTTCACTAGGGTCATACTGGACAG
TGGTCGTTGCCGTGGCTGTGGCCACCGCCATGGCAAGGAGGGCAATAATGAGGAAGGTCTTCATGGTGGGTT
TGTGGTGACTTTTACTTGCTCGCCTTTGATGTTTGTGCTCTCGGTGTGTGAAGGATGAATGATGGATGAGGATC
TTCATGGTGCAGGTCTATTTATATCTGCCATGGCATGATTTCTTTTCATTTTTGCATTTTCTTTTCCTTAAAAAGTG
CTTGTATGCTTCTCGAACTGATTATTTGGTACGTTGTGTTTGGTGCCAGCTACAAATGTAATTTTTGGATTGATC
ATAAAAGTTGCTTTGCCCTTACACTTTTCATCTTTCAACTATCAAGAAAGCTTGATAGTTGGCAATATGTCTAAT
CTTTTTCGCTTTACATGTCAATCACTGATCTATATTGCATCATTTTCTGAAACTAAGTTTGTGTTTTTCTACCGGCT
GTTAGGCATAAACCATATTGTCATGGCTTGTAAAGTTTGTTAACTTGTGTGTGTATAAGGTAGATTAAAGTAGT
AACAGGAAGGATTGTTCTAGACTCCATTTCTTTTTCTGAACAAACATTGTGGTTTTGTATAATACGATGGTAAG
ATGAACTAGCAAGCATGACTCGATCAGATTCGCCTTTCGGTTACACTACCAATTATCAATTCTAATGCTAATTGT
TAAATGCTAATAAAATATGATGTTGCGTCCCTTGTATTTGACTGGTGTTTTGCAGTCACTACTCAACATCGGATG
AACTTAATAGGCTTATGCTTAATGACTCTGCTGTGATTCACAGTACTGAGCGGATGAACCTGATGAGCTTATAT
ATGATGTGTTCTTACTATGCTAGATATCATGAGCTGCTGTATTATTTTGAATGTTCTGTTCCCTGAACTTAAATA
GCTTATTTTTTTACAAGGACTGGAGGCCTATTCAACGCCTGTCCTTCCATTTGATATCTTAGCTGAGAGACCTCC
ATAGCAAAAGTTCGAATTGGCCAGTAATCGTAACATCGCTTTCAGTTGAAGACTAAAATCTCTCATAATTCTAAT
GTTTGAATTGCTACATCTTTGGTGCTAATAATTCACTTCTGGGTGTCCACCTTCGTCACTTGGACCTTTTCAGGA
TAAGGTAATTTCGTTATACTCTGTTCTTGGACTGACTTATTGATTGCATCAACTTAGGAAGGCAGAAACATCATT
TATCTCACAGTAAGAAACCTGATCGCGAGCCCATAGAAATCTGGCGCTACAAAAGGAAGCAAGAAGAGACGC
TGAAACTGTTCATTCGCCACTTTTTTACTAGCTGGTTTTCATCCCAAGAAGTCAACAGATGCTCTCATTCGAGCT

>ChrUn:15651196-15653714

GTAAAACAACAATCAATGTTATGCAGACAAGAACTTTGTCTTTTTTATTTCCAAGTTTGGACTAGTACAAGTCG
GGCGTCACACTTGTTAGTGCATGACCCTTTATTTTATTTGTCACCGCTACAACGATCGGTCCATCGGCTGAACTC

CGATGGTCCATTTATGTACTATAGCCCTTATAGTCTTATCAGAAGCCACTGAGCGGGACAGTAGCAACGGGGC
AGTGCGGGGGGACATACACATCGCACATCGCTGGCAGGGCCTGCAGCGCAAACGCCCTCATCCCCTCGAACT
GAGCTTGCTGCTGAGGCTGGTTGAAAACCTGTTGTAGCTGAGGCTGGAAGACTTGTTGTTGCAGCTGAGGCTG
GAAGACCTGTTGTTGCACCTGGGCCTGTTGTTGCAGCTGGAGCTGGAAGACCTGTTGTTGCAGCTGAGGCTGG
ATGAACTGTTGTTGTTGCTGCTGCTGCAAGATGATGGCTTGCACTACGCTGTGGATGGCCGGGCACCGAAGCT
GCTCAGGGATCTGCGCAAGCTGCCGGCAGCATTGCTGCCTCGTCACCTGGCAGATGGCCTGTCGCAGGATCTG
CGACCGGAGGAACGGAACCGCTGCCACTGGGCTGCACTGTTGCACAAGGAACTGCTTGCACGGGTTCAACTG
TTGCTGCAGAAGTGGCTGGAGAAACATTTGTTGTTGCTGCACAAATGGTTGTTGTTGTTGTACAAATGGCTGTT
GTTGTTGTACAAATGGTTGTTGTTGTACAAATGGCTGTTGTTGTACAAATGGTTGTTGTTGTTGTTGTTGTA
CAAATGGTTGTTCTTGTTGTACAAATGGCGGCTGTTGTTGTACAAATGGTTCTTGTTGCTCGGGATATGGCTGA
TATTGTTCGCTAGGGTCATACTGGACAGTGGTCGTGGCCGTGGTTGTGGCCACCGCCATGGCAAGGATGGCAA
TGATGAGAAAGGTCTTCATGGTGGGTTTGTGGTGACTACTGCTTGCTCGCCTTTGATGTTTGTGCTCTAGGTGT
GTGGATGATGGATGAGAGGATCTTCATGGTGCAGTTCTATTTATATCTGCCATGGCATAGTTTCTTTTCATTTTT
TGCATTTGCTTTTCTCAAAAAGTGCTTGTATGCTTCTCAAACTGATTATTTGGTATGTTGTGTTTGGTGGCACTA
CCTACAAATGTAATTTTTGGGTTCATCATAAAAGTTTGCTTTCCCTTGCACGTATCATCTTTCAACTCTCAAGAAT
TGCTTGATAGTTGGCAATATGATCATCTTATTCACTTTACATGTCAAACACTGATCTATATTGCATCTTTTTCTCA
AACTAAGTTTGTGTTTTTTCTACTGGCTGGCTGTTAGGCATAAACCAGATTGTCATGACTCGCAAAATTTGTAAA
CTTGCGTGTGTATAAGGTAGATTGAACTAGTAACATAAAGGATTGTTCTAGACTCCATTTCTTTTTCTGAACAAA
CATTGTGGTTTTGTACAATAGGATGGTAAGATGAACTAGCAAACATGACTAATCAGATTTATCTTTTTTGTCAA
GGATCGTAATGACGTACAAGTTATCAATACATATCATATTTGCAAGACTTTACACGACTTTGTCTGGTTCACTAC
ATGTGAATGTTAGTTTTGCATTTCATTGTTTTGTTGAACTAAATAAGTTTGTAATATATGCAATTTTTTTAGACAA
TCTCAATGTTTTCTTAGGTGATTTGTTCAAAAAAATTCAGCAGATTATTAAGCATTTATACAATAAATGATGGCA
TATCTTGTTTCCTCACACTACAAATGCATCTATTTAACACACCATCTTCTACAATGAAACCATTCATCGTGTAACA
AAAGTAGTTTCTTTGATTGACACATTTATCTATCGACCTACACTACTAGGAAAACGGCCTTTAGTGGCGCACCT
GCAGTGCGCCACGGGCCTTGCGCTACGGCTATCTCTCAGCCGTGGCGCACTGCCTAGTGCGCCACGGTTGTGT
AGAGATAGGCGTAGCGCACCTCCTAGTGCGCCACAGAAGTTTGACATGGTGCGCTATCCCTTGGATCTTCTGT
GGCGCACTACCTAGTGCGCCACTAAACTTCCATGGTGCGCTTTAGGTGTAGTGTGCCACAGAAAGTAGACCAT
GTGCGCCACAGATGTATTTTTTTTAATTTCCAAGCCAGATTATATGGAATACAGATTATATACAATATTACAGCA
GATATACACACACAGATACAAGATATACAATTATATATATAAATACTCAATATGAAAAACAAGATTGAAACAAA
TACATATAGATAAGTCTCATACATGAATACATAGTGTAAGTTACATTGTTTTGATCATGTTACAAAGTAGTAGTT
GGTGACATGCGAATACATGTGTCGATCGAACAAGTTAGAGCTCGGAAGCTCGCCTAGACATCTAAACTAGCTA
GCTAGACACAAACACGGGATAAATAACCAAAGAAGATAAGCATGATCACCGCCGCGACGAAGGTGTAGGTCT

>ChrUn:15655427-15656827

TTTTTTGTTTTTATTTTAAAAATACTTTTAAATGTTCCAAAAATTCCCAAAAGTGGTAATATATGTCATATTTGTTT
TGTTTTTATTTTATAAATACTTTTAAATGTTCCAAAAATTCCCAAAAGTGATAATATATGTCATAAAAGTGGTCG
ATAGAACCACATAATGAATGCATGCATGCAATGGTCGATAGAAAATTTTGGCATGACCTATCCTAAAATTAGG
ACCTTTGCGAAATGCCAAAGTCTTGCCAAAATGGAAATGAATCAACATTTTGGCAAAACAAATGGCAACTTGTT
CACATACAAACACAAGAGTGAGAGAGAGACCGATCCATACACACATGAACATATCATTATCCTCTCCATACACA
CATGCATGAGCATCATCGATACTCTCCATATACACACACATATGACCATCGATCCTCTCCACACATACACACATC
ATGAGCATCGATCATCTACATACATATGAGCACATCATCCATATAAACATGAACACATCCTTACATGGACAACTT
ACCGAGATGAGAGACCGATCGCGAGAAAGATGGAAAGGAGAGGTGCGCGCGTCGACCGGAGAAGGATAGA
TGAGAGACCGGAGACCTCCTCTATTACTTATGTATCATCCAAAGCAAGTTTTACATGCTAAAAATGGTGAATGT
GTGGAGAAATTGAGGTGGGAAGGAGAAGATCCCGACTAGATCTAAGGAGGAAGAAGAAGGGGAAAGTGAA
GTGTGGGGTGGTGGATGTGTGGGCACACATGTGCACATAAAGTGCTGTCCTAAAAGAATCGACACATCCGTG
GCGCACAAGGCCCTAGTGCGCCACAGGGAGTACAAACTTCTGTGGCGCACTCGGCCGAGTGCGCCACAGATG
TTTGTGGCCGTTTCACGGCAGAGGGGGGCGTGCGCTTAGGCGTAGCGCACCCGCCCCATGTGCGCCACAGAT
GACACAAACAACCGTGGCGCACATGACTGTAGTGCGCTACAGATGTTTGGCGTGCGGCCAACAAGGCCCTGG

CCCACACACATCTGTGGCGCACTATAGTCATGTGCGCCATAGTTGTTCTAGTTACTAGTAGGGCACTGCCCACA
GGTGCGCCACGCCTGTTTTAGCCGTGGGGCACTTCAACCATGTGAGCCACGGATGTGCTGATTACCTATAGCC
ATTTTCCTAGTAGTGCTAGTTGCAAGCTATTTTTTTAATATTATTTATTAAATTCATAAATAATACACAGATTTGA
GGAATCGCAAATCTAGCGTGGCCTCGGGGGATGAACTCCTGACCAGCCCGTCGGGGGATAAACCACCGAGCA
GGCTGTCCGAGGCACGCCGGGCATGACAGAGAGGTCGTTGGGTGTTGTTATCCCAACTAGCCTAAACGGGAT
CTTAACCC

>ChrUn:15673395-15674714

GCCTCGCCACCAAAGAGTTAATTCCTTACTGAATGGTACTTCAACTCAACCCAAGTTTGGCACCCATTTTTTTAT
GTTTTGATTAATTATAGTGCACATGATTGTGATTAGGGATGAACATCTCACTGTGCACATGAACGGAACAACGA
CAAGCAATCGTTATGTAGACAACAACTTTCTTTGTCTGTTATTCCCAAGTTTGGCTAGGGTCATACATGTTGGTG
CATGACCACCTTATTTTTCACCGTCGGCTAAACACCGATGGTCCATCTATCTAGTACTACAACTATTATCTTCTTA
GCAACCACCGATGCCGGCGGCGATACCGCTGACCTGGCCAGTGGAACAATACGGTGGGACCTGCACATTGCA
CATCGCAGGCAGGGTCTGCATCGCTACCACCTTCATCGCCTGGTACTGAGGTAGCTGTTGAGGTTGGATGAAG
ACCTGGCCCGCCATTTGTGTCTGAGGCTGAACGAGACCCATTTGTTGAGCTTGGACAACTTGTTGCTGCTGCAT
GACGATGGCGTGCACCATGCTGTGGATGGCCGGGCACCGGAGCTGCTTGGGGACTTGCGCCAGCTGCCGGCA
ACACTGCTTCCGCATCACGTGGCAGGTGCTCTGTCGCAGGATCTGCGACCGGAGGAATGGTACCATCTCCACC
GGGTTGCACTGTTGCATGAGGAACATCTGGCACGGGATCATCTGTTGCTGCAGCAATGGCTGCATAAATGGTT
GTTGCTGCTGCTGCATAAACGGTTGTTGCTGCTGCATAAATGGTTGTTGTTGCTGCTGCATAAACGGTTGTTGC
TGCTGCATAAATGGTTGTTGCTCGGGATAGGGCTGATATTGTTCGCTAGGGTCATACTGGGCAGTGGCCATGG
TTGCCGCCATGGCGAGGACGACGAGGATGAGGAAGGTCCTCATGGTGGATTTGTGTTGACTAGTGCTTGCCT
GGCTTTGATGGTTGAGCTCTAGGTGTGTGAAGGATGGATGATGGAGGAGGATCATCACGGTGCAGGCCTATT
TATAGCTACCATGGAATGGTTTCTTTCCATTTTTTACGTTTGCTTTCGTTTAAAGTGCTTGGCTGCTTGTCGAACT
GATCGGTTGGTGCGTTGTGGTTGGTGGTGACTACTTACAAAATGTAATTTTGGATTGATCTTCAAAGTTGTTAT
GCCCTTTCGTGTCATCCCGATCTCTGGTAAGATTGATAATTGGCAATGACTCATGTAACTCACTTTACATGTCAA
ACACCAATCTAGATTGTGTTATTTTCTTAAACTAGATTTGTAATGTTCTACATGTTGTTGGGGATA

>ChrUn:1818787-1821231

AGAATATAAAATTGAATAAAGAACAAGACACAAACAGAGGATATAACATGATAAAGTTTTTTATTAAGATAAG
CGGTTATATACCACCCACTTCCACACTTGTAATGCCTTTATTTTACAATATTGGTCCTCACACACTTACTAATTAA
CTCGGATGCTTTAGTAGTAAAAAATGACTCATCCTCGTCCGCTGGATCACATTGGAAGCTCGTCTGGGTAAATT
TTGGAGTGAATATACCCCAAACTCTTCTCCCCTGTTGTTTTTGAGGTTCCGGGCTTTCTGCATTGGCGAGGACAT
CCACAGGCAAGGCACGCGGAATAGAGCTCTTTCCTGCGATGTGGCTAACCATGGAGTTCGGATTGGTCTTGAA
TGAGATATACTGGCATCCTTCATGTTCCGCCTTATTGAGAACAACGTAGTTTTGCGGTACGATTAGCAACTGCC
CTTGGCAAAGACGGTCATTGAATACATTCTGACCATTGTTATTGACAACTTGAACTCGAGCATGTCCTTGGATC
ATGTAGTGTTCCAGAATGGTAAAAGAACGGCATTCTGCAATAGCAATTATTTATATGCCATATTATTAATCGGA
TGTTATGAAGCTCAGAGTATCTAACAATAAGATAGTATGGTGAATATTGTATATACCTAGTATAGATTTACTCTT
GTAGCACTCATTTGCACGAGGTTAAGGATAGGGAAATTGTTGCCATGGAGACGTGTTATCCTGTCAGCACGTG
CGTTGTACGTGTCGGCACGTTTGGGGTTTTCGATGTTCTGCCTTGACTCCAATGAACAAAAGTAAATGTTAGAG
TATACGTATCTGTTAGGGATAGACTAGGTTACTTGTCTTGTACTCCAAGTAGGTTACTTGTCTTGTAATCCAAGT
CGGTCCCTTTACGTCTATATATACTCGCCCGAGAGGCTCAATAATACATCCATCATATTCCGCAATCTCTCTCTCC
CTCTACCCTTCAGCATGGTATCAGCCTAAACCGATCCCAAACCCTAGCCGCCGCCGGCATGCTTCCGCCGCTCC
GCCCCCGGGGAGGTCGATATCCATGATCTCCGCCAGGGGCCGCGCAACCCGTATGTAGGGTTCGTCCGCCGAT
CCGTGATCGGCTGCCCTGCAAGTCTTTTTTCCGATCTCTTGATCGGTTTTCTTTTTTTGCTTTACCGATCTTAGAT
CGGATTGCGTCGCCCATCGCTGTCGTCATCACCATGCCTCTGTAGGGGTTCGTAGCATGGAAAACAAAAATTTT
CTATCGCATACAAACAAACAAGCCAAGATCTATCTACTAGATTGTAGCAACGAGTCGATGGAATAACTTACCCT
CGAAGATTCCAAAGCGGTTAATGAGAGCAGATTCTCGTGGGTGATGTAGTCGTACTCTTTCCGATCTCAAGATC
GTGTAGACGATCCTTCCGGCGCTGCGTTCCGGCAGCGCTCCCGCAGTCGGTCACACGTTCGGTACTCGACGAA

GACCTTCCTCTCCCTTGTTCCAGCAGGCAGCGGAGGTTGTAGCACTCTCCTCGCGATTCCGGCAGCACAACGGC
GTGGTACCGGAAGTAGAGGGGAAATCCCGCAGGGCTTCGCCTTAAGCCGGTGCGAGTATGGTGGAGCTTATG
AGAGAGGCTATTAGTGGTCTAGTAATTAGACACACCAATTAGAAGGCTCTAAACCAATTAGAAGGCTCTAAAC
CGCTGGCTAAAGCAATTAGAAGCTCTAAACCAAAACGGCTCTAAATCGGAGGTAGAAGATAGGAACTTGTGT
GTACTAGGAGGGGAGCCCTGCCCCTCCTTTTATAGATGGGGAGGCGCGGCCAGGGGAGGGGAGTCCCTCCCC
AAGTCGGCCTTGGGCTGGGCGCCGGCTTGGAGGAGTGGGACTCCTCCTCCAAGTTGGTTCCAACTCCTCTTGC
TTGTTTCCCACAGTTTCCTGTTTCCCACAGTTTCCTGTTTTAGCCGTTTGACTTTCAAACTGGCAATTTGACTTTCA
TGTTTTTGGAACAGCAAGAACACTTAGTACATGATGCAATCATTCAATGACACACTCATGTACCCTCCCCCCCG
GAGTGCCGGACAATCCGGAACCTTCTAGAACATTCCCGATGAAAACACCGGAATCATTCCCGAACTCTGAAAC
GGGTCTTTCCTTATATGAATCTTATTCTCCGGACCATTCCGGATCACCTCGTGATATCCCGGATCCCATCCGAGA
CTCCGAACAACATTCGGTCTCTTTCTCATAATACCATAACTACTCAAACAACGTCGAATCCTTAAGTGTGTCTCC
CTACGGTTCGTGAATTATGCGGACATGATCGAGACACTTCTCCGAACAATAACCAATAGAGGAATCTGGAAAT
TCATATGGCTCCCACATATTTCTACGATTACTTTGCGATCGAATGAACCACTTACATACGATACCAATTTCCCTTT

>ChrUn:1881792-1883792

AAAAAAAAAAAGAAAAAAAGACTTTGCTGTGTGTACTGCGAAATACACACGGCAAAGGCCCTTTGCCGTGTGT
ACTACGGAATACACACGGCAAAGGGCCTTTGCCGTGTGTACTACCGAATACACACGGCAAAGGACCAGCGAG
GGCACACGGCAAAGAGACCACCGTCTACGGCCGTTAGCTCCTCCCGTTAAATGGACTTGTGGGGTCACGGGTC
CCACCTCGTTGCCGTGATAACACCTATGCCGTGTGCCGTTGTCAACTTTGCCGAATGTATTTTCTTTGCCGTGTT
CCTTTATGGGTCTTTGCCGTGTATATTTCTTTGCCGTGTTCTTTTTCAGAAATTTGCCGTGTTCACGTACTTTGCC
GTGTATTTTCTGAGTTACACACGGTAAAGACTGTCTTTGCCGTGTTCCGTTGCGTTTGACACACGGCAAAGACG
TGTCTACACGGCAGCGACATTTTTTCCTGTAGTGATAGCAACTTAGCAAGTGGTTAATTATGAATCAACAGTCT
ACATTCTATAGTAGTAGCAGTAGATGCAGATCCAAGGTTTCCATGTGATGCCACCACCTCTGCACATGAAAACC
TCGTATGGATGCACGTTACTTGTGTTATTTTAACCATGCACAAGATGCCCGTCACATCAACAAGACTGGCCCGA
ATATATAAGATGGTATAGGCAGGCAGCATCTACGCTTCCTCCTACACCAAAAATCCCTTCTTGTGAGAACCAAG
AGAGAGTCCAGCCAAAAATGGAGGGCGCGGCGCATGCCATTGTGAGCAATGTTGGGCAGCTGGTGGGGGAG
GAGTTCCGGCAGCTCCGTGGCGTGGGCGGCGAGGTCGCCCGCCTGAGGGACGAGTTGGCCACCATGAATGCC
CTCCTCCGCATGCAGTCCGAAGCCGAGGAGGGTGCCGTGGACCACTTCGTCCGGGAGTGGATGAAGCAGCTG
CGGGAGGTCGGCTACGACGCCCAGGACTGCGTCGACCTCTATTTGTTCCGCGTGAGGACCCGGTCCGGCGAC
AGCCTCATTGGCTGGTGCAAGCGACACCTCAAGACGCTTCTGTGTCGCCATCGCCTCGCCGGTGATATCAGGG
CCCTCCGTGCCCATGCTGCCGCCGTCAACGAGCAACATGCTCGTTACGGCGTCAGCCTCGAGGCACTGCGCCG
CCCTCCTTCTTCATTGTCAGCGGTGCGGGCGTCTGCACATGTGCTCCGCCTGGCCGTTGCCGACGGGCCCAACA
AGCTCGTCGGCATCACGGAACAGGCTATCTTTCTGGCGGATAAGGTGATTGCGCCGAGTGACACTACTGATCT
CAAGGTATTCTCCGTTGTGGGCTTTGGTGGGCTCGGAAAGACCACGCTGGTCATGGAGGTGTGCCGGCACCTG
GAATCCCACTTCCAGCGCCGAGCACAAGTGTCCGTGTCCCAGGTGCTCGACCTCAAGGCACTCCTCAGGAGCG
TGCTTCATCAGATCCTTGAGGTGCATGCAGCGGATTCTGCAAACAATCCCATCCAGCCCCCTGCTGACATCGAC
GGCATGGACACTGAAAATCTCCACCAGAATATCAAGACCATTCTCAAGGAAAAGAGGTACAAATATGAACATC
TTACAGACTTAACGCCATGTTCACTTAGTTTCTACTTTATTCGGATAACTGTTTTGATTGATTTTTTTTATAAAAA
AATCCAAATCAATACACTTCTTATGGAACTAATTGCATTGATAATCTACATTAACTCTCGAAAACAAACTACAGT
TTTCAGCCGGATGTTTACATAACGTGGTACTCTCTCTGTTACAAAATATAAGATGTTTTAGCTTTTTTTCTAAAAC
ACATATATCTAGACATATTTTAGCGTGTAGATTCACAGCAAAATGAGTAACCTATGAACTAAAAGATGTCTAGA
TATATGTGTTTTAGAAAAAGCGAAAACATTTTATATTTTCAAACGGAGGGAGTACGTACTAACTTTGTTCTCAT
GTATCTATGGCAC

>ChrUn:1994446-1996446

GGACGGTTTTTTTTTTGAAATTTGAAAAAAACTTCTGTGGCGCATATTGGCCAAATGCGCCACATAAGTTTGAG
ATCTGGTTCGAGACTTCTGTGGCGCATTTCGGCCATATGCGCCATAGAAGTTTTTCAGACTTCTGTGGCGCATT
TCAATATGCGCCACAAAACTGAACTTTAGTGGCGAGATAACAGTGGCGTATCCCATATGCGCCACAGAAGTCC

CAAAATGGTGCGCCACTGATGGGCCTTTTCCTAGTAGTGACAAGTTGCATGTCAATCATGGAGCAAGTCTCAT
GAACGATGTCATGCAAGGTTAGCCCGGGCCGCTTCATCCCGCCATCCCGCAATATGCAAAGTACACAAACAAC
GCCCACAATGACCATTGTGTTCTACTCGTGCAACAAATCTACATATATACACGACTCTGATACCACTGTAGGGG
TTCGTAGCATGGAAAACAAAAAATTTCTACCGCATGCACACAAACAAGCCAAGATCTATCTACTAGATTGTAGC
AACGAGTCGATTAGAGTACTCACCCTCGAAGATTCCAAAGCGGTTAACGAGAGTAGATTCTCGTGGGTGATGT
AGTCGTACACTTGCCGATCTCAAGATCGCGTAGACGATCCTTCCGGTGTTGCGTTCCGACAGCGCTTCCGCAGT
CGGTCACACATTCTGTACTCGATGAAGACCTTCCTCTCCCTTGTTCCAGCAGGAAGCAGAGGTTGTAGCACTCT
CCTCGTGATTCCGGCAGCACGACGACATGGTGTCGGAAGTAGAGGGAGATCCCGCAGGGCTTCGCCTAAGCC
AAAGCGAGTATGGTGGAGTAGAAGAGGGCGTCGACGATCTAGGTTTTAGATTCCGAGGGAAGAGGGTAGCC
GGCGGCTAGGGTTTAGGCTAGATGGCAGCGATGGTTTAGAGGGGATCTAGGGCGCCGGTGGCTTGGGGGCA
GCGGCGGCCTAGGGATTGGGACTTGGGCGCCTAGGGAAAGGGGGATACCTTGGGGGTGCGCCCCACCCCTC
CTTATATAGATGGGGGCGTCCAAGGGTGTTCCCAACCCAGGTGGAAACCCTCTCTAACTCAGAAACCCTCCCTA
AATCGGAAACCATGTTGGGATTTGACTTTTTCAAACTGCCTTTTTGACTTTCAAATTAGAGTTTGCTTTCTCTCTT
CCAAGTGTCTAGCCAAGTGACCTAGGACATGATGCAAACATTCCACTTTGTCATGACACATCCATGTACCCTTC
CCCTTCCGGATCGGTGAACCTTCTAAACCCTTCTAGAACATTCCGGTGAAAACACCGAAATCATTTCCGAACTCC
GAAATTGGACTTTCCTTATATGAATCCTATTCTCTGGACCATTCCGGATCTCCTCGTGATATCCCTGATCCCATCC
GAGACTCCGAACAACATTCGGTCTCTTTACTCACATTCCATATCTACTAAACAACATCGAATCTTTAAGTGTGTC
TCCCTACGGTTCGTGAATTATGCAGACATGACCGAGACTCTTCTCTGATCAATAAATAATAGAGGAATATGGAA
ATTCATATGGCTCCCACATATTTCTATGATTACTTTGCGATCGAATGAACCATTTACATATGAAACCAATTTCCCT
TGTCTCGCGATATTTTACTTATCTGAGGTCTGATCGTCAGTATCTCTGCATACCTAGATCAACCTCCTTACTGATA
AGTATTCTTTTCTTATTCCCGTGATATGTGATCCCTTATGAGCCAGTCACATGCTTGCAAGCTAATTGGATGACA
TTCCACTGAGAGGGCCCAGAATATATCTATCCGTCATATGCATGGACAAATCCCACTCTTGATCCATGTGCCTC
AACACATACTTTCCAAACACTCAATGCCACCTTTATAGCCACCCAGTTACGGTGTGGTGTTTGATTTCATCAAAG
AATCCTTTCAGTGATGGTGATTAGCATGATCTCATGGTCGAAGAAATTAGGTTACTATGTATCAGAAAGCTTAT
AGCAA

>ChrUn:2000456-2004057

TAATGGACACGTTAATCAATCGGCCGTTTCCAGATTTTCTGGACTGTGGGATGTTACCGATTTGGACATGGTCT
ATGCCCCATGATCTCCCTGACATGCACTATATAAGTATCTGCGCTAAACCTAATCAGTAATCCACACAAGCTCCT
ATTTTCAGAACCTTGCGCTGTCACCTTCGTCTTCCCCATCACGTCTCGCGTCGTGCACTGCAGATCAGGATAGCA
GGCCTCCAGAACTACGCCTCTCGTAAACCTGCACGGATGAGGGGTGATCAGATTTTTGGGAGCGCTCAAGCGC
GACTACTGGCAACCACGACGTCGTCCGACGACGAGTTCCCAAACGACGACATCTTTCCGGACGTCACCAAAAA
TTACTGTTTTATTTATTAAAGTTGCACAACTTTGACATAGAACTTTTAACCTATGTGATTCATAATTTAGGTGGTT
TGAATTTATGAAAAAATCTAGCAACCAAGAACTAAATAAAAAGTGTAGTTATAGGAAGGACCAAAAAAGATGT
ACTTTGAAAAAAAATCCTGTCCAACTGGCCCATATATCTCGTCAATATGCATGCACATGAGGTAAAAACAAGCA
GAAAATCGAGGGATGGGGAAGGAGATTATTCAGAAGAGATAAATTTAAGAAAGGTACAAGACACAAACAAG
TGATATAACATGGTAAAGTTTTTATTCAGGTAAGCACTTATATGACACTAGGTTGCACACTTGCAATGCCTTTAT
TTTACTATACTAGTTCGCATTACACTCATTCACTCAGATGCTCTAACAGCCGAAGATGACCCCTCCTCGATGTCT
TGATAACTCTGGAAGCCTTTTTGGGTAAGTTTAGGTGTGAATGCACCAAACTCTTCTCCTCGGTTGTTTTTAAGG
TTTCGGGCTTCTTGTCTAGAAATGCGGTATGCATTGGCGAGGACATCGATAGGTAAGGCACGTAGGATGGAG
CTCTTTCCTGCGATGTGACTAACCATAGAGTTTGGGTTAGTCTTGAATGAAATGTATTGGCATCCTTCACGCTCT
GCCTTCTTGAGAACAACAAAGTGTTGTGGTACGATTAGCAGTTGTCCTCGACGAAGAATATCATTGAATACAGT
CTGGCCATTGTTGTTGACGACTTGAACTCGAGCATGTCCTTGGATCATGTAGATGACACTGTGCGCATTAATGT
TCCAGAATGGTGAAAGAATGGCATTCTGCAATAGTAATTTATAAGATCATATTATTAACCGATTGTTATGAATC
TTATACTACCTAAAAATAAGAGAGTCTATAGAGTGTAGTATCATATATACCTGGTATAGATTTACTCTTGTAGCA
CTCATTTGCACAATGTTAAGGATGGGGAAATTCTTGCTATTGAGACGTGTTATCCTGCCAGCACGTGGGTTGTA
TGTGTCGGCATGTTGGGGGTTTTCAATGTTTTTCCTTGCCTCCAATGAACAAAAGTTTTCCTCCAAACCGTTGAA
ACTTTGGTCCCATGACTGTCCTGTCTGGTATTGACTTGATTGTCCTCCTTGATATGGGGTTGATTTCCCTACCTG

GTATTGGGTTGATTGCCCTACCTGGTATTGGGTTGCCTGTCCTTCTTGTGTTTGAATTGGTTGGTAGACTTGCTG
CTCTCCAGGTACTTGTTGGGACACAATGGGCTTCAAGAATTGAAGGCCTTGACTCACACGAATTATCTCACCTC
TTTGGTCATTTTGACTTTGGATCCTTTGTGCAGCTTGTTGACTTATACCAAGGGCCTCACTAAGAAGTTGGACAC
TTAATCCACTGAATATGTTGTTTCCAGACTGTTGCTCTCTCTTGTTGTTACCAGCCAACAAGAACTCCTGACAATT
TATATGGATATAGTAAAACAAAGTTCATTAATACCCCAATAATATATACTTTTGTATAATTACCTTTTGTCTAGGT
TCAAGCTGATTAGCGTTGTTGTTTACATCAAAGACATAGATTGCTACAATCGGTGCATCACCGTCGTTGTAGCA
CCAGTGCACAATGCCTGCCGGAAGTGCAACAACATCTCCTTGTTTGAAGCGTTGAACTCTTTGGTGCTCATCCT
TAATAGTTTGGCTTTGGCTTTGACCTTGAGCAAACTGGGATTGATCAAATGGTTGGAACTGTTGTTGGAAGGTC
GCAGGACATCCAGGGAAAGTCAACCCCGTGAAACCTCTACCTAAAATAAAACATATTTGTTTGAATATTTAAAA
CTTAGGAATGGGTTAATATTTGGCACCACTAAACTCCTAAGTGTAGTATGACAATGCAATTCTATATTCACTTAG
ACACTAACCTTGGAGGATATACACCAAGGCAGGAGCGTTGTGGTATTGAGGCAGCACAAGGCCTTGAGGTTC
GATTACACGGCGGATGACGGATACACCGGTACAACGAAATTGCTCATTTTGCTCATCAAAGTACTCGGTGATA
CCTGCTTGTGACCTCACTTGTCGAAGTGGTTCAAATGCTTGTAGCCTATCAAATCTGCACCCACGTAAACCTCCT
TGACGAGAGCTTTGCCATGGAGTAGAGCTCTGGCCAAACAACTGAGCCATGGAACCATGGAACAAGAGGAAA
ATGCAAAAGTAAAACAACATTGATGGAAAACTAGTAGTTGCCATGTTTGTTTGAAGTGATTGTAGAAGATGGA
TTGGTGCTAGTTTCCTAATTTTAAGCTTGGTTTTATAGTCACAAAAAGTAGTTTAAGTTGTACATGGCGACATAA
TTGTTAACAGAAATAAGAAGATATGTATTGTGACTCAGATATGGTTTAGTATAACTTCGCCTCAAAGATACGTG
CTACTTTCCGCTAACAACTAGTGCTCACACATAAACTTAGCACCAAGTTTCGTACATAGATGATGTCCATTGTGT
GACGTTTAATTGAATTACTTGTACTTTCTACAAAAAAATTTGGCACTTGTGAGTCCTTGTCATGGTTTATTTACG
CTTTGGTCTATGAACAACGACGCATGTAAATACTTTTAGTATGTGGACAAACTTCCATGACTCGAGTCTATCCAT
ATAACATAAAGTGTAAAGTGGATGAGTCATGTTCATATCGACACACGAATACTTCCATGGATAAGAAACTTATG
ATGGTGAATAATCCGCCTAAGTAGAATACAAGATAGTGTACAAACAAAATTTGTTGCTTTACTTGATGCCAAAC
GTTGATAACGATATGTAAATTAGTTGTGAATTGCTATCTATTGGGCTCTCTGTCCAATTTGTATTGTCATGTGAA
GATACGAAAAGAAGCATTATAAGGGACTTAAAAGGAGAAGACAAAAATAGACGTCCAAAAATAAACTAAAAG
AGAATATATTGTTGGGCCACGAAAACTAGGGCAAAGATGTATAGTGGGTGCAGCTTACACCAACGAAGCCAA
GTTAATTATGAATGATCCCATCTTAACGTGGTGTGAACCAAGTCACAAAGTTGGAAATGTCCCTATCCTAGCGC
CCAGCTATACAAGGTGAAGATACCTAAAACAAGAGACATGATGTATTCTCTTGGAGGCCAGAAGGATAGCCTC
CCCTAATAAGTTCCCCGAATCGTGTCGATCAGCATGTGTTTTAG

>ChrUn:2025302-2027729

GGGACAGAGATTATTCAGAAGAGATAAATTTAAGAAAGGTACAAGACACAAACAAGTGATATAACATGGTAA
AGTTTTTATTTAGGTAAGCACTTATATGACACTAGGTTGCACACTTGCAATGCCTTTATTTTACTATACTAGTTCC
CATTATACTCATTCACTCGATGCTCTAACAGCCGAAGATGACCCCTCCTCGATGTCTTGATAACTCTGGAAGCCT
GTTTGGGTAAGTTTAGGTGTGAATGCACCAAACTCTTCTCCTCGGTTGTTTTTAAGGTTTCGGGCTTCTTGTCTA
GAAATGCGGTATGCATTGGCGAGGACATCGATAGGTAAGGCACGTAAGATGGAGCTCTTTCCTGCTATGTGAC
TAACCATGGAGTTTGGGTTAGTCTTGAATGAAATGTATTGGCATCCTTCACGCTCTGCCTTCTTGAGAACAACA
AAGTGTTGTGGTACGATTAGCAGTTGTCCTCGACGAAGAATATCATTGAATACAGTCTGGCCATTGTTGTTGAC
GACTTGAACTCGAGCATGTCCTTGGATCATGTAGATGACACTGTGCGCATTAATGTTCCAGAATGGTGAAAGA
ATGGCATTCTGCAATAGTAATTTATAAGATCATATTATTAACCGATTGTTATGAATCTTATACTACCTAAAAATA
AGAGTCTATAGAGTGTAGTATCATATATACCTGGTATAGATTTACTCTTGTAGCACTCATTTGTAGGGGTTCGT
AGCATGGAAAACAAAAATTTTCTATCGCATACAAACAAACAAGCCAAGATCTATCTACTAGATTGTAGCAACGA
GTCGATGGAATAACTCACCCTCGAAGATTCCAAAGTGTTTAACGAGAGCAGATTCTCGTGGGTGATGTAGTCG
TACTCTTTCCGATCTCAAGATCGCGTAGACGATCCTTCCGGCGCCGCGTTCCCGCAGCGCTCCCGCAGTCGGTC
ACACGTTCGGTACTCGACGAAGACCTTCCTCTCCCTTGTTCCAGCAGGCAGCGGAGGTTGTAGCACTCTCCTCG
CGATTCCGGCAGCACAACGGCGTGGTACCGGAGGTAGAGGGGAGATCCCGCAGGGCTTCGCCTTAAGCCGGT
GCGAGTATGGTGGAGCTTATGAGAGAGGCTATTAGTGGTCTAGTAATTAGACACACCAATTAGAGGGCTCTAA
ACCAATTAGAAGGCTCTAAACCGAAACAGCTCTAAACCGGGGTCTAGGTCTAGTTTCTAGTTTAGAGGATAGG
AACTTGTGTGTGTTGGGAGGGGAGCCCTGCCCCTCCCTTTATAGGTGGGAGGGCTGGCGCGCTAGGGGAGGG

AGGGAGTCCCTCCCCAACTCGGCCTAGGGCTGGCCGCCGGCTTGGGGACTCCTTCCCCAAGTCGGTCTCCTCTT
CTCCTCCTCTTCCCACTTTCCAAAACTGGCAGTTTGACTTTCAAACTAGCCGTTTGACTTTTCCAATTTAGGAATT
AAATAAACCCTTTATTTTGGACAGCGAGAACACTTAGTACATGATGCAATCATTCCATCTTGCAATGACACATTC
ATGTACCCCTTCCCCCCCGGAGTGCCGGACATTCCGGGACCTTCTAGAATGTTCCCGATGAAAACACCGGAACC
ATTCCCCAACTCCGAAATGGGACTTTCCTTATATGAATCTTATTCTCCGGACCATTCCGGATCACCTCGTGATAT
CCCGGATCCCATCCGAGACTCCGAACAACATTCGGTCTCTTTCTCATAATACCATAACTACTCAAACAACGTCGA
ATCCTTAAGTGTGTCTCCCTACGGTTCGTGAATTATGCGGACATGATCGAGACACTTCTCCGAACAATAACCAA
TAGAGGAATCTGGAAATTCATATGGCTCCCACATATTTCTACGATTACTTTGCGATCGAATGAACCACTTACATA
CGATACCAATTTCCCTTTGTCTCACGATATGTTACTTATCCGAGGTCATGATCGTCGGTATCTCTGCATACCTAG
TTCAACCTCGTTACTGATAAGTACTCTTTTCTCGTTCCCGTGATATGTGATCCCTTGTGACCAAGTCACATGCTTG
CAAGCTAATTGGATGACATTTCACCGAGAGGGCCCAGAGAATATCTATCCGTCATGTGGATGGACAAATCCCA
CTCTTGATCCATGTGTTTCAACCAATACTTTCCGAACACCCAATGCCACTTTTATAGCCACCCTGTTACGGTGTG
GTGTTTGATGACATCAAAGCATCCATCCAGTAAAGGCAATATGCATGATCTCATGGTCGAAGGAATTAGGTTA
CTATGTATCAGAAAGCATATAGCAATTGAACTATTGAAATGACTTGATCTTTATGCTACGCTTGTCTATAGGAG
TATGTCCACCATATCATTCTCCTAACGATATGATCTTGTTGTTGATAACATCTCAAT

>ChrUn:2033253-2035253

CTCGAAGATTCCAAAGTGTTTAACGAGAGCAGATTCTCGTGGGTGATGTAGTCGTACTCTTTCCGATCTCAAGA
TCGCGTAGACGATCCTTCCGGCGCCGCGTTCCCGCAGCGCTCCCGCAGTCGGTCACACGTTCGGTACTCGACG
AAGACCTTCCTCTCCCTTGTTCCAGCAGGCAGCGGAGGTTGTAGCACTCTCCTCGCGATTCCGGCAGCACAACG
GCGTGGTACCGGAGGTAGAGGGGAGATCCCGCAGGGCTTCGCCTTAAGCCGGTGCGAGTATGGTGGAGCTT
ATGAGAGAGGCTATTAGTGGTCTAGTAATTAGACACACCAATTAGAGGGCTCTAAACCAATTAGAAGGCTCTA
AACCGAAACAGCTCTAAACCGGGGTCTAGGTCTAGTTTCTAGTTTAGAGGATAGGAACTTGTGTGTGTTGGGA
GGGGAGCCCTGCCCCTCCCTTTATAGGTGGGAGGGCTGGCGCGCTAGGGGAGGGAGGGAGTCCCTCCCCAAC
TCGGCCTAGGGCTGGCCGCCGGCTTGGGGACTCCTTCCCCAAGTCGGTCTCCTCTTCTCCTCCTCTTCCCACTTT
CCAAAACTGGCAGTTTGACTTTCAAACTAGCCGTTTGACTTTTCCAATTTAGGAATTAAATAAACCCTTTATTTT
GGACAGCGAGAACACTTAGTACATGATGCAATCATTCCATCTTGCAATGACACATTCATGTACCCCTTCCCCCCC
GGAGTGCCGGACATTCCGGGACCTTCTAGAATGTTCCCGATGAAAACACCGGAACCATTCCCCAACTCCGAAA
TGGGACTTTCCTTATATGAATCTTATTCTCCGGACCATTCCGGATCACCTCGTGATATCCCGGATCCCATCCGAG
ACTCCGAACAACATTCGGTCTCTTTCTCATAATACCATAACTACTCAAACAACGTCGAATCCTTAAGTGTGTCTC
CCTACGGTTCGTGAATTATGCGGACATGATCGAGACACTTCTCCGAACAATAACCAATAGAGGAATCTGGAAA
TTCATATGGCTCCCACATATTTCTACGATTACTTTGCGATCGAATGAACCACTTACATACGATACCAATTTCCCTT
TGTCTCACGATATGTTACTTATCCGAGGTCATGATCGTCGGTATCTCTGCATACCTAGTTCAACCTCGTTACTGA
TAAGTACTCTTTTCTCGTTCCCGTGATATGTGATCCCTTGTGACCAAGTCACATGCTTGCAAGCTAATTGGATGA
CATTTCACCGAGAGGGCCCAGAGAATATCTATCCGTCATGTGGATGGACAAATCCCACTCTTGATCCATGTGTT
TCAACCAATACTTTCCGAACACCCAATGCCACTTTTATAGCCACCCTGTTACGGTGTGGTGTTTGATGACATCAA
AGCATCCATCCAGTAAAGGCAATATGCATGATCTCATGGTCGAAGGAATTAGGTTACTATGTATCAGAAAGCA
TATAGCAATTGAACTATTGAAATGACTTGATCTTTATGCTACGCTTGTCTATAGGAGTATGTCCACCATATCATT
CTCCTAACGATATGATCTTGTTGTTGATAACATCTCAATGTTCATGACAAAGAAACCAGATCATCAATCAACCAA
CAAGCTAGTGAAGTATAGAGGCCATACTAGGGACTTCAAATTAAGTTTACAAATCACACAAGTATTAATGGTTT
CCTTGTTAATACTATTATAGCATGAATGTAAACATTTATCATAAACACAAAGATATATAATAATAACTACATTTA
TTATTACCTCTAGGGCATATCTCCAACATCATTTGCACAATGTTAAGGATGGGGAAATTCTTGCTATTGAGACG
TGTTATCCTGCCAGCACGTGGGTTGTATGTGTCGGCATGTTGGGGGTTTTCAATGTTTTTCCTTGCCTCCAATGA
ACAAAAATTTTCCTCCAAACCATTGAAACTTTGGTCCCATGACTGTCCTGCCTGGTATTGACTTGATTGTCCT

>ChrUn:2034729-2037067

GAAGTATAGAGGCCATACTAGGGACTTCAAATTAAGTTTACAAATCACACAAGTATTAATGGTTTCCTTGTTAA
TACTATTATAGCATGAATGTAAACATTTATCATAAACACAAAGATATATAATAATAACTACATTTATTATTACCTC

TAGGGCATATCTCCAACATCATTTGCACAATGTTAAGGATGGGGAAATTCTTGCTATTGAGACGTGTTATCCTG
CCAGCACGTGGGTTGTATGTGTCGGCATGTTGGGGGTTTTCAATGTTTTTCCTTGCCTCCAATGAACAAAAATT
TTCCTCCAAACCATTGAAACTTTGGTCCCATGACTGTCCTGCCTGGTATTGACTTGATTGTCCTCCTTGATATGG
GGTTGATTGGCCTACCTGGTATTGGGTTGATTGCCCTACCTGGTATTGGGTTGCTTGTCGTTCTTGAGTTTGAAT
TGGTTGGTAGACTTGCTGCTCTACTGGTACGTGTTGGGACACAATGGGCTTCAAGAATTGAAGTCCTTGGCTCA
CACGAATTATCTCACCTCTTTGGTCATTTTGACTTTGGATCCTTTGTGCTGCTTGTTGACTTATACCAAGGGCCTC
ACTAAGAAGTTGGACACTTAATCCACTGAATATGTTGTTTCCGGACTGTTGCTCTCTCTTGTTGTTACCAGCCAA
CAAGAACTCCTAACAATTTATATGGACAGAGAAAAACAAAGTTCATTAATACCCCAATAATCTATACTTAACAA
AAAATTATAGTACTTCTAAACAGTTATATATATGTTATGTGGATTAATTTGTATAATTACCTTTTGTCTAGGTTCA
AGCTGATTAGCGTTGTTGTTTACGTCAAAGACATAGACAGCTACAATCGGTGCATCACCATCGTTGTAGCACCA
GTGCACAATGCCTGCCGGAAGTGCAACAACATCTCCTTGTTTGAAGCGTTGAACTCTTTGGTGCTCATCCTTAA
TAGTTTGCCTTTGGCTTTGACCTTGAGCAAACTGGGATTGATCAAATGGTTGGAACTGTTGTTGGAAGGTTGCA
GGGCATCCAGGGAAAGTCAACCCCGTGAAACCTCTTCCTAAAATAAGGCATATTTGTTTGAATATTAAAAACTT
ATGAATGGGTTAATATTTGGCACCACTAAACTCCTAAGTGTAGTATGATAGTGCAATTCTATATTCACTTAGATA
CTAACCCTGAAGGATGTACACCAAGGCAGGAGCGTTGTGGTACTGAGGTAGCACAAGGCCTTGAGGTTCGAT
TACACGGCGGATGACAGATACACCGGTACAACGAAATTGCTCATTTTGCTCATCAAAGAACTCAGTGATACCTG
CTTGTGACCTCACTTGTCGAAGTGGTTCAAATGCTTGTAGCCTATCAAATCTGCACCCACGTAAACCTCCTTGAC
GAGAGCTTTGCCATGGAGTAGAGCTCTGGCCAAACAACTGAGCCATGGATCCATGGAACAAGAGGAAAATGC
AAAAGTAAAACAACATTGATGGAAAACTAGTAGTTGCCATGTTTGTTTGAATTGATTTTAGAAGATGAATTGGT
GTTAGTTTCCTAATTTTAAGCTTGGTTTTATAGTCACAAAAAGTAGTTTAAGTTGTACATGGTGACATAATTGTT
AACAGAAATAAGAAGATATGTATTGTGACTCGGATATGGTTTGATATAACTTCGCCTCAAAGATATGTGTTACT
TTTCGCTAACAACGAATGCTCAAACATAAACTTAGCACCAAGTTTCGTACATAGCTGATGTCCATTGTGTGACG
TTTAATTGAATACATAATAGTAATTGCACTTTCTACACACAATTTTTTTTGGCACTTGTGACTCCTTGTCATGGTT
TATTTATGCTTTGGTCCATGAACAACGACGCACGTAAATAGTTTTAGTATGTGGCCAAACTTCCATGACTCGAG
TCTATCCATATAACATATAAAGTGTAAAGTGGATGAGTCATGTCCATATCGACACACGAATACTTCCATGGATA
AGAAACTTATGATGGTGAATAATCCGCCTAAGTAGAATACAAGATAGTGTACAAACAAAATTTGTTACTTTACT
TGATGCCAAAAAATTGATAAAGATATGTAAATGAGTTGTGAAGTGCTATCTATTGGACTCTCGCTCCAATCTATA
TTGTTATGTAAAGATACAAAAAGAAGCATTAGAAGGGACTTAAAAGGAGAAGACAGAAATAGACGTCCGAAA
CTATGGCACTGCCAACGTCCAGTGGCGGATCCCAGGGGGGGGGGGGGGGGGGGCAGCGTTCTCTCCGCGGGC
CAGCGCCTCGCGCGCACTGTTCATAAGCAGTGAGCGTGG

>ChrUn:2043136-2045336

GATATAACATGATAAAGTTTTATTCAGGTAAGCACTTATATGACACTAGGTTGCACACTTGCAATGCCTTTATTT
TACTATACTAGTTCCCATTACACTCATTCACTTGGATGCCCTAACAGCCGAAGATGACCCCTCCTCGATTTCTTG
GTAACTCTGGAAGCCCGTTTGGGCAAGTTTAGGAGTGAATGCACCAAACTCTTCTCCCCGGTTGTTTTTGAGGT
TTCGGGCTTCTTGTCTAGAAACACGGTATGCATTGGCAAGGACATCGACAGGTAAGGCACGTAGAATGGAGCT
CTTTCCTGTGATGTGACTAACCATGGAGTTTGGATTAGTCTTAAATGAAATGTATTGGCATCCTTCACGCTCTGA
CTTCTTGATAACAACAAAGTGTTGTGGTATGATTAGCAGTTGTCCTCGACGAAGAATGTCATTGAATACGGTCT
GTCCATGGTTGTTGACGATTTGAACTCAGAGCATGTCCTTGGATCATGTAGATGACACTGTGCGCATTAATGTT
CTAGAATGGTGAAAGAATGGCATTCTGCAACCATATTATTTGAAAGCCATATTATTTACCGGTTTTTATGAATCA
TAGACTCTAAAAATAAGAGTGTGTAGCGCATAGTGTCATATATACCTGGTATAGATTTACTCTTGTAGCACTTA
TTTGCACGAGGTTAAGGATGGGGAAATTCTTGCTATTTTGAGACGTGTTATCCTGCCAGCACGTGGGTTGTAT
GTGTCGGCATGTTGGGGGCTTTCAATGTTTTTCCTTGCCTCCAATGAACAAAAATTCTCCTCCAAACCATTCAAA
CTTCGGTCGGATGACTATCCTTCCTGGTATTGACTTGATTGTCCTTCTTGATATGGGGTTGATGGTCCTACCTGG
TATTGGGTTGATTGCCTTACCTTGTATTGGGTTGATTGTCCTTCTTGAGTTTGAATTGGTTGGTAGACTTGCTGC
TCTACTGGTACTTCTTGGGACACAATGGGCTTCAAGAATTGAAGGCCTTGACTCACATGAATTATGTCACCTCTT
TGGTCATTTTGACTTTGGATTCTTTGTGCTGTTTGTTGACTTATACCAAGGGCCTCACTAAGAAGTTGGACACTT
AATCCTGTGAATATGTTGTTTATAAACTGTTGCTCTCTCTTGTTGTTACCAGCCAACAAGAACTCCTGAAATTTG

ATATGGATGGAATAAAACGAAGTTCATTAATACCTCAATAATATGTACTTAACAAATAAAATTTATATTACTTGT
AAAAAATTATATATCTTTTAGGTGGATTAATTTGTATAATTACCTTTTGTCTAGGTTCAAGCTGATTAGCATTATT
GTTTACGTCAAAGACATAGACAGCTACAAGCGCATCGCCATCGTTGTAGCTCCAGTGTACAATGCCTGCCGGA
AGTGCAACAACATCTCCTTGTTTGAAATGTTGAACTCTTTGGTGCTCATCATTAATAGTTTAGCTTTGGCTTTGA
CGTTGAGCAAACTGGGATTGATCAAATGGTTGGAACTGTTGTTGGAAGGTCCCAGGGCATCCAGGGAAAGCC
AACCCCGTGAAACCACTACCTAAAATGAAACATATTTTTTTGAATATTAAAAATTTATGGATGGGTTAATATTTG
GCACAACTAAACTCCTAATTATAGTATAACAATGCAATTCTATATTTAGTTAGACAATAACCTTGAAGGATGTAC
ACCAAGGCAGGAGCATTGTGGTATTGAGGTAGCAAGAGGCCTTGAGGTTCGATTACACGATGAATGACAGAT
ACACCGGTACAACGAAATTGCTCATTTTGCTCATCAAATTACTCTGTGGTACCCGCTTGTGACTTCACTTGTCGA
AGTGGTTCAAATGATTGTAGCCTATCAAATCTGCACCCACGTAAACCTCCTTGATGAGAGCTTTGCCACGGAGT
AAAGCTCTGGCCAAACAACTGAGCCATGGATCCATGGAACAAGATAAAAACGCAAAAGTAAAACAAGATCGA
TGGAAAACTAGTAGTTGCCATGTTTGTTTGAAGTGATTGTTGGTGGTAGATTGGTGCTAGTTTCCTAATTTTAA
GCTTGGTTTTATAGCCGCAAAAAGTAGTTTAGGTTGCACATGGTGACGTAATTGTTAAGAGAGATAAGAAGAT
ATGCACTGTGACTAGGACATGGTTTGAATTTTGTATATATATATATA

>ChrUn:52047208-52049208

CTTGATTAATGAAAACATCCTTGGCAAATGCTTTCGCAGTTGTTCGTCTTTCATAAATCCAAGAATTTCACCTCT
GACTATGAAATACGAATGCCCCCGACTGTCCCTATTAATCATTACTCCGATCCCGAAGGCCAACACAATAGGAC
CGGAATCCTATGATGTTATCCCATGCTAATGTATCCAGAGCGATGGCTTGCTTTGAGCACTCTAATTTCTTCAAA
GTAACGATGCCGGACACACGACCCGGCCAATTAAGGCTAGGAGCGCATCGCCGGCTGAAGGGTCGAGTAGGT
CGGTGCTCGCCGTGAGGCGGACCGGCCGACCCGGCCCAAAGTCCAACTACGAGCTTTTTAACTGCAACAACTT
AAATATACGCTATTGGAGCTGGAATTACCGCGGCTGCTGGCACCAGACTTGCCCTCCAATGGATCCTCGTTAAG
GGATTTAGATTGTACTCATTCCAATTACCAGACACTAATGCGCCCGGTATTGTTATTTATTGTCACTACCTCCCC
GTGTCAGGATTGGGTAATTTGCGCGCCTGCTGCCTTCCTTGGATGTGGTAGCCGTTTCTCAGGCTCCCTCTCCG
GAATCGAACCCTAATTCTCCGTCACCCGTCACCACCATGGTAGGCCCCTATCCTACCATCGAAAGTTGATAGGG
CAGAAATTTGAATGATGCGTCGCCGGCACGAGGGCCGTGCGATCCGTCGAGTTATCATGAATCATCGGATCAG
CGGGCAGAGCCCGCGTCAGCCTTTTATCTAATAAATGCGCCCCTCCCAGAAGTCGGGGTTTGTTGCACGTATTA
GCTCTAGAATTACTACGGTTATCCGAGTAGCACGTACCATCAAACAAACTATAACTGATTTAATGAGCCATTCG
CAGTTTCACAGTTCAAATTGGTTCATACTTGCACATGCATGGCTTAATCTTTGAGACAAGCATATGACTACTGGC
AGGATCAACCAGGTAGCGAGTCCTCTTCGACGTCCAGCGACGGTAGTCATCCGCCCTTGCAGGCAGACAACAG
CCGGTCCGGTTGTCGCAATTCATCGGGACTAAGCTTTCCTTCACCTACTAGAGACATTTCCAGCGTGTTGCGTCT
CCTACCACAAACTGTGGGGAATACATGCAACACCCCGTACTAAGTCCGTTCTGATCCAGATGGCTTCTTTTGAG
GTGGGTCAACAGCCGTTCCGGTTGTCGCTATTAAGCAGGACTAAGCTTTCCTTCTCCTACTAGAGACATTTCCA
GCGTGATGCGTCTCCTACCACAAAATGTGGGGAATACATGCAACACCCCGTACTAAGACCGTTCTGATCCAGA
GAGCTTGTTTTGAGGTCAATAGCCGGTCCGGTTGTCGCACTTGAACGGGACTAAGCTTTCCTTTTCCTACTAGA
GAGGTTTCCAAGCGTGTTGCTTCTCCCCACAAACTGTGGGAAATACATGCAACACACCGTACTACCGCCGTTCA
GATCCAGAAAGCTTCTTTTCAGGTGGGACTAGCGACAACCAGAAGGGCTATTAACCTTTATCGTGAGCGGCAA
CCAGAAGGGCCATTAACCATTATCGTGAGCGGCATCCGAAACCAATAGCGAGAGCGAGGCTTCCTTGATAGCA
CCGAGCACAAAACGTGCCAGTACCACGAGGTAACGCCGCAAGCGCCATTAGGCCGCAGCGGAACACCCAGAG
GTGTCCGCCGCGAGGCAAGCGTGTACAAAGCACCACTTCCCGTAGGTCGGGTACATGCACGCAAGCACTTGA
AAGGCAACAGTAACATAAGCCACACGAATGACGGGACACGGCGTCGCTAGTCGGCCGCACGAGGACGGGGG
ATCTAACAACAGACACGGGTCCAAAGCTACTCATCCGCTCGCGTATCCACATCGGTCAAGCCAACCAAGCCTCC
CCCCACGCATAGCACGGGGGAATGGCCTGCACGAAGGCGTCCTGAGTGTCCACGGCGCGCGGGTGTTACACC
AATAACGATACCGT

>ChrUn:56166014-56168014

ACCATGGTGGTGACGGGTGACGGAGAATTAGGGTTCGATTCCGGAGAGGGAGCCTGAGAAACGGCTACCAC
ATCCAAGGAAGGCAGCAGGCGCGCAAATTACCCAATCCTGACACGGGGAGGTAGTGACAATAAATAACAATA

CCGGGCGCATTAGTGTCTGGTAATTGGAATGAGTACAATCTAAATCCCTTAACGAGGATCCATTGGAGGGCAA
GTCTGGTGCCAGCAGCCGCGGTAATTCCAGCTCCAATAGCGTATATTTAAGTTGTTGCAGTTAAAAAGCTCGTA
GTTGGACTTTGGGCCGGGTCGGCCGGTCCGCCTCACGGCGAGCACCGACCTACTCGACCCTTCAGCCGGCGAT
GCGCTCCTAGCCTTAATTGGCCGGGTCGTGTGTCCGGCATCGTTACTTTGAAGAAATTAGAGTGCTCAAAGCA
AGCCATCGCTCTGGATACATTAGCATGGGATAACATCATAGGATTCCGGTCCTATTGTGTTGGCCTTCGGGATC
GGAGTAATGATTAATAGGGACAGTCGGGGGCATTCGTATTTCATAGTCAGAGGTGAAATTCTTGGATTTATGA
AAGACGAACAACTGCGAAAGCATTTGCCAAGGATGTTTTCATTAATCAAGAACGAAAGTTGGGGGCTCGAAG
ACGATCAGATACCGTCCTAGTCTCAACCATAAACGATGCCGACCAGGGATCGGCGGATGTTGCTTATAGGACT
CCGCCGGCACCTTATGAGAAATCAAAGTCTTTGGGTTCCGGGGGGAGTATGGTCGCAAGGCTGAAACTTAAA
GGAATTGACGGAAGGGCACCACCAGGCGTGGAGCCTGCGGCTTAATTTGACTCAACACGGGGAAACTTACCA
GGTCCAGACATAGCAAGGATTGACAGACTGAGAGCTCTTTCTTGATTCTATGGGTGGTGGTGCATGGCCGTTC
TTAGTTGGTGGAGCGATTTGTCTGGTTAATTCCGTTAACGAACGAGACCTCAGCCTGCTAACTAGCTATGCGGA
GCCATCCCTCCGCAGCTAGCTTCTTAGAGGGACTATCGCCGTTTAGGCGACGGAAGTTTGAGGCAATAACAGG
TCTGTGATGCCCTTAGATGTTCTGGGCCGCACGCGCGCTACACTGATGTATTCAACGAGTATATAGCCTTGGCC
GACAGGCCCGGGTAATCTTGGGAAATTTCATCGTGATGGGGATAGATCATTGCAATTGTTGGTCTTCAACGAG
GAATGCCTAGTAAGCGCGAGTCATCAGCTCGCGTTGACTACGTCCCTGCCCTTTGTACACACCGCCCGTCGCTC
CTACCGATTGAATGGTCCGGTGAAGTGTTCGGATCGCGGCGACGGGGGCGGTTCGCCGCCCCCGACGTCGCG
AGAAGTCCATTGAACCTTATCATTTAGAGGAAGGAGAAGTCGTAACAAGGTTTCCGTAGGTGAACCTGCGGAA
GGATCATTGTCGTGACCCTGACCAAAACAGACCGAGCACGCGTTATCTATTCCTACTGAGTGGCGGCACCGTC
GTCGCTCAGCCAAATCCTCGATAACCTCCTCTCCTCGGAGTGGGGGCTTGGGGTAAAAGAACCCACGGCGCCG
AAGGCGTCAAGGAACACTGTGCCTAGCTTGGGGACACGACTGGCTTGCTGGCCGCTCCCCTTGCTGCAAAGCT
ATTTAATCCACACGACTCTCGGCAACGGATATCTCGGCTCTCGCATCGATGAAGAACGTAGCGAAATGCGATA
CCTGGTGTGAATTGCAGAATCCCGCGAACCATCGAGTCTTTGAACGCAAGTTGCGCCCGAGGCCATTCGGCCG
AGGGCACGCCTGCCTGGGCGTCACGCTAAACACGCTCCCAACCCCTTACGGGGGAACAGGATGCGGCATTTG
GCTCCCCGTCACCCAAGGGCGGTGGGCCGAAGATATGGCTGCCGGCGCATCGTGTCGGACACAGCGCGTGGT
GAGCGTCCTCGCTATACTTACCGCAGTGTCTCC
