# Supplementary material for: Analysis of oat seed transcriptome with regards to proteins involved in celiac disease
Source: Sci Rep. 2022 May 23;12:8660. doi: 10.1038/s41598-022-12711-6 (PMC9127096; doi:10.1038/s41598-022-12711-6)
Supplement: Supplementary file 5 — Supplementary Information 5. [file 41598_2022_12711_MOESM5_ESM.pdf]

Supplementary material:

Supplementary File S1 - Reference sequence in fasta format extracted from oat reference sequence PepsiCo OT3098 using target loci bed file

Supplementary Table S1 - List of 213 contigs of study target loci with mapping, blast and presence in six oat varieties information

Supplementary Figure S1 - Molecular Phylogenetic analysis of avenins found in public databases including those of reference OT3098 by Maximum Likelihood method based on the Jukes-Cantor model The tree with the highest log likelihood (-7745.16) is shown. The analysis involved 80 nucleotide sequences; there were a total of 1219 positions in the final dataset

Supplementary Figure S2 - Molecular Phylogenetic analysis of globulin sequences of the reference OT3098 and all six oat varieties by Maximum Likelihood method

Supplementary Figure S3 - Molecular Phylogenetic analysis of avenin, glutenin, gliadin, hordein and secalin epitopes by Maximum Likelihood method with bootstrap value 1000
