# Supplementary material for: Analysis of oat seed transcriptome with regards to proteins involved in celiac disease
Source: Sci Rep. 2022 May 23;12:8660. doi: 10.1038/s41598-022-12711-6 (PMC9127096; doi:10.1038/s41598-022-12711-6)

Supplementary Figure S1 - Molecular Phylogenetic analysis of avenins found in public databases including those of reference OT3098 by Maximum Likelihood method based on the Jukes-Cantor model The tree with the highest log likelihood (-7745.16) is shown. The analysis involved 80 nucleotide sequences; there were a total of 1219 positions in the final dataset

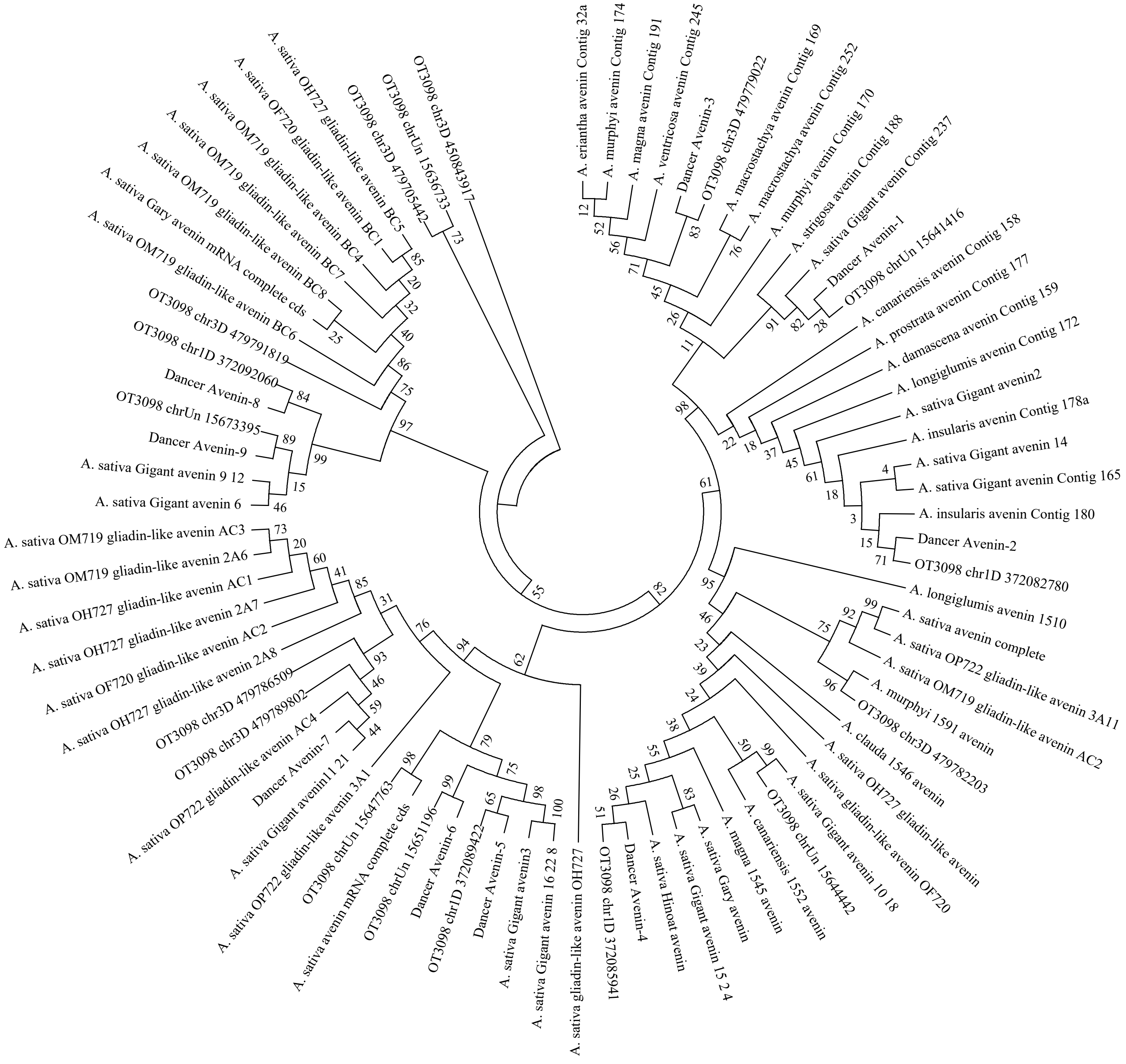

Supplement: Supplementary file 1 — Supplementary Information 1. [file 41598_2022_12711_MOESM1_ESM.pdf]
